# Integrated metabolomic, nanoformulation, and network pharmacology approach reveals multifunctional bioactivities of an *Ocimum sanctum* nanoemulsion

**DOI:** 10.3389/fbioe.2026.1731720

**Published:** 2026-03-20

**Authors:** Mervat S. Mohamed, Amnah Obidan, Hajer Alfarteesh, Seham O. Alsulami, Rehab Almassabi, Noor Alqurashi, Salma Saddeek, Raneen Salem Alsariei, Ayat G. Ali

**Affiliations:** 1 Department of Chemistry, Biochemistry Specialty, Faculty of Science, Cairo University, Giza, Egypt; 2 Department of Biochemistry, Faculty of Science, University of Tabuk, Tabuk, Saudi Arabia; 3 Biology Department, King Khalid University, Abha, Saudi Arabia; 4 Department of Chemistry, Faculty of Sciences, Universty of Hafr Al Batin, Hafr Al Batin, Saudi Arabia; 5 University of Tabuk, Tabuk, Saudi Arabia; 6 Department of Biochemistry, El Sahel Teaching Hospital, GOTHI, Cairo, Egypt

**Keywords:** antibacterial, anticancer, anti-inflammatory, metabolomics, nanoemulsion, network pharmacology, Ocimum sanctum

## Abstract

**Background:**

Although Ocimum sanctum (Holy Basil) exhibits broad pharmacological potential, its therapeutic application is constrained by poor solubility, instability, and limited bioavailability. Previous nanoemulsion studies have not integrated comprehensive metabolomic profiling with mechanistic and multi-bioactivity validation. This study developed and characterized an O. sanctum nanoemulsion to overcome these barriers and enhance biological activity.

**Methods:**

The ethanolic extract was profiled by GC–MS and LC–QTOF–MS/MS, identifying diverse phytochemicals. The optimized oil-in-water nanoemulsion (NE) was prepared using high-energy emulsification followed by high-pressure homogenization and ultrasonication. Physicochemical characterization included droplet size, polydispersity index (PDI), zeta potential, entrapment efficiency, and release profile. Biological activities were evaluated through cytotoxicity assays against Caco-2, HepG2, MDA-MB-231, and A549 cancer cell lines, antibacterial screening, antioxidant testing, and anti-inflammatory validation in LPS-stimulated RAW 264.7 macrophages. Network pharmacology analysis was performed to predict potential anti-inflammatory targets associated with the identified metabolites.

**Results:**

The optimized NE displayed nanoscale spherical droplets (51–73 nm), narrow polydispersity (PDI = 0.264), high negative zeta potential (−42.1 mV), and strong entrapment efficiency (96.2 ± 3.1%). A biphasic release profile reached ∼60% over eight days. The NE showed potent cytotoxicity against Caco-2, HepG2, MDA-MB-231, and A549 cells (IC_50_ = 13–25 μg/mL), demonstrating higher cytotoxic potency than the crude extract (∼200 μg/mL) while maintaining lower toxicity in normal fibroblasts (IC_50_ = 102 μg/mL). Antibacterial screening revealed inhibition zones up to 14.8 ± 0.3 mm, and antioxidant testing demonstrated enhanced radical-scavenging activity (SC_50_ = 16.4 μg/mL) compared with the crude extract (20.8 μg/mL). Network pharmacology analysis of identified metabolites predicted anti-inflammatory targets, highlighting AKT1, STAT3, PTGS2, and TLR4 as key regulators. Guided by these predictions, anti-inflammatory efficacy was experimentally validated in LPS-stimulated RAW 264.7 macrophages, where both the extract and NE significantly reduced TNF-α and IL-6 secretion (p < 0.0001), with stronger suppression by the NE.

**Conclusion:**

Collectively, this metabolomics-guided O. sanctum nanoemulsion represents a promising plant-derived nanosystem. This preliminary study demonstrates its potential anticancer, antibacterial, antioxidant, and anti-inflammatory activities in vitro. However, further detailed investigations, including more extensive in vitro studies as well as in vivo evaluations, are required to better elucidate its mechanisms, safety, and potential biological applications.

## Graphical Abstract

**Figure:**
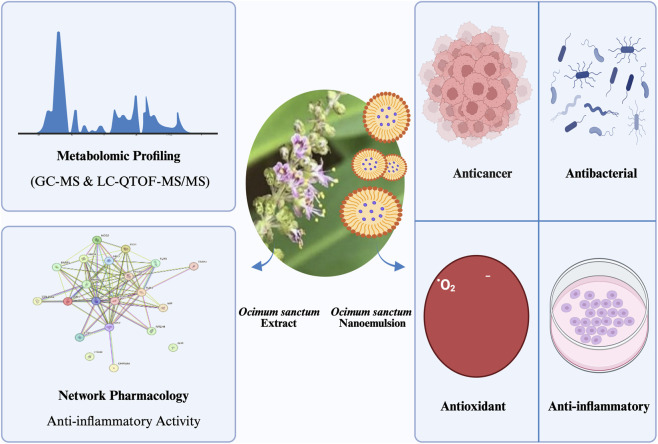

## 1 Introduction

Nanoemulsion (NE) is a nanoscale colloidal dispersion of two immiscible liquids—typically oil and water—stabilized by surfactants, with droplet diameters generally ranging from 20 to 200 nm. They are kinetically stable, optically translucent or transparent, and possess a high surface area and interfacial energy that enhance interactions with biological systems (Preeti et al., 2023). Unlike conventional emulsions, their smaller droplet size and Brownian motion minimize sedimentation and creaming, resulting in greater kinetic stability (Ezike et al., 2023).

Bioactive phytocompounds have long been recognized as valuable agents in human health management and remain key sources for modern drug discovery. However, their therapeutic application is restricted by limitations such as low solubility, instability, poor bioavailability, and short biological half-life. Nanoformulation technologies—particularly nanoemulsions—have emerged as promising delivery strategies to overcome these issues by improving solubility, protecting labile constituents, and enhancing pharmacokinetic behavior (Han et al., 2022). Previous studies have demonstrated that NE-based delivery systems exhibit superior antimicrobial, antioxidant, antibiofilm, and anticancer activities compared with conventional formulations (da Silva et al., 2023; Shehabeldine et al., 2023).

Nanoemulsions have been widely investigated for anticancer applications. Many phytochemicals with cytotoxic, pro-apoptotic, and anti-proliferative activities face limitations in achieving effective intracellular concentrations. The nanoscale size of NEs facilitates endocytosis and close interaction with cancer cell membranes, while the oil phase enhances the solubilization of hydrophobic compounds. These characteristics contribute to improved cellular uptake, modulation of signaling pathways, and enhanced induction of apoptosis or cell cycle arrest in cancer cells (Al-Balushi et al., 2025).

Nanoemulsions have shown considerable promise in enhancing anti-inflammatory effects by improving absorption, stabilizing labile actives, and enabling controlled delivery. In addition, by protecting sensitive compounds from degradation and supporting sustained release, NEs extend the duration of anti-inflammatory actions, offering a more efficient and targeted therapeutic approach (Chatzidaki and Mitsou, 2025). Previous studies have shown that NEs exert anti-inflammatory effects in animal models. They can reduce paw edema (Souza et al., 2023; Gavzan et al., 2025) and downregulate pro-inflammatory cytokines such as IL-1β (Souza et al., 2023). Compared with free agents, nanoemulsified formulations frequently produce stronger and longer-lasting suppression of inflammation (Chatzidaki and Mitsou, 2025; Souza et al., 2023; Gavzan et al., 2025). Building on these general advantages of NEs, it is valuable to explore their application to medicinal plants with established pharmacological activities.

Within this context, *O. sanctum* (*Ocimum sanctum*) stands out as a medicinal herb of growing pharmacological interest. Reports highlight its anticancer, antimicrobial, antioxidant and anti-inflammatory activities (Dharsono et al., 2022; Arya et al., 2024). These properties are attributed to its bioactive constituents, including key anticancer compounds such as eugenol, ursolic acid, rosmarinic acid, apigenin, and luteolin, which act through antioxidant, pro-apoptotic, anti-proliferative, and anti-metastatic mechanisms (Hasan et al., 2023). Its essential oil components, such as camphor, eucalyptol, β-caryophyllene and bisabolenes further support anticancer, antimicrobial, and antioxidant effects (Hasan et al., 2023). The anticancer potential of *O. sanctum* has been well documented in both *in vitro* and *in vivo* models, where its extracts and bioactive constituents have demonstrated cytotoxic, anti-proliferative, pro-apoptotic, and cell cycle–arresting effects (Arya et al., 2024; Perna et al., 2022).

Beyond its anticancer activity, *O. sanctum* has also demonstrated notable anti-inflammatory effects, as reported in experimental studies. In macrophage models, ethanolic extracts reduced TNF-α, IL-6 and IL-1β expression while downregulating iNOS and COX-2, leading to decreased NO and PGE_2_ release (Srichok et al., 2022). Under hyperglycemic *in vitro* conditions, the extract inhibited protein denaturation, displayed anti-proteinase activity, and exerted antioxidant effects; molecular docking analyses further suggested potential interactions with redox-related enzymes (Anwar et al., 2024). *In vivo*, oral administration following photothrombotic stroke modulated brain and plasma lipid profiles toward recovery-associated patterns, consistent with neuroprotective and inflammation-regulating effects (Yadav et al., 2023).

Nanoemulsion-based delivery systems have therefore attracted increasing attention in addressing formulation challenges associated with phytochemical-based therapeutics (Chatzidaki and Mitsou, 2025). Although *O. sanctum* has been extensively studied, most research has focused on crude extracts or isolated compounds, with relatively few investigations into advanced delivery systems (Flegkas et al., 2019). For example, flow cytometry analyses have demonstrated apoptosis and cell-cycle arrest in response to *O. sanctum* constituents, however, such mechanistic evaluations remain limited for NE-based formulations (Manaharan et al., 2016).

Although a few studies have explored *O. sanctum* nanoformulations, these investigations generally focused on basic characterization or evaluation of single biological activities and did not incorporate comprehensive metabolomic profiling, mechanistic network analysis, or multi-bioactivity validation. In contrast, the present study uniquely integrates metabolomics-guided phytochemical characterization, nanoemulsion formulation, network pharmacology prediction, and experimental validation of anticancer, antibacterial, antioxidant, and anti-inflammatory activities, providing a more holistic and mechanistic understanding of *O. sanctum*’s therapeutic potential.

Addressing a key research gap and moving beyond conventional extract-based studies, the present work aimed to develop and characterize a NE formulation of *O*. *sanctum*—a plant long used in herbal medicine—to enhance its therapeutic performance. The phytochemical composition was comprehensively analyzed using GC–MS and LC–QTOF–MS/MS techniques, while anticancer, antibacterial, antioxidant, and anti-inflammatory activities were systematically evaluated in comparison with the crude extract. To elucidate the underlying mechanisms, network pharmacology analysis was employed to predict molecular targets related to the anti-inflammatory effects, followed by experimental validation in LPS-stimulated RAW 264.7 macrophages *via* ELISA quantification of TNF-α and IL-6. Collectively, this integrated approach highlights the superior biological efficacy of the nanoemulsion over the crude extract and supports the potential of *O. sanctum* as a multifunctional, plant-derived nanoformulation with dual anticancer and anti-inflammatory activities, along with notable antibacterial effects.

## 2 Materials and methods

### 2.1 Preparation of plant extract (maceration method)

Dried aerial parts (leaves and stems) of *O. sanctum* were purchased from Frontier Co-op (Product Code: FRO-04440; UPC: 089836044402; United States). The plant material (100 g) was washed, shade-dried, and ground into a coarse powder. The powder was macerated with 70% ethanol under continuous stirring at 50 °C for 4 h. After completion of the maceration process, the extract was filtered, and the filtrate was concentrated using a rotary evaporator (D-Lab, United States) to obtain the dried ethanolic extract.

### 2.2 Phytochemical profiling

#### 2.2.1 Gas chromatography–mass spectrometry (GC-MS)

Volatile constituents of the *O. sanctum* extract were analyzed using GC–MS following solid-phase microextraction (SPME). Briefly, aliquots of the plant extract were placed in sealed glass vials and exposed to SPME fibers for 20 min at 50 °C to allow adsorption of volatile components onto the fiber surface. After extraction, the fibers were immediately inserted into the GC injector port for thermal desorption.

The analysis was performed using an Agilent 7890B GC system coupled to a 5977A mass selective detector (Agilent Technologies, United States). Separation was achieved on an HP-5MS capillary column (30 m × 0.25 mm, 0.25 µm film thickness) using hydrogen as the carrier gas at a constant flow rate of 1.1 mL/min under splitless injection mode. The oven temperature was programmed as follows: initially held at 50 °C (0 min), ramped to 200 °C at 5 °C/min, then further increased to 280 °C at 20 °C/min, and held for 6 min at the final temperature. The injector and detector temperatures were maintained at 250 °C and 320 °C, respectively. The mass spectrometer was operated in electron ionization (EI) mode at 70 eV, scanning a mass range of m/z 50–600. The ion source and quadrupole temperatures were set to 230 °C and 150 °C, respectively.

Identification of volatile compounds was accomplished by comparing their mass spectra with those available in the NIST MainLib, RepLib, and Wiley Registry libraries.

#### 2.2.2 LC-QTOF–MS/MS analysis

The ethanolic extract of *O. sanctum* was analyzed using a XEVO G3 Q-TOF mass spectrometer (Waters Corporation, Milford, MA, United States) equipped with UNIFI software (v.3.3.1). Compound identification was assisted by the Waters Traditional Medicine Library (June 2023 release). Chromatographic separation was performed with mobile phase A (water with 0.1% formic acid) and mobile phase B (acetonitrile with 0.1% formic acid) under the following gradient conditions: 0–5 min, 30% B; 5–15 min, linear to 70% B; 15–22 min, linear to 90% B; 22–25 min, hold at 90% B; 25–32 min, re-equilibrate to 10% B. The flow rate was 0.4 mL/min, and UV monitoring was carried out at 254 nm.

The mass spectrometer was operated in both positive and negative ionization modes with the following parameters: capillary voltage, 3.0 kV; sampling cone, 40 V; desolvation temperature, 550 °C; desolvation gas flow, 1000 L/h. Data were acquired in MSe mode over an *m/z* range of 100–1,200 using a low collision energy of 6 eV and a ramped high collision energy of 20–40 eV. Compound identification was based on accurate mass measurements and MS/MS fragmentation patterns obtained in both positive and negative ionization modes. Compound identities were further supported by comparison with previously reported phytochemical profiles of species belonging to the Lamiaceae family.

### 2.3 Nanoemulsion preparation

The high-energy emulsification method was employed to prepare the *O. sanctum* NE using a mixed oil phase composed of jojoba oil and tea tree oil as carrier oils, with slight modifications (Mohamed et al., 2025). The oil phase contained a total of 7% w/w mixed oils (4.5% w/w jojoba oil and 2.5% w/w tea tree oil) and 6% w/w *O. sanctum* plant extract. This oil–extract mixture was combined with a surfactant blend consisting of 8.0% w/w Tween 80, 1.0% w/w Span 80, 0.005% w/w sodium lauryl sulfate, and 0.10% w/w lecithin. The components were stirred using a magnetic stirrer (Cimarec+™ Stirring Hotplate, Thermo Fisher Scientific, United States) at ambient temperature until a homogeneous mixture was obtained. Deionized distilled water (DDH_2_O) was then added as the continuous phase (q.s to 100% w/w). The resulting pre-emulsion was subjected to high-pressure homogenization, followed by ultrasonication at 50 W for 5 min (VCX 500, Sonics, United States) in an ice bath to prevent heat buildup. The cavitation produced during ultrasonication effectively reduced droplet size, yielding a stable, isotropic oil-in-water nanoemulsion suitable for subsequent biological evaluation.

### 2.4 Physicochemical characterization of NE

#### 2.4.1 Transmission electron microscopy (TEM)

High-resolution TEM was performed using a JEOL JEM-2100 microscope operated at 200 kV. For sample preparation, a drop of the NE suspension was deposited onto a Formvar carbon-coated copper grid (300 mesh) and allowed to air dry at room temperature under ambient conditions before imaging.

#### 2.4.2 Particle size, polydispersity index (PDI), and zeta potential

The particle size and PDI of the NE samples were determined by dynamic light scattering (DLS) using a Zetasizer Nano ZN (Malvern Panalytical Ltd., United Kingdom). Measurements were carried out at a fixed scattering angle of 173° and a temperature of 25 °C, with each sample analyzed in triplicate. Zeta potential was measured on the same instrument under identical conditions. The pH and osmolarity of the *O. sanctum* NE formulations were measured in triplicate at 25 °C using a digital pH meter (Mettler Toledo, Switzerland) and an osmometer (Osmomat 030, Gonotec, Germany), respectively.

#### 2.4.3 Fourier Transform Infrared spectroscopy (FTIR)

The functional groups of the *O. sanctum* NE were characterized using a Bruker Alpha II FTIR spectrometer (Bruker, Germany) equipped with an attenuated total reflectance (ATR) accessory. Spectra were recorded in the range of 400–4,000 cm^-1^ at room temperature. Three sample conditions were analyzed: (A) *O. sanctum* extract nanocapsulated within the NE, (B) blank NE without the extract, and (C) pure *O. sanctum* extract. A small amount of each sample was directly applied onto the diamond crystal of the ATR unit to ensure optimal contact. The ATR-FTIR technique enables direct analysis of liquids, solids, and pastes without extensive sample preparation. The resulting spectra were interpreted to identify characteristic absorption bands, and functional group assignments were made according to established reference values.

#### 2.4.4 Determination of loading capacity and loading efficiency

A calibration curve for the extract was constructed within the concentration range of 2,500 μg/mL to 9.7 μg/mL. To prepare the stock solution (2,500 μg/mL), 25 mg of extract was dissolved in phosphate buffer (pH 7.4) containing 0.5% Tween 80, and the final volume was adjusted to 10 mL The entrapment efficiency of the extract in the nanoemulsion was quantified using a UV–Vis spectrophotometer (Cary series UV–Vis–NIR, Australia). Following the separation of nanoemulsion phase from the reaction mixture, the absorbance of the supernatant (representing free extract**)** was measured at 550.4 nm. The extract content was calculated using the equation:

$$\text{Entrapment efficiency }\left(\text{EE}\%\right)=\left(\text{Total extract}–\text{Free extract}\right)/\text{Total extract}\times 100$$

All measurements were performed in triplicate (n = 3).

The loading capacity (LC%) was calculated as the ratio of the amount of entrapped extract to the total weight of the nanoemulsion, according to the equation:

$$\text{LC}\%=\left(\text{Amount of entrapped extract }/\text{ Total weight of nanoemulsion}\right)\times 100$$

#### 2.4.5 *In vitro* release study

A total of 5 g of the NE was placed into a dialysis membrane (MWCO 10–12 kDa) and immersed in 45 mL of phosphate-buffered saline (PBS, pH 7.4) maintained at 37 °C. At specific time intervals, the release medium was sampled and analyzed by UV–Vis spectrophotometry at 550.4 nm to determine the concentration of released plant extract. The cumulative release percentage was then calculated.

### 2.5 *In vitro* biological evaluation

#### 2.5.1 Cell lines and culture conditions

The following cell lines were obtained from the American Type Culture Collection (ATCC, United States): human colorectal adenocarcinoma (Caco-2), human hepatocellular carcinoma (HepG2), human mammary gland triple-negative breast adenocarcinoma (MDA-MB-231), human lung carcinoma epithelial-like cells (A549), human neonatal primary dermal fibroblasts (HDFn), and murine macrophages (RAW 264.7). All cell lines, except HDFn, were routinely cultured in Dulbecco’s Modified Eagle Medium (DMEM) supplemented with high glucose. HDFn cells were maintained in Basal Medium Eagle. Complete growth media contained 10% fetal bovine serum (FBS), 2 mM L-glutamine, and 1% antibiotic–antimycotic solution (all from Biowest, Nuaillé, France). Cells were incubated at 37 °C in a humidified atmosphere with 5% CO_2_ and sub-cultured at sub-confluent densities. For passaging, adherent cells (Caco-2, HepG2, MDA-MB-231, A549, and HDFn) were detached using trypsin-EDTA at 37 °C, while RAW 264.7 cells were harvested without trypsinization.

#### 2.5.2 Cytotoxicity assay (MTT)

Cytotoxicity was assessed using the MTT assay as described by (Hansen et al., 1989). Cells (1 × 10^4^ cells/well) were seeded in 96-well plates and treated with various concentrations of the test samples (0–200 μg/mL) for 24 h at 37 °C in a humidified atmosphere containing 5% CO_2_. After treatment, 40 µL of MTT solution was added to each well and incubated for 4 h. The resulting formazan crystals were dissolved in 180 µL of acidified isopropanol, and absorbance was measured at 570 nm using a microplate reader (FLUOstar OPTIMA, BMG LABTECH, Germany). All concentrations were tested in triplicate, and cell viability (%) was calculated compared with untreated control cells. The half-maximal inhibitory concentration (IC_50_) values were determined from the dose–response curves for the cancer cell lines, whereas for RAW 264.7 macrophages only cell viability (%) was evaluated to confirm non-cytotoxic concentrations for subsequent anti-inflammatory assays.

#### 2.5.3 Antimicrobial screening

##### 2.5.3.1 Bacterial strains

Bacterial strains including *Escherichia coli* O157:H7 86-24, *Salmonella Typhimurium* ATTC 14028, *Staph*. *aureus* ATCC 25923, *Pseudomonas aeruginosa* ATCC 9027 were provided by Microbiology Dept. Faculty of Agriculture, Cairo University, *klebsiella pneumonia* was isolated and provided by Dairy Department, National Research Centre, Cairo, Egypt. All bacterial strains were preserved in 25% glycerol at −20 °C.

##### 2.5.3.2 Agar well diffusion method

Screening the antimicrobial activity of *O. sanctum* extract and *O. sanctum* NE against foodborne bacteria performed using the agar diffusion method. Foodborne bacterial strains were activated overnight in tryptone soy broth at 37 °C. Soft Mueller-Hinton agar was prepared by dissolving 0.7% of agar in Mueller-Hinton broth and autoclaving for 15min at 121 °C then cooled down to 45 °C and inoculated with different activated microbial cultures to reach ≈10^7^ cfu/mL then 4 mL were overplayed on solid Mueller-Hinton agar plates. Wells of 6 mm diameter were holed by cork borer after complete solidification of over layer, and then 50 µL extract and NE at concentration of 10 mg/mL was pipetted onto the wells then plates incubated for 24 h at 37 °C. Diameters of inhibition clear zones; without microbial growth were measured using a caliper.

#### 2.5.4 Antioxidant activity: DPPH radical scavenging assay

The antioxidant activity of *O. sanctum* and *O. sanctum* NE was evaluated using the DPPH radical scavenging assay, following the method of (Ratty et al., 1988). Ethanolic DPPH solution (0.1 mM) was prepared, and ascorbic acid (0–50 μg/mL) was used as a standard to generate the calibration curve. In 96-well microplates, 20 µL of the test samples (0–500 μg/mL) were mixed with 180 µL of DPPH solution and incubated at 37 °C for 30 min. Absorbance was then measured at 520 nm using a microplate reader (ELISA reader). All concentrations were tested in triplicate. The half-maximal scavenging concentration (SC_50_) values were determined from dose–response curves.

#### 2.5.5 Integrated network pharmacology and experimental assessment of anti-inflammatory potential

##### 2.5.5.1 Network pharmacology analysis

The bioactive metabolites of *O. sanctum* were retrieved from the PubChem database (https://pubchem.ncbi.nlm.nih.gov/) by obtaining their SMILES notations. The compounds were evaluated using the ADMETlab 3.0 platform to assess pharmacokinetic properties, including oral drug-likeness based on Lipinski’s rule of five (no violations) and quantitative estimate of drug-likeness (QED >0.6). These thresholds were applied to retain molecules with physicochemical characteristics comparable to known bioactive compounds. Metabolites meeting these criteria were selected for further *in silico* analysis. The SwissTargetPrediction database (https://www.swisstargetprediction.ch/) was then used to predict the potential molecular targets of the selected compounds using their canonical SMILES strings.

To identify genes associated with inflammation, relevant targets were collected from DisGeNET database using the keyword “inflammation.” Duplicate targets were removed, and the intersection between *O. sanctum* compound targets and inflammation-related targets was determined using an online Venn diagram tool https://bioinformatics.psb.ugent.be/webtools/Venn/).

The intersecting genes were subjected to protein–protein interaction (PPI) analysis using the STRING v11.5 database (https://string-db.org/). The species was limited to *Homo sapiens*, and a high confidence interaction score (>0.9) was applied. The PPI network was visualized and analyzed in Cytoscape v3.10.0**,** and CytoHubba plugin was used to identify the top hub genes based on degree centrality. Functional enrichment analysis was performed using ShinyGO v0.85**,** covering Gene Ontology (GO) categories—Biological Process (BP), Molecular Function (MF), and Cellular Component (CC)—as well as Kyoto Encyclopedia of Genes and Genomes (KEGG) pathways. Pathways with a *p*-value <0.05 were considered statistically significant.

##### 2.5.5.2 Assessment of cytokine release

RAW 264.7 cells were seeded in 12-well plates at a density of 2 × 10^5^ cells/well and incubated for 24 h. The cells were pretreated with *O. sanctum* extract, *O. sanctum* NE, or dexamethasone (Dex; 1 μM, equivalent to 0.39 μg/mL) for 1 h, followed by stimulation with lipopolysaccharide (LPS; 1 μg/mL) for 18 h to induce an inflammatory response. Dex and LPS were obtained from Sigma-Aldrich (St. Louis, MO, United States). After incubation, culture supernatants were collected, and the levels of inflammatory cytokines TNF-α and IL-6 were quantified using ELISA kits (R&D Systems, Minneapolis, MN, United States) according to the manufacturer’s instructions. All experiments were performed in triplicate.

### 2.6 Statistical analysis

All data were expressed as mean ± standard deviation (SD). The half-maximal inhibitory concentration (IC_50_) values were calculated using non-linear regression analysis (sigmoidal dose–response curve, variable slope). Comparisons of IC_50_ mean values between groups were performed using an unpaired Student’s t-test. For antioxidant activity (DPPH assay) and cytokine levels (TNF-α, IL-6), statistical differences among multiple groups were analyzed using one-way ANOVA followed by Tukey’s multiple comparison *post hoc* test. A p-value of less than 0.05 was considered statistically significant. All statistical analyses were performed using GraphPad Prism software (version 10; GraphPad Software, San Diego, CA, United States). All experiments were performed in three independent biological replicates, and values represent mean ± SD unless otherwise stated.

## 3 Results

### 3.1 Metabolite profiling of the *Ocimum sanctum* extract

#### 3.1.1 GC–MS analysis of volatile and semi-volatile compounds

**FIGURE 1:**
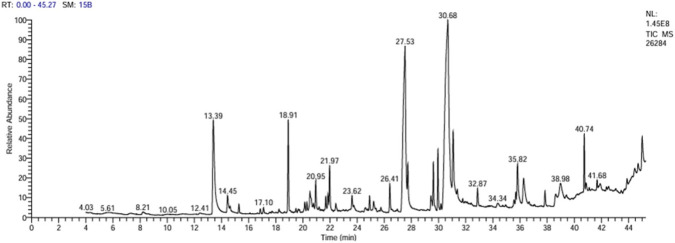
GC–MS total ion chromatogram of the *Ocimum sanctum* extract.

Gas chromatography–mass spectrometry analysis of *O. sanctum* metabolites revealed a chemically diverse profile comprising volatile and semi-volatile phytoconstituents. The total ion chromatogram is shown in Figure 1, while the complete list of identified metabolites is provided in the Supplementary Material S1. Among the detected constituents, several well-known bioactive compounds were identified, including eugenol, caryophyllene, and caryophyllene oxide, together with a series of sesquiterpenes such as α-copaene, spathulenol, and globulol. These identified metabolites were subsequently used for downstream computational assessment. The acquired MS data have been deposited in the MassIVE database under accession number MSV000100925.

#### 3.1.2 LC–QTOF–MS/MS analysis of non-volatile phytochemicals

LC–QTOF–MS/MS analysis of the ethanolic extract of *O. sanctum* revealed a chemically diverse profile comprising numerous non-volatile metabolites across multiple phytochemical classes. Representative compounds included flavonoids (luteolin, apigenin, quercetin, kaempferol, and their glycosides), phenolic acids (rosmarinic acid, caffeic acid derivatives, caffeoyl glucosides), and sterols (β-sitosterol-α-glucoside, α-spinasterol, 7α-hydroxycampesterol, and related phytosterols). In addition to these, a wide range of terpenoids, fatty acid derivatives, and organic acids were also detected. The full metabolite profile with retention times, accurate masses, and MS/MS spectra is provided in the Supplementary Material S2, S2b (positive mode) and (negative mode). The acquired LC–MS/MS data have also been deposited in the MassIVE repository under accession number MSV000100925. All identified compounds from GC-MS and QTOF were retrieved from PubChem in SMILES format and incorporated into the network pharmacology workflow.

### 3.2 Physicochemical properties of the *Ocimum sanctum* NE

#### 3.2.1 Morphological characterization by TEM

**FIGURE 2:**
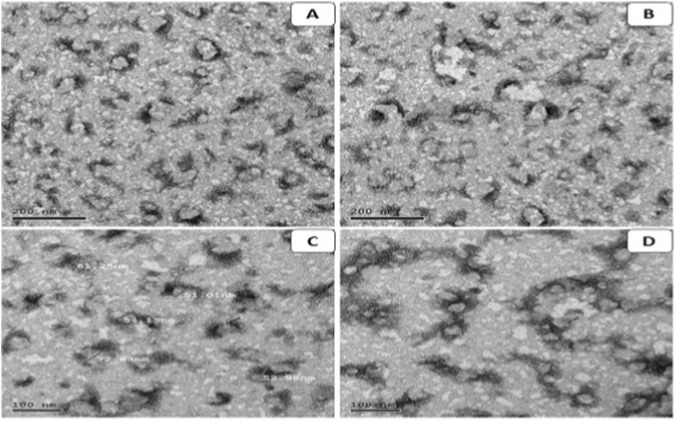
TEM images of the *Ocimum sanctum* NE at different magnifications. **(A)** Spherical droplets at 300,00× with 200 nm scale bar; **(B)** droplet distribution across the copper grid; **(C)** particle size measurements showing diameters of 51–73 nm; **(D)** intact spherical droplets at 500,00× with 100 nm scale bar.

The morphology and size distribution of the *O. sanctum* NE were examined by TEM as shown in Figure 2, the droplets exhibited predominantly spherical to near-spherical morphology with uniform distribution and minimal aggregation. At 300,00× magnification with a 200 nm scale bar (Figures 2A,B), the NE displayed well-dispersed droplets across the copper grid. Higher magnification (∼500,00×; 100 nm scale bar) was used for size analysis (Figure 2C), revealing droplet diameters ranging from 51 to 73 nm. Intact droplet structures and well-defined spherical morphology were further confirmed at higher magnification (Figure 2D).

#### 3.2.2 Particle size and PDI

**FIGURE 3:**
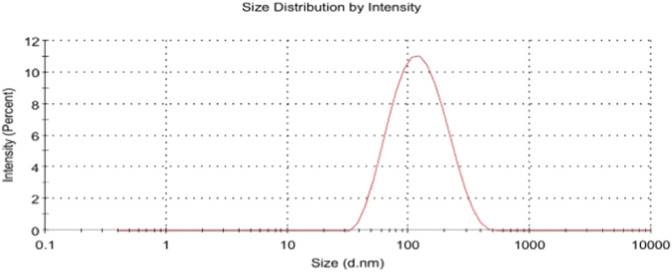
Dynamic light scattering (DLS) profile of the *Ocimum sanctum* NE showing size distribution by intensity.

Dynamic light scattering analysis was performed to determine the overall droplet size distribution and polydispersity of the nanoemulsion, Figure 3, the NE exhibited a mean hydrodynamic diameter of 133.8 ± 22 nm with a PDI of 0.264. The measurement showed a single dominant peak accounting for 100% of the scattering intensity. The intercept value was 0.953, indicating high-quality correlation data. The intensity-based size distribution graph displayed a symmetric peak within the nanoscale range, with no evidence of secondary peaks or large aggregates.

#### 3.2.3 Zeta potential measurements of the NE

**FIGURE 4:**
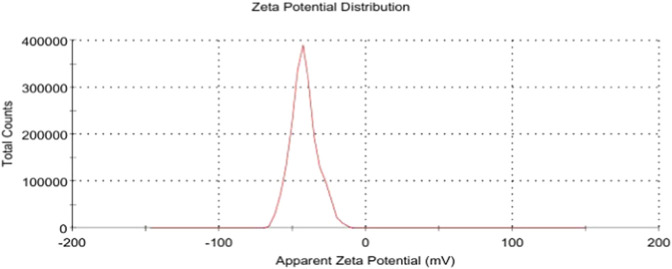
Zeta potential distribution of the *Ocimum sanctum* NE.

Zeta potential measurements were performed to evaluate the surface charge and colloidal stability of the *O. sanctum* NE, as shown in Figure 4. The recorded zeta potential was −42.1 ± 9.25 mV. This value indicates moderate-to-good colloidal stability of the NE containing nanoencapsulated *O. sanctum*. A single peak was observed, accounting for 100% of the area, confirming a homogeneous surface charge distribution. The conductivity of the sample was 0.0687 m/cm, attributed to the presence of charged surfactants within the formulation.

#### 3.2.4 Fourier Transform Infrared analysis of NE components

**FIGURE 5:**
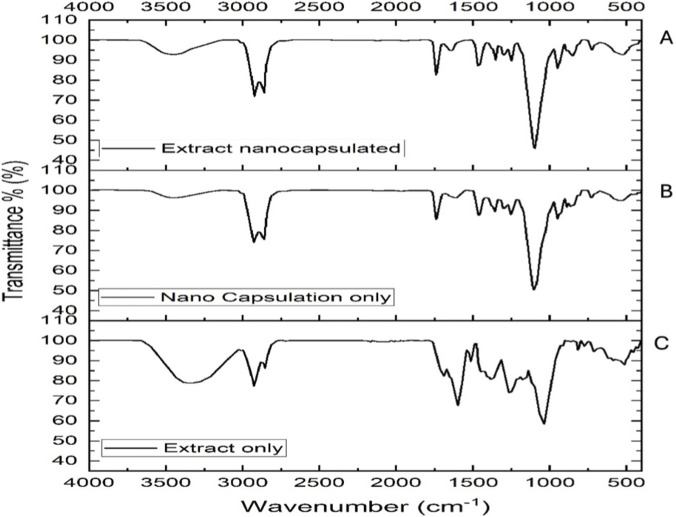
FTIR spectra of **(A)** *Ocimum sanctum* extract nanocapsulated **(B)** blank NE without extract, and **(C)** pure *Ocimum sanctum* extract.

FTIR analysis of *O*. *sanctum* extract revealed characteristic phytochemical groups, including a broad O–H stretch (3,320 cm^-1^), aliphatic C–H stretches (2,930, 2,852 cm^-1^), C=O vibrations (1700–1,600 cm^-1^), C=C stretching (1,500 cm^-1^), and strong C–O peaks (1,250, 1,041 cm^-1^), with aromatic C–H bending evident in the fingerprint region (<1,000 cm^-1^). The empty NE showed typical surfactant bands, including O–H stretching at 3,450 cm^-1^, C–H stretching at 2,920–2,860 cm^-1^, ester C=O at 1730 cm^-1^, and signals for phosphate (1,080 cm^-1^) and sulfate (1,105 cm^-1^) groups. The spectrum of the *O. sanctum* NE largely overlapped with the surfactant matrix, but broadened O–H and minor extract peaks confirmed successful encapsulation of the plant extract within the NE (Figure 5).

#### 3.2.5 UV–vis quantification of *Ocimum sanctum* extract loading

**FIGURE 6:**
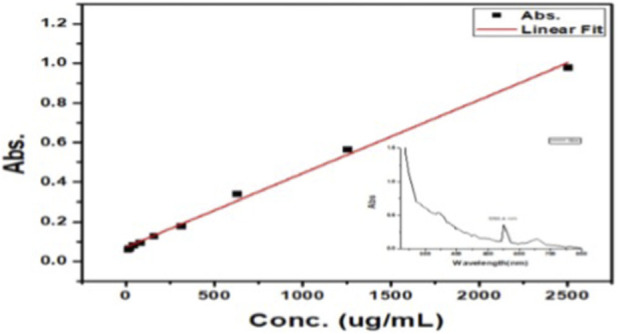
Standard calibration curve of *Ocimum sanctum* extract at 550.4 nm for quantification in the NE formulation.

UV–Vis spectroscopy was employed to quantify the loading of *O*. *sanctum* extract in the mixed oil–based NE. Absorbance was measured at 550.4 nm, showing a shift from the native 515 nm. The calibration curve (Figure 6) exhibited a clear linear relationship between concentration and absorbance over the range of 0–2,500 μg/mL. The encapsulation efficiency was 96.23% ± 3.12% indicating high formulation reproducibility and content uniformity. These results confirm that *O. sanctum* extract was successfully incorporated into the mixed oil–based NE system in a reproducible and consistent manner. Moreover, they validate the reliability of both the formulation procedure and the analytical method employed. The observed bathochromic shift in λmax reflects the effect of nanoemulsion droplets which alter the local polarity and stabilize the excited state of the encapsulated phytochemicals, resulting in a longer-wavelength absorption.

#### 3.2.6 *In vitro* release profile of the *Ocimum sanctum* NE

**FIGURE 7:**
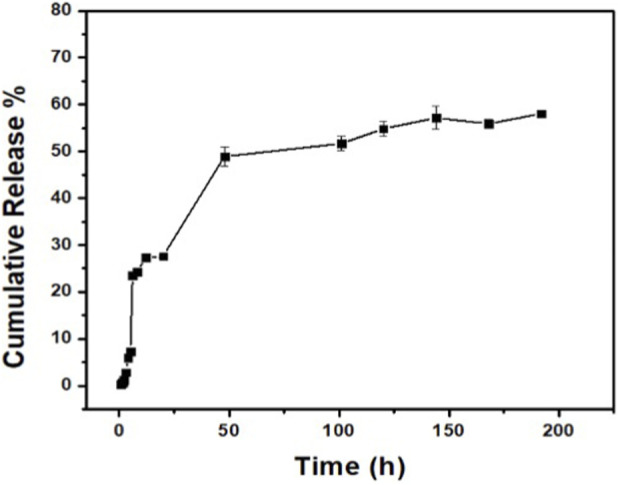
Cumulative *in vitro* release profile of *Ocimum sanctum* extract from the mixed oil–based NE over 8 days (192 h).

The cumulative release of *O. sanctum* extract from the mixed oil–based NE was monitored under *in vitro* conditions for 8 days (192 h). As shown in Figure 7, an initial burst release of approximately 30% occurred within the first 24 h, followed by a gradual and sustained release. By 48 h, the cumulative release reached about 50%, increasing to nearly 55% at 120 h, and approaching 60% at the end of the study.

The release behavior exhibited a biphasic pattern—an initial burst phase followed by a sustained, diffusion-controlled phase—attributable to the encapsulation efficiency and carrier characteristics of the mixed oil phase. This controlled and time-dependent release profile describes immediate availability followed by retention of the extract within the carrier system and characterizes formulation behavior under the experimental conditions. The later release phase reflects retention within the nanodroplets and does not represent the duration of biological activity under the *in-vitro* assay timeframe.

### 3.3 Biological activity and network pharmacology

#### 3.3.1 Anticancer activity (MTT assay): cell viability and IC_50_

**FIGURE 8:**
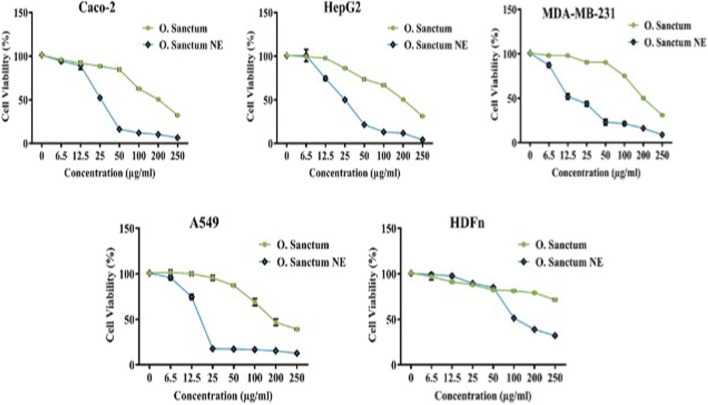
Dose-dependent cytotoxicity of *Ocimum sanctum* and its NE in different cell lines. Cell viability (%) was determined by MTT assay after 24 h treatment with increasing concentrations (0–250 μg/mL) of each formulation. Values represent mean ± SD from three independent biological experiments. *Ocimum sanctum* NE exhibited significantly greater cytotoxicity in Caco-2, HepG2, MDA-MB-231, and A549 cells, while minimal toxicity was observed in normal HDFn cells.

**FIGURE 9:**
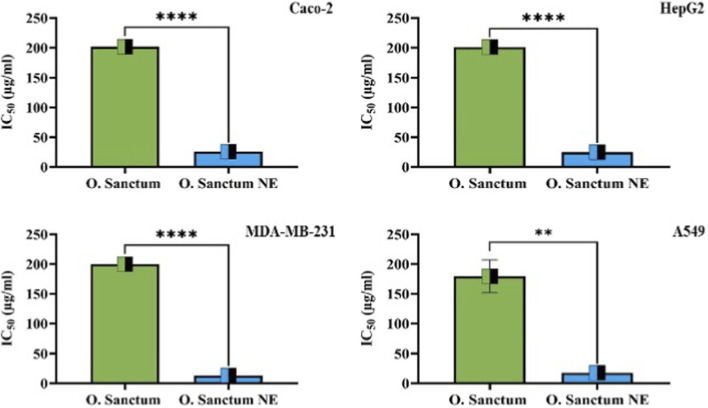
Comparison of IC_50_ values for *Ocimum sanctum* and *Ocimum sanctum* NE in cancer cell lines. The bar graphs represent mean ± SD from three independent biological experiments. Statistical analysis was performed using unpaired Student’s t-test: p < 0.01 (**), p < 0.0001 (****). The NE formulation exhibited significantly lower IC_50_ values in all cancer cell lines, confirming superior anticancer potency.

The cytotoxic activity of *O. sanctum* extract and its NE was evaluated in Caco-2, HepG2, MDA-MB-231, A549, and normal HDFn cells after 24 h exposure. As illustrated in Figures 8, 9, both formulations reduced cell viability in a concentration-dependent manner, with the NE producing markedly stronger effects across all cancer cell lines.

The NE consistently required much lower concentrations to inhibit cell growth compared with the crude extract (Table 1). Among the tested cells, MDA-MB-231 and A549 were the most sensitive to the nanoformulation, whereas the crude extract exhibited comparatively weak cytotoxicity within the studied range.

**TABLE 1:** IC_50_ values of *Ocimum sanctum*, O. sanctum NE and Blank NE after 24 h MTT assay. Values represent mean ± SD from three independent biological experiments.

Sample	Caco-2 (µg/mL)	HepG2 (µg/mL)	MDA-MB-231 (µg/mL)	A549 (µg/mL)	HDFn (µg/mL)
O. sanctum extract	201.7 ± 2.98	201.0 ± 1.47	199.7 ± 2.44	179.4 ± 27.56	>250
O. sanctum NE	25.78 ± 0.24	24.89 ± 0.93	13.03 ± 0.79	17.53 ± 0.73	102.3 ± 0.29
Blank NE (vehicle)	>250	>250	>250	>250	>250

Normal HDFn fibroblasts showed limited susceptibility, indicating a degree of selectivity toward malignant cells. In contrast, the blank NE did not decrease cell viability below 50% at any tested concentration, and therefore its IC_50_ could not be determined (>250 μg/mL).

Statistical analysis demonstrated significant differences between the extract and NE treatments (p < 0.01–0.0001).

#### 3.3.2 Antibacterial screening (agar well diffusion)

**FIGURE 10:**
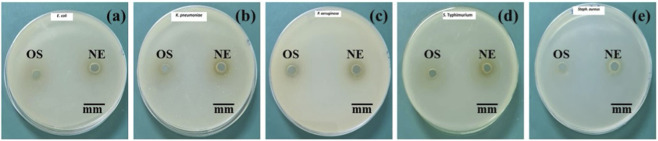
Antibacterial activity of *Ocimum sanctum* extract (OS) and its nanoemulsion (NE) against **(a)** *Escherichia coli*, **(b)** *Klebsiella pneumoniae*, **(c)** *Pseudomonas aeruginosa*, **(d)** *Salmonella Typhimurium*, and **(e)** *Staphylococcus aureus* using the agar well diffusion method. The nanoemulsion (NE) exhibited distinct zones of inhibition, while the crude extract (OS) showed no detectable antibacterial effect. Inhibition zones were measured in mm (mean ± SD, n = 3 independent experiments).

The antibacterial activity of *O. sanctum* extract and its NE was evaluated using the agar well diffusion method against *E. coli*, *Klebsiella pneumoniae*, *Pseudomonas aeruginosa*, *Salmonella Typhimurium*, and *Staphylococcus aureus*. A volume of 50 µL of each sample at a concentration of 10 mg/mL was added to the wells, and the plates were incubated at 37 °C for 24 h. The inhibition zones are shown in Figure 10 and summarized in Table 2. No inhibition zones were observed for the *O. sanctum* extract against any of the tested bacterial strains at the applied concentration. In contrast, the NE produced clear inhibition zones ranging from 8.00 ± 0.35 mm to 14.80 ± 0.26 mm. The largest zone of inhibition was recorded against *Salmonella Typhimurium* (14.80 ± 0.26 mm), followed by *E. coli* (10.60 ± 0.55 mm), *Klebsiella pneumoniae* (10.53 ± 0.50 mm), and *P. aeruginosa* (10.16 ± 0.15 mm). The smallest inhibition zone was observed against *S. aureus* (8.00 ± 0.35 mm).

**TABLE 2:** Zone of inhibition (mm) of *Ocimum sanctum* extract (OS) and its NE against selected pathogenic bacteria. Values represent mean ± SD from three independent biological experiments.

Bacterial strains	Inhibition zones (mm)	
	*Ocimum sanctum* extract	*Ocimum sanctum* NE
*Escherichia coli*	0	10.60 ± 0.55
*Salmonella Typhimurium*	0	14.80 ± 0.26
*Pseudomonas aeruginosa*	0	10.16 ± 0.15
*Klebsiella pneumonia*	0	10.53 ± 0.50
*Staphylococcus aureus*	0	8.00 ± 0.35

#### 3.3.3 Antioxidant activity (DPPH assay)

**FIGURE 11:**
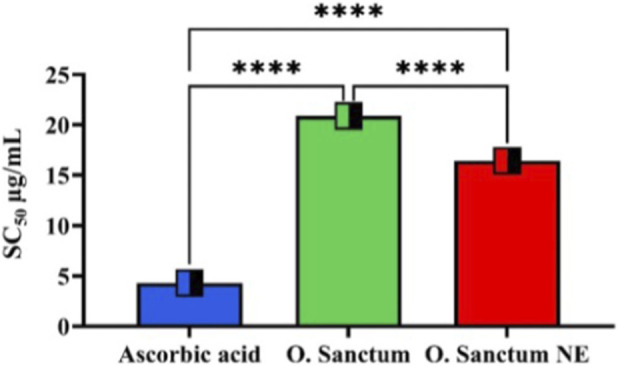
Comparative analysis of the SC_50_ values for DPPH radical scavenging activity of ascorbic acid, *Ocimum sanctum* extract, and *O. sanctum* NE. Ascorbic acid exhibited the lowest SC_50_ value, indicating the strongest antioxidant activity, followed by *O. sanctum* NE and the crude *O. sanctum* extract. Values represent mean ± SD from three independent biological experiments. Statistical analysis showed highly significant differences among all groups (p < 0.0001).

The DPPH radical scavenging assay showed variation in SC_50_ values among the tested samples. As shown in Figure 11 **,** ascorbic acid (standard) exhibited an SC_50_ value of 4.31 ± 0.37 μg/mL**,** the *O. sanctum* NE showed 16.42 ± 0.30 μg/mL**,** and the crude *O. sanctum* extract recorded 20.83 ± 0.11 μg/mL. Statistical analysis revealed a significant difference between the NE and the crude extract **(**p < 0.0001**),** as well as among all tested groups.

#### 3.3.4 Network pharmacology analysis and anti-inflammatory activity

##### 3.3.4.1 Network pharmacology analysis of *Ocimum sanctum* metabolites

**FIGURE 12:**
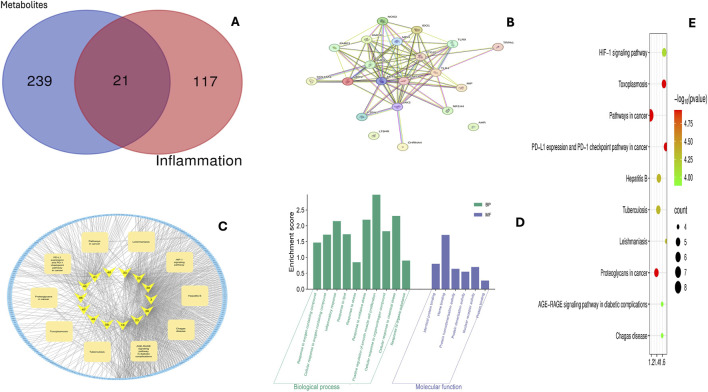
**(A)** Venn diagram showing the overlapping targets between *Ocimum sanctum* metabolites and inflammation-related genes. **(B)** Protein–protein interaction (PPI) network of overlapping anti-inflammatory targets constructed using STRING. **(C)** Merged compound–target–pathway network illustrating interactions between *Ocimum sanctum* metabolites and inflammation-associated targets and pathways. Yellow nodes are *Ocimum sanctum* compounds labelled by their ADMET IDs (Supplementary Table S3); blue nodes are targets; beige rectangles are enriched KEGG pathways. **(D)** Gene Ontology enrichment analysis for biological processes and molecular functions. **(E)** KEGG pathway enrichment of targets associated with *Ocimum sanctum* metabolites. The top enriched pathways include HIF-1 signalling, PD-1/PD-L1 checkpoint, AGE–RAGE signalling in diabetic complications, infection-related pathways (hepatitis B, tuberculosis, leishmaniasis, toxoplasmosis, Chagas disease), and cancer-associated modules (proteoglycans in cancer, pathways in cancer). Bubble size indicates gene count; colour indicates −log10(p).

The network pharmacology analysis identified several overlapping targets shared between *O. sanctum* metabolites and inflammation-related genes (Figure 12A). These common targets represent potential molecular mediators of the anti-inflammatory activity of the plant.

The PPI network constructed for these targets revealed a densely interconnected structure, indicating strong functional relationships among them (Figure 12B). Analysis of the network topology using the CytoHubba plugin identified AKT1, STAT3, PTGS2, MMP9, MMP2, TLR4, JAK2, MPO, and HMOX1 as key hub genes with the highest degree of connectivity (Table 3 **)**.

**TABLE 3:** Top 10 hub genes identified by CytoHubba in Cytoscape based on degree centrality.

Top 10 in network String_Interactions_Short.txt ranked by degree method		
Rank	Name	Degree
1	AKT1	16.0
2	PTGS2	14.0
3	STAT3	14.0
4	MMP9	14.0
5	TLR4	14.0
6	JAK2	13.0
7	HMOX1	11.0
8	MMP2	10.0
9	MPO	10.0
10	IDO1	9.0

The compound–target–pathway merged network showed that several *O. sanctum* metabolites are connected to multiple inflammation-related targets and pathways (Figure 12C). In the network, compound nodes are labelled with their numerical identifiers that correspond to the ADMET profile in the Supplementary Table S3.

The GO enrichment analysis indicated that the overlapping targets were predominantly associated with inflammatory response, cytokine-mediated signalling, oxidative stress regulation, and apoptosis-related processes. The enriched molecular functions included enzyme binding, cytokine receptor activity, and oxidoreductase activity (Figure 12D).

KEGG pathway enrichment identified immune/inflammation–associated signalling pathways—HIF-1 signalling, PD-1/PD-L1 checkpoint, and AGE–RAGE signalling in diabetic complications—along with infection-related pathways (hepatitis B, tuberculosis, leishmaniasis, toxoplasmosis, Chagas disease) and cancer-associated modules (proteoglycans in cancer, pathways in cancer) (Figure 12E).

##### 3.3.4.2 Effect of *Ocimum sanctum* and its NE on RAW 264.7 cell viability (MTT assay)

**FIGURE 13:**
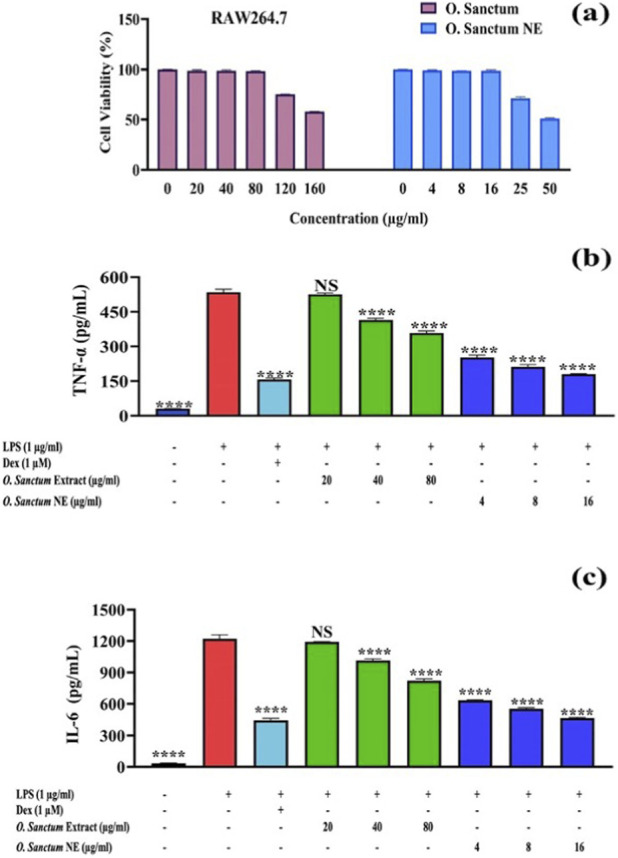
Effects of *Ocimum sanctum* extract and its NE on LPS-induced cytokine production in RAW 264.7 macrophages. **(a)** Effect of *O. sanctum* extract and its NE on the viability of RAW 264.7 macrophage cells. Cells were treated with increasing concentrations of *O. sanctum* (0–160 μg/mL) or *O. sanctum* NE (0–50 μg/mL) for 24 h, and cell viability was evaluated using the MTT assay. Values represent mean ± SD from three independent biological experiments. The treatments significantly reduced TNF-α **(b)** and IL-6 **(c)** levels compared with the LPS-stimulated control (p < 0.0001), with the NE showing a stronger inhibitory effect than the crude extract. Dex was used as a positive control. ****p < 0.0001 vs. LPS-stimulated control; NS, not significant.

The network pharmacology analysis predicted several *O. sanctum* phytoconstituents to possess anti-inflammatory potential in relation to signaling pathways involved in macrophage activation. To examine the biological response suggested by these predictions, the cytotoxicity of *O. sanctum* and its NE was evaluated in RAW 264.7 macrophages using the MTT assay to determine safe concentrations for treatment. As shown in Figure 13, the crude extract maintained >95% cell viability up to 80 μg/mL, while viability decreased to approximately 55% at 160 μg/mL. In contrast, the NE formulation exhibited a concentration-dependent decline in viability, remaining non-toxic up to 16 μg/mL but reducing viability to around 50% at 50 μg/mL. Based on these results, concentrations maintaining ≥90% cell viability were selected for subsequent cytokine assays to ensure non-cytotoxic evaluation of anti-inflammatory effects.

##### 3.3.4.3 Modulation of cytokine release in LPS-Stimulated RAW 264.7 macrophages

Following LPS stimulation, RAW 264.7 macrophages showed a marked increase in TNF-α and IL-6 secretion, indicating an inflammatory response (Figures 13b,c). Pretreatment with Dex, *O. sanctum* extract, or its NE significantly and dose-dependently reduced cytokine levels compared with the LPS-stimulated control (p < 0.0001), with the NE showing a more pronounced inhibitory effect.

## 4 Discussion

### 4.1 Interpretation of chemical profiling data

#### 4.1.1 GC–MS-based phytochemical insights

The volatile profile of *O. sanctum*, dominated by eugenol, caryophyllene, and caryophyllene oxide, along with sesquiterpenes such as α-copaene, spathulenol, and globulol, is consistent with chemotypes previously described for *Ocimum* spp. (Dharsono et al., 2022; Verma et al., 2013). Eugenol is well known for its antioxidant, anti-inflammatory, and adjunct anticancer properties via NF-κB modulation (Damasceno et al., 2024), while caryophyllene oxide displays antiproliferative and pro-apoptotic activity (Delgado et al., 2021; Xiu et al., 2022). These sesquiterpenes are likely to contribute synergistically to the overall biological effects. However, the therapeutic application of such volatiles is often limited by instability and poor bioavailability. In our work, the NE formulation was designed to enhance the stability, retention, and delivery of these volatile constituents, thereby strengthening their pharmacological potential (Kelidari et al., 2025).

#### 4.1.2 LC–QTOF–MS/MS-derived metabolite characterization

LC–QTOF–MS/MS analysis confirmed the presence of flavonoids (luteolin, apigenin, quercetin derivatives, kaempferol), phenolic acids (rosmarinic and caffeic acid derivatives), and sterols (β-sitosterol, α-spinasterol) in *O. sanctum*. These metabolites are widely reported to exert antioxidant, anti-inflammatory, and anticancer effects through modulation of key signaling pathways and suppression of oxidative stress (Lv et al., 2025; Allemailem et al., 2024; Noor et al., 2022). Despite their therapeutic promise, many of these phytochemicals have poor aqueous solubility and stability, which can restrict their clinical applicability. Nanoemulsions provide an effective delivery strategy by enhancing solubility, protecting labile compounds, and improving absorption and tissue distribution, thereby amplifying both anti-inflammatory and anticancer activities of *O. sanctum* phytoconstituents (Chatzidaki and Mitsou, 2025). Such integration of phytochemical richness with advanced formulation technology highlights the potential of *O. sanctum* NE as promising candidates for future therapeutic applications.

### 4.2 Implications of NE characterization

#### 4.2.1 Morphological implications from TEM

Transmission electron microscopy (Figures 2A–D) revealed predominantly spherical to near-spherical droplets with uniform distribution and no obvious aggregation, consistent with features of kinetically stable nanoemulsions. The annotated measurements in Figure 2C (≈51–73 nm) fall within the nanometric size range typically reported for stable NE systems (Preeti et al., 2023; Ahmad I. et al., 2024). Such small, spherical droplets provide an efficient platform for encapsulating *O. sanctum* phytochemicals, ensuring homogeneous dispersion in aqueous media. This morphology is expected to enhance the apparent solubility and bioavailability of lipophilic constituents compared with the crude extract (Preeti et al., 2023; Ahmad I. et al., 2024).

#### 4.2.2 Particle size and distribution (DLS)

Dynamic light scattering analysis revealed a Z-average hydrodynamic diameter of ∼100 nm with a PDI of 0.264, Figure 3, indicating a narrow size distribution and high sample homogeneity. PDI values below 0.3 are generally regarded as acceptable for well-controlled formulations, supporting the physical stability of the NE during storage (Danaei et al., 2018). The unimodal size distribution is consistent with TEM observations, while the slightly larger DLS-derived size is expected since DLS reports intensity-weighted hydrodynamic diameters, whereas TEM provides number-based geometric sizes (Filippov et al., 2023). Taken together, these findings confirm effective droplet size reduction and the formation of a stable colloidal dispersion.

#### 4.2.3 Zeta potential and stability implications

The NE exhibited a zeta potential of −42.1 mV, Figure 4, well above the conventional stability threshold of ±30 mV. Such a high interfacial charge indicates strong electrostatic repulsion among droplets, minimizing the risk of aggregation or coalescence. This electrostatic stabilization helps preserve *O. sanctum* phytochemicals—particularly volatile or oxidation-prone constituents—by maintaining them within intact nanodroplets. These findings are consistent with established zeta-potential guidelines for colloidal stability and with recent reviews emphasizing the role of surface charge in governing nanoparticle behavior in drug delivery systems (Öztürk et al., 2024).

#### 4.2.4 Interpretation of FT-IR spectral findings

The Fourier Transformed Infrared spectra, Figure 5, confirmed the successful encapsulation of *O. sanctum* extract into the NE system. The extract-only spectrum displayed characteristic phytochemical peaks, including broad O–H stretching (∼3,300–3,400 cm^-1^), aliphatic C–H stretches (∼2,930 and 2,850 cm^-1^), C=O stretching near 1700 cm^-1^, aromatic C=C vibrations (1,500–1,600 cm^-1^), and C–O stretches (1,250–1,040 cm^-1^), consistent with phenolics, flavonoids, and terpenoids (Ahmad N. et al., 2024). The blank NE spectrum was dominated by surfactant signals, including broadened O–H (∼3,450 cm^-1^), strong C–H (2,920–2,860 cm^-1^), ester C=O (∼1730 cm^-1^), PO_2_^−^ (∼1,080 cm^-1^), and sulfate vibrations (∼1,100 cm^-1^), in line with previous formulations (Singh et al., 2019). The extract-loaded NE spectrum largely resembled the blank, with extract-specific peaks masked by surfactant bands, indicating entrapment within the surfactant/lipid matrix through noncovalent forces rather than covalent bonding (Ahmad N. et al., 2024; Singh et al., 2019). Together with TEM, DLS, and zeta potential results, these findings confirm the stable encapsulation of *O. sanctum* phytochemicals in the NE system.

#### 4.2.5 Entrapment efficiency and its implications

The UV–Vis spectroscopy analysis revealed an entrapment efficiency of 96.23% ± 3.12%, confirming that the majority of phytochemicals were successfully incorporated into the NE system. This high efficiency suggests that the oil phase provided an effective solubilization medium, while the surfactant layer stabilized the droplets and minimized drug leakage. Comparable findings were reported by (Boonrueang et al., 2025), who achieved high loading efficiencies for curcumin and capsaicin in a triple-loaded NE system, accompanied by improved photostability and protective effects. In line with their results, the present study indicates that efficient entrapment not only enhances the chemical stability of plant-derived bioactives but also increases the likelihood of improved bioavailability compared to the unformulated extract (Boonrueang et al., 2025).

#### 4.2.6 *In vitro* release profile

In our study, the NE exhibited a distinct biphasic release pattern, Figure 7, An initial burst release of nearly 30% was observed within the first 24 h, which we attribute to the presence of loosely surface-associated phytochemicals. This was followed by a sustained release phase, with cumulative release reaching ∼60% over 8 days as the phytochemicals gradually diffused from the droplet core. Such biphasic behavior is consistent with reports from other NE systems (Suresh et al., 2022). The dual-phase release observed in our formulation indicates immediate availability followed by retention of phytochemicals within the carrier system and describes the physicochemical release behavior under *in-vitro* conditions rather than the duration of biological activity.

### 4.3 Multifunctional biological activities of the *Ocimum sanctum* nanoemulsion

#### 4.3.1 Nanoemulsion-mediated enhancement of anticancer potency and selectivity

The nanoemulsion formulation of *O*. *sanctum* markedly enhanced the cytotoxic potency of the extract across all tested cancer cell lines while exhibiting comparatively lower toxicity toward normal fibroblasts, Figures 8, 9. These findings are presented as a comparative, formulation-focused evaluation**,** as no standard chemotherapeutic reference drug was included in this study. The observed differences therefore reflect the impact of nanoemulsification on extract performance rather than a direct assessment of anticancer potency relative to established drugs. The enhanced cytotoxic response may be attributed to formulation-related factors such as improved solubility, increased cellular contact, and facilitated uptake of bioactive phytoconstituents associated with nanoscale droplets, as reported for other phytochemical nanoemulsion systems (Preeti et al., 2023; Chatzidaki and Mitsou, 2025). The enhanced cellular response observed for *O. sanctum* NE may also be attributed to improved stability and controlled release of its bioactive compounds, which are often prone to oxidation or degradation in crude extracts. The absence of cytotoxicity observed for the blank nanoemulsion indicates that the biological activity is related to the incorporated phytoconstituents rather than the formulation excipients. Nanoemulsion encapsulation protects such sensitive phytochemicals from environmental stress, thereby preserving their chemical stability and enhancing bioavailability and cellular uptake (Chatzidaki and Mitsou, 2025; Liu et al., 2019). Consistent with these observations, plant-derived nanomaterials are increasingly recognized as valuable tools in medicinal chemistry, offering innovative strategies to combat complex diseases such as cancer (Song et al., 2025). Phytochemicals are known to influence multiple cellular pathways, including proliferation, apoptosis, and epigenetic regulation, thereby contributing to diverse biological responses relevant to cancer biology (Pan et al., 2025). Overall, the present results suggest that nanoemulsification improves the cellular activity profile of *O. sanctum* extract under *in-vitro* conditions.

#### 4.3.2 Formulation-enhanced antibacterial activity of *Ocimum sanctum* nanoemulsion

The agar well diffusion assay performed in this study represents a preliminary screening method for antibacterial activity, and the findings reflect the qualitative nature of this approach. Although *O. sanctum* is well known for its intrinsic antibacterial properties, the crude extract in this study produced no measurable inhibition when tested at a low concentration and incubated for 24 h at 37 °C. In contrast, the NE formulation of *O. sanctum* exhibited distinct antibacterial activity under the same conditions, Figure 10 and Table 2, indicating a formulation-dependent difference in observable antibacterial response even at very low concentrations. Nanoemulsions are known to improve the solubility, diffusion, and membrane interaction of hydrophobic phytoconstituents, which may influence interaction with bacterial cells (Teixeira et al., 2007). These observations are consistent with earlier reports describing broad-spectrum antibacterial activity among *Ocimum* species, primarily linked to essential oil constituents active against both Gram-positive and Gram-negative bacteria (Dharsono et al., 2022; Rokonuzzaman et al., 2024). Collectively, these findings suggest that while *O. sanctum* itself possesses antibacterial potential, the nanoemulsion-based delivery system modifies the observable antibacterial behavior under the tested conditions through improved solubility and dispersion.

#### 4.3.3 Antioxidant capacity (DPPH)

The enhanced DPPH radical–scavenging activity observed for the *O. sanctum* NE compared with the crude extract in this study, Figure 11, can be attributed to the superior physicochemical characteristics of NE -based delivery systems. In our findings, the NE demonstrated stronger free radical–scavenging efficiency than the corresponding extract, indicating that the encapsulation process effectively improved the antioxidant performance of *O. sanctum* constituents. Such nanoformulations are known to encapsulate and protect phenolic and flavonoid antioxidants, thereby minimizing oxidative degradation and preserving their reactivity during storage and testing (Tadros et al., 2004). The nanoscale droplet size increases surface area and reduces interfacial tension, enhancing molecular diffusion and facilitating closer contact between active compounds and DPPH radicals (Baspinar and Borchert, 2012). Moreover, nanoemulsions improve permeability across biological membranes and prolong retention, which may translate into enhanced bioavailability and systemic antioxidant potential (Chatzidaki and Mitsou, 2025). Collectively, these formulation-related advantages provide a rational explanation for the stronger scavenging capacity observed in our *O. sanctum* NE. In this regard, the formulation of nutraceutical products has emerged as an important strategy to improve the bioavailability of antioxidant compounds. Various nanotechnology-based formulation approaches, including nanoemulsions, have been developed in the past decade to enhance stability, solubility, and physiological effectiveness of plant-derived antioxidants (Nisar and Wan, 2025).

#### 4.3.4 Network-based regulation of inflammatory cytokines

Network pharmacology analysis of inflammation-related genes identified several hub regulators that control cytokine expression, Table 3; Figure 12C. Based on degree centrality within the PPI network, Figure 12b, AKT1**,** PTGS2**,** STAT3**,** and TLR4 emerged as the most interconnected nodes, forming a highly interconnected network that integrates signal transduction, transcriptional control, and inflammatory mediator synthesis. AKT1**,** ranked as the top hub, functions as a central kinase within the PI3K/AKT pathway, linking upstream receptor activation to intracellular signaling cascades. It modulates NF-κB and mTOR activity, thereby regulating macrophage polarization and the expression of pro-inflammatory genes (Androulidaki et al., 2009). The high connectivity of AKT1 indicates its involvement bridging multiple inflammatory mediators. PTGS2 (COX-2), the second-ranked node, catalyzes the conversion of arachidonic acid into prostaglandins, which amplify cytokine release and sustain inflammatory tone. The COX-2–PGE_2_ cascade plays a dual role, contributing to both the propagation and resolution of inflammation depending on the cellular context (Turini and DuBois, 2002; Martín-Vázquez et al., 2023). STAT3 serves as a dynamic transcriptional regulator that fine-tunes inflammatory signaling downstream of TLR4**.** In LPS-stimulated macrophages**,** STAT3 inhibition enhances early NF-κB and PI3K/Akt activation, promoting iNOS and COX-2 expression. Conversely, during the late phase of stimulation, STAT3 activation suppresses NF-κB signaling and nitric oxide production, thus maintaining a balance between pro- and anti-inflammatory responses (Ahuja et al., 2020). TLR4, another major hub, acts as the upstream pattern-recognition receptor that detects LPS and initiates inflammatory signaling through MyD88-and TRIF-dependent pathways, leading to the activation of NF-κB and MAPKs and the transcription of pro-inflammatory cytokines (Lu et al., 2008).

This network suggests that inflammation involves through multiple interconnected pathways rather than a single signaling event. The network analysis further suggests that the bioactive metabolites of *O*. *sanctum* may interact with several key regulatory targets—particularly AKT1**,** STAT3, and PTGS2—which play central roles in cytokine production and inflammatory signaling. Therefore, RAW264.7 macrophages were used to evaluate the anti-inflammatory potential of *O. sanctum* extracts, both crude and nano-formulated, by measuring TNF-α and IL-6 levels using ELISA. This experimental approach provides functional assessment in relation to the prediction and determines whether nano-formulation enhances the ability of *O. sanctum* metabolites to modulate cytokine production compared with the crude extract.

#### 4.3.5 Enhanced anti-inflammatory activity of *Ocimum sanctum* nanoemulsion compared to the crude extract in RAW 264.7 cells

For cytokine measurements, RAW 264.7 macrophages were treated with certain concentrations of O. sanctum extract (20, 40, and 80 μg/mL) and its nanoemulsion (4, 8, and 16 μg/mL), selected based on the MTT viability data showing >90% cell viability within this range (Figure 13a), non-toxic concentrations of O. sanctum extract and its NE were selected for cytokine assays to ensure that the observed effects were not influenced by cytotoxicity. LPS stimulation significantly elevated TNF-α and IL-6 levels, confirming inflammation induction while pretreatment with the extract or its NE significantly reduced cytokine levels (p < 0.0001), Figures 13b,c. The NE produced a stronger inhibitory effect, suggesting enhanced cellular uptake and bioavailability of active metabolites. These reductions in TNF-α and IL-6 are consistent with the pathways highlighted of AKT1, STAT3, and PTGS2, targets implicated of cytokine transcription identified in the network analysis, providing a biological context for the observed anti-inflammatory activity. Consistent with these findings, O. tenuiflorum (syn. *Ocimum sanctum*) extract has been shown to suppress macrophage-mediated inflammation by downregulating TNF-α, IL-6, IL-1β, iNOS, and COX-2 expression in LPS-stimulated RAW 264.7 cells (Srichok et al., 2022). Similarly, O. sanctum leaf extracts have been reported to attenuate inflammatory activation in human monocytic (THP-1) cells through inhibition of NF-κB and COX-2 (Choudhury et al., 2014). Moreover, nanoemulsion-based delivery systems have demonstrated superior anti-inflammatory efficacy by enhancing solubility, stability, and targeted delivery of bioactive compounds to macrophages (Vichare et al., 2025). Overall, O. sanctum NE exhibited stronger anti-inflammatory potential than the crude extract, highlighting its improved formulation-dependent anti-inflammatory activity under the tested conditions**.**

## 5 Conclusion

This study developed a stable *O. sanctum* nanoemulsion with nanoscale uniformity, high entrapment efficiency, and a sustained release profile. Compared with the crude extract, the nanoemulsion exhibited enhanced *in vitro* cytotoxic, antibacterial, antioxidant, and anti-inflammatory activities. Network pharmacology analysis predicted AKT1, STAT3, PTGS2, and TLR4 as potential inflammation-related targets, while experimental validation was limited to cytokine suppression (TNF-α and IL-6) in LPS-stimulated RAW 264.7 macrophages. Overall, these findings support the nanoemulsion as a promising plant-derived formulation for further investigation. Future studies will focus on detailed mechanistic validation and *in vivo* evaluation to further substantiate safety, efficacy, and translational potential.

## Data Availability

The datasets presented in this study can be found in online repositories. The names of the repository/repositories and accession number(s) can be found in the article/Supplementary Material.

## Glossary

ADMET: Absorption, Distribution, Metabolism, Excretion, and Toxicity
AKT1 (gene): Protein kinase B alpha
ATCC: American Type Culture Collection
ATR-FTIR: Attenuated Total Reflectance–Fourier Transform Infrared (spectroscopy)
COX-2: Cyclooxygenase-2 (see PTGS2)
Dex: Dexamethasone
DMEM: Dulbecco’s Modified Eagle Medium
EI: Electron Ionization
FBS: Fetal Bovine Serum
GC–MS: Gas Chromatography–Mass Spectrometry
HDFn: Human Dermal Fibroblasts neonatal
HMOX1 (gene): Heme Oxygenase-1
HRTEM: High-Resolution Transmission Electron Microscopy
IL-1β: Interleukin-1 beta
JAK2 (gene): Janus Kinase-2
LC–QTOF–MS/MS: Liquid Chromatography–Quadrupole Time-of-Flight Tandem Mass Spectrometry
MAPK(s): Mitogen-Activated Protein Kinase(s)
MPO (gene): Myeloperoxidase
MSe: Data-independent acquisition mode (Waters)
MWCO: Molecular Weight Cut-Off
NF-κB: Nuclear Factor kappa-light-chain-enhancer of activated B cells
NO: Nitric Oxide
PDI: Polydispersity Index
**PGE**_**2**_: Prostaglandin E_2_
PPI: Protein–Protein Interaction
QED: Quantitative Estimate of Drug-likeness
RAW 264.7: Murine macrophage cell line (designation)
SD: Standard Deviation
SPME: Solid-Phase Microextraction
STRING: Search Tool for the Retrieval of Interacting Genes/Proteins
TLR4 (gene): Toll-Like Receptor-4
TOF: Time-of-Flight
UV–Vis: Ultraviolet–Visible (spectroscopy)
AGE–RAGE: Advanced Glycation End-products–Receptor for Advanced Glycation End-products
ANOVA: Analysis of Variance
ATR: Attenuated Total Reflectance
CC (GO): Cellular Component (Gene Ontology)
**DDH**_**2**_ **O**: Double-distilled water
DLS: Dynamic Light Scattering
DPPH: 2,2-Diphenyl-1-picrylhydrazyl
ELISA: Enzyme-Linked Immunosorbent Assay
FTIR: Fourier Transform Infrared (spectroscopy)
GO: Gene Ontology
HIF-1: Hypoxia-Inducible Factor-1
HP-5MS: (GC column) 5% phenyl methyl siloxane capillary column
**IC**_**50**_: Half-maximal inhibitory concentration
IL-6: Interleukin-6
KEGG: Kyoto Encyclopedia of Genes and Genomes
LPS: Lipopolysaccharide
MF (GO): Molecular Function (Gene Ontology)
MS/MS: Tandem Mass Spectrometry
MTT: 3-(4,5-Dimethylthiazol-2-yl)-2,5-Diphenyltetrazolium Bromide
NE: Nanoemulsion
NIST: National Institute of Standards and Technology
PBS: Phosphate-Buffered Saline
PD-1/PD-L1: Programmed cell death protein-1 / Programmed death-ligand-1
PI3K: Phosphoinositide 3-kinase
PTGS2 (gene): Prostaglandin-Endoperoxide Synthase-2 (COX-2)
QTOF: Quadrupole Time-of-Flight
**SC**_**50**_: Half-maximal scavenging concentration
SMILES: Simplified Molecular-Input Line-Entry System
STAT3 (gene): Signal Transducer and Activator of Transcription-3
TEM: Transmission Electron Microscopy
TNF-α: Tumor Necrosis Factor-alpha
USP: United States Pharmacopeia
