# Supplementary material for: Integrated metabolomic, nanoformulation, and network pharmacology approach reveals multifunctional bioactivities of an Ocimum sanctum nanoemulsion
Source: Front Bioeng Biotechnol. 2026 Mar 20;14:1731720. doi: 10.3389/fbioe.2026.1731720 (PMC13047145; doi:10.3389/fbioe.2026.1731720)
Supplement: Supplementary file 1 [file DataSheet2.pdf]

Created by: Ayad, Younan

Created on: Sep 18, 2025

Item name: Lamiaceae Family +ve mode

Created time: 13:52:10 Egypt Standard Time

Analysis Information

Item name:	Lamiaceae Family +ve mode	Analysis Method Item name:	Extract 20min +ve libirary
Version:	2	Analysis Method Version:	1
Modified date:	Sep 18, 2025 13:04:26 Egypt Standard Time	Sample Set Created date:	
Modified by:	Ayad, Younan	Sample Set Instrument system name:	
Folder:	Company/LAB/data		

Analysis injection list

.	Item name	Item description	Replicate number	Sample position	Injection volume (µL)
1	Sep257+ve	Mervat253	1	1:A,2	0.50

Item name: Sep257+ve

Channel name: 2: TOF MSe (100-1200) 6V ESI+ (BPI) : Integrated : Smoothed

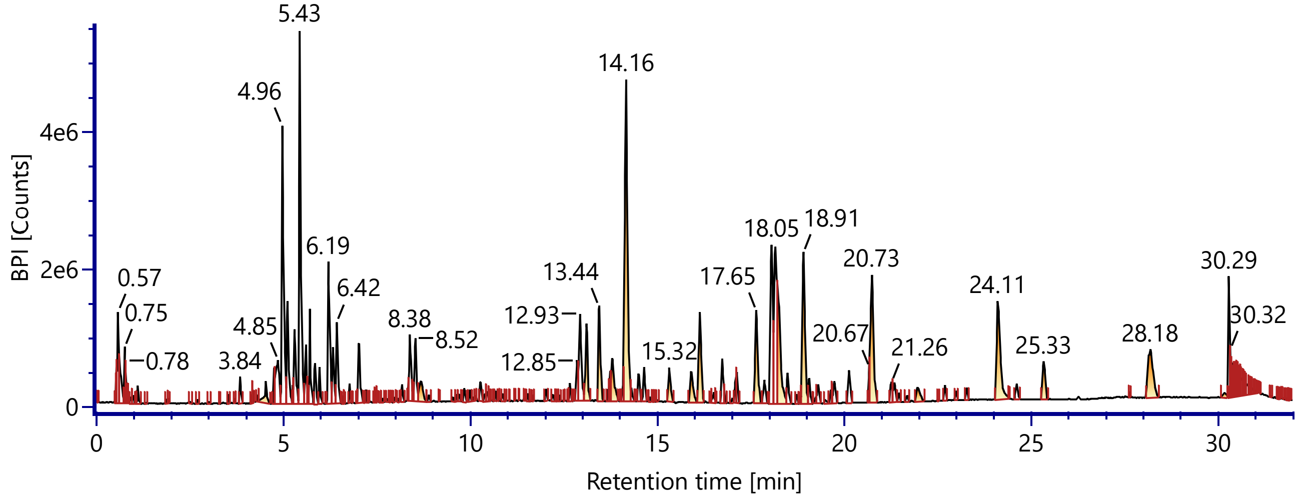

Item name: Sep257+ve

Item name: Sep257+ve
Channel name: 2: TOF MSe (100-1200) 6V ESI+ (BPI)

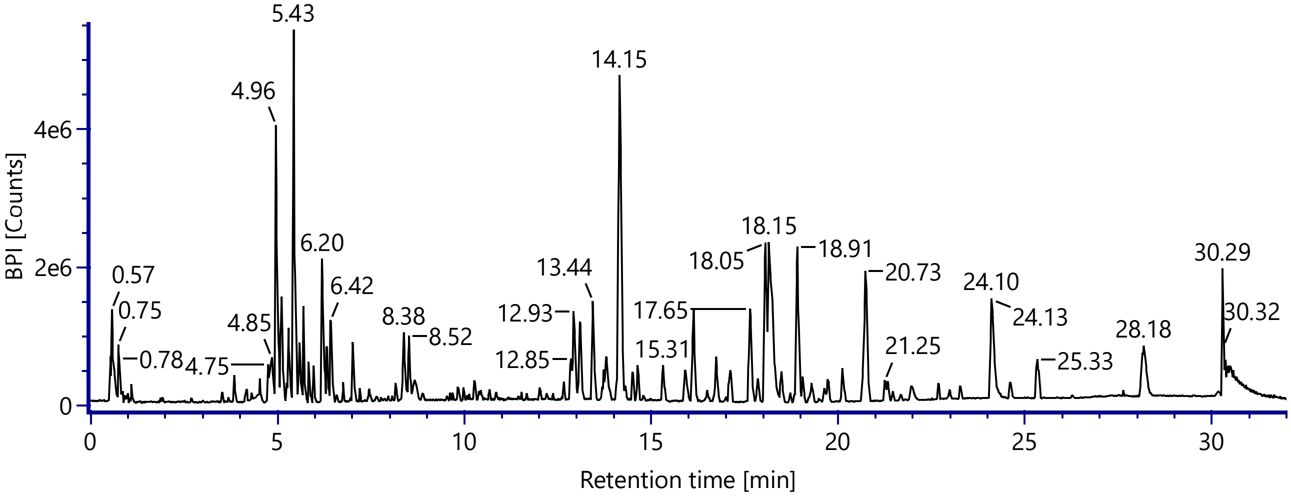

Item name: Sep257+ve, Sample position: 1:A,2, Replicate number: 1

.	Component name	Formula	Observed m/z	Mass error (ppm)	Observed RT (min)	Response	Adducts
1	6-O-Caffeoyl glucoside	C15H18O10	381.0802	2.4	0.58	225487	+Na
2	5-O-Caffeoylquinic acid butyl ester	C20H26O9	411.1663	3.3	3.53	158900	+H
3	Apigenin-7-O-gentiobioside	C27H30O15	595.1660	0.5	3.84	301092	+H, +Na
4	Kaempferol-3-O-glucoside	C21H20O11	449.1085	1.5	4.53	47417	+H, +Na
5	Luteolin	C15H10O6	287.0549	-0.3	4.53	162597	+H
6	5alpha,6beta-Dihydroxydaucosterol	C35H62O8	633.4318	-2.9	4.67	49298	+Na
7	Kaempferol 7-O-β-D-glucopyranosyl (1→4)β-D-glucopyranoside	C27H30O16	633.1433	1.0	4.73	38579	+Na, +H
8	Quercetin dihydrate	C15H10O7	303.0502	0.9	4.73	32033	+H
9	Apigenin 7-glucoside	C21H20O10	433.1137	1.9	4.76	238901	+H, +Na
10	Kaempferol-3-O-glucoside	C21H20O11	449.1090	2.5	4.93	49068	+H
11	Kaempferol	C15H10O6	287.0552	0.5	4.96	36784	+H

.	Component name	Formula	Observed m/z	Mass error (ppm)	Observed RT (min)	Response	Adducts
12	Kaempferol-3-glucuronide	C21H18O12	463.0881	2.2	4.97	2259985	+H, +Na
13	Querciturone	C21H18O13	479.0831	2.3	4.97	51947	+H
14	1,3-Bis-[2-(3,4-dihydroxyphenyl)-1-carboxy]ethoxycarbonyl-2-(3,4-dihydroxyphenyl)-7,8-dihydroxy-1,2-dihydronaphthalene	C36H30O16	741.1431	0.6	5.25	153371	+Na
15	Kaempferol-O3-alpha-rhamnopyranoside	C21H20O10	433.1138	1.9	5.36	41210	+H
16	Apigenin	C15H10O5	271.0603	0.7	5.43	40159	+H
17	Apigenin-4'-O-glucuronide	C21H18O11	447.0928	1.4	5.43	2853122	+H, +Na
18	Luteolin-7-O-(6′′-methyl ester)-β-D-glucuronide	C22H20O12	477.1037	2.0	5.50	127409	+H
19	Rosmarinic acid	C18H16O8	383.0741	1.0	5.60	121971	+Na, +H
20	trans Caffeic acid	C9H8O4	181.0499	2.1	5.60	62894	+H
21	7,8-Dihydroxy-2-(3,4-dihydroxyphenyl)-1,2-dihydronaphthalene-1,3-dicarboxylic acid	C18H14O8	381.0589	2.1	5.60	75603	+Na, +H
22	Quercilicoside A	C36H58O11	689.3875	0.5	5.78	55352	+Na
23	2-O-Caffeoyl arbutin	C21H22O10	435.1265	-4.8	6.24	89289	+H
24	Luteolin	C15H10O6	287.0547	-1.2	6.33	438816	+H
25	Quercetin 3'-methyl ether	C16H12O7	317.0655	-0.4	6.63	33327	+H
26	(E)-Calamenene	C15H22	203.1795	0.6	6.96	44283	+H
27	Apigenin	C15H10O5	271.0598	-1.0	7.02	388072	+H
28	Diosmetin	C16H12O6	301.0718	3.6	7.16	45464	+H
29	Quercetin 3,4'-dimethyl ether	C17H14O7	331.0811	-0.5	7.34	38673	+H

.	Component name	Formula	Observed m/z	Mass error (ppm)	Observed RT (min)	Response	Adducts
30	7,8-Dihydroxy-2-(3,4-dihydroxyphenyl)-1,2-dihydronaphthalene-1,3-dicarboxylic acid	C18H14O8	381.0582	0.2	7.47	20605	+Na
31	Diosmetin	C16H12O6	301.0714	2.3	7.53	32690	+H
32	Quercetin 3,4'-dimethyl ether	C17H14O7	331.0808	-1.2	7.66	69525	+H
33	(1aR,4aS,7R,7aR,7bR)-1,1,7-Trimethyl-4-methylidenedecahydro-1H-cyclopropa(e)azulen-7-ol	C15H24O	221.1898	-0.8	8.19	72426	+H
34	(E)-Calamenene	C15H22	203.1794	-0.1	8.19	89514	+H
35	(E)-Calamenene	C15H22	203.1795	0.2	8.38	40113	+H
36	Quercetin tetramethyl (3',4',5,7) ether	C19H18O7	359.1133	2.2	12.24	81674	+H, +Na
37	(E)-Calamenene	C15H22	203.1796	1.0	13.33	45251	+H
38	(E)-Calamenene	C15H22	203.1795	0.4	13.69	135241	+H
39	(E)-Calamenene	C15H22	203.1794	0.1	13.82	297984	+H
40	(E)-Calamenene	C15H22	203.1795	0.6	14.06	91175	+H
41	(E)-Calamenene	C15H22	203.1795	0.6	14.30	191246	+H
42	(E)-Calamenene	C15H22	203.1795	0.6	15.32	332380	+H
43	(1aR,4aS,7R,7aR,7bR)-1,1,7-Trimethyl-4-methylidenedecahydro-1H-cyclopropa(e)azulen-7-ol	C15H24O	221.1900	0.1	15.32	46966	+H, +Na
44	beta-Bourbonene	C15H24	205.1952	0.7	15.48	34695	+H
45	7alpha-Hydroxycampesterol	C28H48O2	439.3565	4.2	17.64	1341678	+Na
46	7alpha-Hydroxycampesterol	C28H48O2	439.3567	4.8	18.15	3705053	+Na
47	(E)-Calamenene	C15H22	203.1794	0.1	18.16	58181	+H
48	alpha-Amorphene	C15H24	205.1949	-0.6	18.23	20053	+H

.	Component name	Formula	Observed m/z	Mass error (ppm)	Observed RT (min)	Response	Adducts
49	3-Epioleanolic acid	C30H48O3	457.3671	-1.1	18.23	118726	+H
50	25-Dehydrochondrillasterol	C29H46O	411.3620	-0.4	18.23	718565	+H
51	α-Spinasterol	C29H48O	413.3779	0.2	24.61	53334	+H
52	5alpha,6beta-Dihydroxydaucosterol	C35H62O8	611.4538	3.3	25.26	40670	+H
53	beta-Sitosterol-alpha-glucoside	C35H60O6	599.4283	0.1	26.28	22420	+Na
54	(24s)-Saringosterol	C29H48O2	429.3732	1.2	30.24	31216	+H
55	(24s)-Saringosterol	C29H48O2	429.3730	0.6	30.32	42591	+H
56	(24s)-Saringosterol	C29H48O2	429.3726	-0.2	30.39	26183	+H

Component name: 6-O-Caffeoyl glucoside

Item name: Sep257+ve
Channel name: 6-O-Caffeoyl glucoside [+Na] : (48.1 PPM) 381.0802

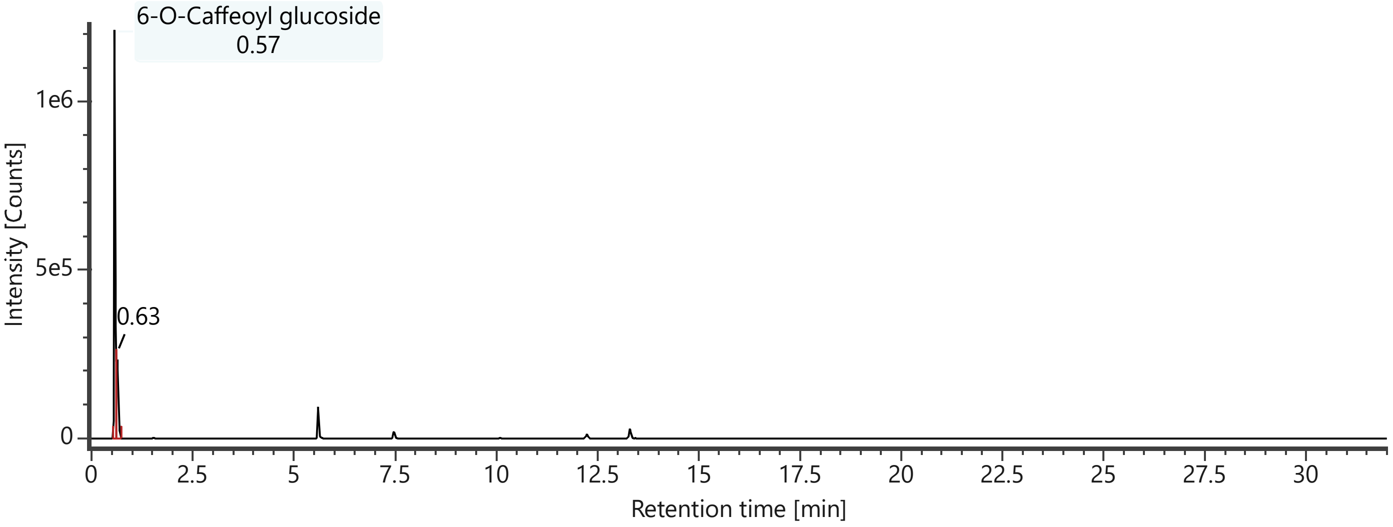

Item name: Sep257+ve
Item description: Mervat253
Channel name: Low energy : Time 0.5776 +/- 0.0237 minutes

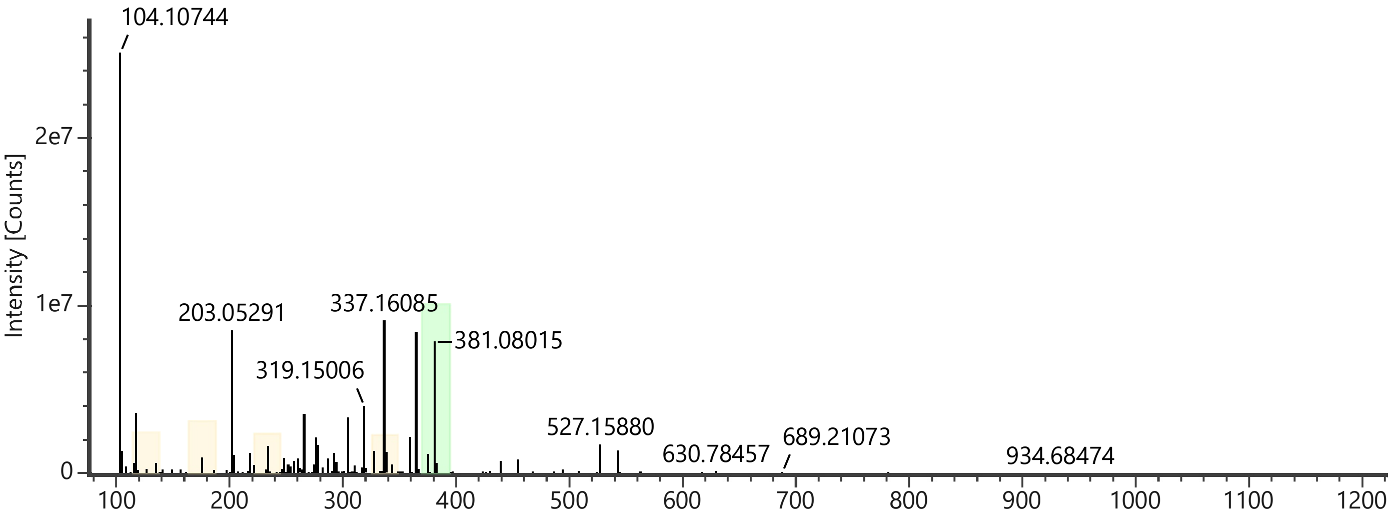

Item name: Sep257+ve
Item description: Mervat253
Channel name: High energy : Time 0.5776 +/- 0.0237 minutes

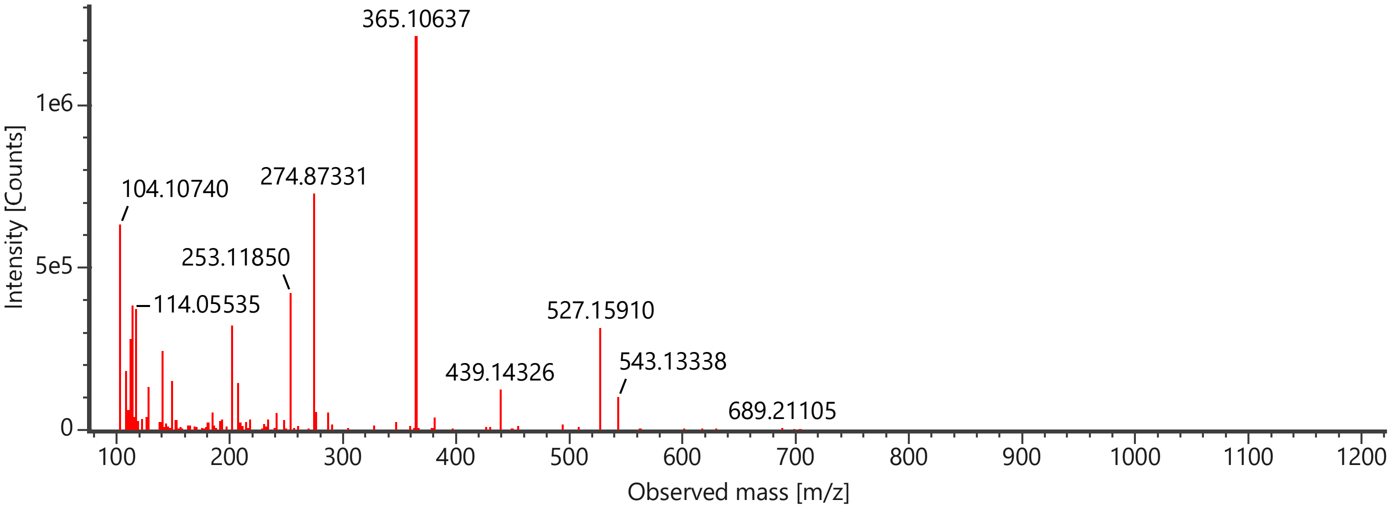

Component name: 5-O-Caffeoylquinic acid butyl ester

Item name: Sep257+ve
Channel name: 5-O-Caffeoylquinic acid butyl ester [+H] : (48.1 PPM) 411.1663

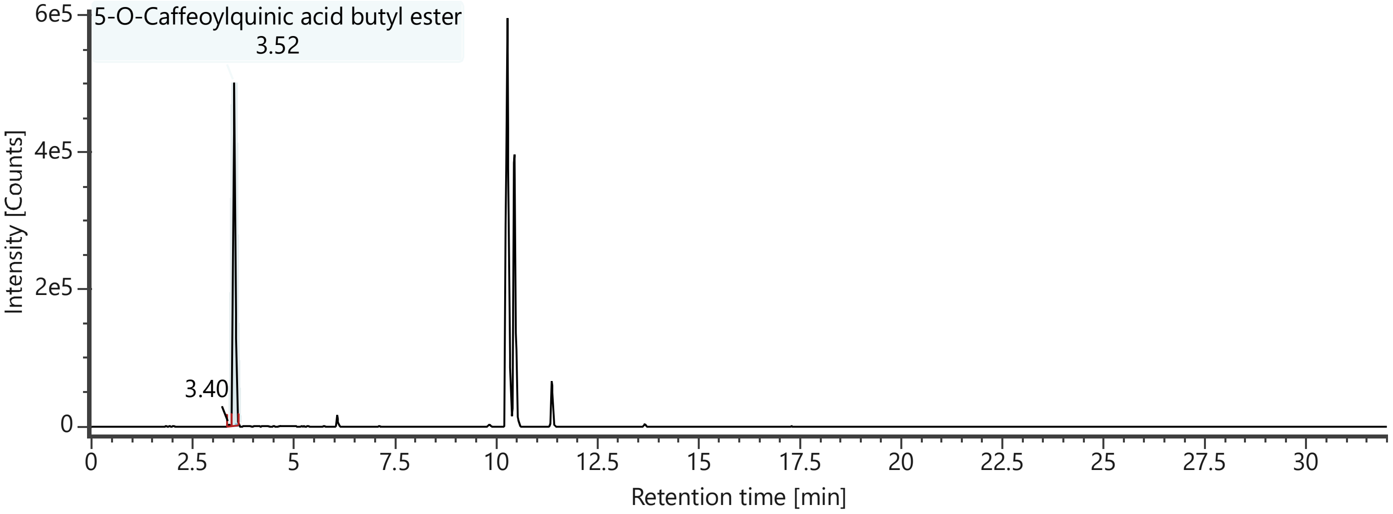

Item name: Sep257+ve
Item description: Mervat253
Channel name: Low energy : Time 3.5278 +/- 0.0237 minutes

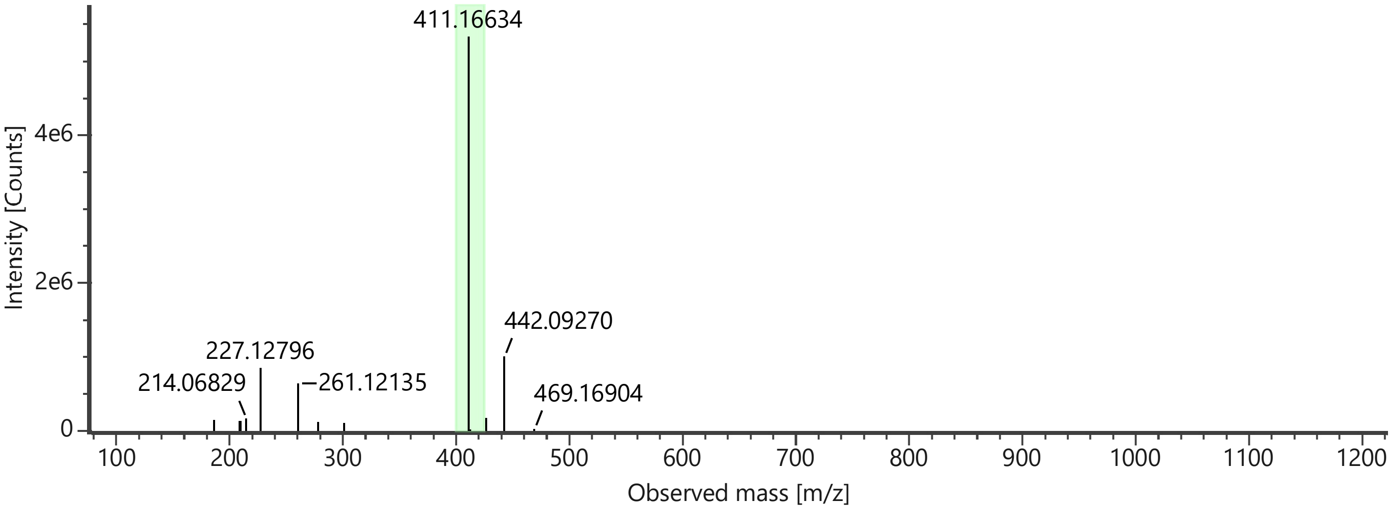

Item name: Sep257+ve
Item description: Mervat253

Channel name: High energy : Time 3.5278 +/- 0.0237 minutes

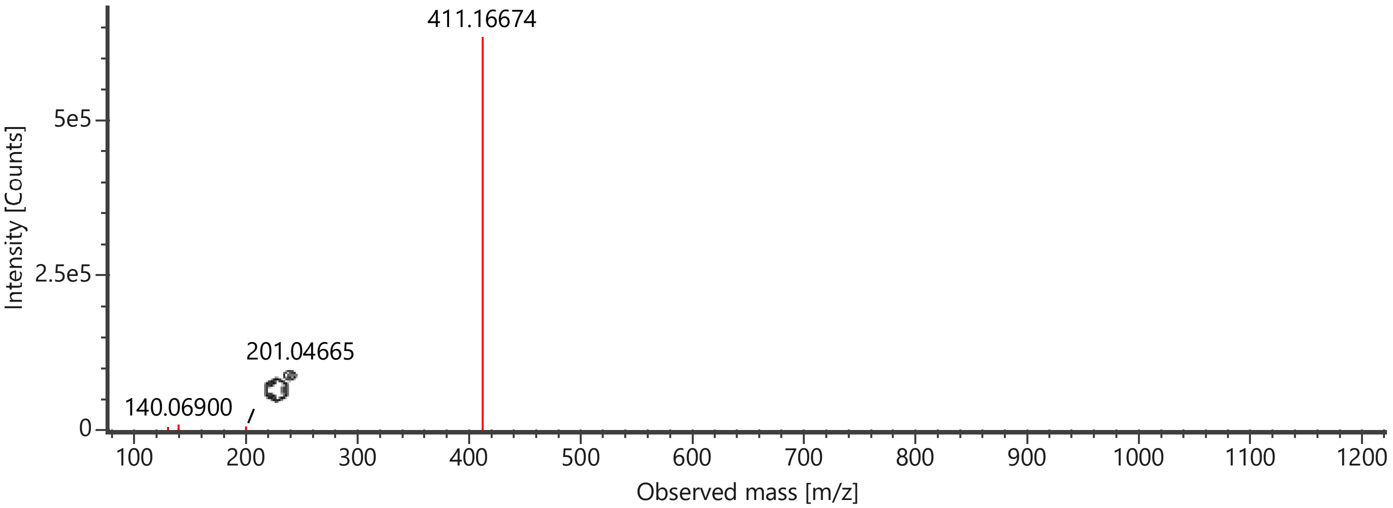

Component name: Apigenin-7-O-gentiobioside

Item name: Sep257+ve
Channel name: Apigenin-7-O-gentiobioside [+H] : (48.1 PPM) 595.1660

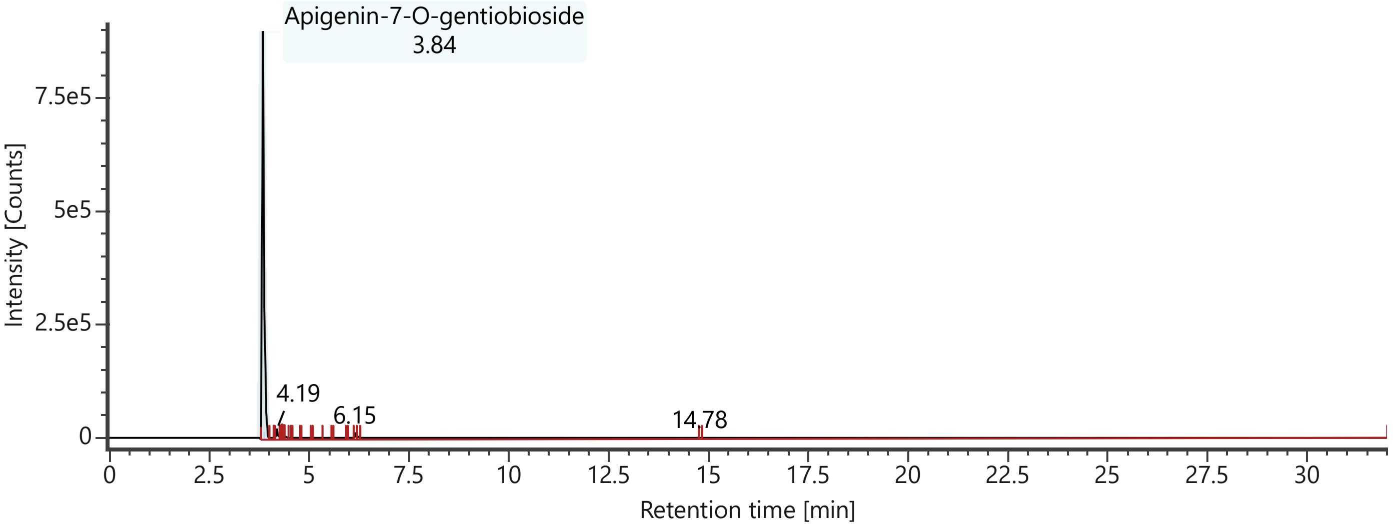

Item name: Sep257+ve
Item description: Mervat253
Channel name: Low energy : Time 3.8405 +/- 0.0237 minutes

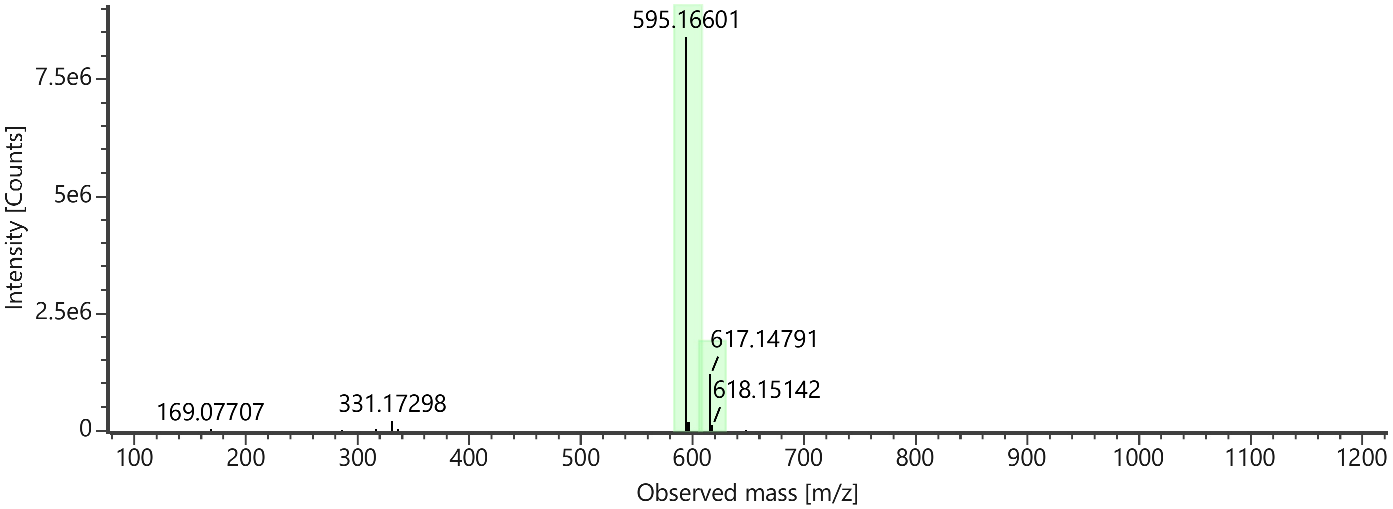

Item name: Sep257+ve
Item description: Mervat253

Channel name: High energy : Time 3.8405 +/- 0.0237 minutes

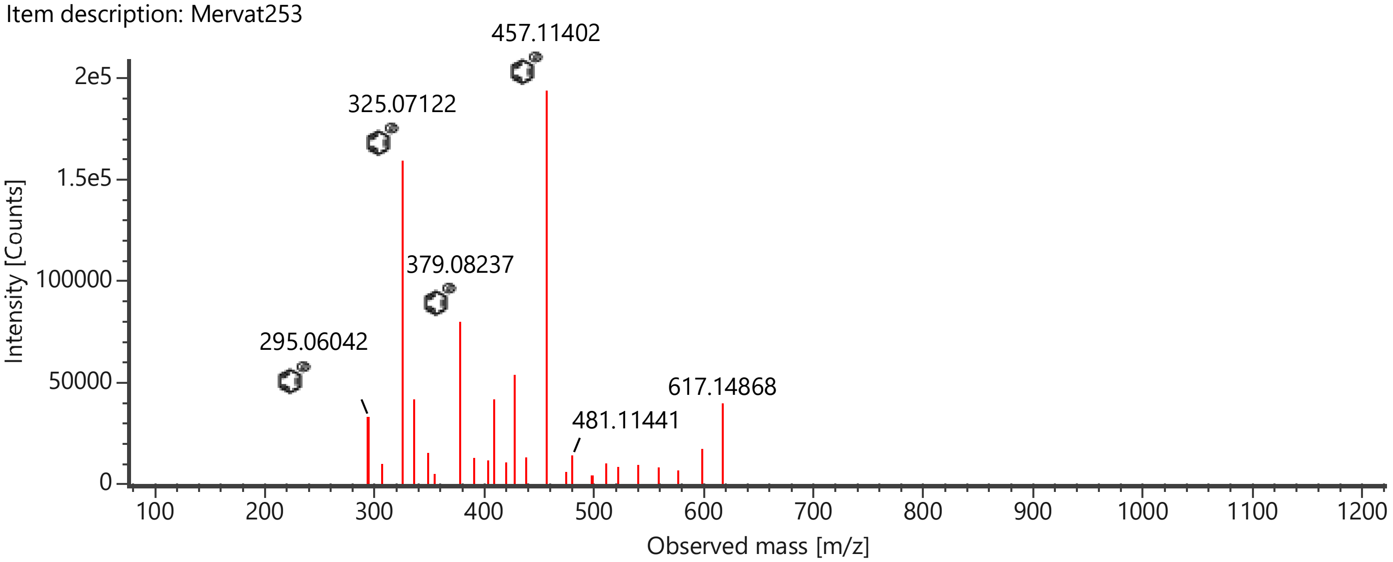

Component name: Kaempferol-3-O-glucoside

Item name: Sep257+ve
Channel name: Kaempferol-3-O-glucoside [+H] : (48.1 PPM) 449.1085

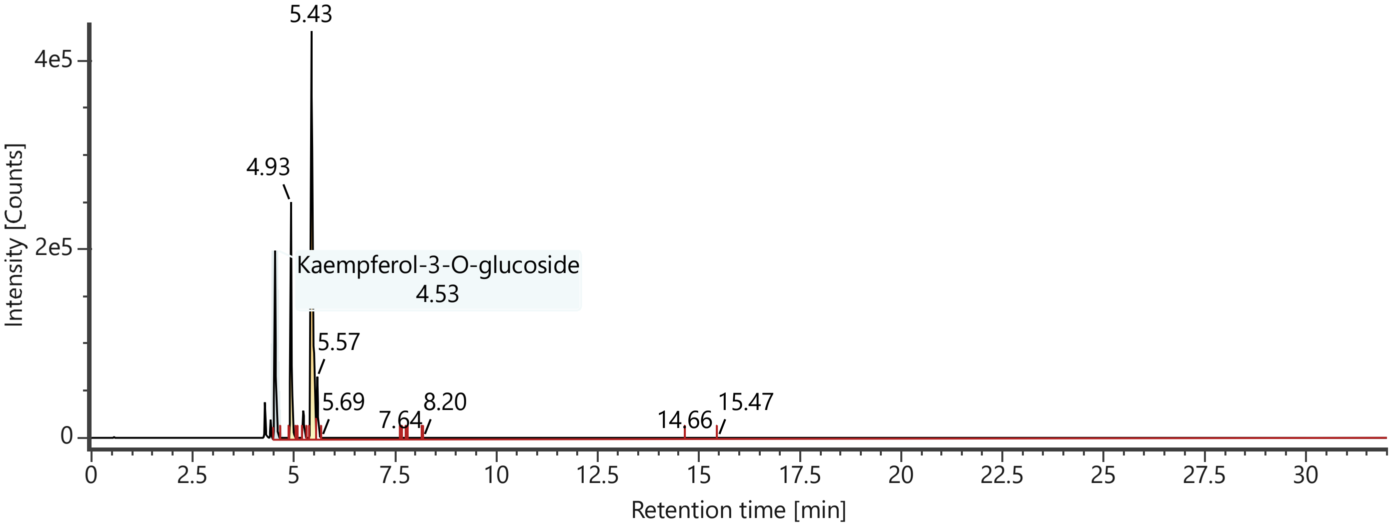

Item name: Sep257+ve
Item description: Mervat253
Channel name: Low energy : Time 4.5330 +/- 0.0237 minutes

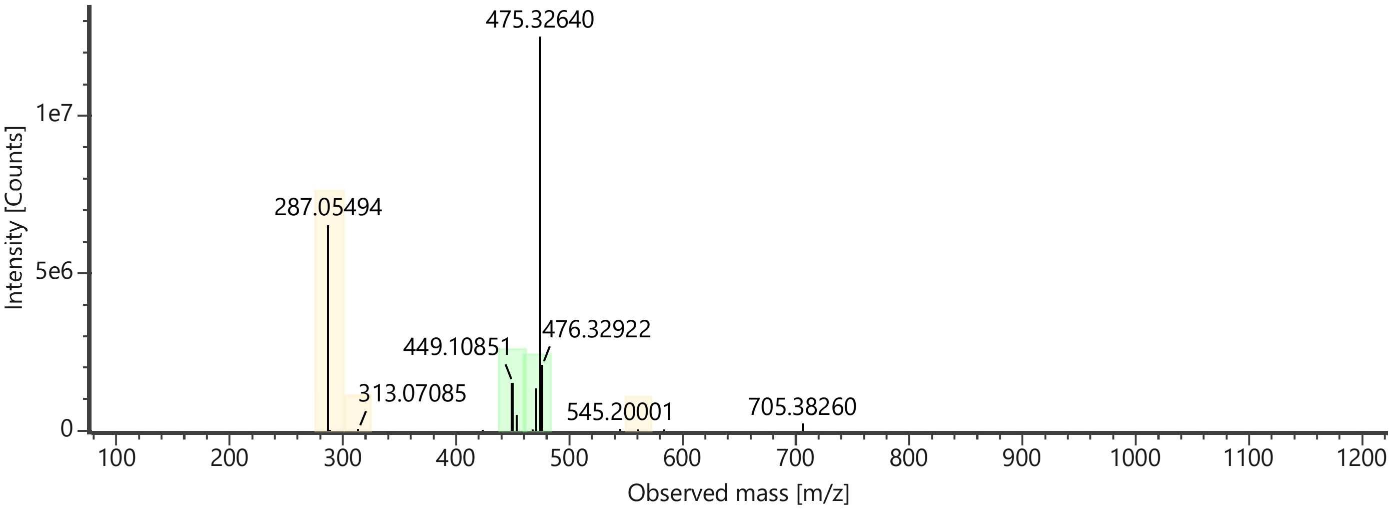

Item name: Sep257+ve
Item description: Mervat253

Channel name: High energy : Time 4.5330 +/- 0.0237 minutes

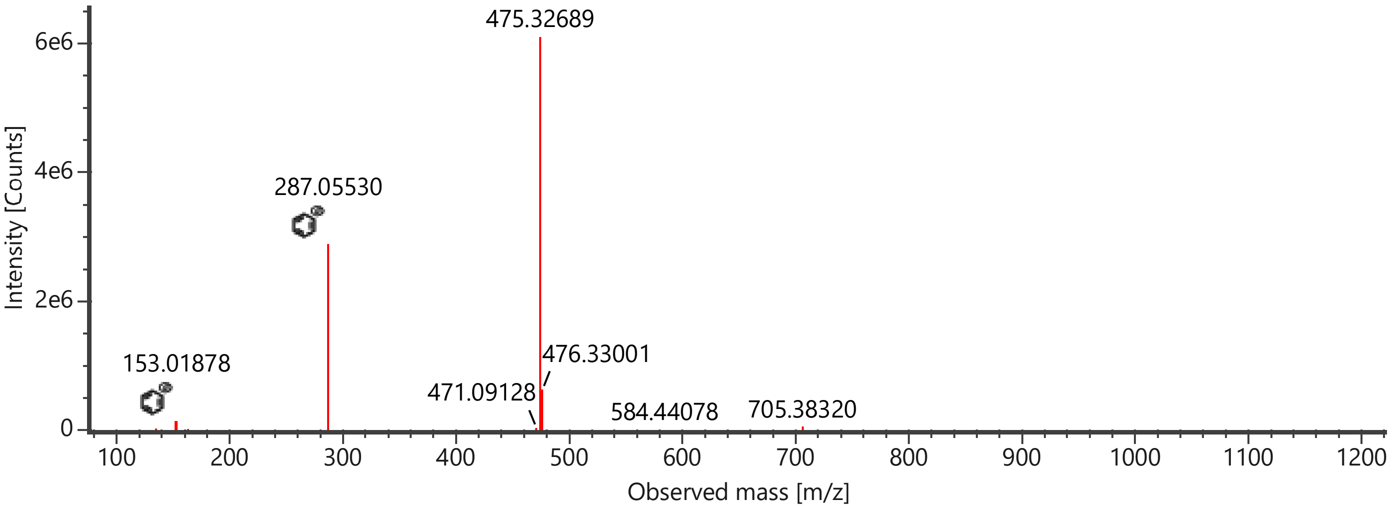

Component name: Luteolin

Item name: Sep257+ve
Channel name: Luteolin [+H] : (48.1 PPM) 287.0549

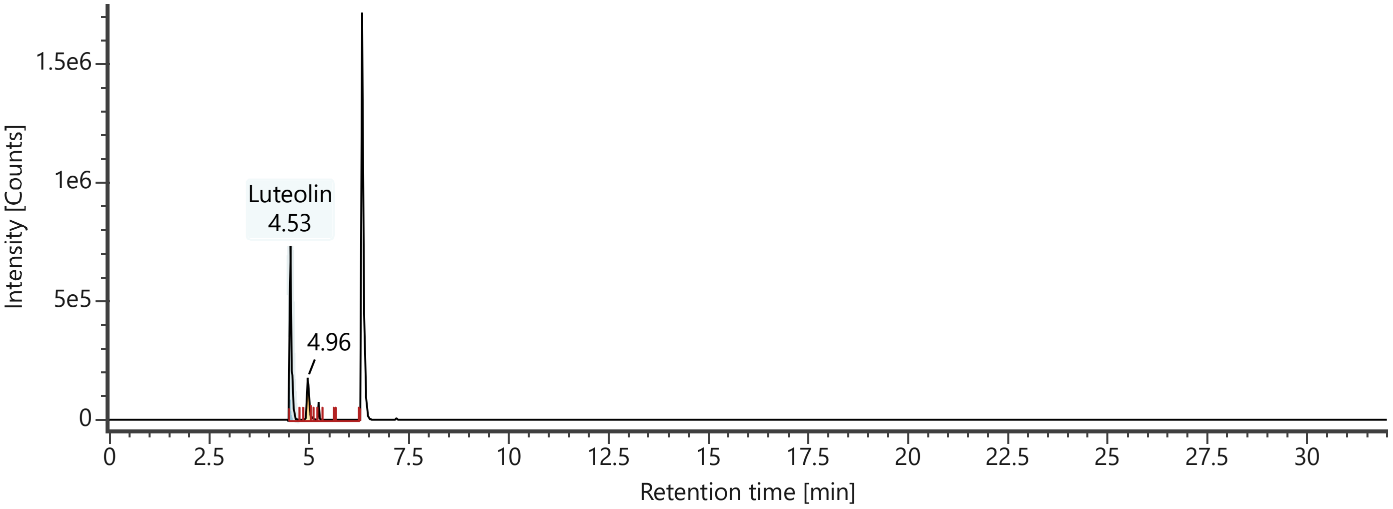

Item name: Sep257+ve
Item description: Mervat253
Channel name: Low energy : Time 4.5335 +/- 0.0237 minutes

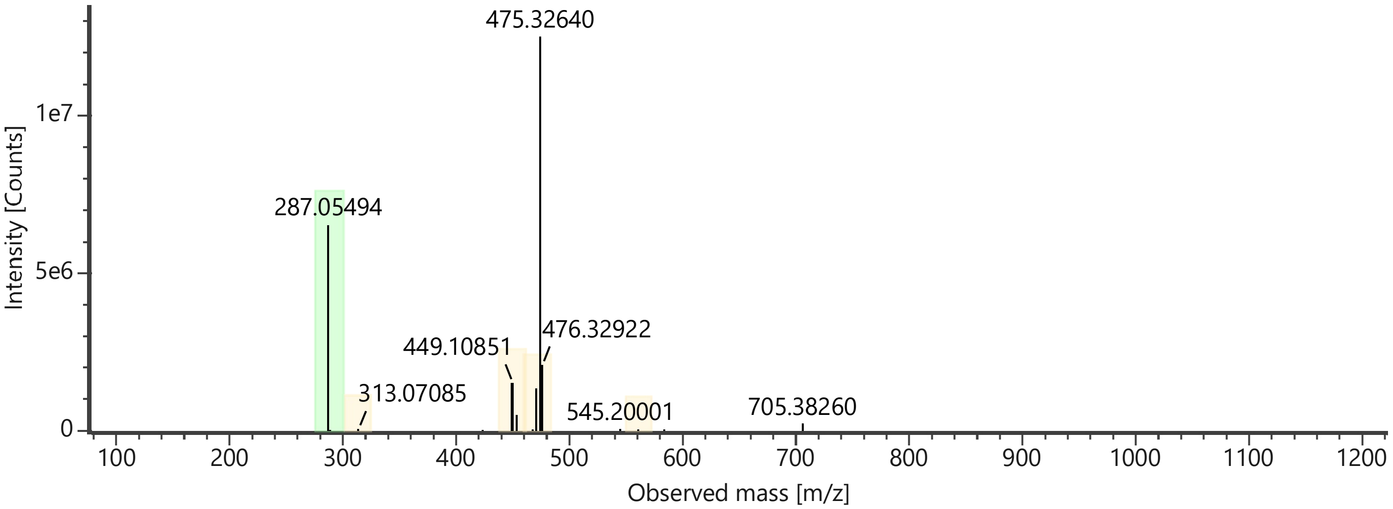

Created by: Ayad, Younan

Created on: Sep 18, 2025

Item name: Lamiaceae Family +ve mode

Created time: 13:52:10 Egypt Standard Time

Item name: Sep257+ve
Item description: Mervat253

Channel name: High energy : Time 4.5335 +/- 0.0237 minutes

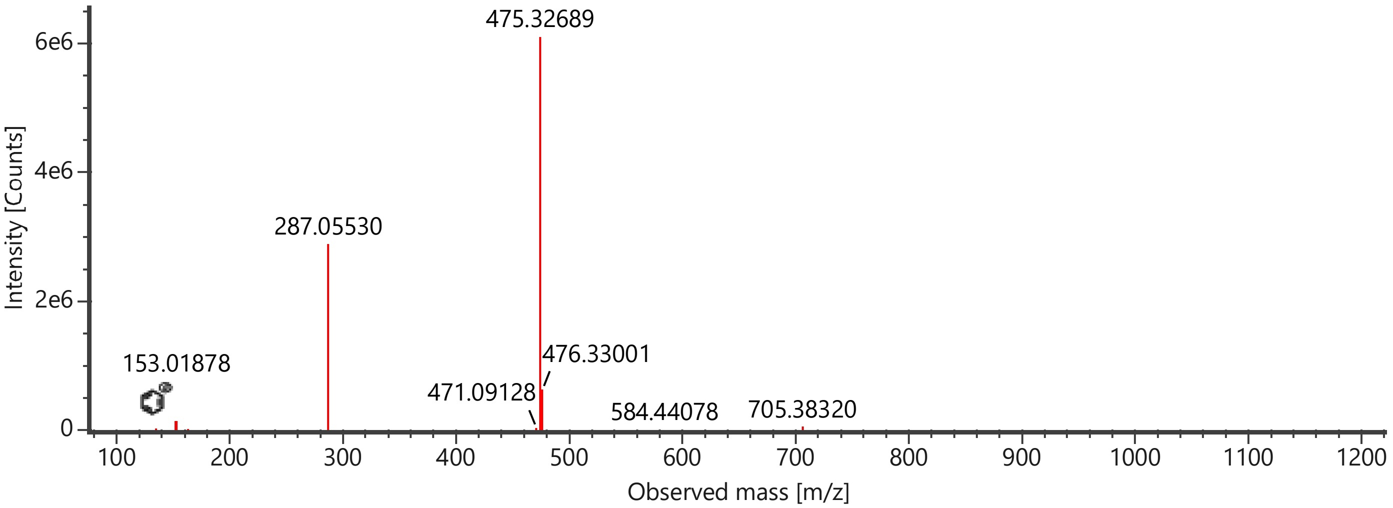

Component name: 5alpha,6beta-Dihydroxydaucosterol

Item name: Sep257+ve
Channel name: 5alpha,6beta-Dihydroxydaucosterol [+Na] : (48.1 PPM) 633.4318

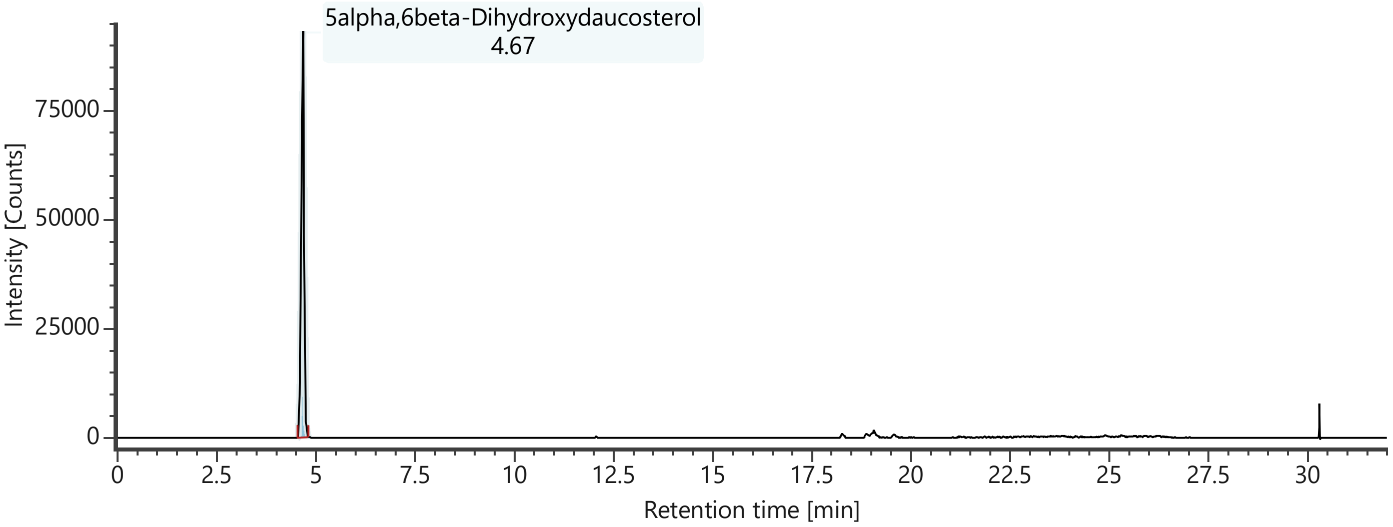

Item name: Sep257+ve
Item description: Mervat253
Channel name: Low energy : Time 4.6663 +/- 0.0237 minutes

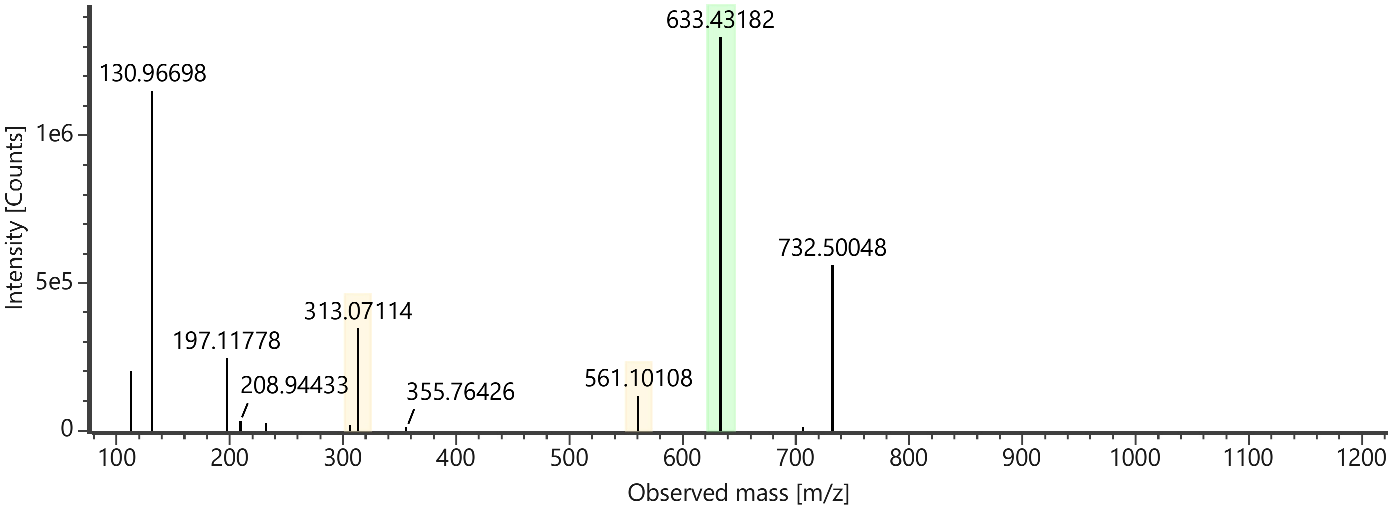

Item name: Sep257+ve
Item description: Mervat253

Channel name: High energy : Time 4.6663 +/- 0.0237 minutes

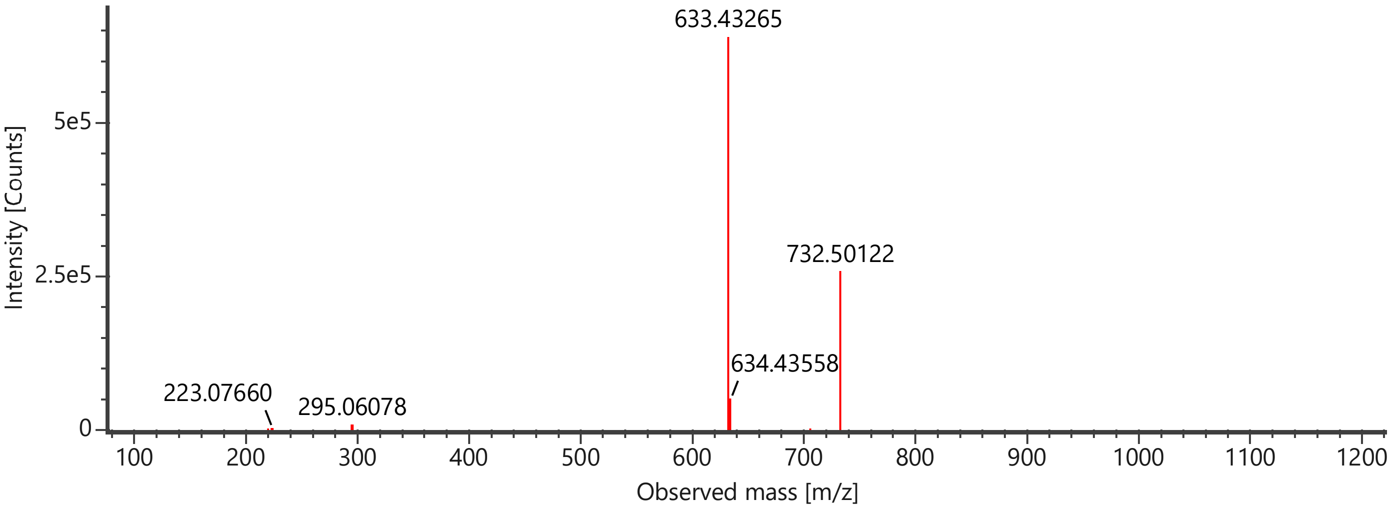

Component name: Kaempferol 7-O-β-D-glucopyranosyl(1→4)β-D-glucopyranoside

Item name: Sep257+ve
Channel name: Kaempferol 7-O-β-D-glucopyranosyl(1→4)β-D-glucopyranoside [+Na] : (48.1 PPM) 633.1433

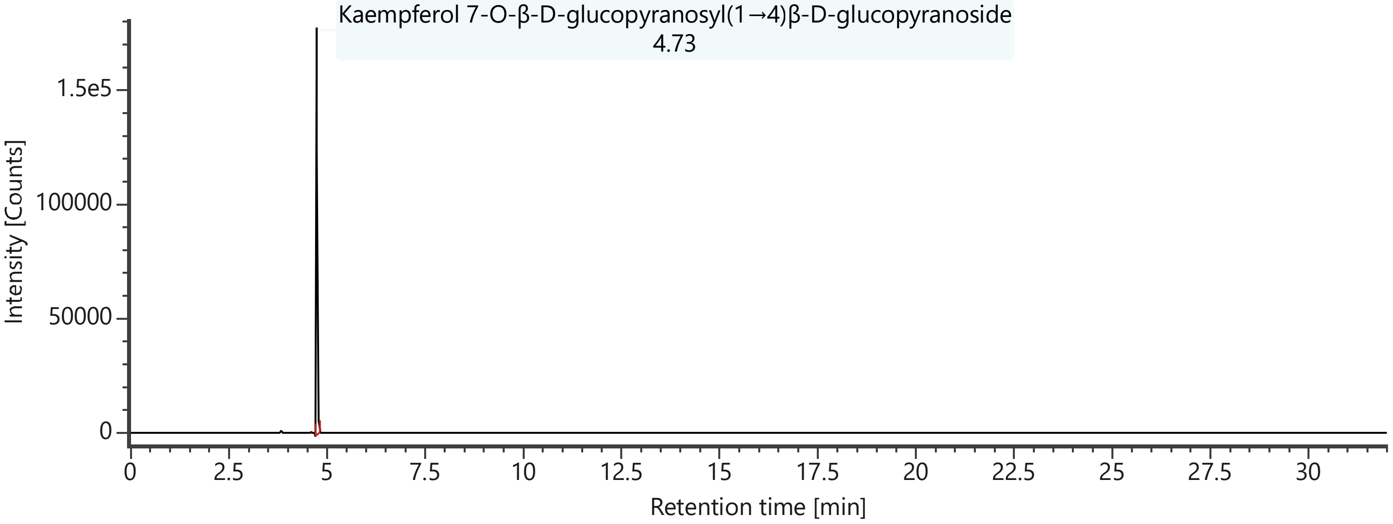

Item name: Sep257+ve
Item description: Mervat253
Channel name: Low energy : Time 4.7324 +/- 0.0237 minutes

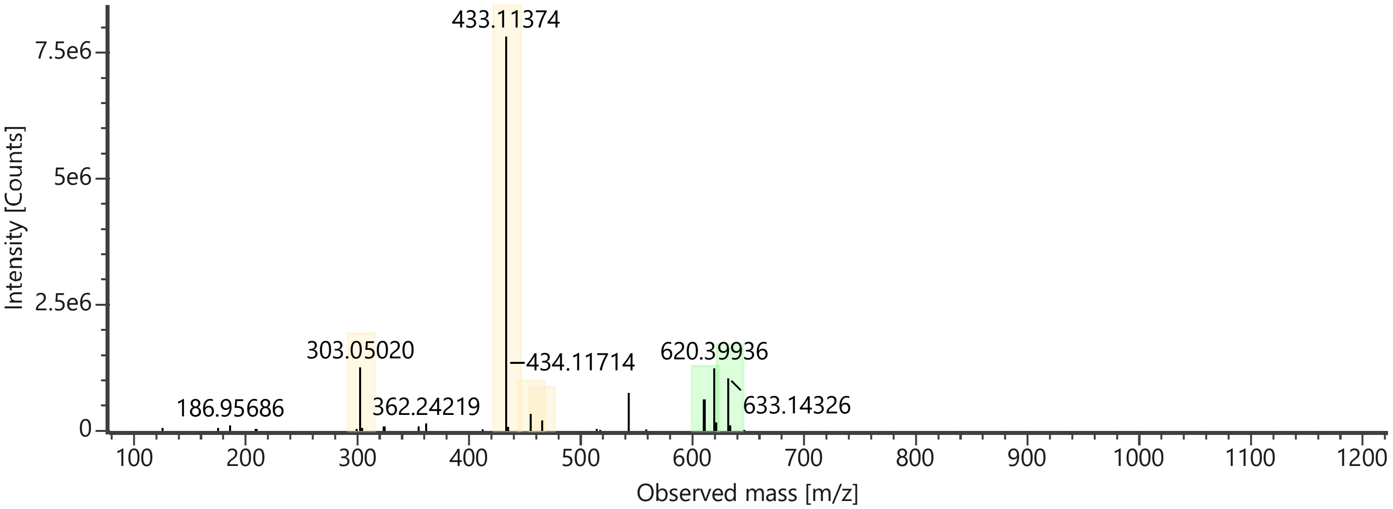

Created by: Ayad, Younan

Created on: Sep 18, 2025

Item name: Lamiaceae Family +ve mode

Created time: 13:52:10 Egypt Standard Time

Item name: Sep257+ve
Item description: Mervat253

Channel name: High energy : Time 4.7324 +/- 0.0237 minutes

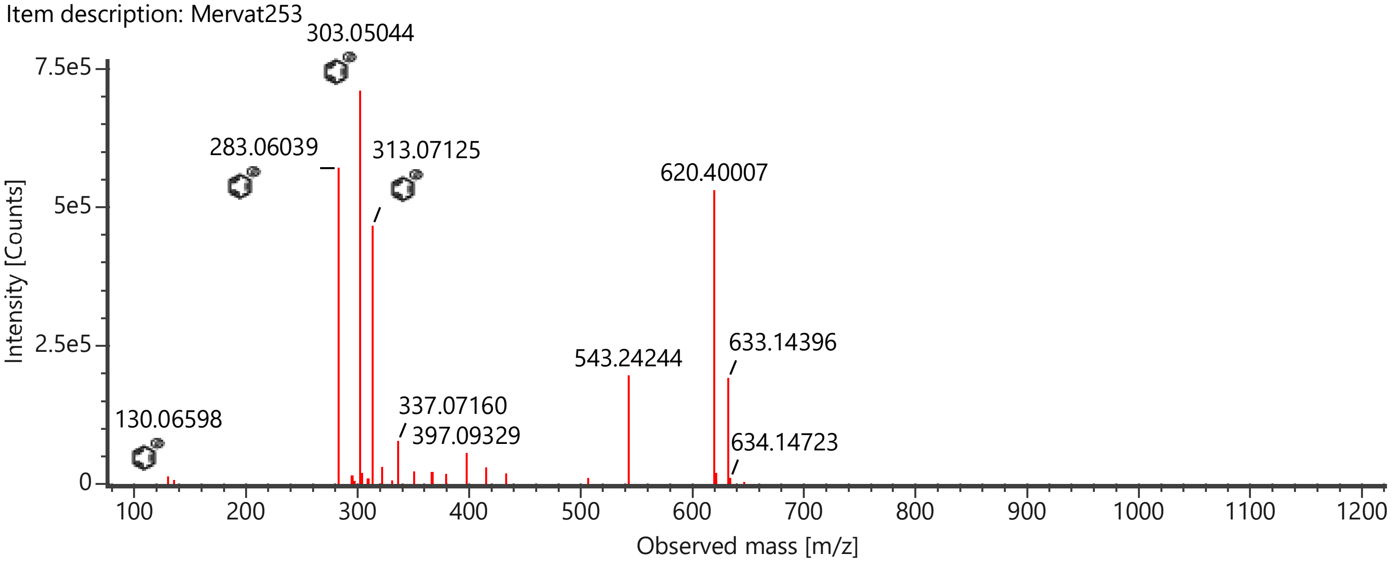

Component name: Quercetin dihydrate

Item name: Sep257+ve
Channel name: Quercetin dihydrate [+H] : (48.1 PPM) 303.0502

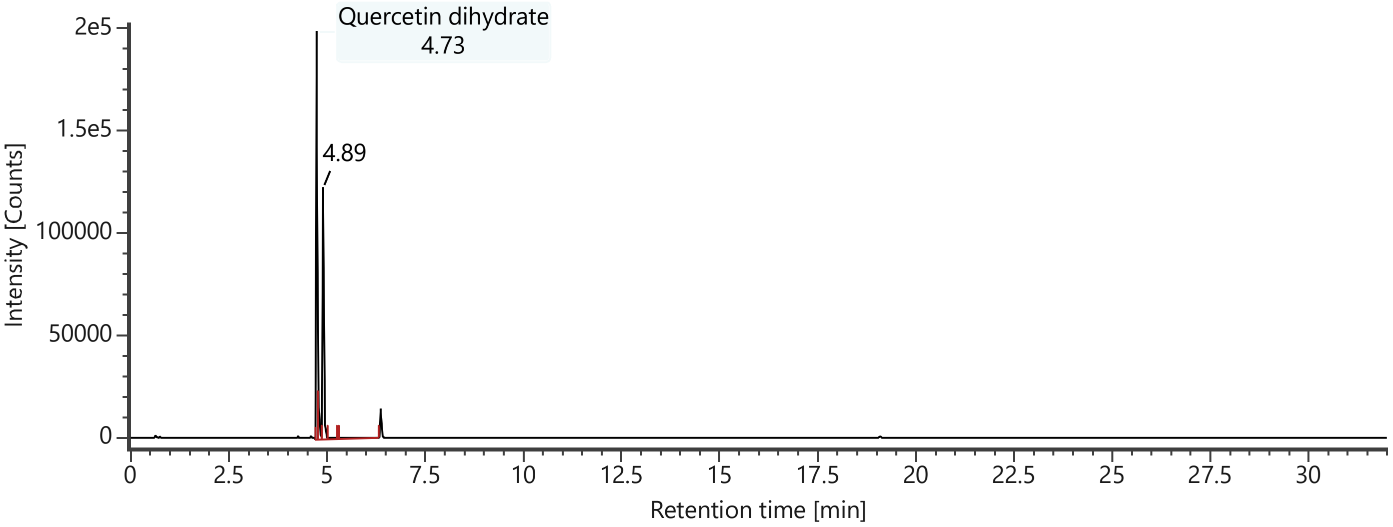

Item name: Sep257+ve
Item description: Mervat253

Channel name: Low energy : Time 4.7324 +/- 0.0237 minutes

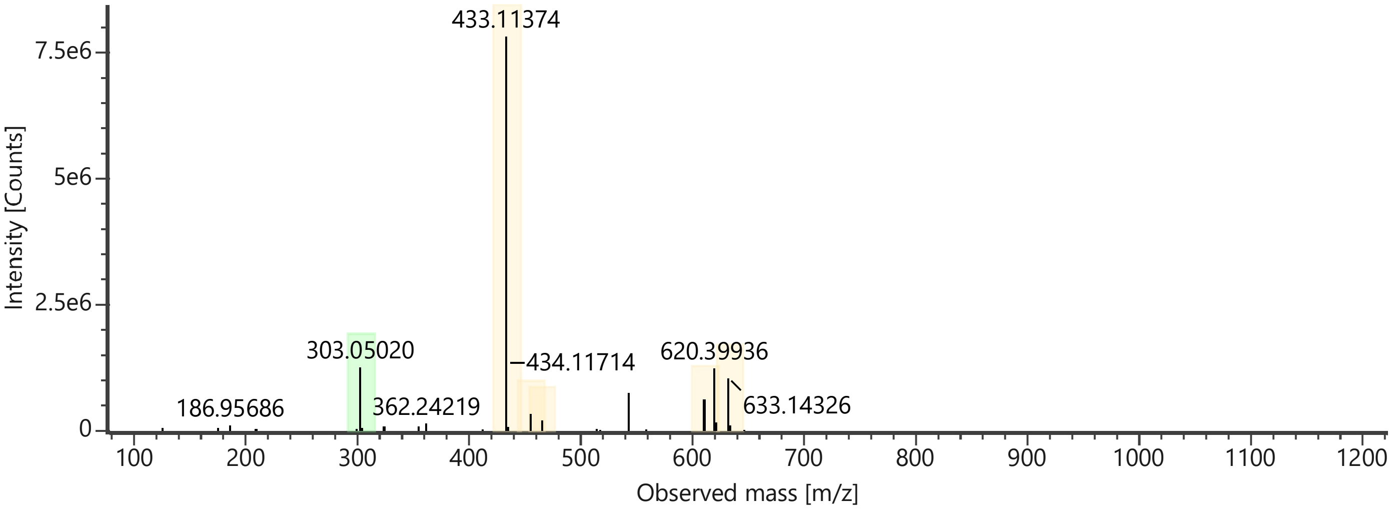

Item name: Sep257+ve
Item description: Mervat253

Channel name: High energy : Time 4.7324 +/- 0.0237 minutes

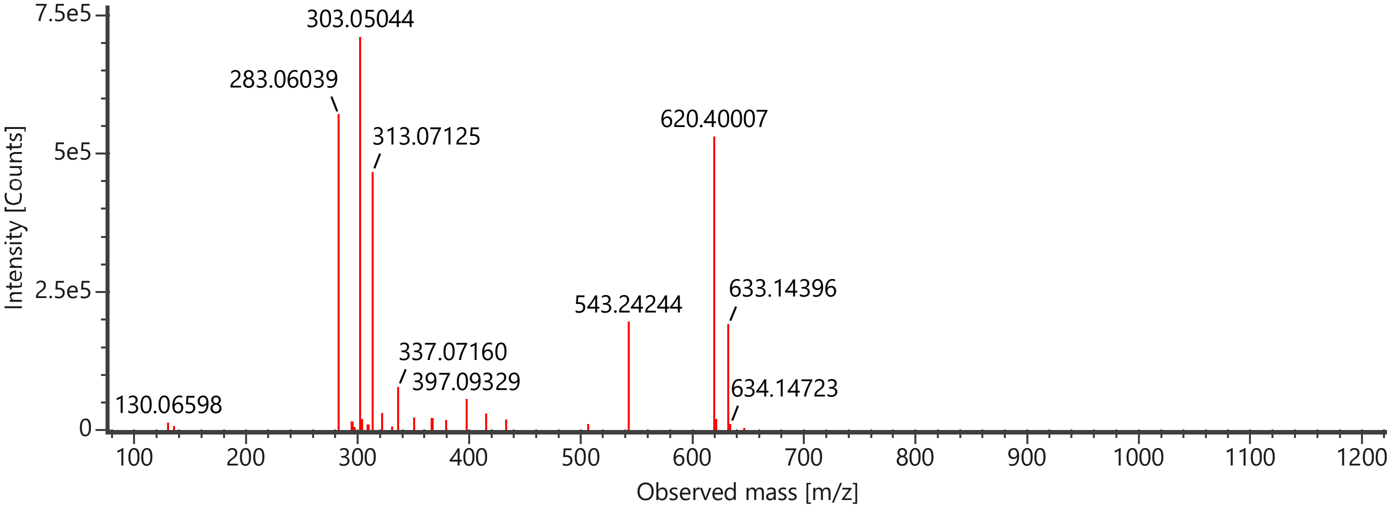

Component name: Apigenin 7-glucoside

Item name: Sep257+ve
Channel name: Apigenin 7-glucoside [+H] : (48.1 PPM) 433.1137

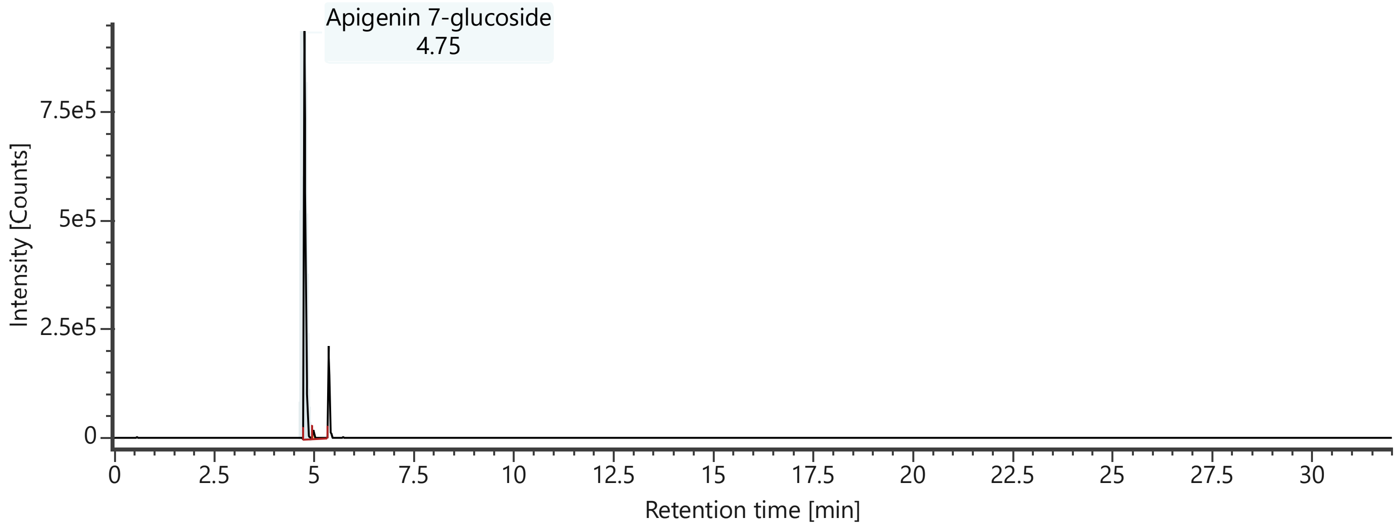

Item name: Sep257+ve
Item description: Mervat253

Channel name: Low energy : Time 4.7556 +/- 0.0237 minutes

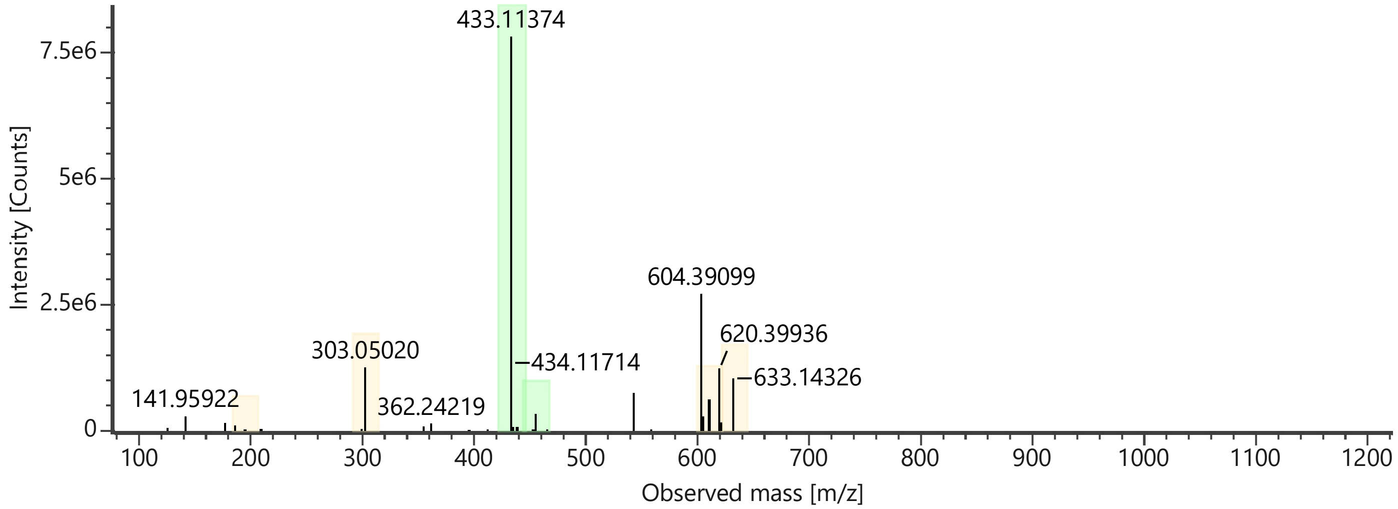

Item name: Sep257+ve
Item description: Mervat253

Channel name: High energy : Time 4.7556 +/- 0.0237 minutes

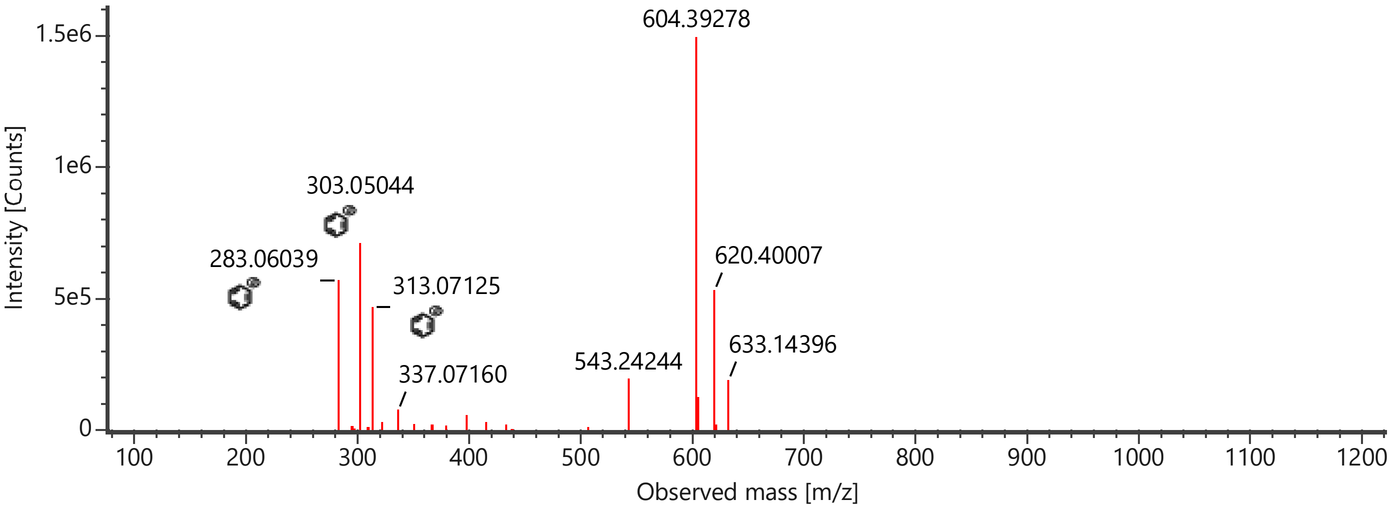

Component name: Kaempferol-3-O-glucoside

Item name: Sep257+ve
Channel name: Kaempferol-3-O-glucoside [+H] : (48.1 PPM) 449.1090

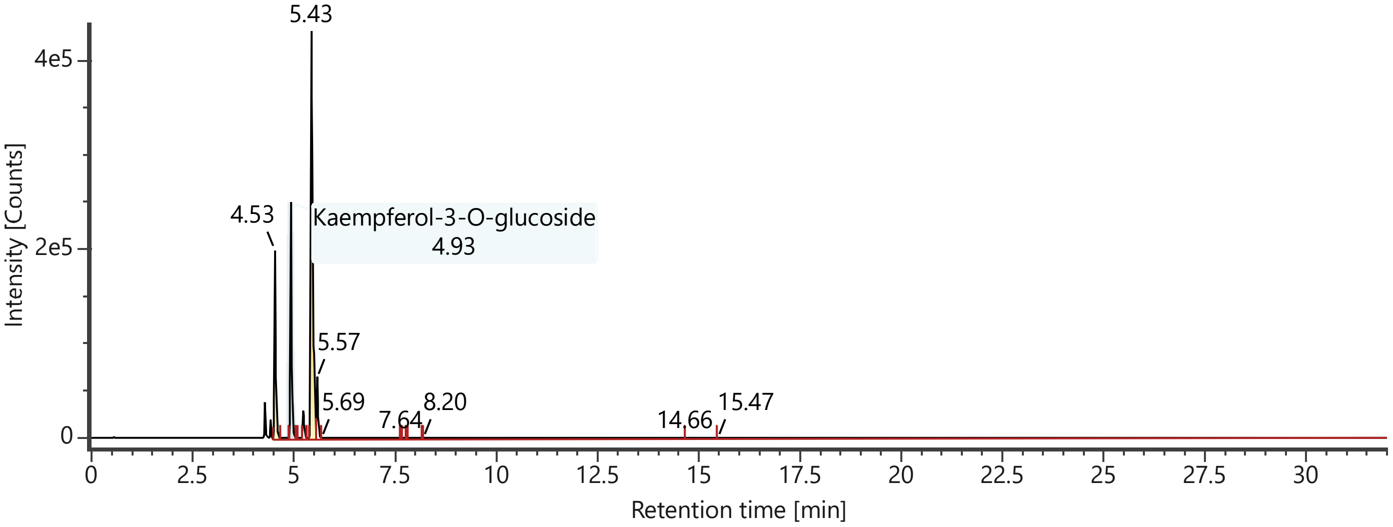

Item name: Sep257+ve
Item description: Mervat253
Channel name: Low energy : Time 4.9285 +/- 0.0237 minutes

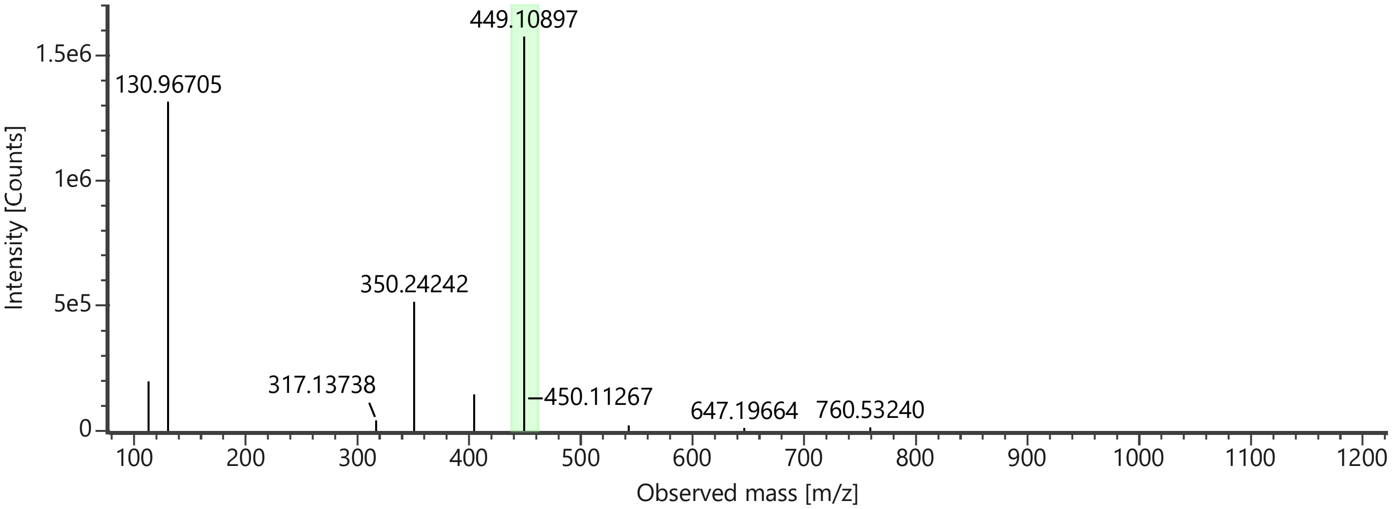

Created by: Ayad, Younan

Created on: Sep 18, 2025

Item name: Lamiaceae Family +ve mode

Created time: 13:52:10 Egypt Standard Time

Item name: Sep257+ve
Item description: Mervat253

Channel name: High energy : Time 4.9285 +/- 0.0237 minutes

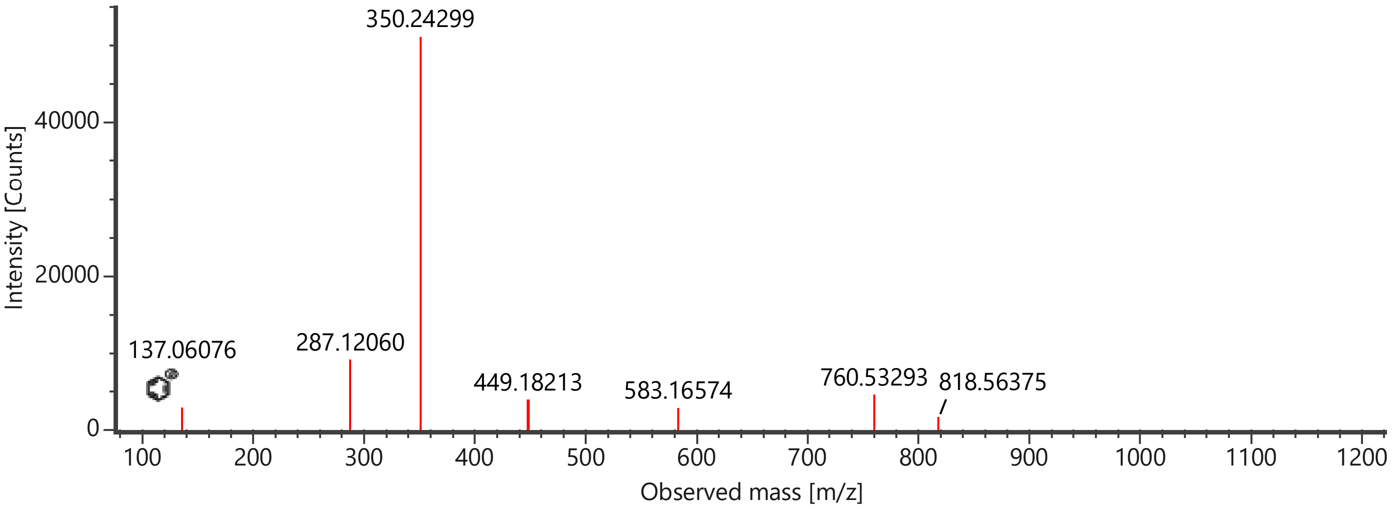

Component name: Kaempferol

Item name: Sep257+ve
Channel name: Kaempferol [+H] : (48.1 PPM) 287.0552

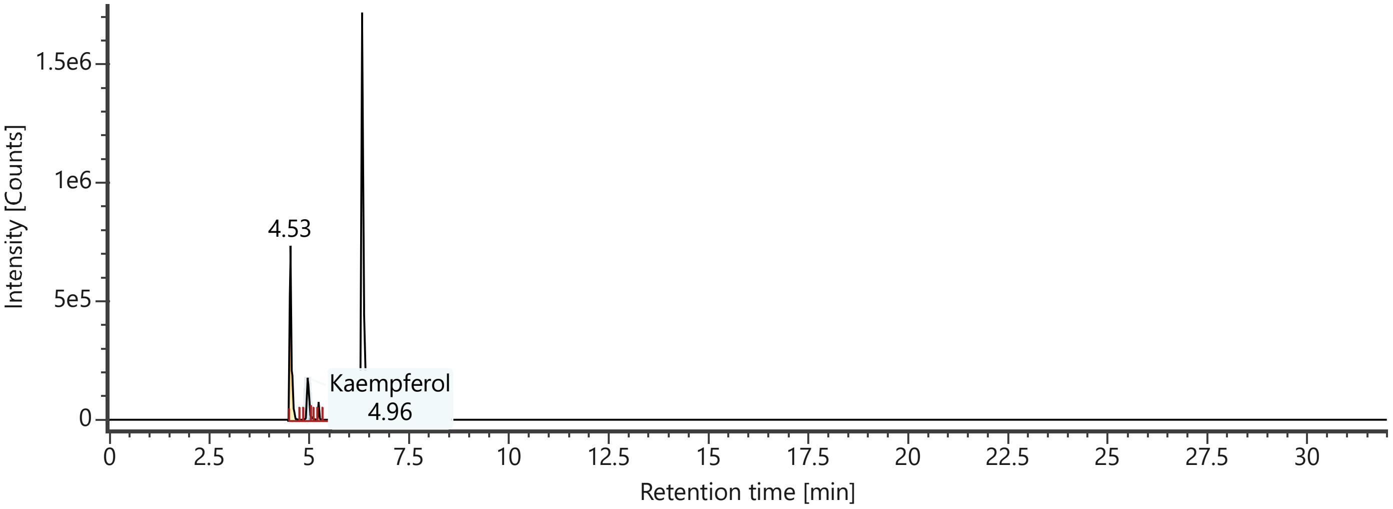

Item name: Sep257+ve
Item description: Mervat253
Channel name: Low energy : Time 4.9638 +/- 0.0237 minutes

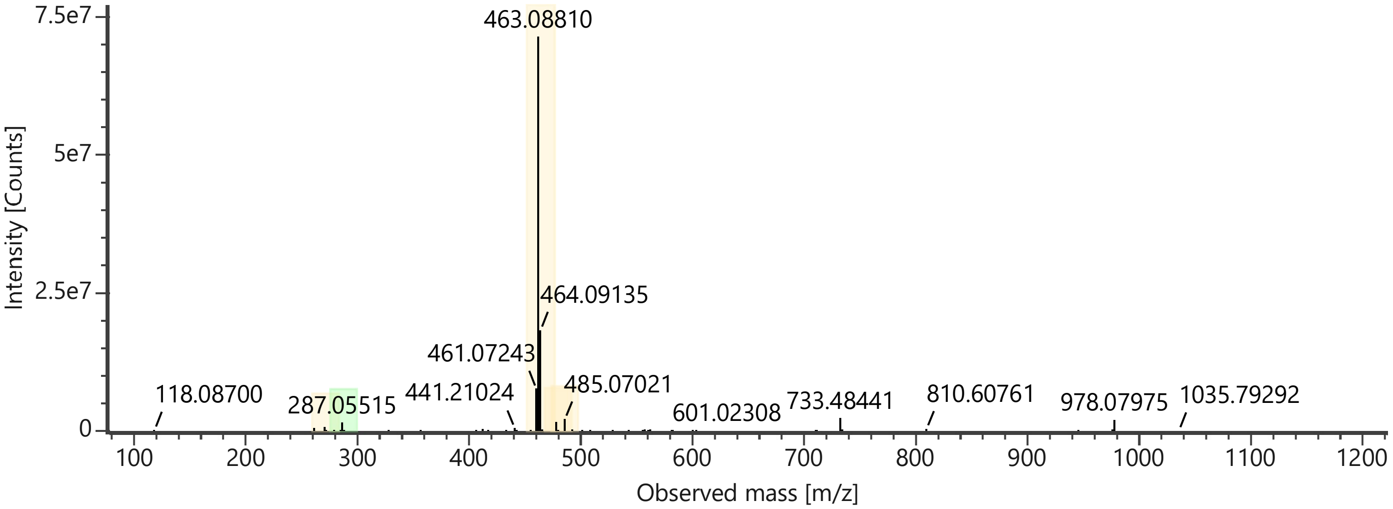

Item name: Sep257+ve
Item description: Mervat253

Channel name: High energy : Time 4.9638 +/- 0.0237 minutes

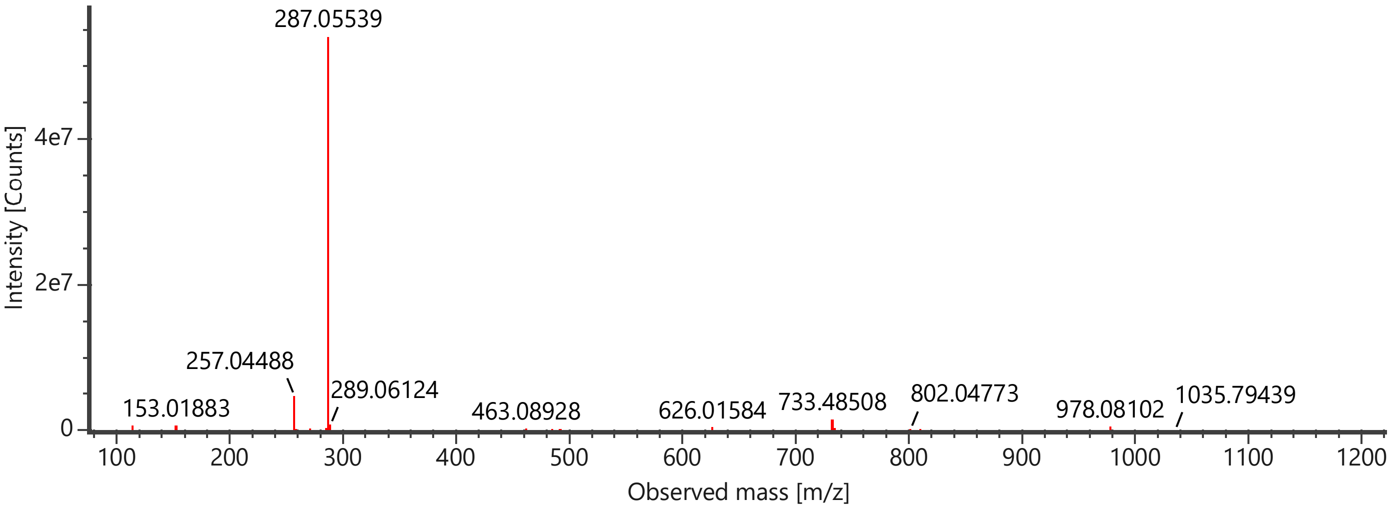

Component name: Kaempferol-3-glucuronide

Item name: Sep257+ve
Channel name: Kaempferol-3-glucuronide [+H] : (48.1 PPM) 463.0881

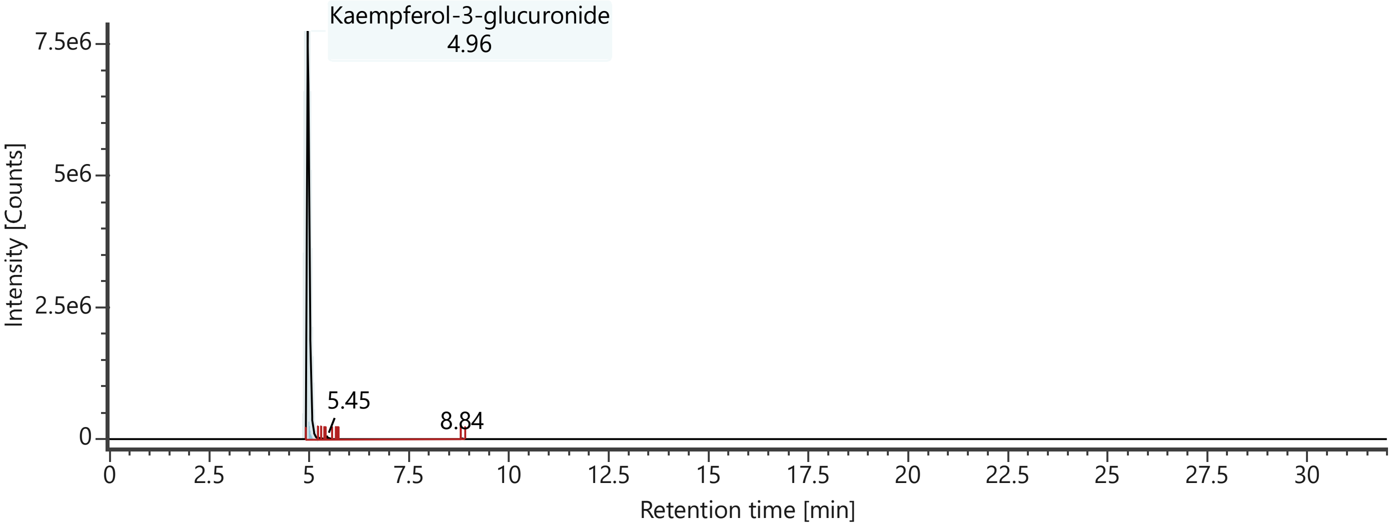

Item name: Sep257+ve
Item description: Mervat253
Channel name: Low energy : Time 4.9653 +/- 0.0237 minutes

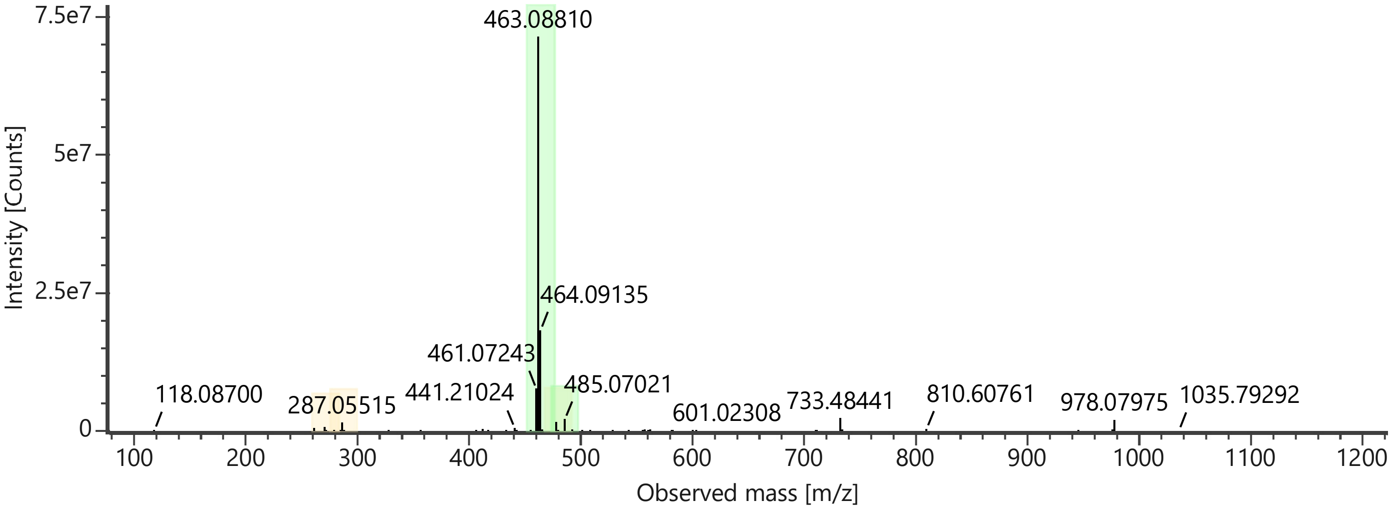

Item name: Sep257+ve
Item description: Mervat253

Channel name: High energy : Time 4.9653 +/- 0.0237 minutes

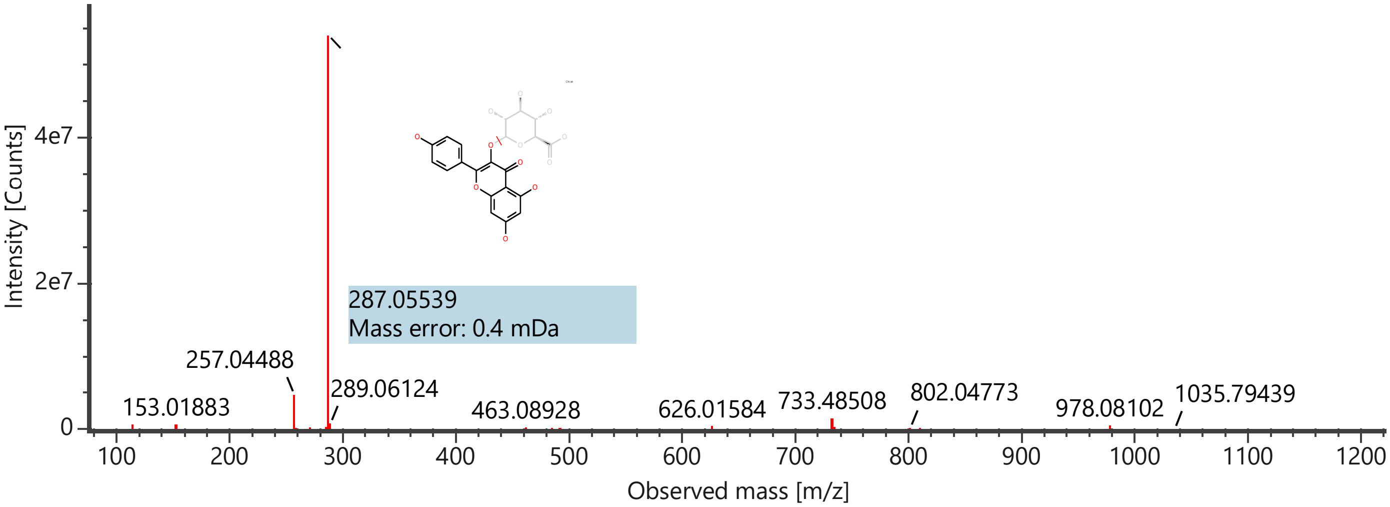

Component name: Quercituronе

Item name: Sep257+ve
Channel name: Quercituronе [+H] : (48.1 PPM) 479.0831

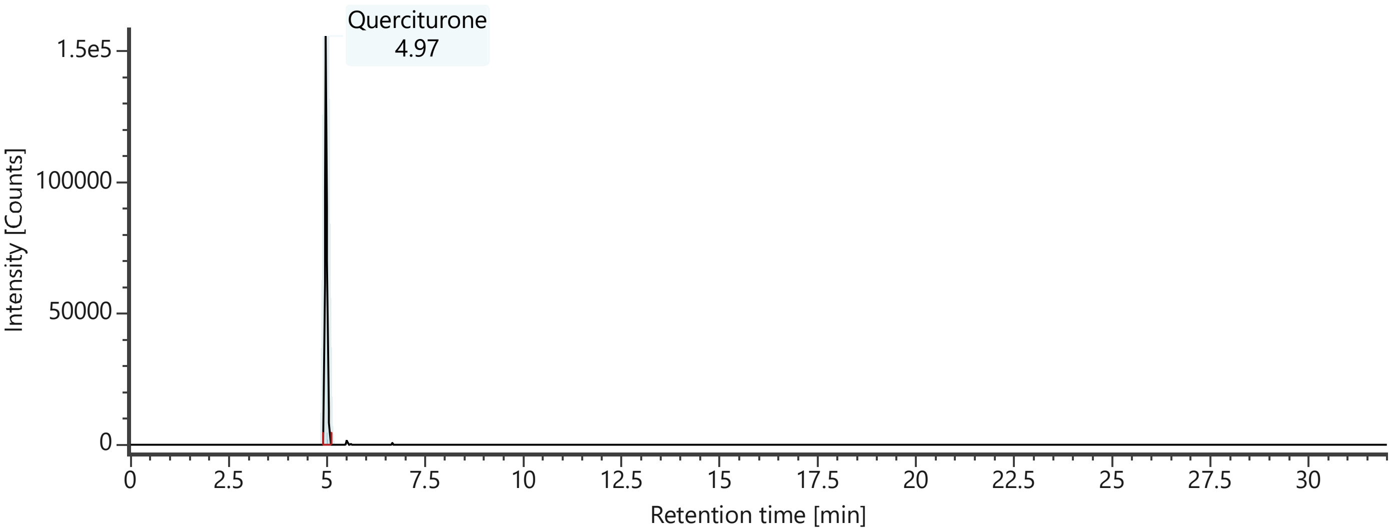

Item name: Sep257+ve
Item description: Mervat253

Channel name: Low energy : Time 4.9723 +/- 0.0237 minutes

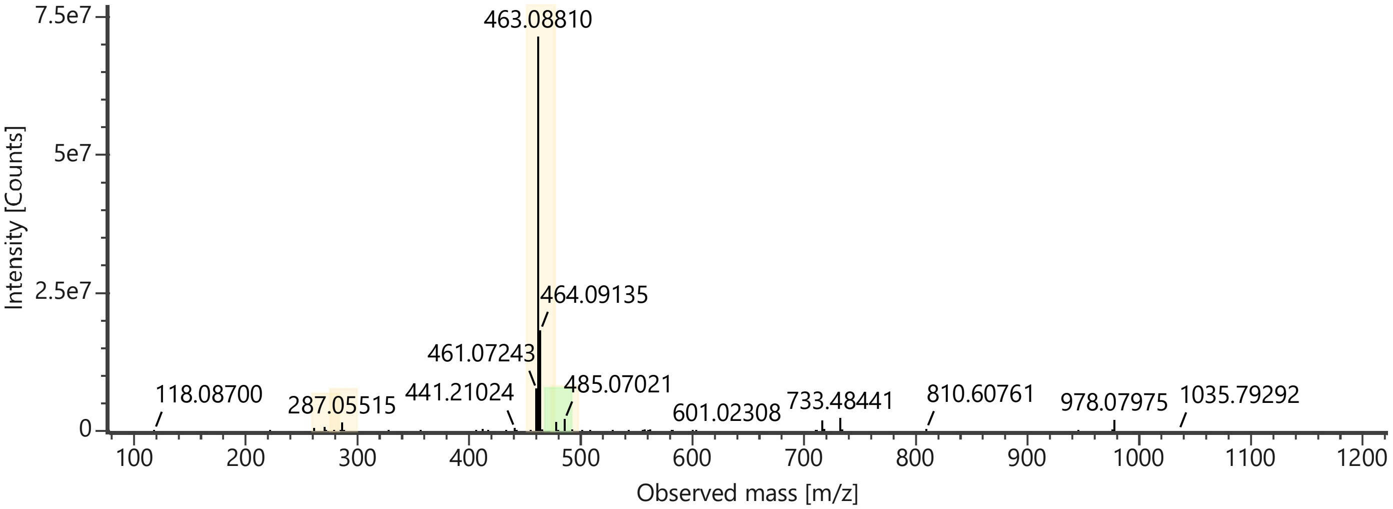

Item name: Sep257+ve
Item description: Mervat253

Channel name: High energy : Time 4.9723 +/- 0.0237 minutes

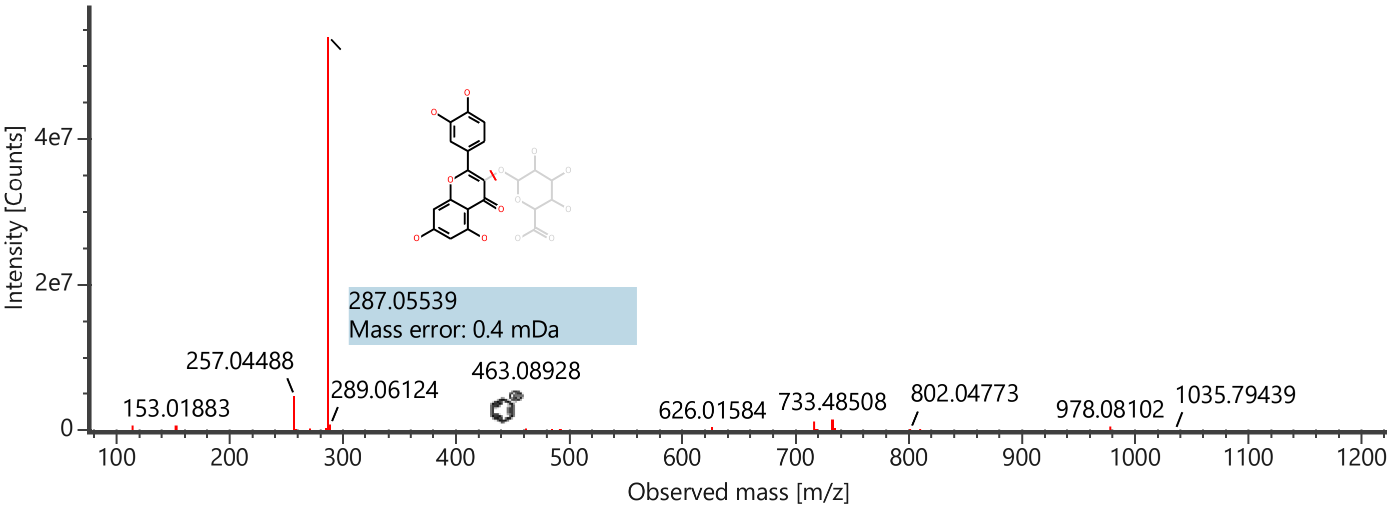

Component name: 1,3-Bis-[2-(3,4-dihydroxyphenyl)-1-carboxy]ethoxycarbonyl-2-(3,4-dihydroxyphenyl)-7,8-dihydroxy-1,2-dihydronaphthalene

Item name: Sep257+ve
Channel name: 1,3-Bis-[2-(3,4-dihydroxyphenyl)-1-carboxy]ethoxycarbonyl-2-(3,4-dihydroxyphenyl)-7,8-dihydroxy-1,2-dihydronaphthalene [+Na] : (48.1 PPM) 741.1431

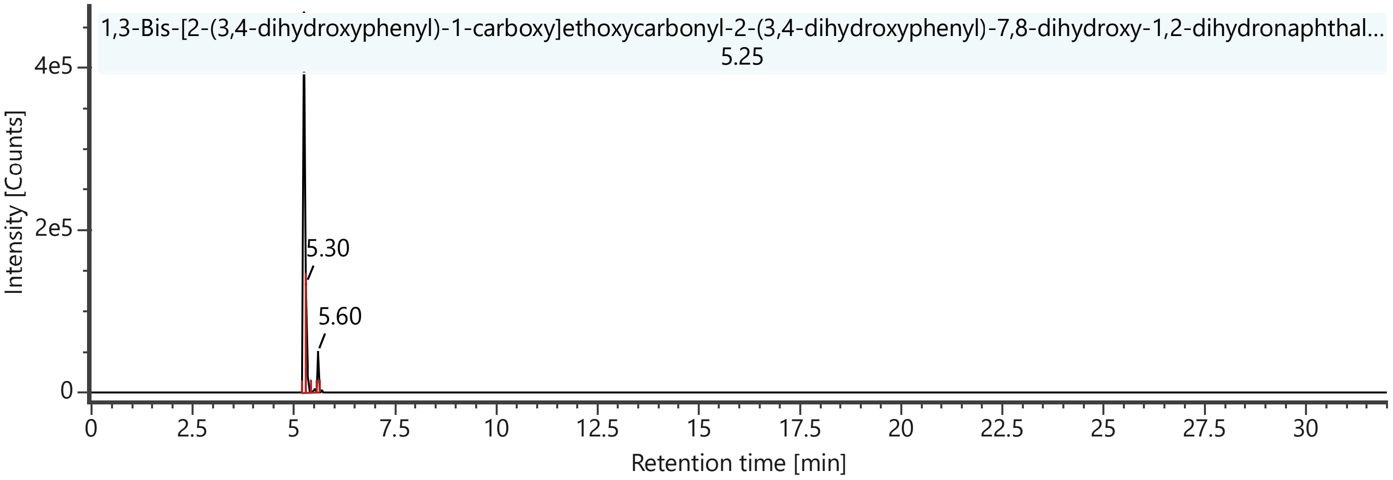

Item name: Sep257+ve
Item description: Mervat253
Channel name: Low energy : Time 5.2517 +/- 0.0237 minutes

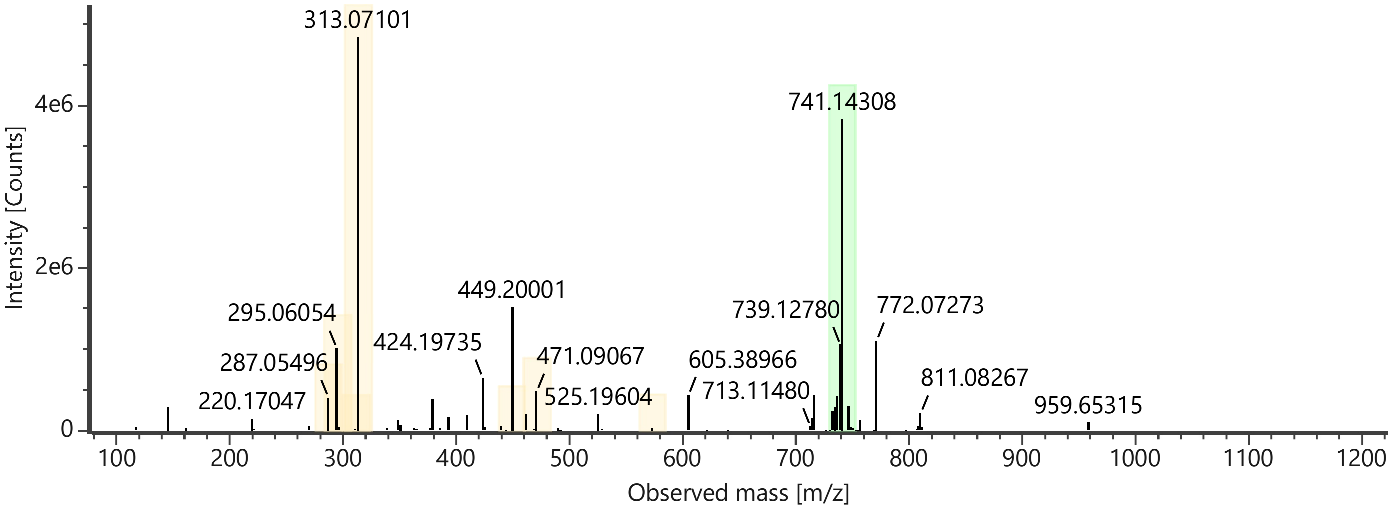

Item name: Sep257+ve
Item description: Mervat253

Channel name: High energy : Time 5.2517 +/- 0.0237 minutes

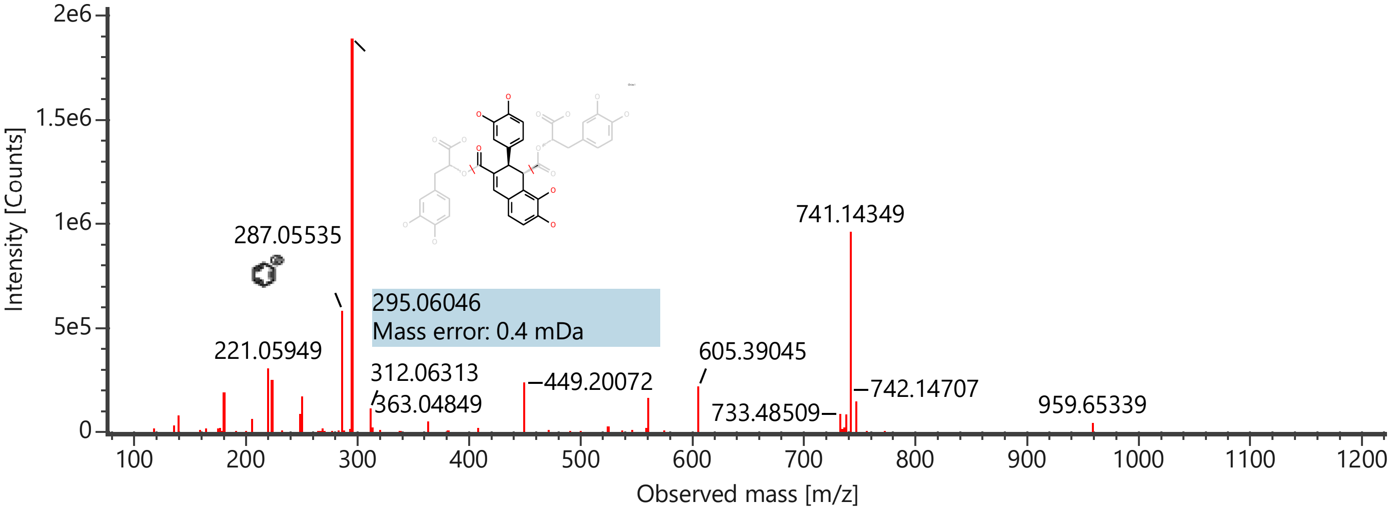

Component name: Kaempferol-O3-alpha-rhamnopyranoside

Item name: Sep257+ve
Channel name: Kaempferol-O3-alpha-rhamnopyranoside [+H] : (48.1 PPM) 433.1138

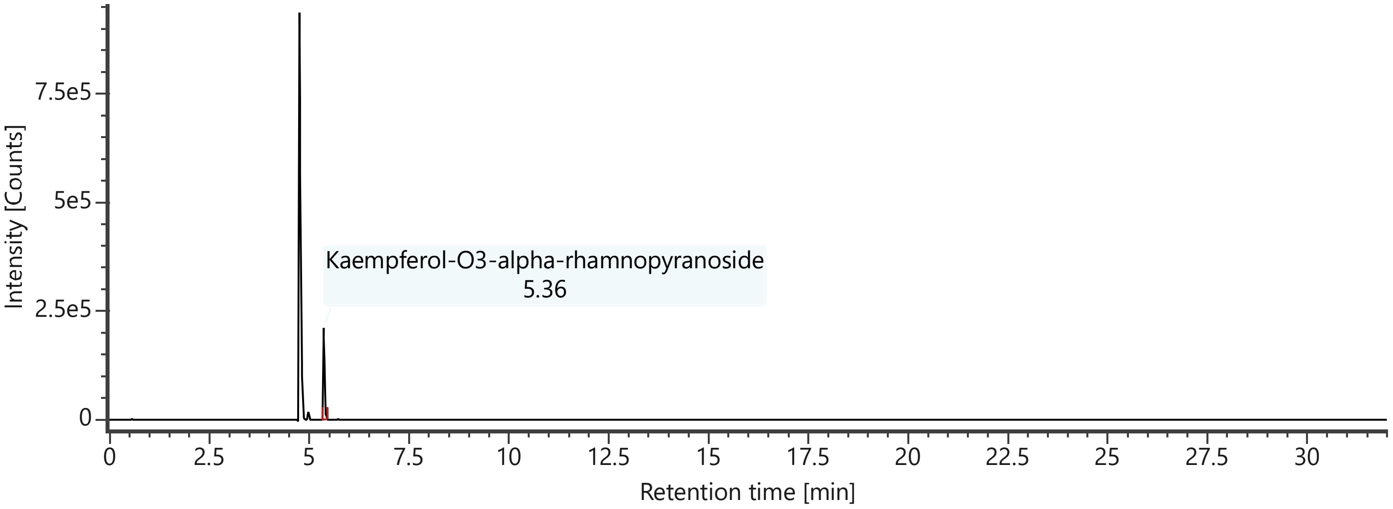

Item name: Sep257+ve
Item description: Mervat253
Channel name: Low energy : Time 5.3636 +/- 0.0237 minutes

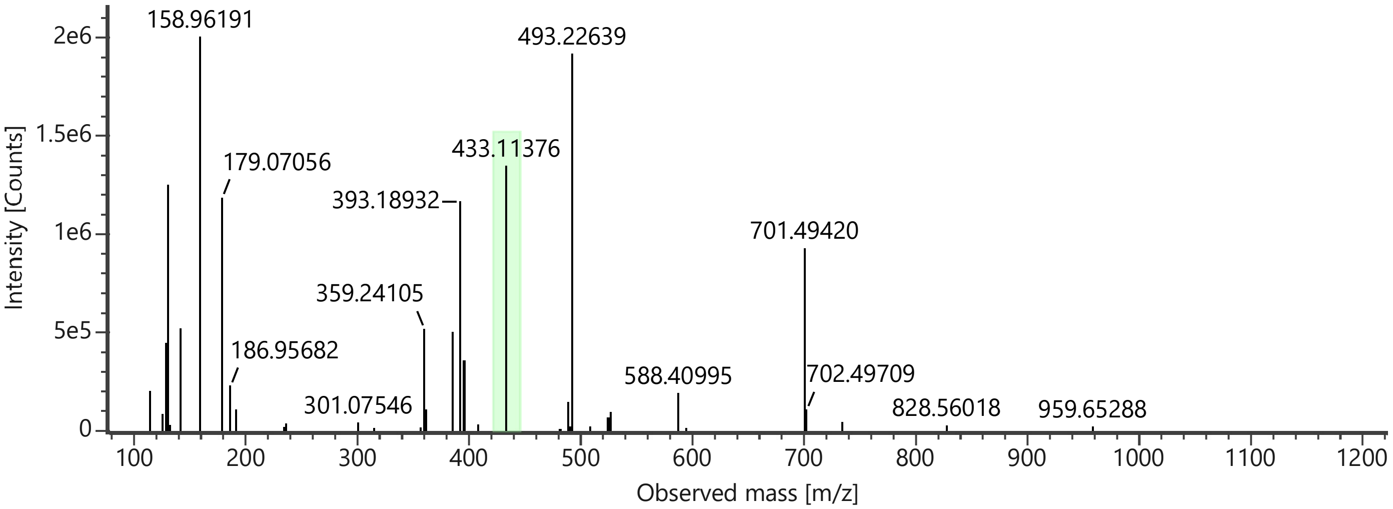

Item name: Sep257+ve
Item description: Mervat253

Channel name: High energy : Time 5.3636 +/- 0.0237 minutes

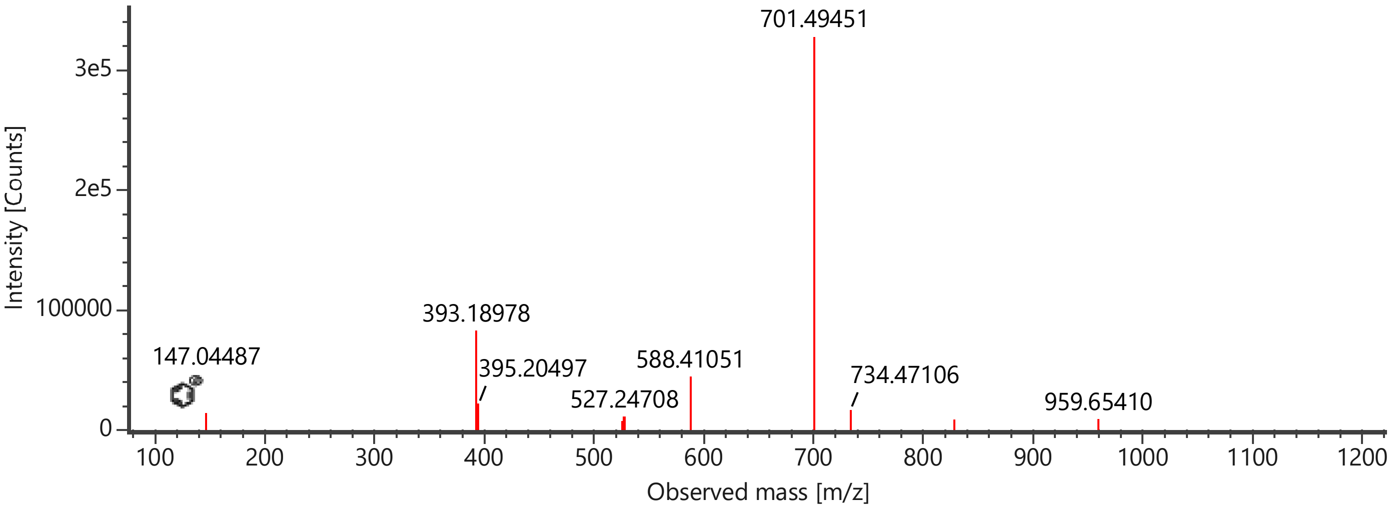

Component name: Apigenin

Item name: Sep257+ve
Channel name: Apigenin [+H] : (48.1 PPM) 271.0603

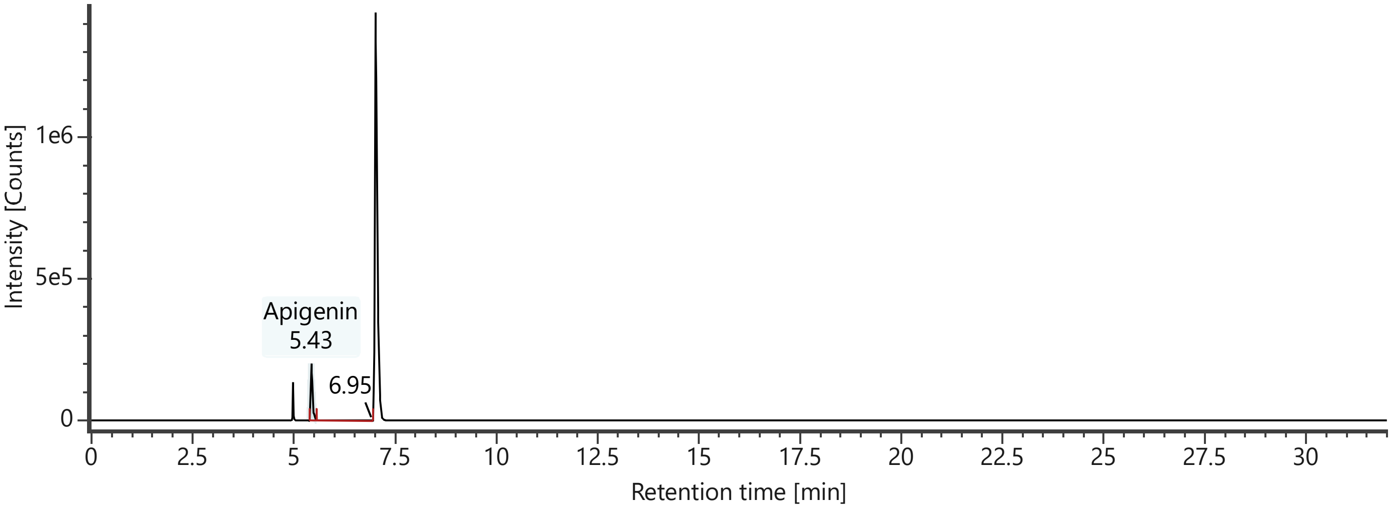

Item name: Sep257+ve
Item description: Mervat253
Channel name: Low energy : Time 5.4337 +/- 0.0237 minutes

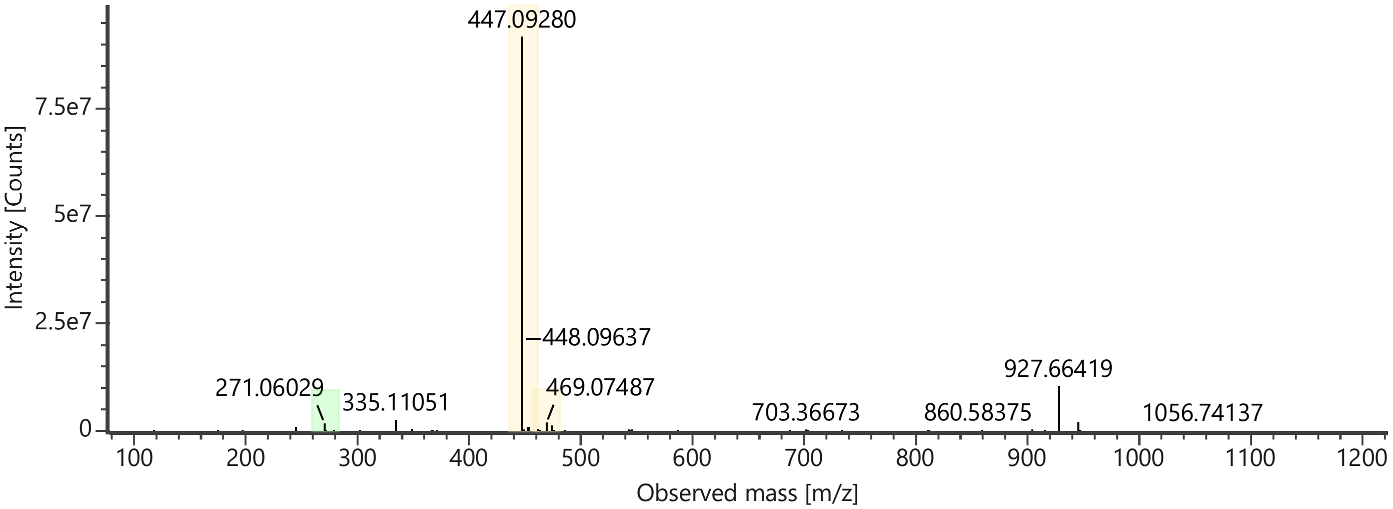

Item name: Sep257+ve
Item description: Mervat253

Channel name: High energy : Time 5.4337 +/- 0.0237 minutes

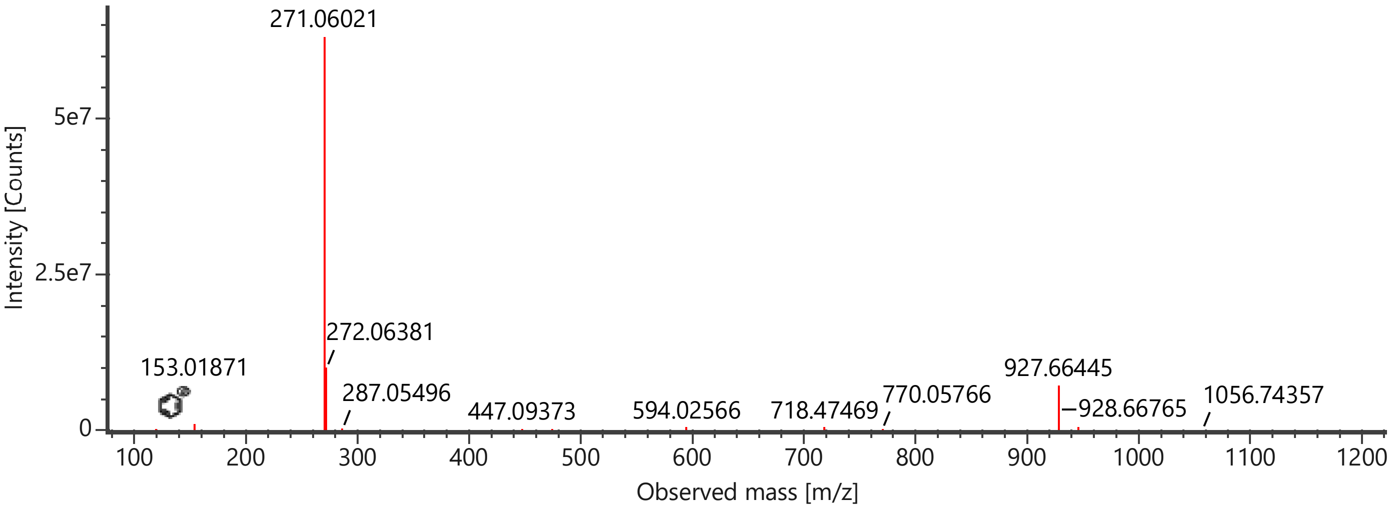

Component name: Apigenin-4'-O-glucuronide

Item name: Sep257+ve
Channel name: Apigenin-4'-O-glucuronide [+H] : (48.1 PPM) 447.0928

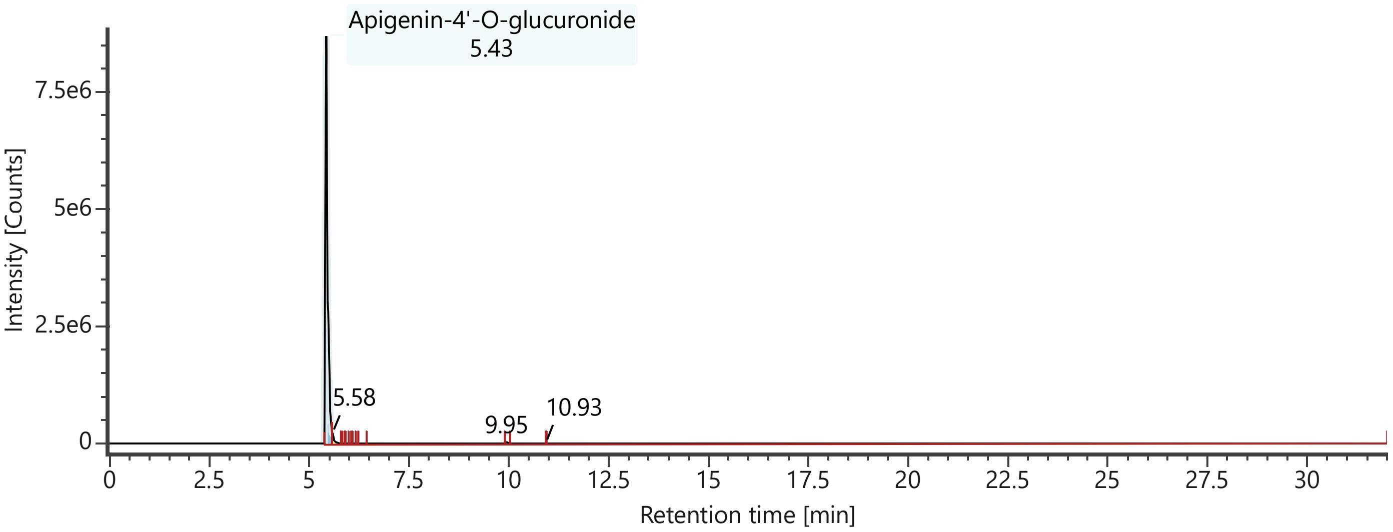

Item name: Sep257+ve
Item description: Mervat253
Channel name: Low energy : Time 5.4341 +/- 0.0711 minutes

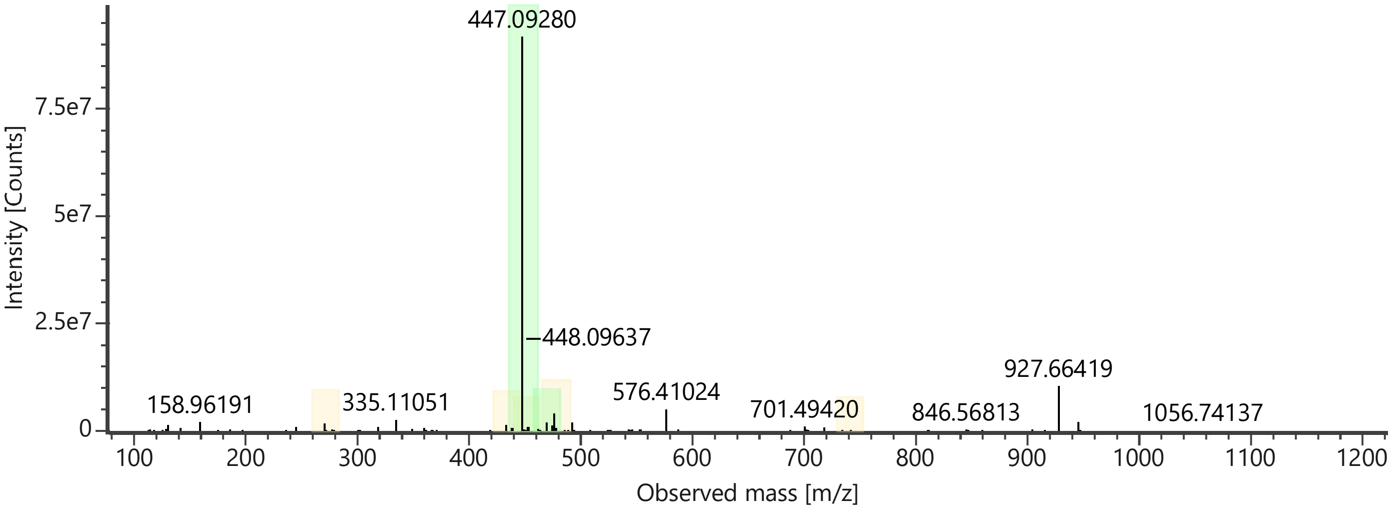

Item name: Sep257+ve
Item description: Mervat253

Channel name: High energy : Time 5.4341 + - 0.0711 minutes

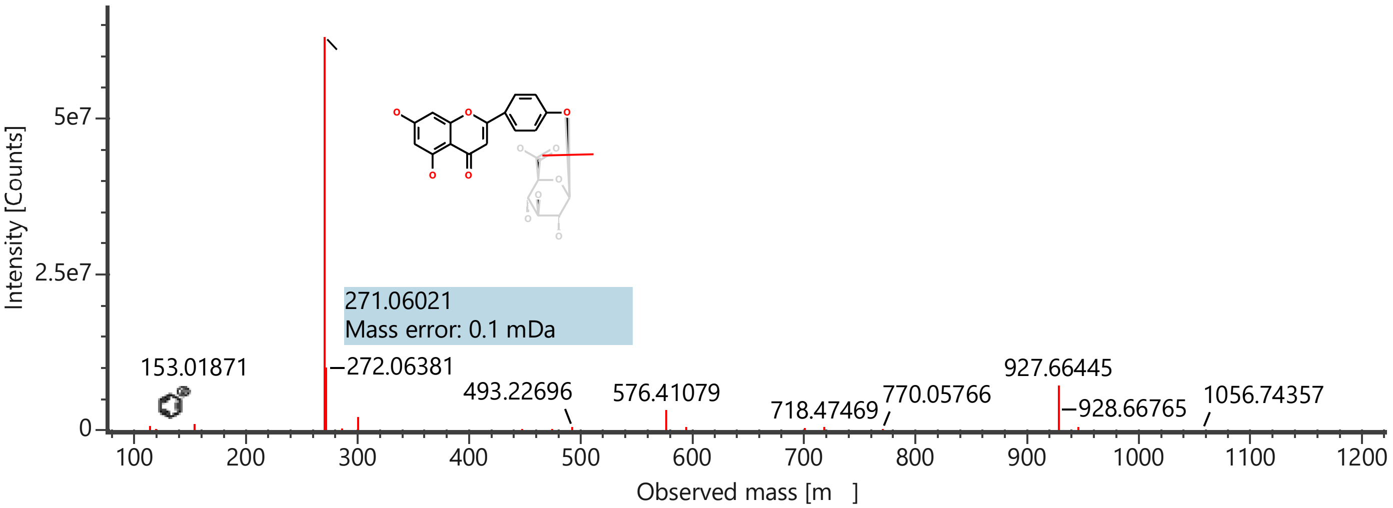

Created by: Ayad ounan

Created on: ep

tem name: amiaceae Family ve mode

Created time: : : gypt tandard Time

o po e a e eo e e er g ro de

tem name: ep ve
C annel name: uteolin- -O-(6 -met yl ester)- - -glucuronide : (. M) .

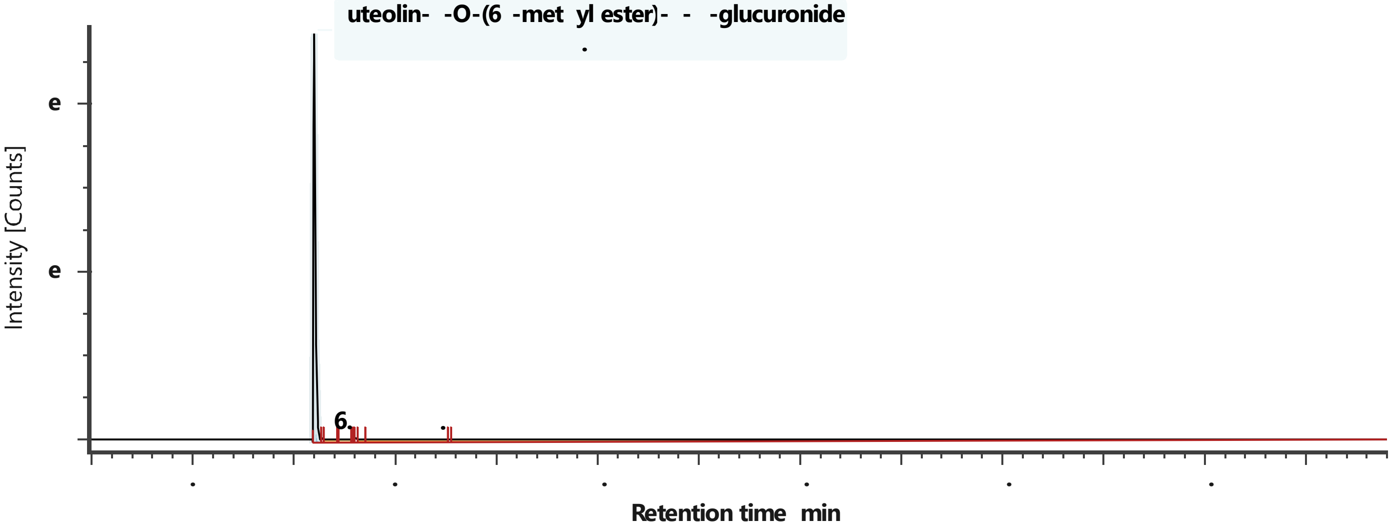

tem name: ep ve
tem description: Mervat

C annel name: o energy : Time . /- . minutes

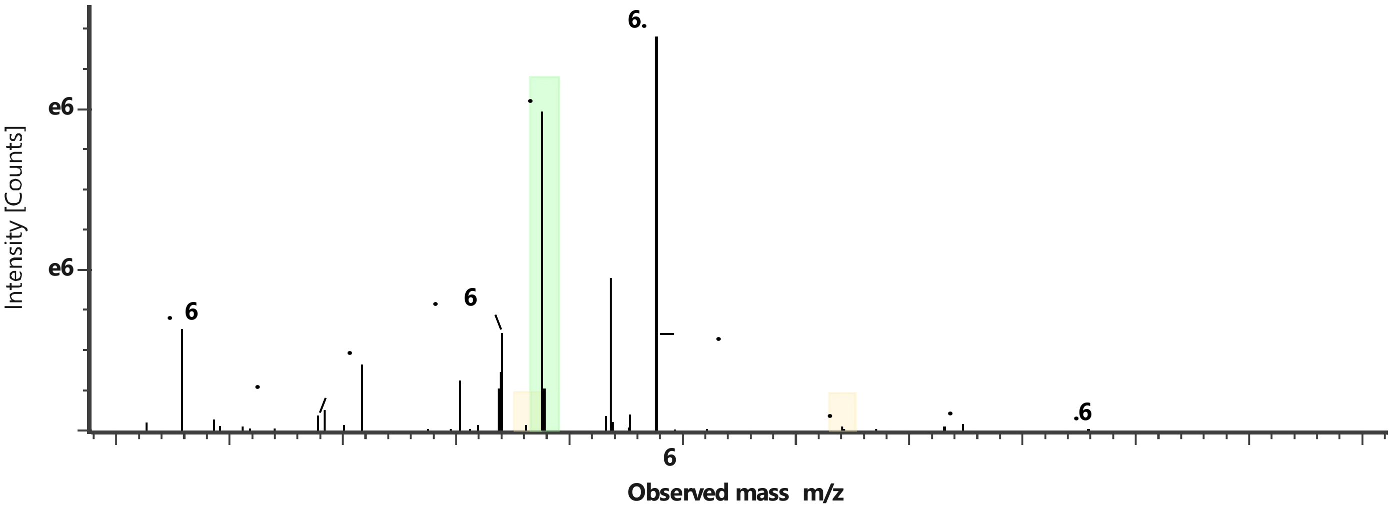

I e a e a a eae Fa e ode rea ed e 1 52 1 g p a dard e

I e a e ep25 e a e a e g e erg e 5 5 5 2 e
I e de r p o Mer a 25

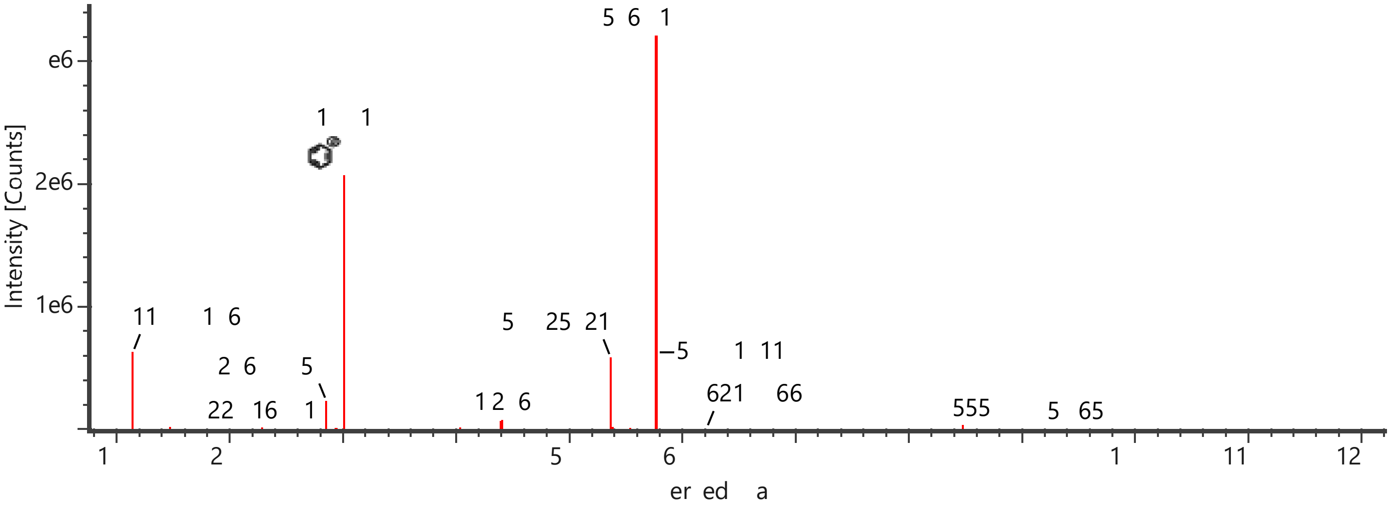

Component name: Rosmarinic acid

tem name: ep 5 ve

C annel name: Rosmarinic acid a : (. M) .

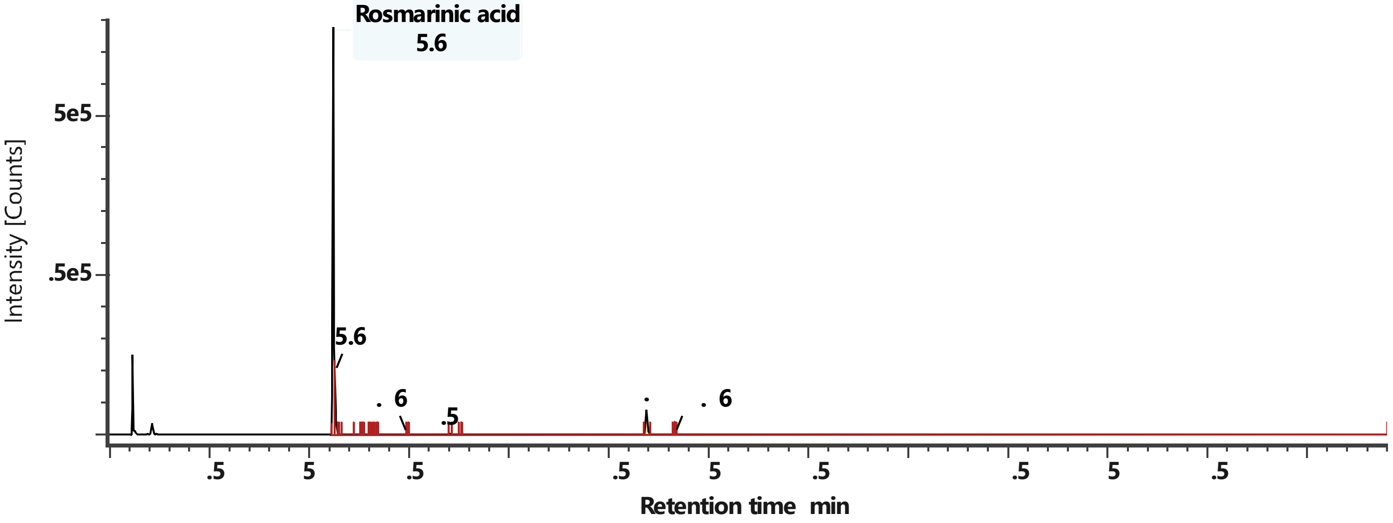

tem name: ep 5 ve

tem description: Mervat 5

C annel name: o energy : Time 5.5 /- . minutes

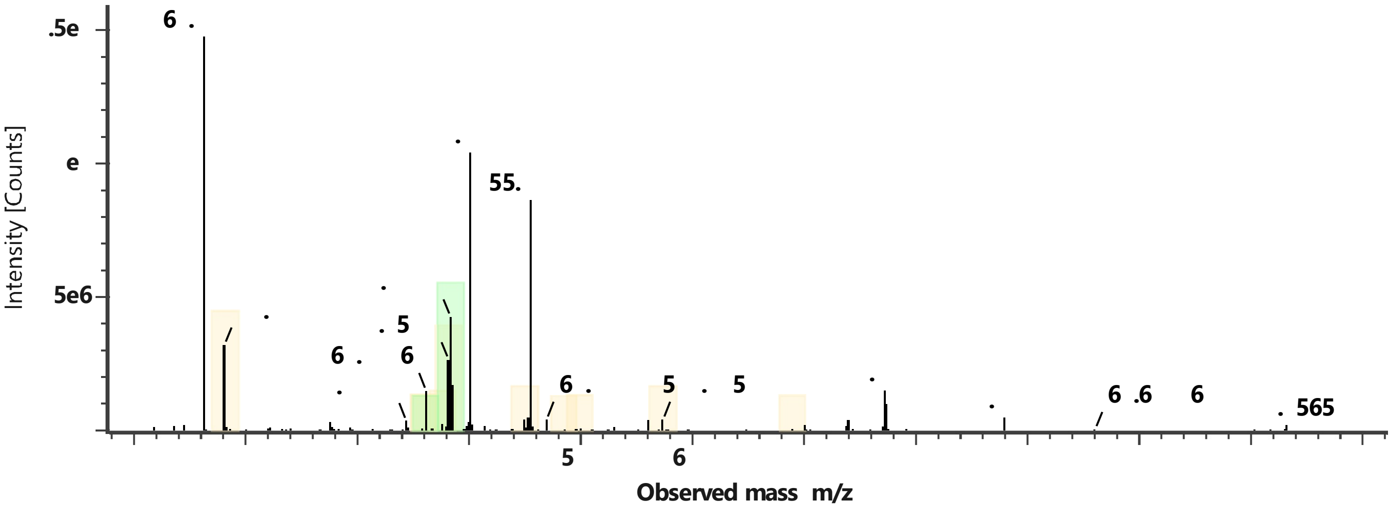

Created by: Ayad, Younan

Created on: Sep 18, 2025

Item name: Lamiaceae Family +ve mode

Created time: 13:52:10 Egypt Standard Time

Item name: Sep257+ve
Item description: Mervat253

Channel name: High energy : Time 5.5982 +/- 0.0237 minutes

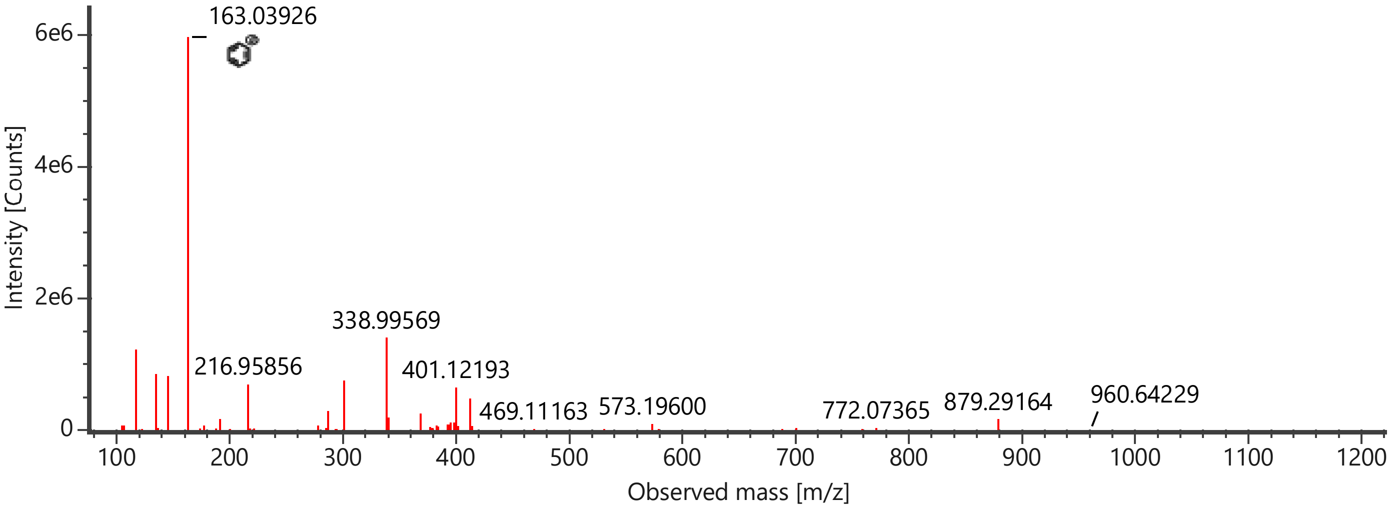

Component name: trans Caffeic acid

Item name: Sep257+ve
Channel name: trans Caffeic acid [+H] : (48.1 PPM) 181.0499

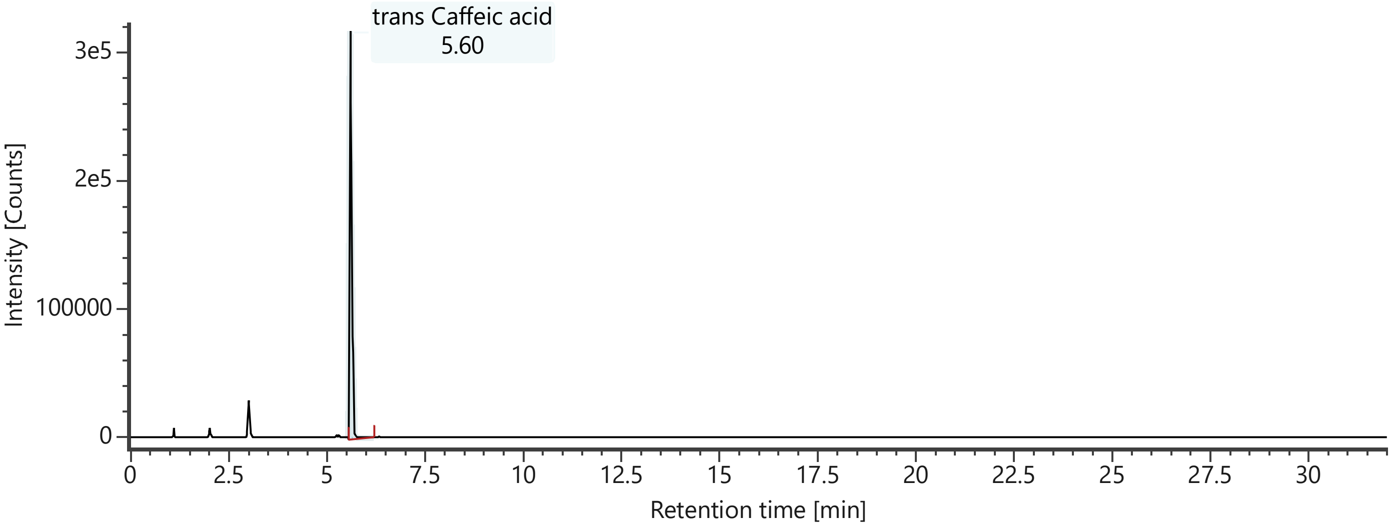

Item name: Sep257+ve
Item description: Mervat253

Channel name: Low energy : Time 5.6000 +/- 0.0237 minutes

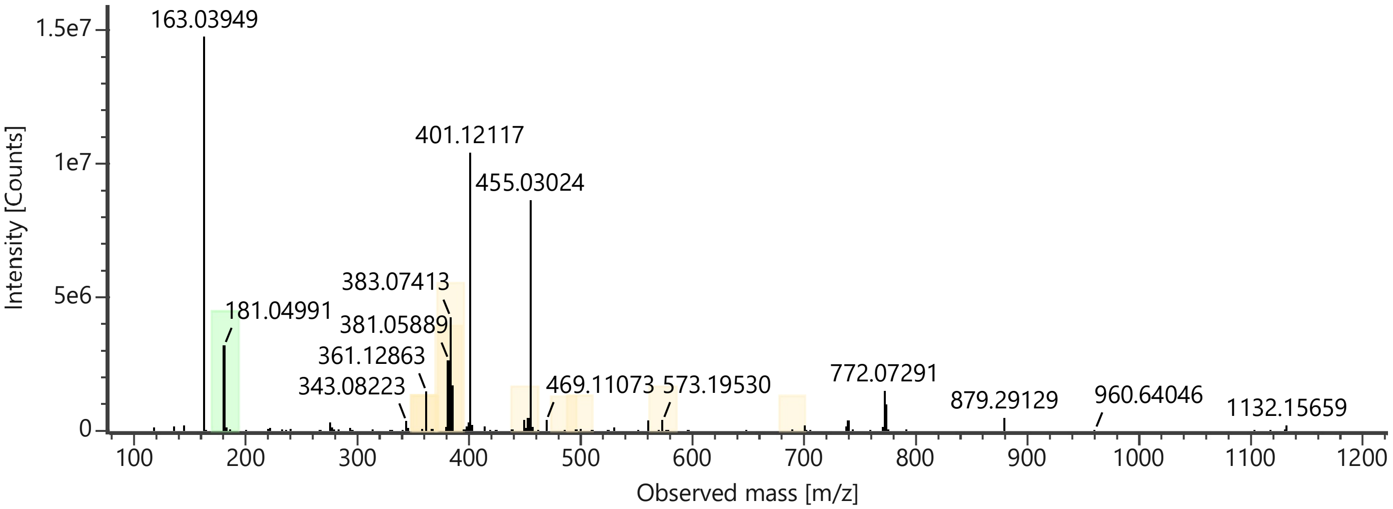

Item name: Sep257+ve
Item description: Mervat253

Channel name: High energy : Time 5.6000 +/- 0.0237 minutes

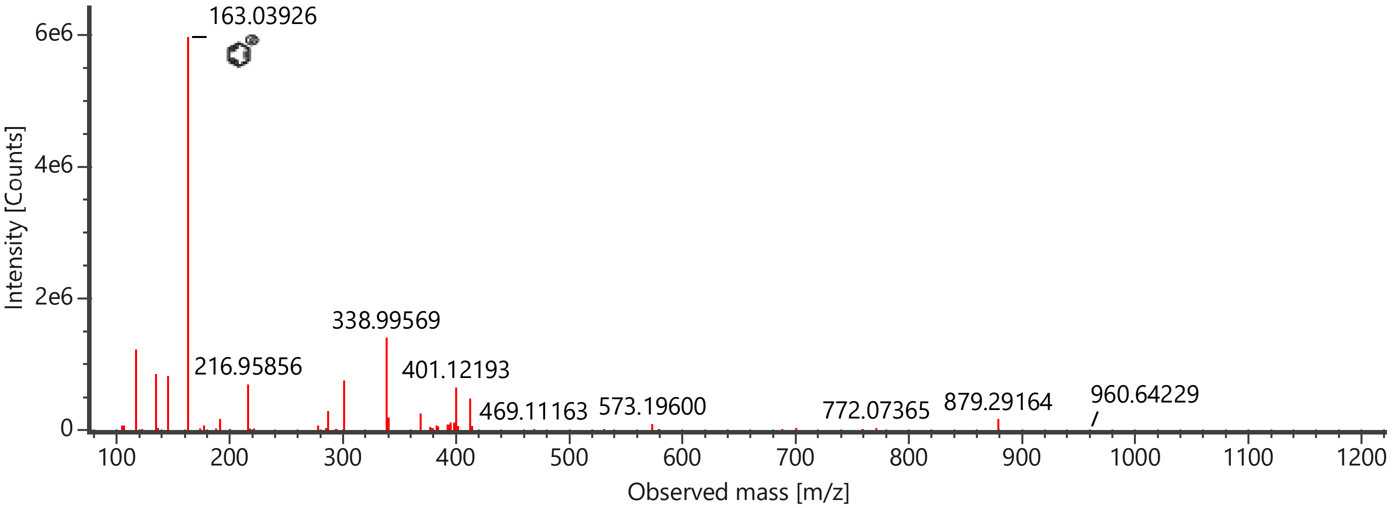

Component name: 7,8-Dihydroxy-2-(3,4-dihydroxyphenyl)-1,2-dihydronaphthalene-1,3-dicarboxylic acid

Item name: Sep257+ve
Channel name: 7,8-Dihydroxy-2-(3,4-dihydroxyphenyl)-1,2-dihydronaphthalene-1,3-dicarboxylic acid [+Na] : (48.1 PPM) 381.0589

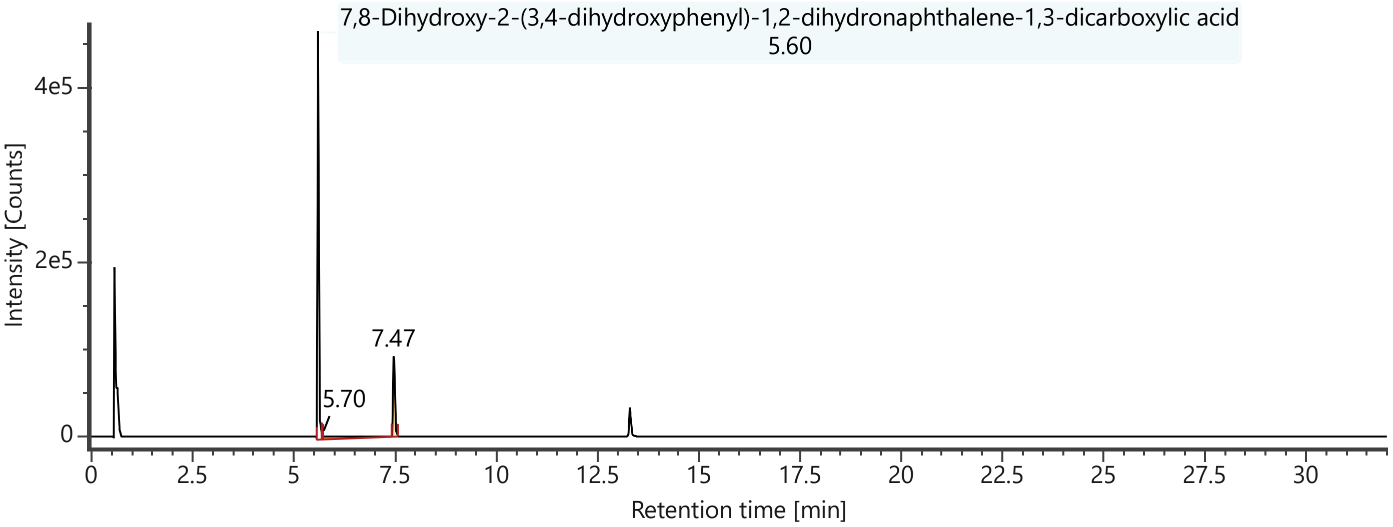

Item name: Sep257+ve
Item description: Mervat253
Channel name: Low energy : Time 5.6001 +/- 0.0237 minutes

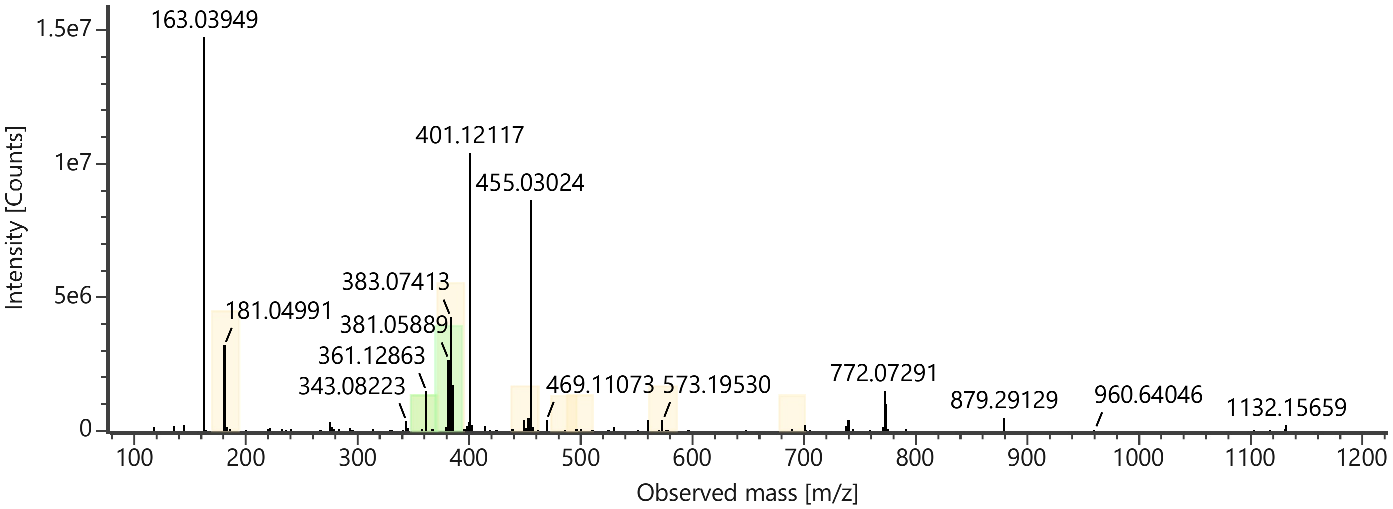

Created by: Ayad, Younan

Created on: Sep 18, 2025

Item name: Lamiaceae Family +ve mode

Created time: 13:52:10 Egypt Standard Time

Item name: Sep257+ve
Item description: Mervat253

Channel name: High energy : Time 5.6001 +/- 0.0237 minutes

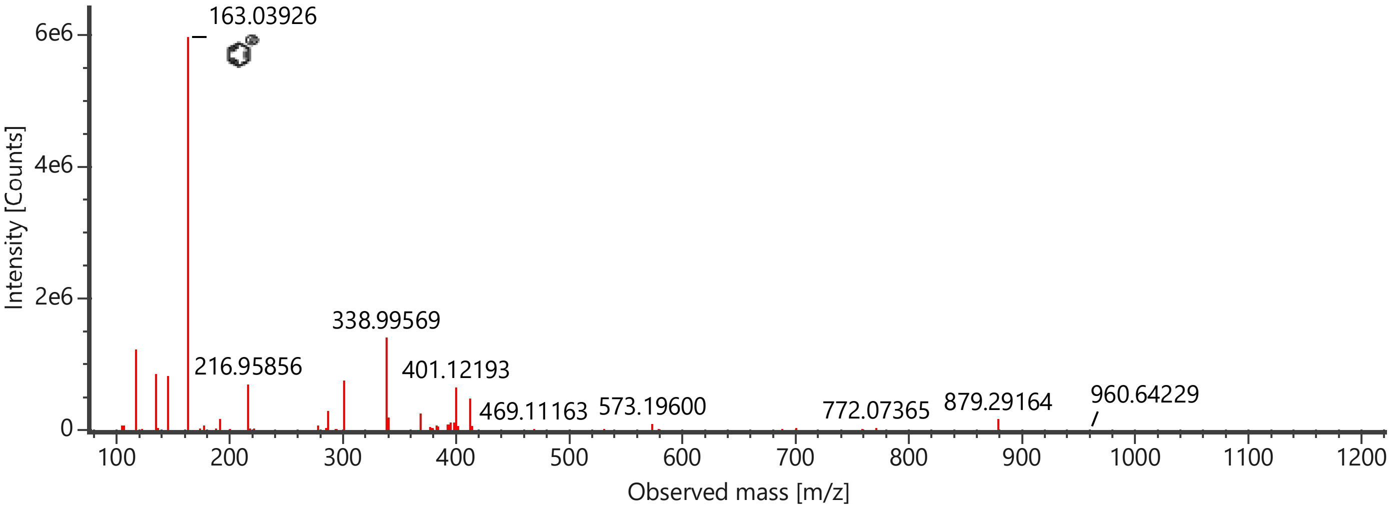

Created by: Ayad, Younan

Created on: Sep 18, 2025

Item name: Lamiaceae Family +ve mode

Created time: 13:52:10 Egypt Standard Time

Component name: Quercilicoside A

Item name: Sep257+ve
Channel name: Quercilicoside A [+Na] : (48.1 PPM) 689.3875

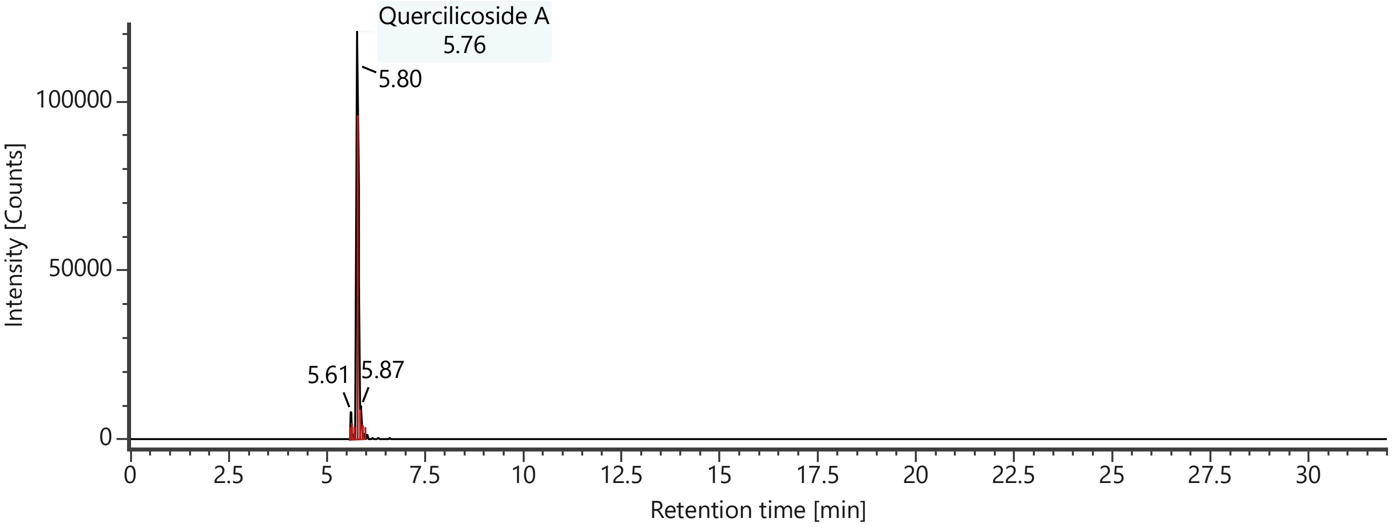

Item name: Sep257+ve
Item description: Mervat253

Channel name: Low energy : Time 5.7779 +/- 0.0237 minutes

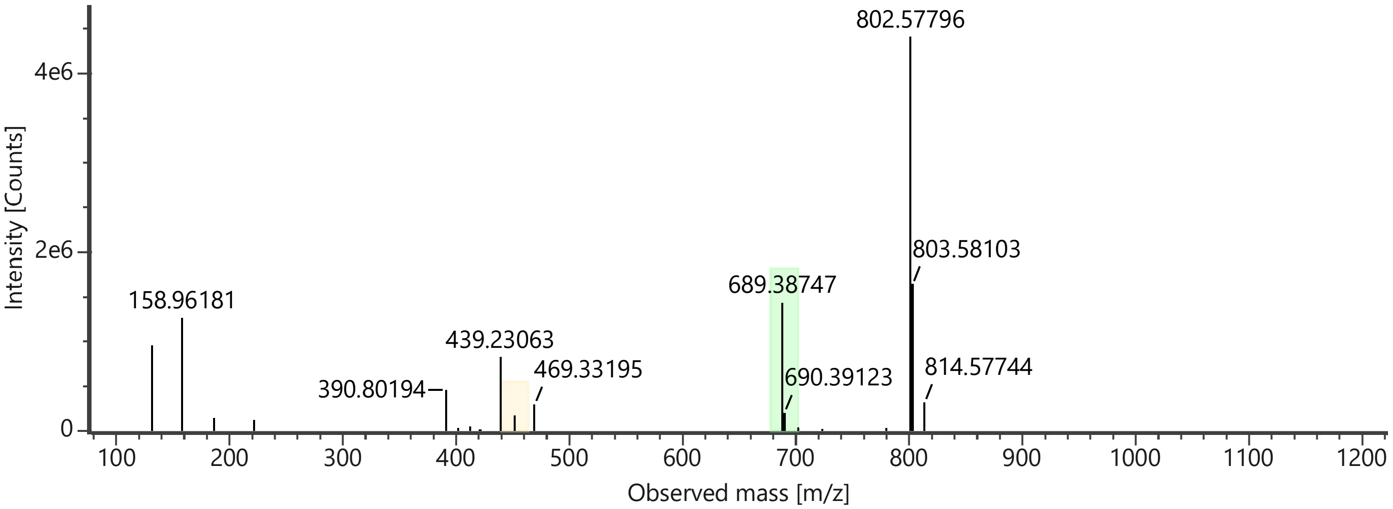

Item name: Sep257+ve
Item description: Mervat253

Channel name: High energy : Time 5.7779 +/- 0.0237 minutes

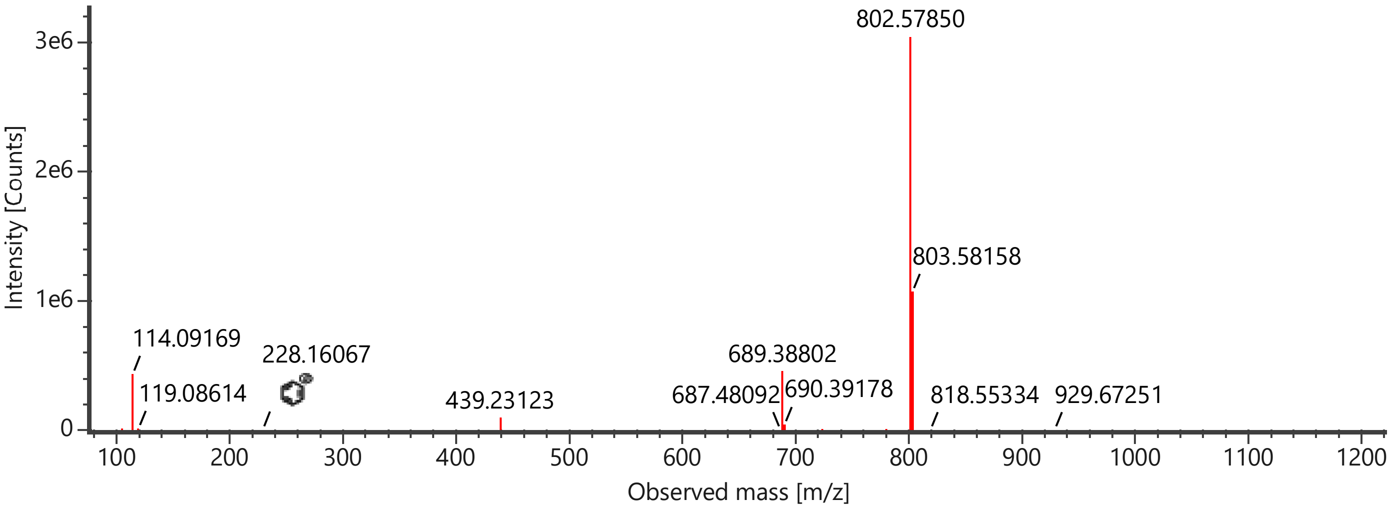

Component name: 2-O-Caffeoyl arbutin

Item name: Sep257+ve
Channel name: 2-O-Caffeoyl arbutin [+H] : (48.1 PPM) 435.1265

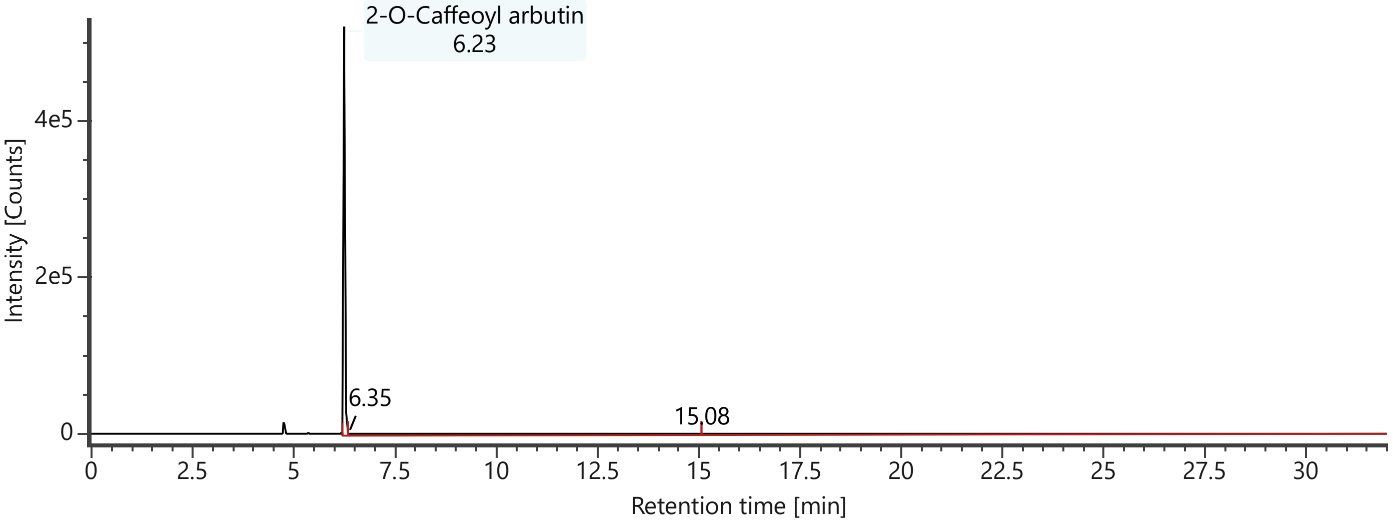

Item name: Sep257+ve
Item description: Mervat253
Channel name: Low energy : Time 6.2373 +/- 0.0237 minutes

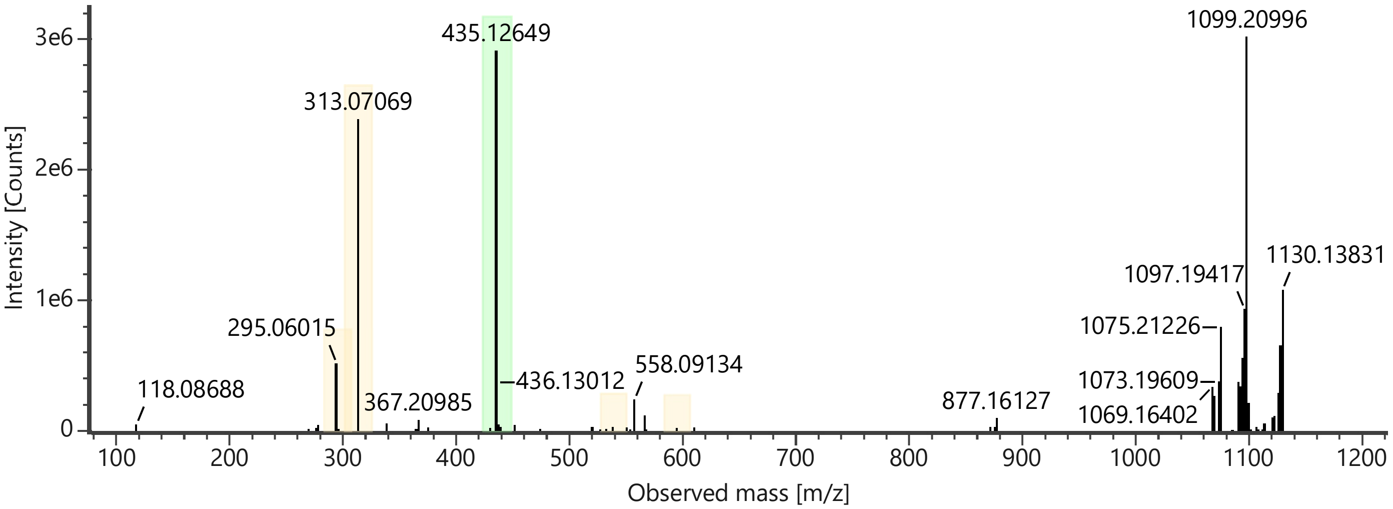

Item name: Sep257+ve
Item description: Mervat253

Channel name: High energy : Time 6.2373 +/- 0.0237 minutes

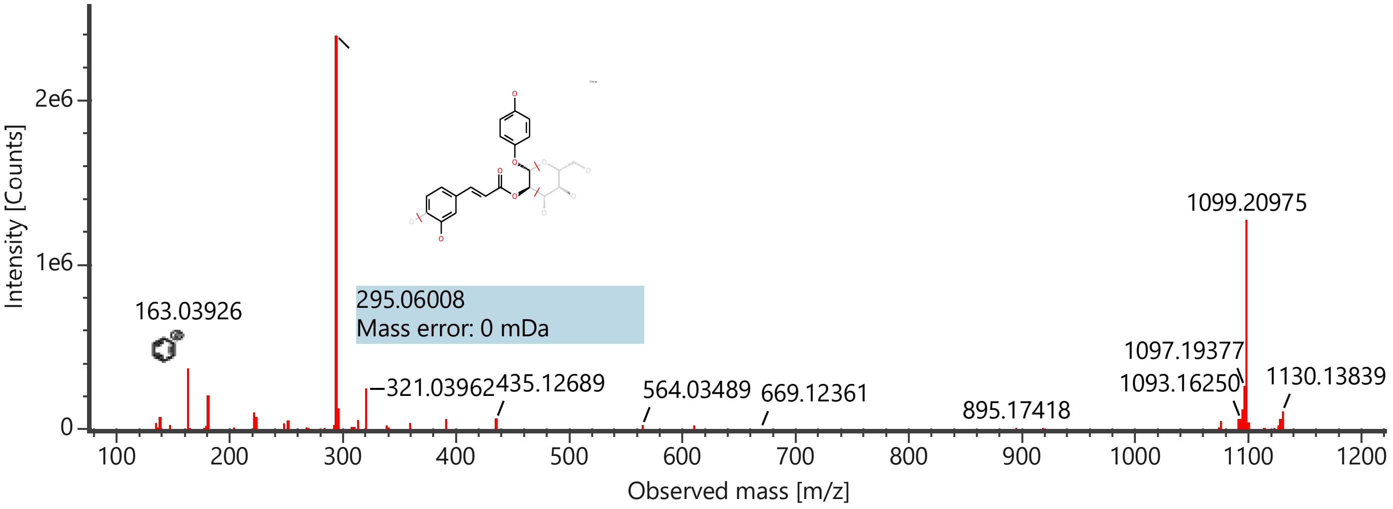

Component name: Luteolin

Item name: Sep257+ve
Channel name: Luteolin [+H] : (48.1 PPM) 287.0547

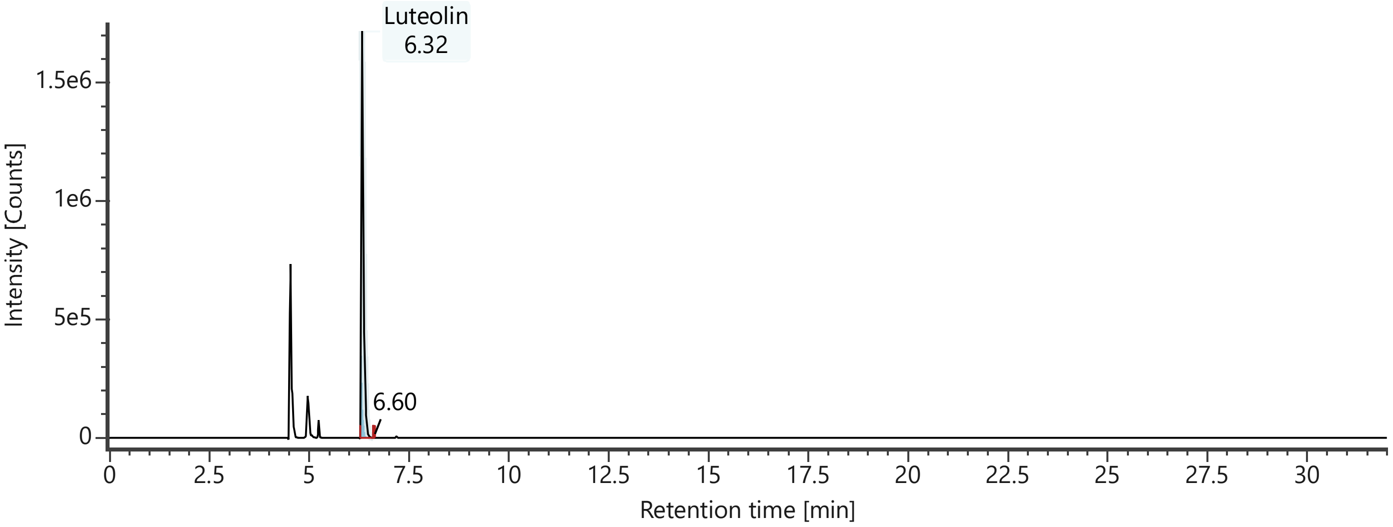

Item name: Sep257+ve
Item description: Mervat253

Channel name: Low energy : Time 6.3264 +/- 0.0237 minutes

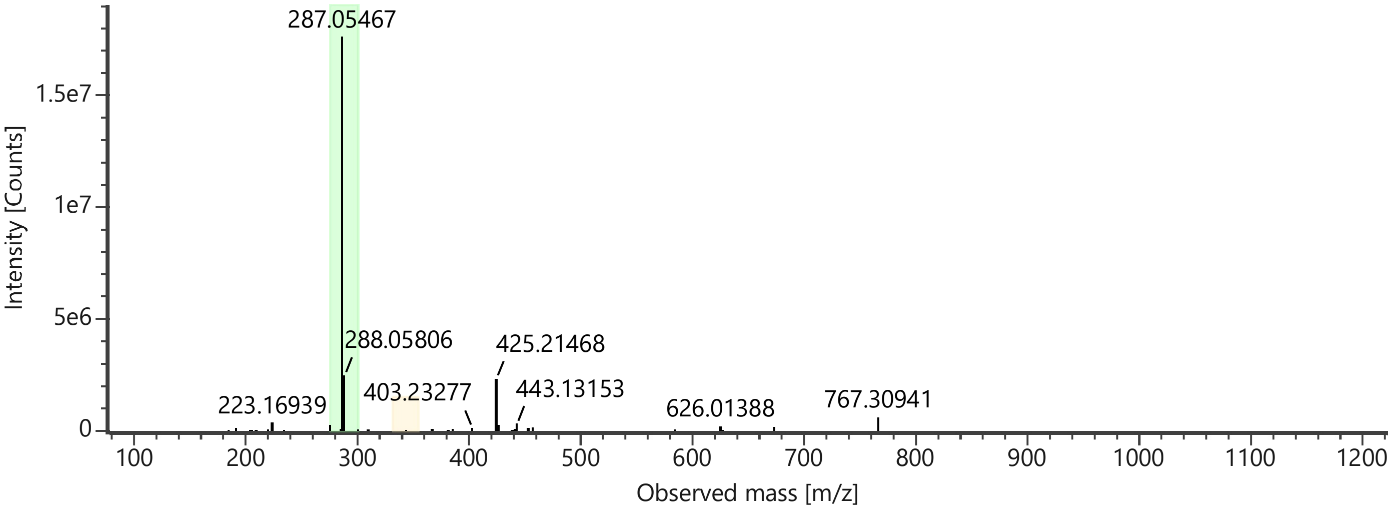

Item name: Lamiaceae Family +ve mode

Created by: Ayad, Younan

Created on: Sep 18, 2025

Created time: 13:52:10 Egypt Standard Time

Item name: Sep257+ve
Item description: Mervat253

Channel name: High energy : Time 6.3264 +/- 0.0237 minutes

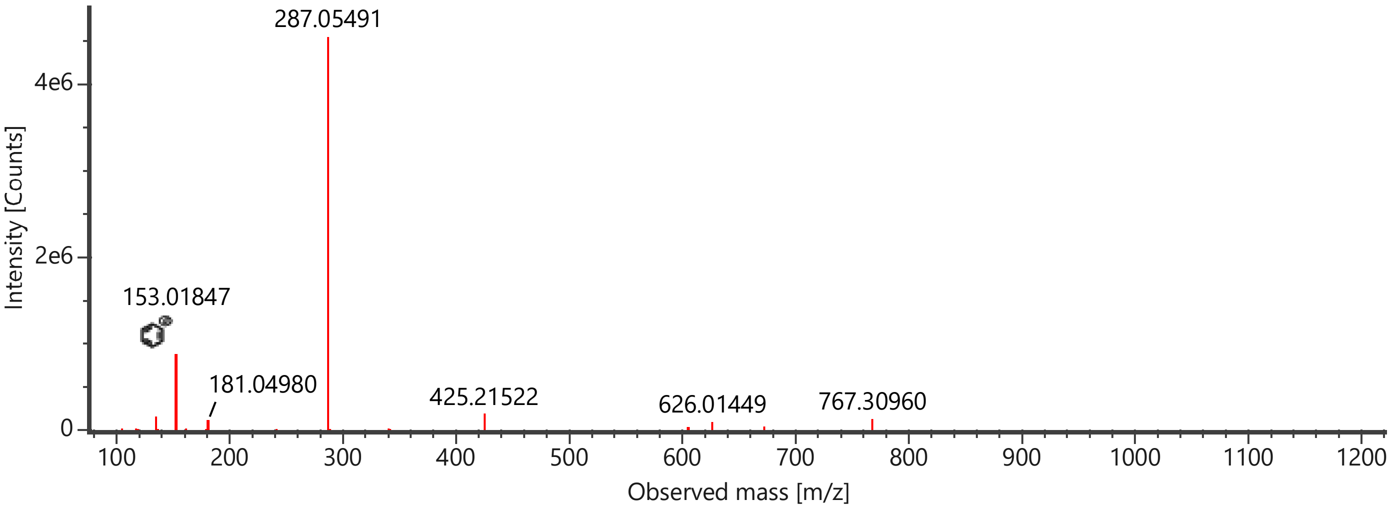

Component name: Quercetin 3'-methyl ether

Item name: Sep257+ve
Channel name: Quercetin 3'-methyl ether [+H] : (48.1 PPM) 317.0655

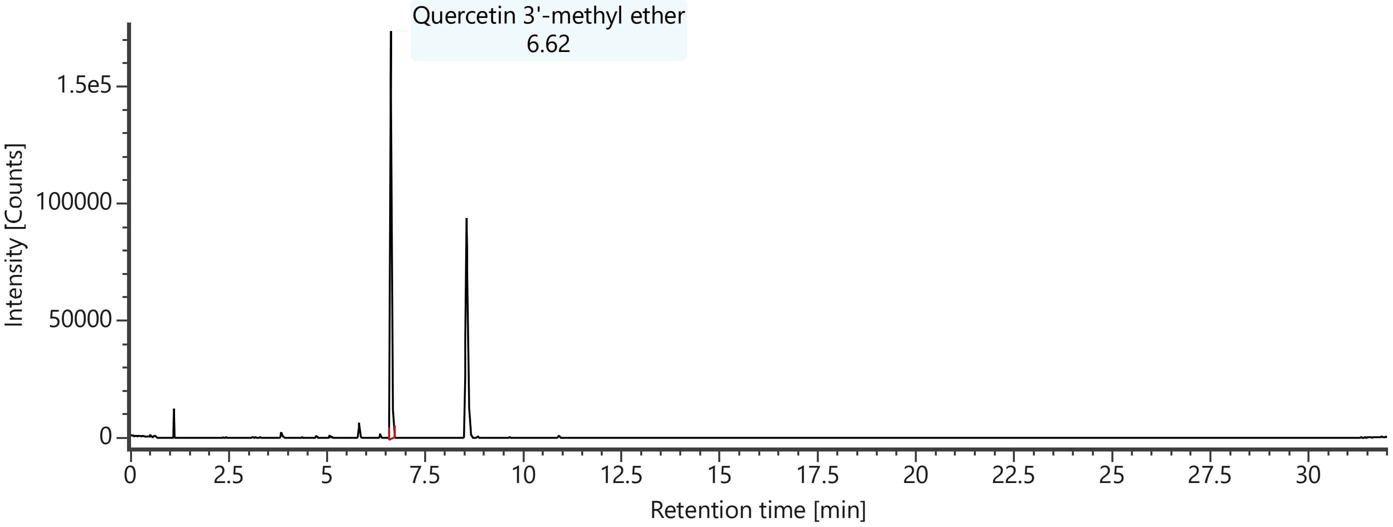

Item name: Sep257+ve
Item description: Mervat253
Channel name: Low energy : Time 6.6255 +/- 0.0237 minutes

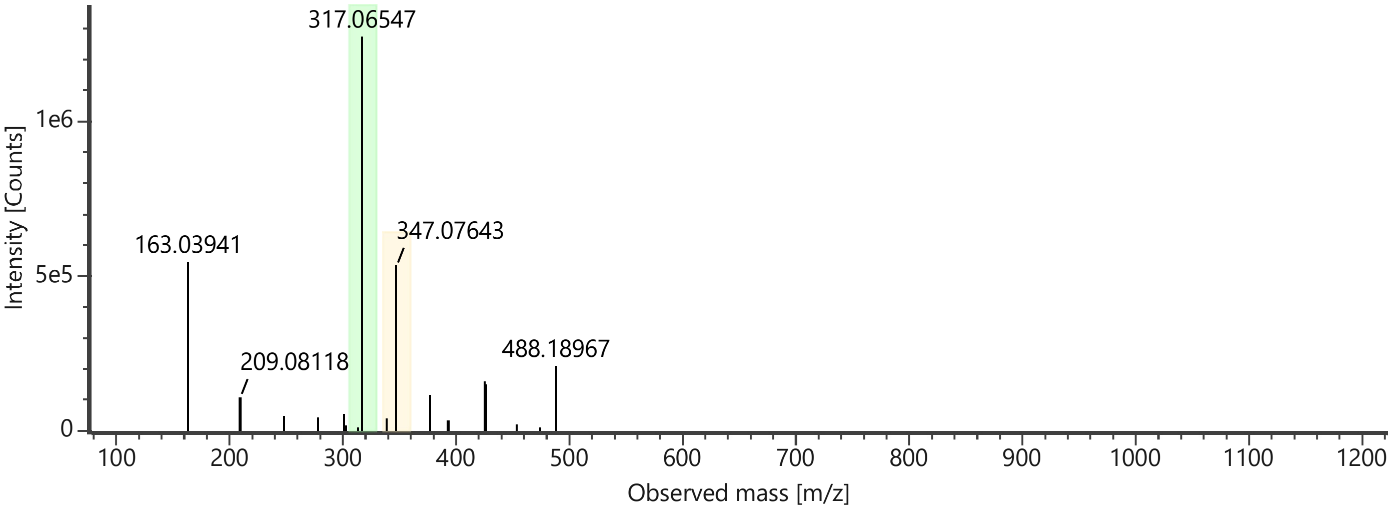

Created by: Ayad, Younan

Created on: Sep 18, 2025

Item name: Lamiaceae Family +ve mode

Created time: 13:52:10 Egypt Standard Time

Item name: Sep257+ve

Item description: Mervat253

Channel name: High energy : Time 6.6255 +/- 0.0237 minutes

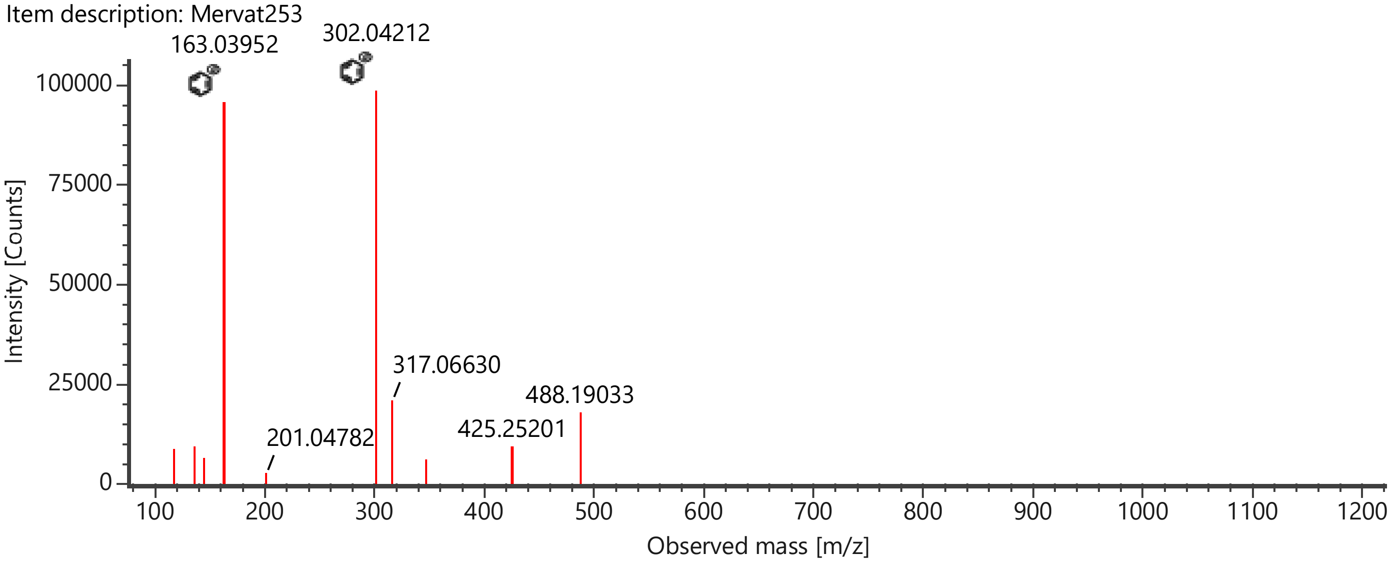

Component name: (E)-Calamenene

Item name: Sep257+ve
Channel name: (E)-Calamenene [+H] : (48.1 PPM) 203.1795

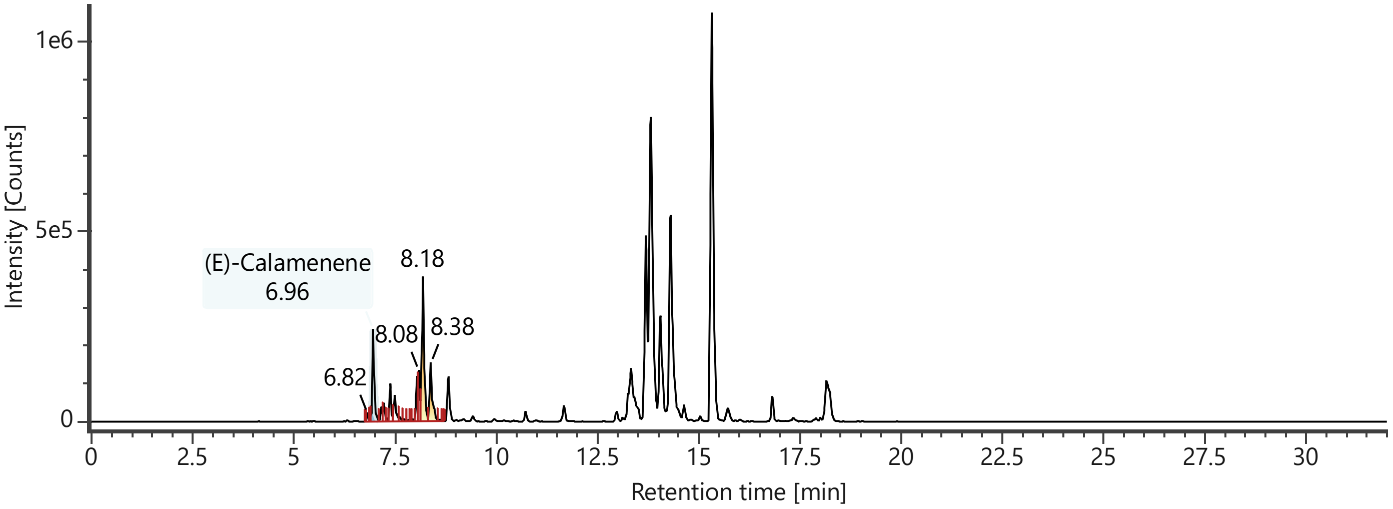

Item name: Sep257+ve
Item description: Mervat253
Channel name: Low energy : Time 6.9610 +/- 0.0237 minutes

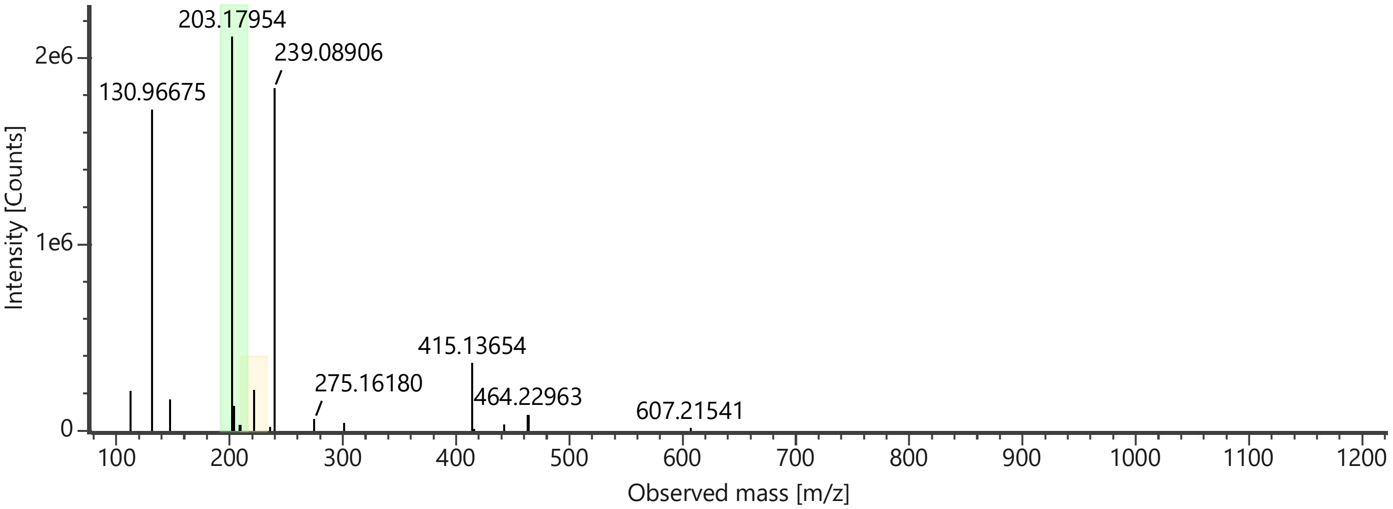

Item name: Sep257+ve
Item description: Mervat253

Channel name: High energy : Time 6.9610 +/- 0.0237 minutes

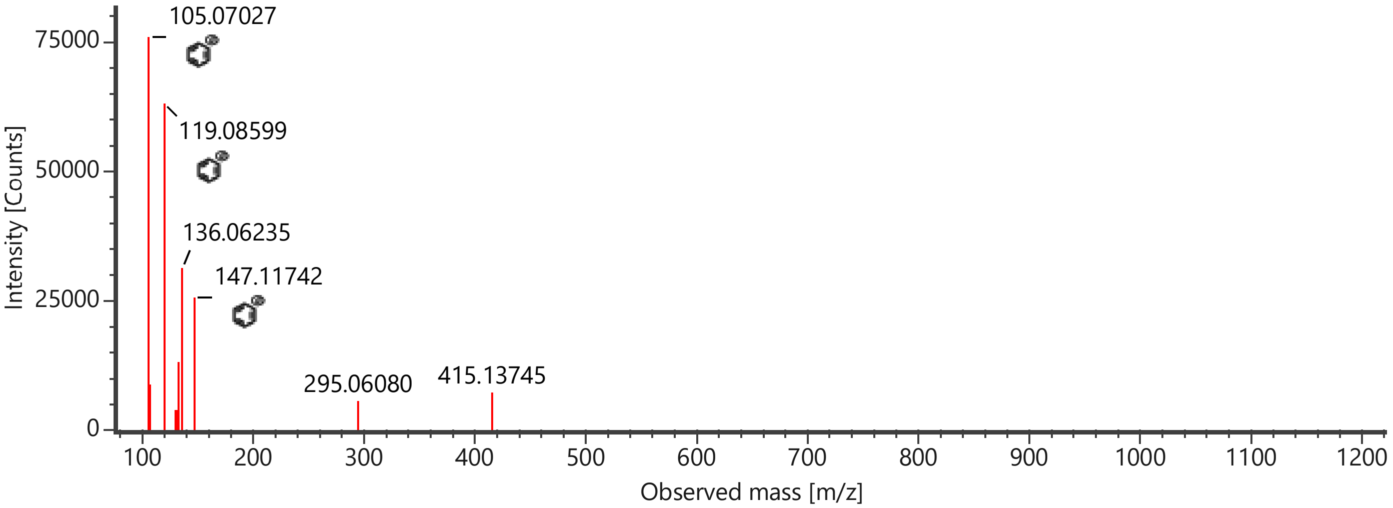

Component name: Apigenin

Item name: Sep257+ve
Channel name: Apigenin [+H] : (48.1 PPM) 271.0598

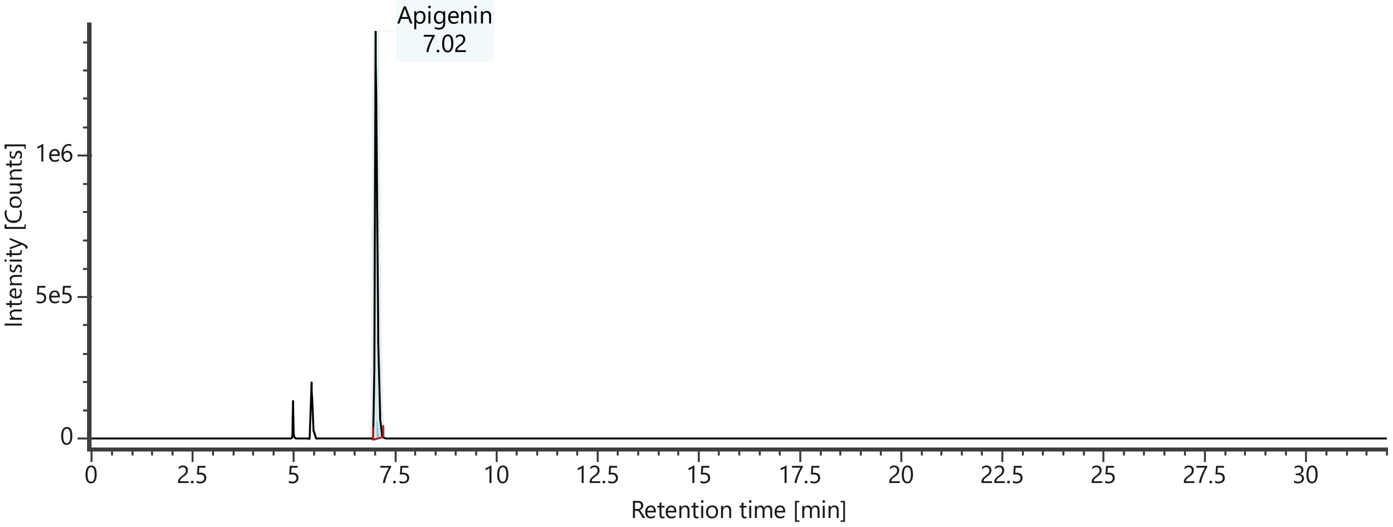

Item name: Sep257+ve
Item description: Mervat253

Channel name: Low energy : Time 7.0189 +/- 0.0237 minutes

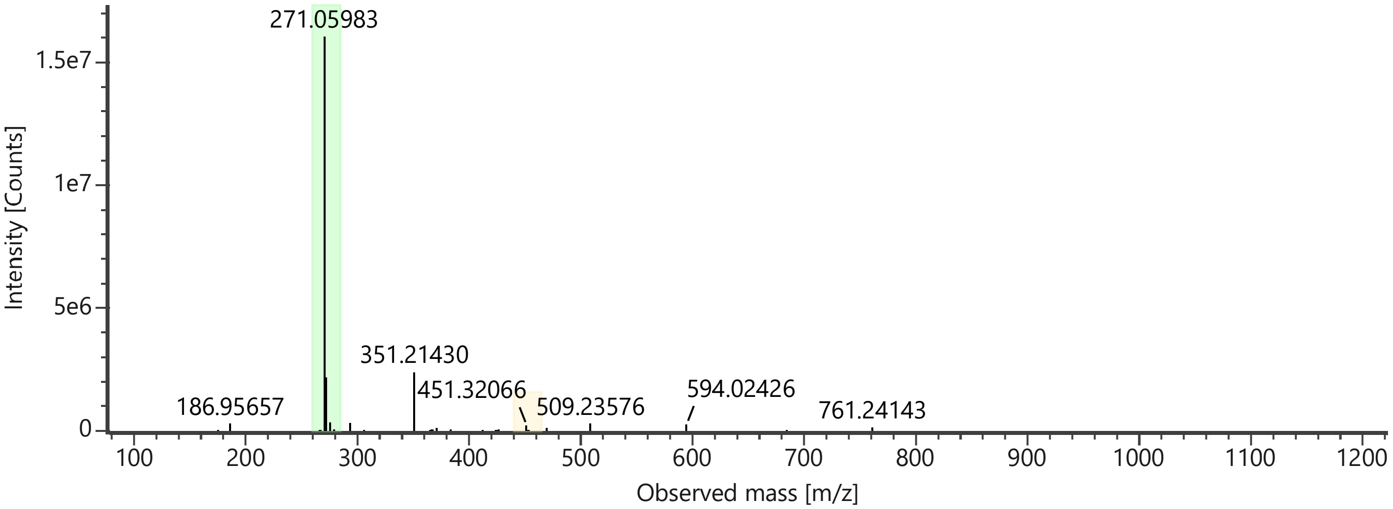

Created by: Ayad, Younan

Created on: Sep 18, 2025

Item name: Lamiaceae Family +ve mode

Created time: 13:52:10 Egypt Standard Time

Item name: Sep257+ve
Item description: Mervat253

Channel name: High energy : Time 7.0189 +/- 0.0237 minutes

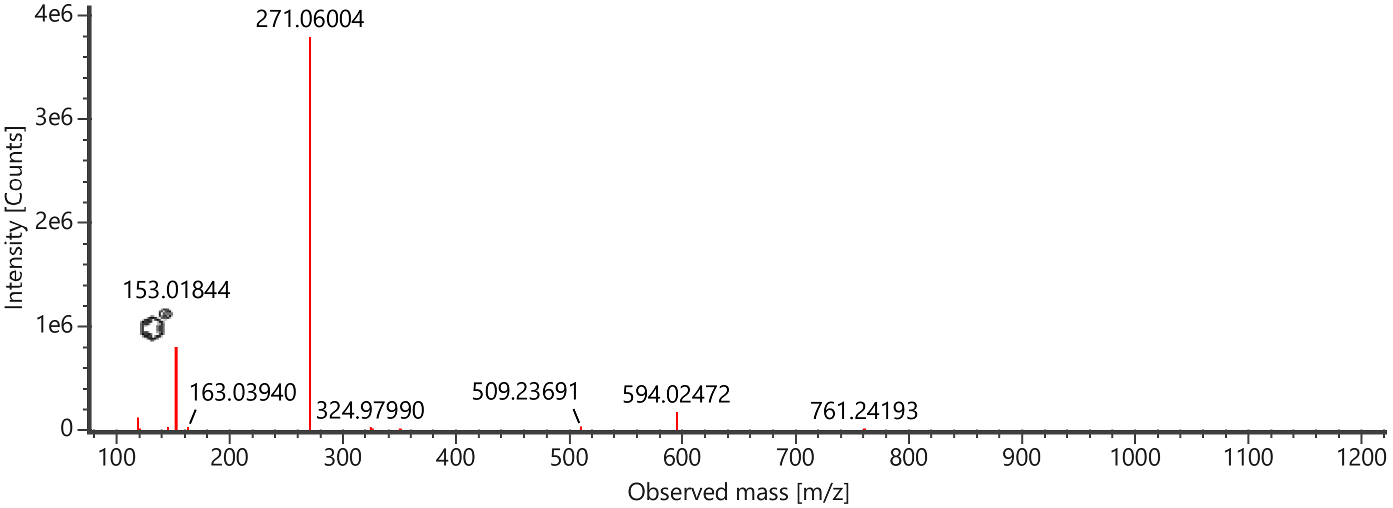

Component name: Diosmetin

Item name: Sep257+ve
Channel name: Diosmetin [+H] : (48.1 PPM) 301.0718

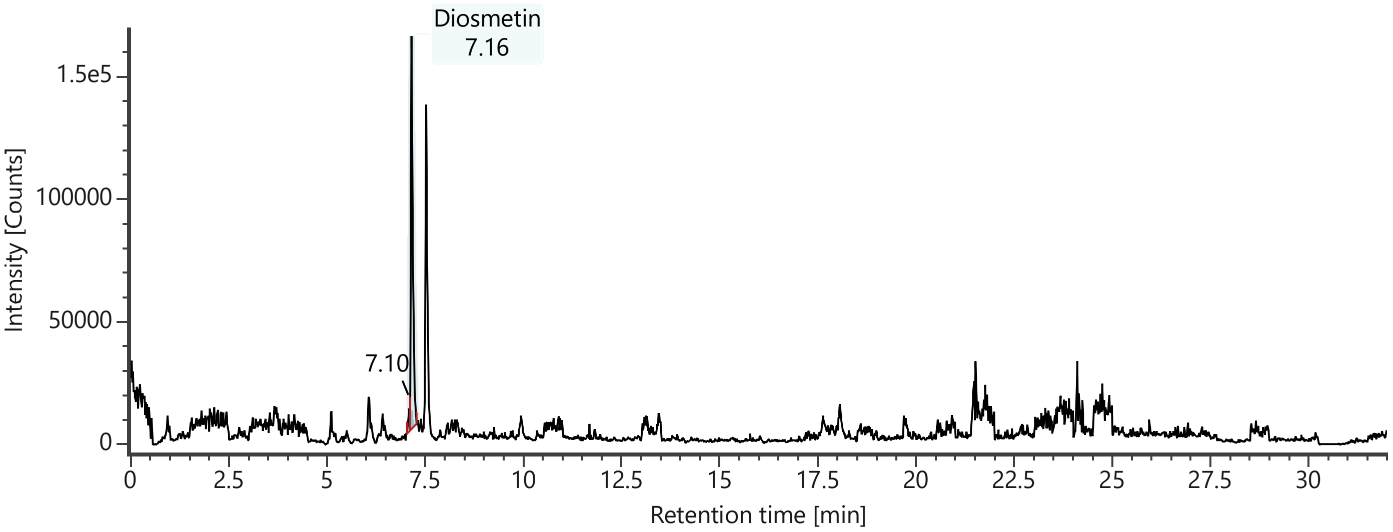

Item name: Sep257+ve
Item description: Mervat253
Channel name: Low energy : Time 7.1604 +/- 0.0237 minutes

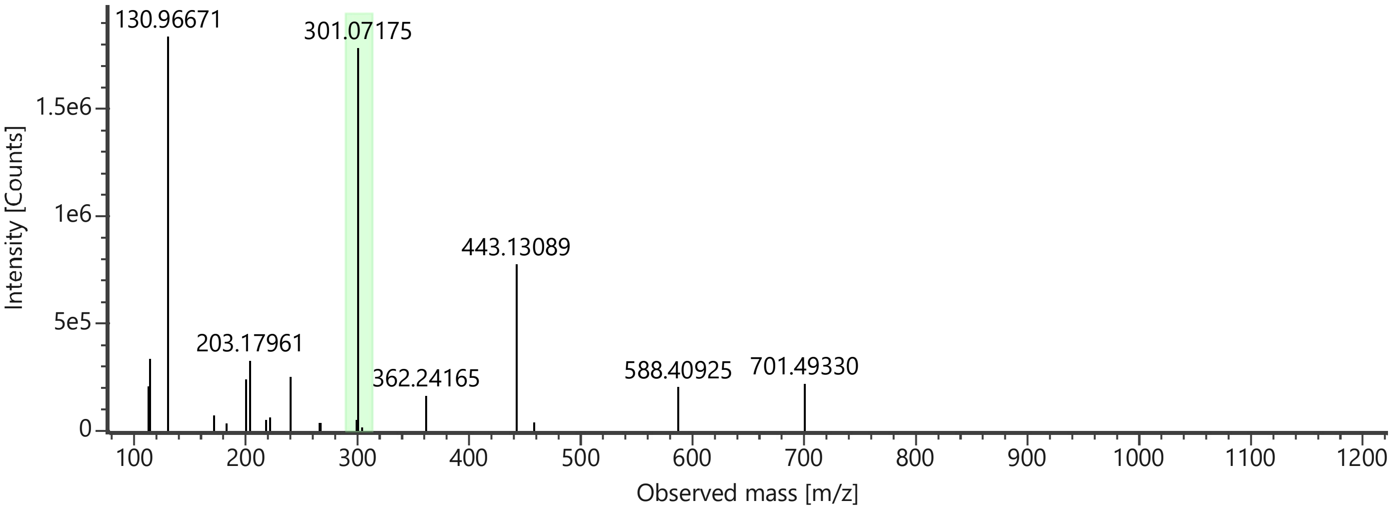

Created by: Ayad, Younan

Created on: Sep 18, 2025

Item name: Lamiaceae Family +ve mode

Created time: 13:52:10 Egypt Standard Time

Item name: Sep257+ve
Item description: Mervat253

Channel name: High energy : Time 7.1604 +/- 0.0237 minutes

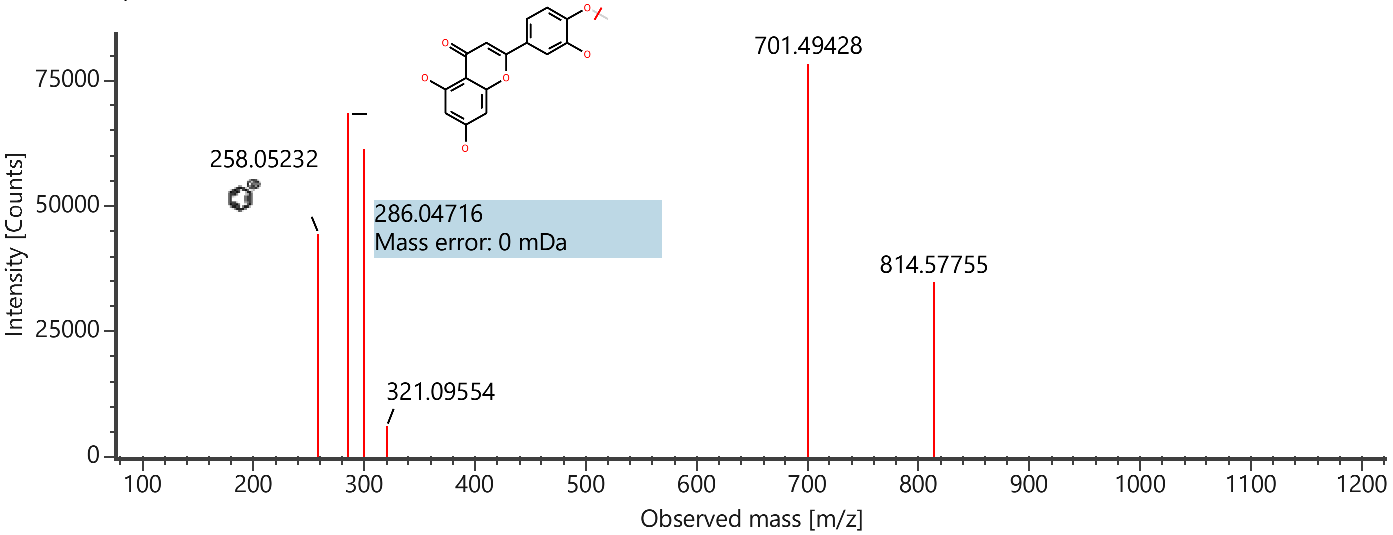

Component name: Quercetin 3,4'-dimethyl ether

Item name: Sep257+ve
Channel name: Quercetin 3,4'-dimethyl ether [+H] : (48.1 PPM) 331.0811

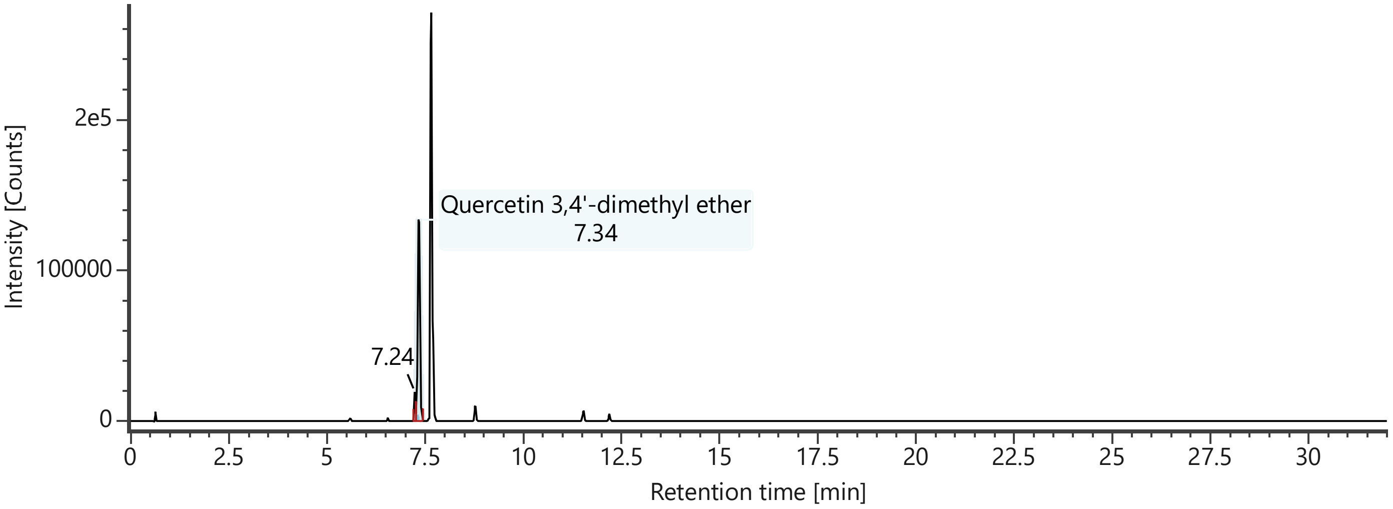

Item name: Sep257+ve
Item description: Mervat253
Channel name: Low energy : Time 7.3358 +/- 0.0237 minutes

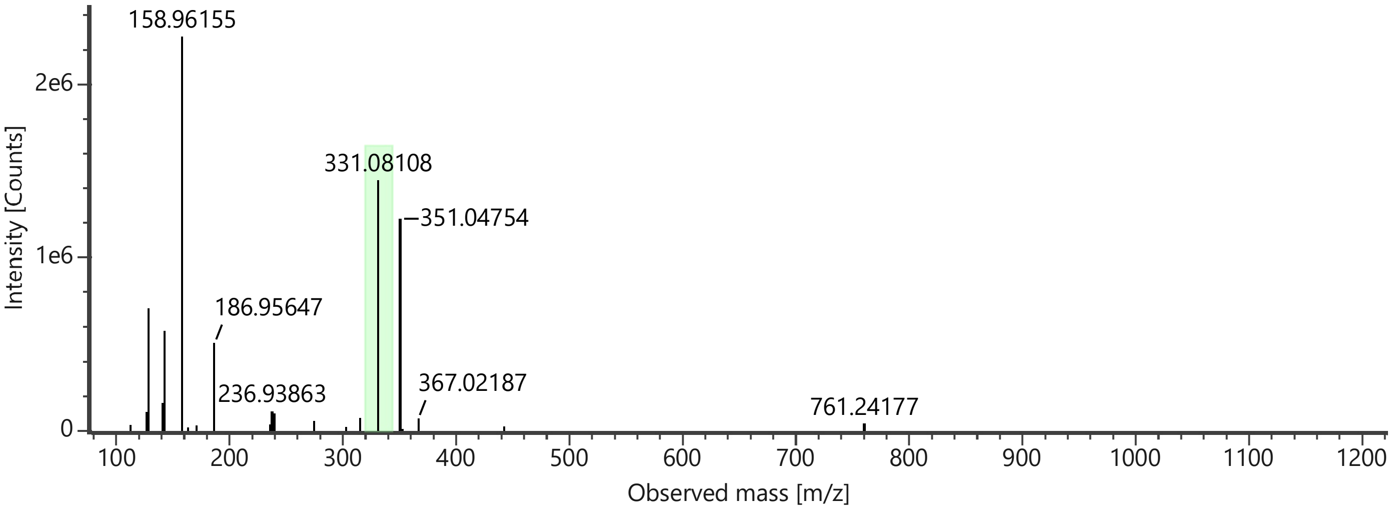

Created by: Ayad, Younan

Created on: Sep 18, 2025

Item name: Lamiaceae Family +ve mode

Created time: 13:52:10 Egypt Standard Time

Item name: Sep257+ve
Item description: Mervat253

Channel name: High energy : Time 7.3358 +/- 0.0237 minutes

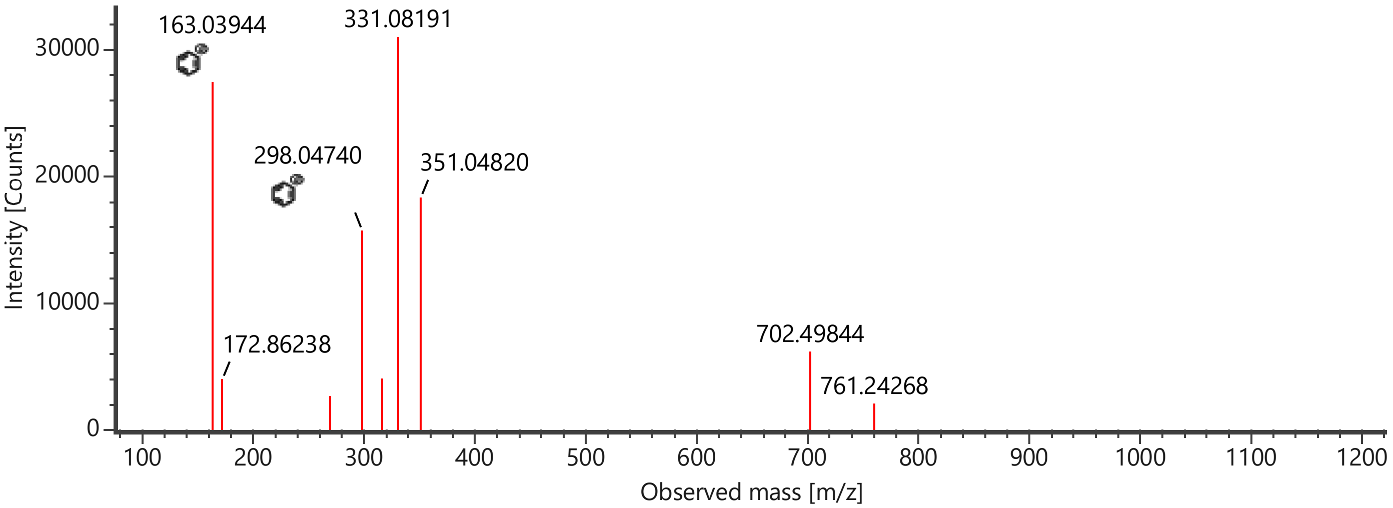

Component name: 7,8-Dihydroxy-2-(3,4-dihydroxyphenyl)-1,2-dihydronaphthalene-1,3-dicarboxylic acid

Item name: Sep257+ve
Channel name: 7,8-Dihydroxy-2-(3,4-dihydroxyphenyl)-1,2-dihydronaphthalene-1,3-dicarboxylic acid [+Na] : (48.1 PPM) 381.0582

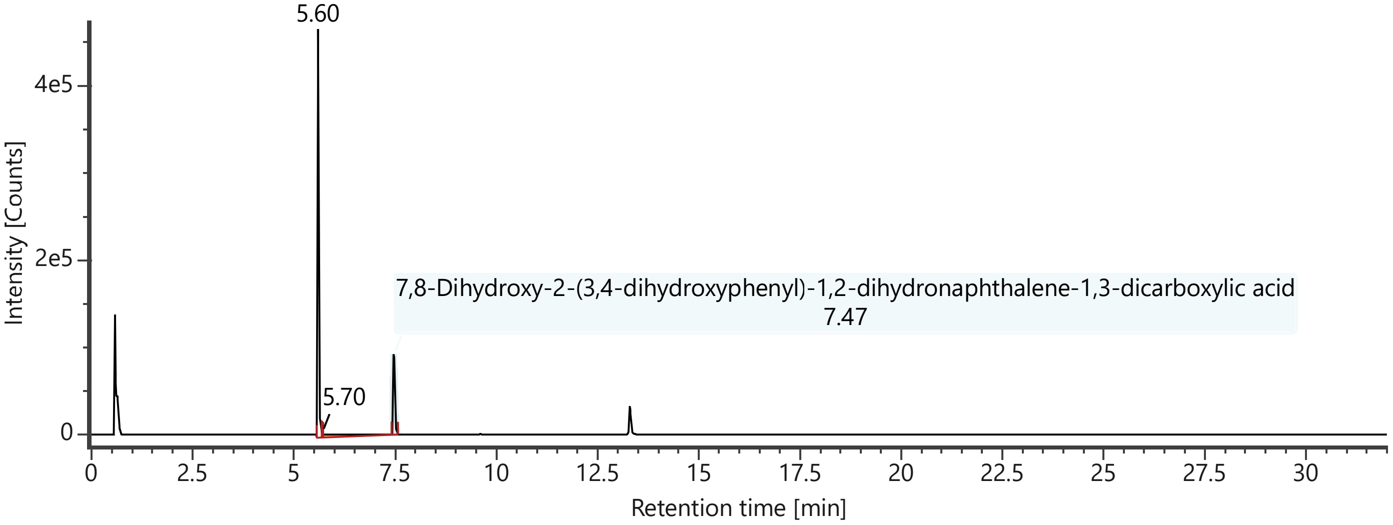

Item name: Sep257+ve
Item description: Mervat253
Channel name: Low energy : Time 7.4705 +/- 0.0237 minutes

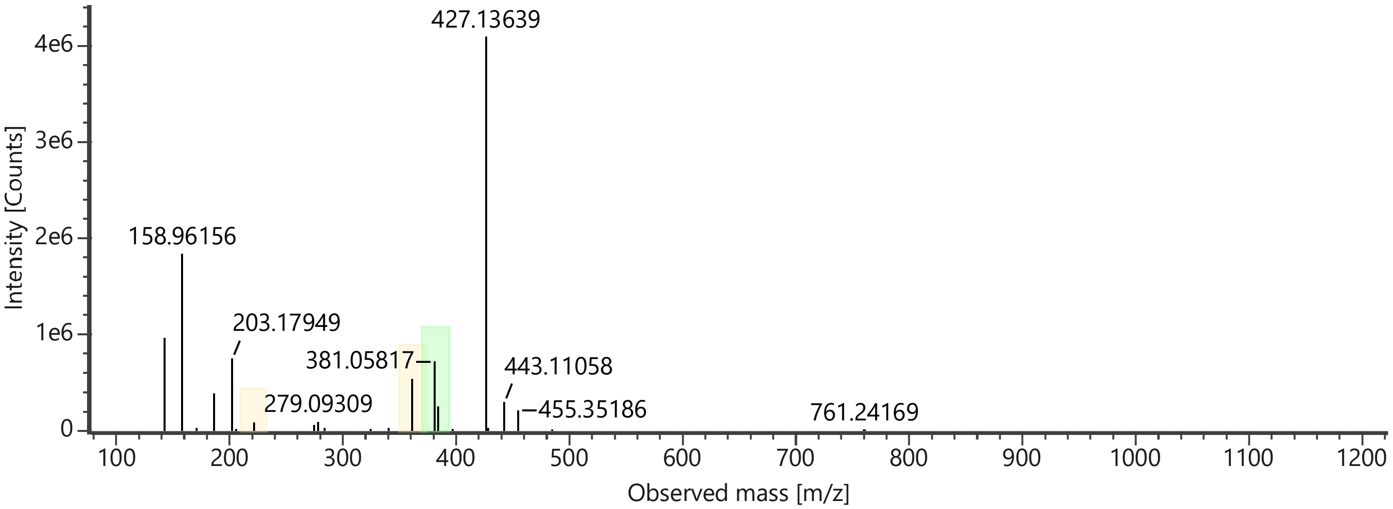

Item name: Sep257+ve
Item description: Mervat253

Channel name: High energy : Time 7.4705 +/- 0.0237 minutes

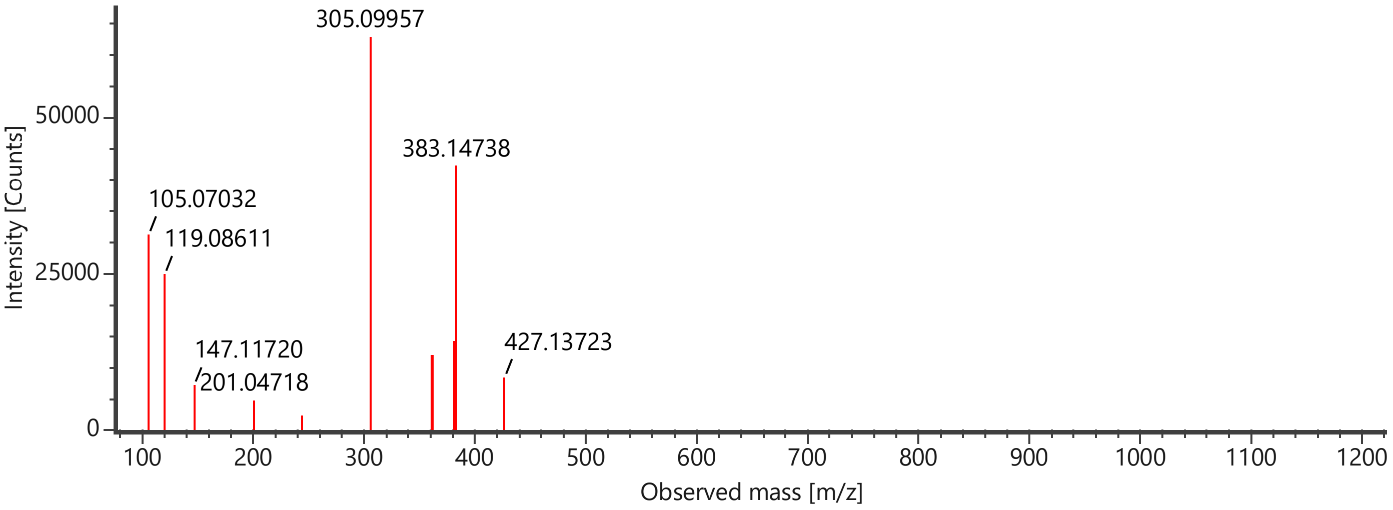

Component name: Diosmetin

Item name: Sep257+ve
Channel name: Diosmetin [+H] : (48.1 PPM) 301.0714

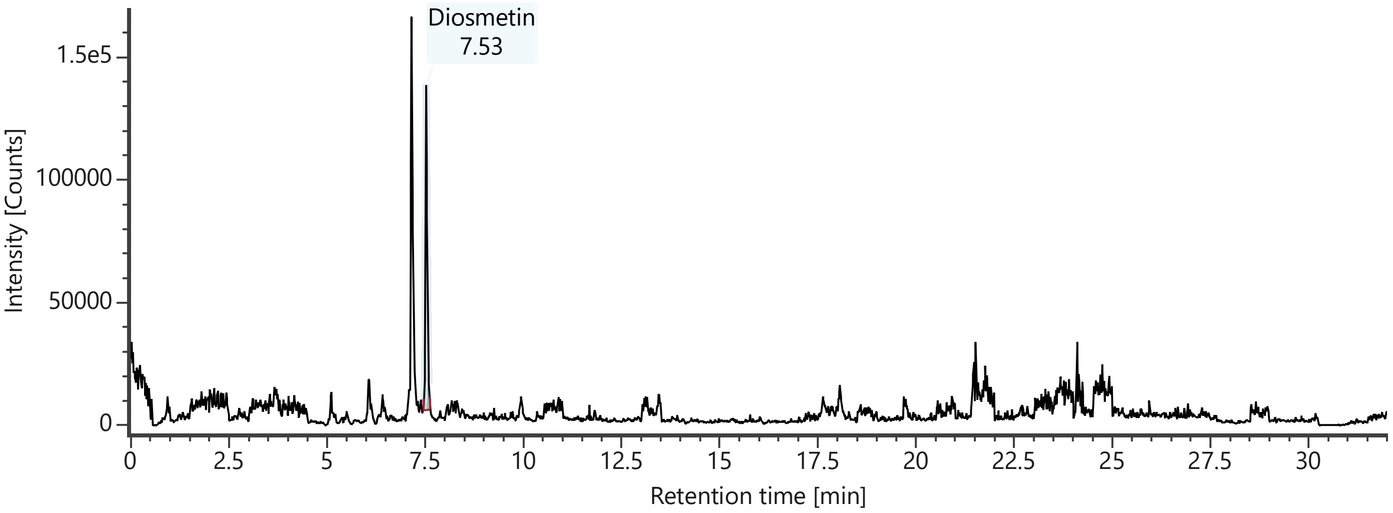

Item name: Sep257+ve
Item description: Mervat253
Channel name: Low energy : Time 7.5291 +/- 0.0237 minutes

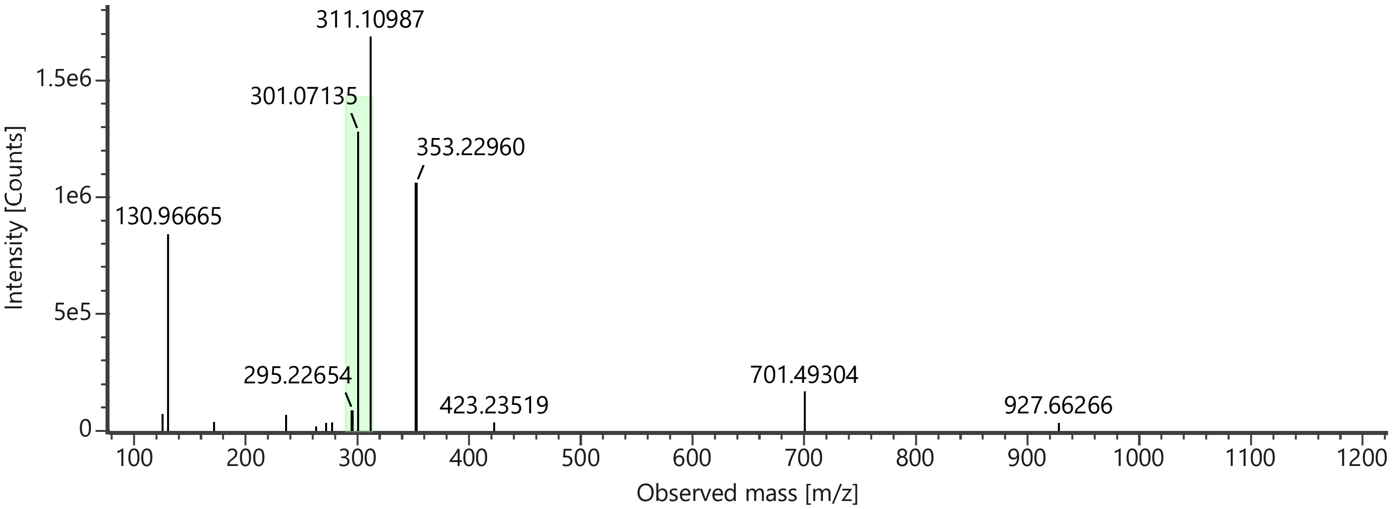

Item name: Sep257+ve
Item description: Mervat253

Channel name: High energy : Time 7.5291 +/- 0.0237 minutes

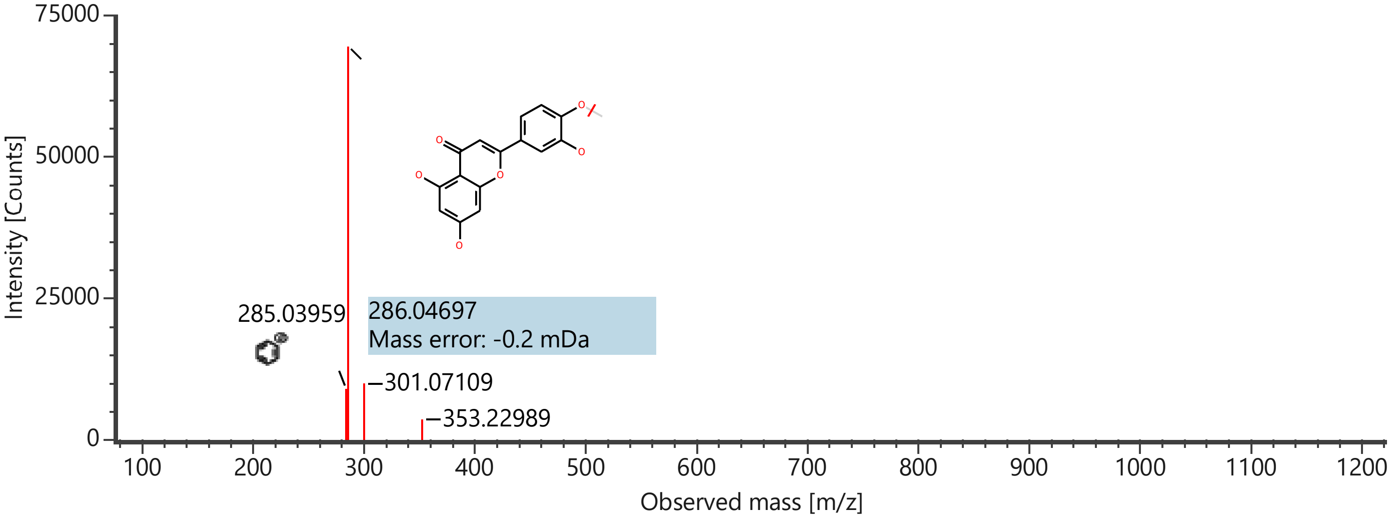

Component name: Quercetin 3,4'-dimethyl ether

Item name: Sep257+ve
Channel name: Quercetin 3,4'-dimethyl ether [+H] : (48.1 PPM) 331.0808

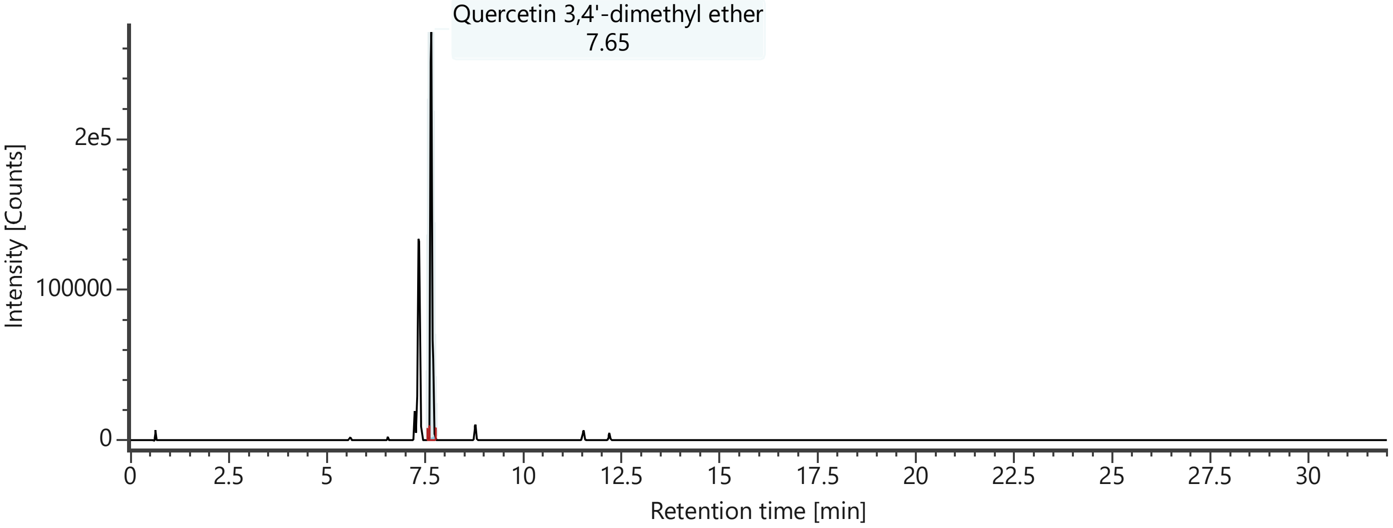

Item name: Sep257+ve
Item description: Mervat253
Channel name: Low energy : Time 7.6571 +/- 0.0237 minutes

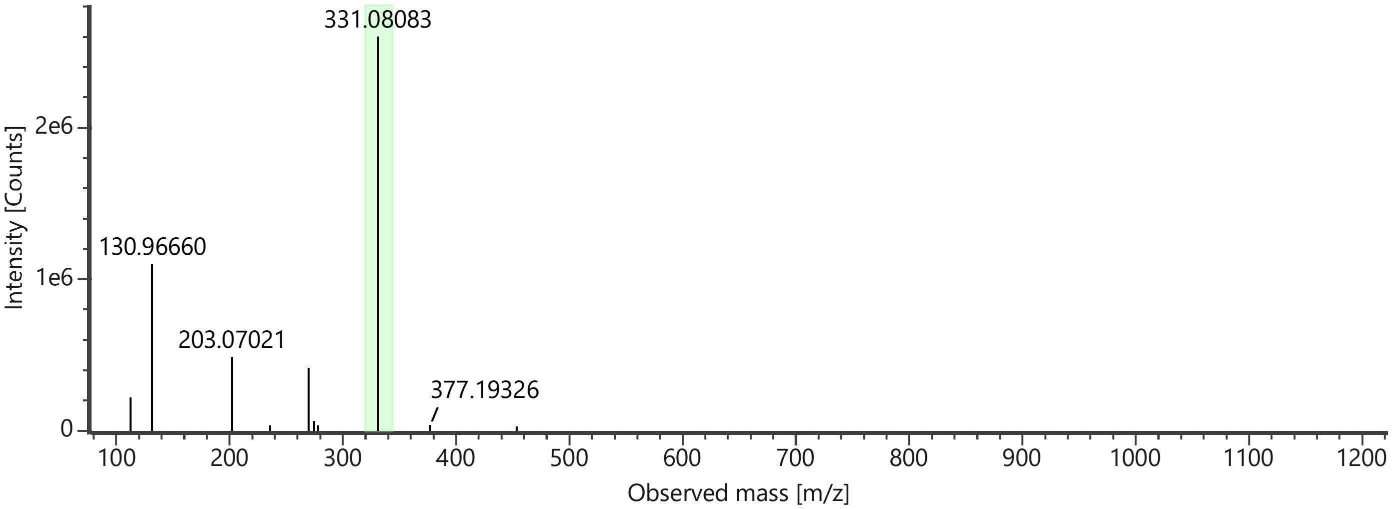

Created by: Ayad, Younan

Created on: Sep 18, 2025

Item name: Lamiaceae Family +ve mode

Created time: 13:52:10 Egypt Standard Time

Item name: Sep257+ve

Item description: Mervat253

Channel name: High energy : Time 7.6571 +/- 0.0237 minutes

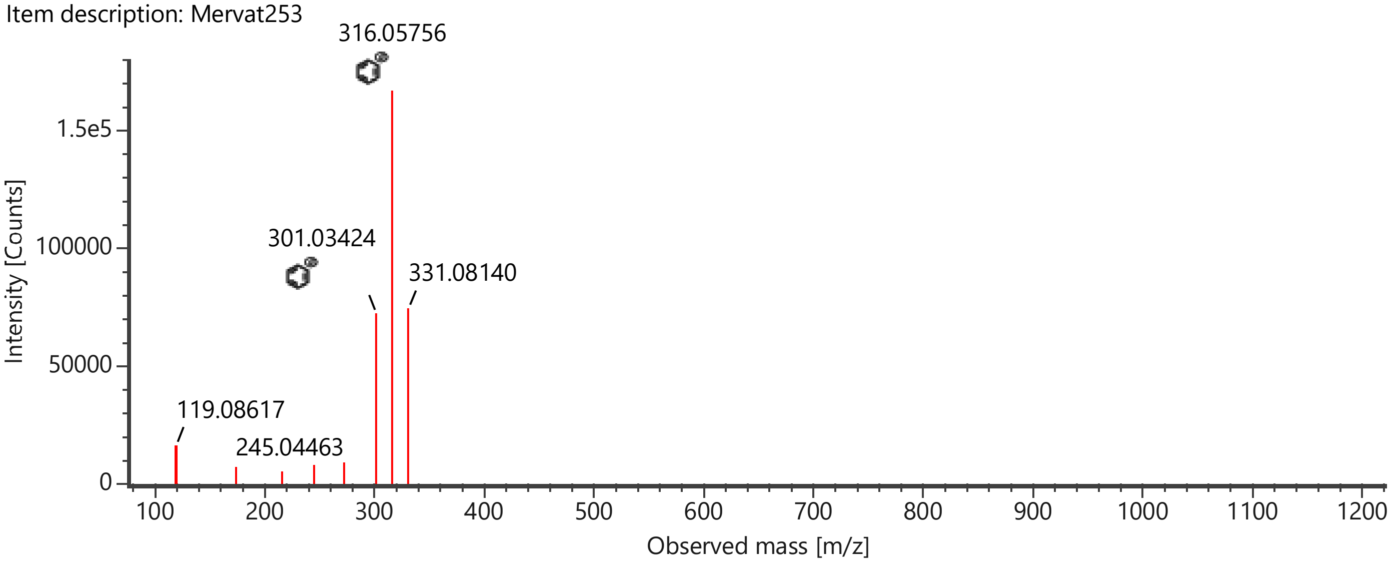

Component name: (1aR,4aS,7R,7aR,7bR)-1,1,7-Trimethyl-4-methylidenedecahydro-1H-cyclopropa(e)azulen-7-ol

Item name: Sep257+ve
Channel name: (1aR,4aS,7R,7aR,7bR)-1,1,7-Trimethyl-4-methylidenedecahydro-1H-cyclopropa(e)azulen-7-ol [+H] : (48.1 PPM) 221.1898

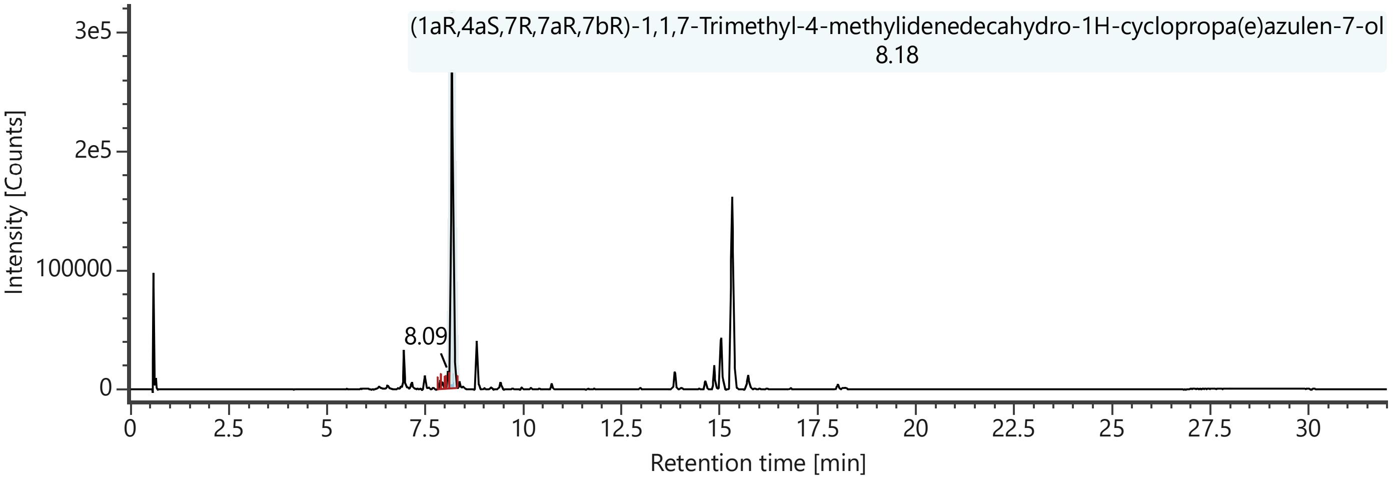

Item name: Sep257+ve
Item description: Mervat253
Channel name: Low energy : Time 8.1856 +/- 0.0237 minutes

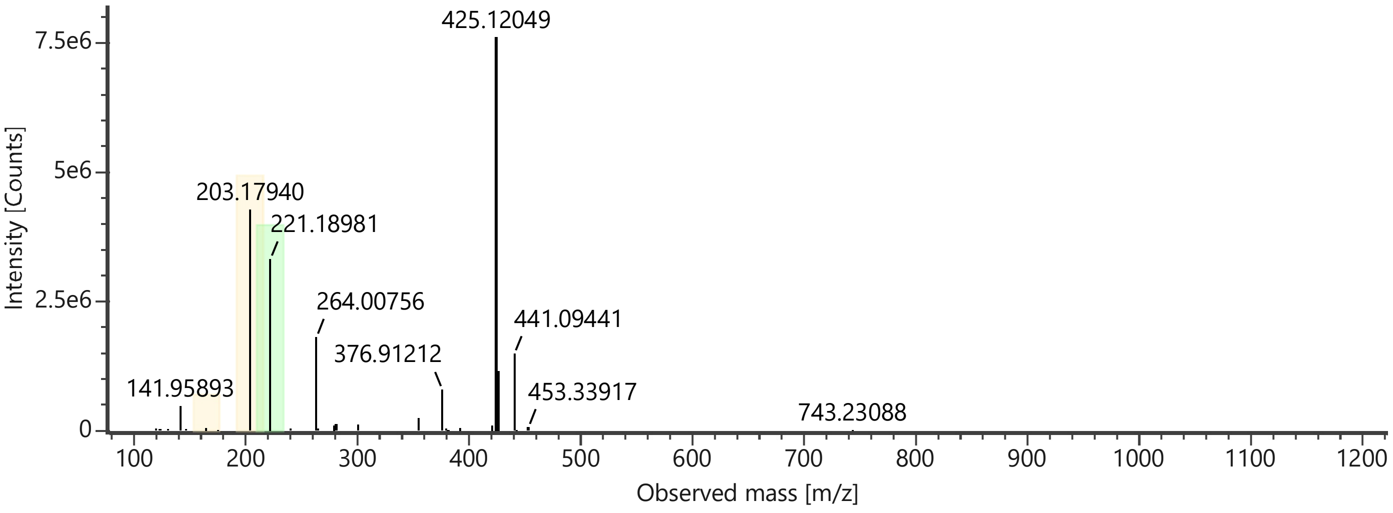

Item name: Sep257+ve
Item description: Mervat253

Channel name: High energy : Time 8.1856 +/- 0.0237 minutes

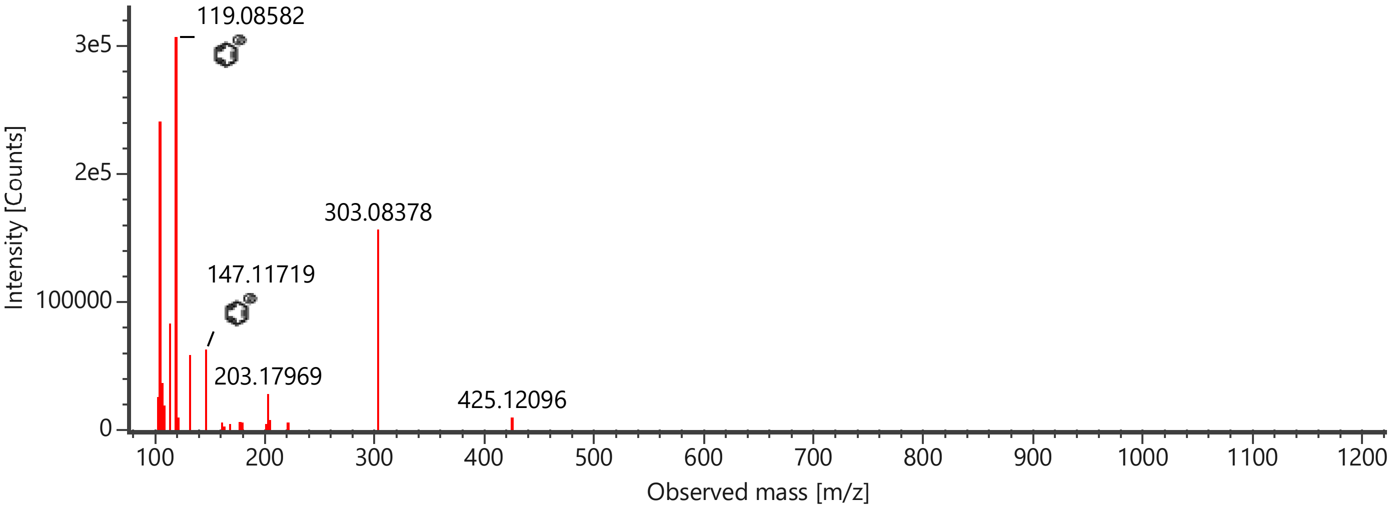

Component name: (E)-Calamenene

Item name: Sep257+ve
Channel name: (E)-Calamenene [+H] : (48.1 PPM) 203.1794

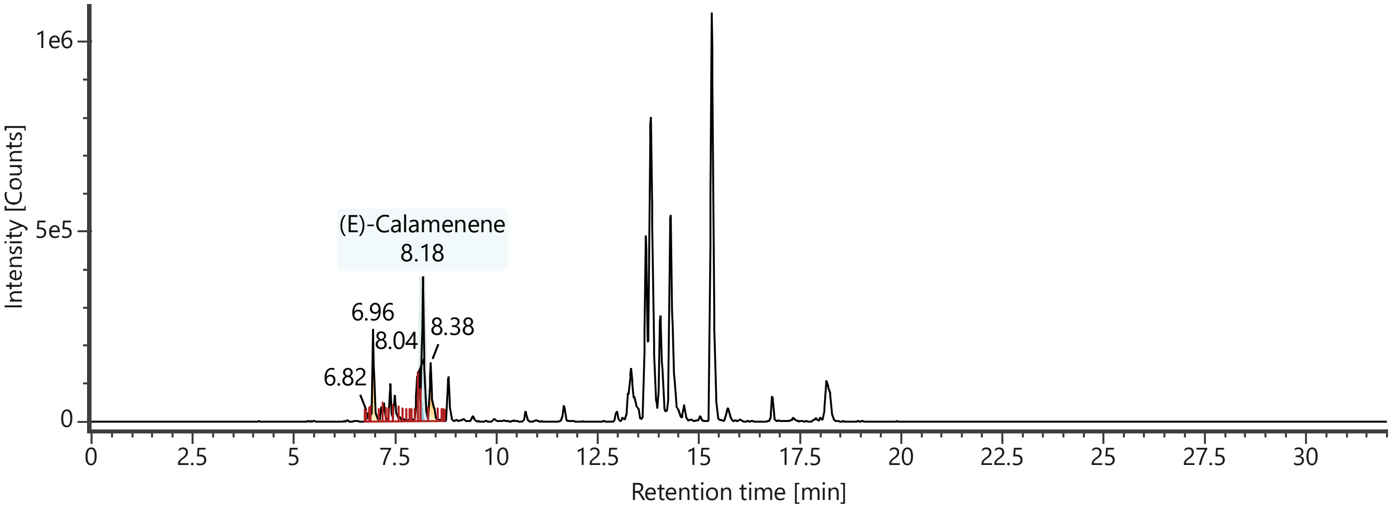

Item name: Sep257+ve
Item description: Mervat253
Channel name: Low energy : Time 8.1857 +/- 0.0237 minutes

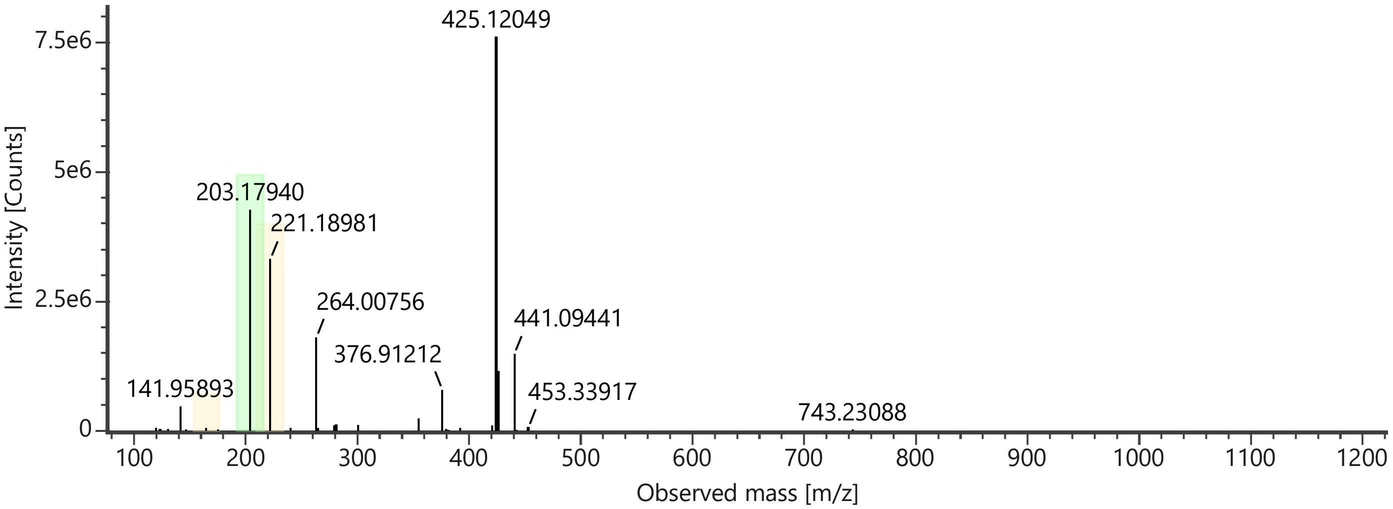

Item name: Sep257+ve
Item description: Mervat253

Channel name: High energy : Time 8.1857 +/- 0.0237 minutes

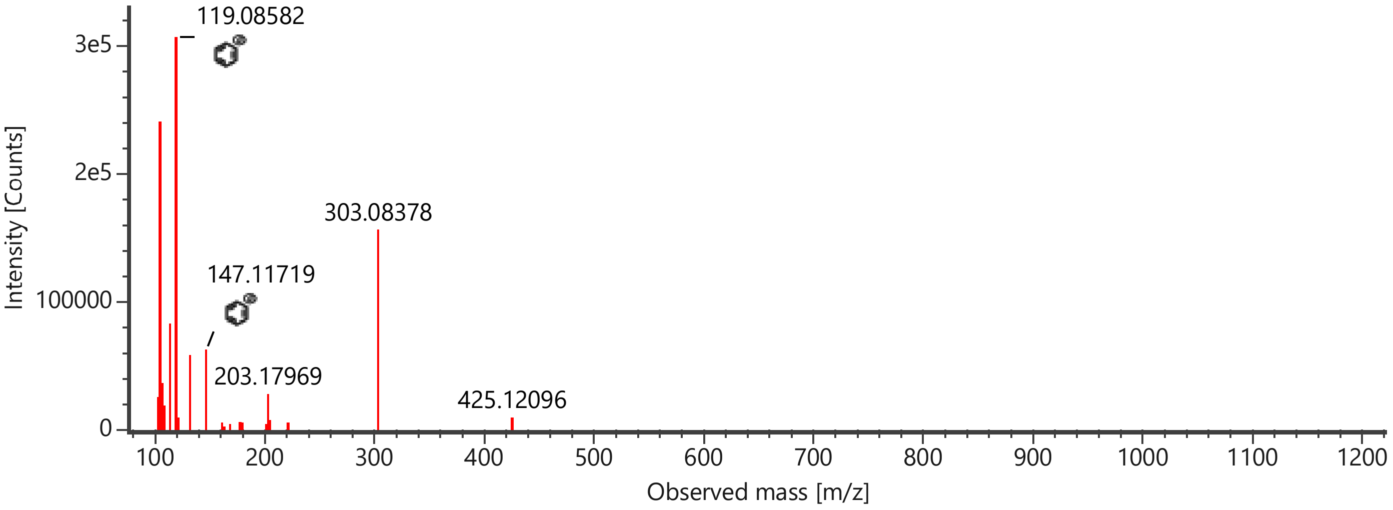

Component name: (E)-Calamenene

Item name: Sep257+ve
Channel name: (E)-Calamenene [+H] : (48.1 PPM) 203.1795

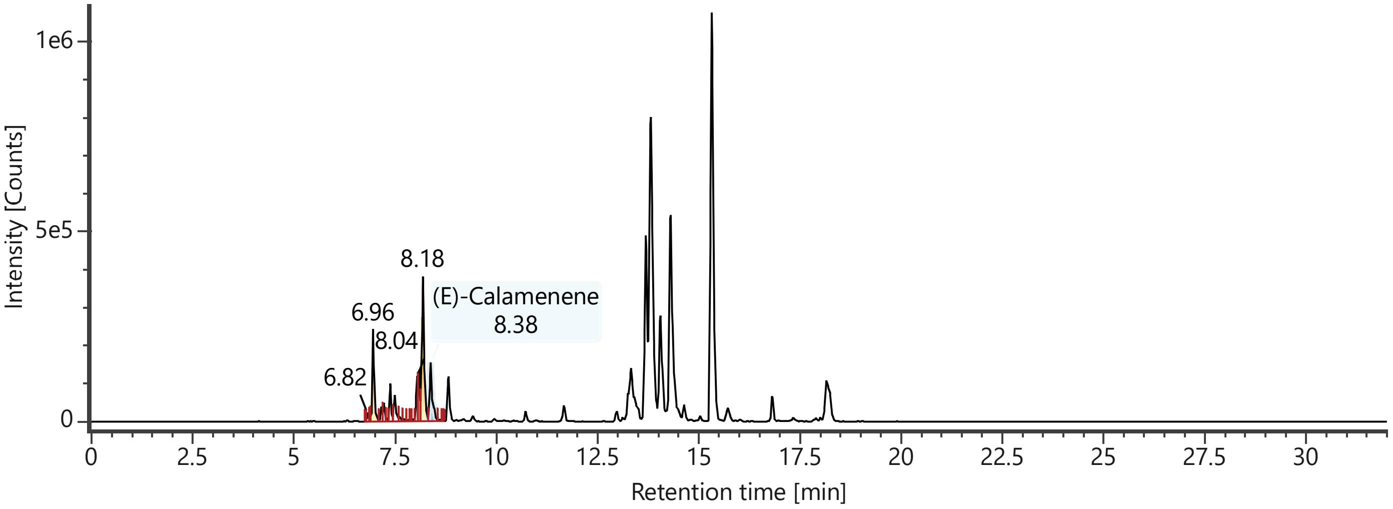

Item name: Sep257+ve
Item description: Mervat253
Channel name: Low energy : Time 8.3825 +/- 0.0237 minutes

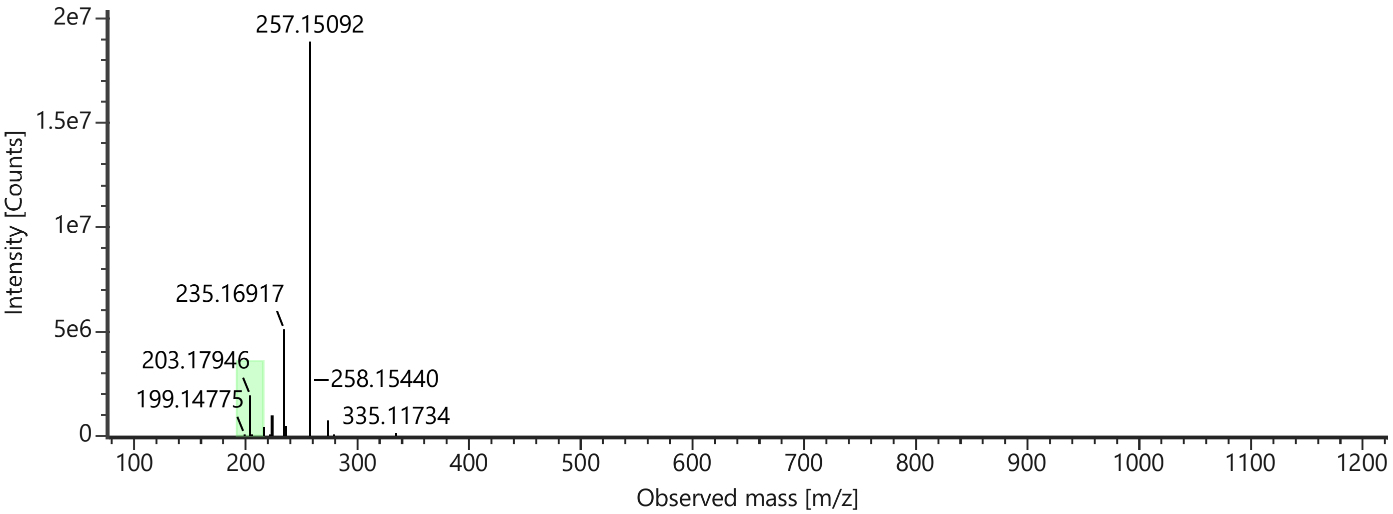

Item name: Sep257+ve
Item description: Mervat253

Channel name: High energy : Time 8.3825 +/- 0.0237 minutes

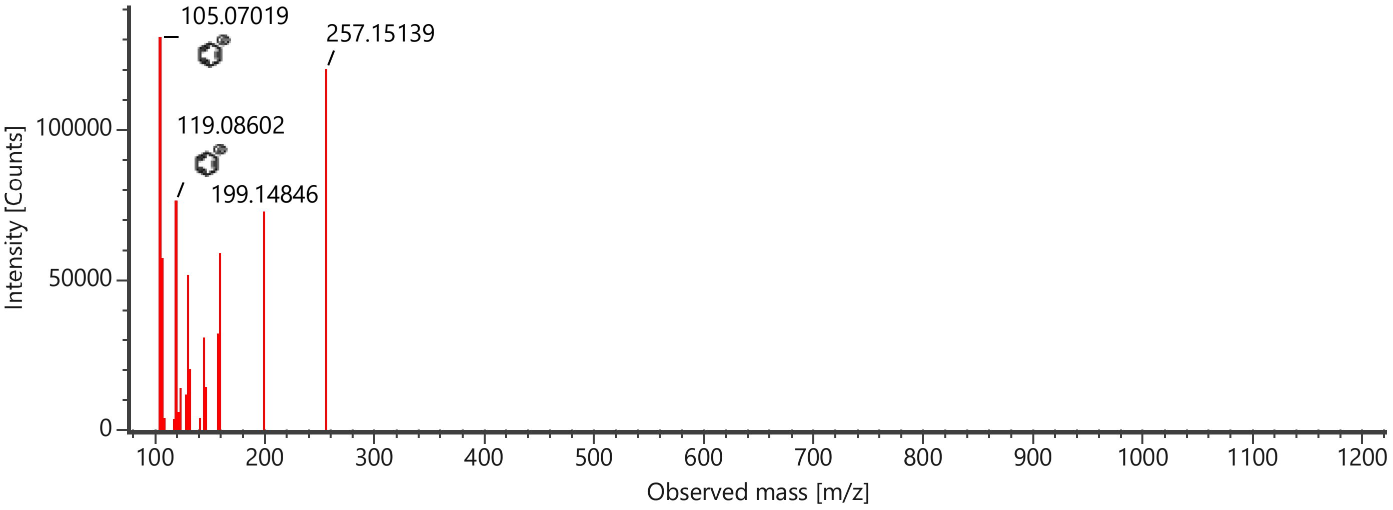

Component name: Quercetin tetramethyl(3',4',5,7) ether

Item name: Sep257+ve
Channel name: Quercetin tetramethyl(3',4',5,7) ether [+H] : (48.1 PPM) 359.1133

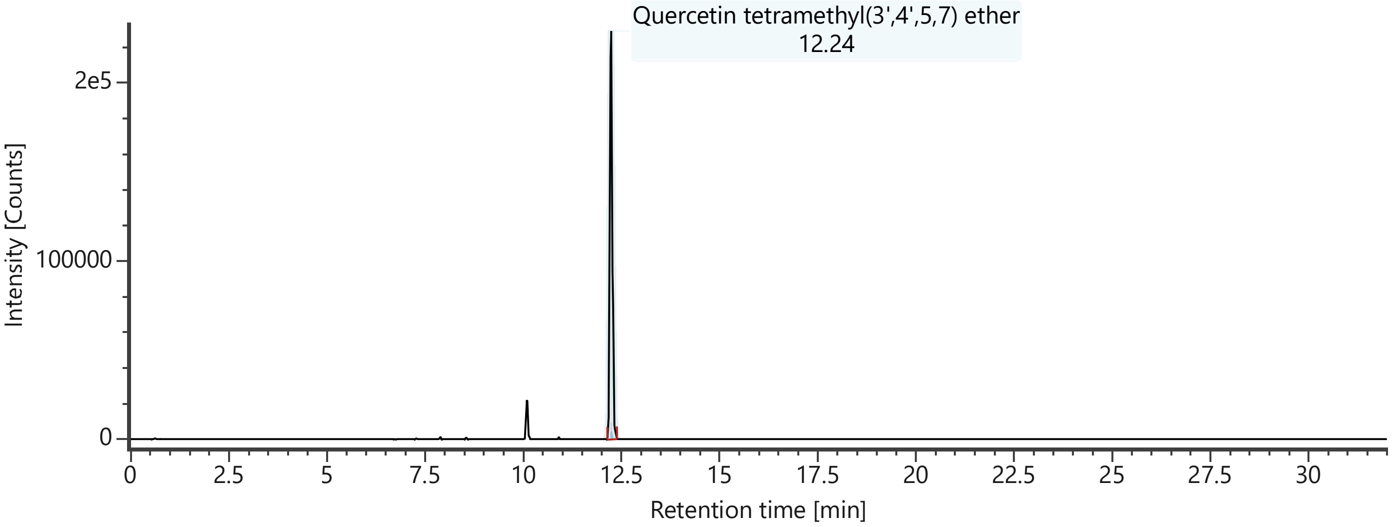

Item name: Sep257+ve
Item description: Mervat253
Channel name: Low energy : Time 12.2411 +/- 0.0237 minutes

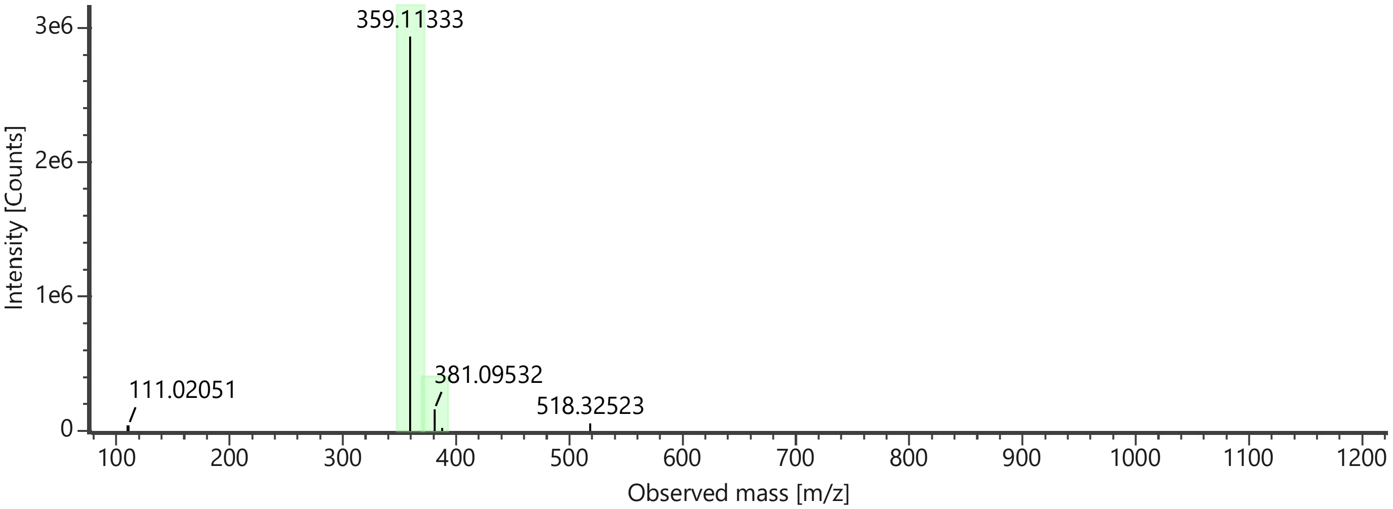

Item name: Sep257+ve
Item description: Mervat253

Channel name: High energy : Time 12.2411 +/- 0.0237 minutes

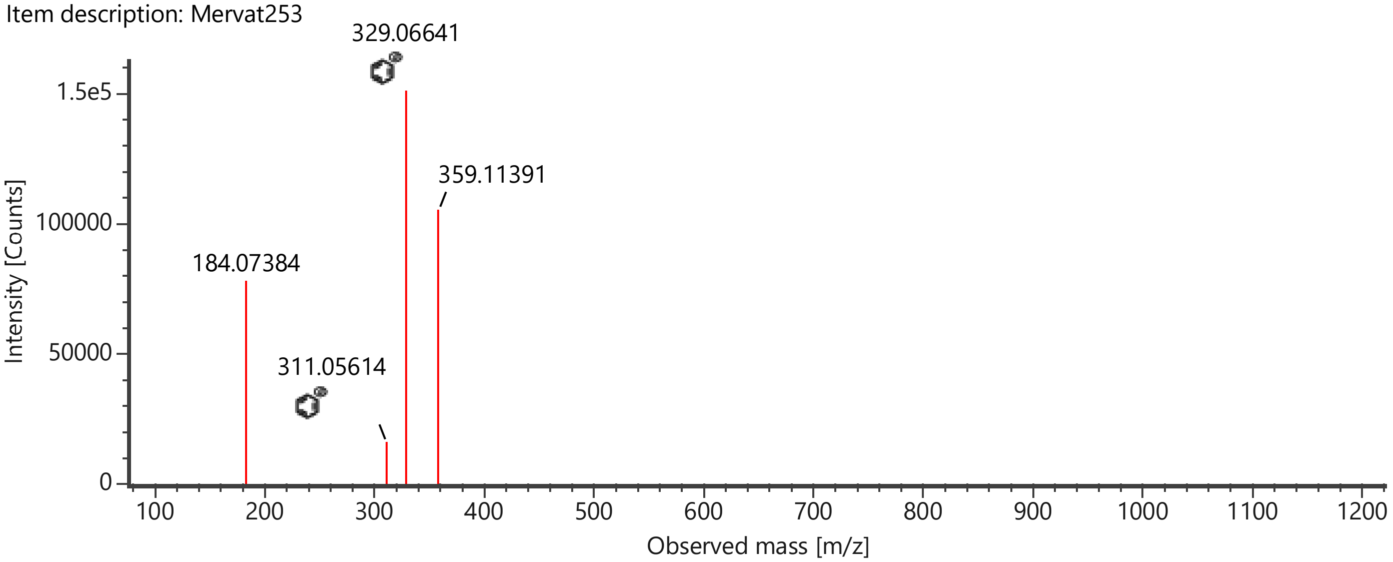

Component name: -Ca amenene

Item name: Sep257+ve
Channel name: (E)-Calamenene [+H] : (48.1 PPM) 203.1796

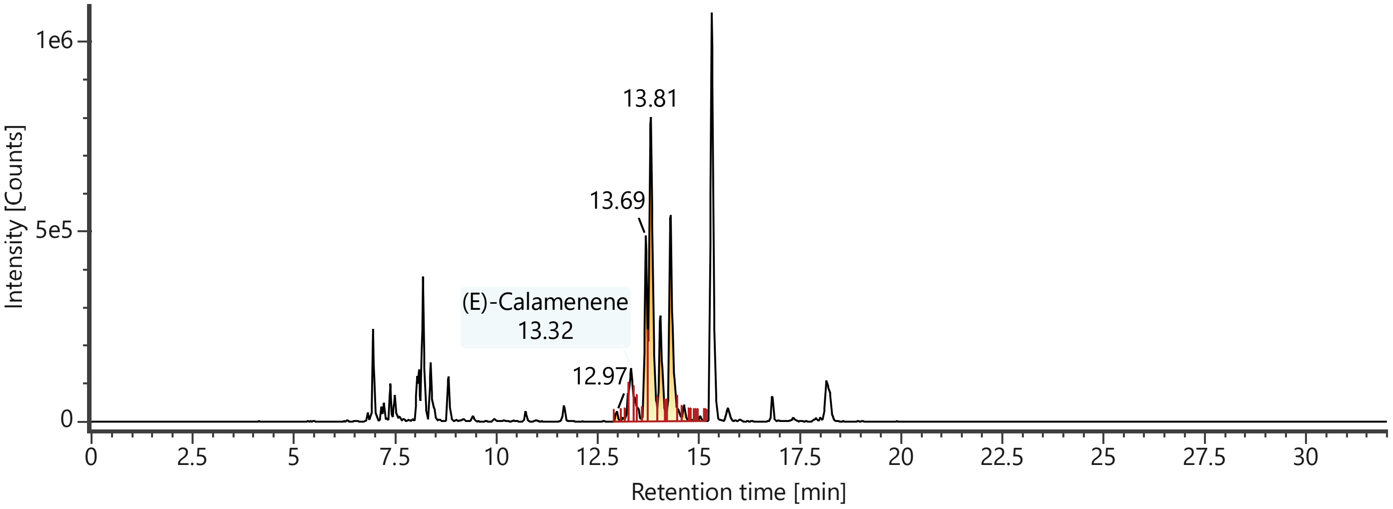

Item name: Sep257+ve
Item description: Mervat253
Channel name: Low energy : Time 13.3273 +/- 0.0237 minutes

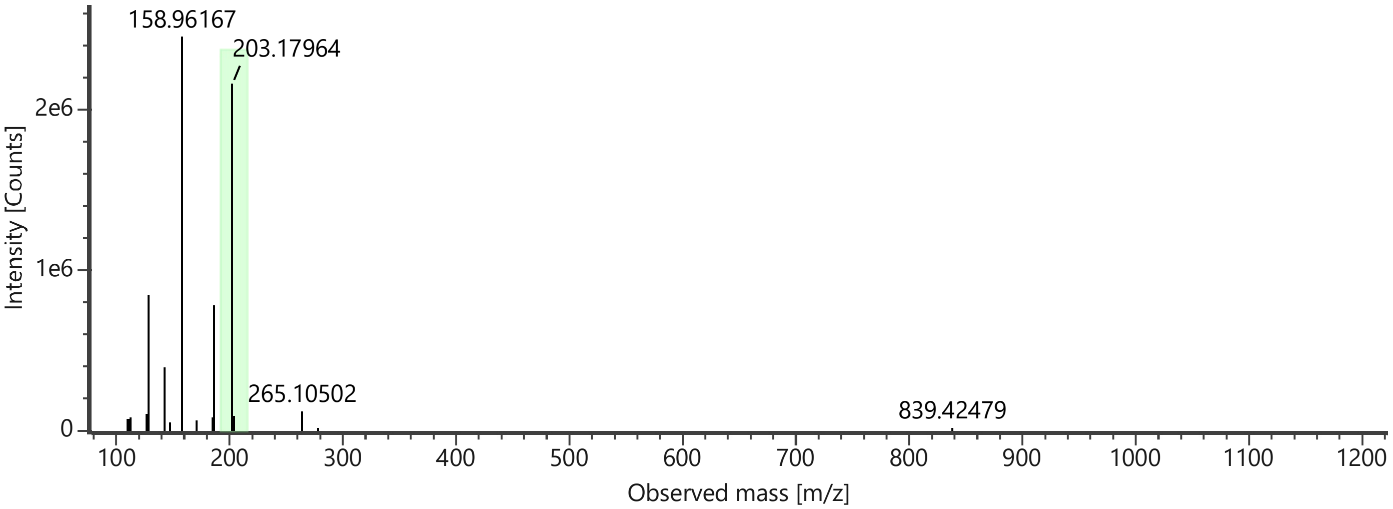

Created by: Ayad, Younan

Created on: Sep 18, 2025

Item name: Lamiaceae Family +ve mode

Created time: 13:52:10 Egypt Standard Time

Item name: Sep257+ve

Item description: Mervat253

Channel name: High energy : Time 13.3273 +/- 0.0237 minutes

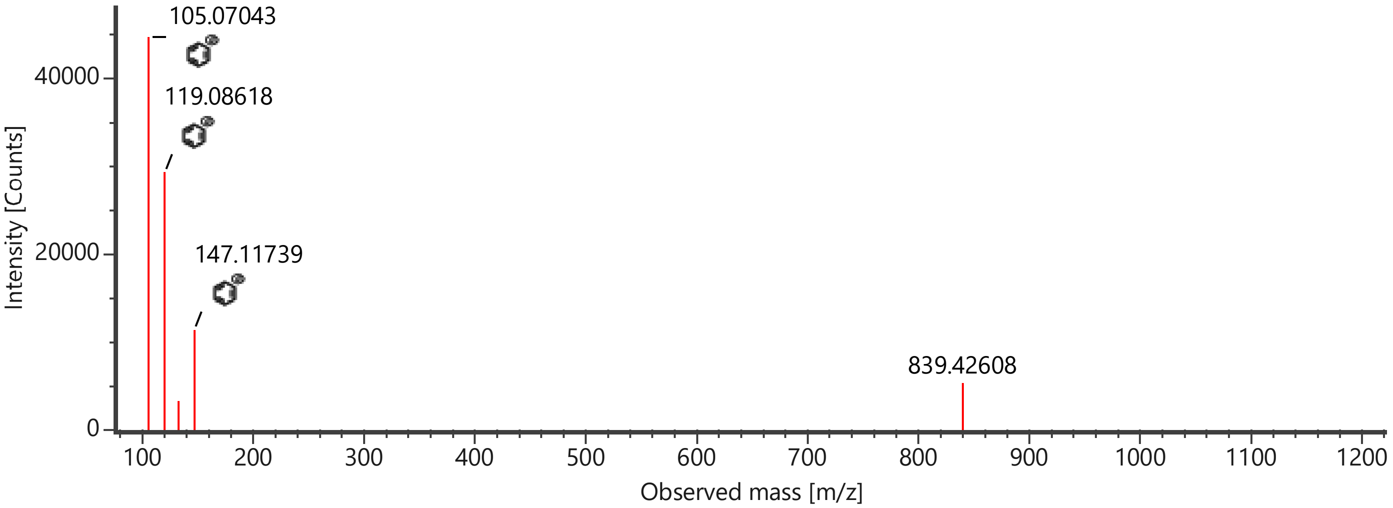

Component name: -Calamenene

Item name: Sep257+ve
Channel name: (E)-Calamenene [+H] : (48.1 PPM) 203.1795

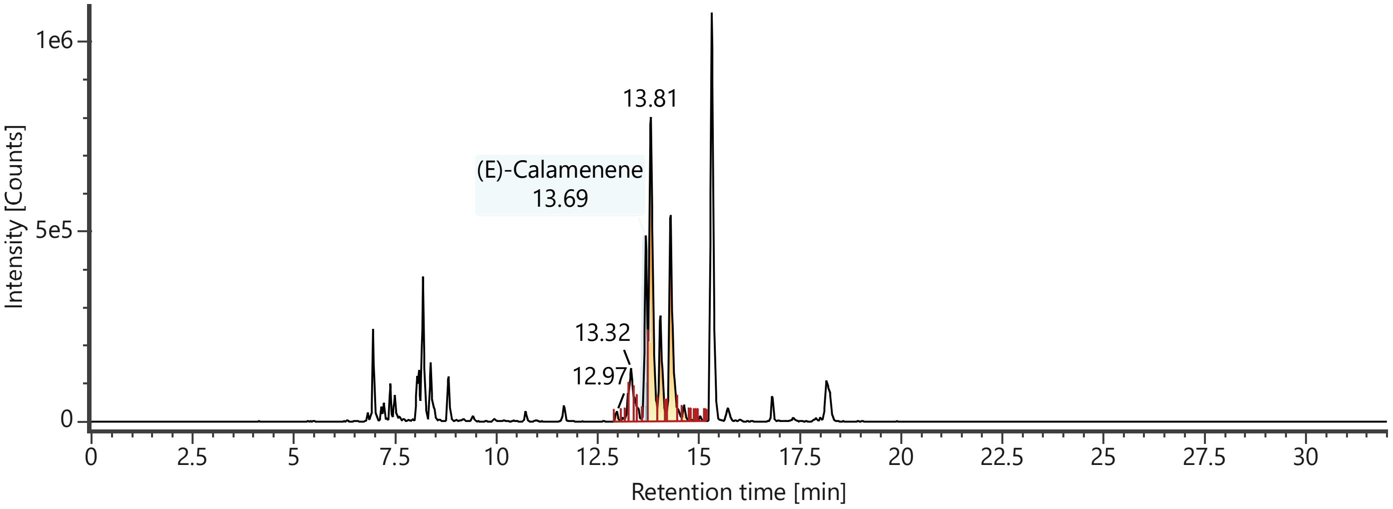

Item name: Sep257+ve
Item description: Mervat253
Channel name: Low energy : Time 13.6902 +/- 0.0237 minutes

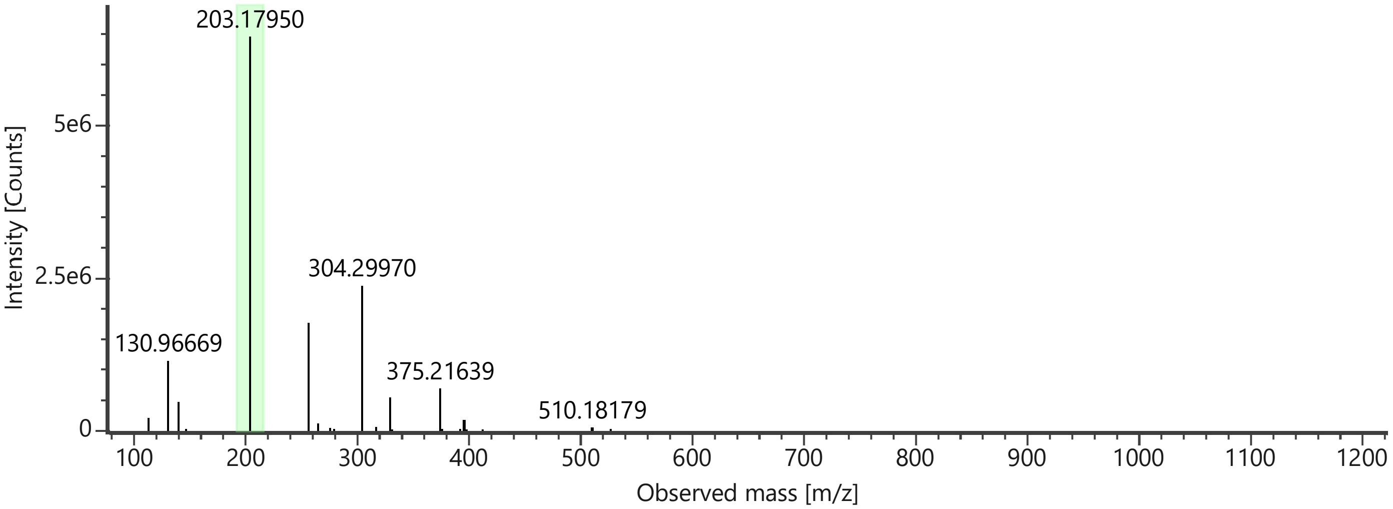

Item name: Sep257+ve
Item description: Mervat253

Channel name: High energy : Time 13.6902 +/- 0.0237 minutes

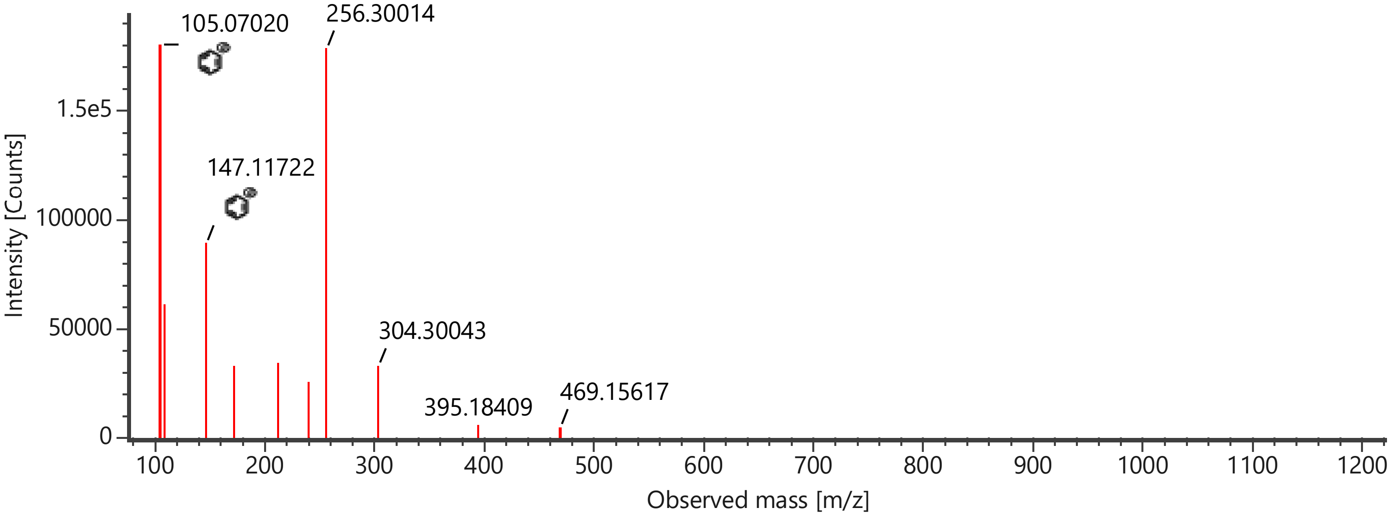

Component name: (E)-Calamenene

Item name: Sep257+ve
Channel name: (E)-Calamenene [+H] : (48.1 PPM) 203.1794

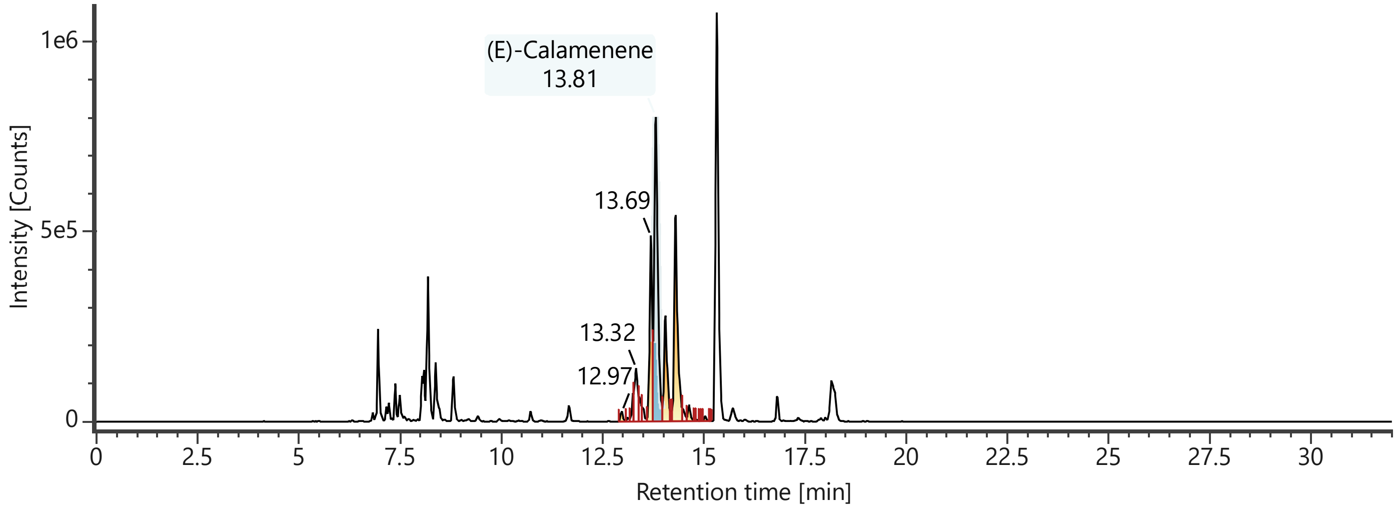

Item name: Sep257+ve
Item description: Mervat253
Channel name: Low energy : Time 13.8173 +/- 0.0237 minutes

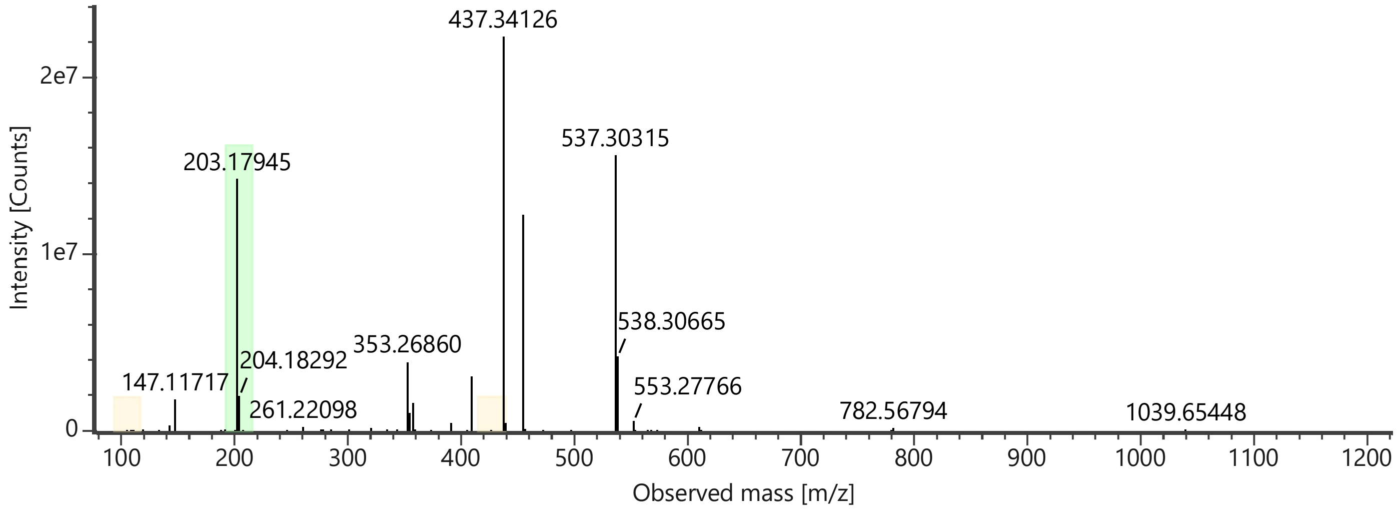

Item name: Sep257+ve
Item description: Mervat253

Channel name: High energy : Time 13.8173 +/- 0.0237 minutes

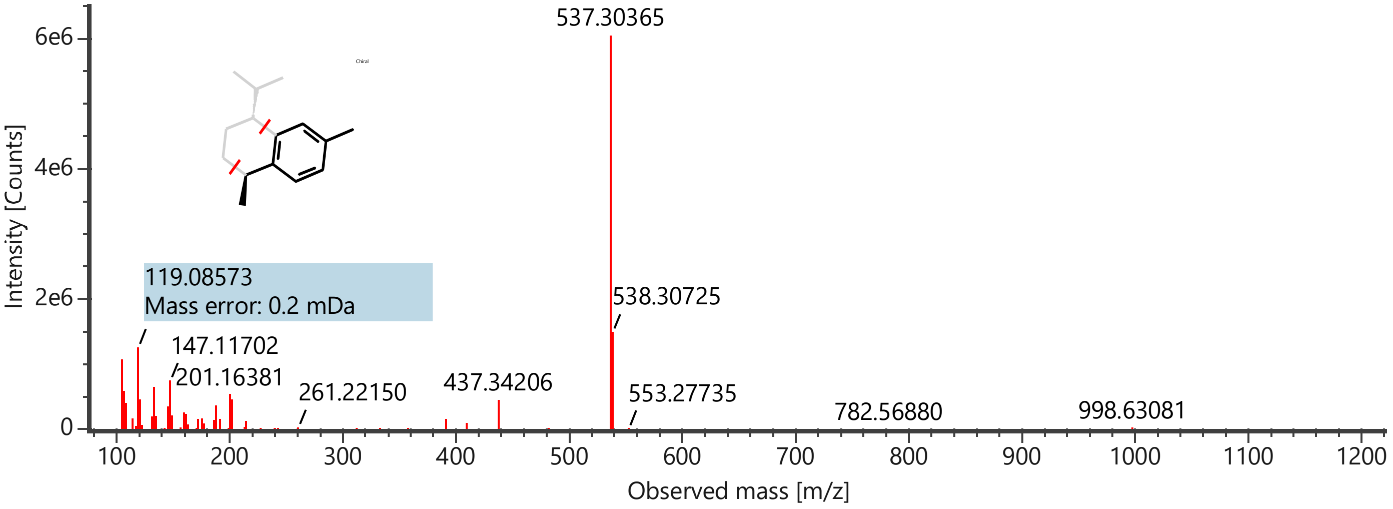

Component name: (E)-Calamenene

Item name: Sep257+ve
Channel name: (E)-Calamenene [+H] : (48.1 PPM) 203.1795

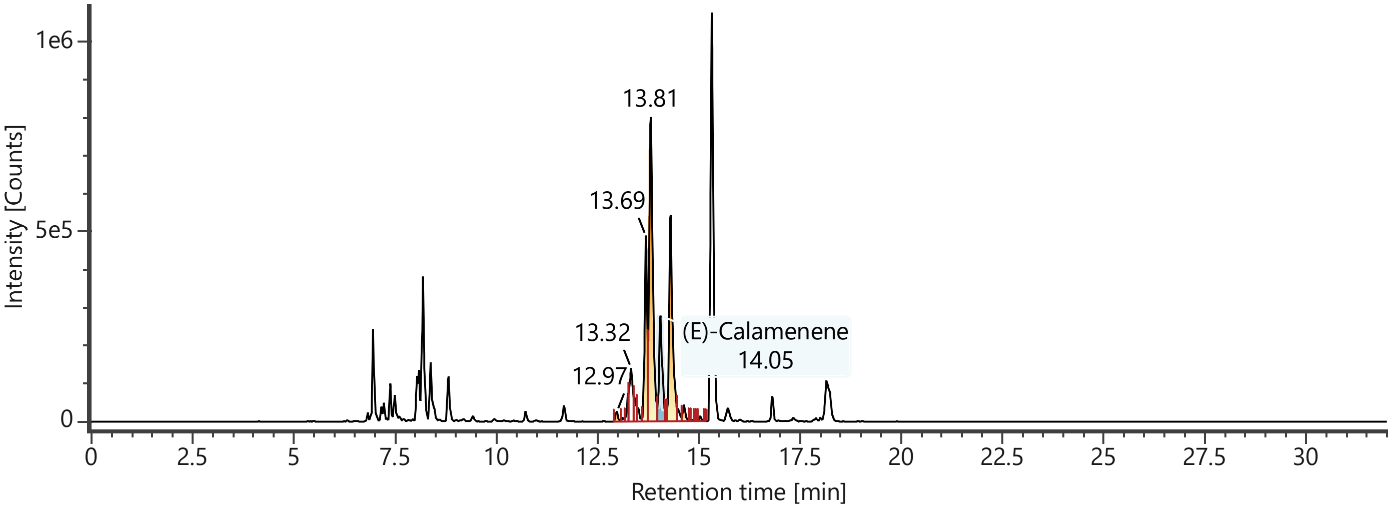

Item name: Sep257+ve
Item description: Mervat253

Channel name: Low energy : Time 14.0567 +/- 0.0237 minutes

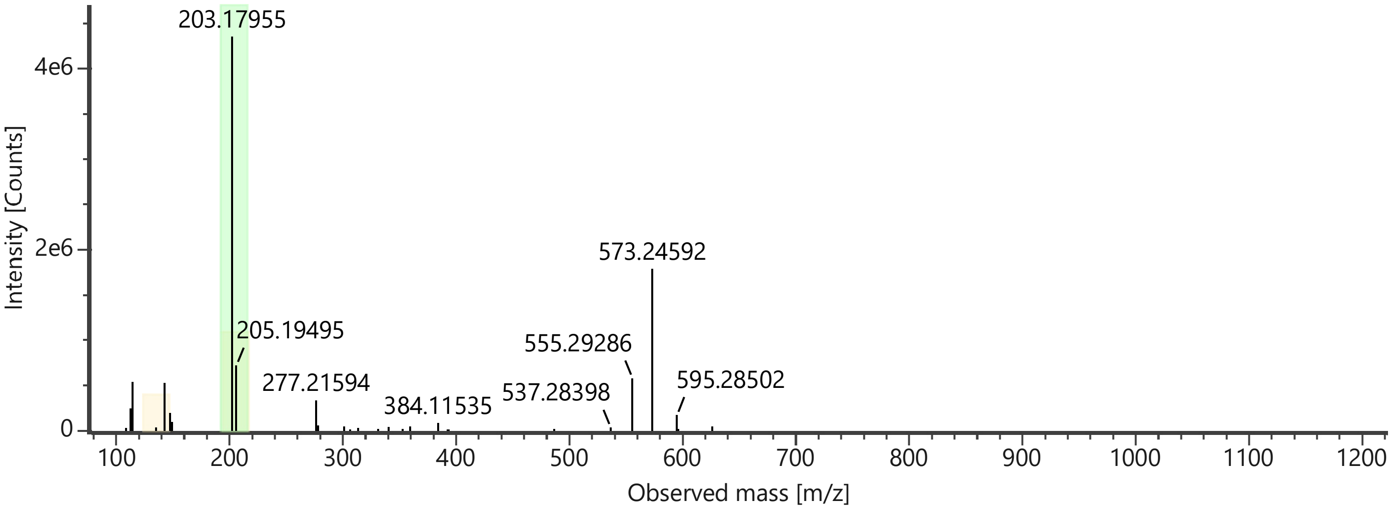

Created by: Ayad, Younan

Created on: Sep 18, 2025

Item name: Lamiaceae Family +ve mode

Created time: 13:52:10 Egypt Standard Time

Item name: Sep257+ve
Item description: Mervat253

Channel name: High energy : Time 14.0567 +/- 0.0237 minutes

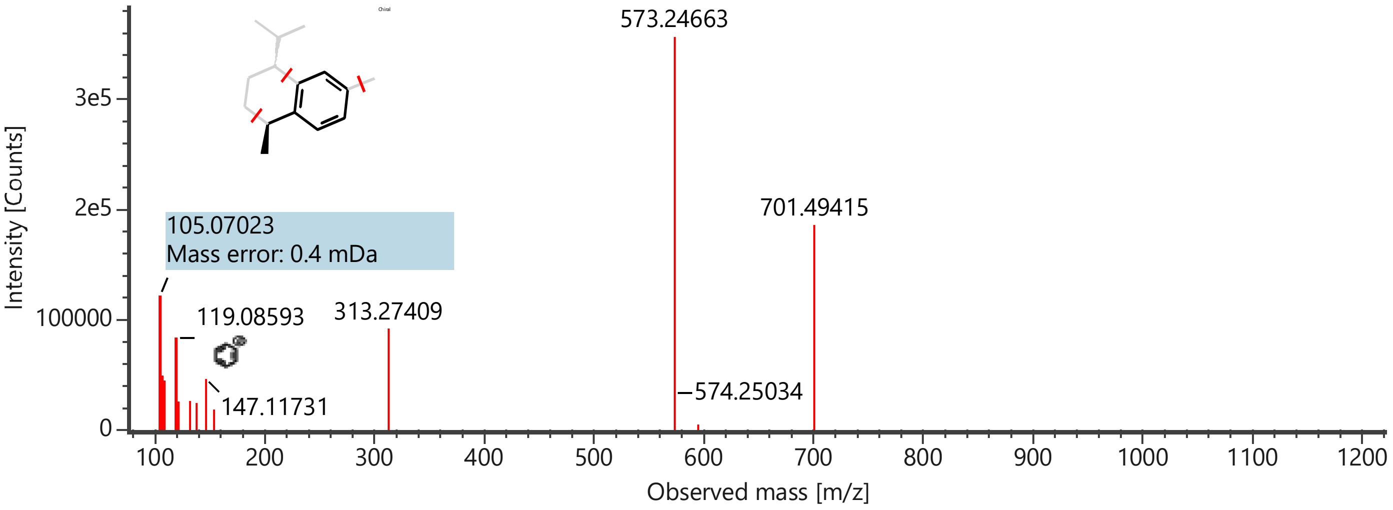

Component name: (E)-Calamenene

Item name: Sep257+ve
Channel name: (E)-Calamenene [+H] : (48.1 PPM) 203.1795

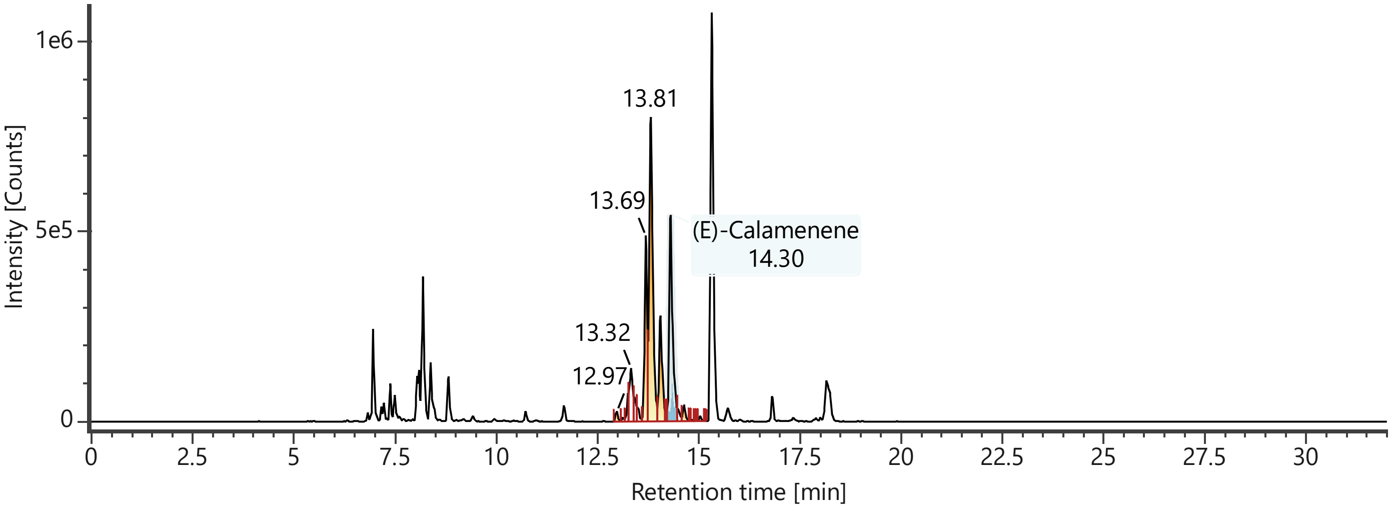

Item name: Sep257+ve
Item description: Mervat253

Channel name: Low energy : Time 14.3047 +/- 0.0237 minutes

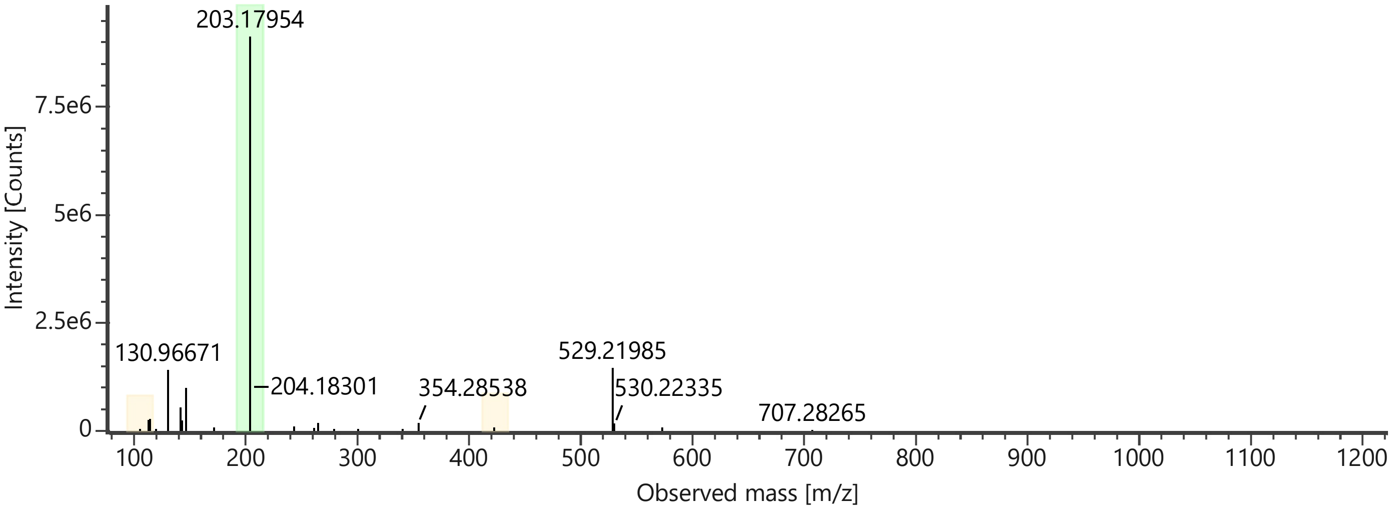

Created by: Ayad, Younan

Created on: Sep 18, 2025

Item name: Lamiaceae Family +ve mode

Created time: 13:52:10 Egypt Standard Time

Item name: Sep257+ve
Item description: Mervat253

Channel name: High energy : Time 14.3047 +/- 0.0237 minutes

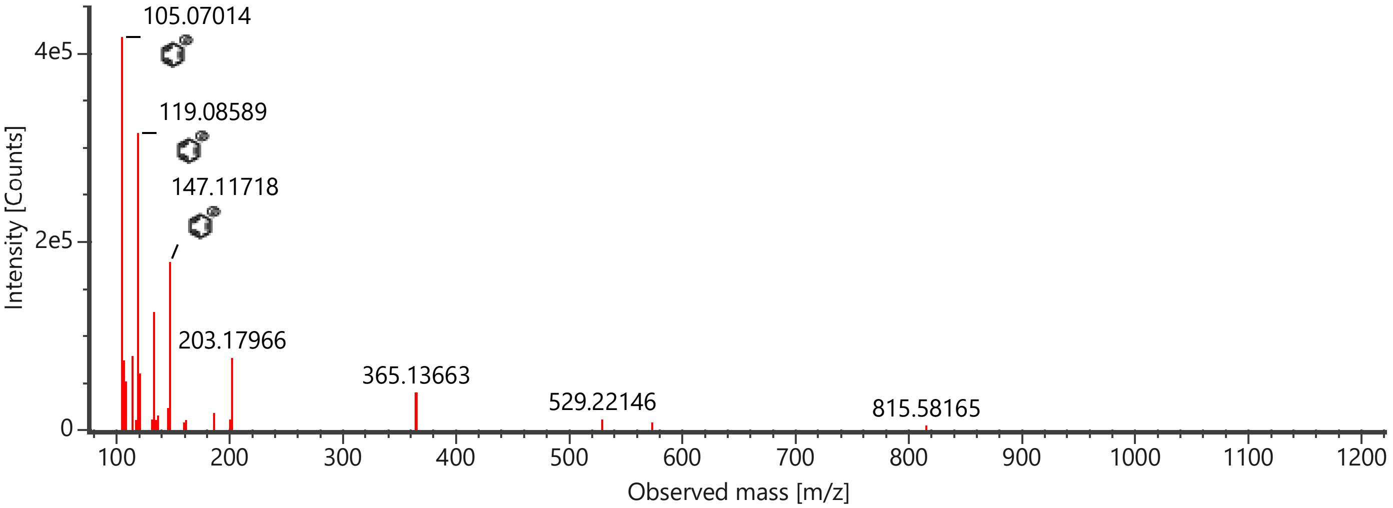

Component name: (E)-Calamenene

Item name: Sep257+ve
Channel name: (E)-Calamenene [+H] : (48.1 PPM) 203.1795

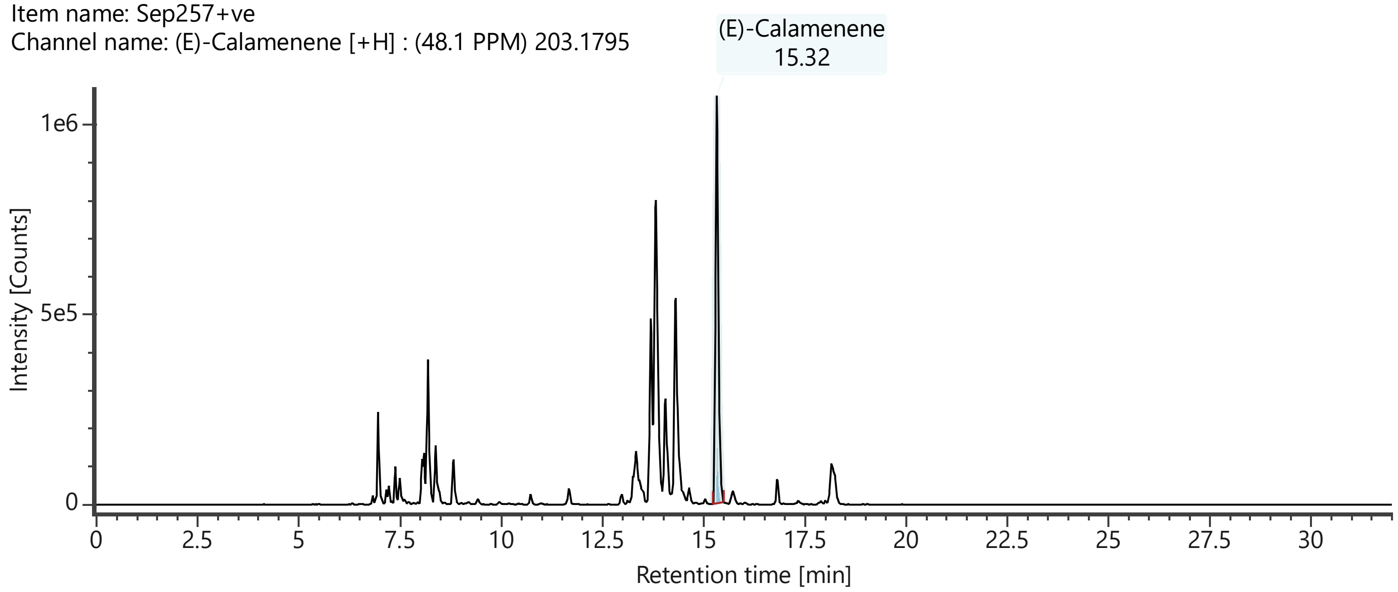

Item name: Sep257+ve
Item description: Mervat253

Channel name: Low energy : Time 15.3194 +/- 0.0237 minutes

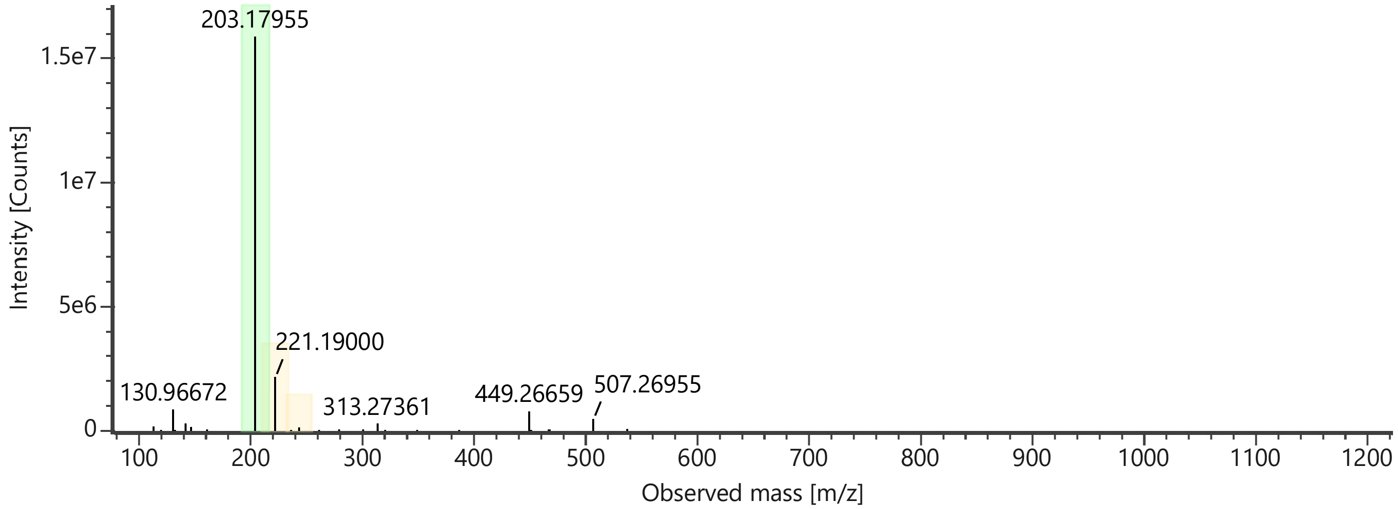

Created by: Ayad, Younan

Created on: Sep 18, 2025

Item name: Lamiaceae Family +ve mode

Created time: 13:52:10 Egypt Standard Time

Item name: Sep257+ve
Item description: Mervat253

Channel name: High energy : Time 15.3194 +/- 0.0237 minutes

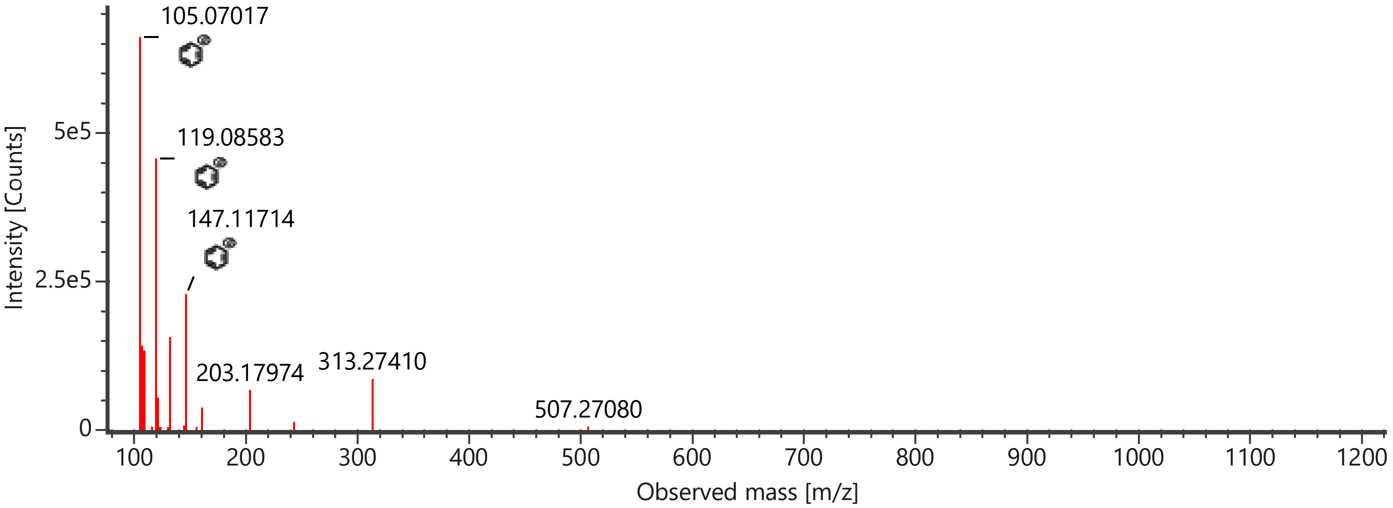

Component name: (1aR,4aS,7R,7aR,7bR)-1,1,7-Trimethyl-4-methylidenedecahydro-1H-cyclopropa(e) azulen-7-ol

Item name: Sep257+ve
Channel name: (1aR,4aS,7R,7aR,7bR)-1,1,7-Trimethyl-4-methylidenedecahydro-1H-cyclopropa(e)azulen-7-ol [+H] : (48.1 PPM) 221.1900

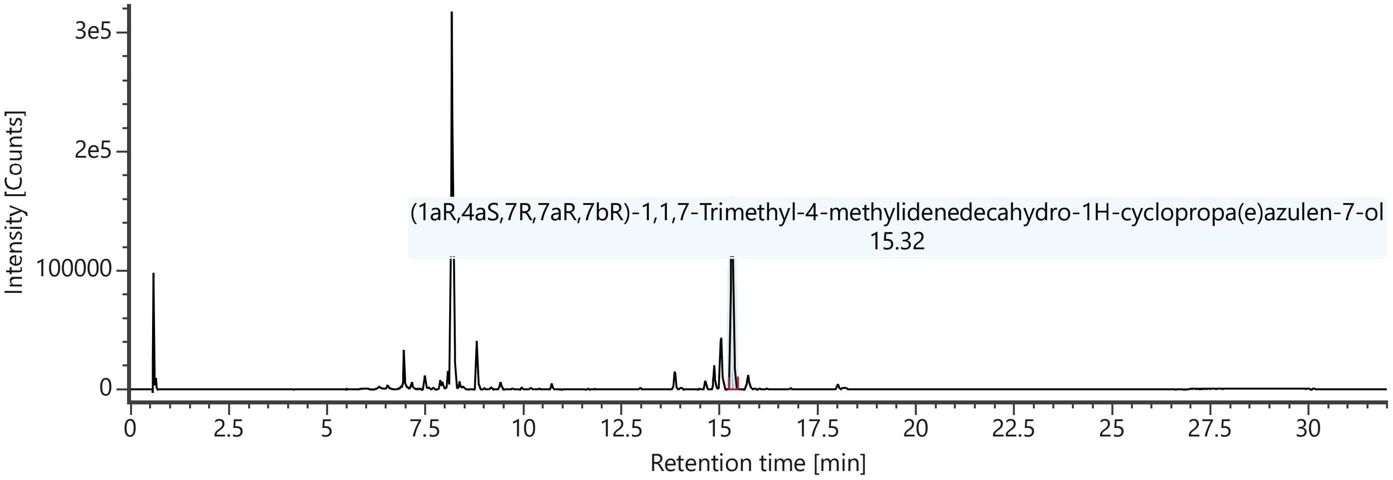

Item name: Sep257+ve
Item description: Mervat253
Channel name: Low energy : Time 15.3198 +/- 0.0237 minutes

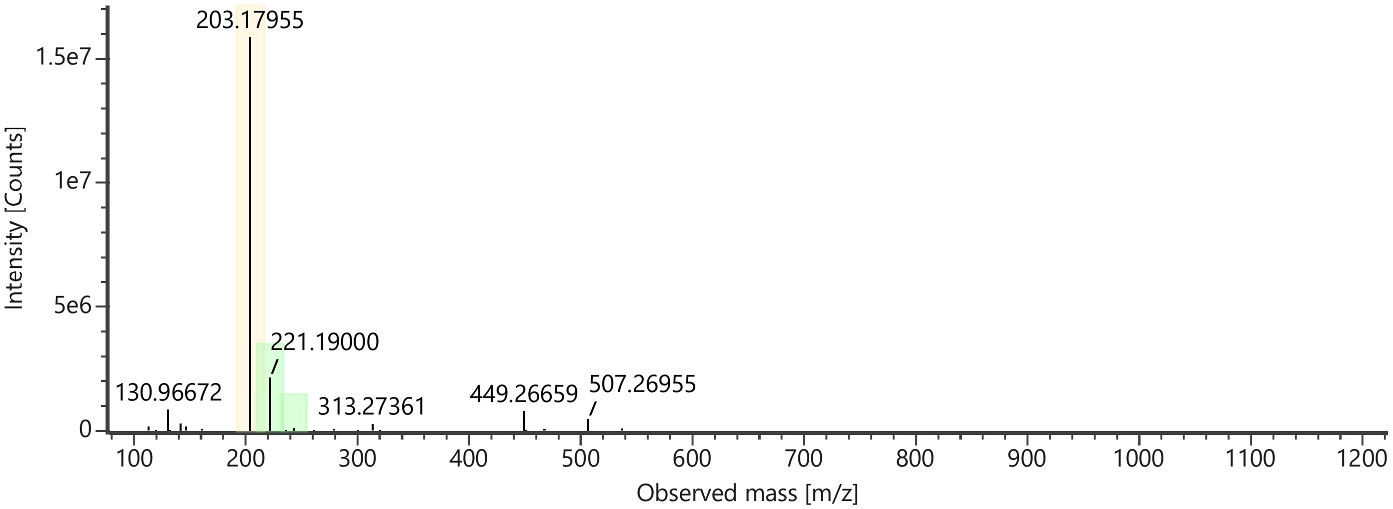

Item name: Sep257+ve
Item description: Mervat253

Channel name: High energy : Time 15.3198 +/- 0.0237 minutes

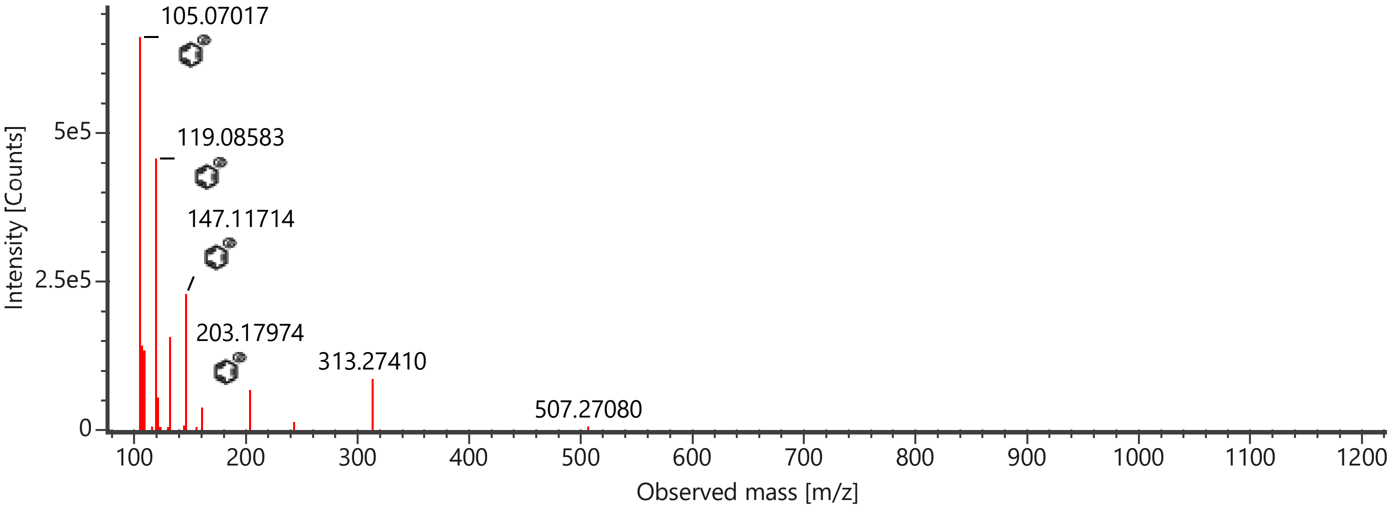

Component name: beta-Bourbonene

Item name: Sep257+ve
Channel name: beta-Bourbonene [+H] : (48.1 PPM) 205.1952

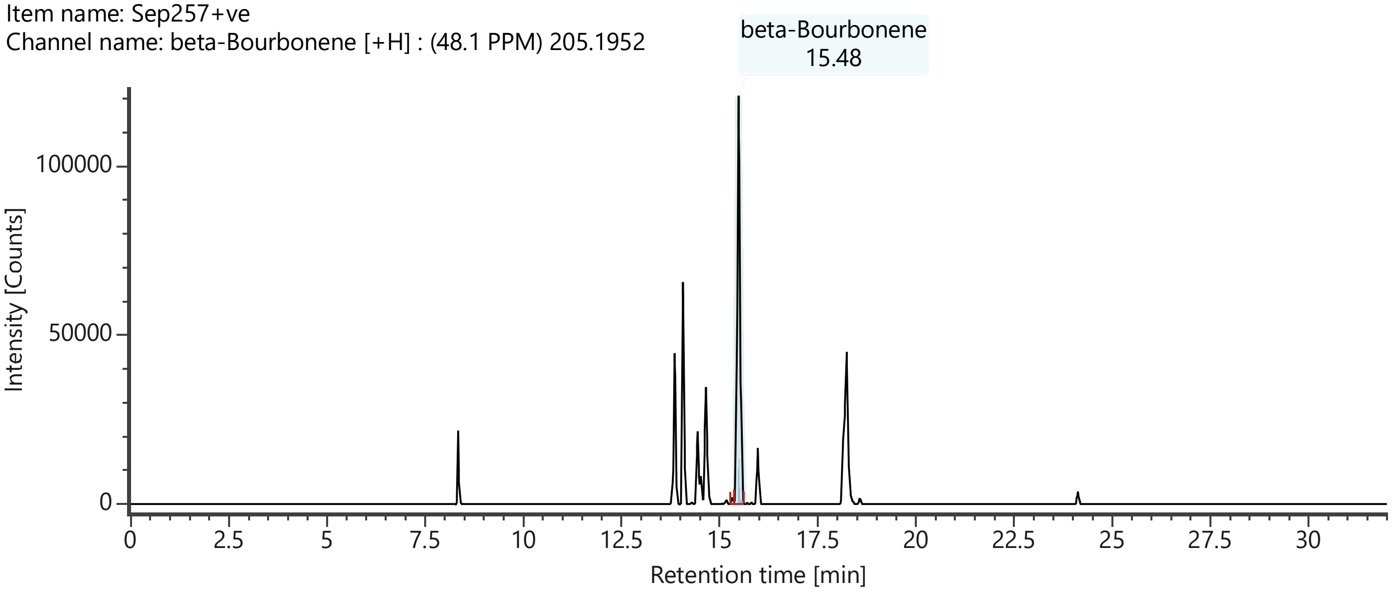

Item name: Sep257+ve
Item description: Mervat253
Channel name: Low energy : Time 15.4799 +/- 0.0237 minutes

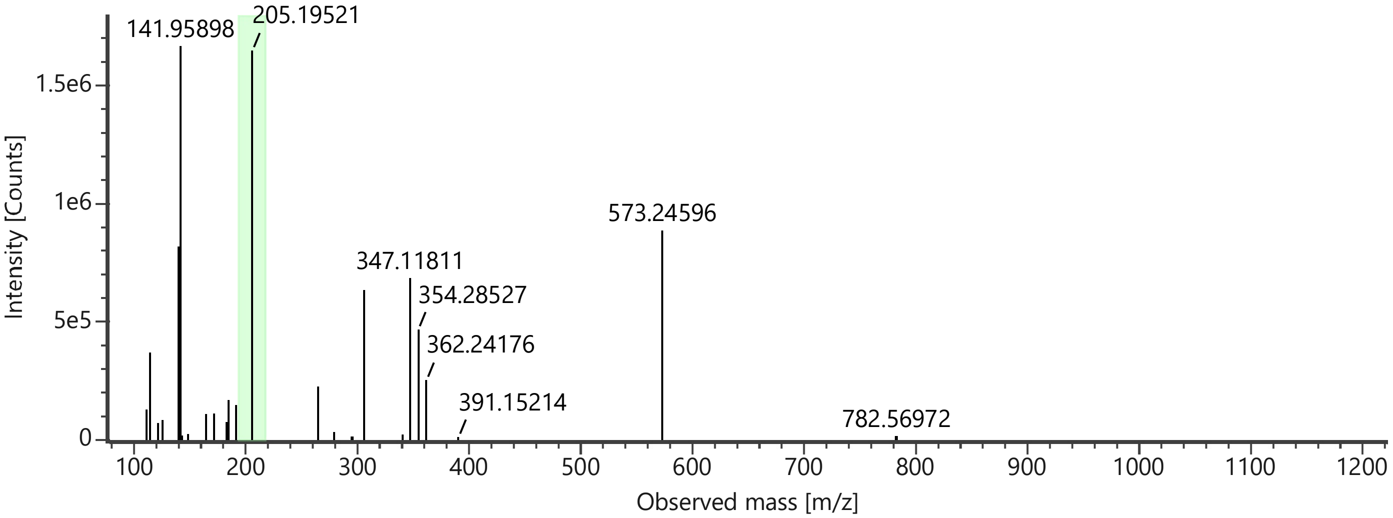

Created by: Ayad, Younan

Created on: Sep 18, 2025

Item name: Lamiaceae Family +ve mode

Created time: 13:52:10 Egypt Standard Time

Item name: Sep257+ve
Item description: Mervat253

Channel name: High energy : Time 15.4799 +/- 0.0237 minutes

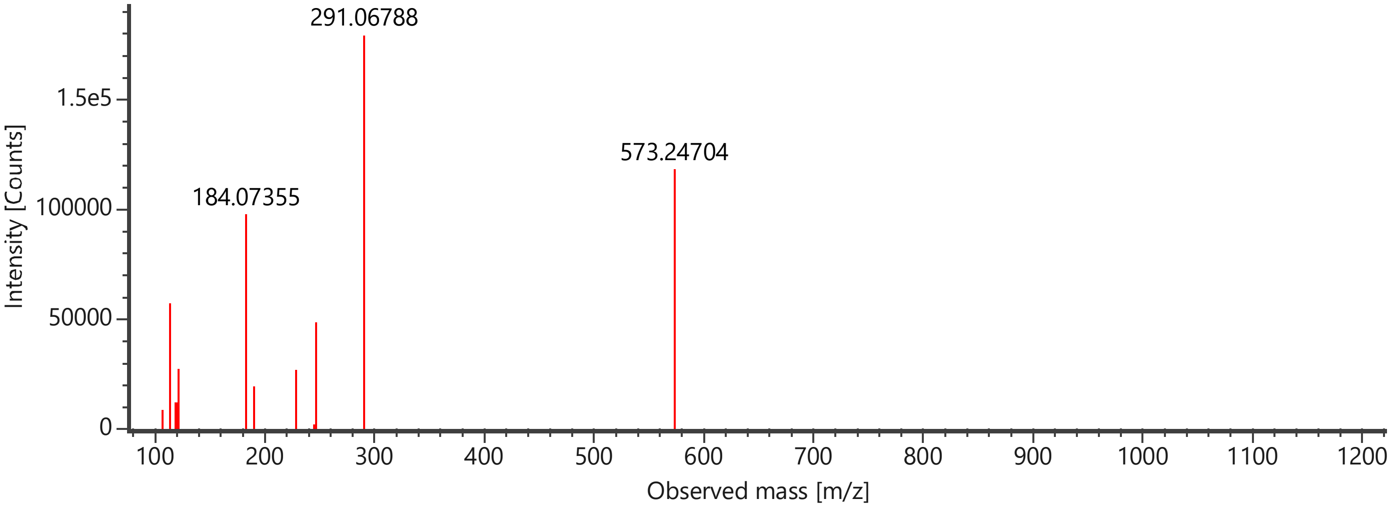

Component name: 7alpha-Hydroxycampesterol

Item name: Sep257+ve
Channel name: 7alpha-Hydroxycampesterol [+Na] : (48.1 PPM) 439.3565

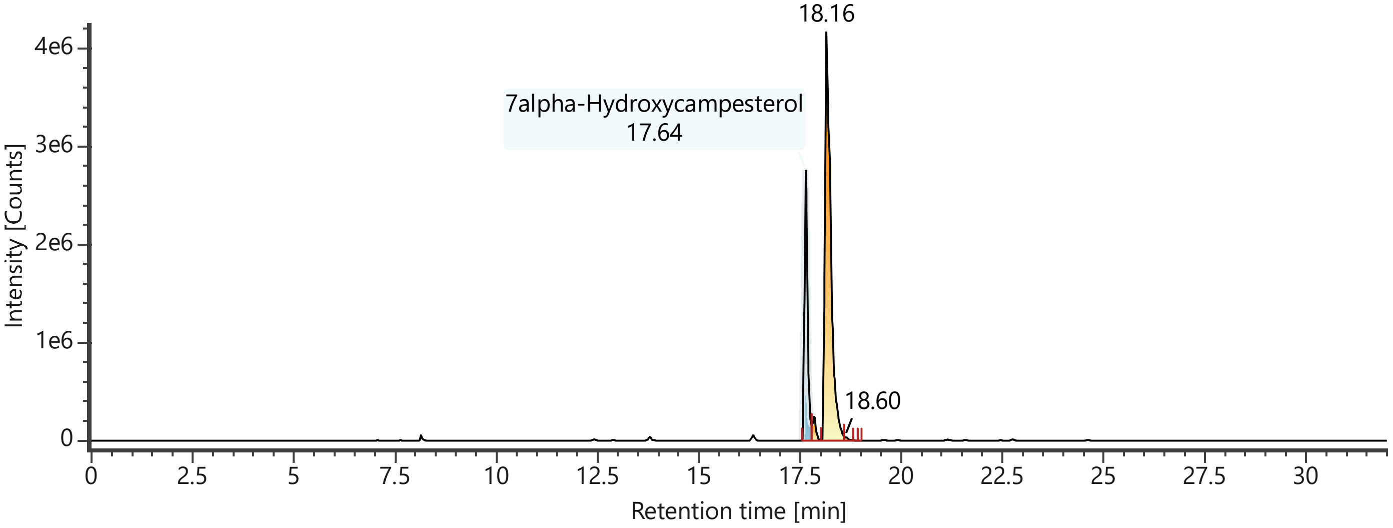

Item name: Sep257+ve
Item description: Mervat253
Channel name: Low energy : Time 17.6448 +/- 0.0237 minutes

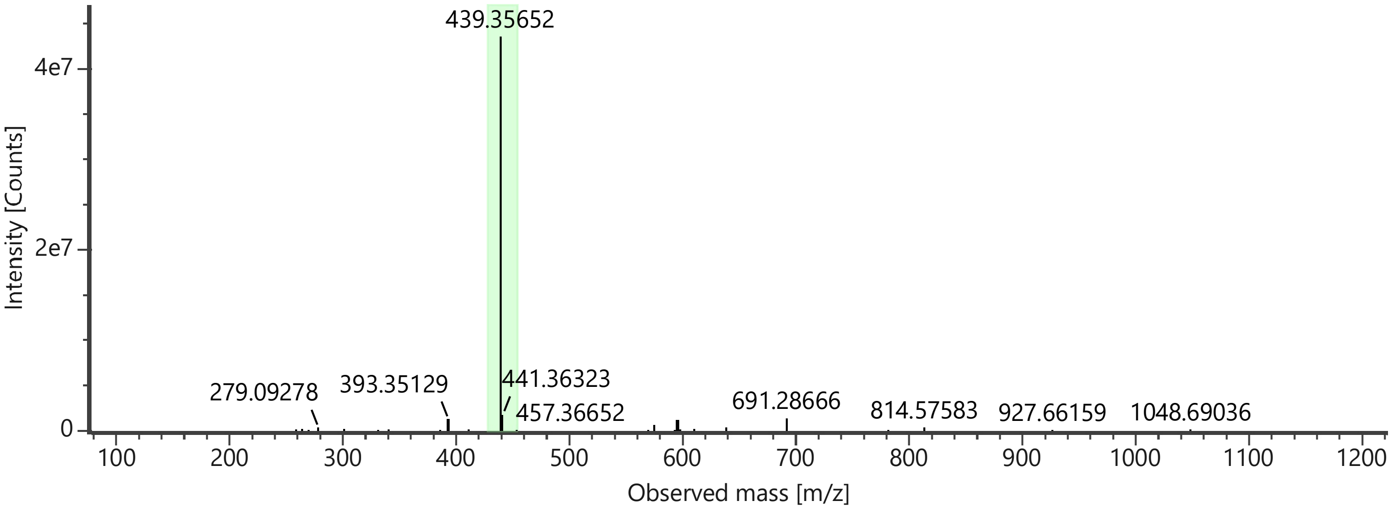

Item name: Sep257+ve
Item description: Mervat253

Channel name: High energy : Time 17.6448 +/- 0.0237 minutes

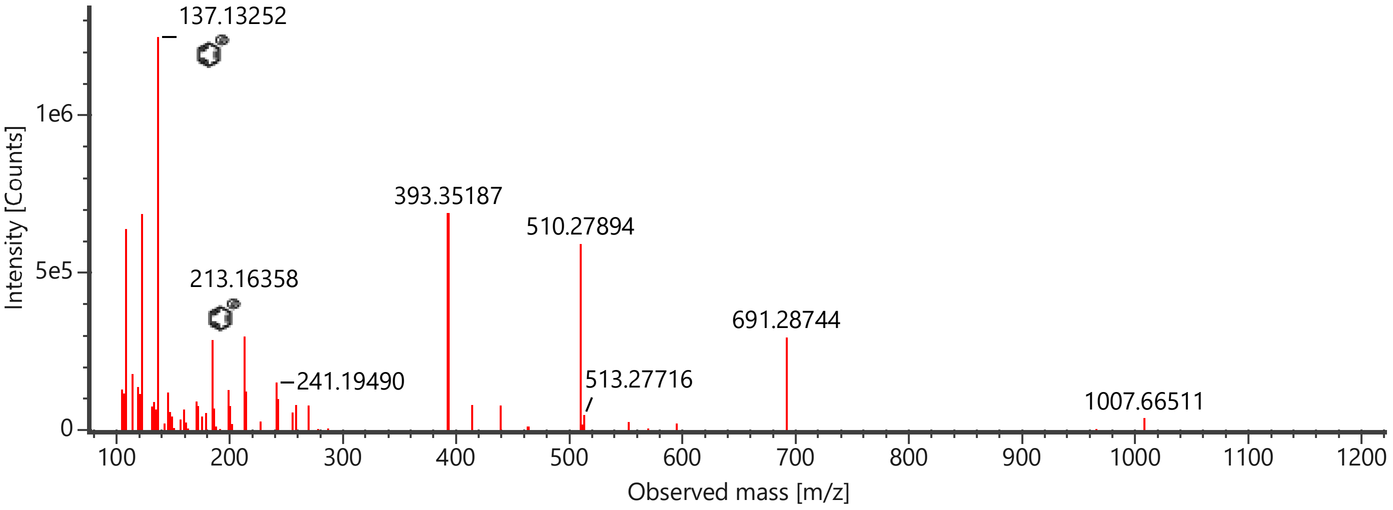

Component name: 7alpha-Hydroxycampesterol

Item name: Sep257+ve
Channel name: 7alpha-Hydroxycampesterol [+Na] : (48.1 PPM) 439.3567

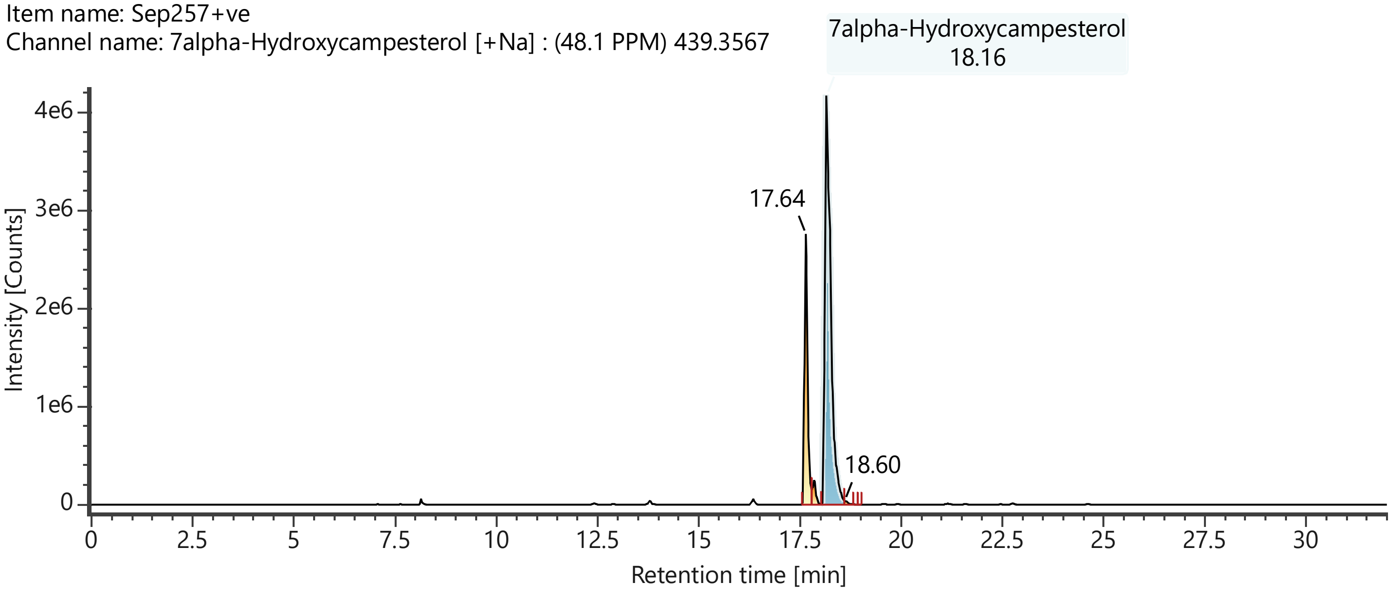

Item name: Sep257+ve
Item description: Mervat253
Channel name: Low energy : Time 18.1549 +/- 0.0237 minutes

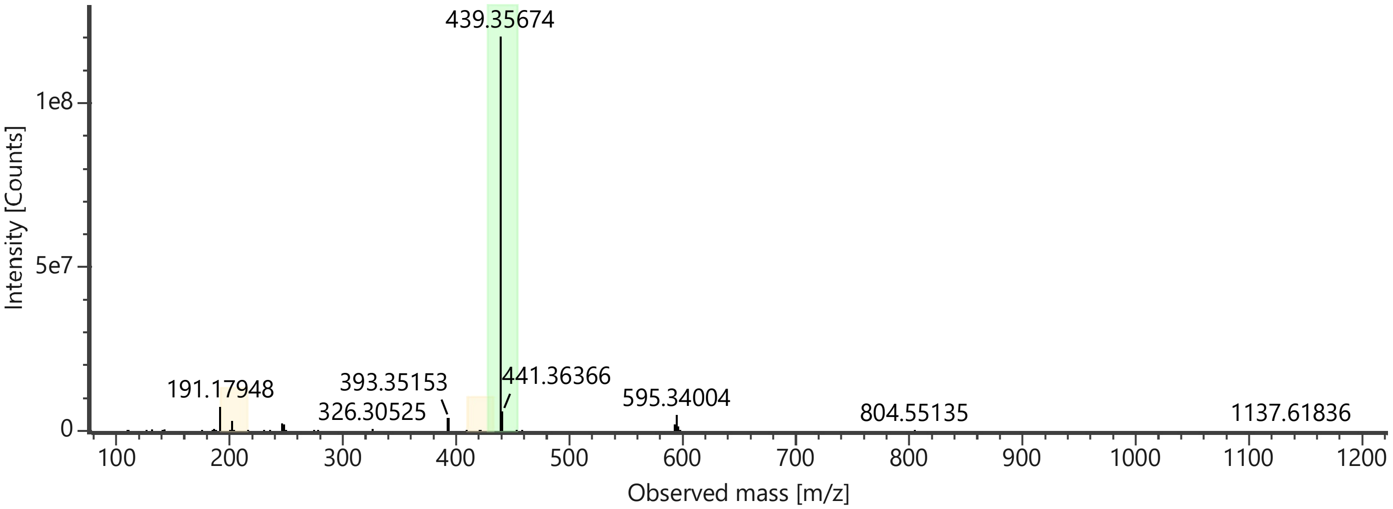

Created by: Ayad, Younan

Created on: Sep 18, 2025

Item name: Lamiaceae Family +ve mode

Created time: 13:52:10 Egypt Standard Time

Item name: Sep257+ve
Item description: Mervat253

Channel name: High energy : Time 18.1549 +/- 0.0237 minutes

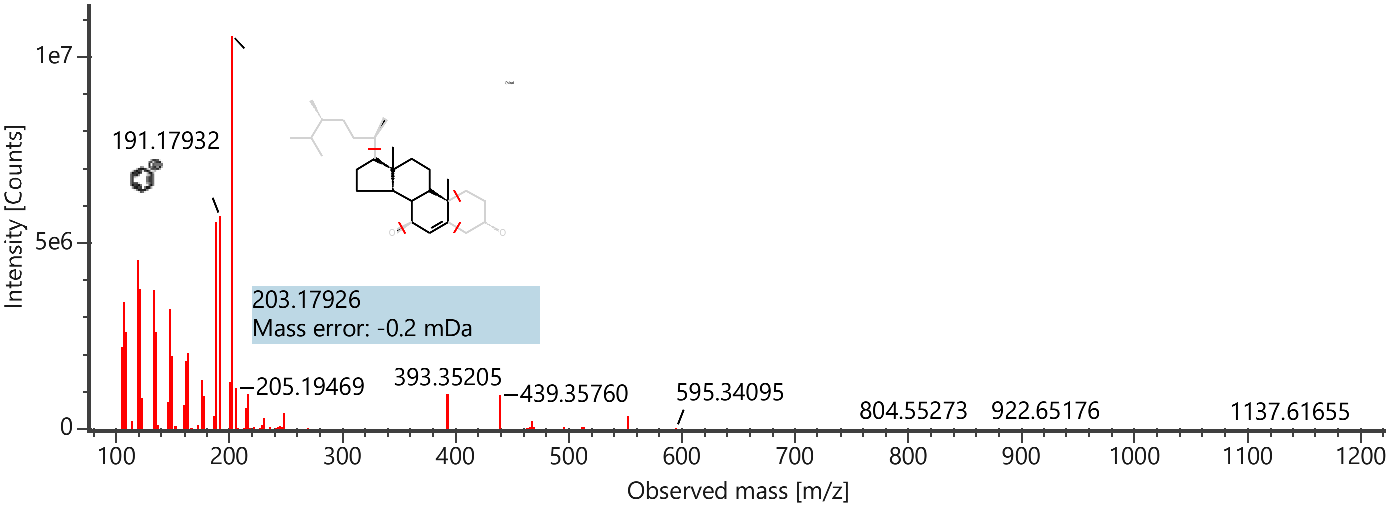

Component name: (E)-Calamenene

Item name: Sep257+ve
Channel name: (E)-Calamenene [+H] : (48.1 PPM) 203.1794

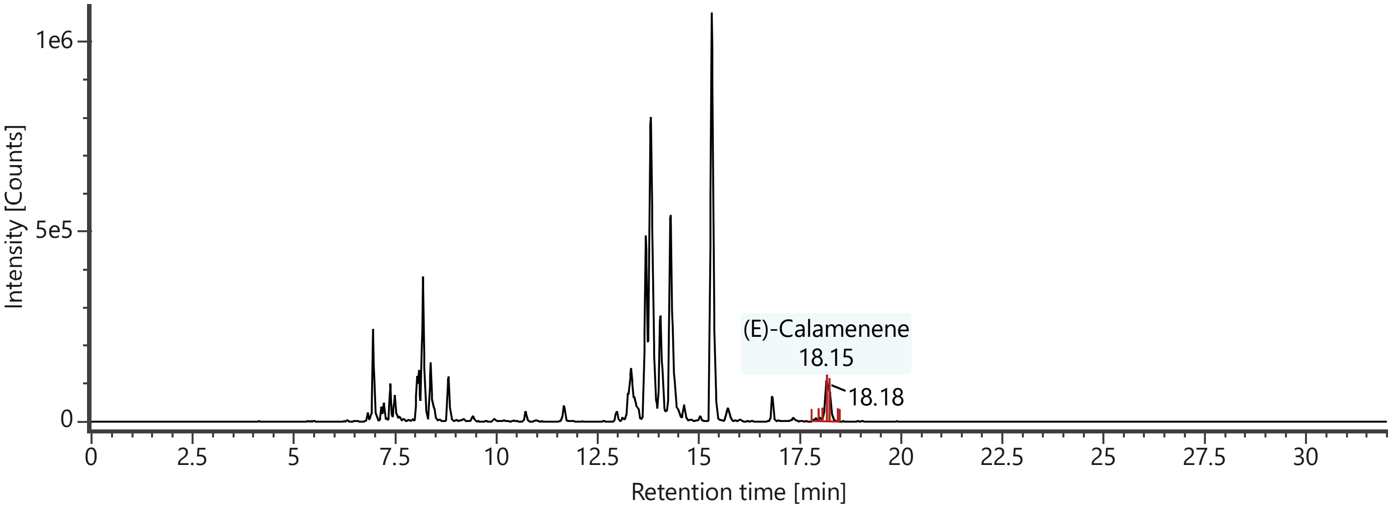

Item name: Sep257+ve
Item description: Mervat253
Channel name: Low energy : Time 18.1560 +/- 0.0237 minutes

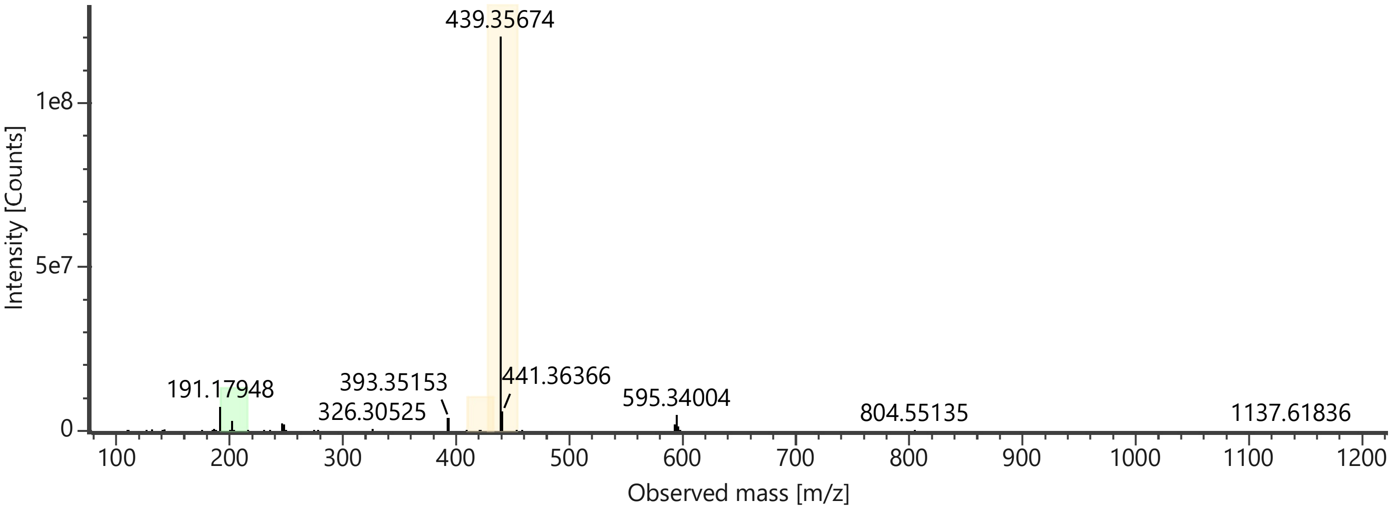

Item name: Sep257+ve
Item description: Mervat253

Channel name: High energy : Time 18.1560 +/- 0.0237 minutes

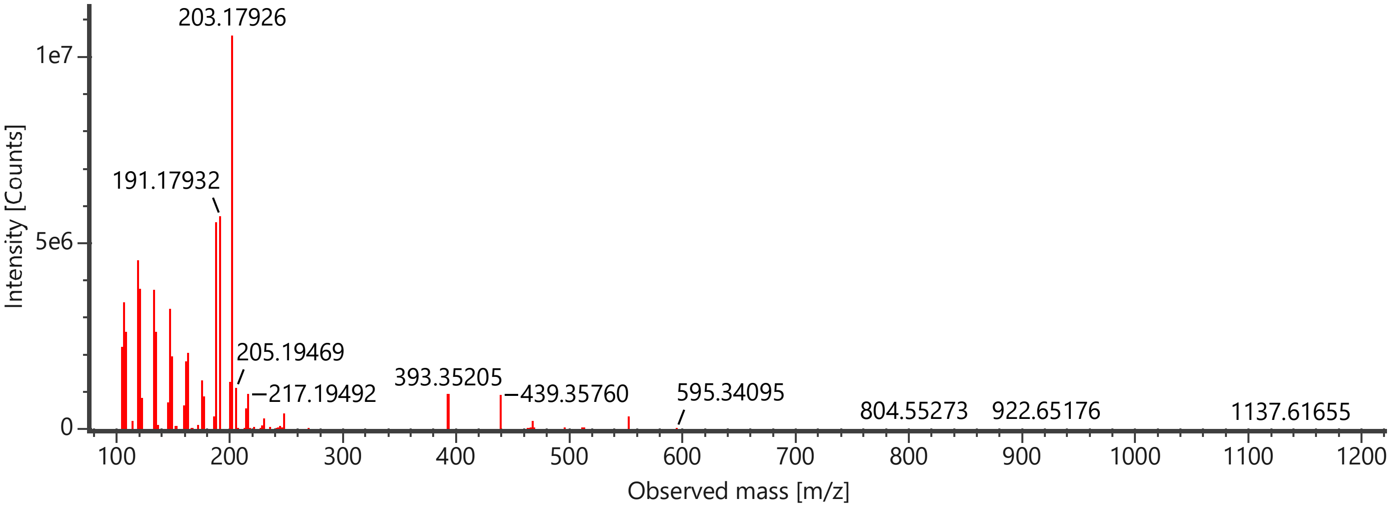

Component name: alpha-Amorphene

Item name: Sep257+ve
Channel name: alpha-Amorphene [+H] : (48.1 PPM) 205.1949

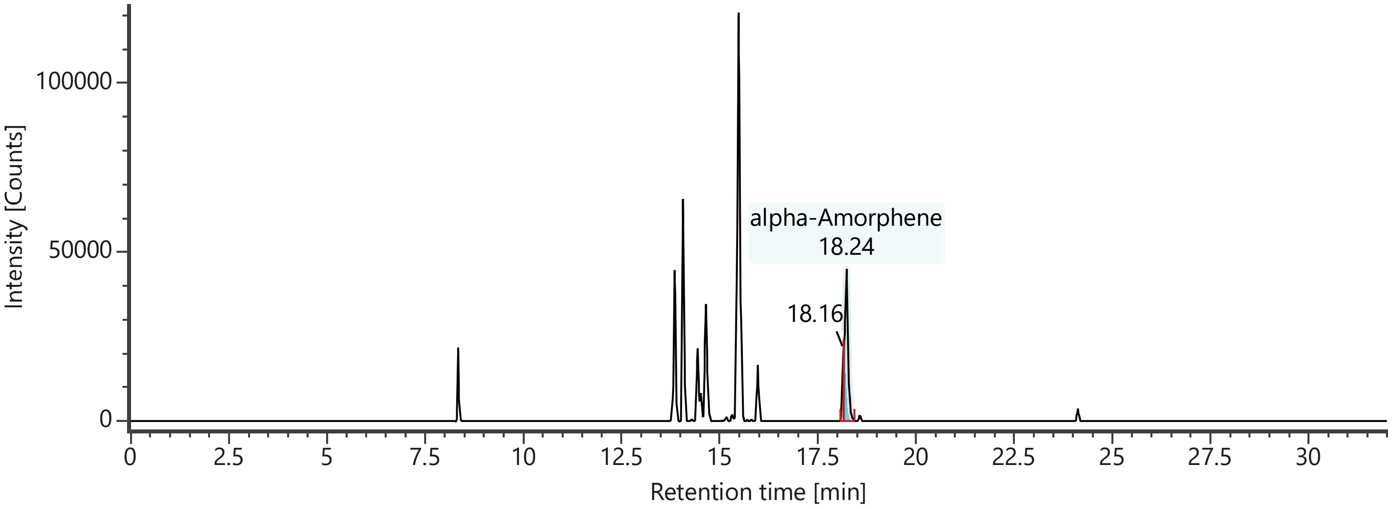

Item name: Sep257+ve
Item description: Mervat253
Channel name: Low energy : Time 18.2343 +/- 0.0237 minutes

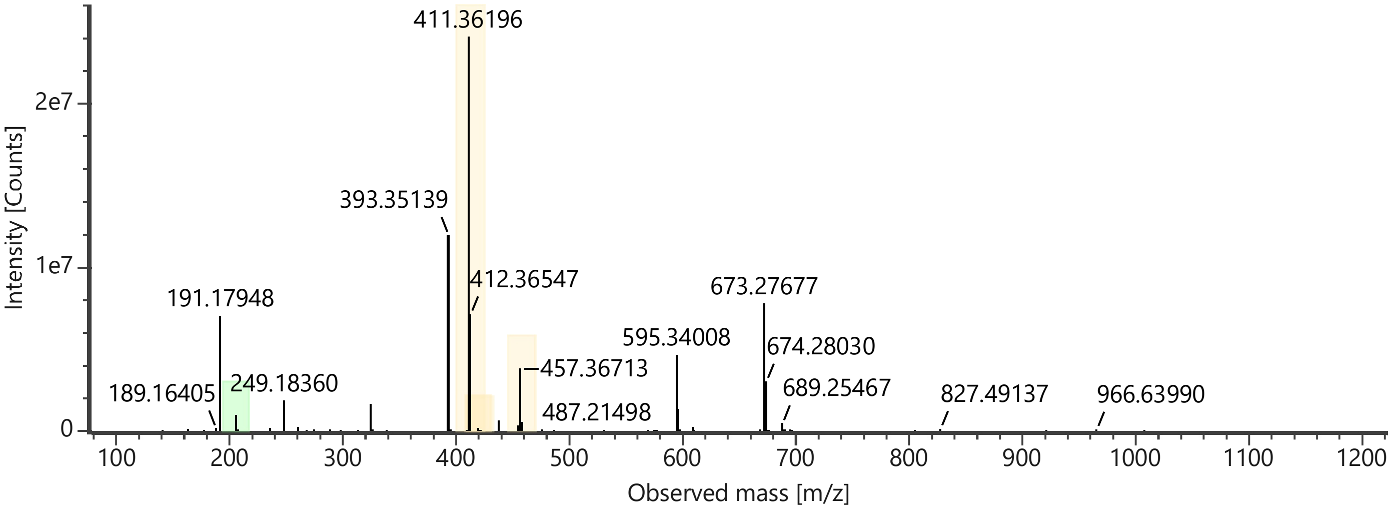

Item name: Lamiaceae Family +ve mode

Created by: Ayad, Younan

Created on: Sep 18, 2025

Created time: 13:52:10 Egypt Standard Time

Item name: Sep257+ve

Item description: Mervat253

Channel name: High energy : Time 18.2343 +/- 0.0237 minutes

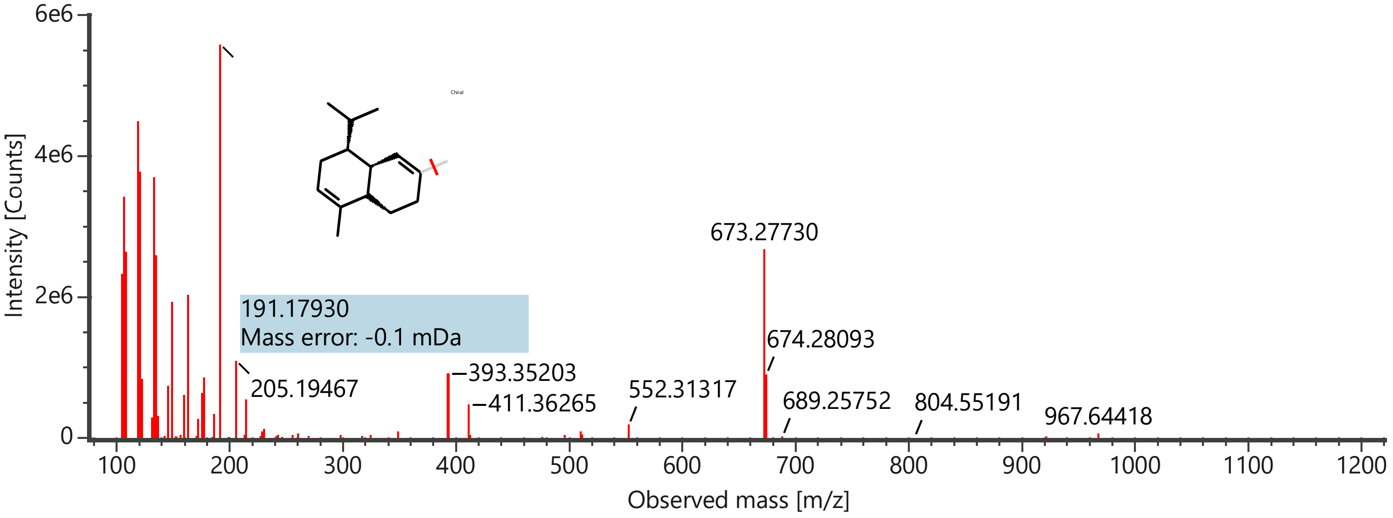

Component name: 3-Epioleanolic acid

Item name: Sep257+ve
Channel name: 3-Epioleanolic acid [+H] : (48.1 PPM) 457.3671

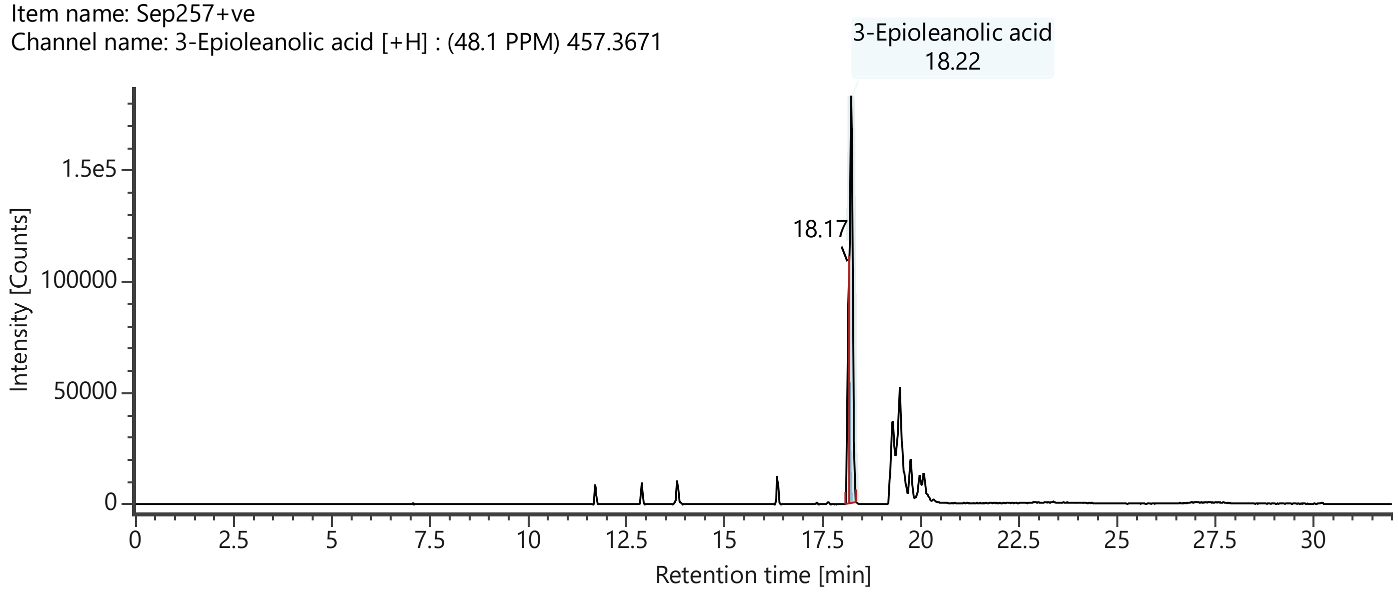

Item name: Sep257+ve
Item description: Mervat253
Channel name: Low energy : Time 18.2344 +/- 0.0237 minutes

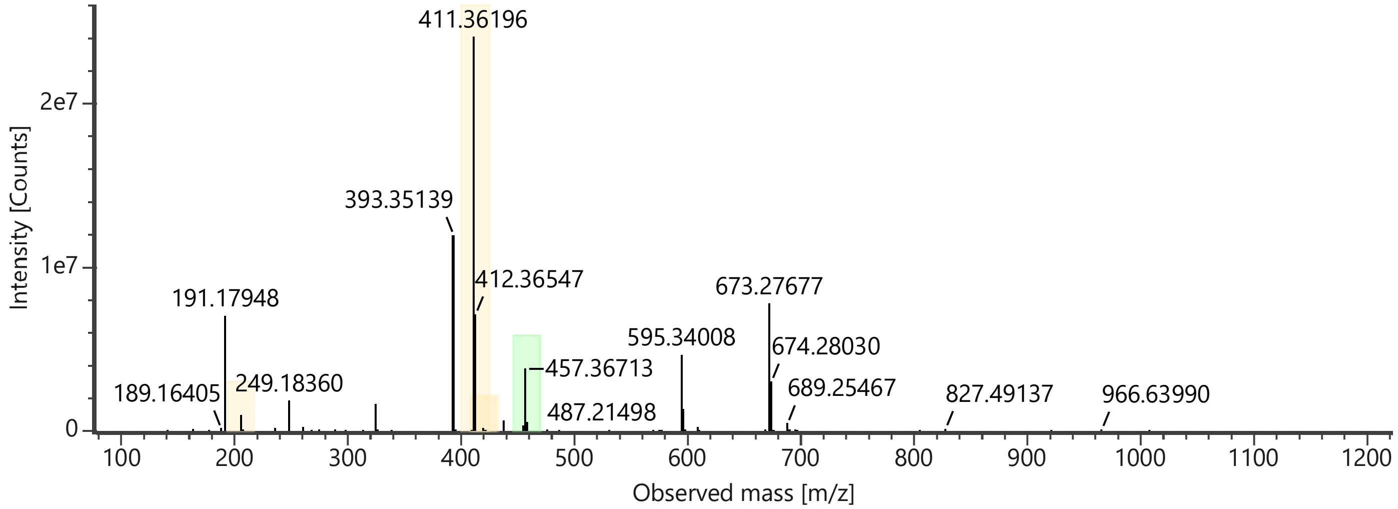

Item name: Sep257+ve
Item description: Mervat253

Channel name: High energy : Time 18.2344 +/- 0.0237 minutes

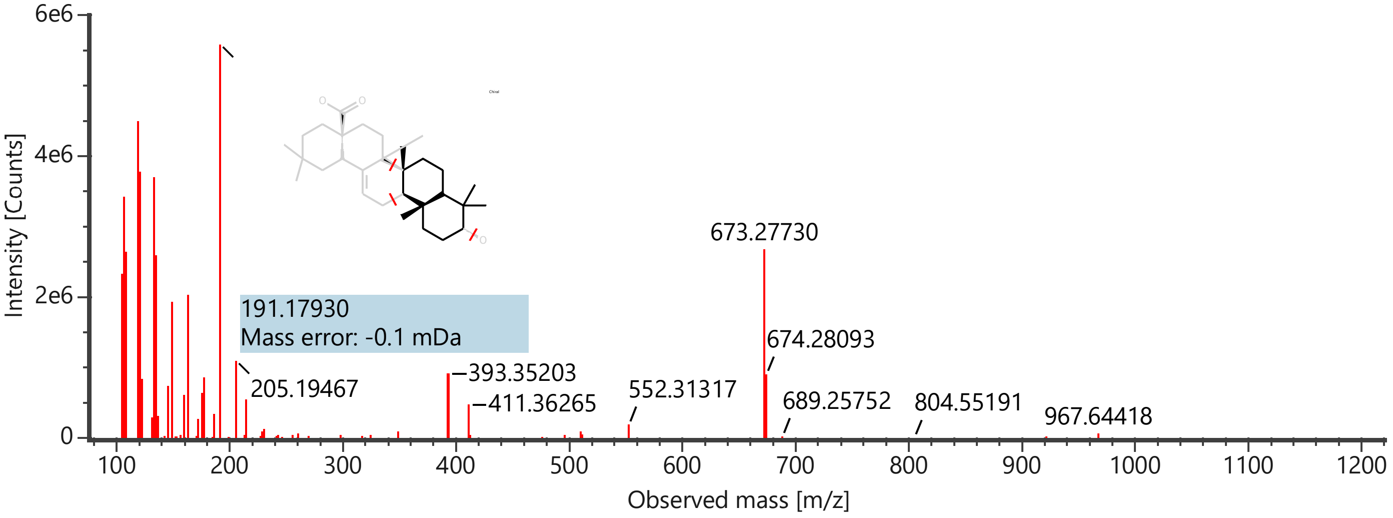

Component name: 25-Dehydrochondrillasterol

Item name: Sep257+ve
Channel name: 25-Dehydrochondrillasterol [+H] : (48.1 PPM) 411.3620

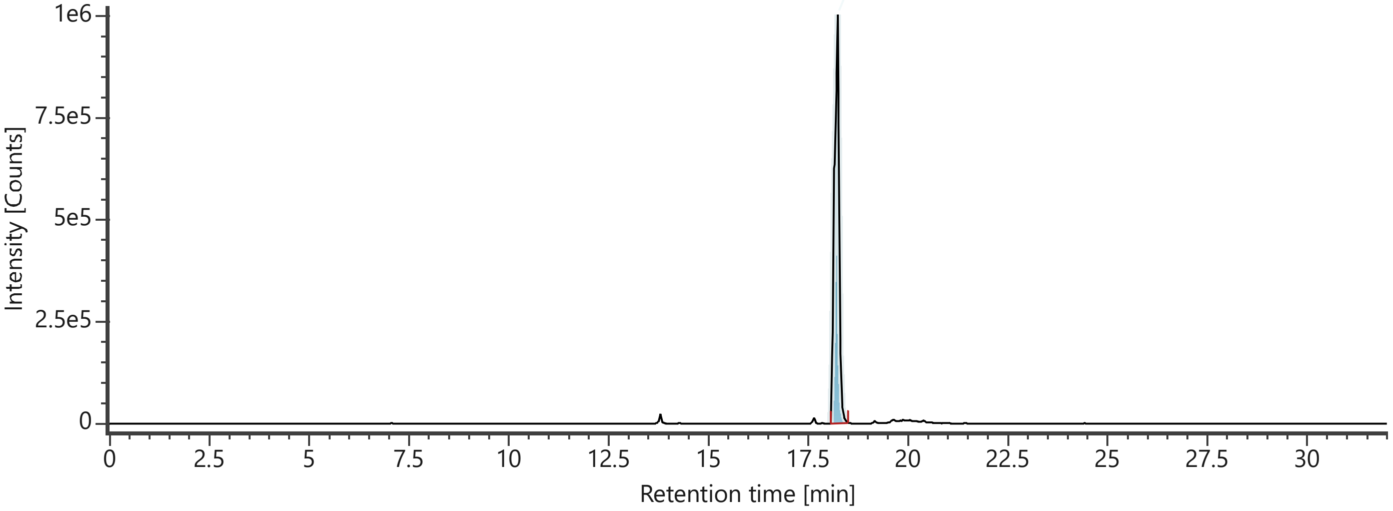

Item name: Sep257+ve
Item description: Mervat253
Channel name: Low energy : Time 18.2345 +/- 0.0237 minutes

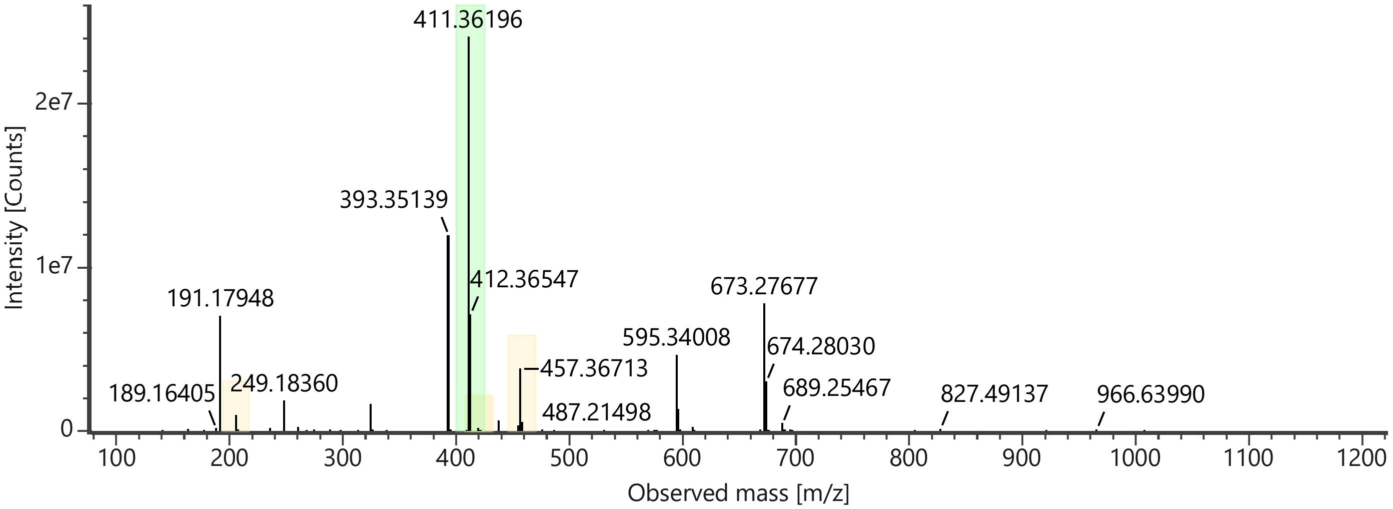

Created by: Ayad, Younan

Created on: Sep 18, 2025

Item name: Lamiaceae Family +ve mode

Created time: 13:52:10 Egypt Standard Time

Item name: Sep257+ve

Item description: Mervat253

Channel name: High energy : Time 18.2345 +/- 0.0237 minutes

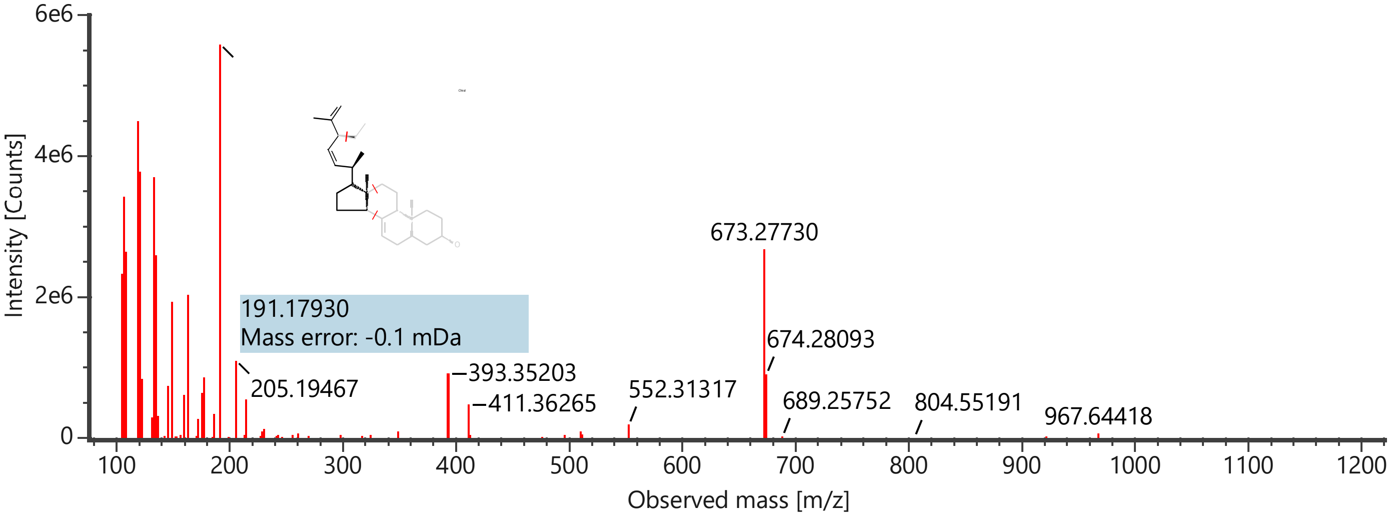

Component name: α-Spinasterol

Item name: Sep257+ve
Channel name: α-Spinasterol [+H] : (48.1 PPM) 413.3779

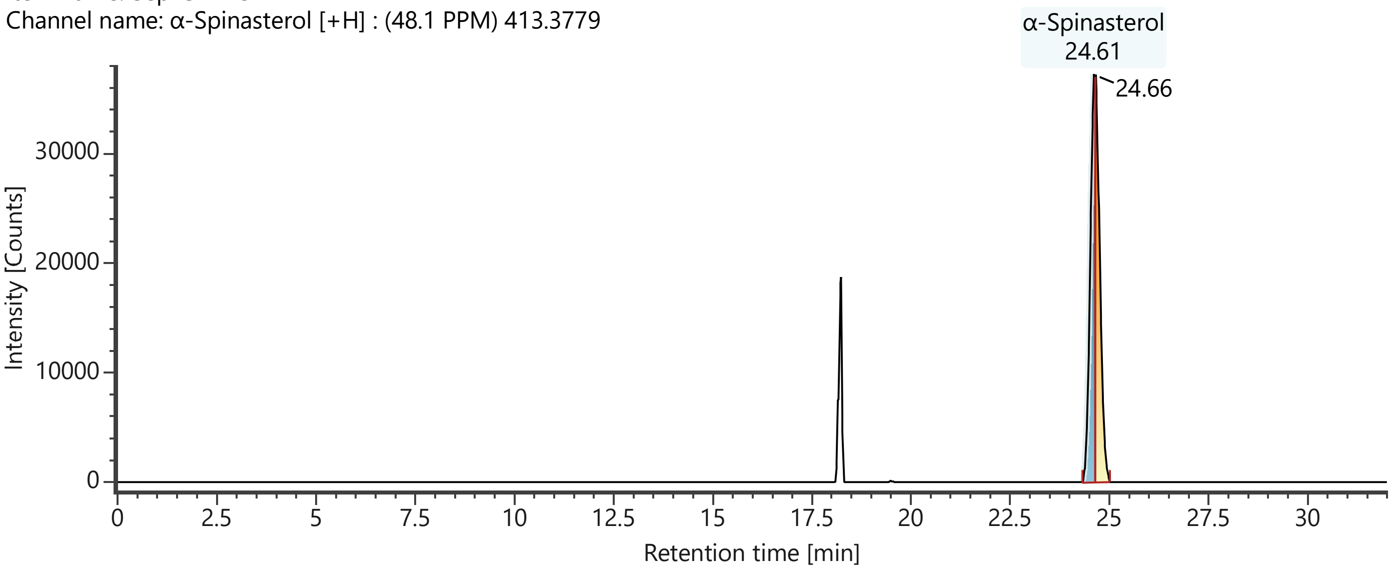

Item name: Sep257+ve
Item description: Mervat253
Channel name: Low energy : Time 24.6148 +/- 0.0237 minutes

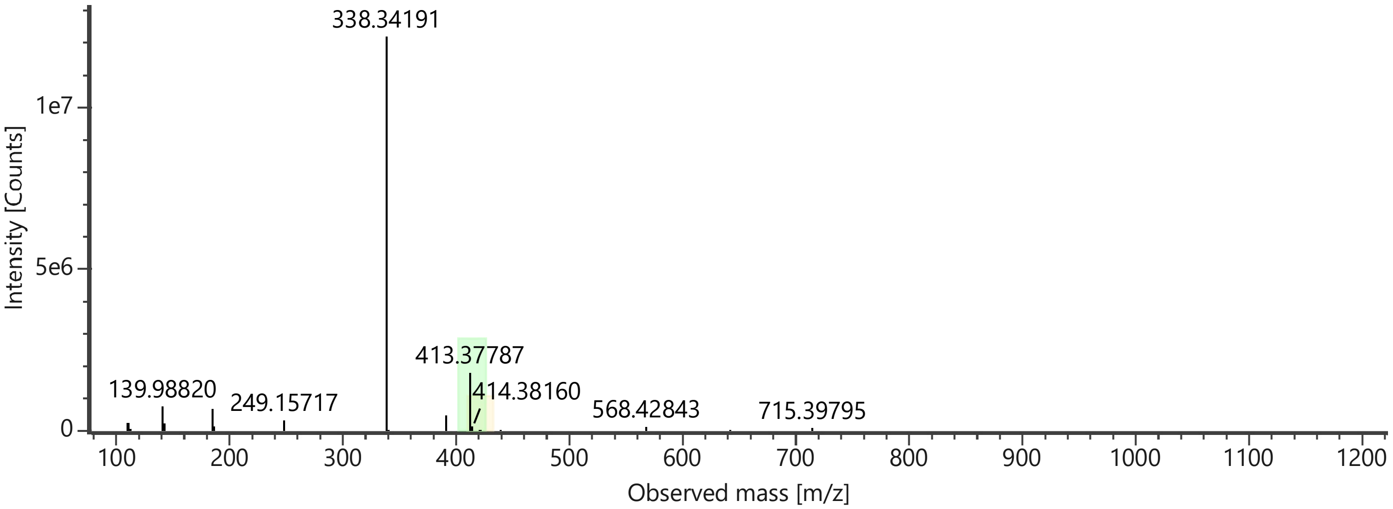

Item name: Sep257+ve
Item description: Mervat253

Channel name: High energy : Time 24.6148 +/- 0.0237 minutes

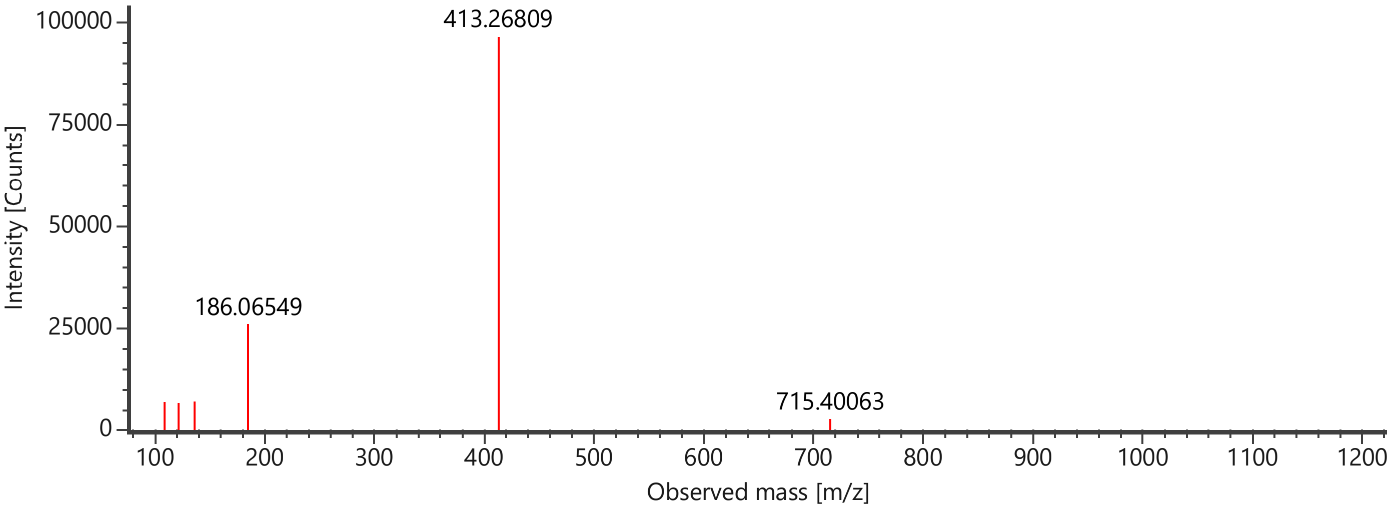

Component name: 5alpha,6beta-Dihydroxydaucosterol

Item name: Sep257+ve
Channel name: 5alpha,6beta-Dihydroxydaucosterol [+H] : (48.1 PPM) 611.4538

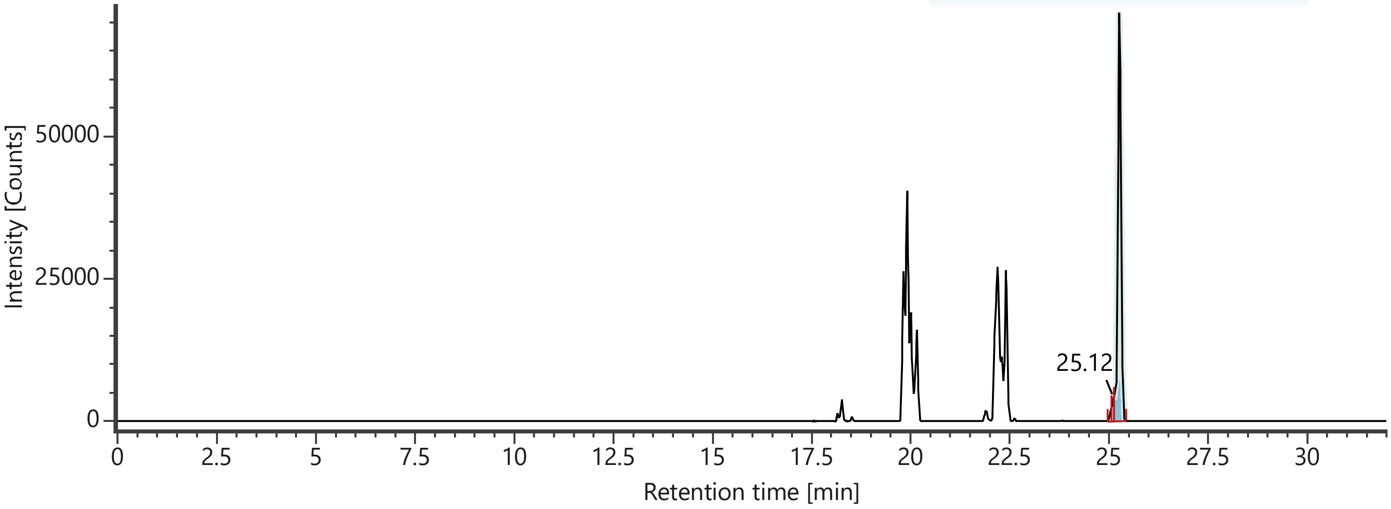

Item name: Sep257+ve
Item description: Mervat253
Channel name: Low energy : Time 25.2638 +/- 0.0237 minutes

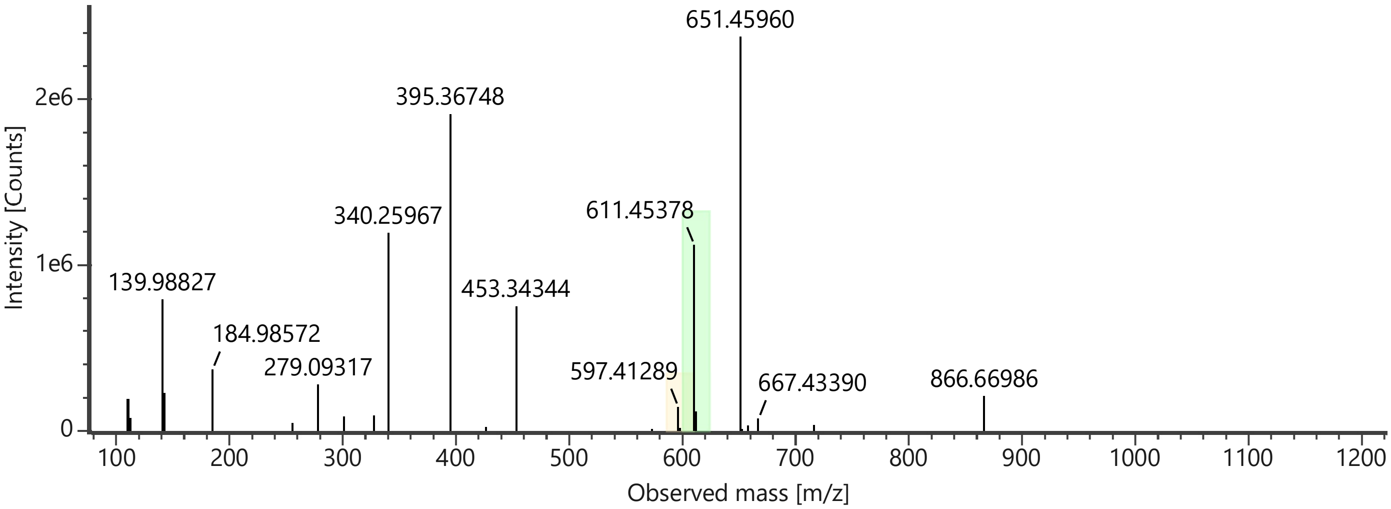

Item name: Sep257+ve
Item description: Mervat253

Channel name: High energy : Time 25.2638 +/- 0.0237 minutes

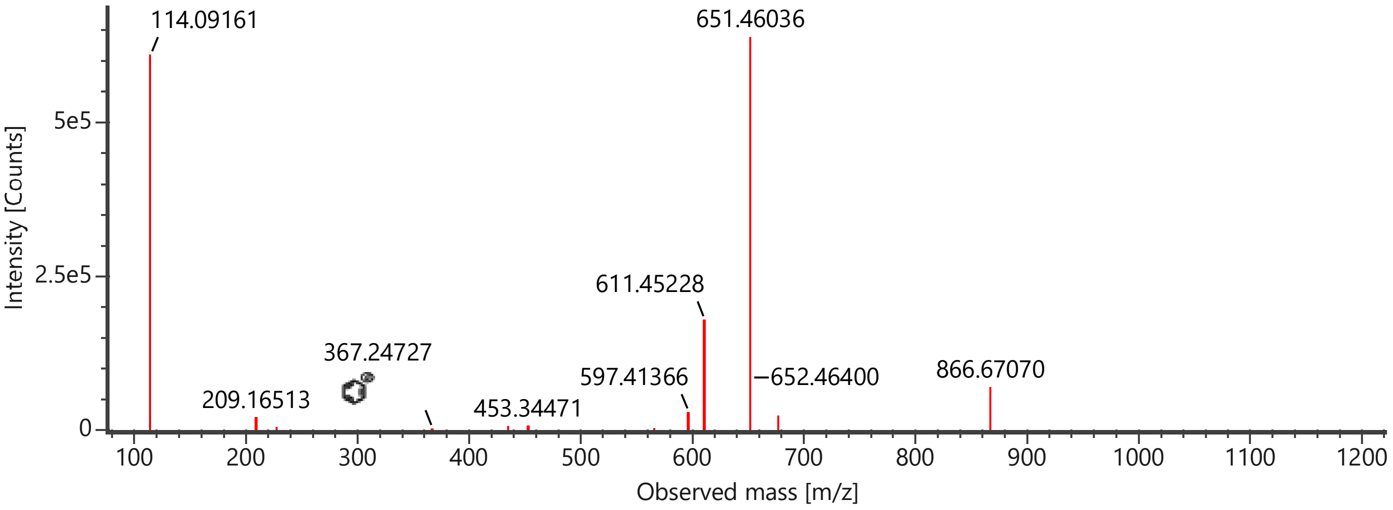

Component name: beta-Sitosterol-alpha-glucoside

Item name: Sep257+ve
Channel name: beta-Sitosterol-alpha-glucoside [+Na] : (48.1 PPM) 599.4283

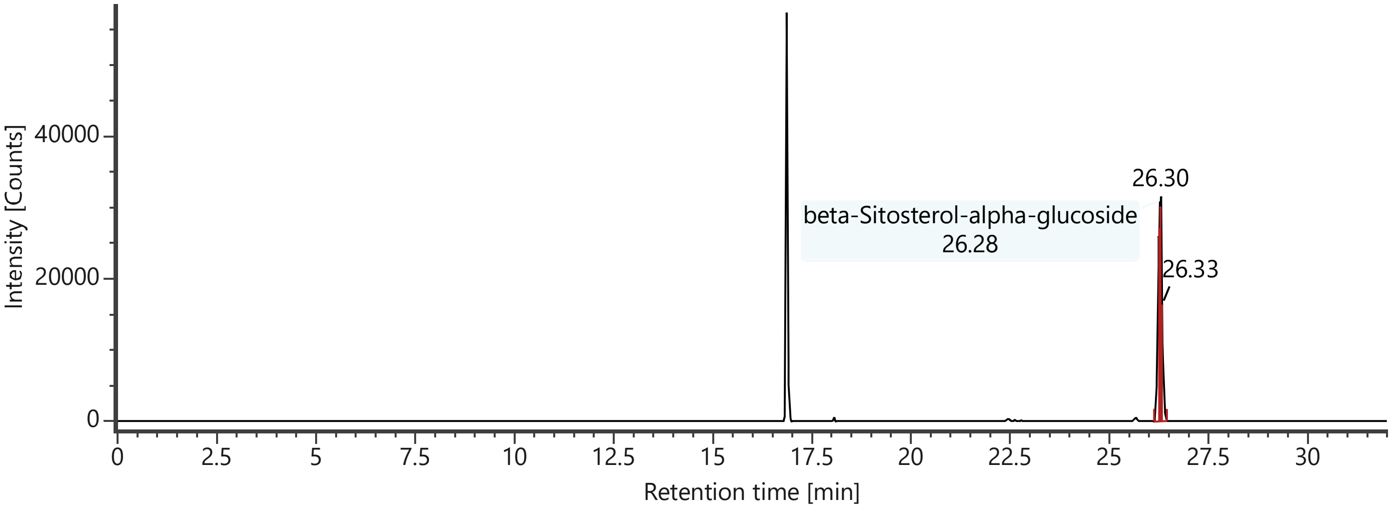

Item name: Sep257+ve
Item description: Mervat253
Channel name: Low energy : Time 26.2820 +/- 0.0237 minutes

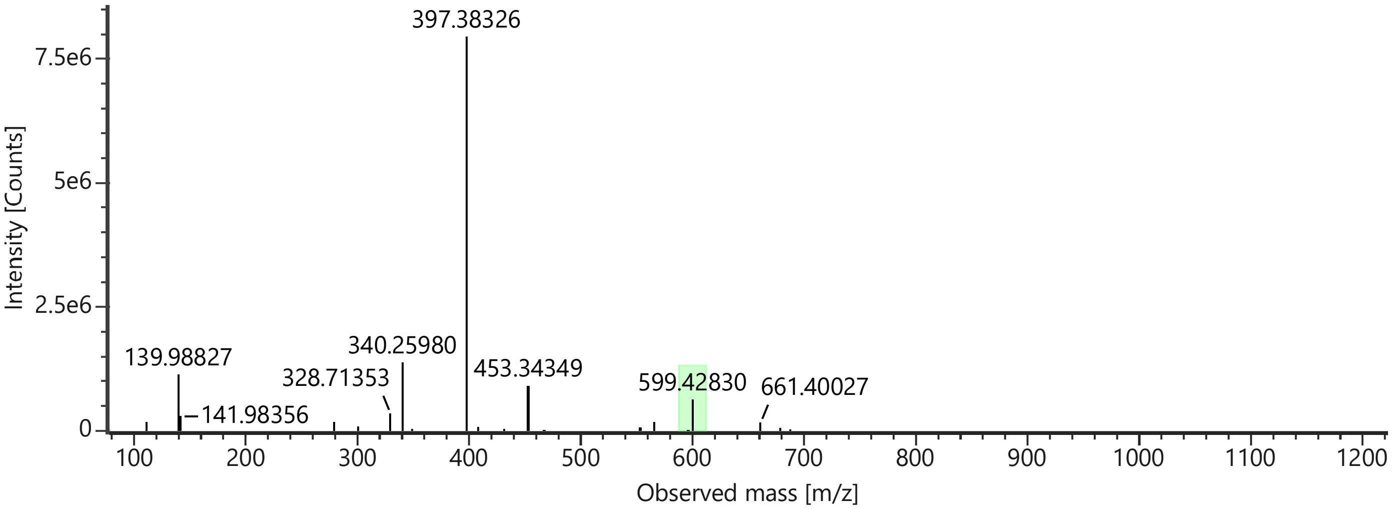

Item name: Sep257+ve
Item description: Mervat253

Channel name: High energy : Time 26.2820 +/- 0.0237 minutes

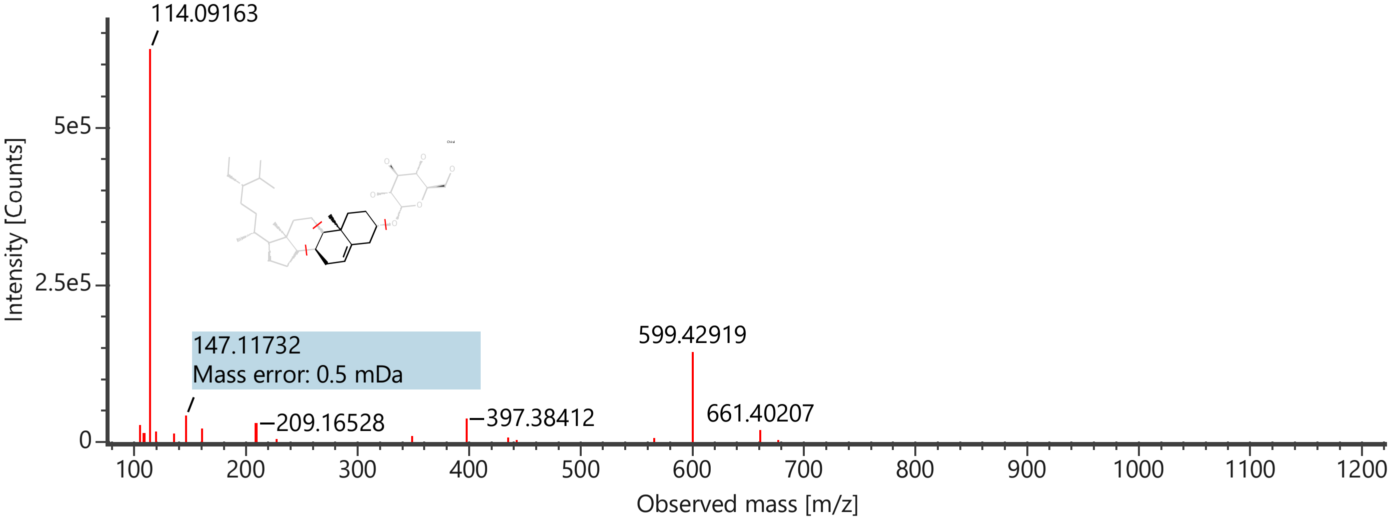

o po e a e 2 ar go ero

Item name: Sep257+ve
Channel name: (24s)-Saringosterol [+H] : (48.1 PPM) 429.3732

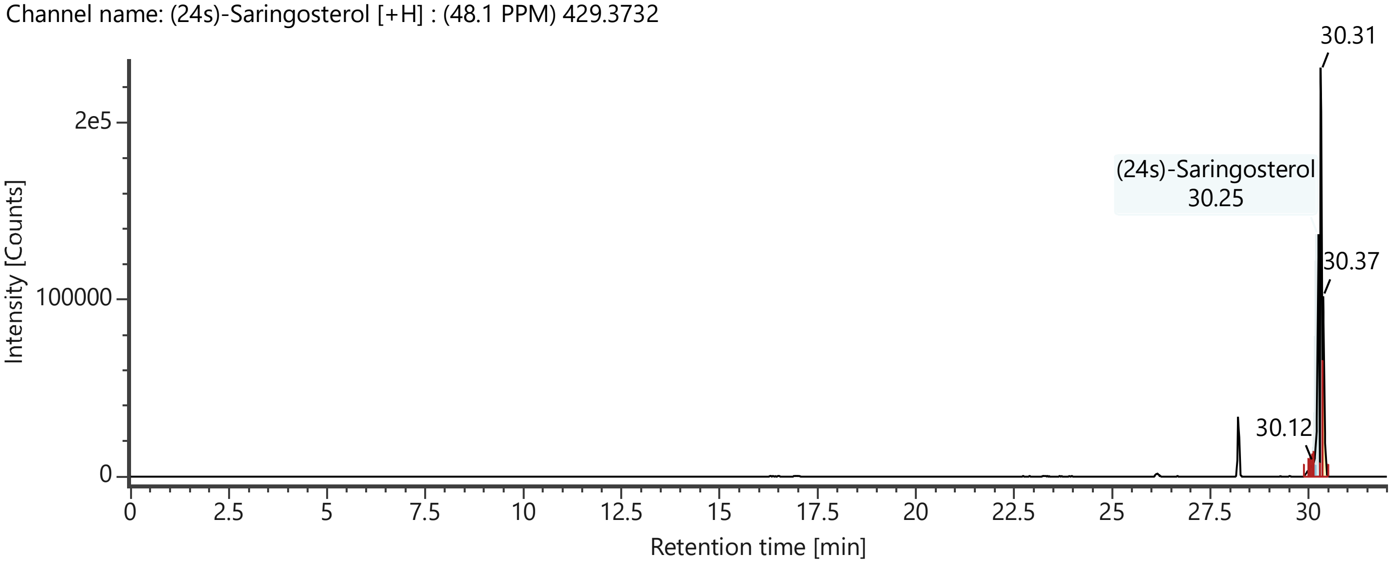

Item name: Sep257+ve
Item description: Mervat253
Channel name: Low energy : Time 30.2431 +/- 0.0237 minutes

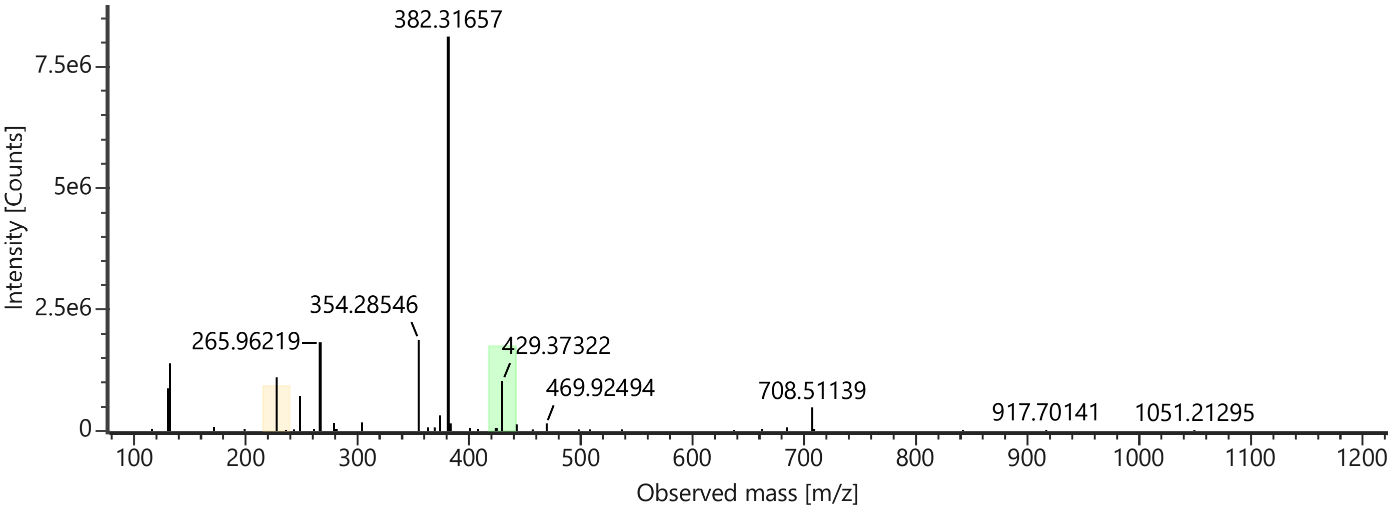

rea ed ad o a

rea ed o ep 1 202

I e a e a a eae Fa e ode rea ed e 1 2 10 g p a dard e

I e a e ep2 e a e a e g e erg e 0 2 1 0 02 e

I e de r p o Mer a 2

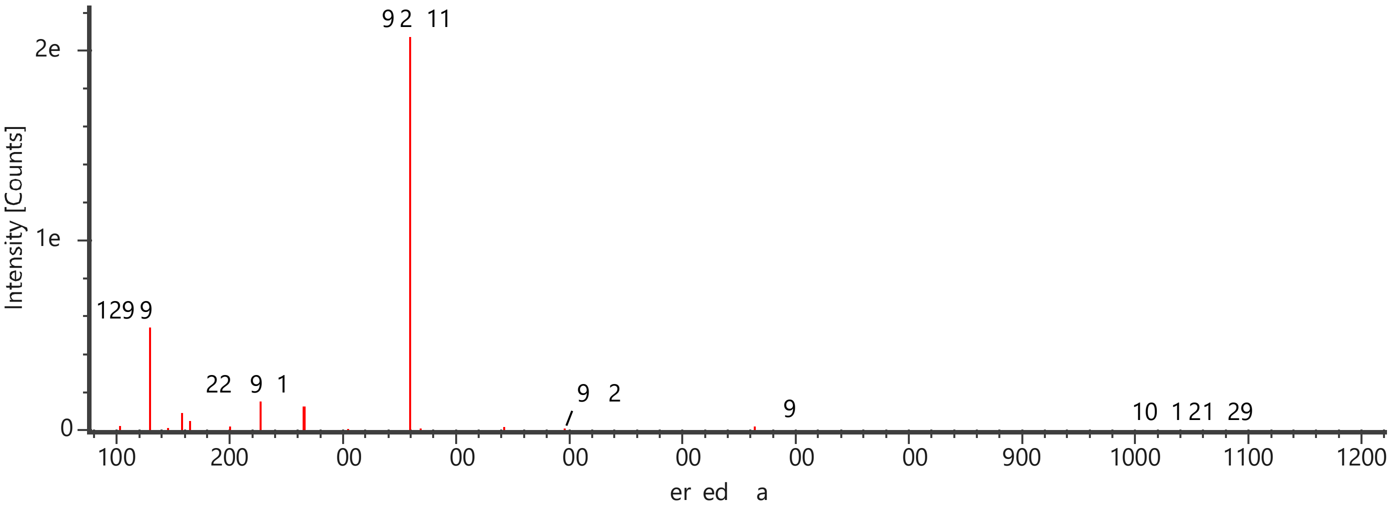

o po e a e 2 ar go ero

tem name: ep e
C annel name: arin o terol : M

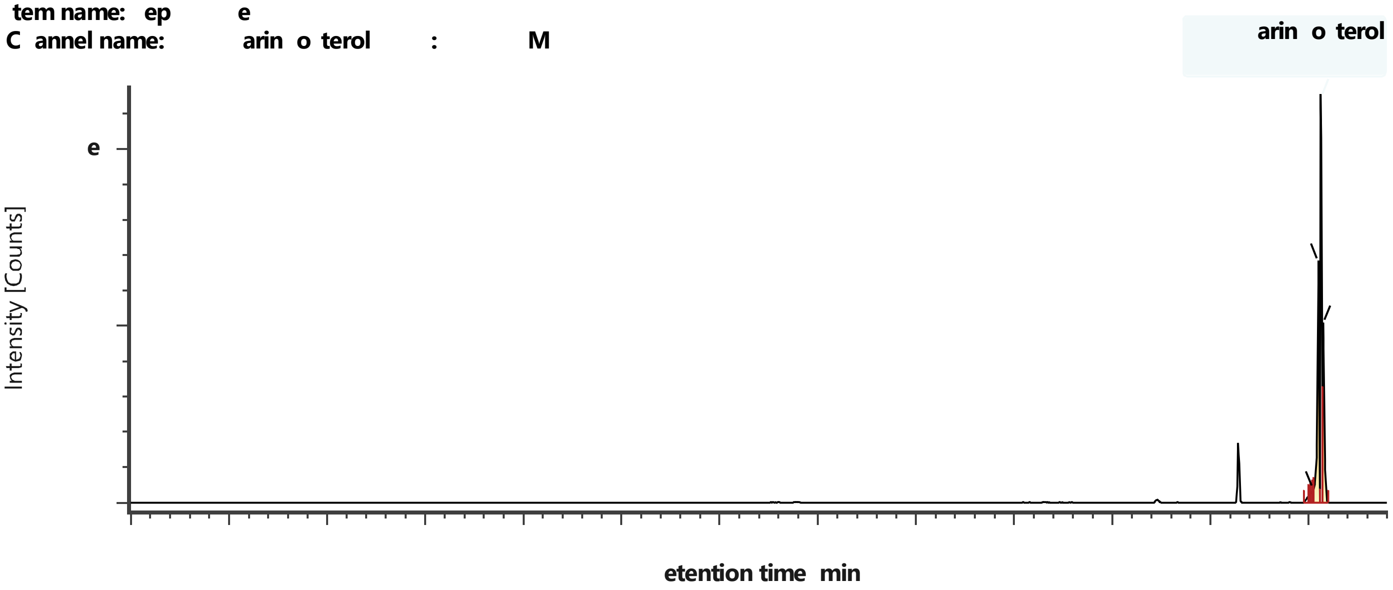

tem name: ep e
tem de ription: Mer at
C annel name: Lo ener : ime minute

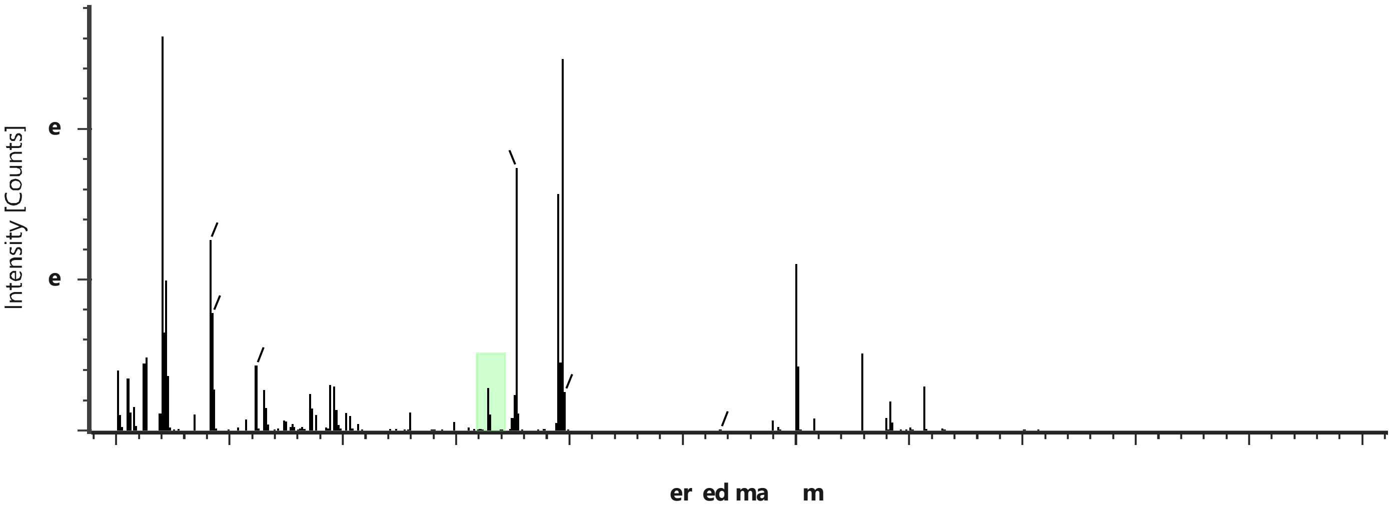

I e a e a a eae Fa e ode rea ed e 13 2 1 g p a dard e

I e a e ep2 e a e a e g e erg e 3 32 1 23 e
I e de r p o Mer a 2 3

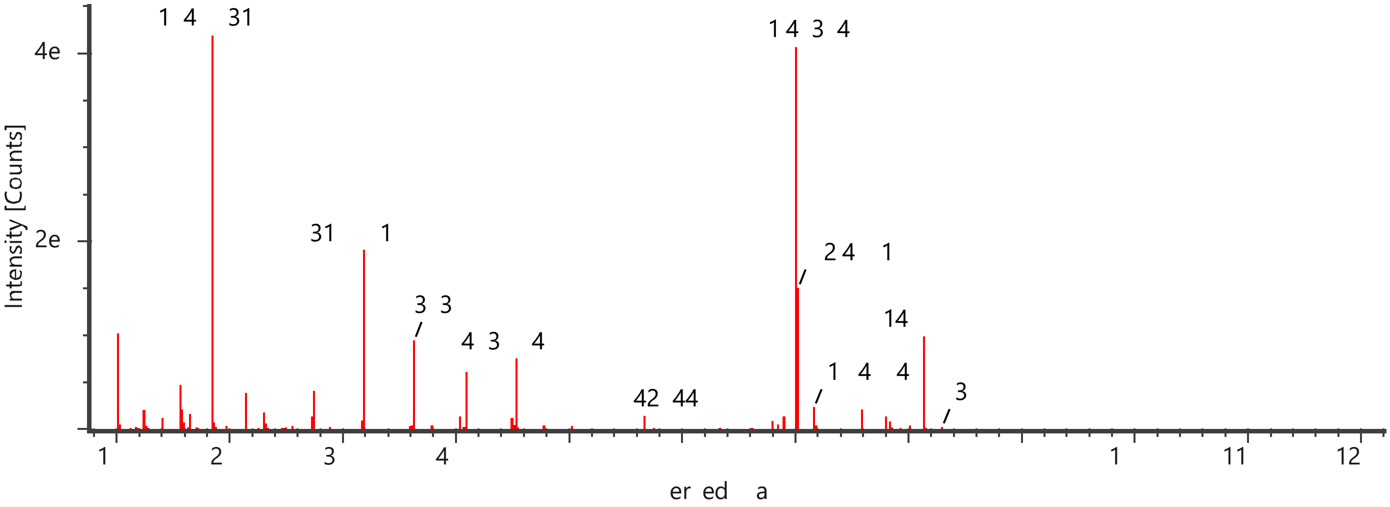

Component name: (24s)-Saringosterol

tem name: ep 5 e
Channel name: s - arin osterol : M 6

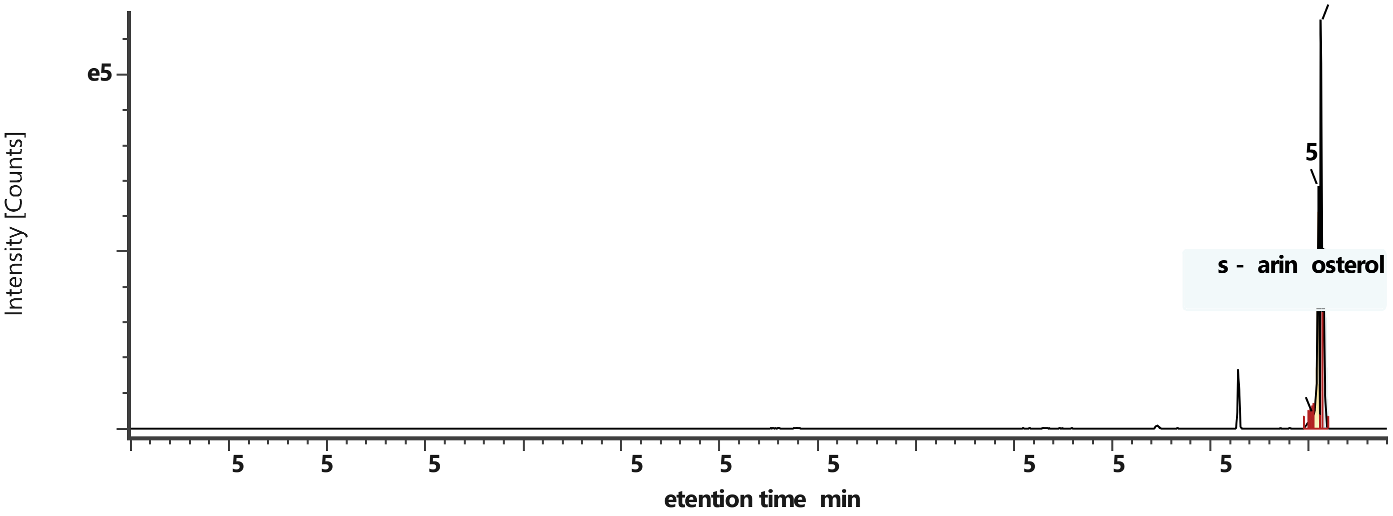

tem name: ep 5 e
tem description: Mer at 5
Channel name: Lo ener y : ime 5 - minutes

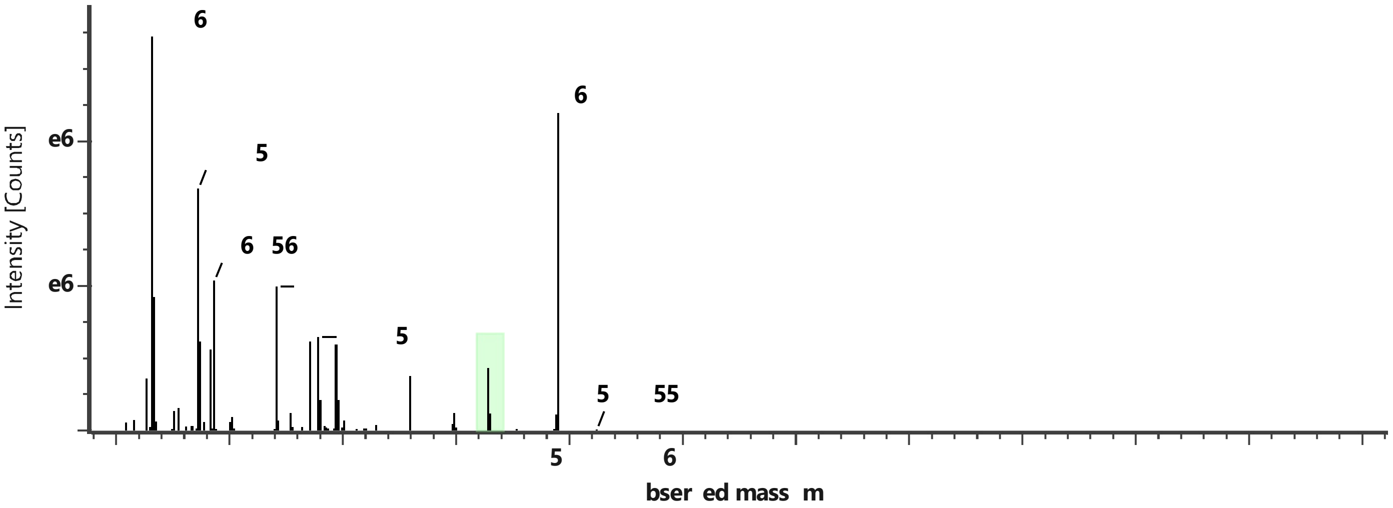

Item name: Sep257+ve
Item description: Mervat253

Channel name: High energy : Time 30.3857 +/- 0.0237 minutes

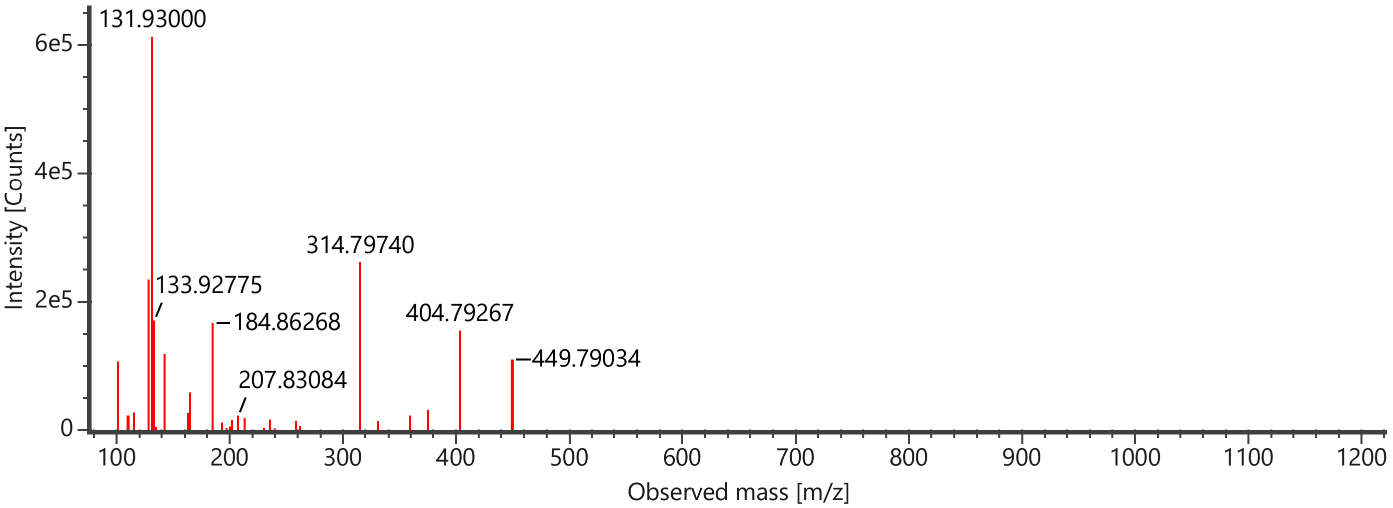

Acquisition was carried out on XEVO G3 QTof instrument , Waters Corporaton ,Milford,MA01757 U.S.A, Mass Spectrometer.

Library Name

Waters Traditional Medicine Library [Jun23]

Experimental Record : Sep257+ve

Quaternary Solvent Manager

Promoted Parameters

Gradient Table

General

Solvent Name A: Waters+0.1%FA

Solvent Name B: ACN+0.1%FA

Solvent Name C: Water+Methanol

Solvent Name D: Methanol

Comment:

Low Pressure Limit: 0 psi

High Pressure Limit: 15000 psi

Gradient Table

Time (min)	Flow Rate (mL/min)	Composition A (%)	Composition B (%)	Composition C (%)	Composition D (%)	Curve
0.00	0.400	90.0	10.0	0.0	0.0	Initial
2.00	0.400	90.0	10.0	0.0	0.0	6
5.00	0.400	70.0	30.0	0.0	0.0	6
15.00	0.400	30.0	70.0	0.0	0.0	6
22.00	0.400	10.0	90.0	0.0	0.0	6
25.00	0.400	10.0	90.0	0.0	0.0	6
26.00	0.400	0.0	100.0	0.0	0.0	6
29.00	0.400	0.0	100.0	0.0	0.0	6
32.00	0.400	90.0	10.0	0.0	0.0	1

Data

System Pressure (psi) Channel Enable: Yes

Flow Rate (mL/min) Channel Enable: No

A Composition (%) Channel Enable: No

B Composition (%) Channel Enable: No

C Composition (%) Channel Enable: No

D Composition (%) Channel Enable: No

Primary Pressure (psi) Channel Enable: No

Accumulator Pressure (psi) Channel Enable: No

Degasser Pressure (psi) Channel Enable: No

Miscellaneous

Seal Wash Period: 5.00 min

Ramp rate accelerate to 2 mL/min in: 0.45 min

Calculated ramp rate: 4.444 mL/min2

Gradient start: At injection

Sample Manager FTN

General

Wash Solvent: Water

Wash Solvent Pre Inject: 0 s

Wash Solvent Post Inject: 6 s

Purge Solvent: Water

Load Ahead: Disabled

Loop Offline: Disabled

Column Temperature Enable: No

Sample Temperature Enable: No

Active Preheater: Disabled

Comment:

Data

Sample Temperature (°C) Channel Enabled: Yes

Column Temperature (°C) Channel Enabled: No

Ambient Temperature (°C) Channel Enabled: Yes

Sample Pressure (psi) Channel Enabled: No

Seal Force (%) Channel Enabled: No

Pre Heater Temperature (°C) Channel Enabled: No

Dilution

Dilution Enable: Disabled

Needle Placement (from bottom): Disabled

Dilution Dispense Purge Solvent: Disabled

Post Dilution Delay: Disabled

Events

Run Events: No

Advanced

Syringe Draw Rate: Automatic

Needle Placement (from bottom): Automatic

Pre Aspirate Air: Automatic

Post Aspirate Air: Automatic

Mix Stroke Cycles: Automatic

Mix Stroke Volume: Automatic

Xevo G3 QTof

Method

Polarity: Positive

Analyzer mode: Sensitivity

MS^E

Start time: 0.00 min

End time: 32.00 min

Low mass: 100 m/z

High mass: 1200 m/z

Scan time: 0.150 s

Low collision energy: 6 V

High collision energy ramp start: 20 V

High collision energy ramp end: 45 V

Intelligent Data Capture: On

Intelligent Data Capture threshold: Medium (10)

Source parameters

Source type: ESI

Source temperature: 120 °C

Desolvation temperature: 550 °C

Cone gas: 50 L/h

Desolvation gas: 1000 L/h

Capillary voltage: 1.00 kV

Sample cone voltage: 40 V

Lock correction

Mode: Automatic

Automatic sampling interval: Yes

Options

Acquisition check failure: Continue with lock correction

Automatic detector check: Off

Events

Time (min)	Event	Parameters
Initial	Flow state	LC, Sample

Method trigger

Trigger type: Network

Post Run Report

Quaternary Solvent Manager

Software version: 3.3.1

Firmware version: 1.72.415 (Aug 7 2018)

Checksum: 0x93efe762

Serial number: D23QSP719A

Minimum system pressure: 4926 psi

Maximum system pressure: 11965 psi

Mean system pressure: 8782 psi

Messages:

Sample Manager FTN

Software version: 3.3.1

Firmware Version: 1.71.395 (Feb 14 2018)

Checksum: 0x285858d7

Serial Number: L22FTP271G

Sample Syringe Size: 100 µL

Extended Loop Size: 0 µL

Needle Size: 15 µL

Column Type: ACQUITY UPLC® BEH C18 1.7µm

Column Serial Number: 02483432115760

Column Injections: 612

Min Sample Temperature: 23.0 °C

Max Sample Temperature: 23.1 °C

Mean Sample Temperature: 23.0 °C

Min Column Temperature: 22.9 °C

Max Column Temperature: 23.0 °C

Mean Column Temperature: 23.0 °C

Auto Defrost Enabled: False

Messages:

Xevo G3 QTof

Serial number: YGA0187

Instrument driver version: 1.2.0

Dynamic parameters

Scan time for function Reference: 0.050 s

Scan time for function MSe Low Collision: 0.150 s

Scan time for function MSe High Collision: 0.150 s

Calibration

Calibrated

Modes

ADC mode: Signum

Enhance mode: None

MSMS mode: MS

Analyser mode: Sensitivity

Polarity: Positive

Quad mode: 3940

Source mode: ESI

TOF mode: TOF

Quadrupole options: Automatic profile

ESI LockSpray

Capillary voltage: 1.00 kV

Reference capillary voltage: 3.00 kV

Sampling cone voltage: 40 V

Source temperature: 120 °C

Desolvation temperature: 550 °C

Cone gas flow rate: 50 L/h

Desolvation gas flow rate: 1000 L/h

Sample Fluidics

Reservoir: Wash

Flow path: Infusion

Infusion flow rate: 100.0 µl/min

Fill volume: 250 µL

Wash cycle: 2

Reference Fluidics

Reservoir: B

Flow path: Infusion

Infusion flow rate: 20.0 µl/min

Baffle position: Sample

Illumination: Off

StepWave

Source offset: 30 V
StepWave RF: 150 V
Ion guide RF: 350 V
Body gradient: 10.0 V
Head gradient: 10.0 V
Ion guide 2 offset: 0.3 V
Ion guide 1 offset: 3.0 V
Diff aperture 2: 0.0 V
Wave height: 0.5 V
Wave velocity: 150 m/s

Quadrupole

Low mass resolution: 4.7
High mass resolution: 15.0
Pre-filter: 2.0 V
Ion energy: 0.2 V

DRE

Collector: 60 V
Collector pulse: 10 V
Stopper: 10 V
Stopper pulse: 20 V
pDRE attenuate: Off
pDRE transmission: 1.0 %

Collision

Entrance: 2 V
Gradient: 1.0 V
Static offset: 120 V
Offset B: 0.0 V
Offset C: 0.5 V
Exit: 15 V
Cell RF: 150 V
Cell 2 RF gain: 10 V

Tof

Acceleration 1: 30 V
Acceleration 2: 120 V
Acceleration steering: 1.00 V
Aperture 2: 30 V
Transport 1: 30 V
Transport 2: 30 V
Steering: 0.60 V
Tube lens: 17 V
Entrance: 14 V
Pusher: 1900 V
Pusher offset: -0.78 V
Puller: 1400 V
Puller offset: 0.00 V
Flight tube: 9.00 kV
Reflectron: 1.602 kV
Reflectron grid: 1.7055 kV

ADC

Baseline threshold: -2363
Amplitude threshold: 11
Ion area threshold: 0
T0: -250 ns
Veff: 6332.891 V
Trigger threshold: 1.0
Centroid threshold: -1
Ion area offset: 0
Average single ion intensity: 28.8
Measured m/z: 556.2766
Measured charge: 1
ADC algorithm: ADC
Detector voltage: 2752 V

MS Profile

Quadrupole options: Automatic profile

Set mass: 785.80

Report Log

Template Report

Body Objects

Chapter: Analysis information

Untitled

Report Object [17]: Autoscaled chromatogram

Object saved in the Report Template was modified

Chapter: Analysis results

Untitled

Report Object [10]: Autoscaled chromatogram

Object saved in the Report Template was modified

Report Object [4]: Sample results

Object saved in the Report Template was modified

Group Report Object [5]: Untitled

Untitled

Group Report Object [5]: Untitled

Object saved in the Report Template was modified
