# Supplementary material for: Integrated metabolomic, nanoformulation, and network pharmacology approach reveals multifunctional bioactivities of an Ocimum sanctum nanoemulsion
Source: Front Bioeng Biotechnol. 2026 Mar 20;14:1731720. doi: 10.3389/fbioe.2026.1731720 (PMC13047145; doi:10.3389/fbioe.2026.1731720)
Supplement: Supplementary file 2 [file DataSheet3.pdf]

Created by: Ayad, Younan

Created on: Sep 18, 2025

Item name: Lamiaceae family -ve mode

Created time: 13:05:43 Egypt Standard Time

Analysis Information

Item name:	Lamiaceae family -ve mode	Analysis Method Item name:	Extract -ve mode
Version:	2	Analysis Method Version:	1
Modified date:	Sep 18, 2025 13:04:14 Egypt Standard Time	Sample Set Created date:	
Modified by:	Ayad, Younan	Sample Set Instrument system name:	
Folder:	Company/LAB/data		

Analysis injection list

.	Item name	Item description	Replicate number	Sample position	Injection volume (µL)
1	Sep257-ve	Mervat253	1	1:A,2	0.50

Item name: Sep257-ve
Channel name: 2: TOF MSe (50-1200) 6V ESI- (BPI) : Integrated : Smoothed

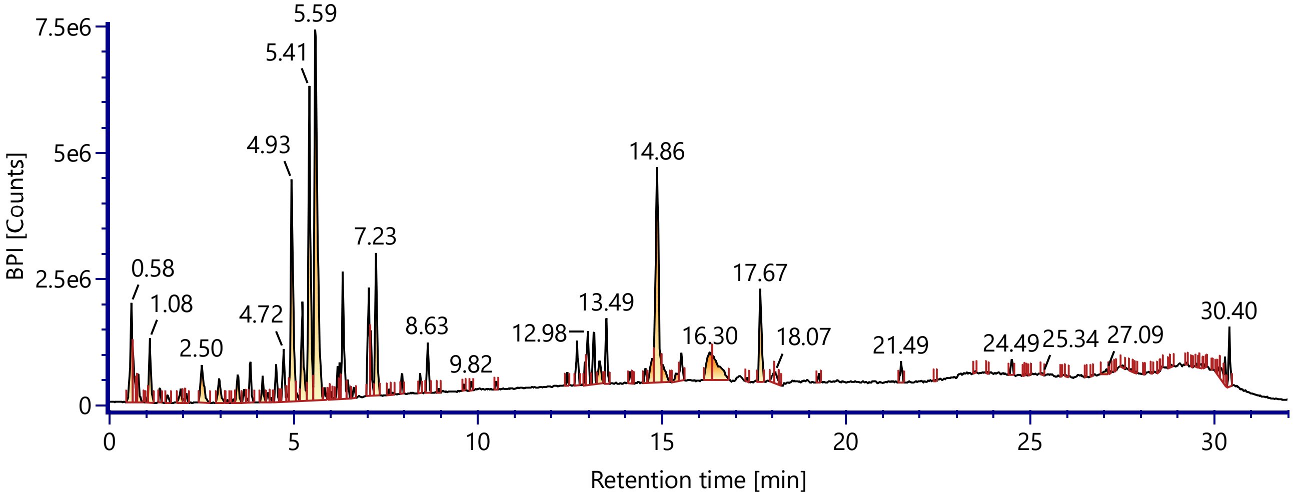

Item name: Sep257-ve

Item name: Sep257-ve
Channel name: 2: TOF MSe (50-1200) 6V ESI- (BPI)

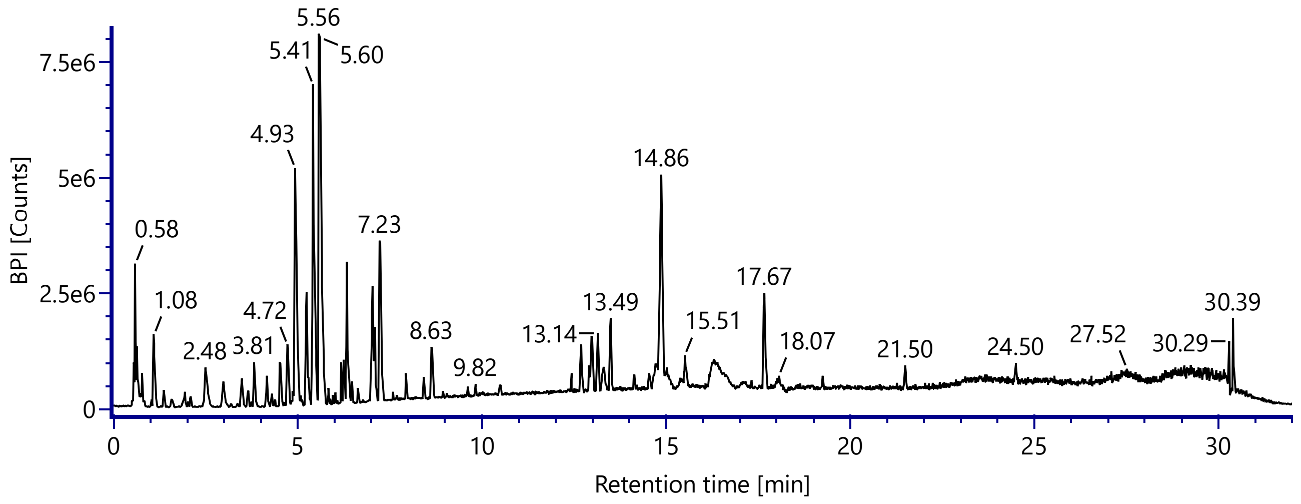

Item name: Sep257-ve, Sample position: 1:A,2, Replicate number: 1

.	Component name	Formula	Observed m/z	Mass error (ppm)	Observed RT (min)	Response	Adducts
1	trans Caffeic acid	C9H8O4	179.0356	3.3	1.08	34668	-H
2	1-O-Caffeoyl-β-D-glucopyranoside	C15H18O9	341.0884	1.6	1.40	27561	-H
3	1-O-Caffeoyl-β-D-glucopyranoside	C15H18O9	341.0885	2.0	1.96	22118	-H
4	p-Coumaric acid	C9H8O3	163.0402	1.1	2.50	56455	-H
5	trans Caffeic acid	C9H8O4	179.0353	1.6	2.97	296055	-H
6	1-Caffeoylglycerol	C12H14O6	253.0718	0.1	3.19	66827	-H
7	Kaempferol 3-O-β-Rutinoside	C27H30O15	593.1498	-2.4	3.82	628386	-H
8	Kaempferol 3-O-β-Rutinoside	C27H30O15	593.1508	-0.6	4.18	23221	-H
9	Kaempferol 3-O-α-L-rhamnosyl (1→2)β-D-xyloside	C26H28O14	563.1405	-0.2	4.27	37477	-H
10	Kaempferol-3-O-β-D-galactoside	C21H20O11	447.0933	0.1	4.28	34762	-H
11	p-Coumaric acid	C9H8O3	163.0407	3.9	4.38	54578	-H
12	Luteolin-4'-beta-D-glucoside	C21H20O11	447.0931	-0.4	4.52	438443	-H

.	Component name	Formula	Observed m/z	Mass error (ppm)	Observed RT (min)	Response	Adducts
13	1-[2-(3,4-Dihydroxyphenyl)-1-carboxy]ethoxycarbonyl-2-(3,4-dihydroxyphenyl)-7,8-dihydroxy-1,2-dihydronaphthalene-3-carboxylic acid	C27H22O12	537.1033	-1.0	4.52	42571	-H
14	1-[2-(3,4-Dihydroxyphenyl)-1-carboxy]ethoxycarbonyl-2-(3,4-dihydroxyphenyl)-7,8-dihydroxy-1,2-dihydronaphthalene-3-carboxylic acid	C27H22O12	537.1040	0.2	4.63	88303	-H
15	Kaempferol 7-O-β-D-glucopyranosyl(1→4)β-D-glucopyranoside	C27H30O16	609.1465	0.6	4.70	430274	-H
16	Kaempferol-7-O-α-L-rhamnoside	C21H20O10	431.0989	1.1	4.73	584792	-H
17	Quercetin-4'-O-β-D-galactoside	C21H20O12	463.0884	0.5	4.87	146242	-H
18	Luteolin 3'-glucoside	C21H20O11	447.0941	1.7	4.89	97957	-H
19	Kaempferol	C15H10O6	285.0414	3.4	4.93	31191	-H
20	Luteolin 7-O-glucuronide	C21H18O12	461.0732	1.4	4.93	3066383	-H
21	Querciturone	C21H18O13	477.0679	0.9	4.94	123291	-H
22	Kaempferol 3-glucoside-7-rhamnoside	C27H30O15	593.1520	1.4	5.04	35382	-H
23	Kaempferol-7-O-β-D-glucoside	C21H20O11	447.0944	2.5	5.22	126468	-H

.	Component name	Formula	Observed m/z	Mass error (ppm)	Observed RT (min)	Response	Adducts
24	(2S)-2-[(1S,2R)-3-[(1S)-1-Carboxy-2-(3,4-dihydroxyphenyl)ethoxy]carbonyl-2-(3,4-dihydroxyphenyl)-7,8-dihydroxy-1,2-dihydronaphthalene-1-carbonyl]oxy-3-(3,4-dihydroxyphenyl)propanoic acid	C36H30O16	717.1467	0.8	5.23	1676364	-H
25	Apigenin 7-O-beta-D-glucopyranoside	C21H20O10	431.0993	2.1	5.34	126934	-H
26	Apigenin-4'-O-glucuronide	C21H18O11	445.0782	1.2	5.41	3604192	-H
27	Apigenin	C15H10O5	269.0465	3.7	5.41	66753	-H
28	(2S)-2-[(1S,2R)-3-[(1S)-1-Carboxy-2-(3,4-dihydroxyphenyl)ethoxy]carbonyl-2-(3,4-dihydroxyphenyl)-7,8-dihydroxy-1,2-dihydronaphthalene-1-carbonyl]oxy-3-(3,4-dihydroxyphenyl)propanoic acid	C36H30O16	717.1463	0.2	5.48	116833	-H
29	Luteolin-7-O-(6′′-methyl ester)-β-D-glucuronide	C22H20O12	475.0889	1.5	5.49	210342	-H
30	Kaempferol-7-O-β-D-glucoside	C21H20O11	447.0936	0.7	5.55	33961	-H
31	Rosmarinic acid	C18H16O8	359.0779	1.8	5.59	5295190	-H

.	Component name	Formula	Observed m/z	Mass error (ppm)	Observed RT (min)	Response	Adducts
32	(2S)-2-[(1S,2R)-3-[(1S)-1-Carboxy-2-(3,4-dihydroxyphenyl)ethoxy]carbonyl-2-(3,4-dihydroxyphenyl)-7,8-dihydroxy-1,2-dihydronaphthalene-1-carbonyl]oxy-3-(3,4-dihydroxyphenyl)propanoic acid	C36H30O16	717.1466	0.7	5.59	2124110	-H
33	7,8-Dihydroxy-2-(3,4-dihydroxyphenyl)-1,2-dihydronaphthalene-1,3-dicarboxylic acid	C18H14O8	357.0625	2.6	5.59	171496	-H
34	3,5-Dihydroxy-4',7-dimethoxyflavone	C17H14O6	313.0726	2.8	5.59	348849	-H
35	cis-Ferulic acid	C10H10O4	193.0515	4.5	5.72	33713	-H
36	Quercilicoside A	C36H58O11	665.3906	0.0	5.77	83583	-H
37	Kaempferol-3-O-6'-trans-coumaroyl-β-D-glucoside	C30H26O13	593.1315	2.4	6.16	29098	-H
38	Casticin	C19H18O8	373.0932	0.8	6.19	35281	-H
39	1-[2-(3,4-Dihydroxyphenyl)-1-carboxy]ethoxycarbonyl-2-(3,4-dihydroxyphenyl)-7,8-dihydroxy-1,2-dihydronaphthalene-3-carboxylic acid	C27H22O12	537.1024	-2.8	6.25	483036	-H
40	6'-O-Caffeoylerigeroside	C20H20O11	435.0937	1.0	6.30	33145	-H
41	Luteolin	C15H10O6	285.0408	1.2	6.33	1187536	-H
42	Quercetin 3'-methyl ether	C16H12O7	315.0512	0.7	6.63	116394	-H

.	Component name	Formula	Observed m/z	Mass error (ppm)	Observed RT (min)	Response	Adducts
43	Quercetagetin 3,4'-Dimethyl Ether	C17H14O8	345.0618	0.5	6.65	64159	-H
44	3,5-Dihydroxy-4',7-dimethoxyflavone	C17H14O6	313.0719	0.3	6.88	60917	-H
45	Apigenin	C15H10O5	269.0459	1.2	7.03	1083313	-H
46	3'-Hydroxygenkwanin	C16H12O6	299.0564	1.1	7.18	78895	-H
47	Ombuin	C17H14O7	329.0667	0.2	7.36	99958	-H
48	3,5-Dihydroxy-4',7-dimethoxyflavone	C17H14O6	313.0719	0.3	7.37	48564	-H
49	Kaempferide	C16H12O6	299.0564	0.9	7.56	84481	-H
50	Quercetin 3,4'-dimethyl ether	C17H14O7	329.0669	0.8	7.70	143863	-H
51	3,5-Dihydroxy-4',7-dimethoxyflavone	C17H14O6	313.0718	0.0	8.43	342849	-H
52	3-Epioleanolic acid	C30H48O3	455.3532	0.2	17.67	1760626	-H
53	3-Epioleanolic acid	C30H48O3	455.3534	0.6	17.87	147630	-H
54	3-Epioleanolic acid	C30H48O3	455.3535	1.0	18.14	977923	-H
55	3-Epioleanolic acid	C30H48O3	455.3514	-3.8	24.51	200783	-H

Component name: trans Caffeic acid

Item name: Sep257-ve
Channel name: trans Caffeic acid [-H] : (52.5 PPM) 179.0356

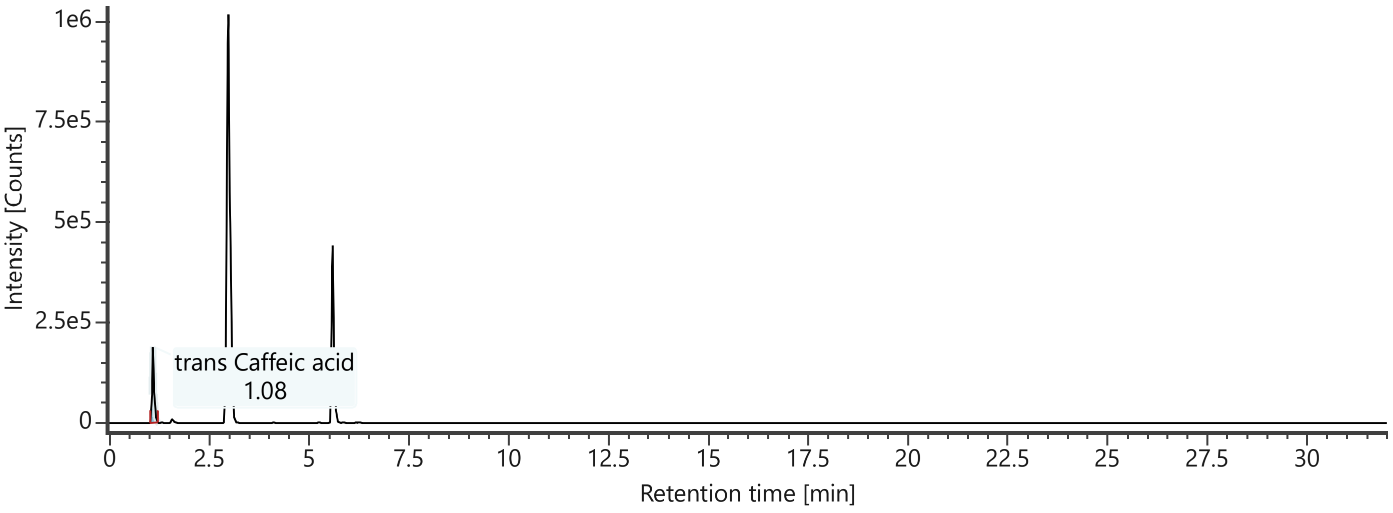

Item name: Sep257-ve
Item description: Mervat253

Channel name: Low energy : Time 1.0836 +/- 0.0222 minutes

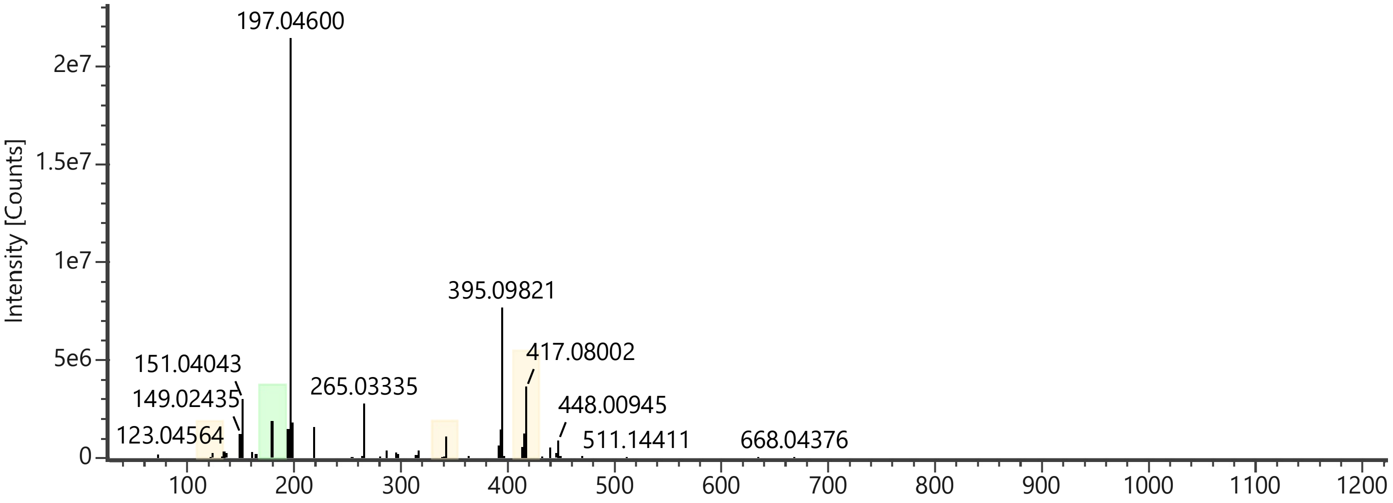

Item name: Sep257-ve
Item description: Mervat253

Channel name: High energy : Time 1.0836 +/- 0.0222 minutes

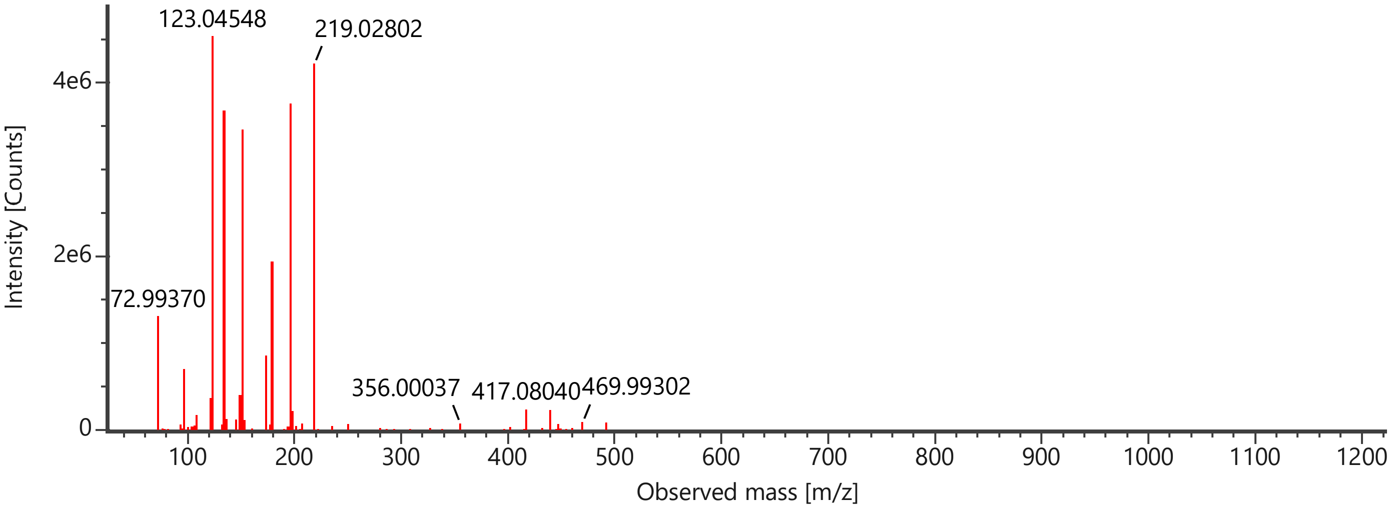

Component name: 1-O-Caffeoyl-β-D-glucopyranoside

Item name: Sep257-ve
Channel name: 1-O-Caffeoyl-β-D-glucopyranoside [-H] : (52.5 PPM) 341.0884

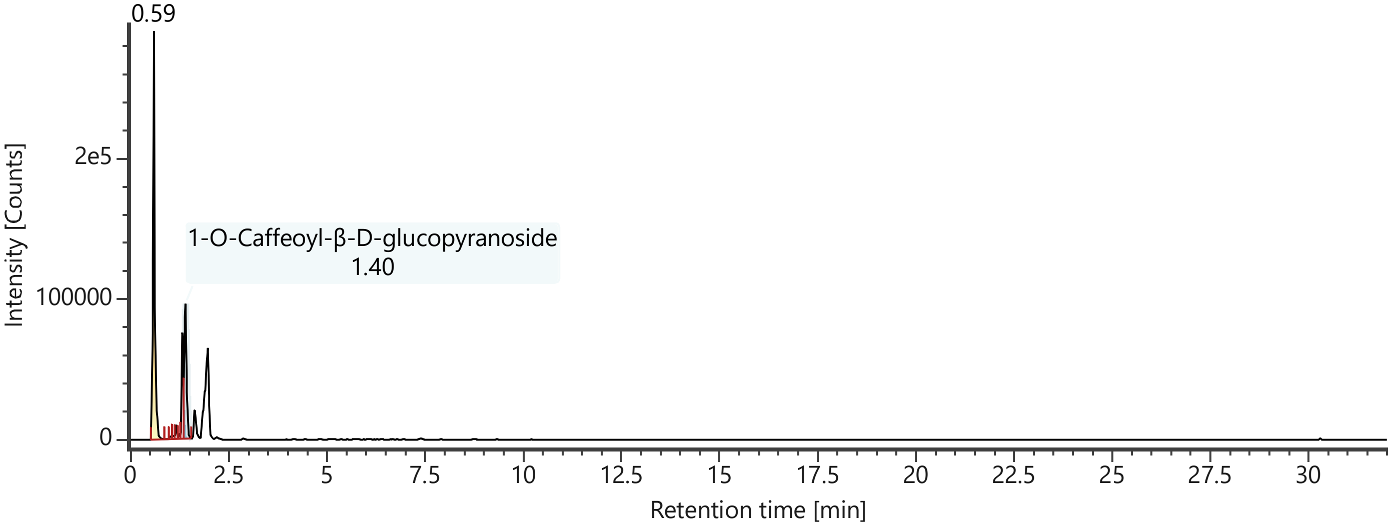

Item name: Sep257-ve
Item description: Mervat253
Channel name: Low energy : Time 1.3991 +/- 0.0222 minutes

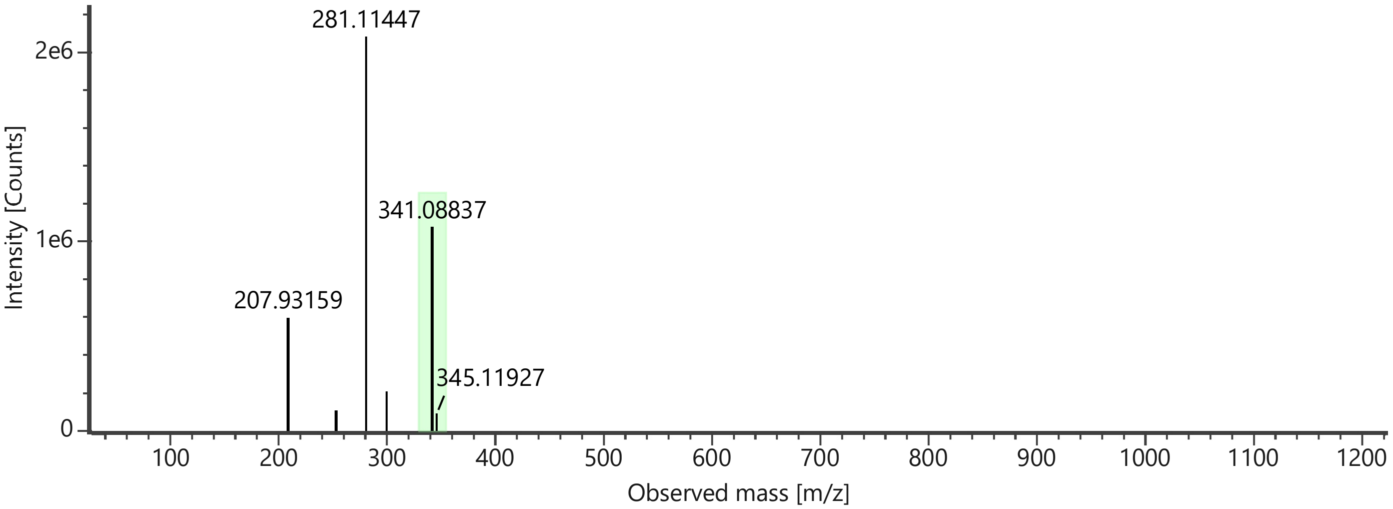

Item name: Sep257-ve
Item description: Mervat253

Channel name: High energy : Time 1.3991 +/- 0.0222 minutes

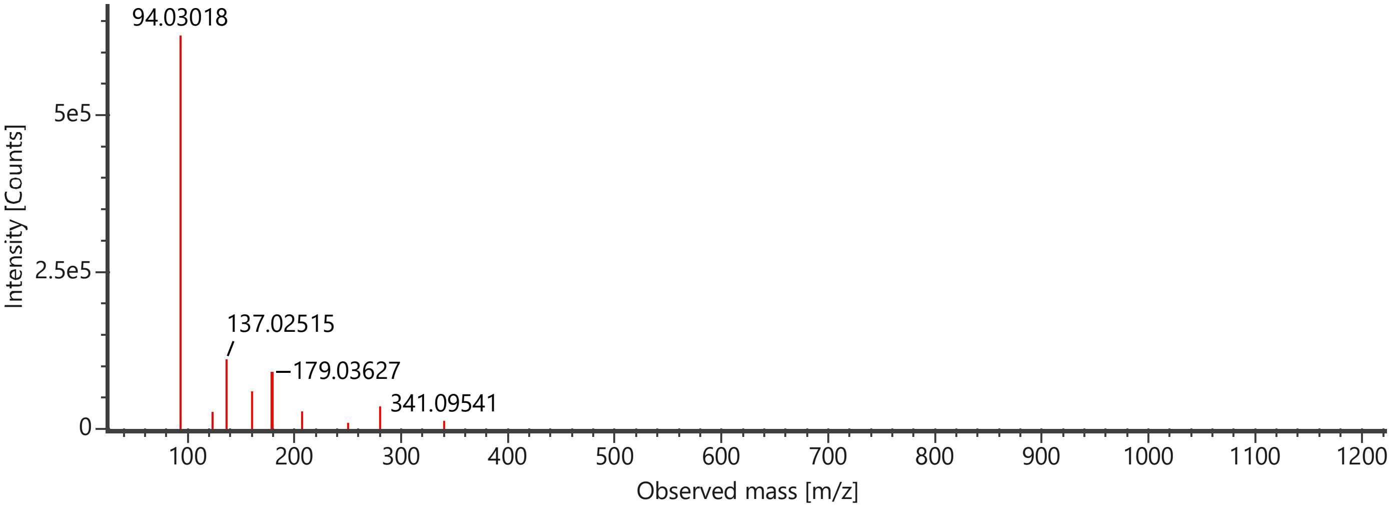

Component name: 1-O-Caffeoyl-β-D-glucopyranoside

Item name: Sep257-ve
Channel name: 1-O-Caffeoyl-β-D-glucopyranoside [-H] : (52.5 PPM) 341.0885

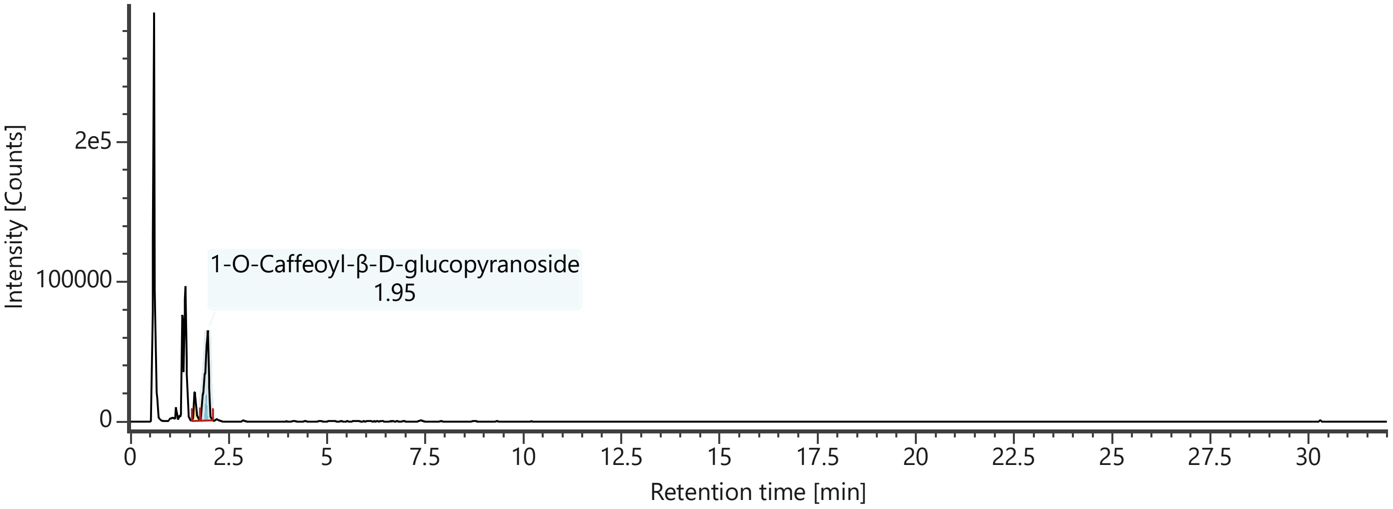

Item name: Sep257-ve
Item description: Mervat253
Channel name: Low energy : Time 1.9564 +/- 0.0222 minutes

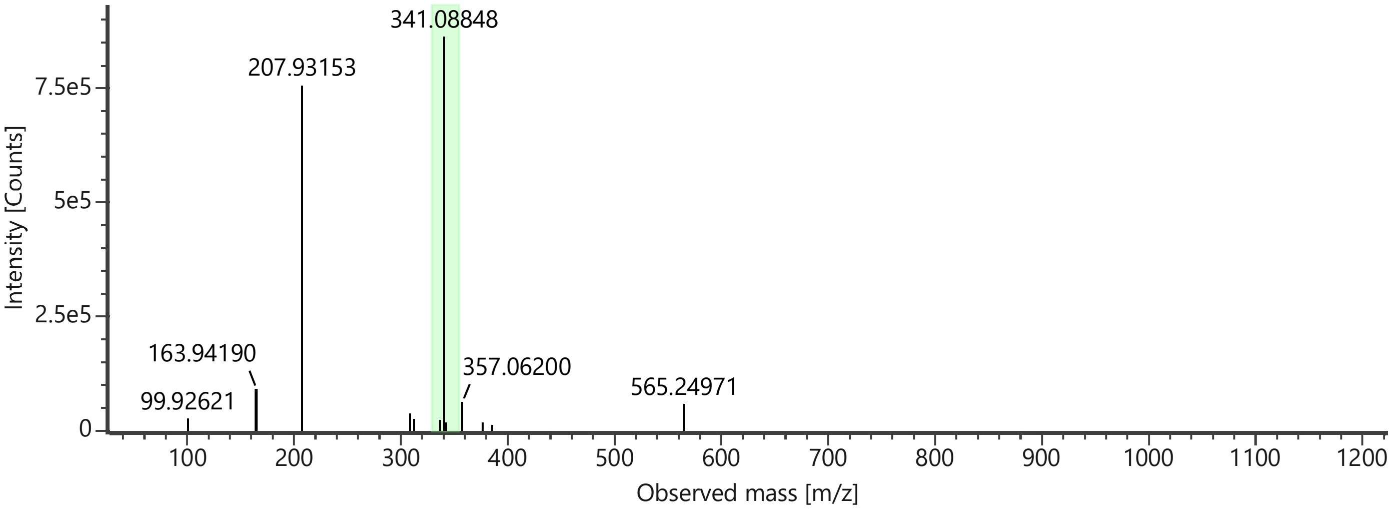

Item name: Sep257-ve
Item description: Mervat253

Channel name: High energy : Time 1.9564 +/- 0.0222 minutes

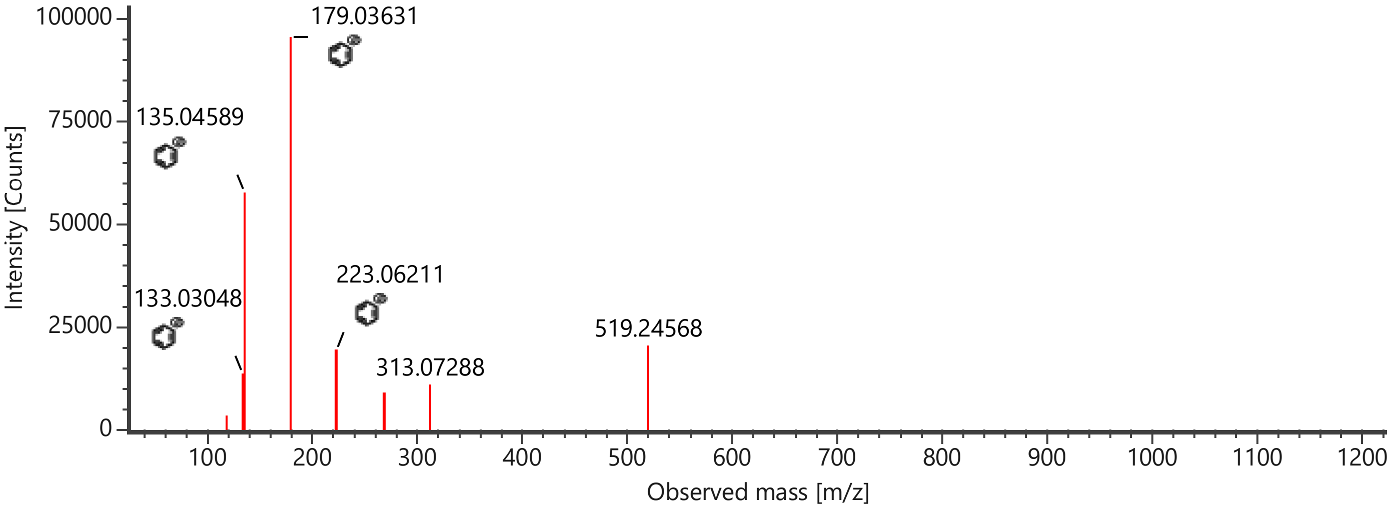

Component name: p-Coumaric acid

Item name: Sep257-ve
Channel name: p-Coumaric acid [-H] : (52.5 PPM) 163.0402

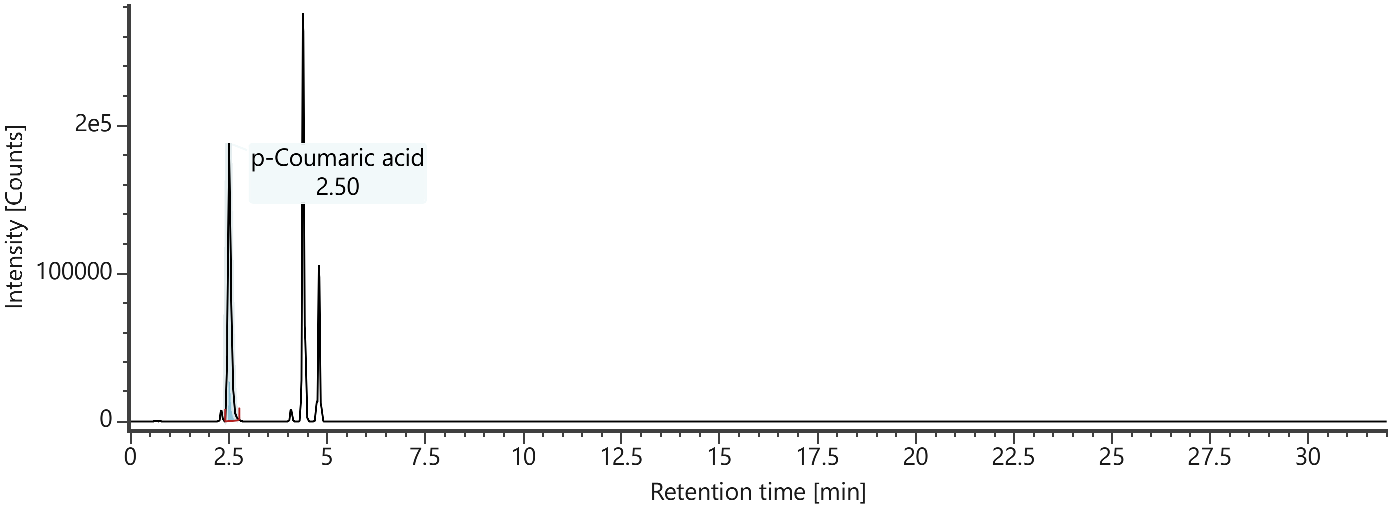

Item name: Sep257-ve
Item description: Mervat253

Channel name: Low energy : Time 2.4966 +/- 0.0222 minutes

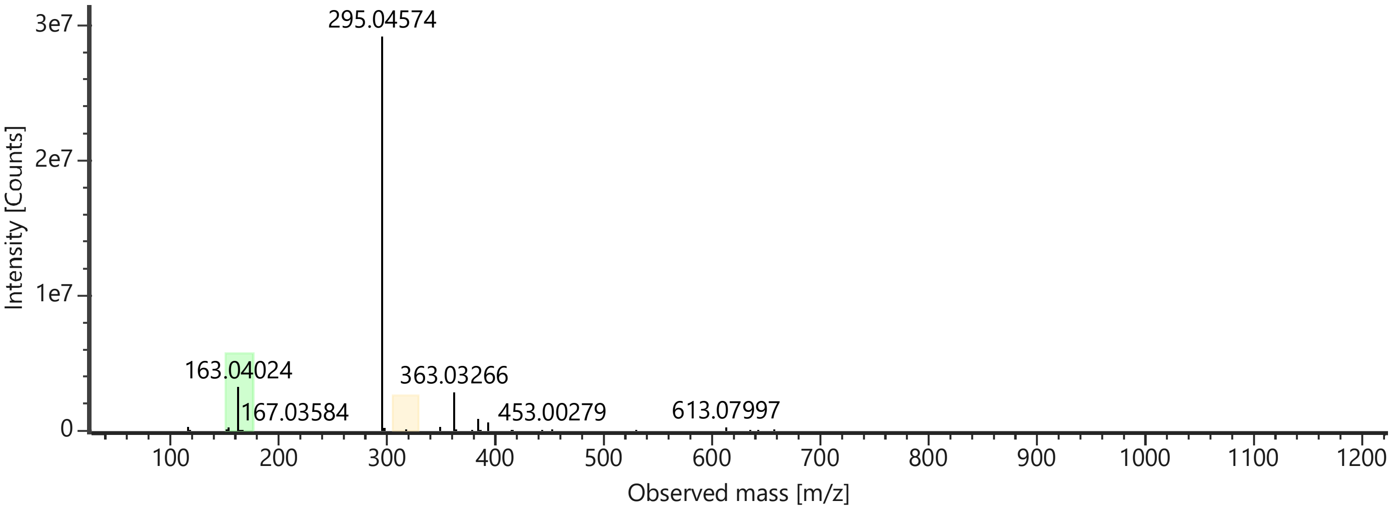

Item name: Sep257-ve
Item description: Mervat253

Channel name: High energy : Time 2.4966 +/- 0.0222 minutes

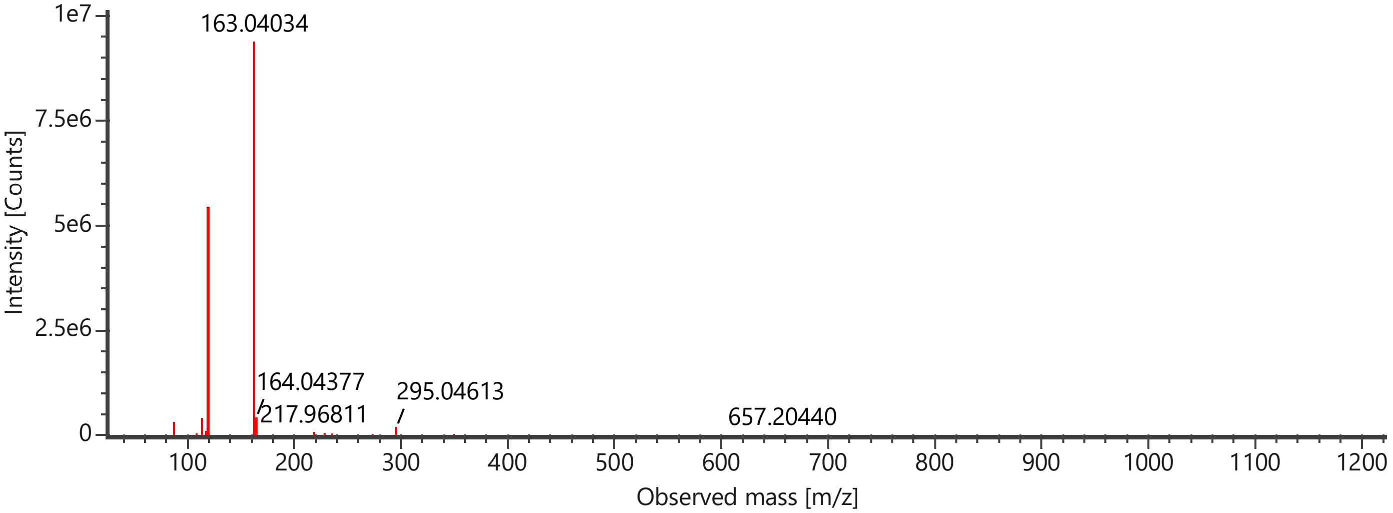

Component name: trans Caffeic acid

Item name: Sep257-ve
Channel name: trans Caffeic acid [-H] : (52.5 PPM) 179.0353

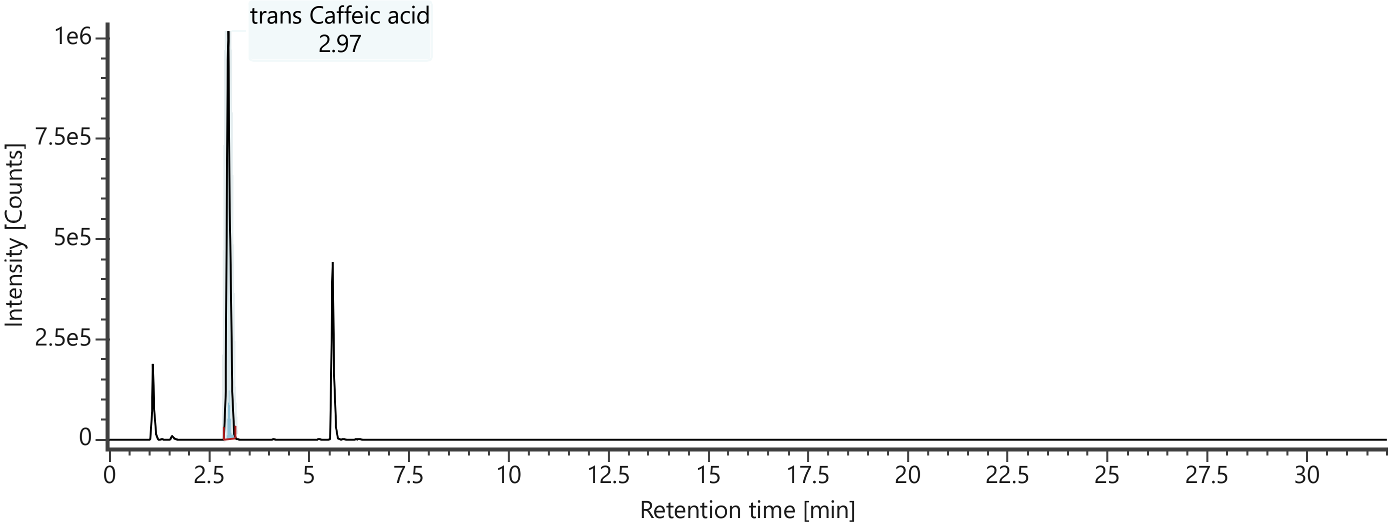

Item name: Sep257-ve
Item description: Mervat253

Channel name: Low energy : Time 2.9727 +/- 0.0222 minutes

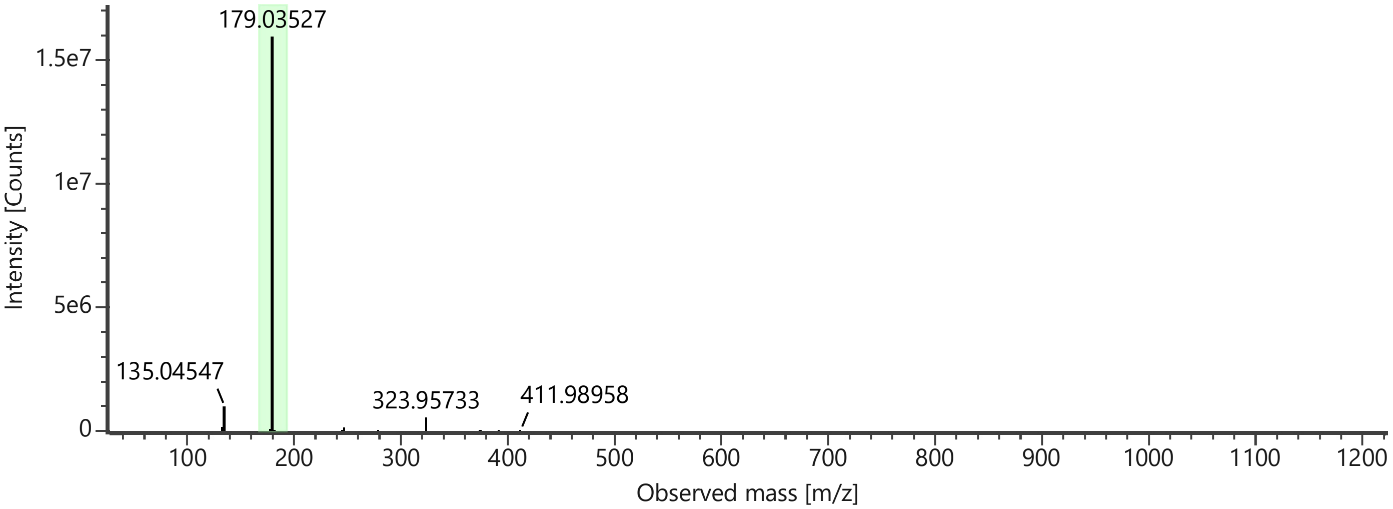

Item name: Sep257-ve
Item description: Mervat253

Channel name: High energy : Time 2.9727 +/- 0.0222 minutes

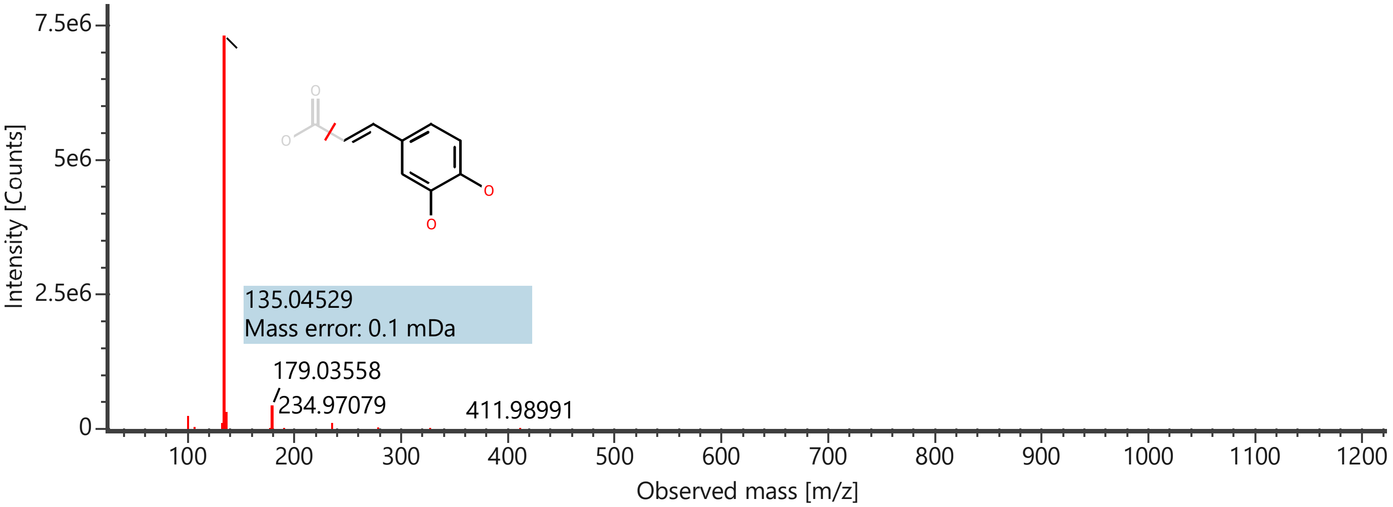

Component name: 1-Caffeoylglycerol

Item name: Sep257-ve
Channel name: 1-Caffeoylglycerol [-H] : (52.5 PPM) 253.0718

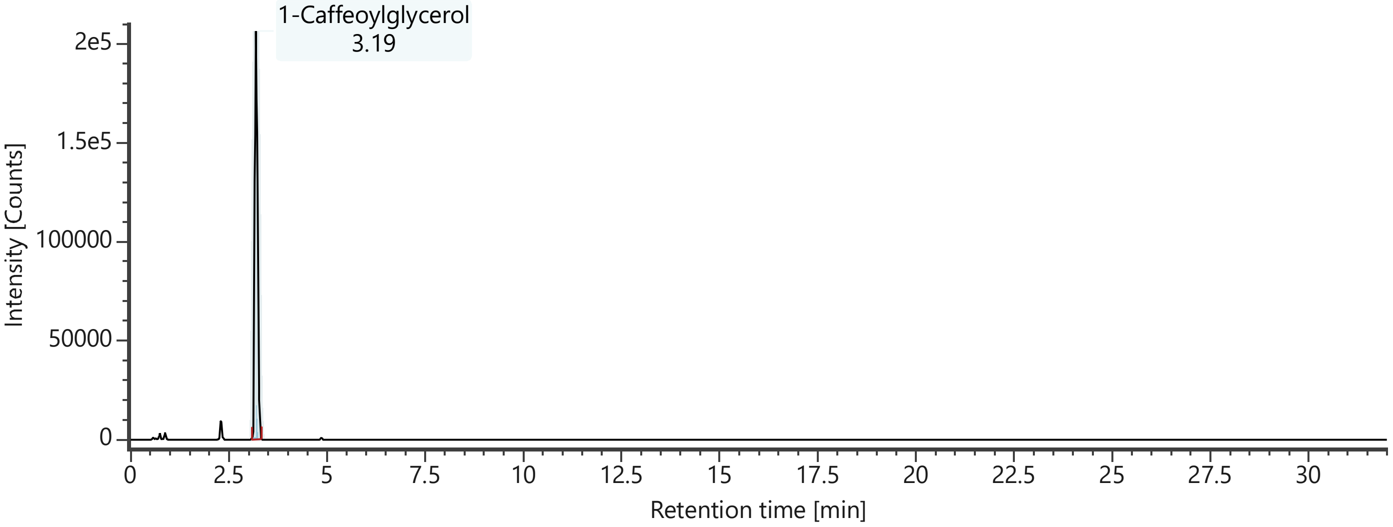

Item name: Sep257-ve
Item description: Mervat253
Channel name: Low energy : Time 3.1904 +/- 0.0222 minutes

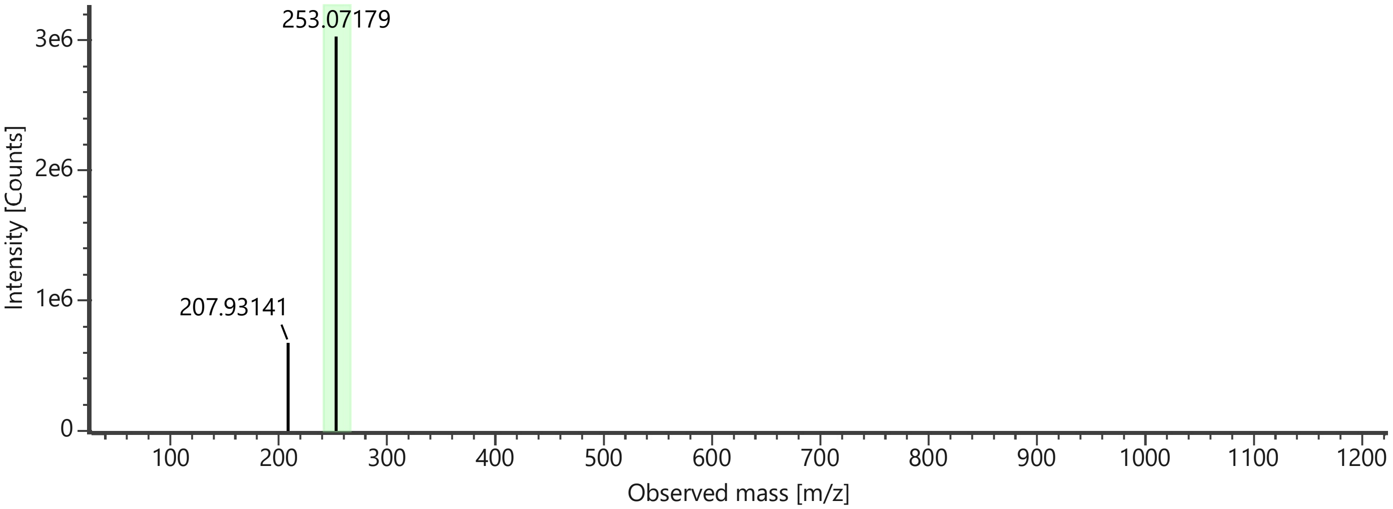

Item name: Lamiaceae family -ve mode

Created by: Ayad, Younan

Created on: Sep 18, 2025

Created time: 13:05:43 Egypt Standard Time

Item name: Sep257-ve

Item description: Mervat253

Channel name: High energy : Time 3.1904 +/- 0.0222 minutes

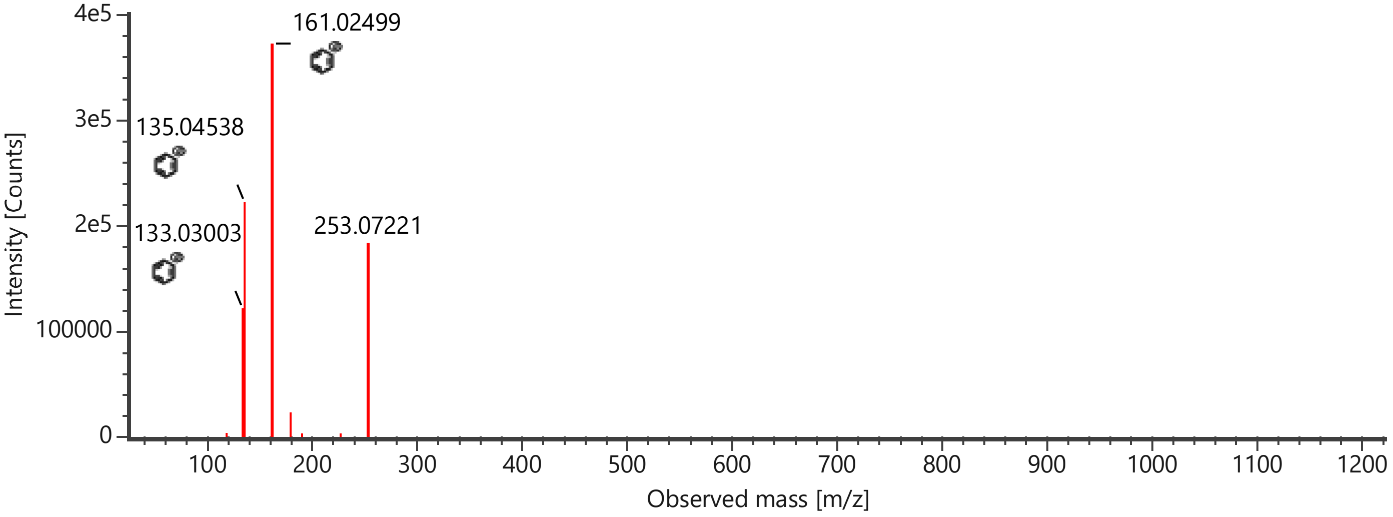

Component name: Kaempferol 3-O-β-Rutinoside

Item name: Sep257-ve
Channel name: Kaempferol 3-O-β-Rutinoside [-H] : (52.5 PPM) 593.1498

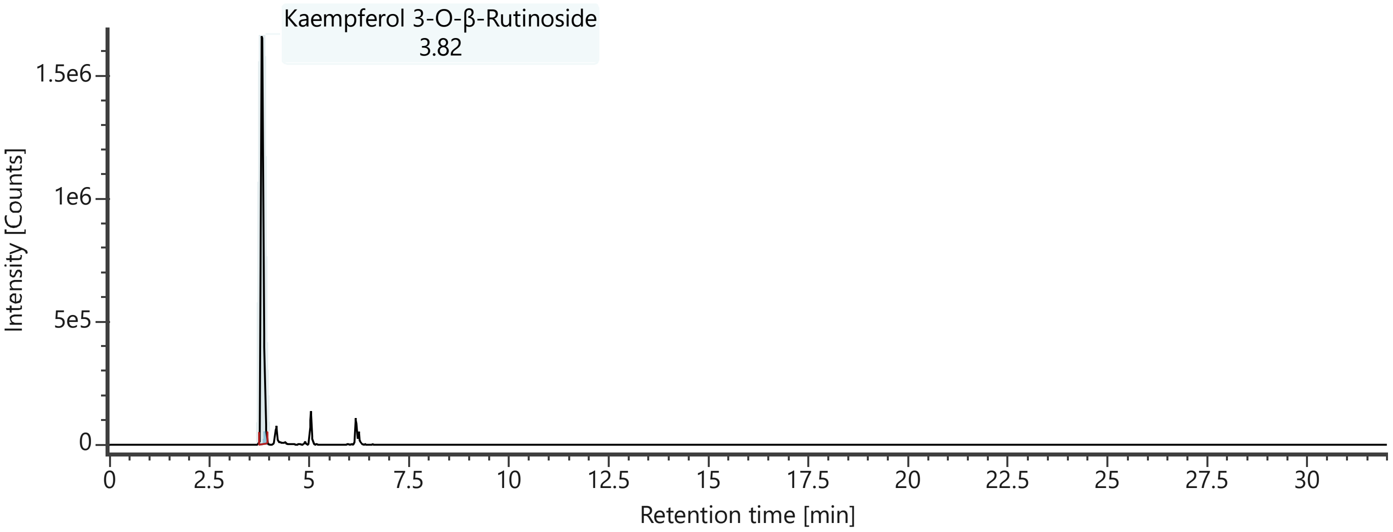

Item name: Sep257-ve
Item description: Mervat253
Channel name: Low energy : Time 3.8190 +/- 0.0222 minutes

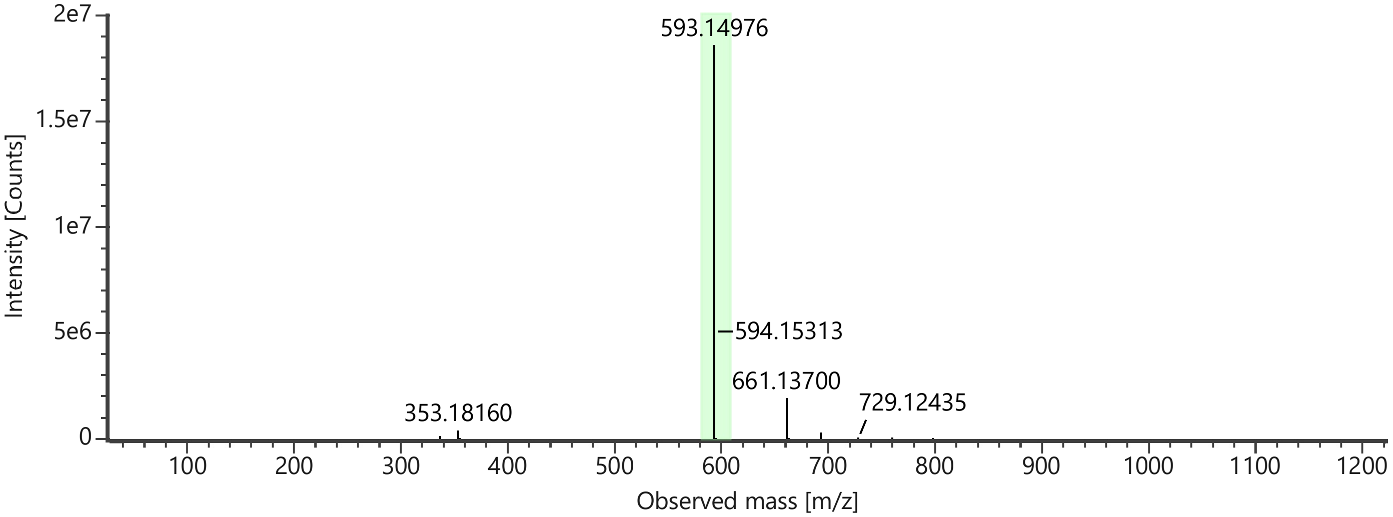

Item name: Sep257-ve
Item description: Mervat253

Channel name: High energy : Time 3.8190 +/- 0.0222 minutes

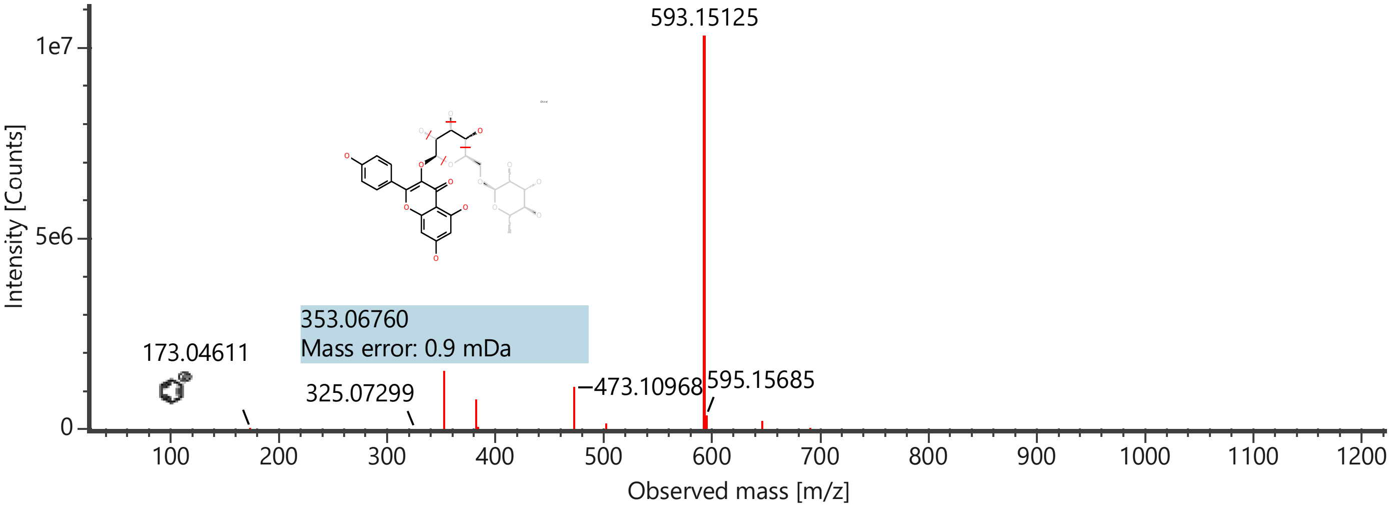

Component name: Kaempferol 3-O-β-Rutinoside

Item name: Sep257-ve
Channel name: Kaempferol 3-O-β-Rutinoside [-H] : (52.5 PPM) 593.1508

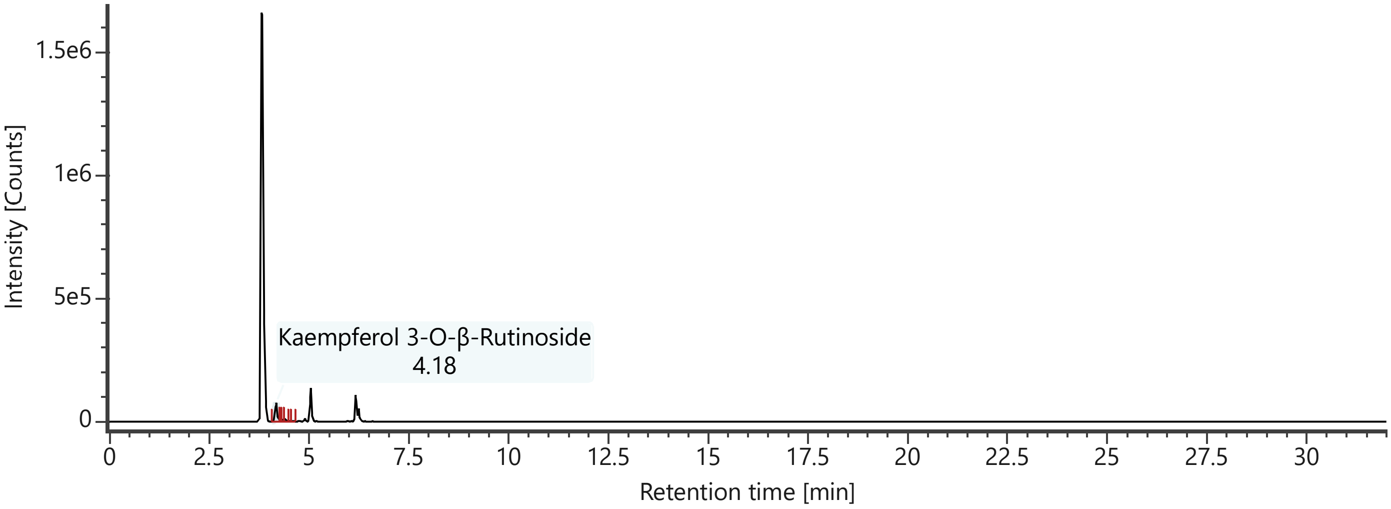

Item name: Sep257-ve
Item description: Mervat253
Channel name: Low energy : Time 4.1786 +/- 0.0222 minutes

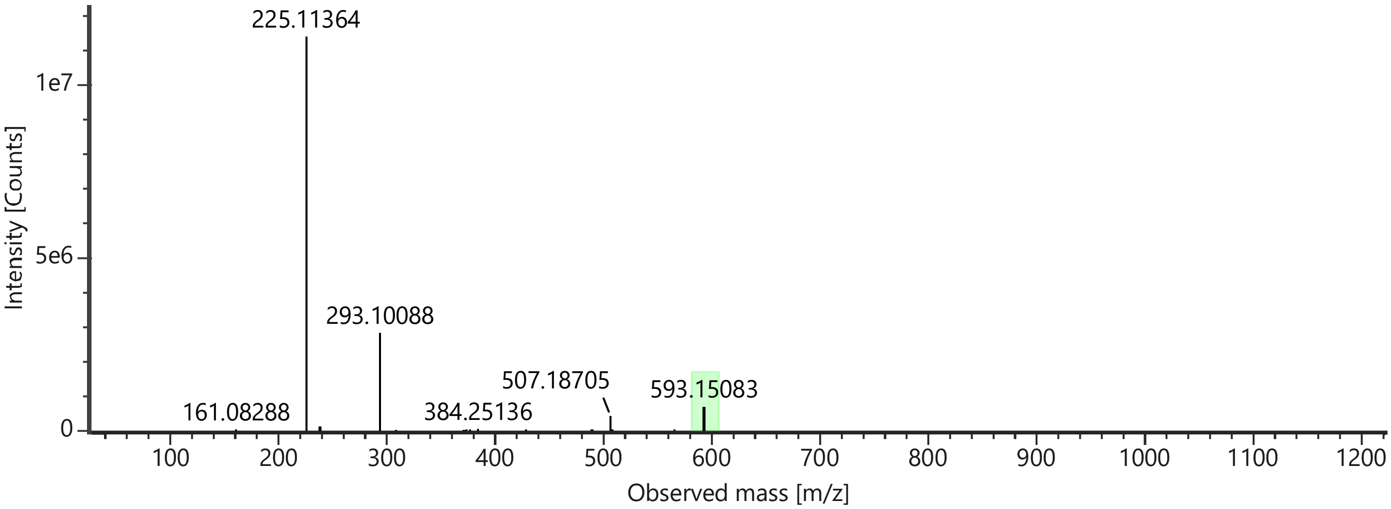

Item name: Sep257-ve
Item description: Mervat253

Channel name: High energy : Time 4.1786 +/- 0.0222 minutes

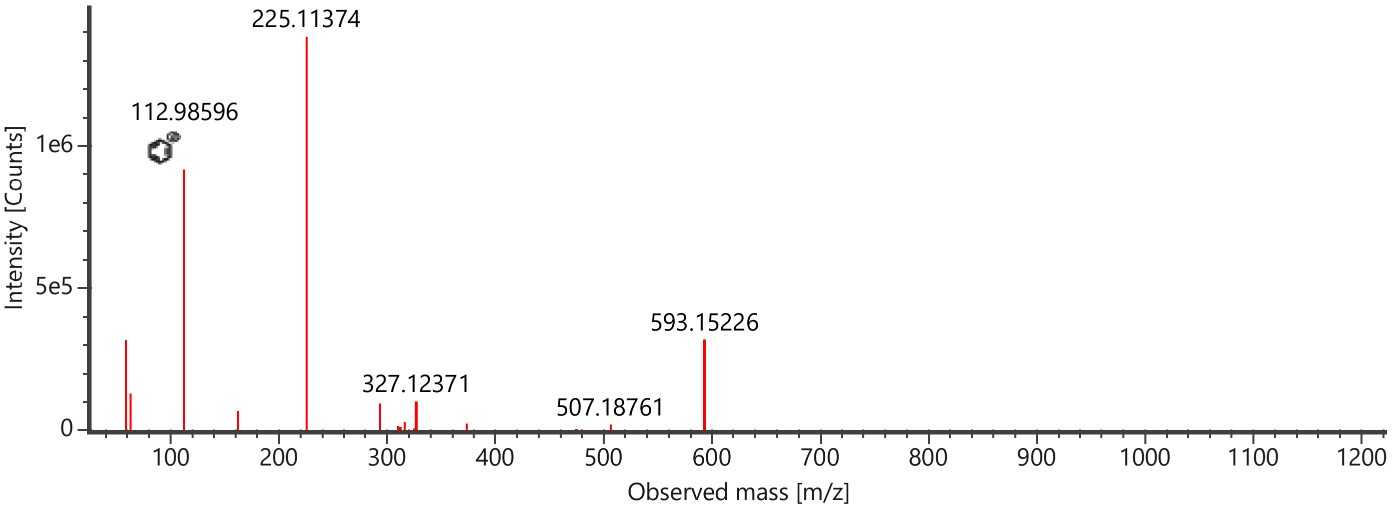

Component name: Kaempferol 3-O-α-L-rhamnosyl (1→2)β-D-xyloside

Item name: Sep257-ve
Channel name: Kaempferol 3-O-α-L-rhamnosyl (1→2)β-D-xyloside [-H] : (52.5 PPM) 563.1405

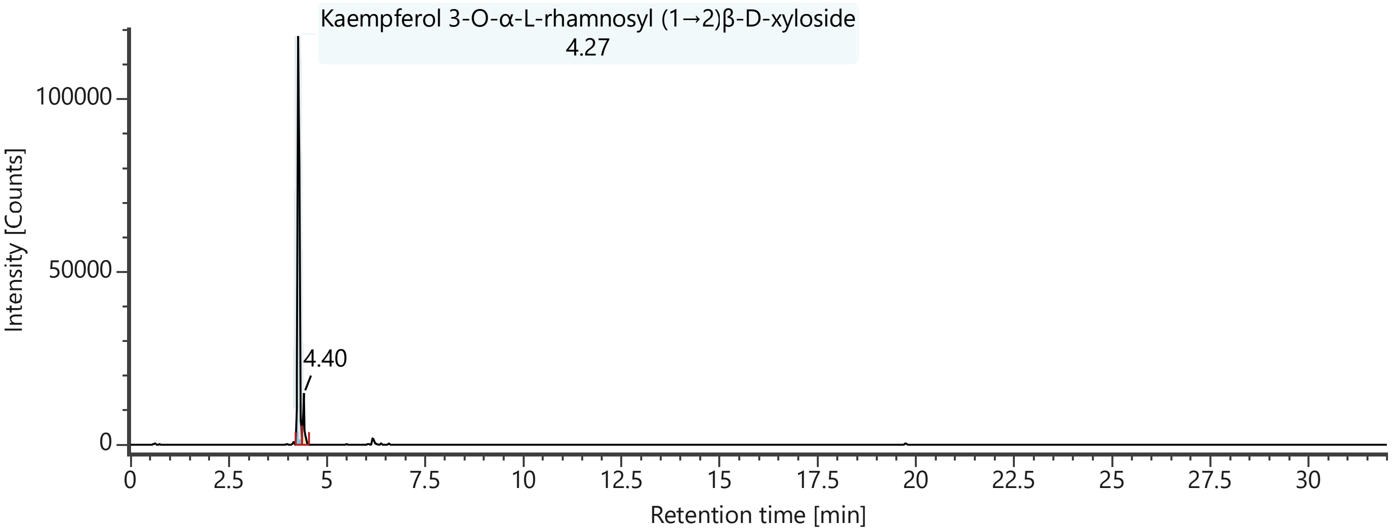

Item name: Sep257-ve
Item description: Mervat253
Channel name: Low energy : Time 4.2713 +/- 0.0222 minutes

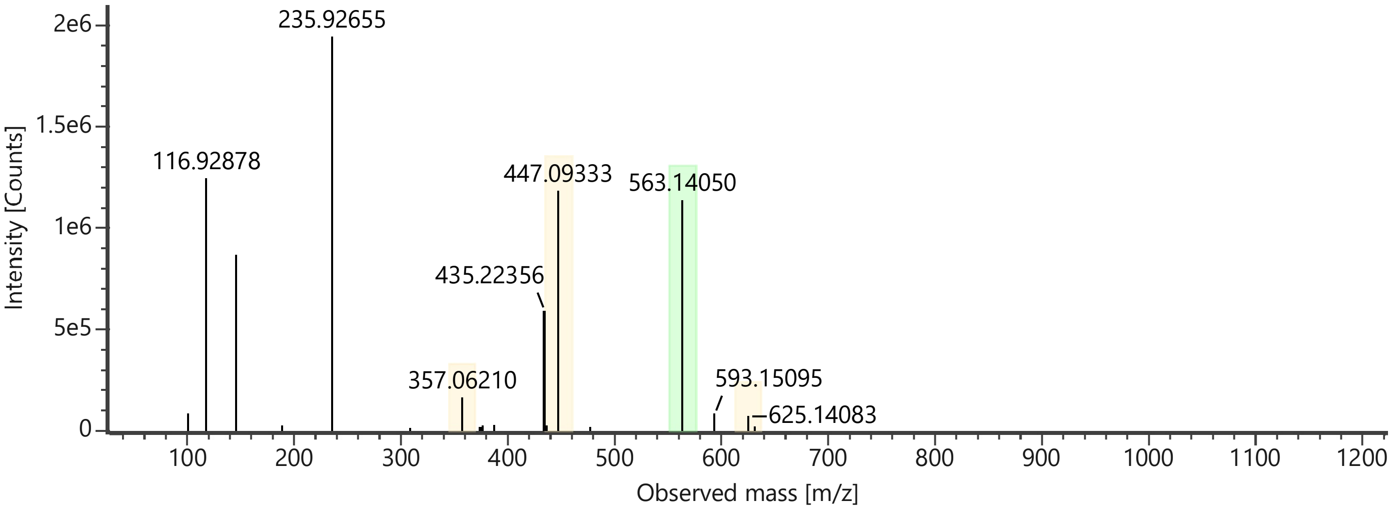

Item name: Sep257-ve
Item description: Mervat253

Channel name: High energy : Time 4.2713 +/- 0.0222 minutes

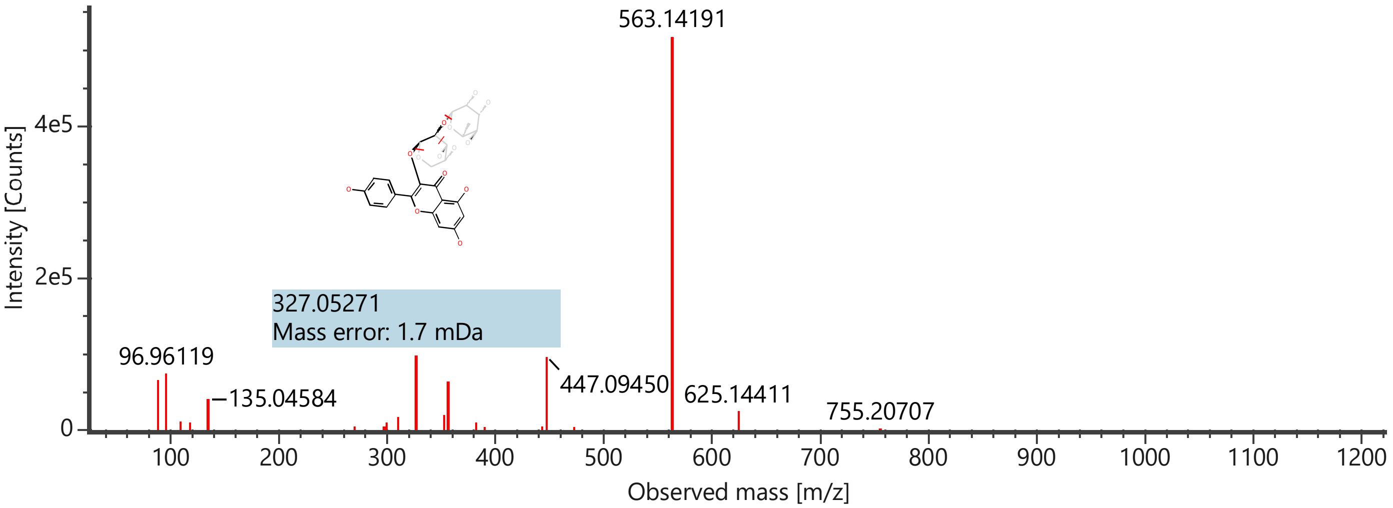

Component name: Kaempferol-3-O-β-D-galactoside

Item name: Sep257-ve
Channel name: Kaempferol-3-O-β-D-galactoside [-H] : (52.5 PPM) 447.0933

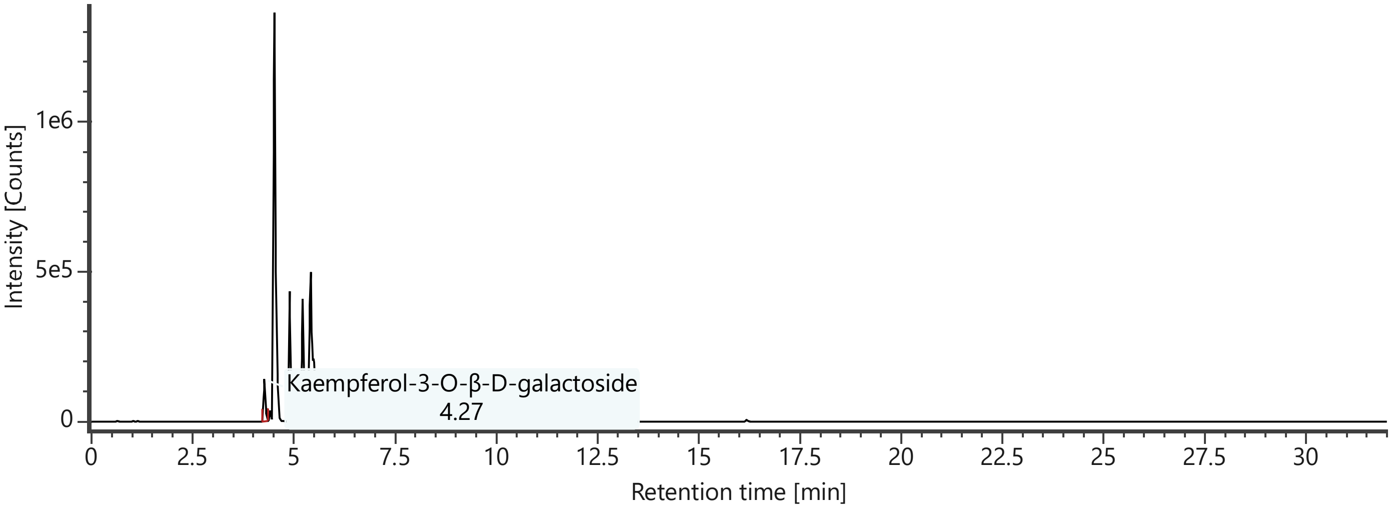

Item name: Sep257-ve
Item description: Mervat253
Channel name: Low energy : Time 4.2769 +/- 0.0222 minutes

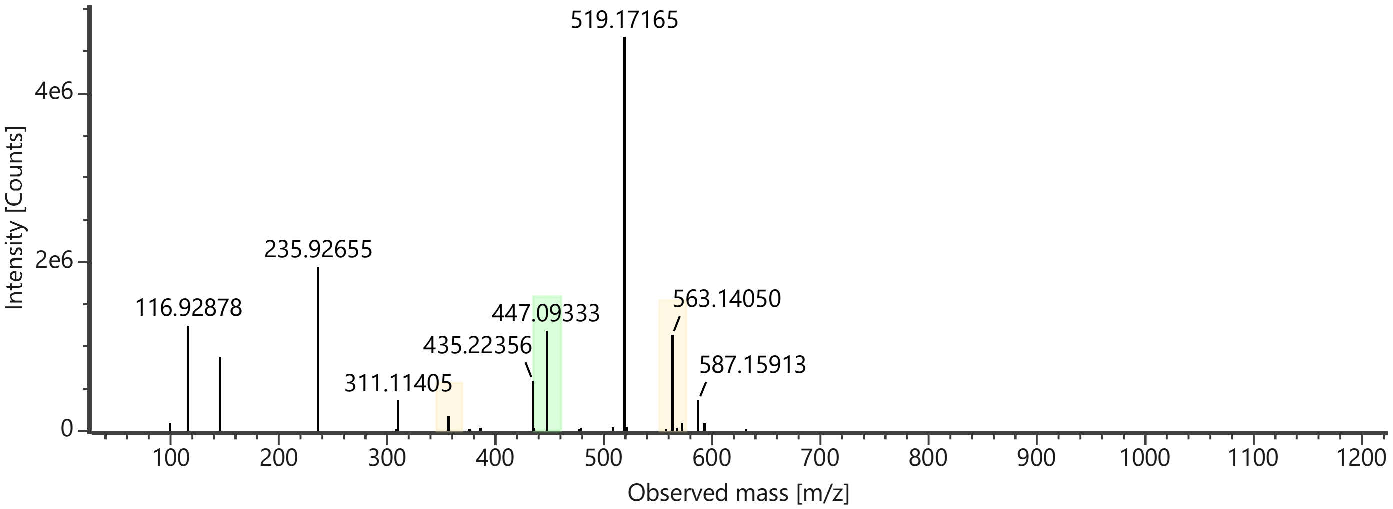

Item name: Sep257-ve
Item description: Mervat253

Channel name: High energy : Time 4.2769 +/- 0.0222 minutes

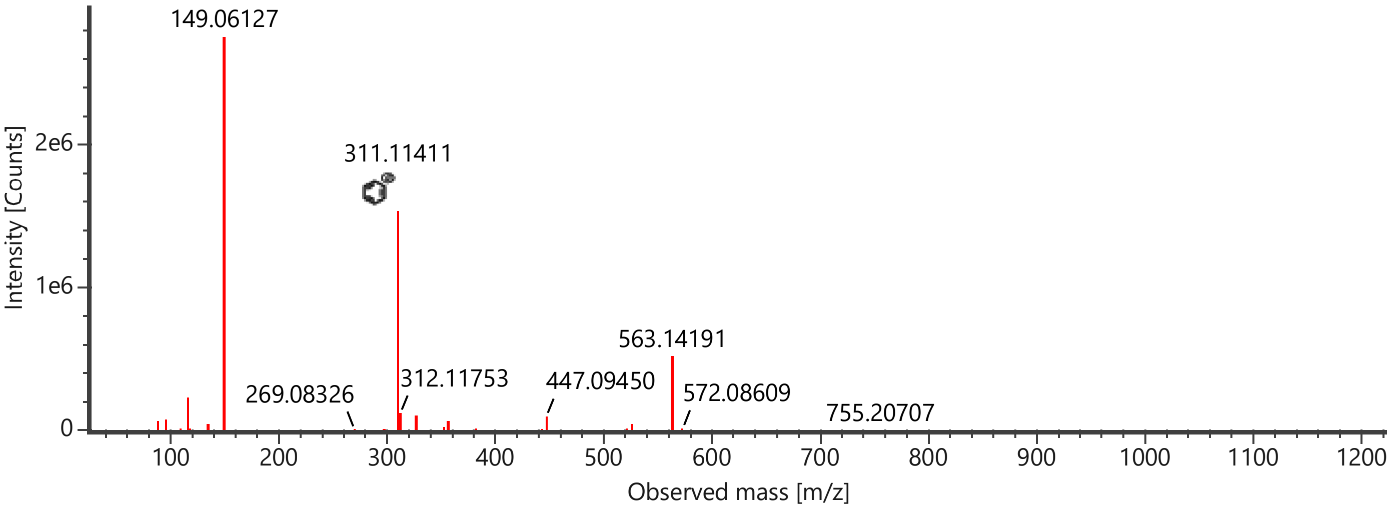

Component name: p-Coumaric acid

Item name: Sep257-ve
Channel name: p-Coumaric acid [-H] : (52.5 PPM) 163.0407

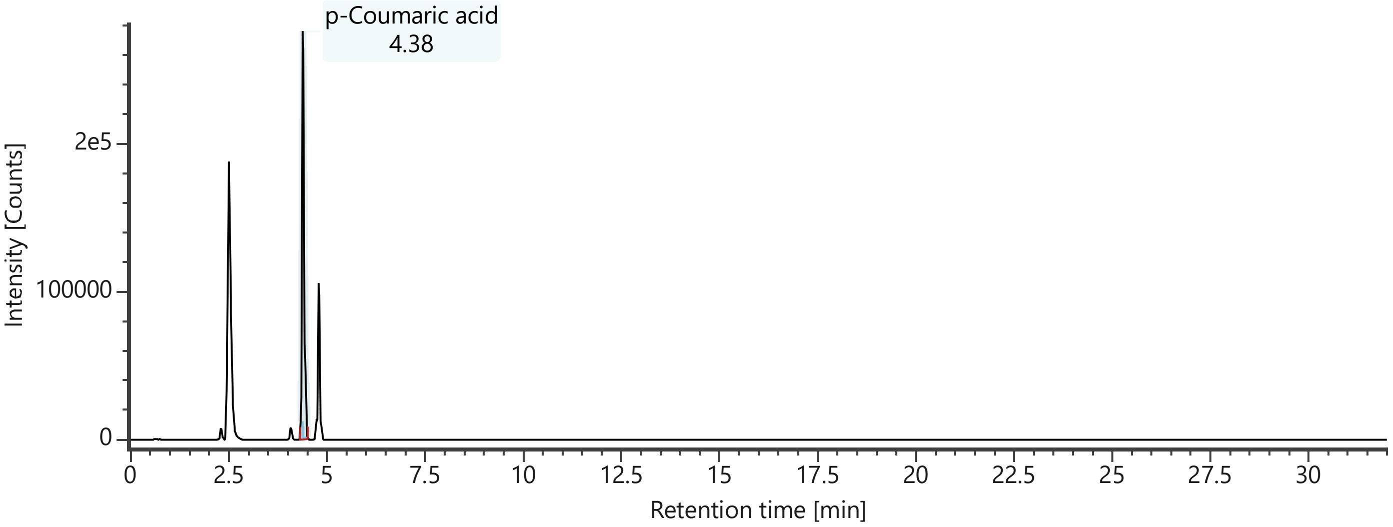

Item name: Sep257-ve
Item description: Mervat253

Channel name: Low energy : Time 4.3822 +/- 0.0222 minutes

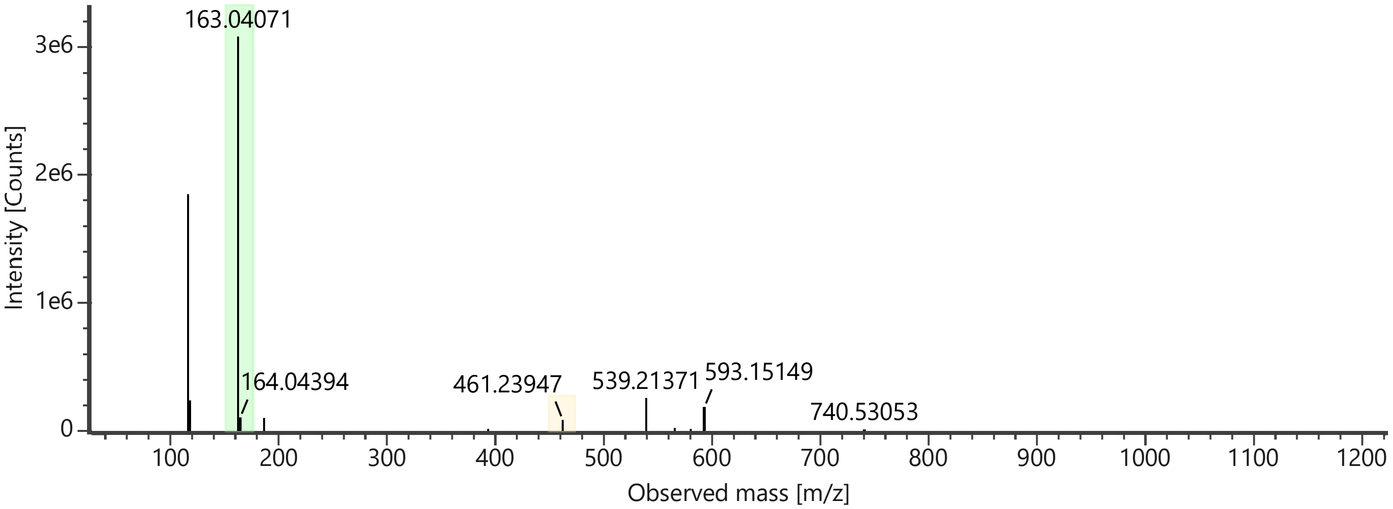

Item name: Sep257-ve
Item description: Mervat253

Channel name: High energy : Time 4.3822 +/- 0.0222 minutes

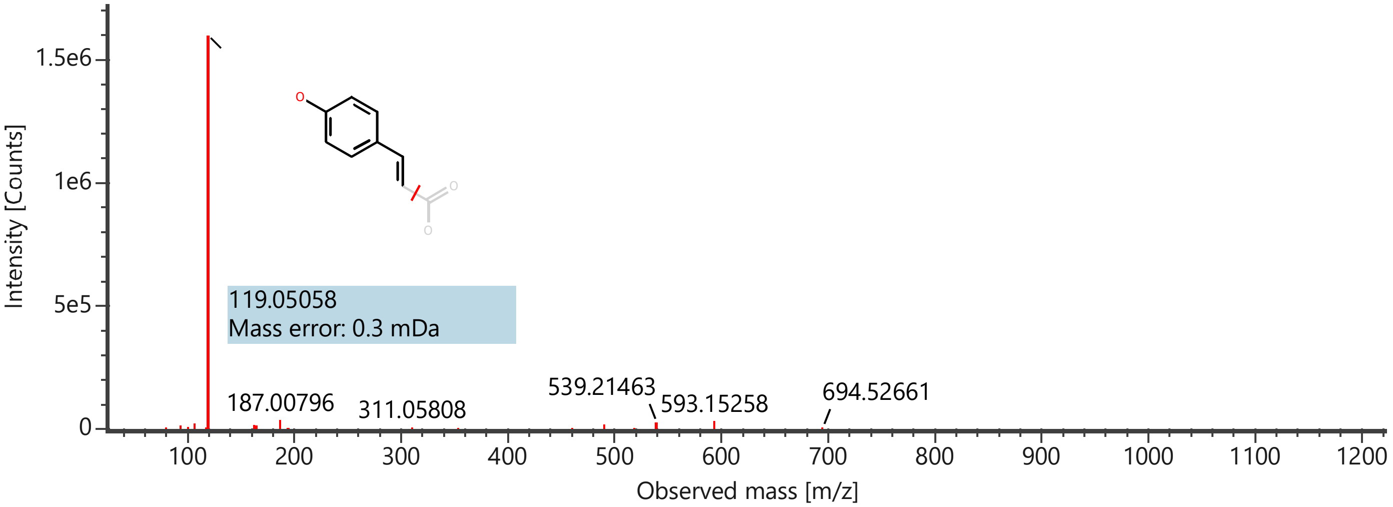

Component name: Luteolin-4'-beta-D-glucoside

Item name: Sep257-ve
Channel name: Luteolin-4'-beta-D-glucoside [-H] : (52.5 PPM) 447.0931

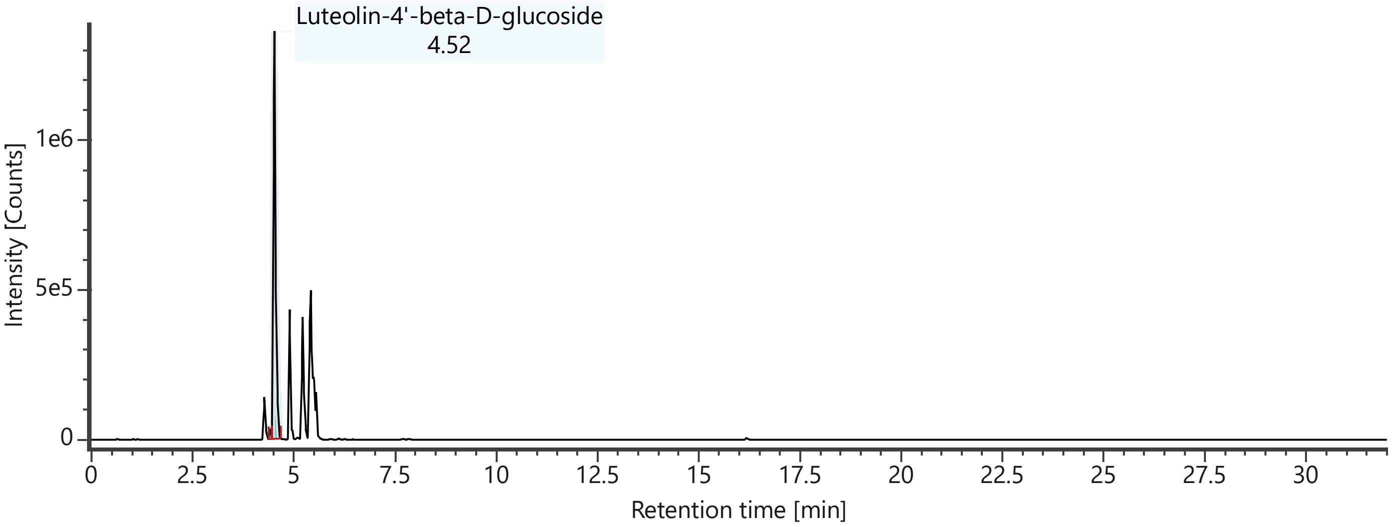

Item name: Sep257-ve
Item description: Mervat253
Channel name: Low energy : Time 4.5175 +/- 0.0222 minutes

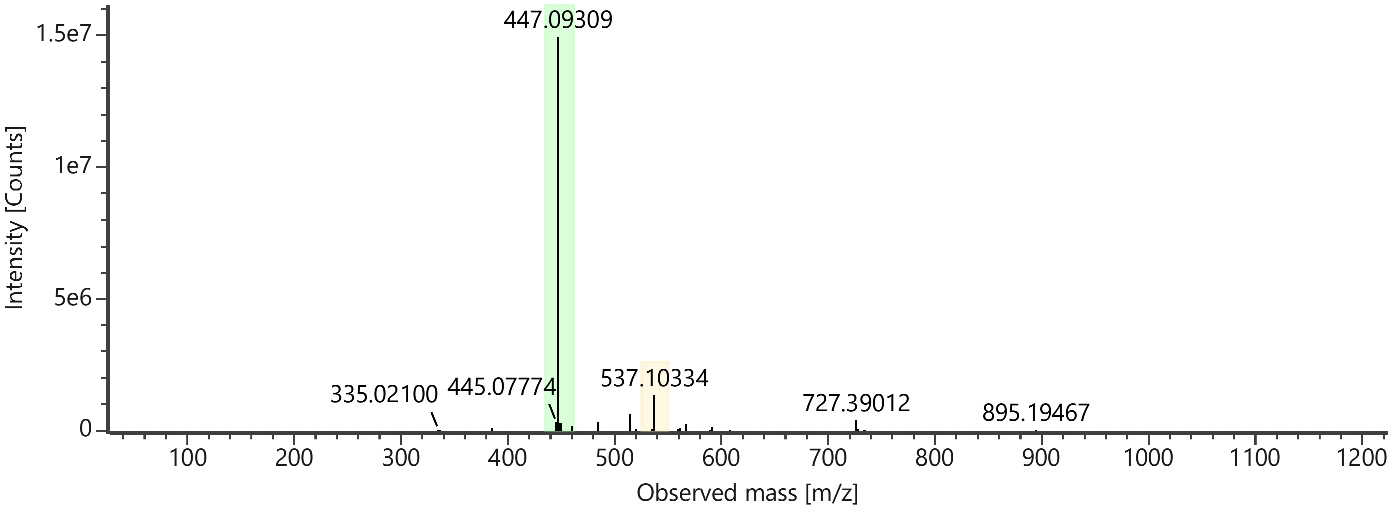

Item name: Sep257-ve
Item description: Mervat253

Channel name: High energy : Time 4.5175 +/- 0.0222 minutes

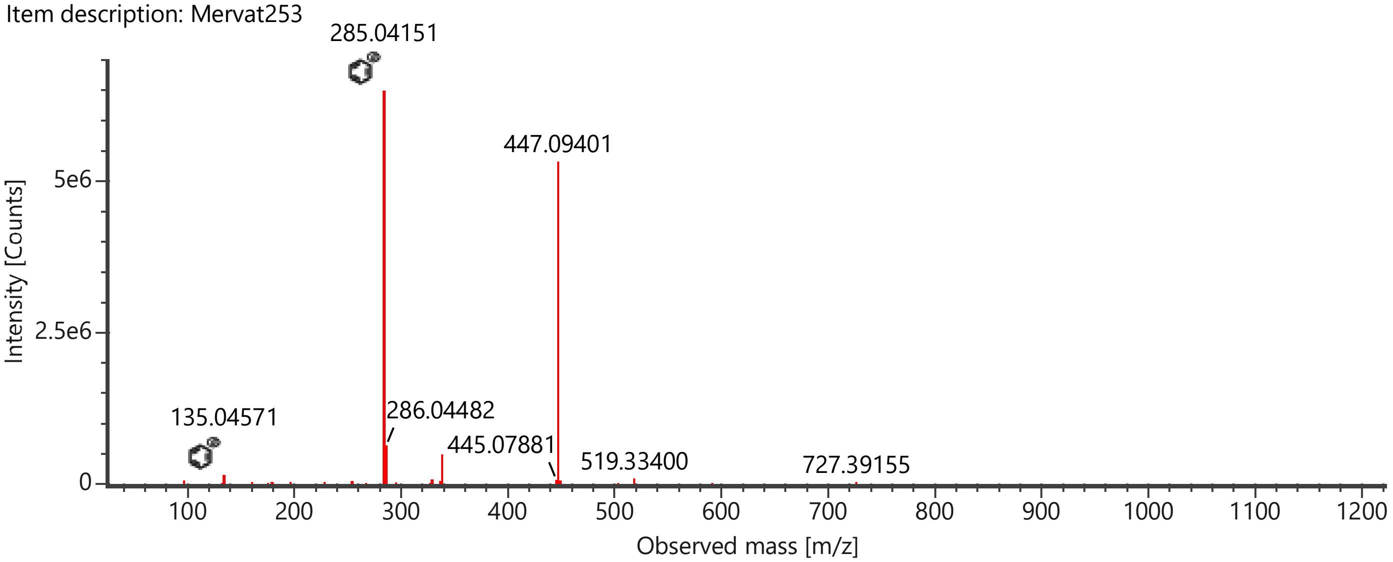

Component name: 1-[2-(3,4-Dihydroxyphenyl)-1-carboxy]ethoxycarbonyl-2-(3,4-dihydroxyphenyl)-7,8-dihydroxy-1,2-dihydronaphthalene-3-carboxylic acid

Item name: Sep257-ve
Channel name: 1-[2-(3,4-Dihydroxyphenyl)-1-carboxy]ethoxycarbonyl-2-(3,4-dihydroxyphenyl)-7,8-dihydroxy-1,2-dihydronaphthalene-3-carboxylic acid [-H] : (52.5 PPM) 537.1033

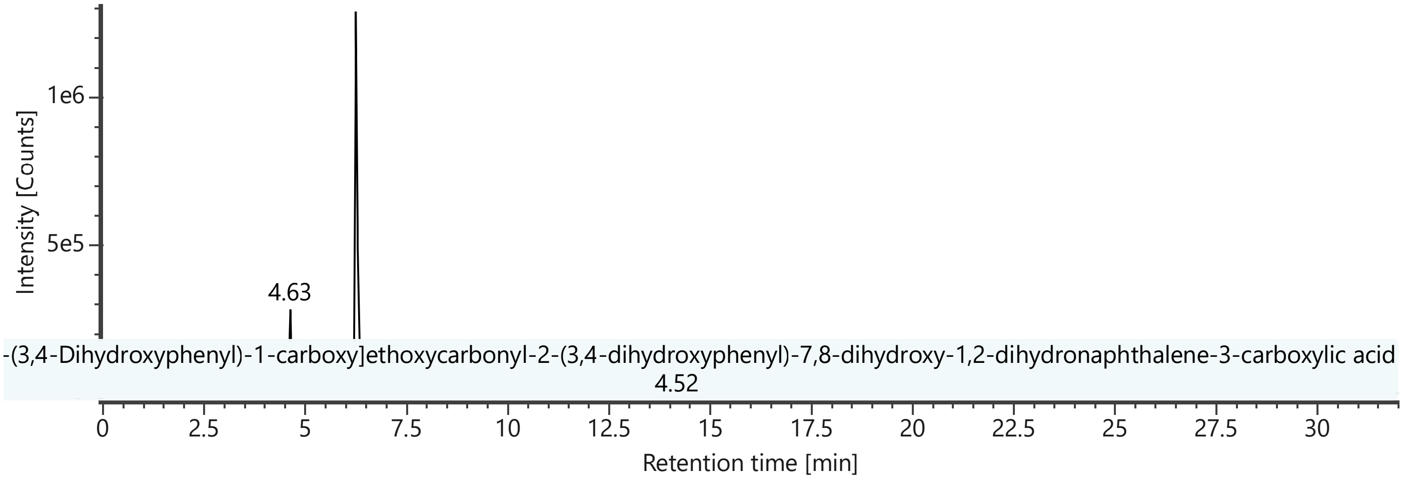

Item name: Sep257-ve
Item description: Mervat253
Channel name: Low energy : Time 4.5193 +/- 0.0222 minutes

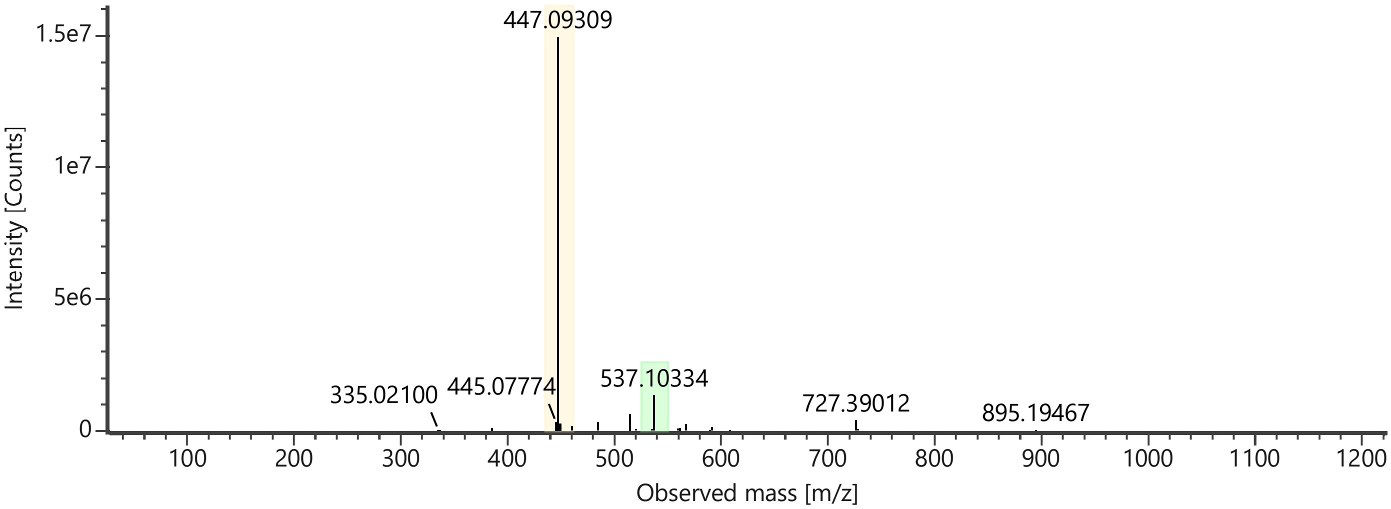

Item name: Sep257-ve
Item description: Mervat253

Channel name: High energy : Time 4.5193 +/- 0.0222 minutes

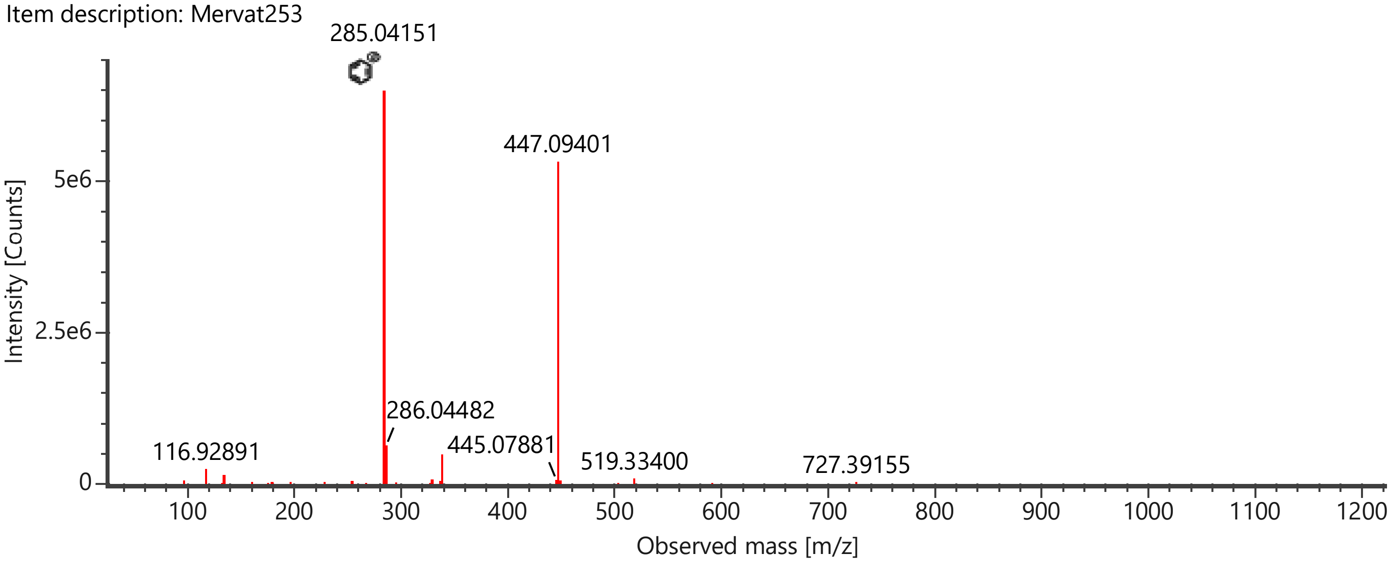

Component name: **1-[2-(3,4-Dihydroxyphenyl)-1-carboxy]ethoxycarbonyl-2-(3,4-dihydroxyphenyl)-7,8-dihydroxy-1,2-dihydronaphthalene-3-carboxylic acid**

Item name: Sep257-ve
Channel name: 1-[2-(3,4-Dihydroxyphenyl)-1-carboxy]ethoxycarbonyl-2-(3,4-dihydroxyphenyl)-7,8-dihydroxy-1,2-dihydronaphthalene-3-carboxylic acid [-H] : (52.5 PPM) 537.1040

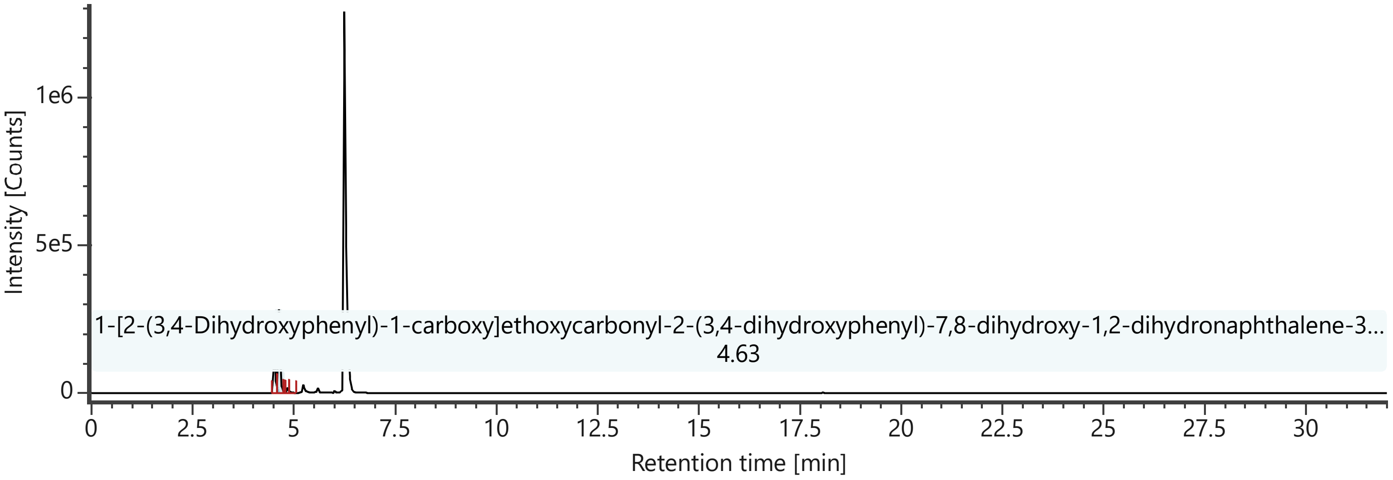

Item name: Sep257-ve
Item description: Mervat253
Channel name: Low energy : Time 4.6294 +/- 0.0222 minutes

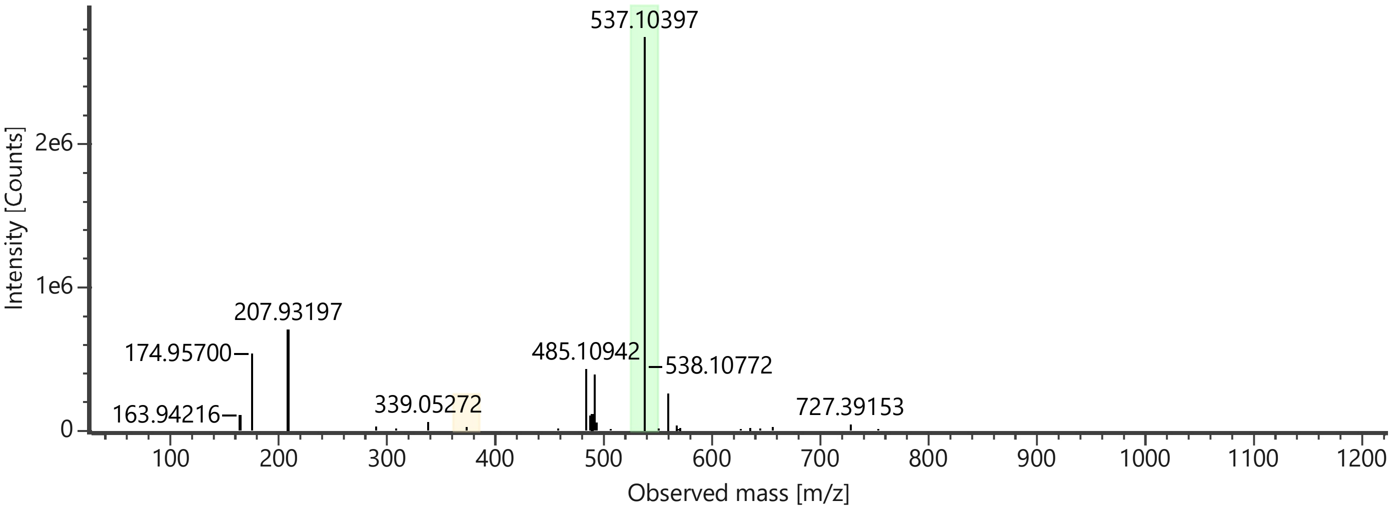

Item name: Sep257-ve
Item description: Mervat253

Channel name: High energy : Time 4.6294 +/- 0.0222 minutes

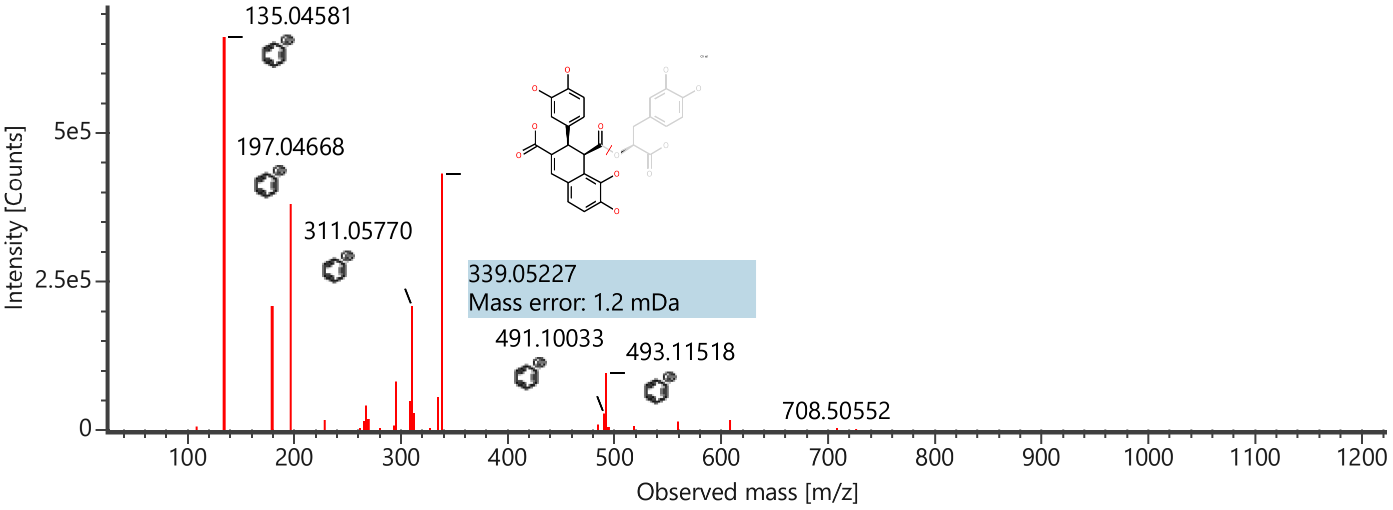

Component name: Kaempferol 7-O-β-D-glucopyranosyl(1→4)β-D-glucopyranoside

Item name: Sep257-ve
Channel name: Kaempferol 7-O-β-D-glucopyranosyl(1→4)β-D-glucopyranoside [-H] : (52.5 PPM) 609.1465

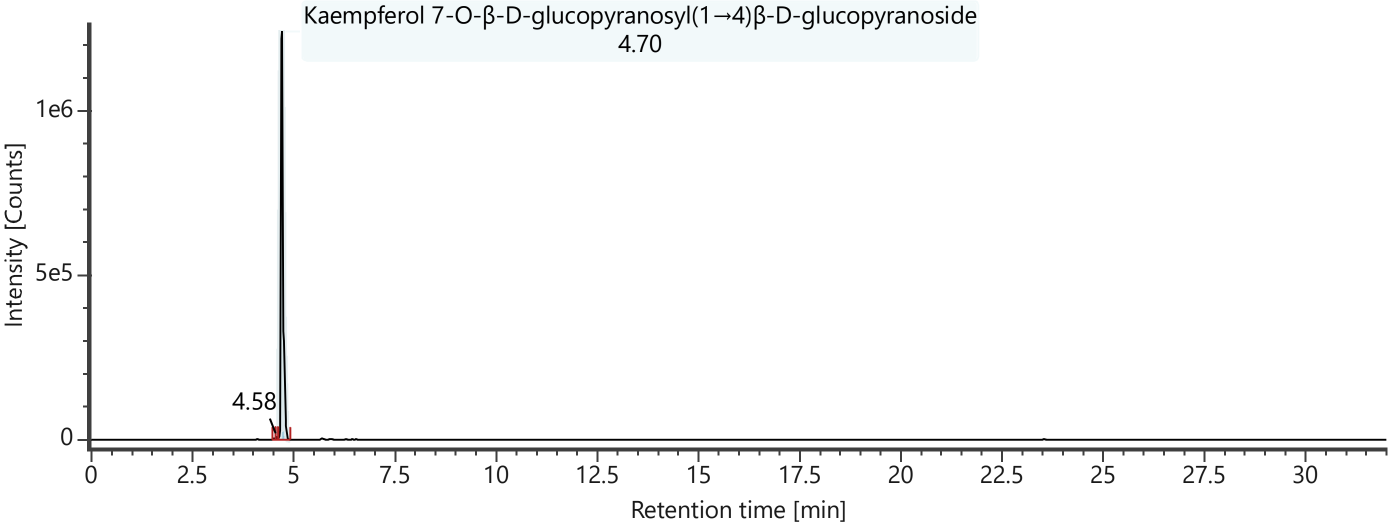

Item name: Sep257-ve
Item description: Mervat253
Channel name: Low energy : Time 4.7035 +/- 0.0222 minutes

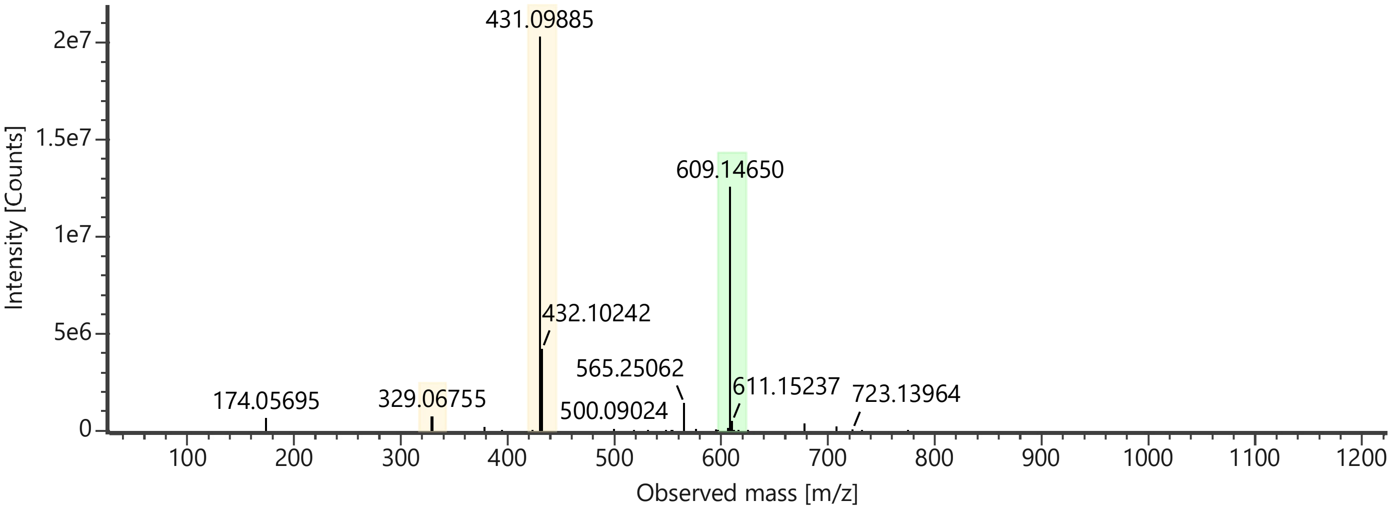

Item name: Sep257-ve
Item description: Mervat253

Channel name: High energy : Time 4.7035 +/- 0.0222 minutes

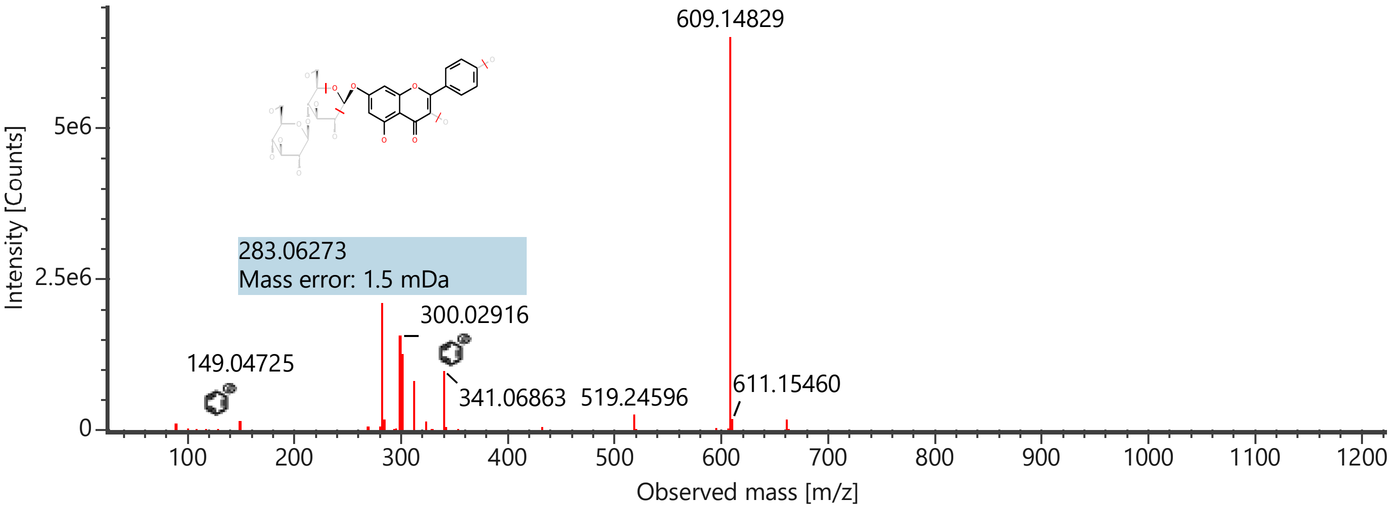

Component name: Kaempferol-7-O-α-L-rhamnoside

Item name: Sep257-ve
Channel name: Kaempferol-7-O-α-L-rhamnoside [-H] : (52.5 PPM) 431.0989

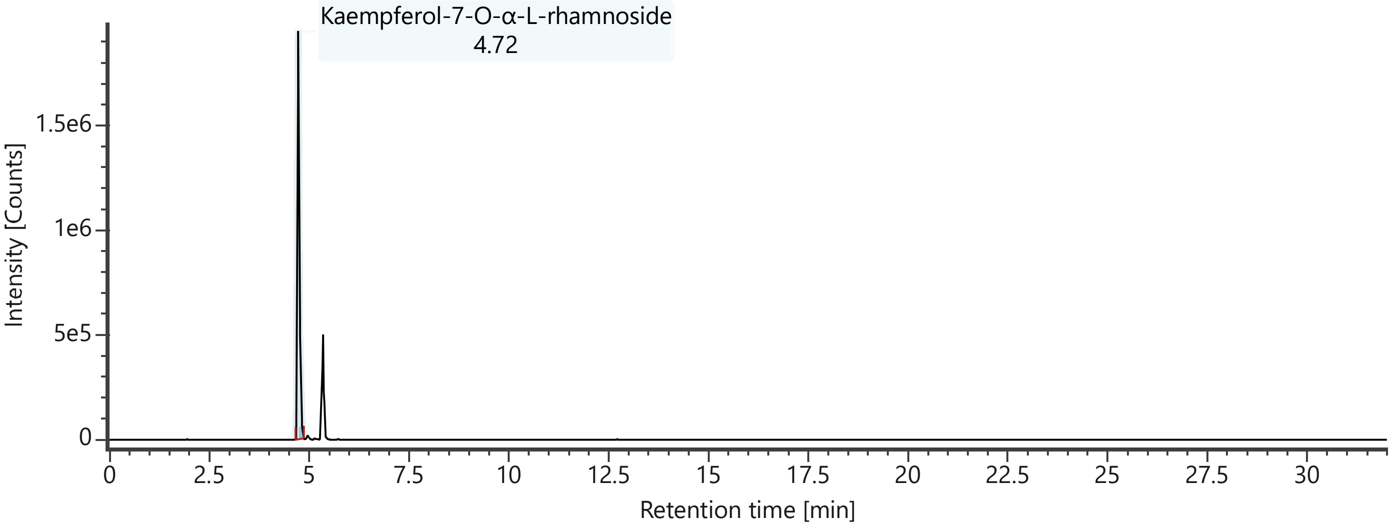

Item name: Sep257-ve
Item description: Mervat253
Channel name: Low energy : Time 4.7254 +/- 0.0222 minutes

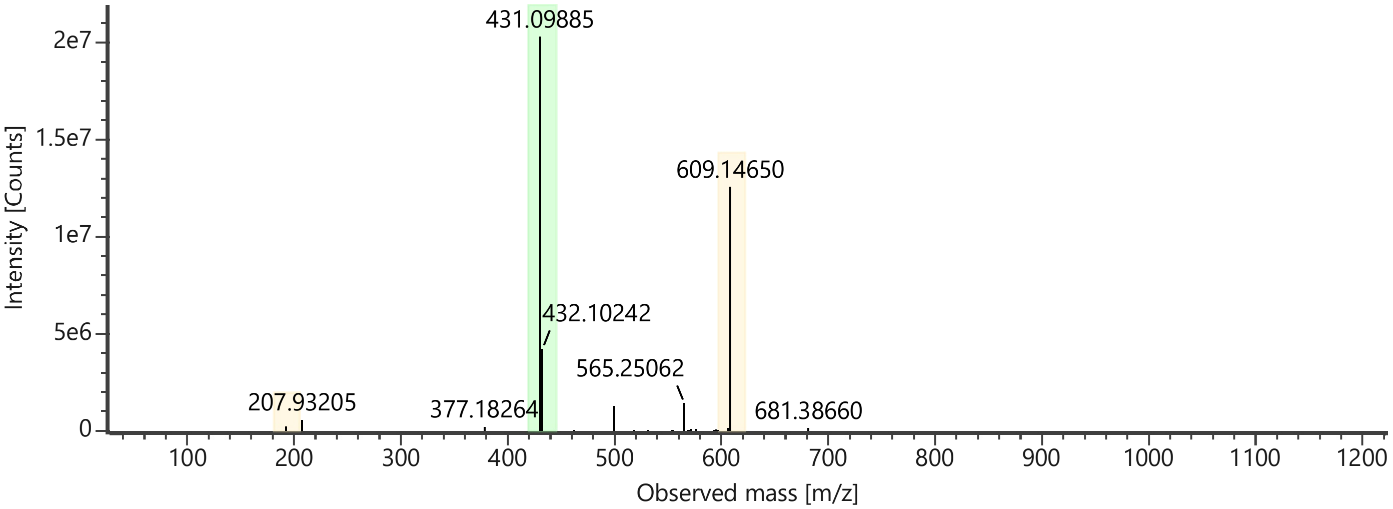

Created by: Ayad, Younan

Created on: Sep 18, 2025

Item name: Lamiaceae family -ve mode

Created time: 13:05:43 Egypt Standard Time

Item name: Sep257-ve

Item description: Mervat253

Channel name: High energy : Time 4.7254 +/- 0.0222 minutes

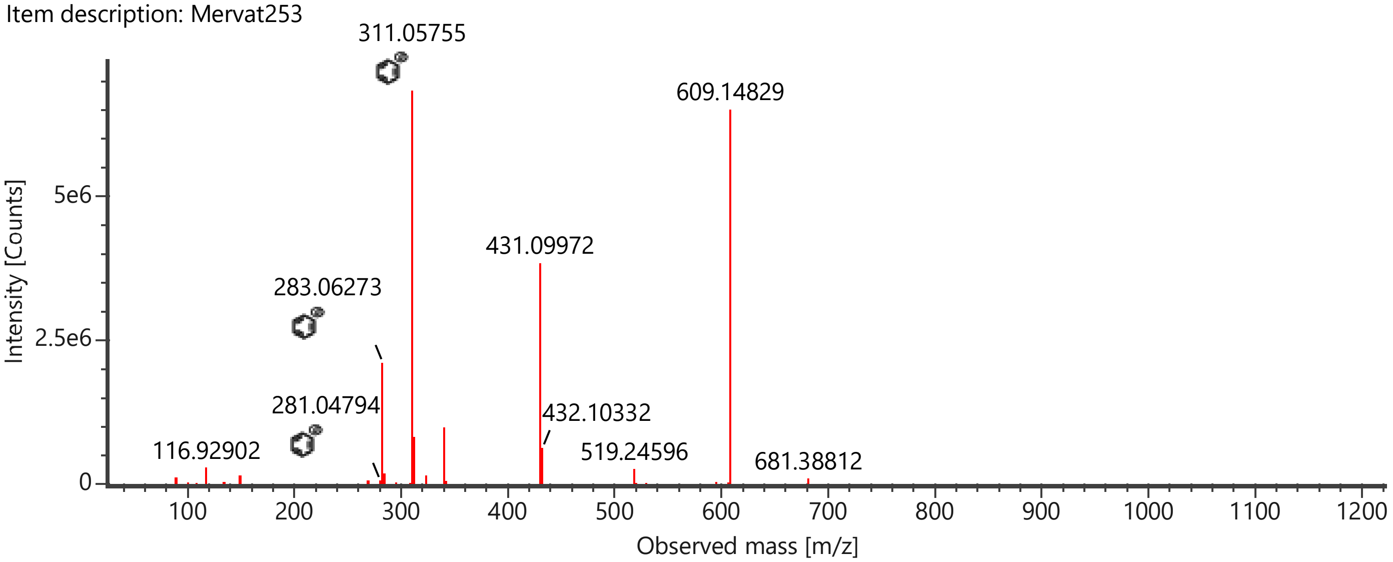

Component name: Quercetin-4'-O-β-D-galactoside

Item name: Sep257-ve
Channel name: Quercetin-4'-O-β-D-galactoside [-H] : (52.5 PPM) 463.0884

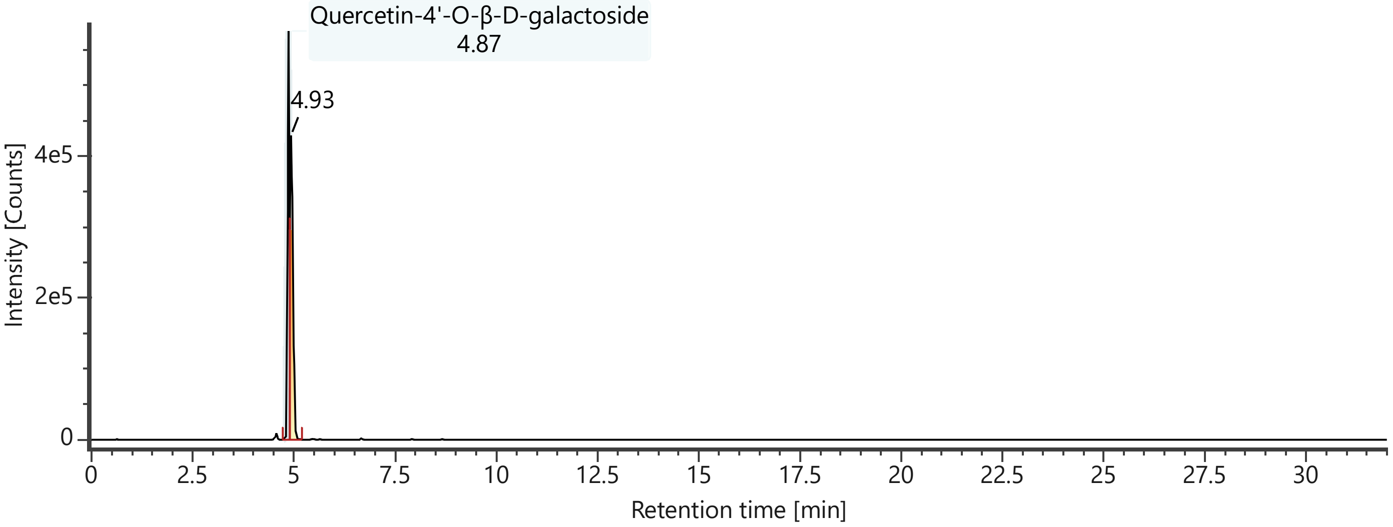

Item name: Sep257-ve
Item description: Mervat253
Channel name: Low energy : Time 4.8660 +/- 0.0222 minutes

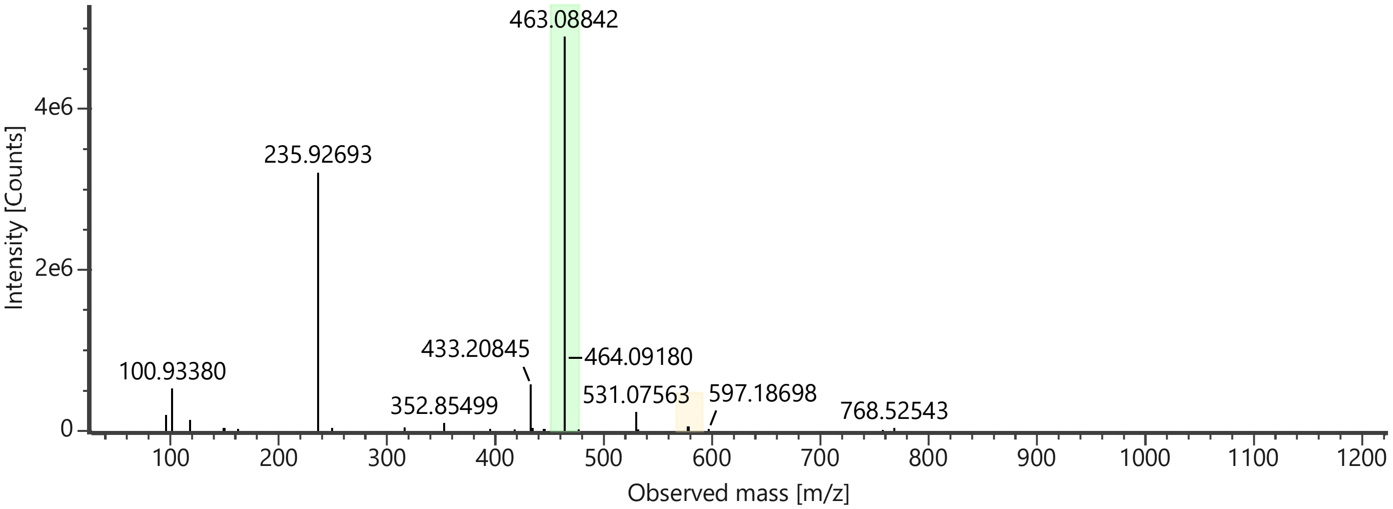

Item name: Sep257-ve
Item description: Mervat253

Channel name: High energy : Time 4.8660 +/- 0.0222 minutes

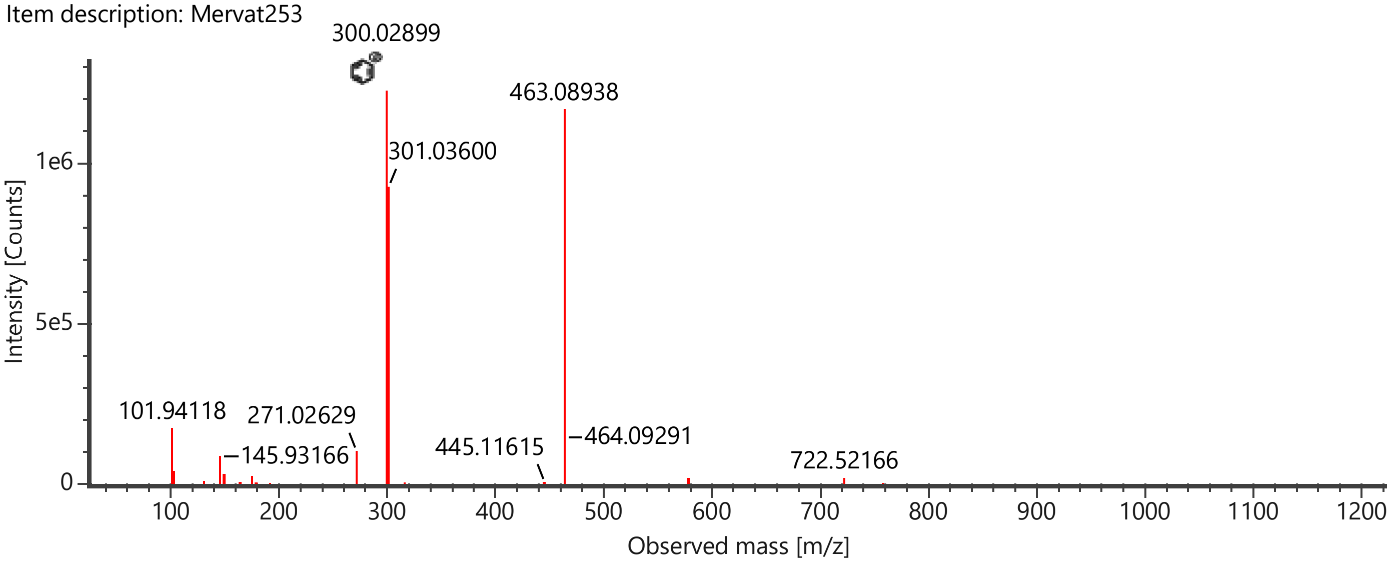

Component name: Luteolin 3'-glucoside

Item name: Sep257-ve
Channel name: Luteolin 3'-glucoside [-H] : (52.5 PPM) 447.0941

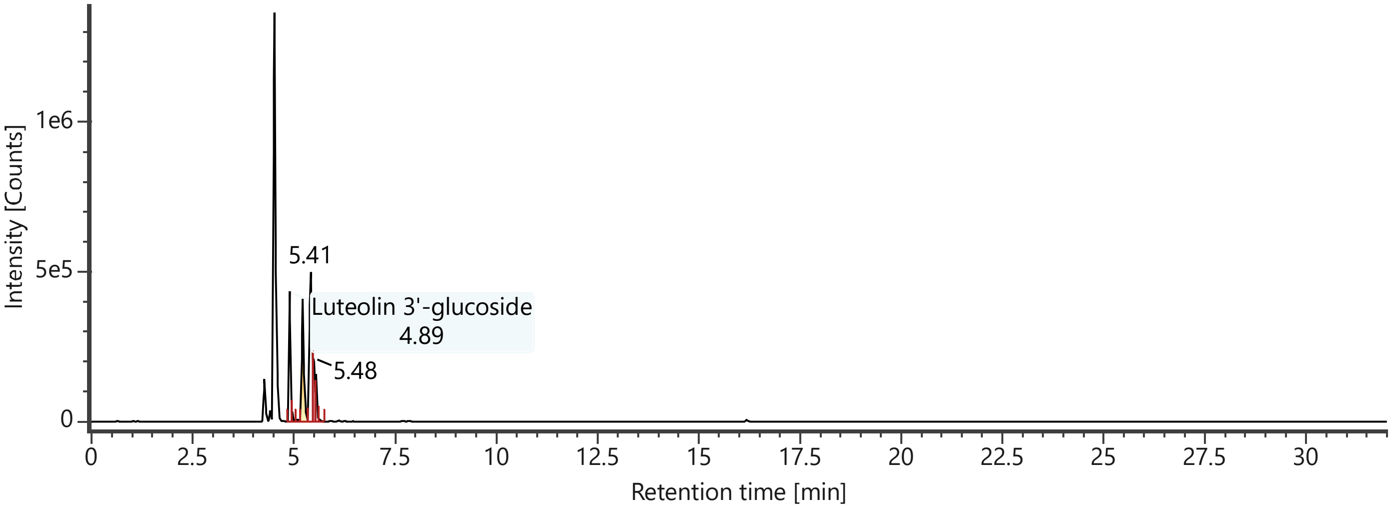

Item name: Sep257-ve
Item description: Mervat253
Channel name: Low energy : Time 4.8946 +/- 0.0222 minutes

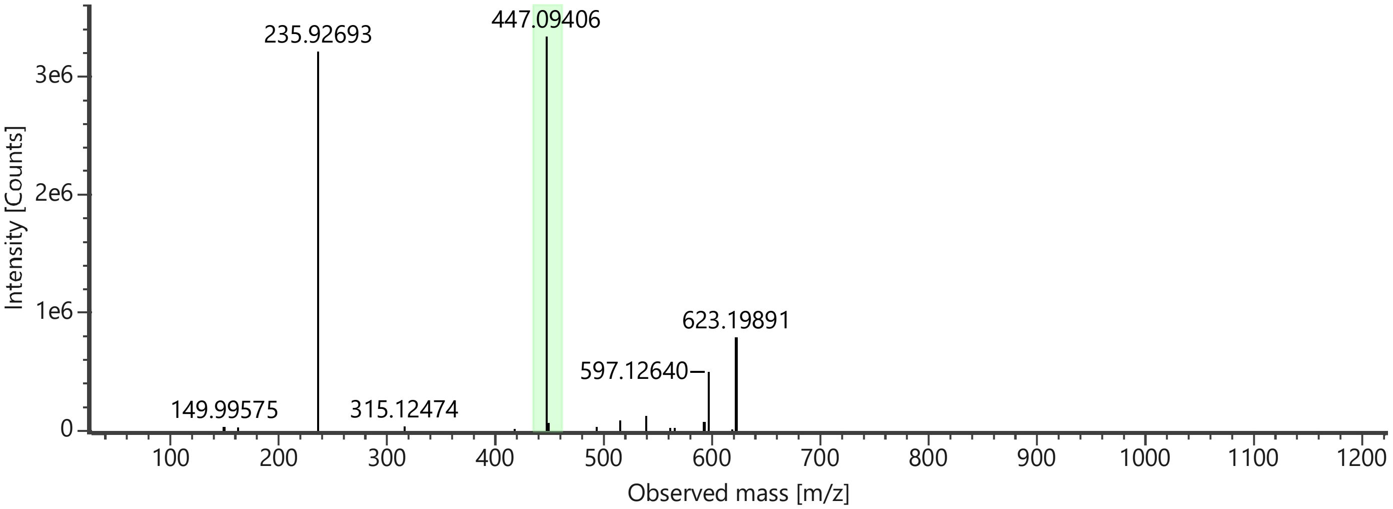

Item name: Sep257-ve
Item description: Mervat253

Channel name: High energy : Time 4.8946 +/- 0.0222 minutes

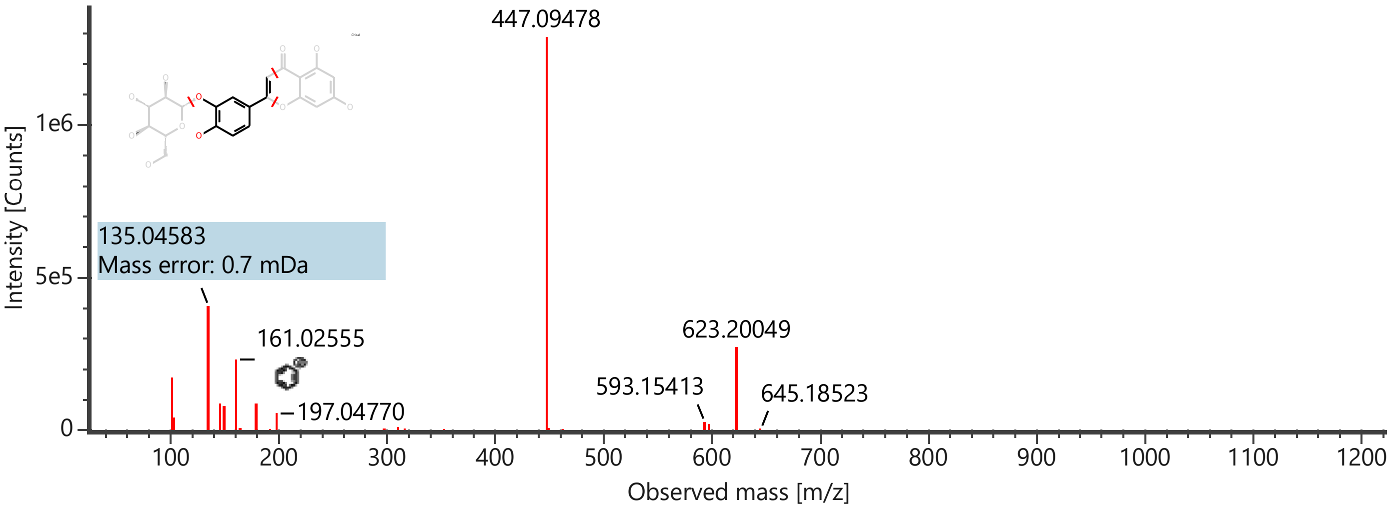

Component name: Kaempferol

Item name: Sep257-ve
Channel name: Kaempferol [-H] : (52.5 PPM) 285.0414

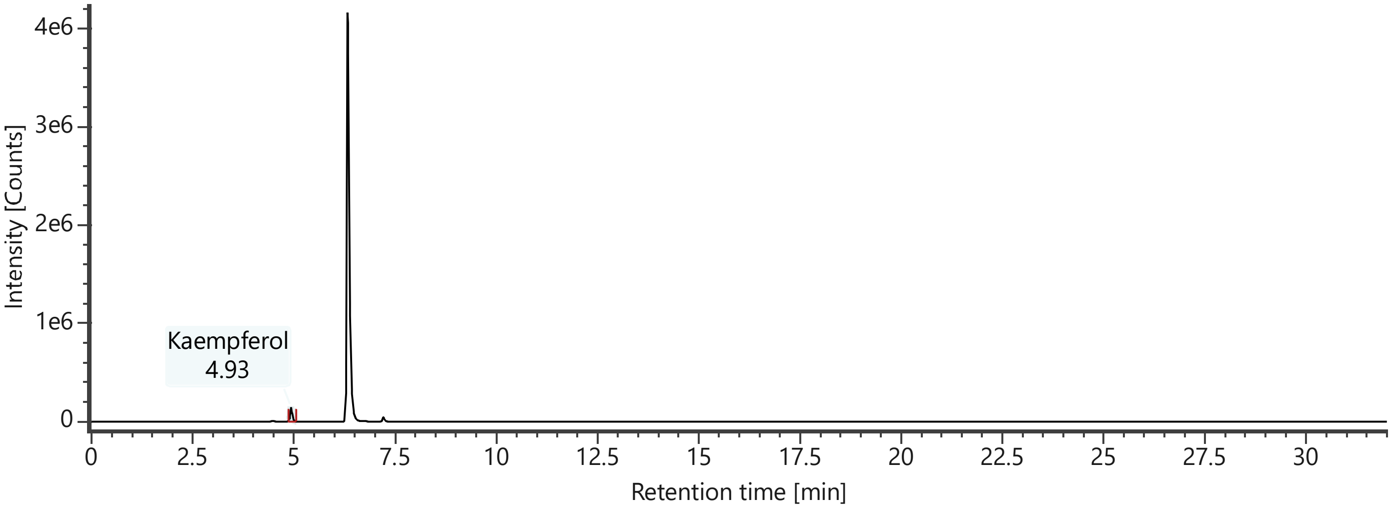

Item name: Sep257-ve
Item description: Mervat253
Channel name: Low energy : Time 4.9330 +/- 0.0222 minutes

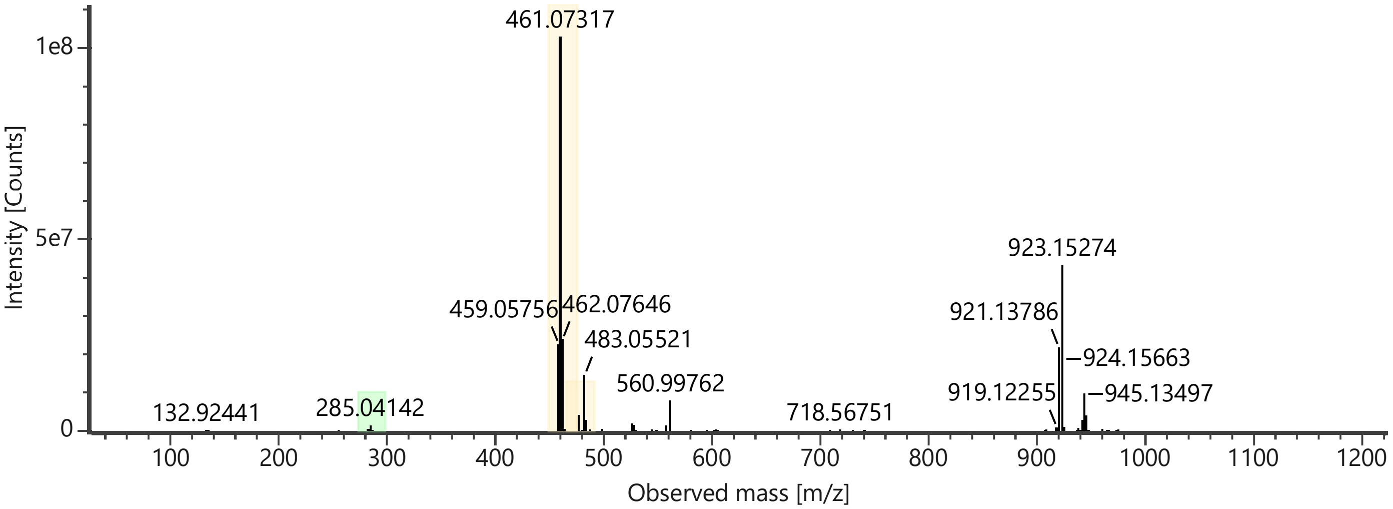

Created by: Ayad, Younan

Created on: Sep 18, 2025

Item name: Lamiaceae family -ve mode

Created time: 13:05:43 Egypt Standard Time

Item name: Sep257-ve
Item description: Mervat253

Channel name: High energy : Time 4.9330 +/- 0.0222 minutes

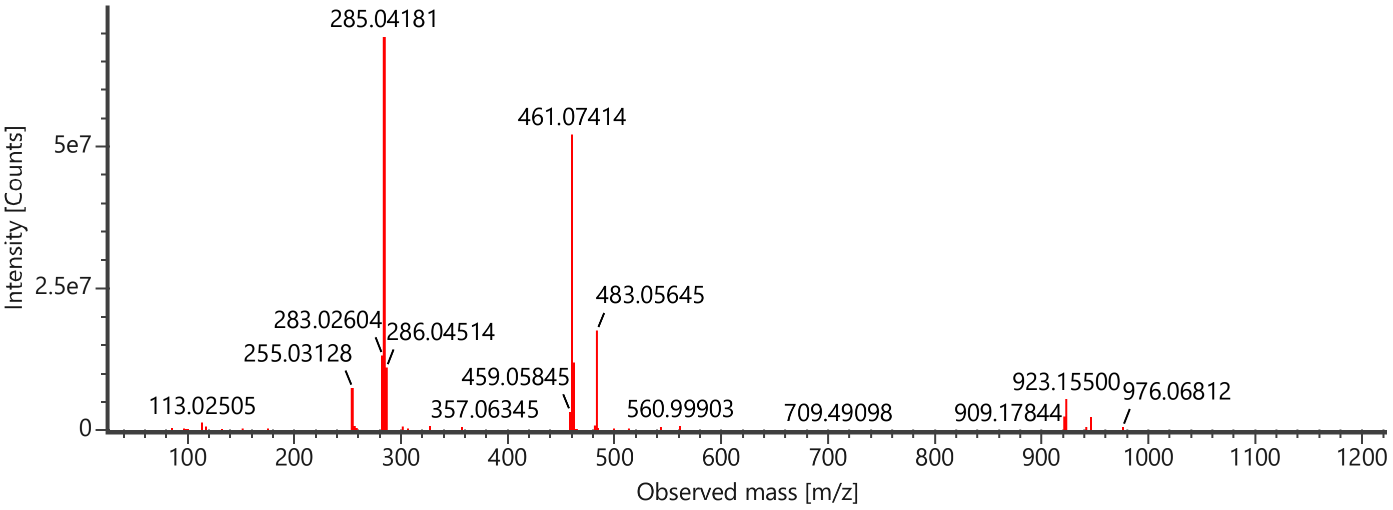

Component name: Luteolin 7-O-glucuronide

Item name: Sep257-ve
Channel name: Luteolin 7-O-glucuronide [-H] : (52.5 PPM) 461.0732

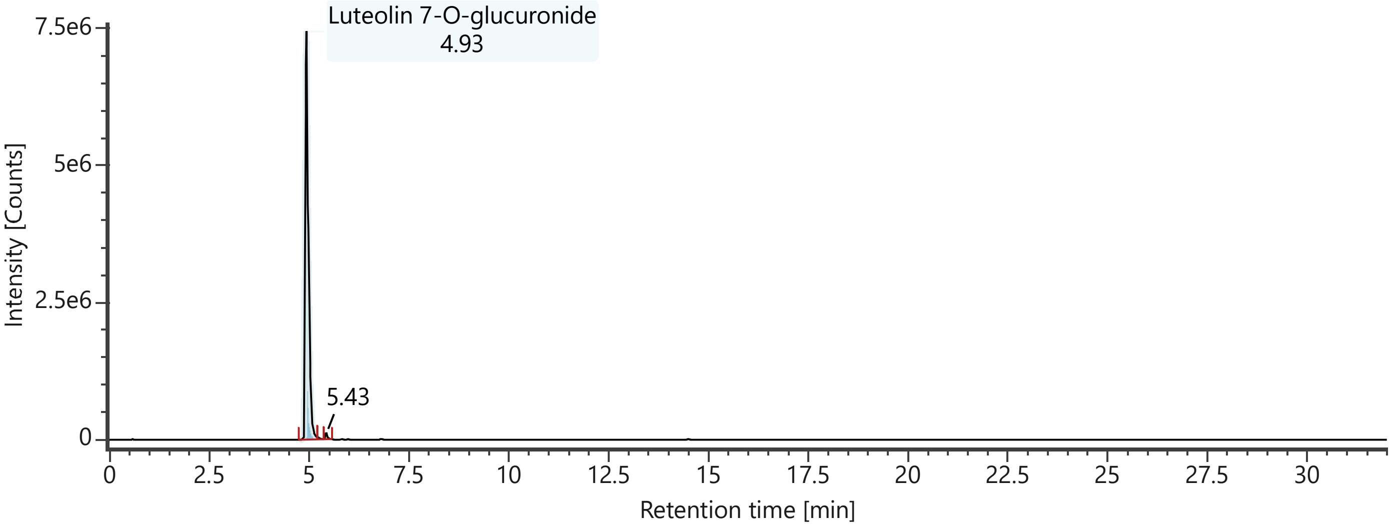

Item name: Sep257-ve
Item description: Mervat253
Channel name: Low energy : Time 4.9340 +/- 0.0666 minutes

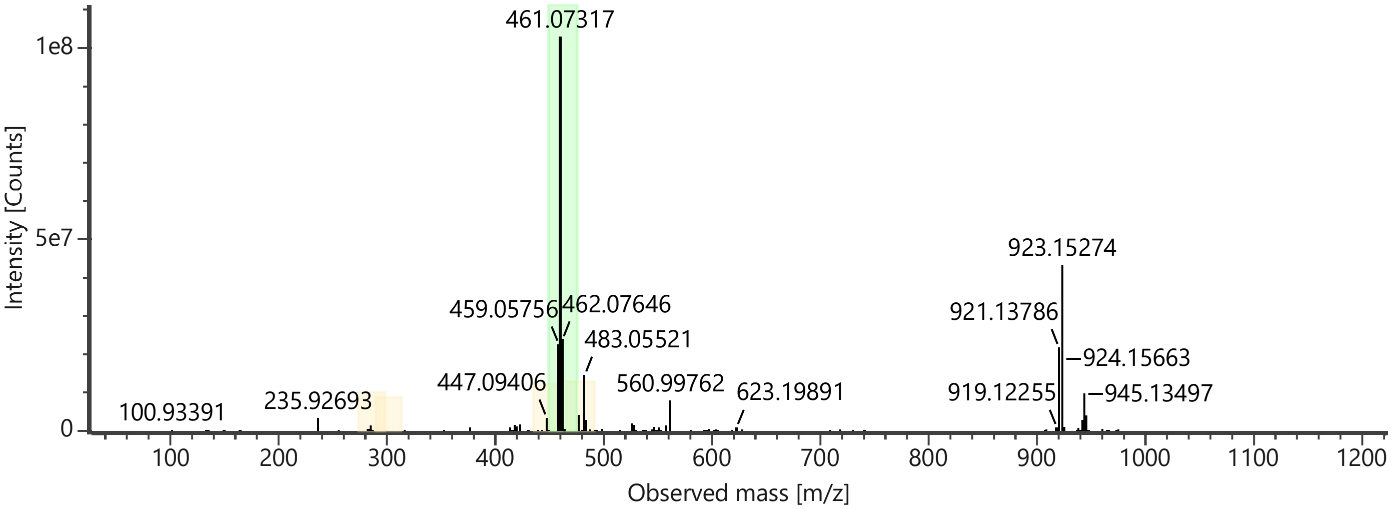

Item name: Sep257-ve
Channel name: High energy : Time 4.9340 +/- 0.0666 minutes
Item description: Mervat253

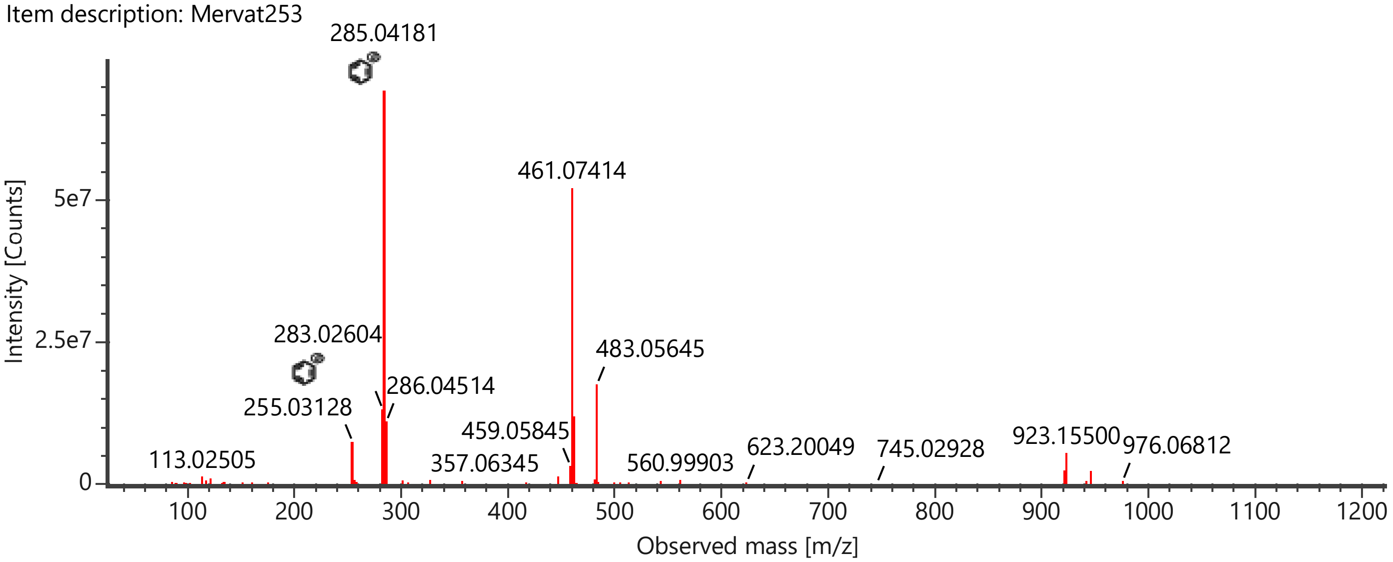

Component name: Quercitrone

Item name: Sep257-ve
Channel name: Querciturone [-H] : (52.5 PPM) 477.0679

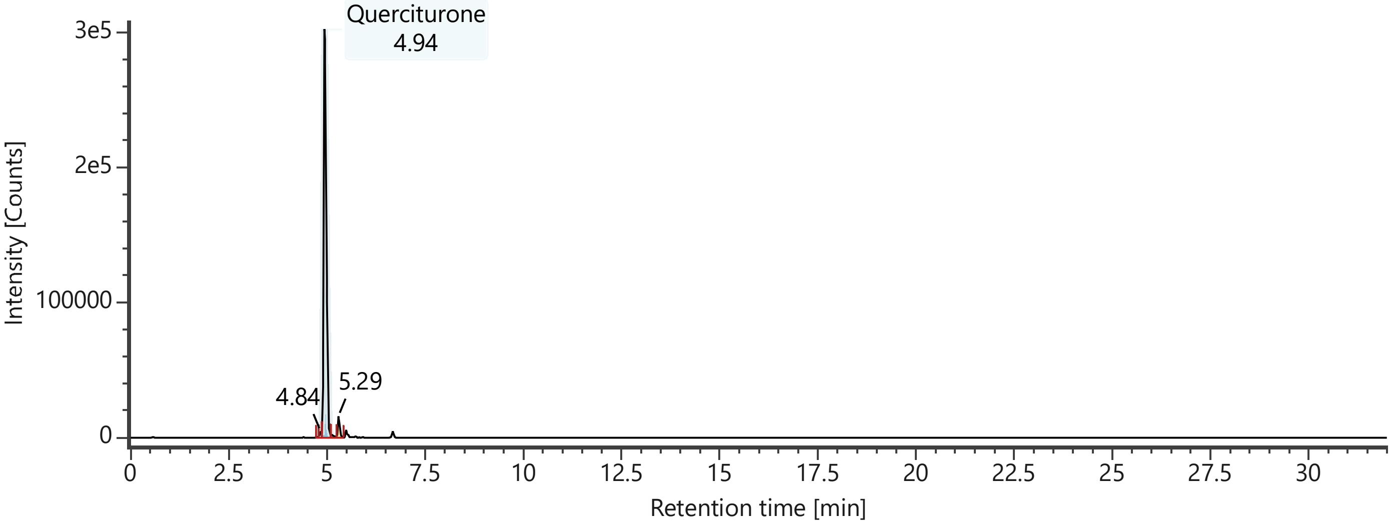

Item name: Sep257-ve
Item description: Mervat253
Channel name: Low energy : Time 4.9396 +/- 0.0222 minutes

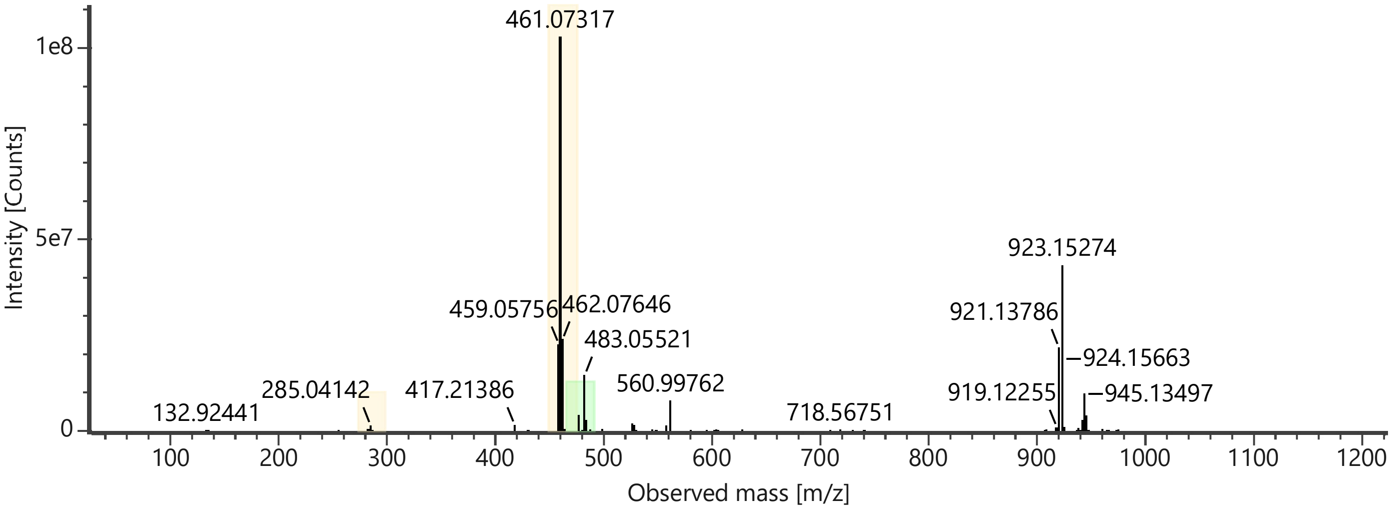

Item name: Sep257-ve
Item description: Mervat253

Channel name: High energy : Time 4.9396 +/- 0.0222 minutes

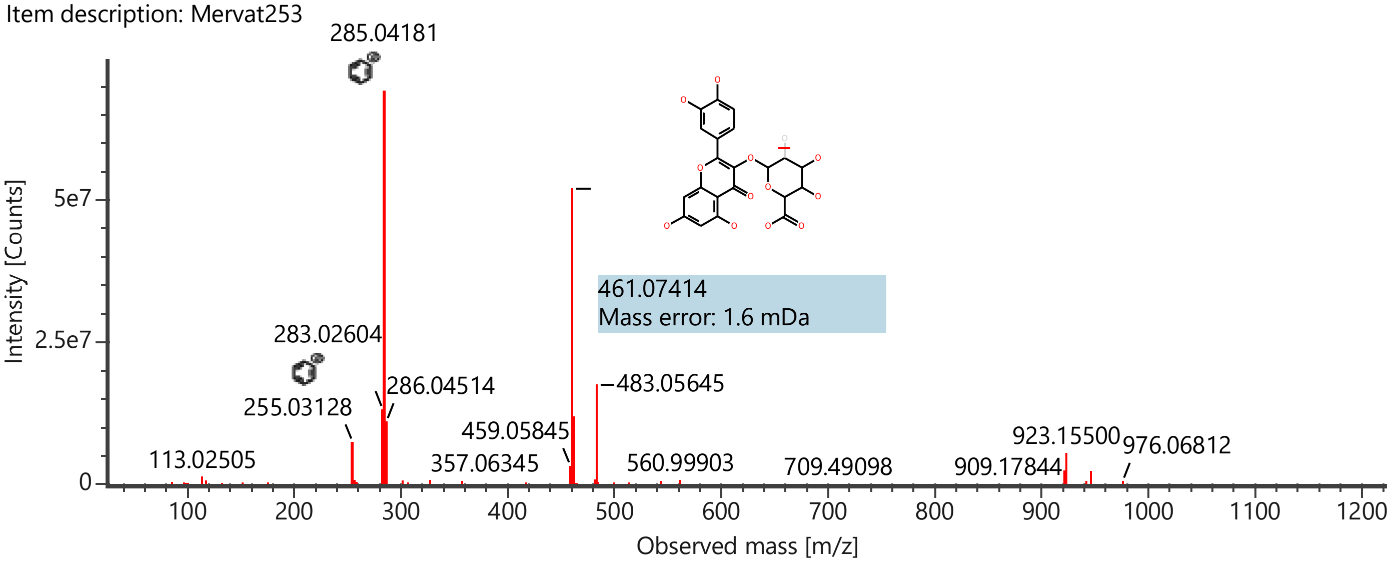

Component name: Kaempferol 3-glucoside-7-rhamnoside

Item name: Sep257-ve
Channel name: Kaempferol 3-glucoside-7-rhamnoside [-H] : (52.5 PPM) 593.1520

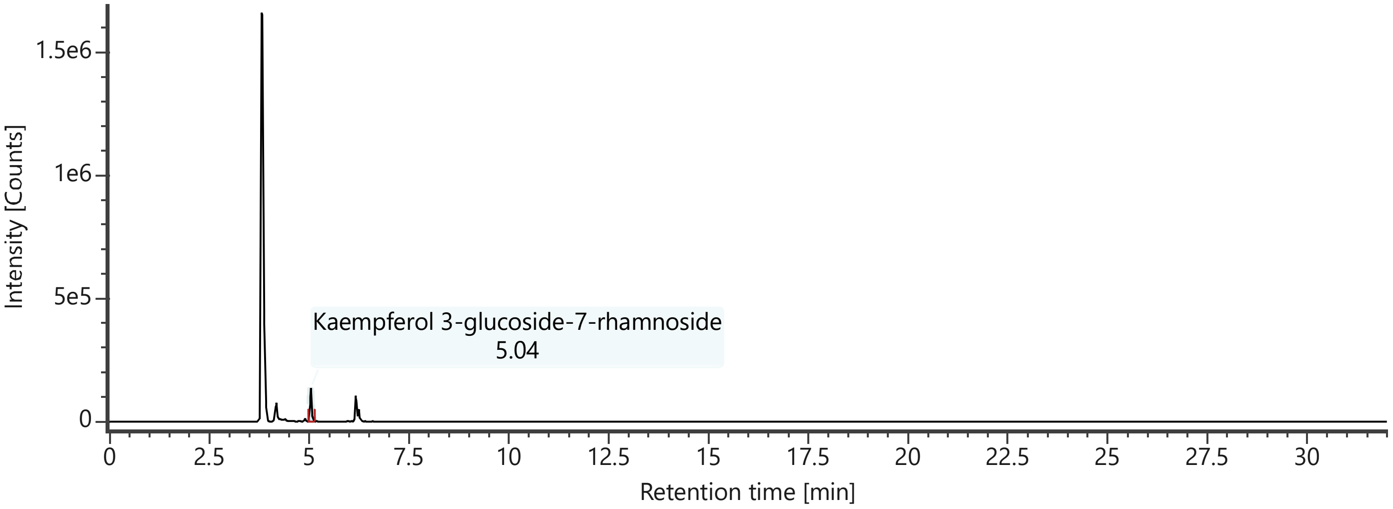

Item name: Sep257-ve
Item description: Mervat253
Channel name: Low energy : Time 5.0391 +/- 0.0222 minutes

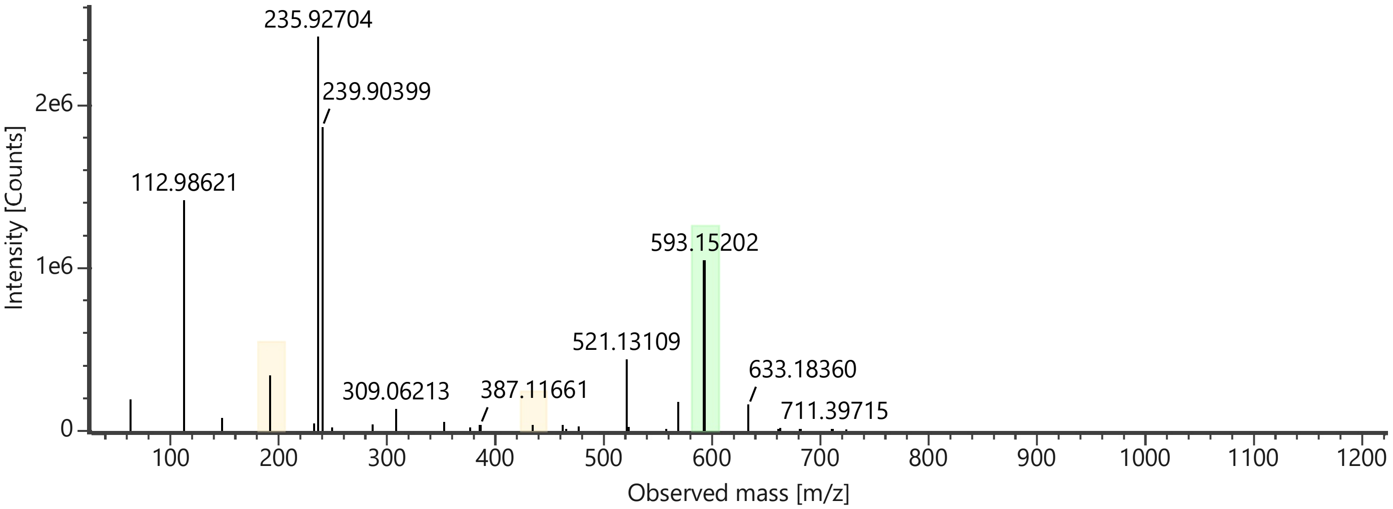

Item name: Sep257-ve
Item description: Mervat253

Channel name: High energy : Time 5.0391 +/- 0.0222 minutes

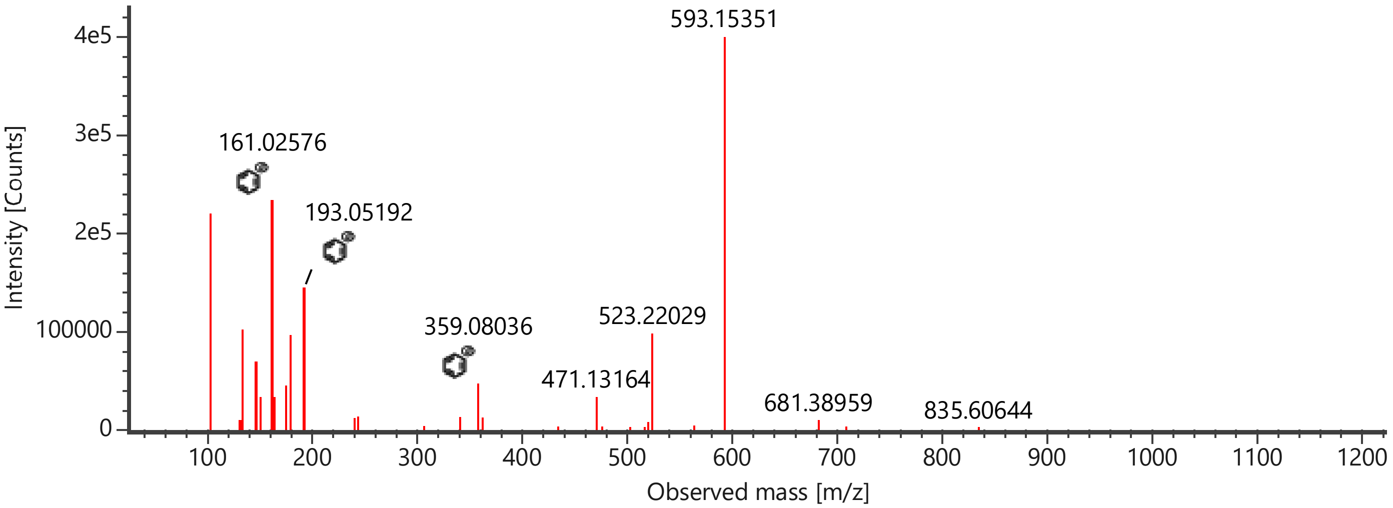

Component name: Kaempferol-7-O-β-D-glucoside

Item name: Sep257-ve
Channel name: Kaempferol-7-O-β-D-glucoside [-H] : (52.5 PPM) 447.0944

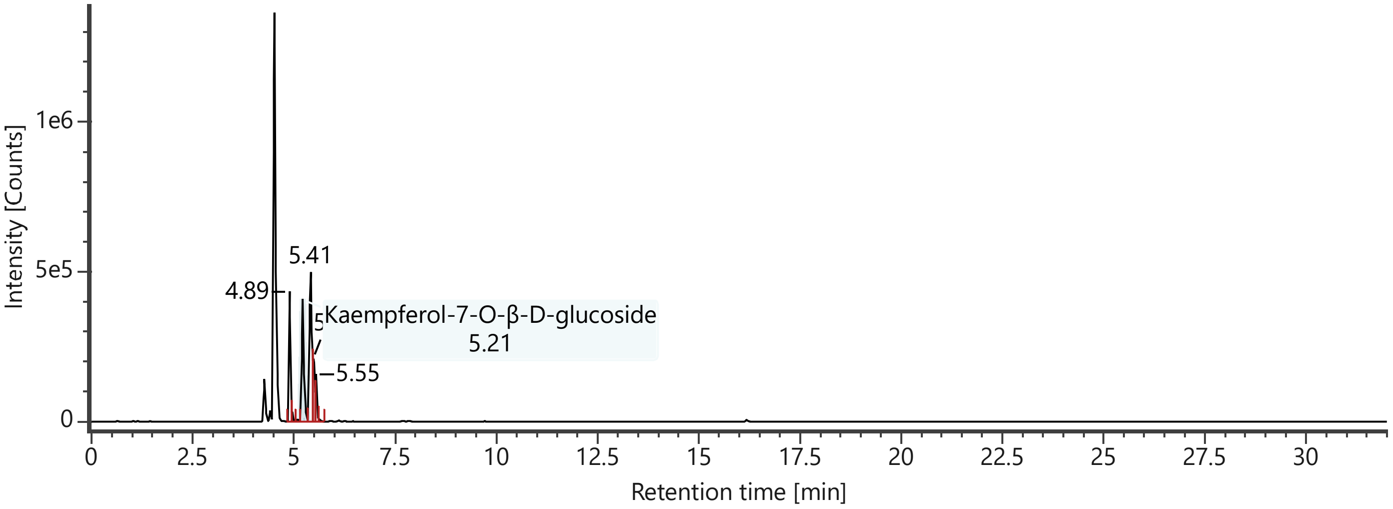

Item name: Sep257-ve
Item description: Mervat253
Channel name: Low energy : Time 5.2160 +/- 0.0222 minutes

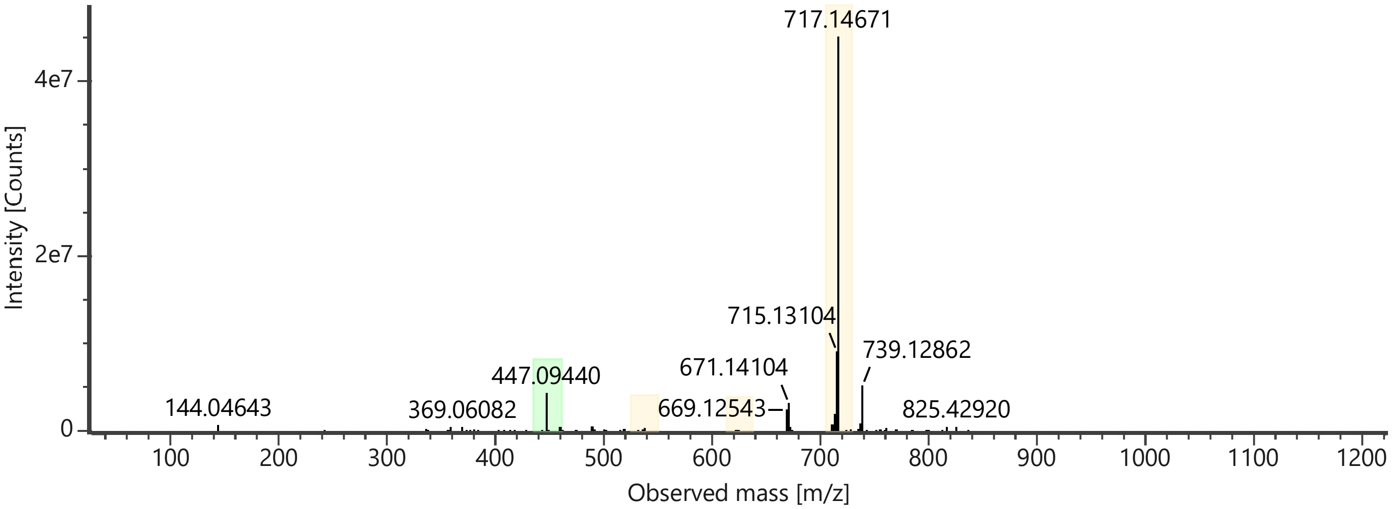

Item name: Sep257-ve
Item description: Mervat253
Channel name: High energy : Time 5.2160 +/- 0.0222 minutes

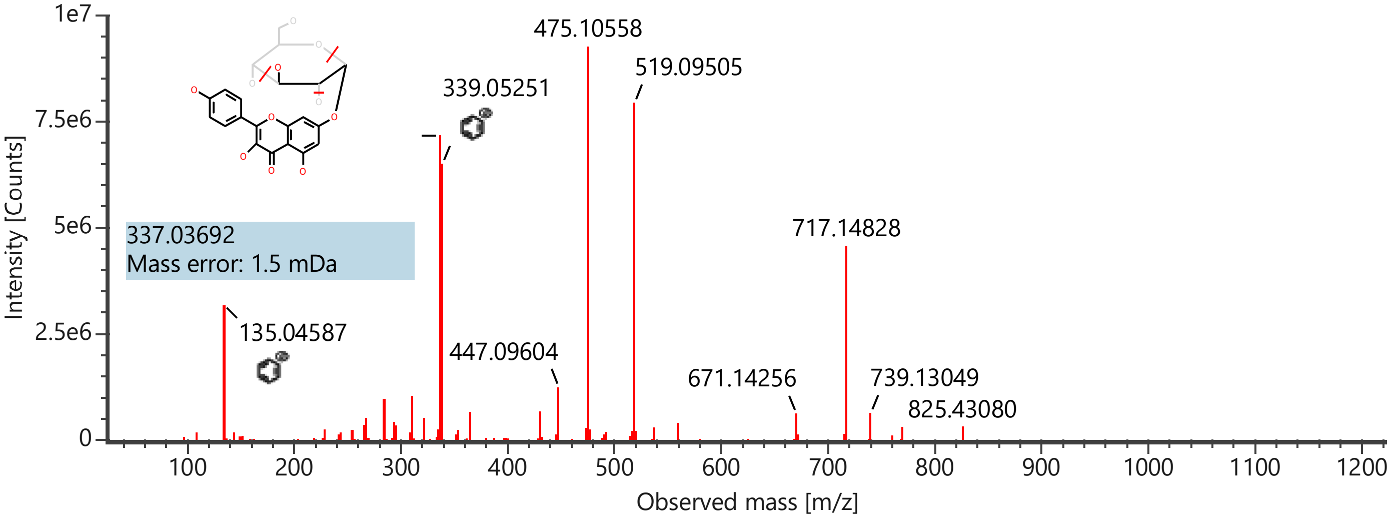

Created by: Ayad, Younan

Created on: Sep 18, 2025

Item name: Lamiaceae family -ve mode

Created time: 13:05:43 Egypt Standard Time

Component name: (2S)-2-[(1S,2R)-3-[(1S)-1-Carboxy-2-(3,4-dihydroxyphenyl)ethoxy]carbonyl-2-(3,4-dihydroxyphenyl)-7,8-dihydroxy-1,2-dihydronaphthalene-1-carbonyl]oxy-3-(3,4-dihydroxyphenyl)propanoic acid

Item name: Sep257-ve
Channel name: (2S)-2-[(1S,2R)-3-[(1S)-1-Carboxy-2-(3,4-dihydroxyphenyl)ethoxy]carbonyl-2-(3,4-dihydroxyphenyl)-7,8-dihydroxy-1,2-dihydronaphthalene-1-carbonyl]oxy-3-(3,4-dihydroxyphenyl)propanoic acid [-H] : (52.5 PPM) 717.1467

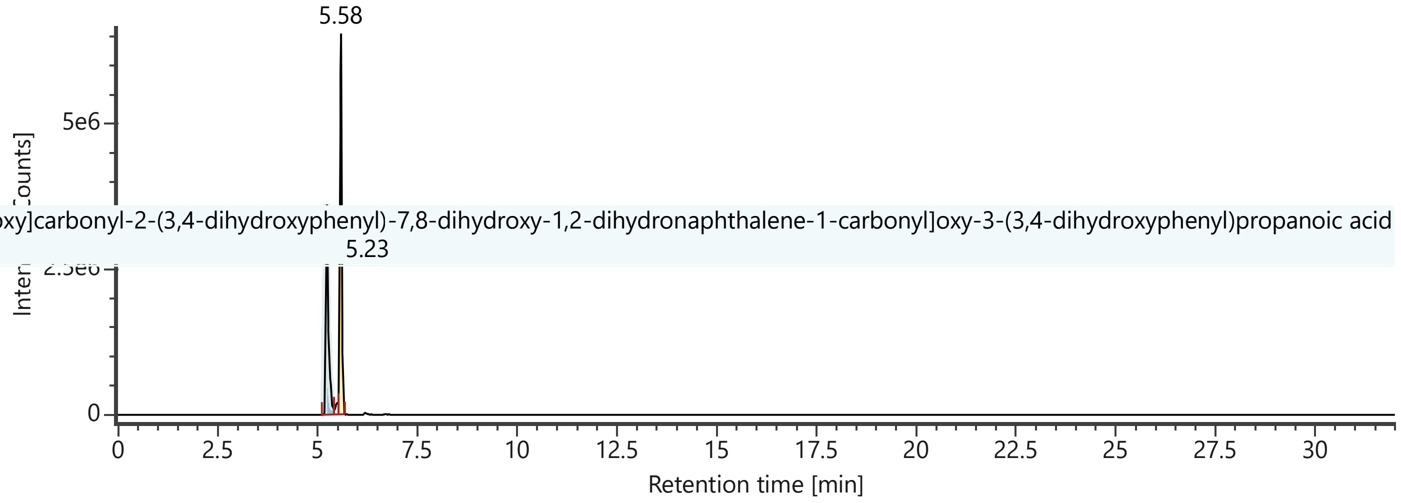

Item name: Sep257-ve
Item description: Mervat253

Channel name: Low energy : Time 5.2325 +/- 0.0222 minutes

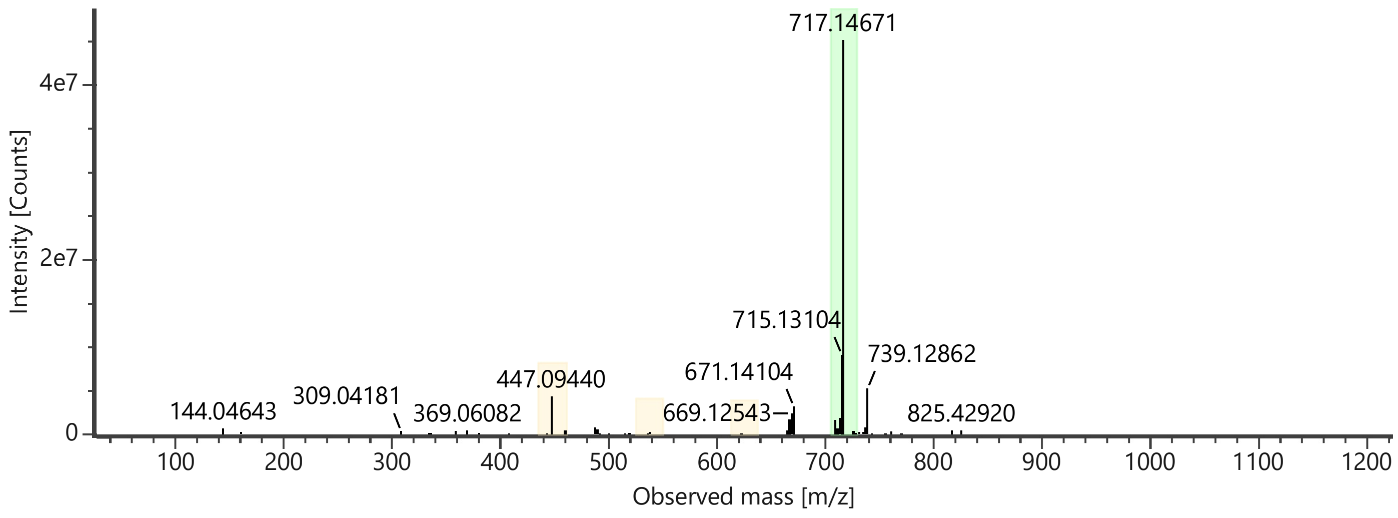

Item name: Sep257-ve
Item description: Mervat253

Channel name: High energy : Time 5.2325 +/- 0.0222 minutes

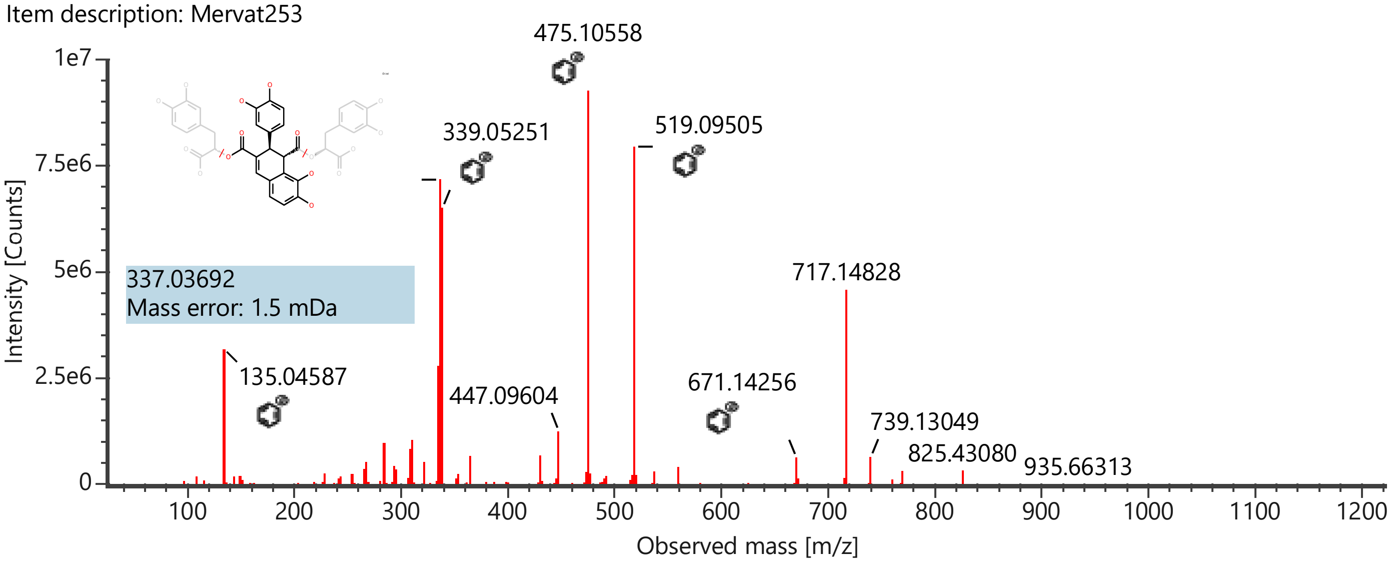

Component name: Apigenin 7-O-beta-D-glucopyranoside

Item name: Sep257-ve
Channel name: Apigenin 7-O-beta-D-glucopyranoside [-H] : (52.5 PPM) 431.0993

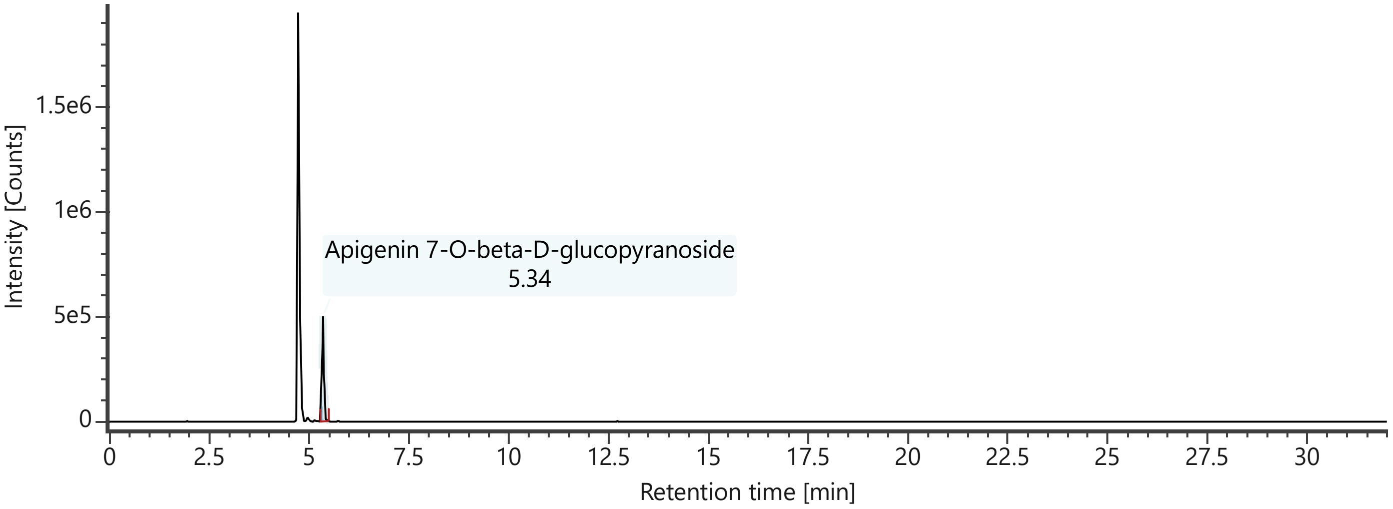

Item name: Sep257-ve
Item description: Mervat253
Channel name: Low energy : Time 5.3427 +/- 0.0222 minutes

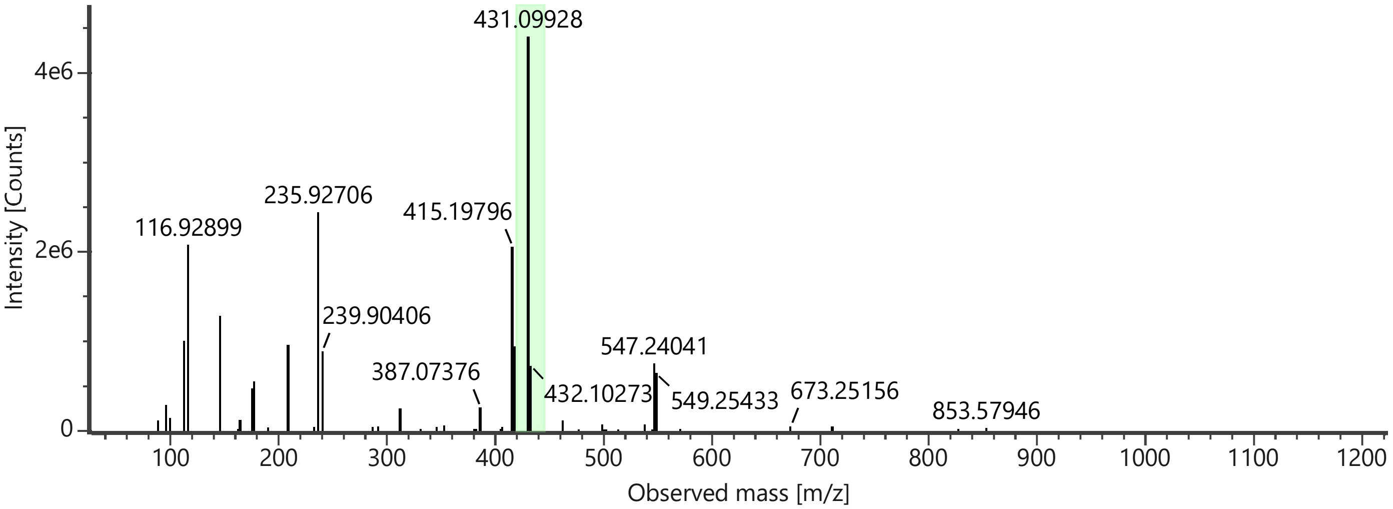

Item name: Sep257-ve
Item description: Mervat253

Channel name: High energy : Time 5.3427 +/- 0.0222 minutes

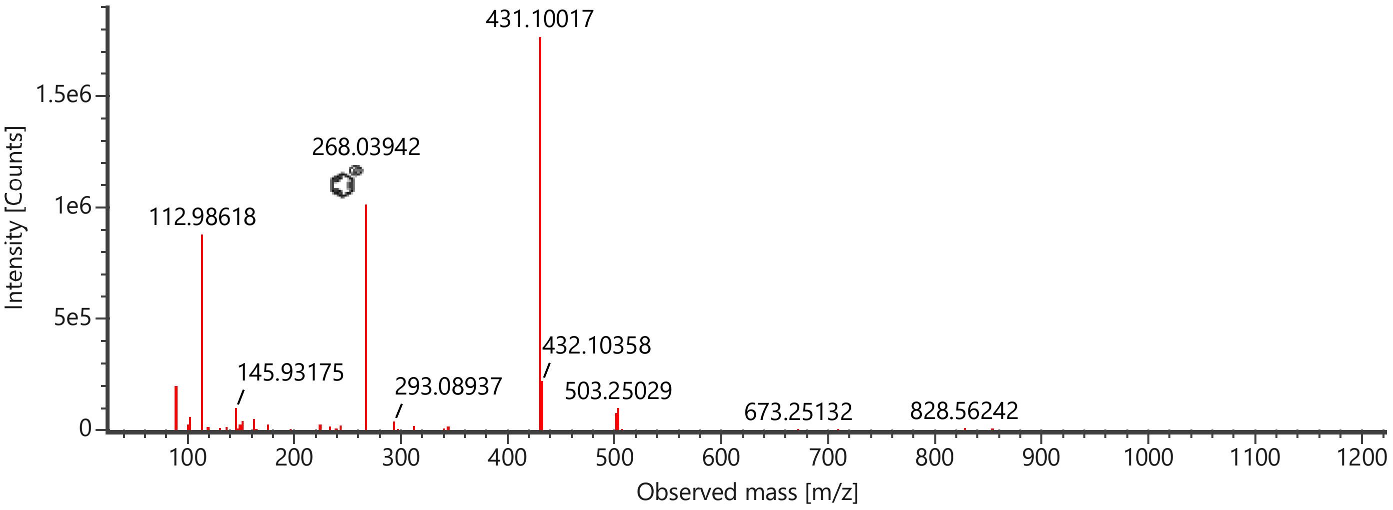

Component name: Apigenin-4'-O-glucuronide

Item name: Sep257-ve
Channel name: Apigenin-4'-O-glucuronide [-H] : (52.5 PPM) 445.0782

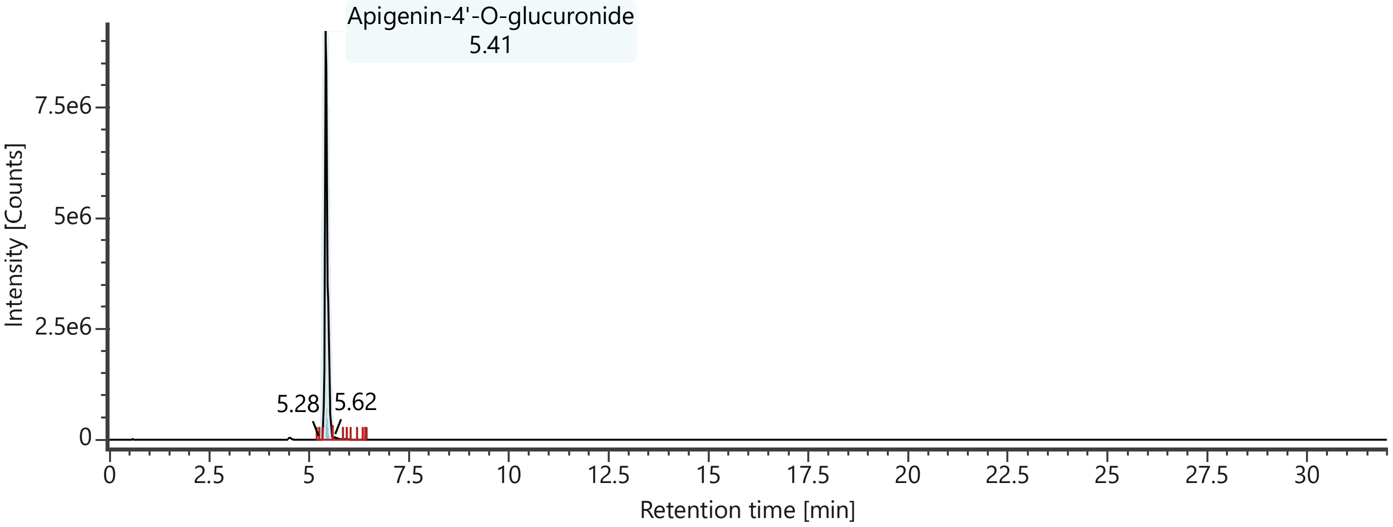

Item name: Sep257-ve
Item description: Mervat253
Channel name: Low energy : Time 5.4140 +/- 0.0666 minutes

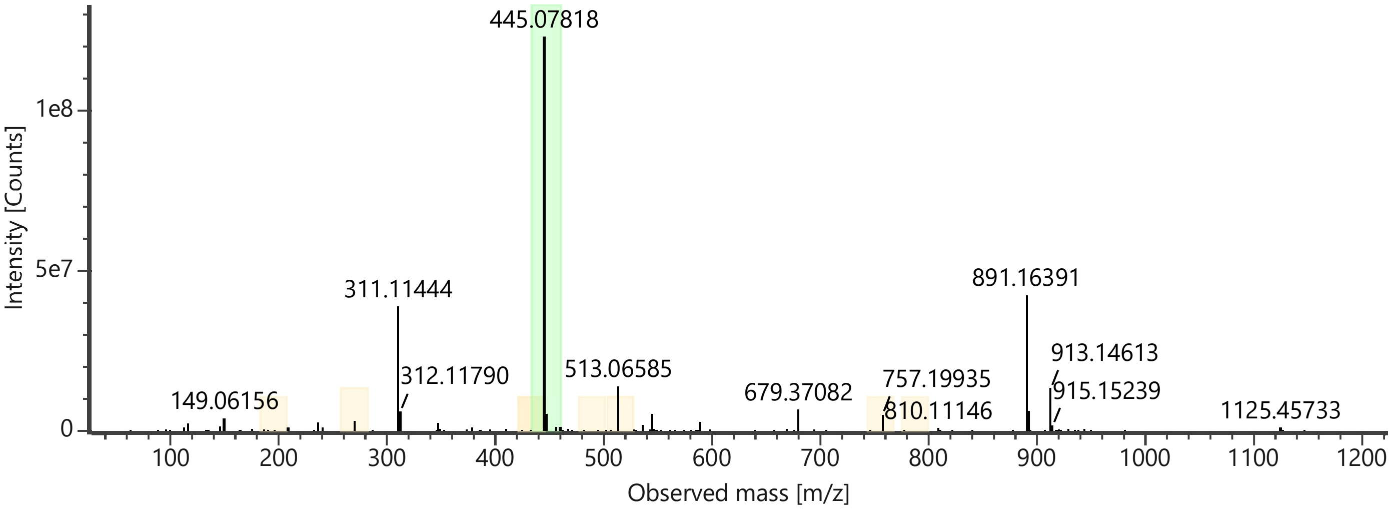

Created by: Ayad, Younan

Created on: Sep 18, 2025

Item name: Lamiaceae family -ve mode

Created time: 13:05:43 Egypt Standard Time

Item name: Sep257-ve

Item description: Mervat253

Channel name: High energy : Time 5.4140 +/- 0.0666 minutes

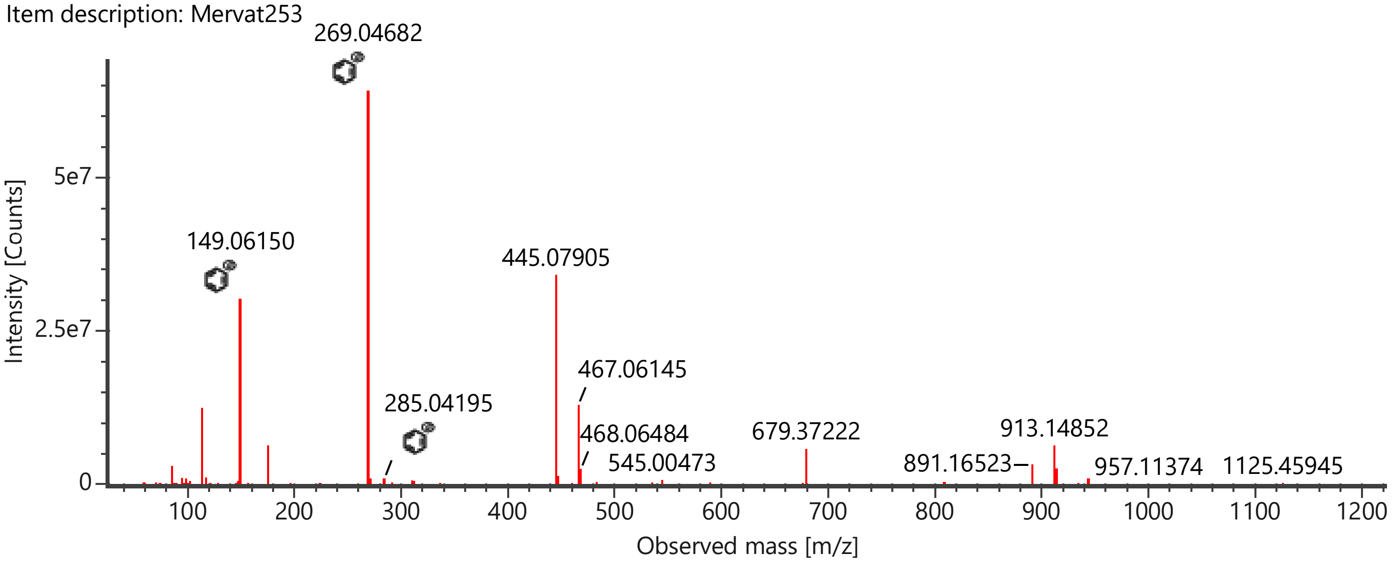

Component name: Apigenin

Item name: Sep257-ve
Channel name: Apigenin [-H] : (52.5 PPM) 269.0465

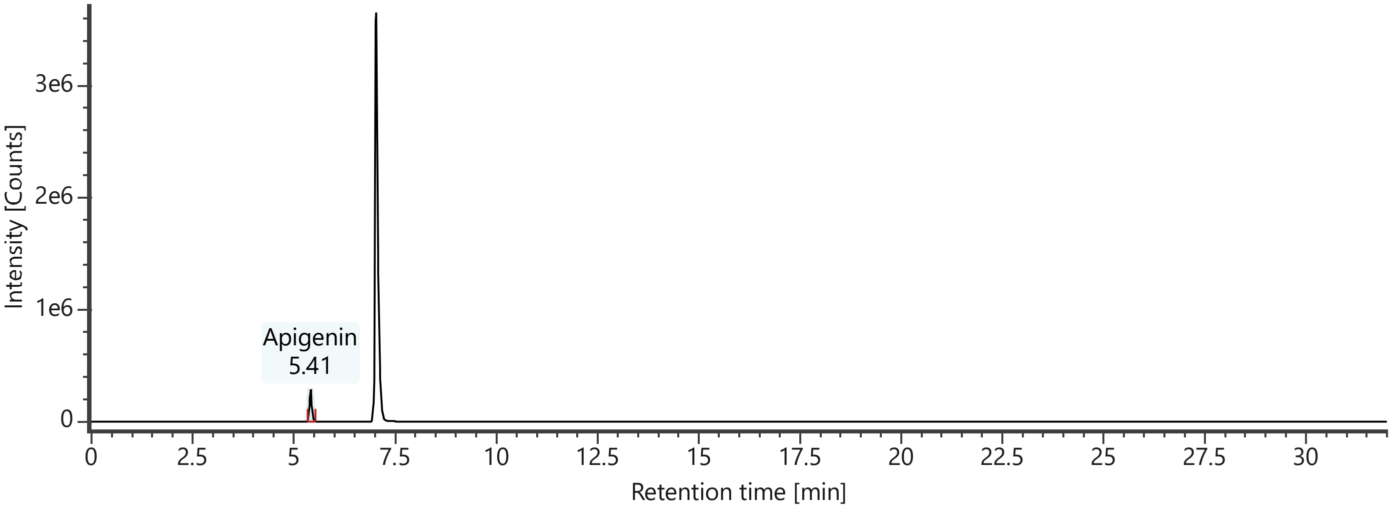

Item name: Sep257-ve
Item description: Mervat253
Channel name: Low energy : Time 5.4141 +/- 0.0222 minutes

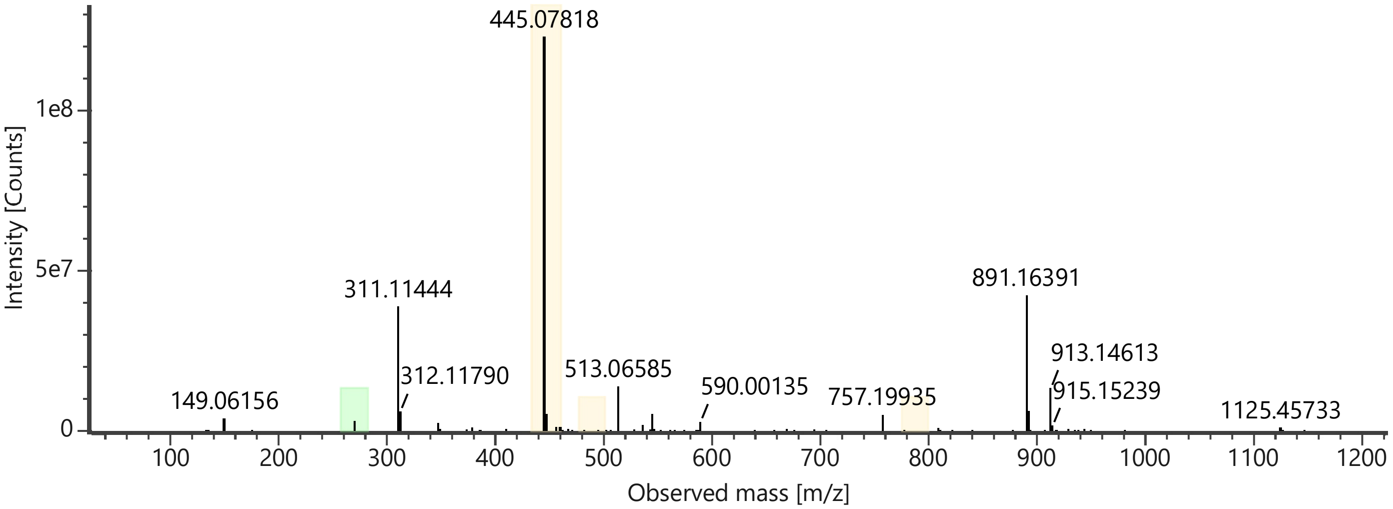

Item name: Sep257-ve
Item description: Mervat253

Channel name: High energy : Time 5.4141 +/- 0.0222 minutes

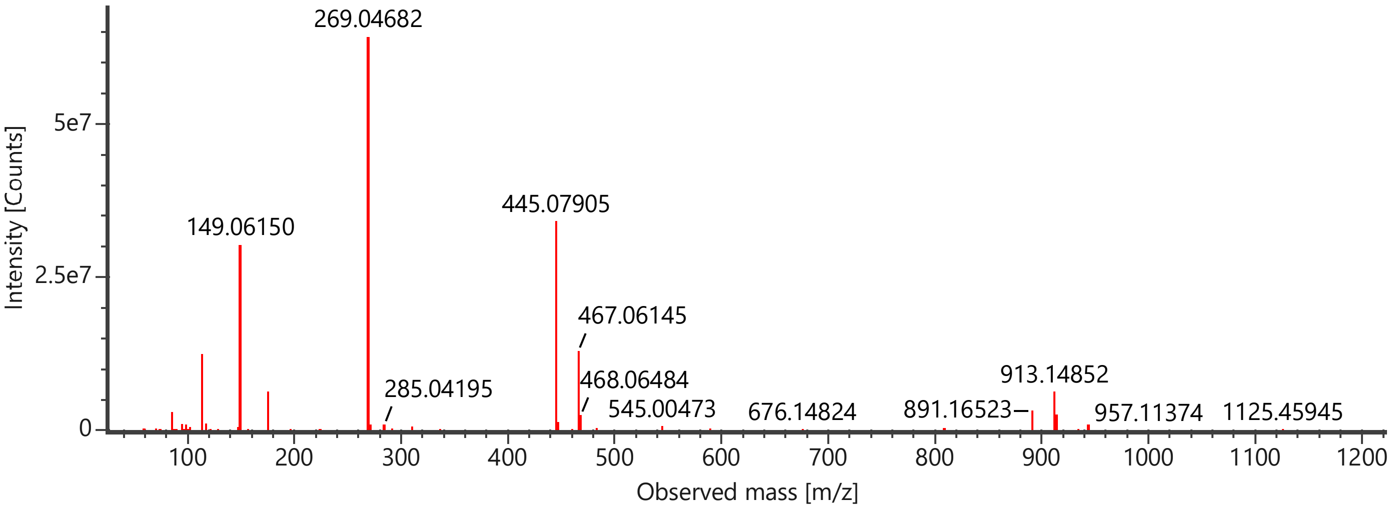

Component name: (2S)-2-[(1S,2R)-3-[(1S)-1-Carboxy-2-(3,4-dihydroxyphenyl)ethoxy]carbonyl-2-(3,4-dihydroxyphenyl)-7,8-dihydroxy-1,2-dihydronaphthalene-1-carbonyl]oxy-3-(3,4-dihydroxyphenyl)propanoic acid

Item name: Sep257-ve
Channel name: (2S)-2-[(1S,2R)-3-[(1S)-1-Carboxy-2-(3,4-dihydroxyphenyl)ethoxy]carbonyl-2-(3,4-dihydroxyphenyl)-7,8-dihydroxy-1,2-dihydronaphthalene-1-carbonyl]oxy-3-(3,4-dihydroxyphenyl)propanoic acid [-H] : (52.5 PPM) 717.1463

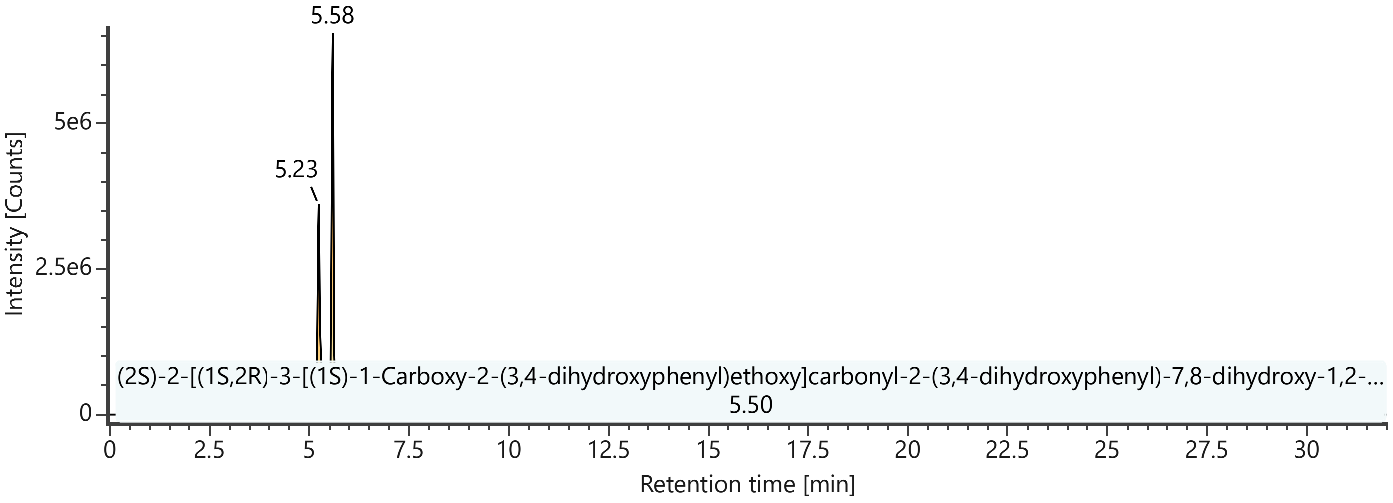

Item name: Sep257-ve
Item description: Mervat253
Channel name: Low energy : Time 5.4850 +/- 0.0222 minutes

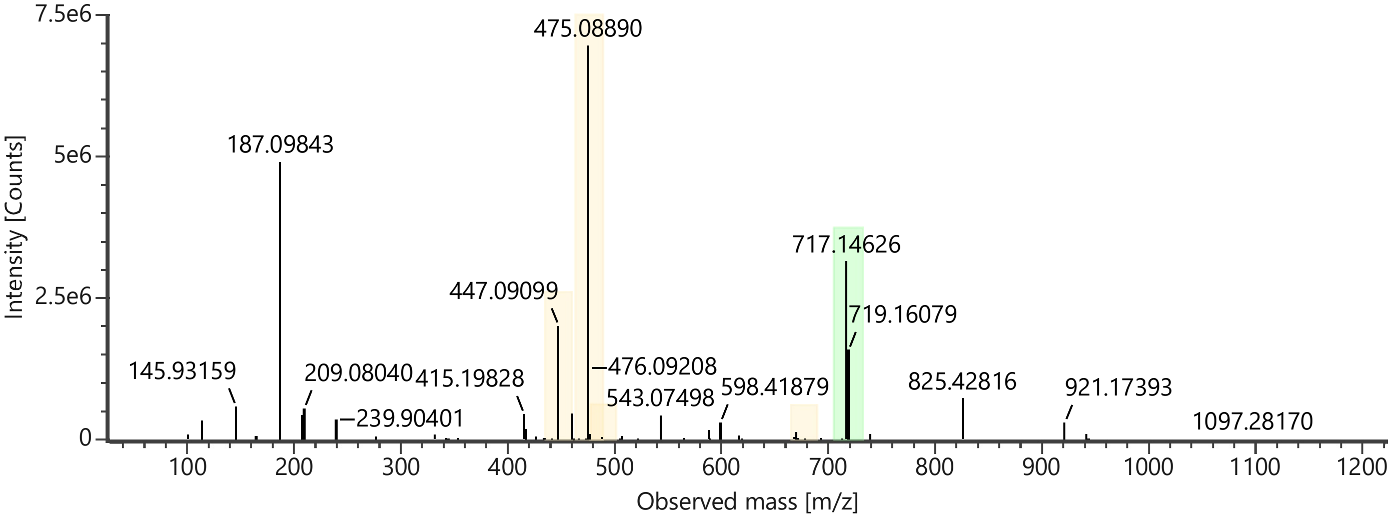

Item name: Sep257-ve
Item description: Mervat253

Channel name: High energy : Time 5.4850 +/- 0.0222 minutes

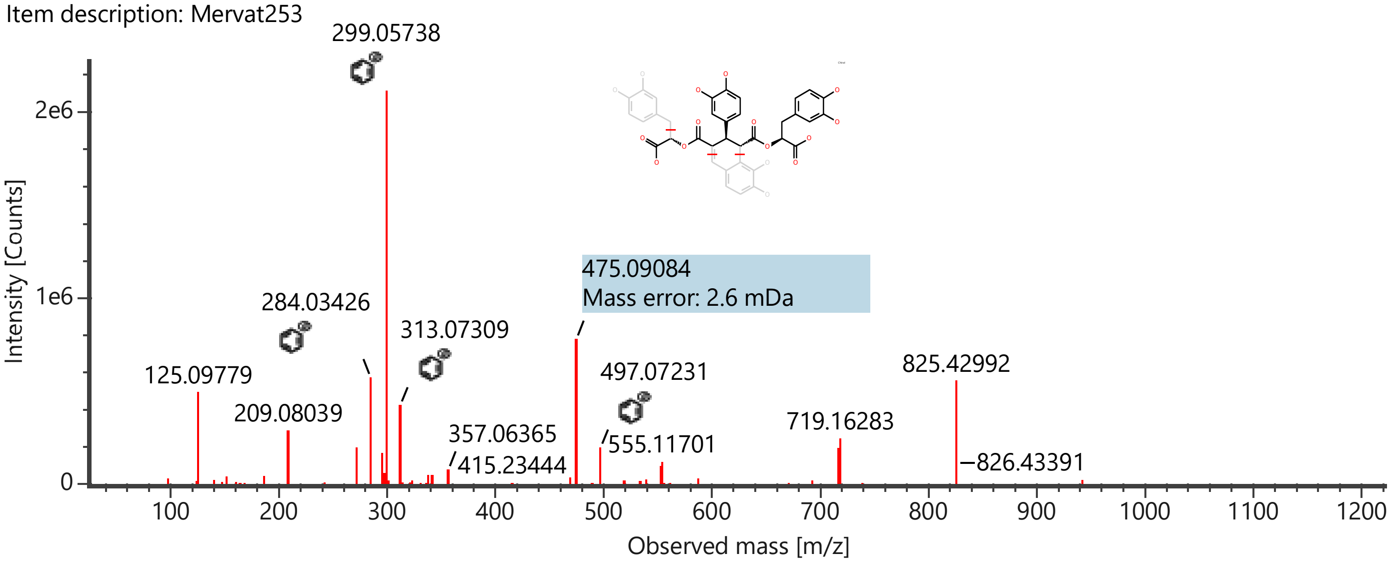

Component name: Luteolin-7-O-(6′′-methyl ester)-β-D-glucuronide

Item name: Sep257-ve
Channel name: Luteolin-7-O-(6′′-methyl ester)-β-D-glucuronide [-H] : (52.5 PPM) 475.0889

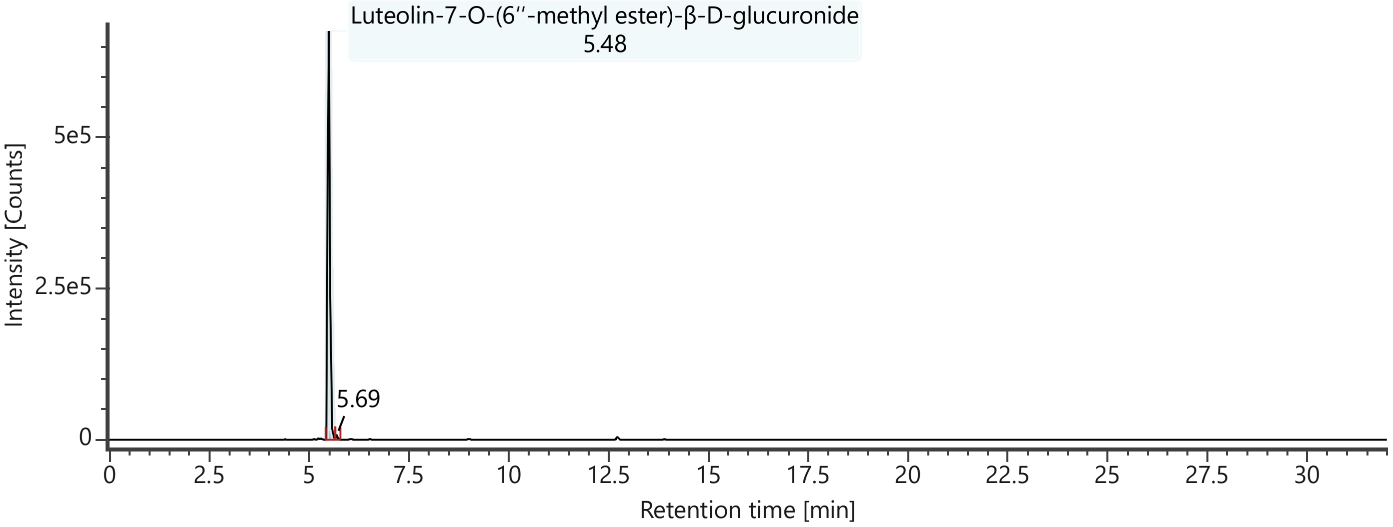

Item name: Sep257-ve
Item description: Mervat253
Channel name: Low energy : Time 5.4871 +/- 0.0222 minutes

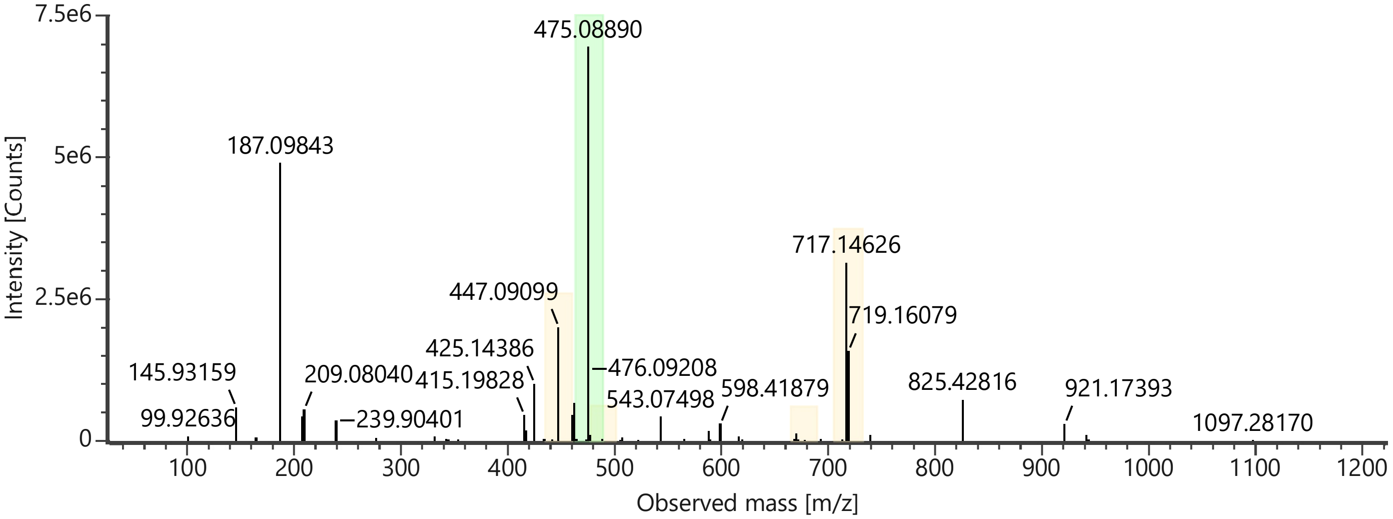

Item name: Sep257-ve
Item description: Mervat253

Channel name: High energy : Time 5.4871 +/- 0.0222 minutes

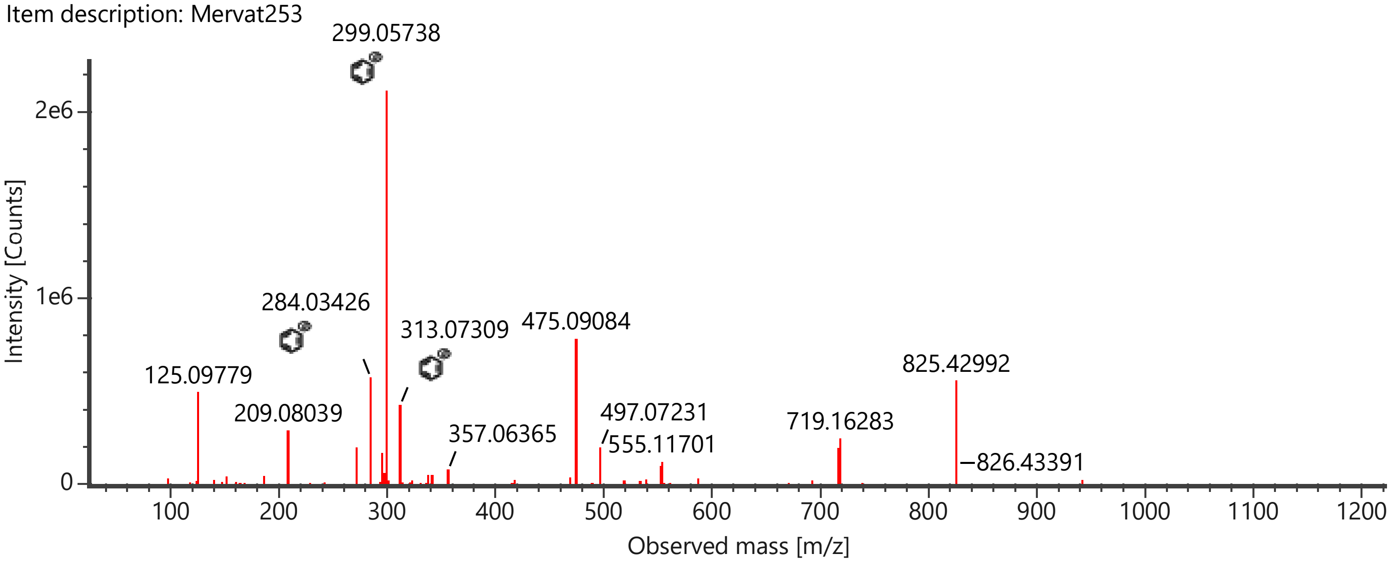

Component name: Kaempferol-7-O-β-D-glucoside

Item name: Sep257-ve
Channel name: Kaempferol-7-O-β-D-glucoside [-H] : (52.5 PPM) 447.0936

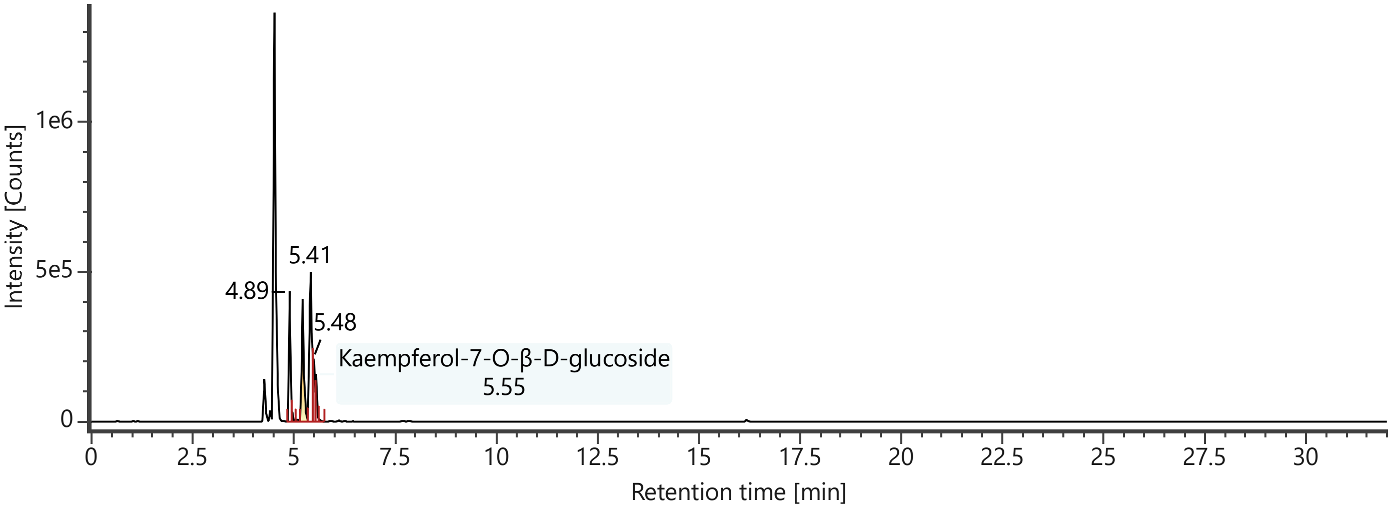

Item name: Sep257-ve
Item description: Mervat253
Channel name: Low energy : Time 5.5534 +/- 0.0222 minutes

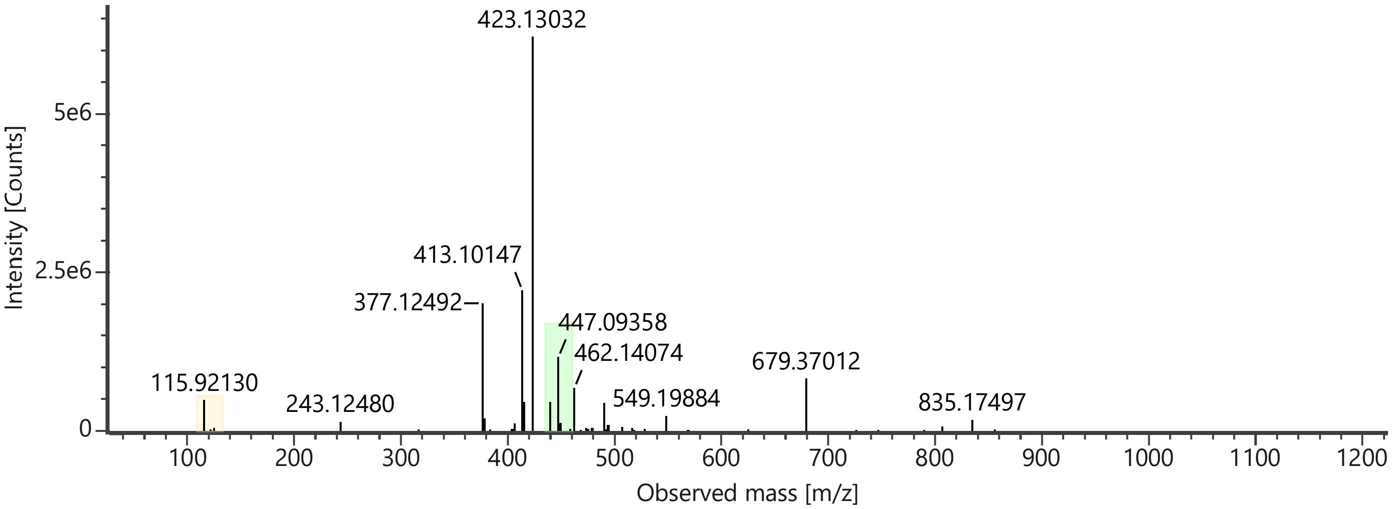

Item name: Sep257-ve
Item description: Mervat253

Channel name: High energy : Time 5.5534 +/- 0.0222 minutes

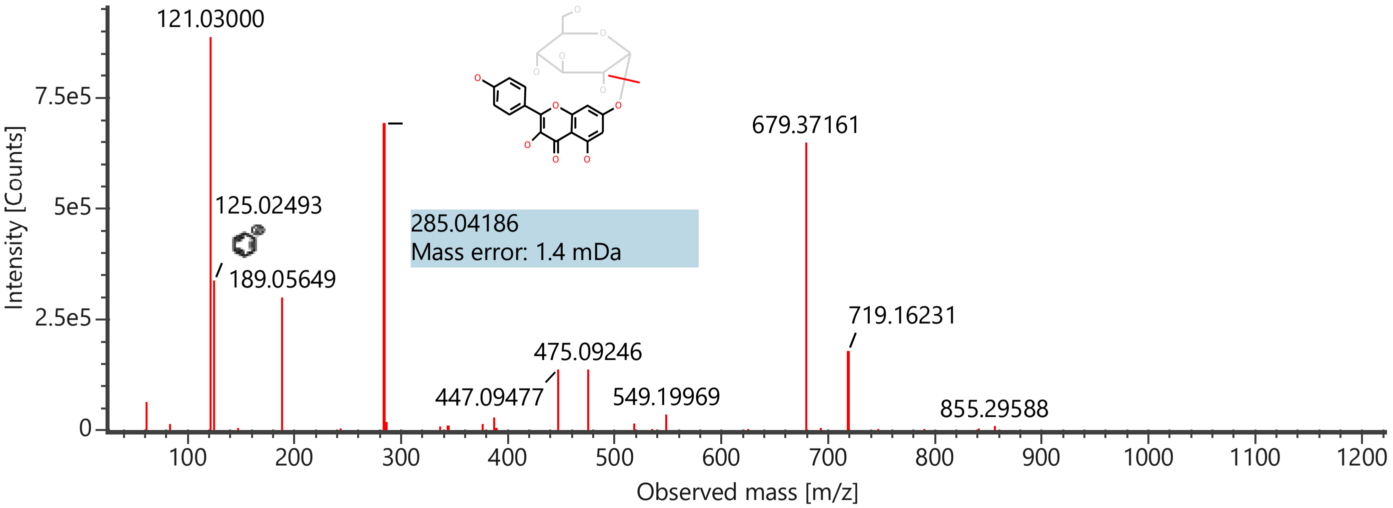

Component name: Rosmarinic acid

Item name: Sep257-ve
Channel name: Rosmarinic acid [-H] : (52.5 PPM) 359.0779

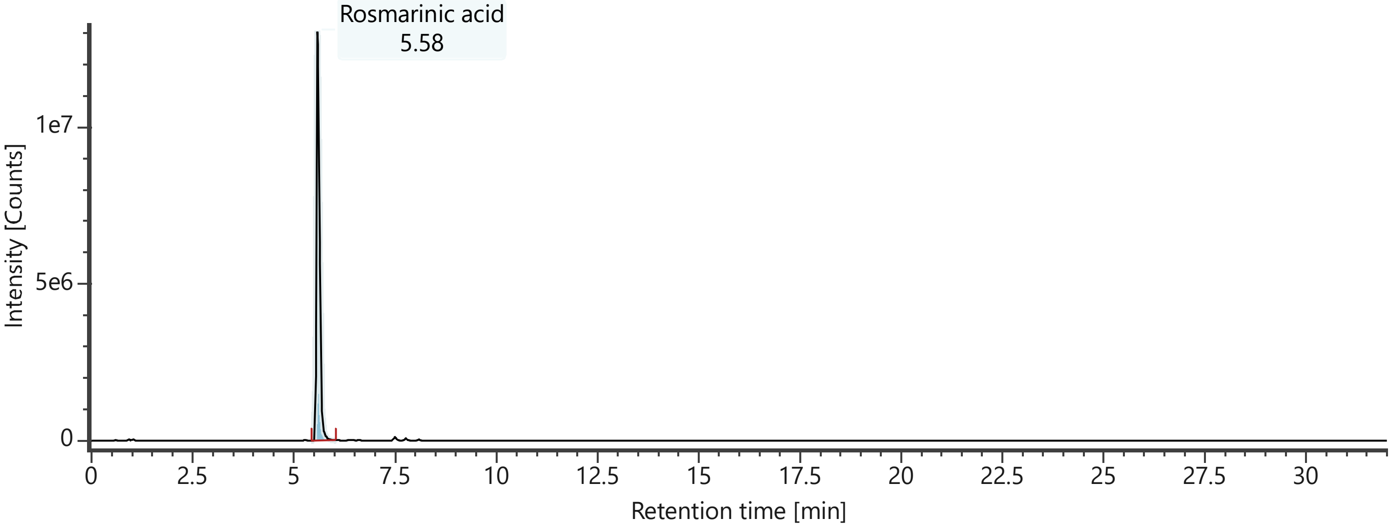

Item name: Sep257-ve
Item description: Mervat253
Channel name: Low energy : Time 5.5850 +/- 0.0666 minutes

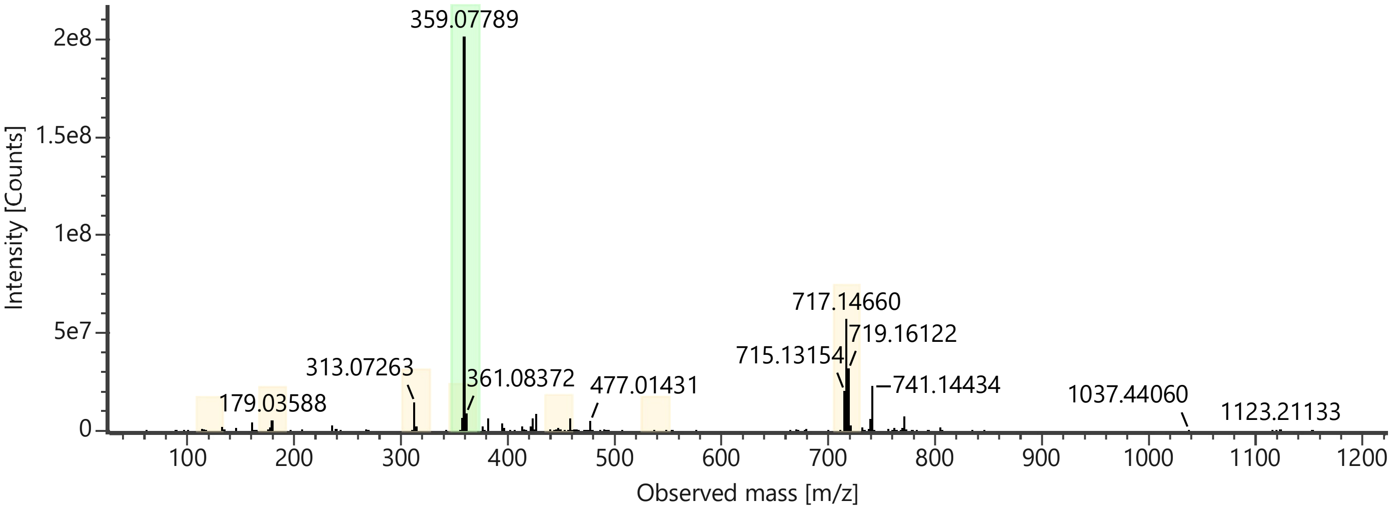

Item name: Sep257-ve
Item description: Mervat253

Channel name: High energy : Time 5.5850 +/- 0.0666 minutes

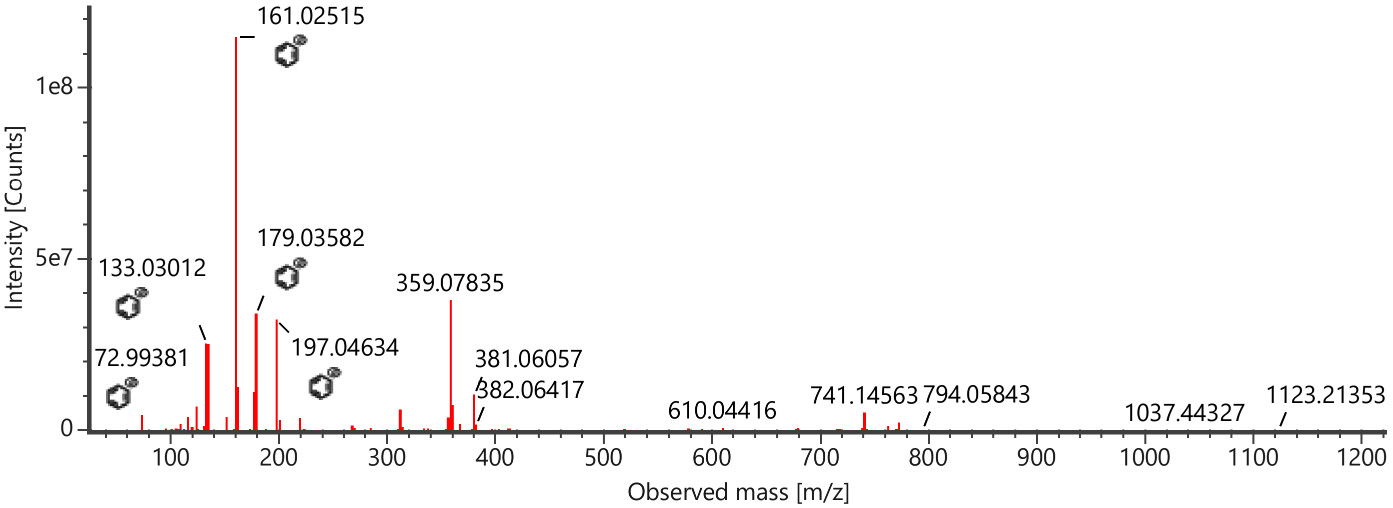

Component name: (2S)-2-[(1S,2R)-3-[(1S)-1-Carboxy-2-(3,4-dihydroxyphenyl)ethoxy]carbonyl-2-(3,4-dihydroxyphenyl)-7,8-dihydroxy-1,2-dihydronaphthalene-1-carbonyl]oxy-3-(3,4-dihydroxyphenyl)propanoic acid

Item name: Sep257-ve
Channel name: (2S)-2-[(1S,2R)-3-[(1S)-1-Carboxy-2-(3,4-dihydroxyphenyl)ethoxy]carbonyl-2-(3,4-dihydroxyphenyl)-7,8-dihydroxy-1,2-dihydronaphthalene-1-carbonyl]oxy-3-(3,4-dihydroxyphenyl)propanoic acid [-H] : (52.5 PPM) 717.1466

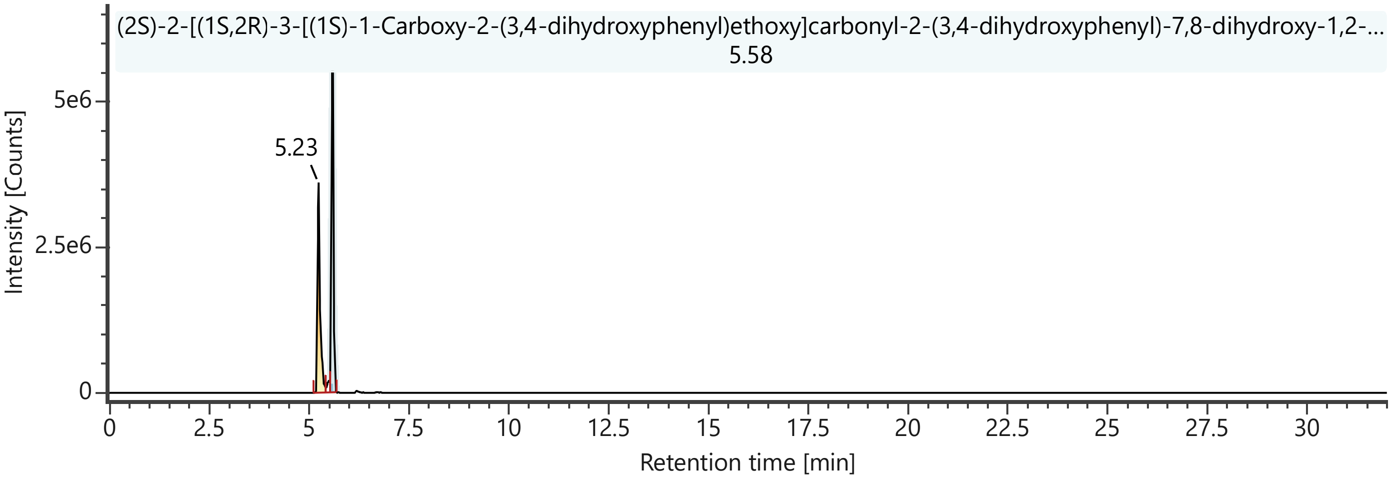

Item name: Sep257-ve
Item description: Mervat253
Channel name: Low energy : Time 5.5852 +/- 0.0222 minutes

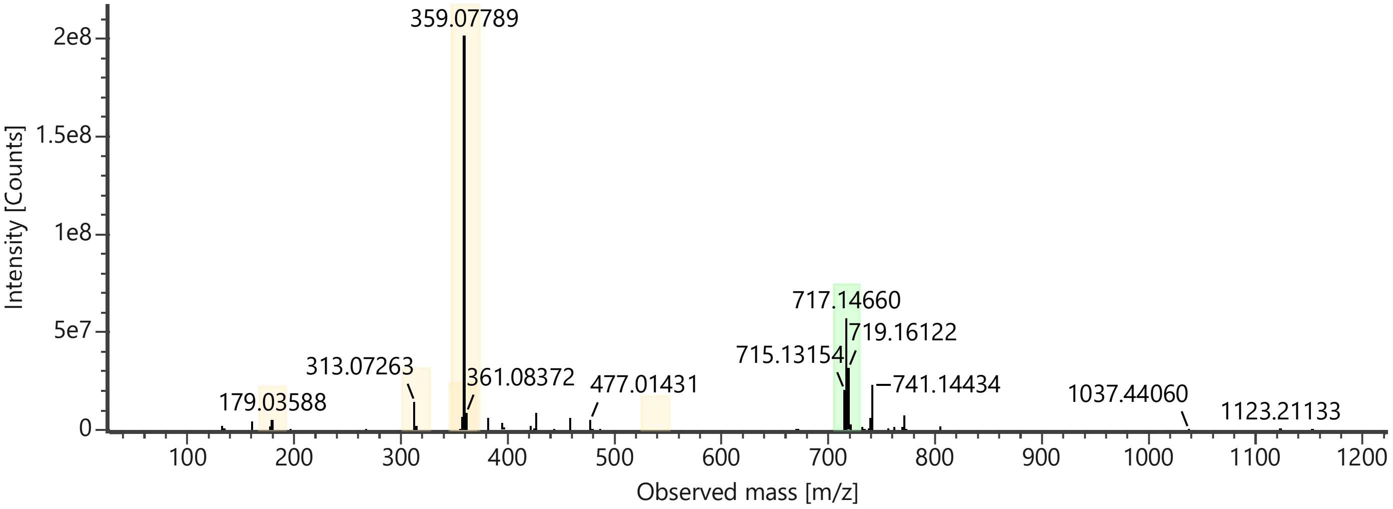

Item name: Sep257-ve
Item description: Mervat253

Channel name: High energy : Time 5.5852 +/- 0.0222 minutes

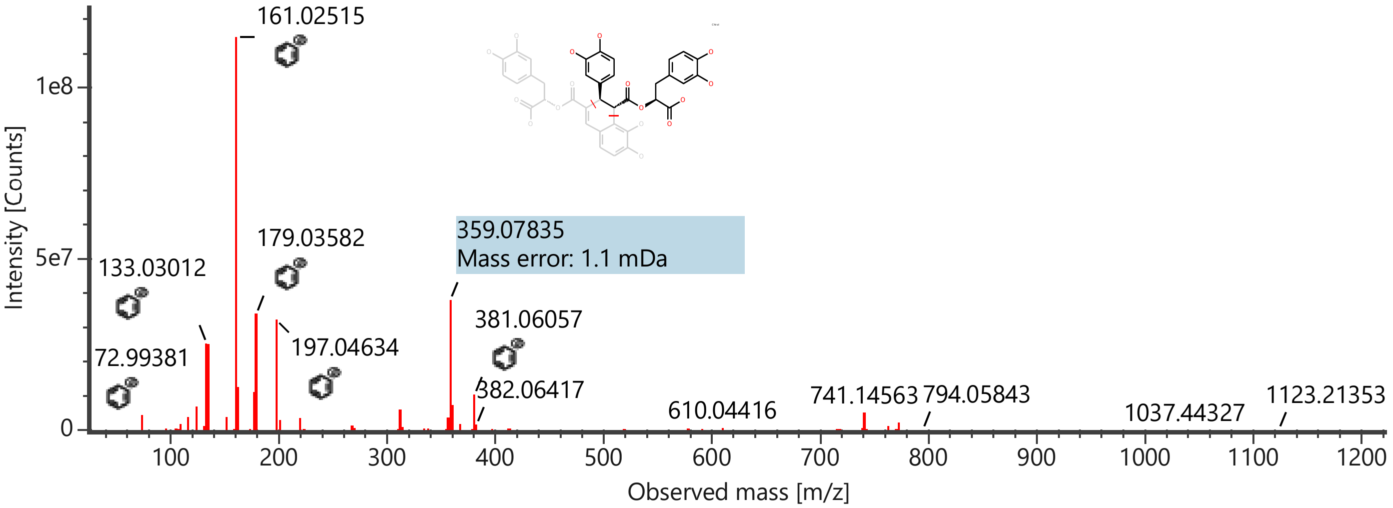

Component name: 7,8-Dihydroxy-2-(3,4-dihydroxyphenyl)-1,2-dihydronaphthalene-1,3-dicarboxylic acid

Item name: Sep257-ve
Channel name: 7,8-Dihydroxy-2-(3,4-dihydroxyphenyl)-1,2-dihydronaphthalene-1,3-dicarboxylic acid [-H] : (52.5 PPM) 357.0625

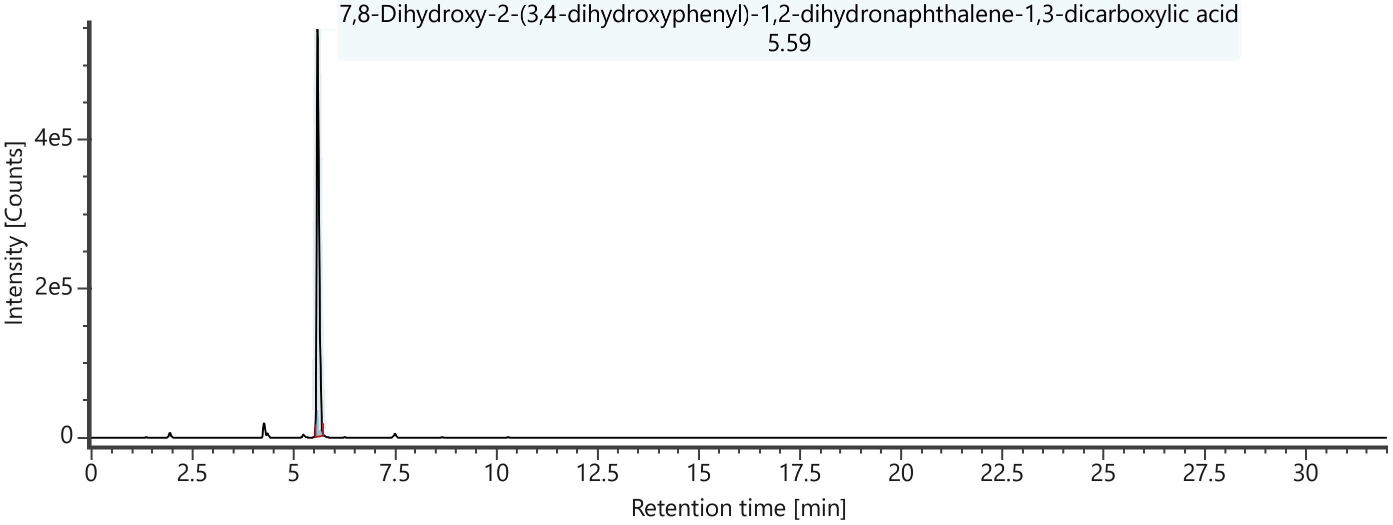

Item name: Sep257-ve
Item description: Mervat253
Channel name: Low energy : Time 5.5875 +/- 0.0222 minutes

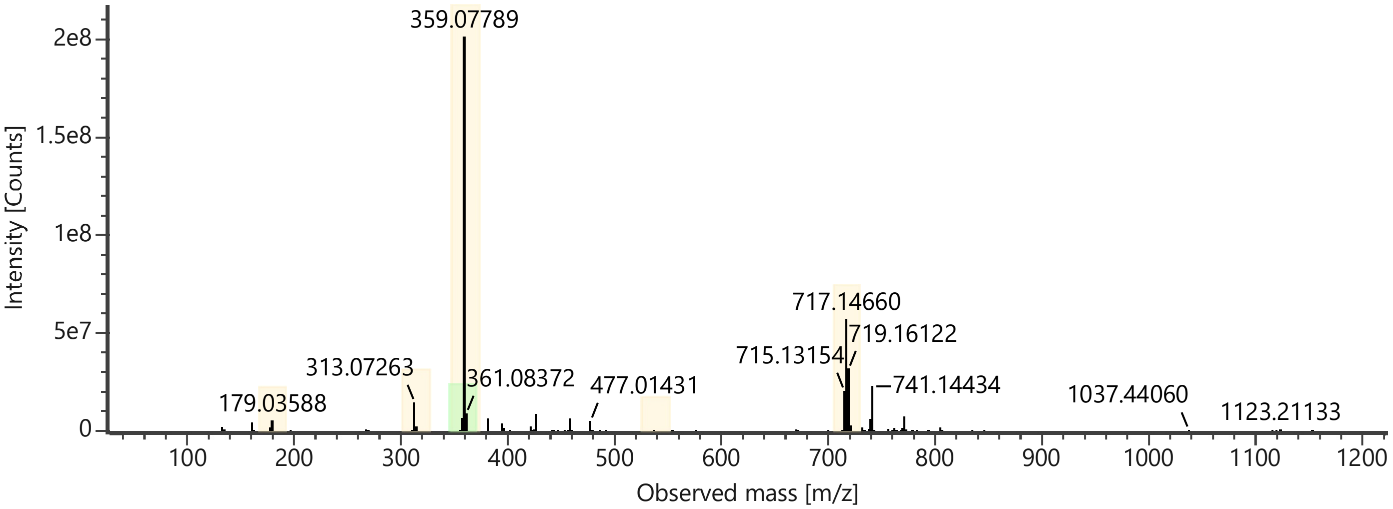

Item name: Sep257-ve
Item description: Mervat253

Channel name: High energy : Time 5.5875 +/- 0.0222 minutes

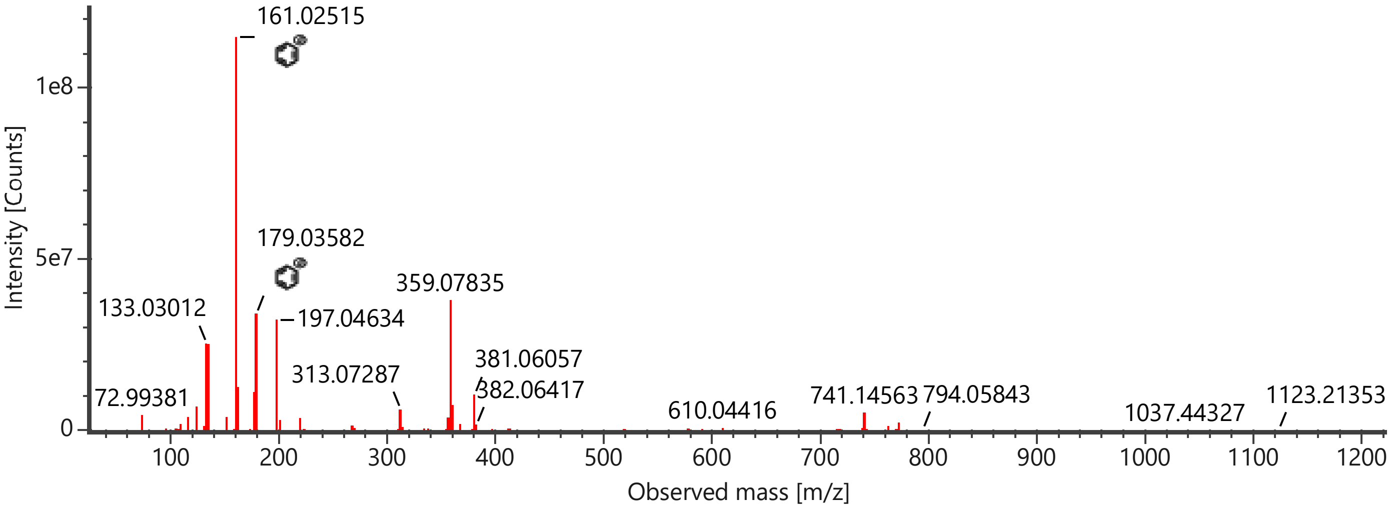

Component name: 3,5-Dihydroxy-4',7-dimethoxyflavone

Item name: Sep257-ve
Channel name: 3,5-Dihydroxy-4',7-dimethoxyflavone [-H] : (52.5 PPM) 313.0726

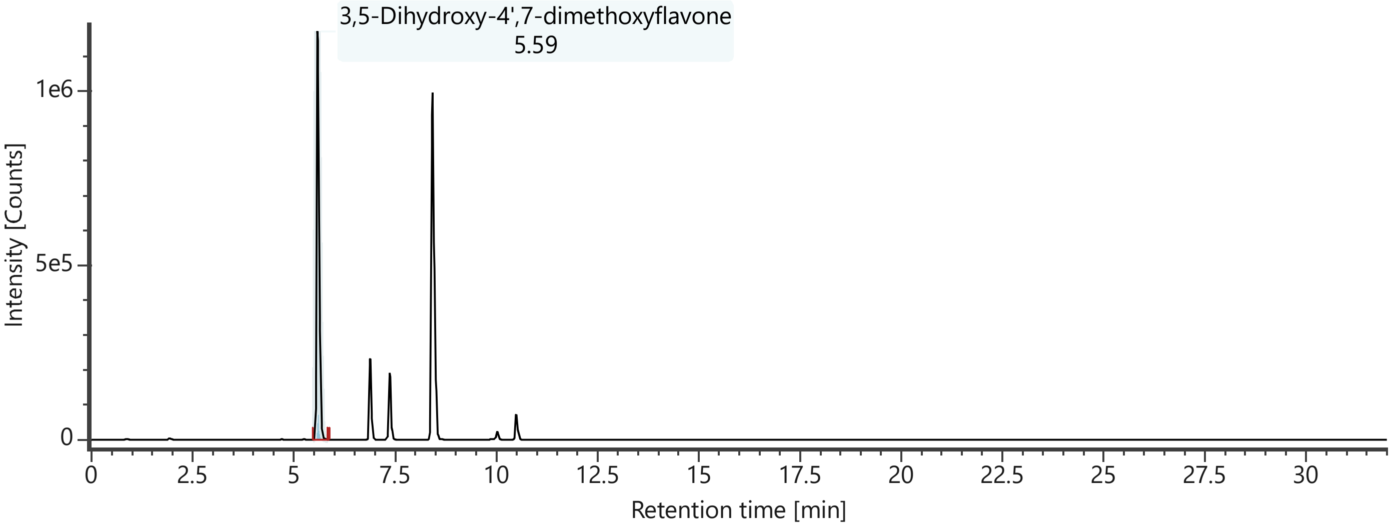

Item name: Sep257-ve
Item description: Mervat253
Channel name: Low energy : Time 5.5876 +/- 0.0222 minutes

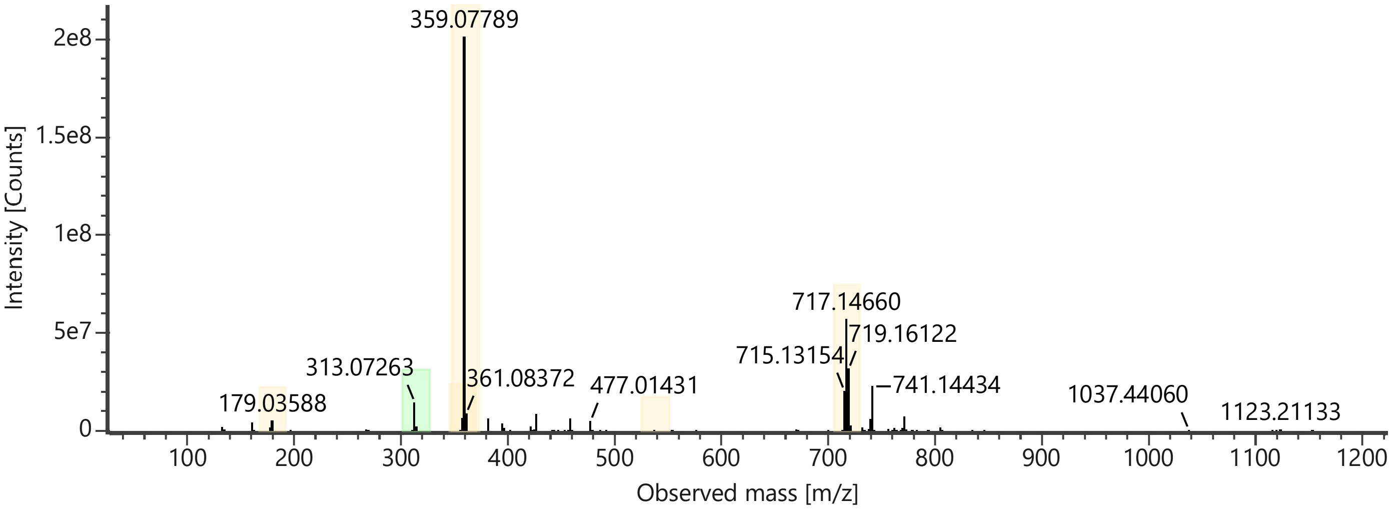

Item name: Sep257-ve
Item description: Mervat253

Channel name: High energy : Time 5.5876 +/- 0.0222 minutes

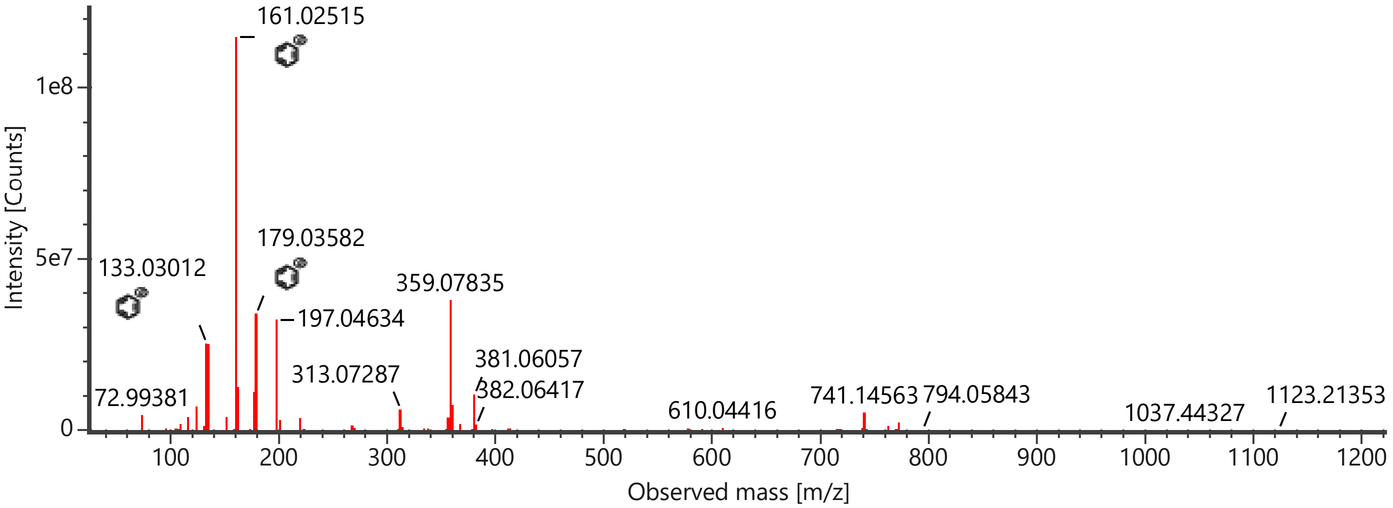

Component name: cis-Ferulic acid

Item name: Sep257-ve
Channel name: cis-Ferulic acid [-H] : (52.5 PPM) 193.0515

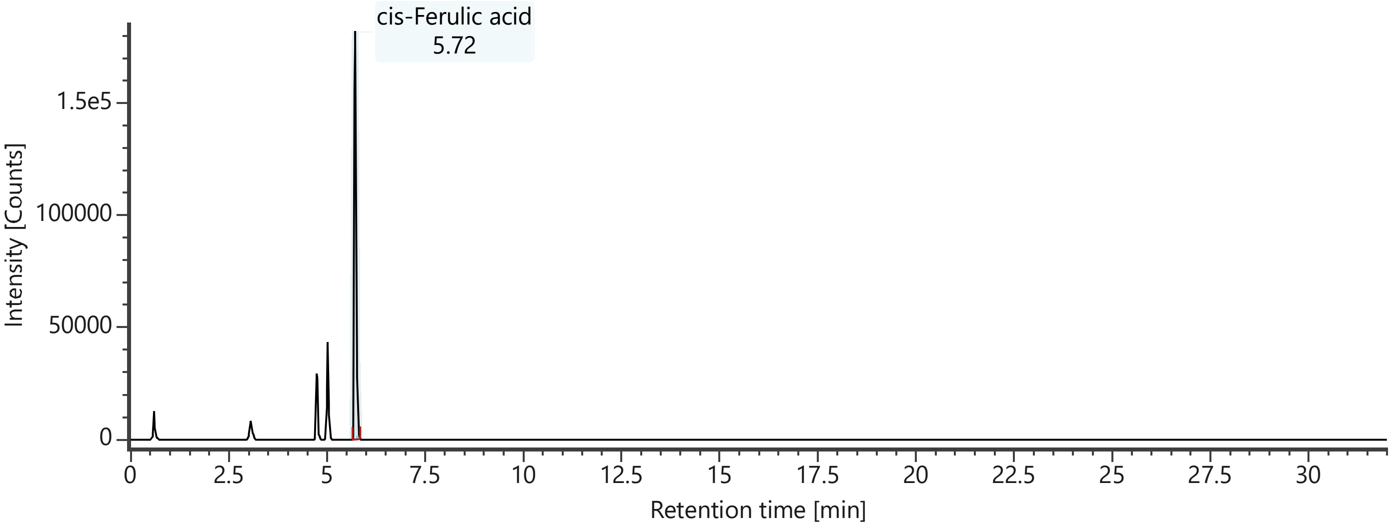

Item name: Sep257-ve
Item description: Mervat253
Channel name: Low energy : Time 5.7182 +/- 0.0222 minutes

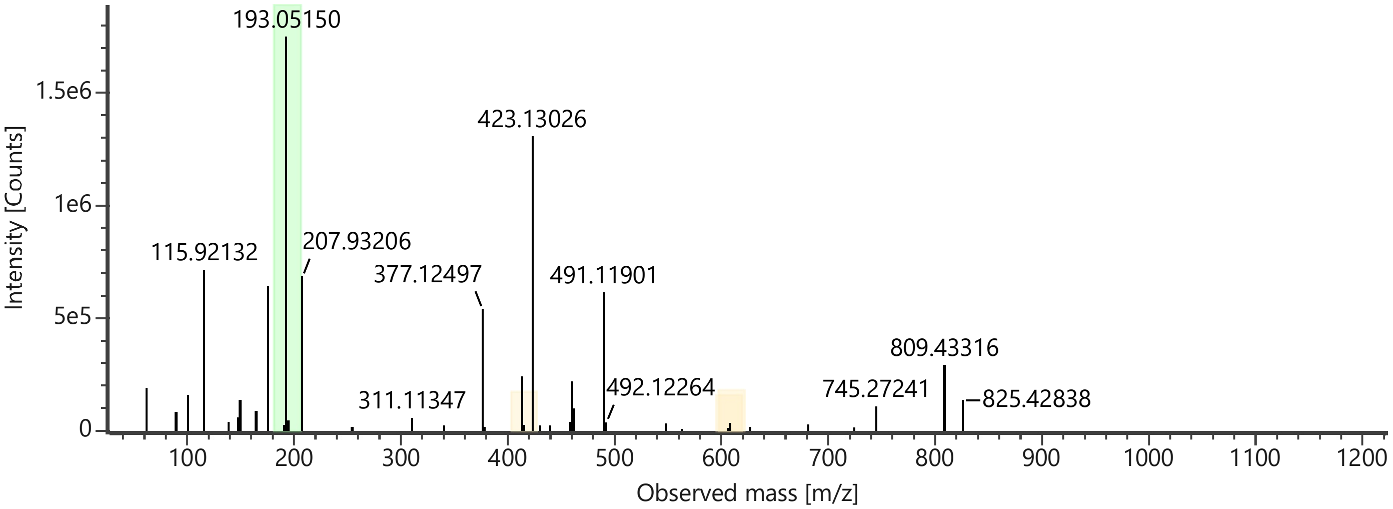

Item name: Sep257-ve
Item description: Mervat253

Channel name: High energy : Time 5.7182 +/- 0.0222 minutes

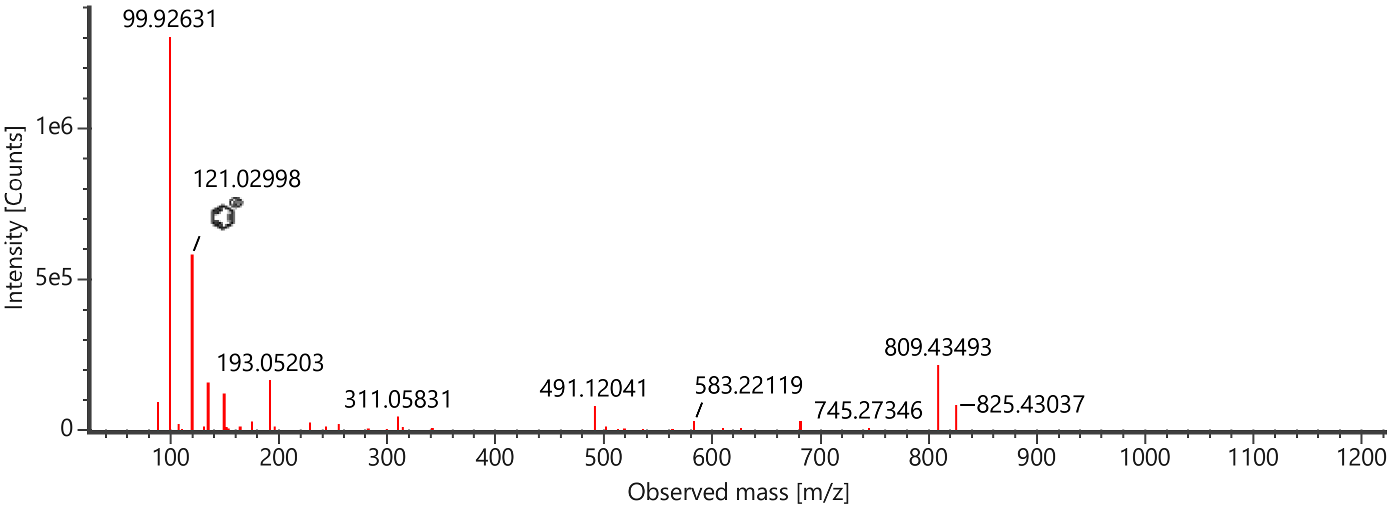

Component name: Quercilicoside A

Item name: Sep257-ve
Channel name: Quercilicoside A [-H] : (52.5 PPM) 665.3906

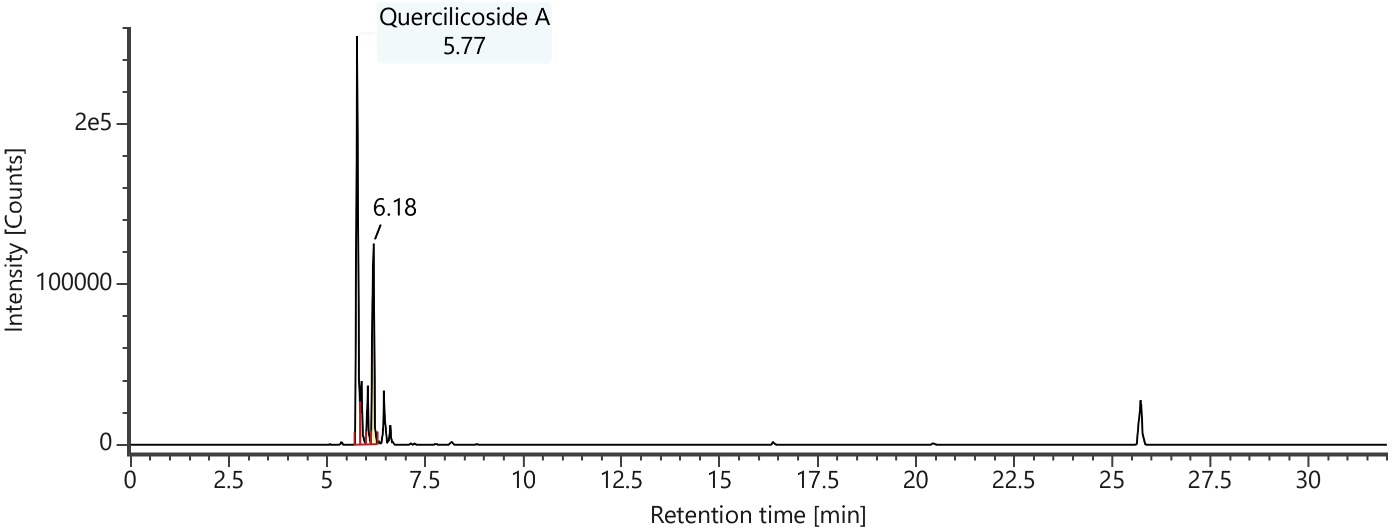

Item name: Sep257-ve
Item description: Mervat253
Channel name: Low energy : Time 5.7702 +/- 0.0222 minutes

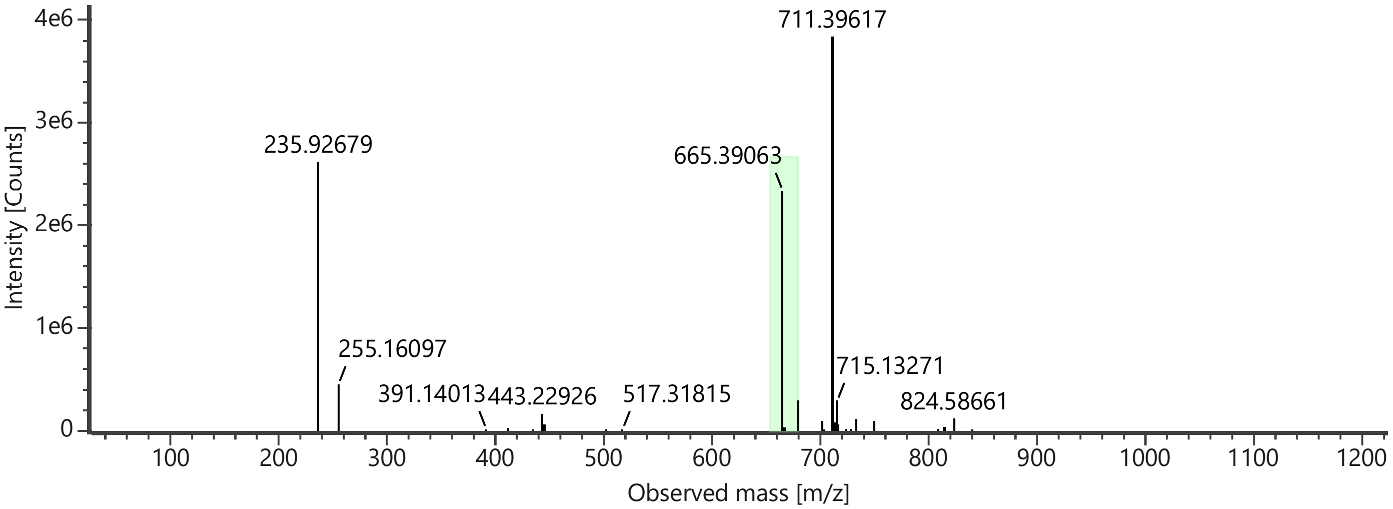

Item name: Sep257-ve
Item description: Mervat253

Channel name: High energy : Time 5.7702 +/- 0.0222 minutes

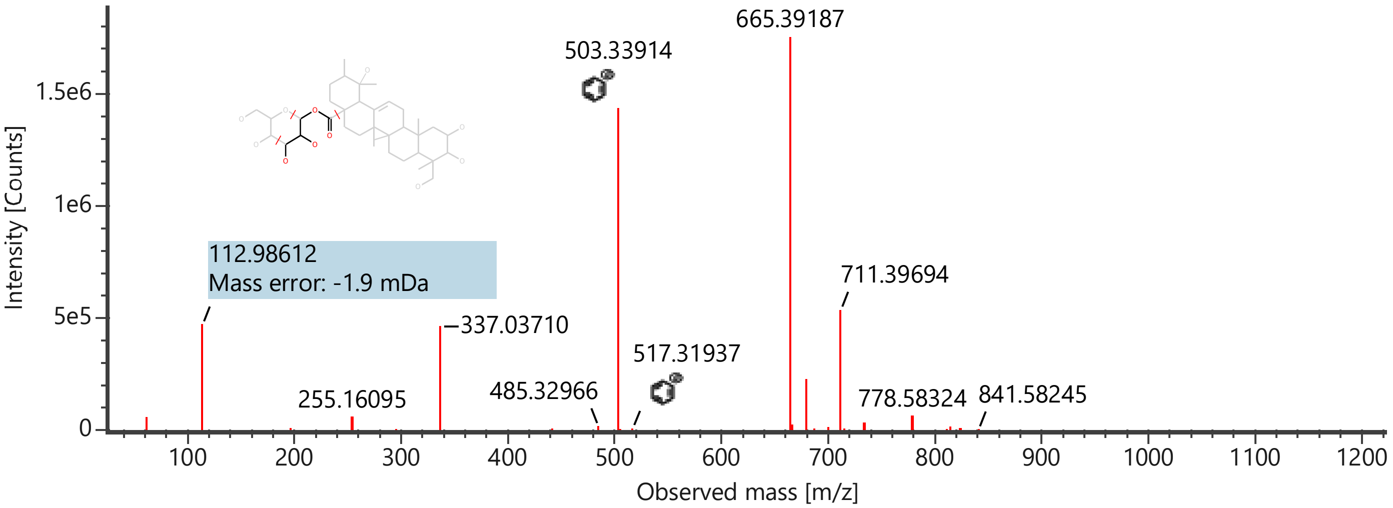

Component name: Kaempferol-3-O-6'-trans-coumaroyl-β-D-glucoside

Item name: Sep257-ve
Channel name: Kaempferol-3-O-6'-trans-coumaroyl-β-D-glucoside [-H] : (52.5 PPM) 593.1315

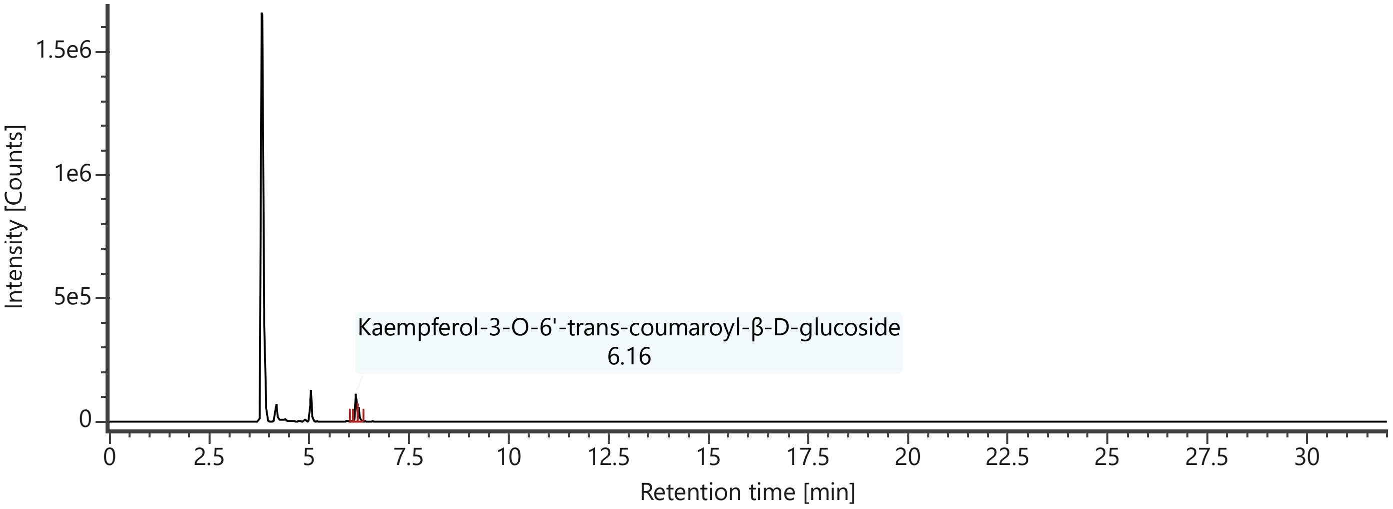

Item name: Sep257-ve
Item description: Mervat253
Channel name: Low energy : Time 6.1626 +/- 0.0222 minutes

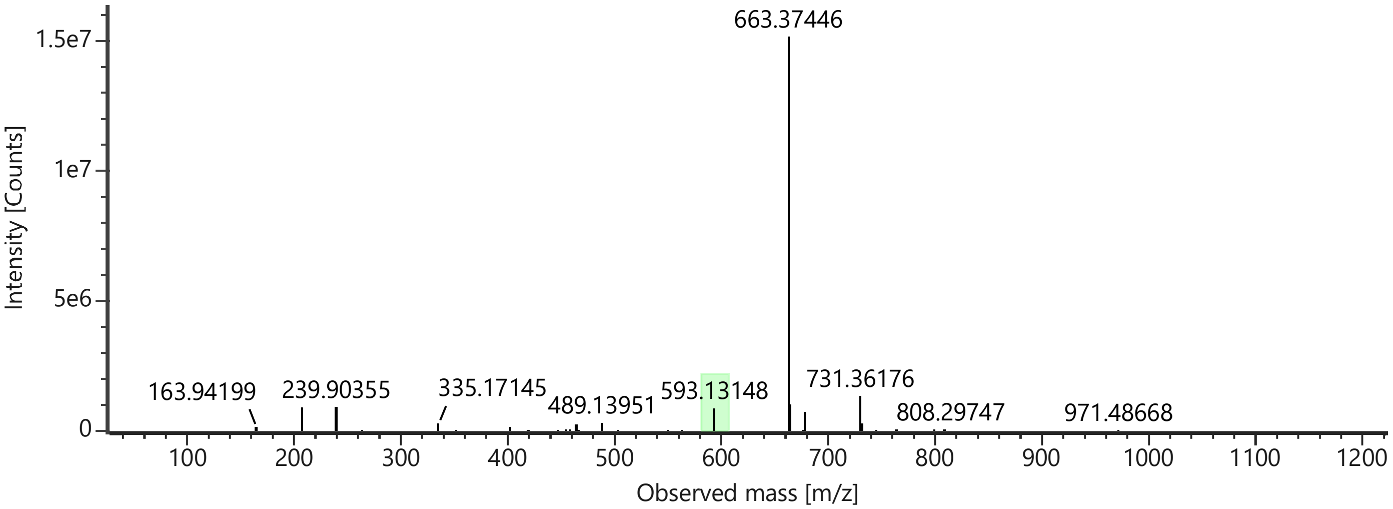

Created by: Ayad, Younan

Created on: Sep 18, 2025

Item name: Lamiaceae family -ve mode

Created time: 13:05:43 Egypt Standard Time

Item name: Sep257-ve

Item description: Mervat253

Channel name: High energy : Time 6.1626 +/- 0.0222 minutes

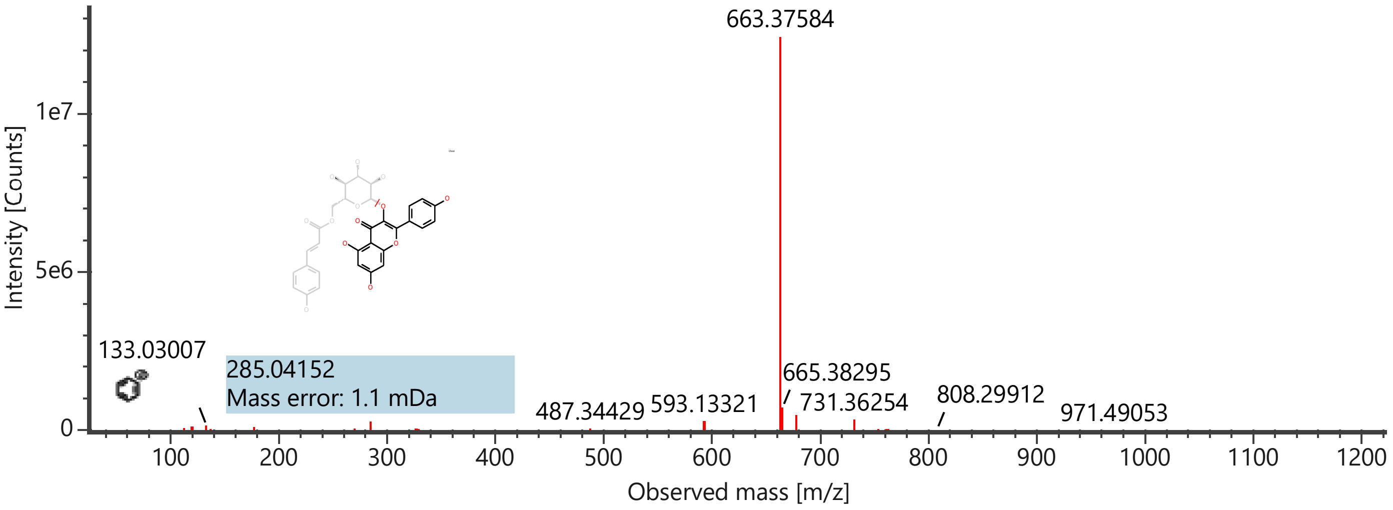

Component name: Casticin

Item name: Sep257-ve
Channel name: Casticin [-H] : (52.5 PPM) 373.0932

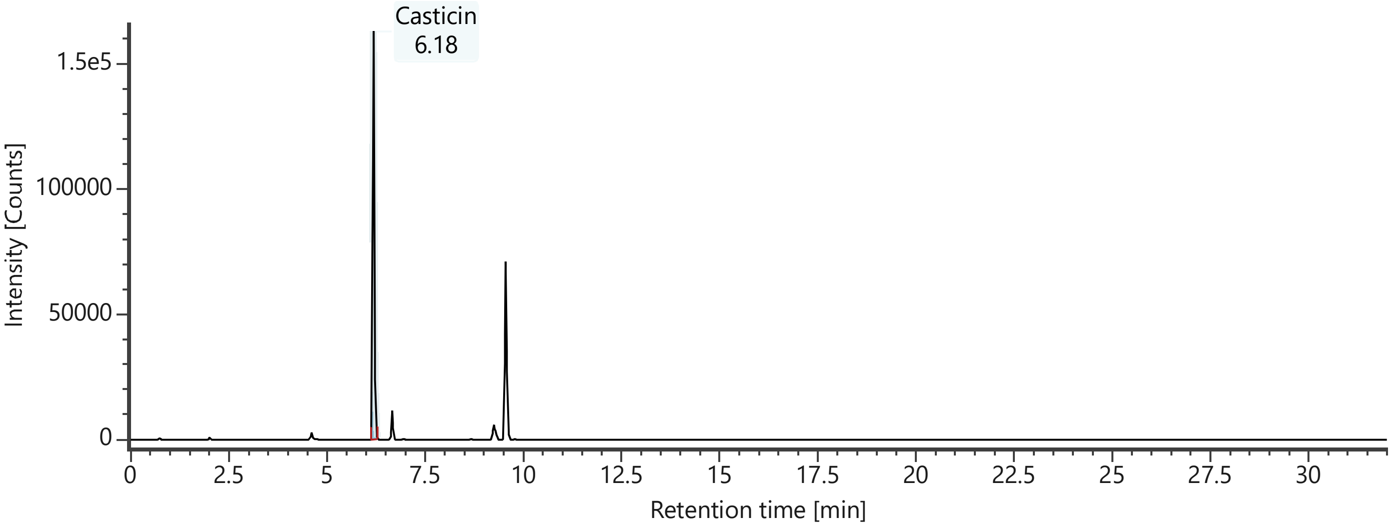

Item name: Sep257-ve
Item description: Mervat253
Channel name: Low energy : Time 6.1858 +/- 0.0222 minutes

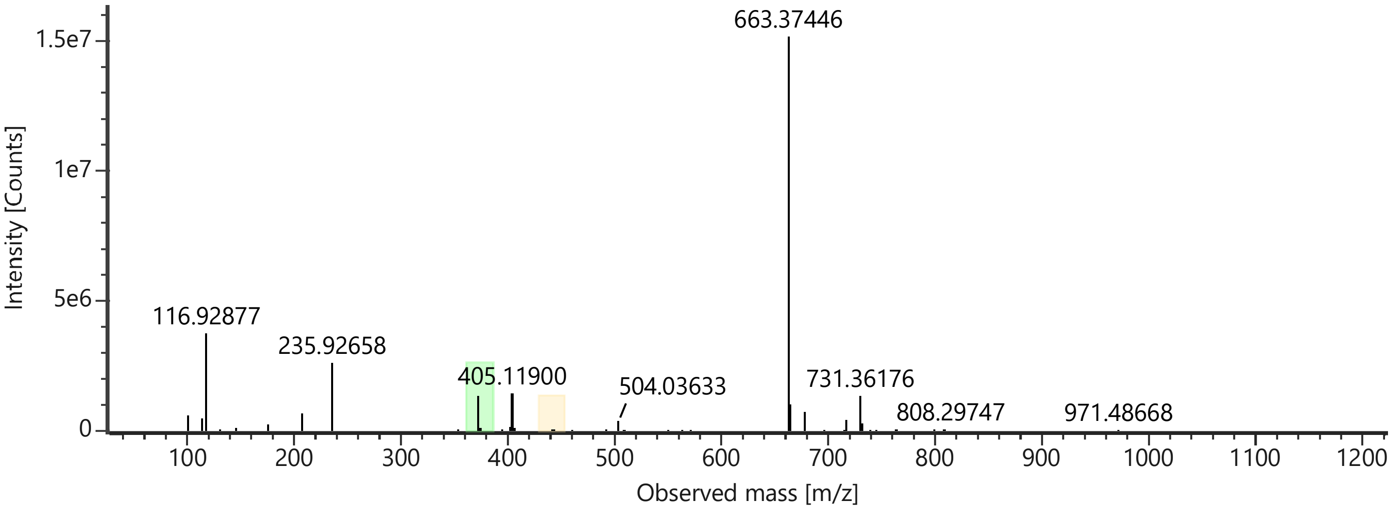

Item name: Sep257-ve
Item description: Mervat253

Channel name: High energy : Time 6.1858 +/- 0.0222 minutes

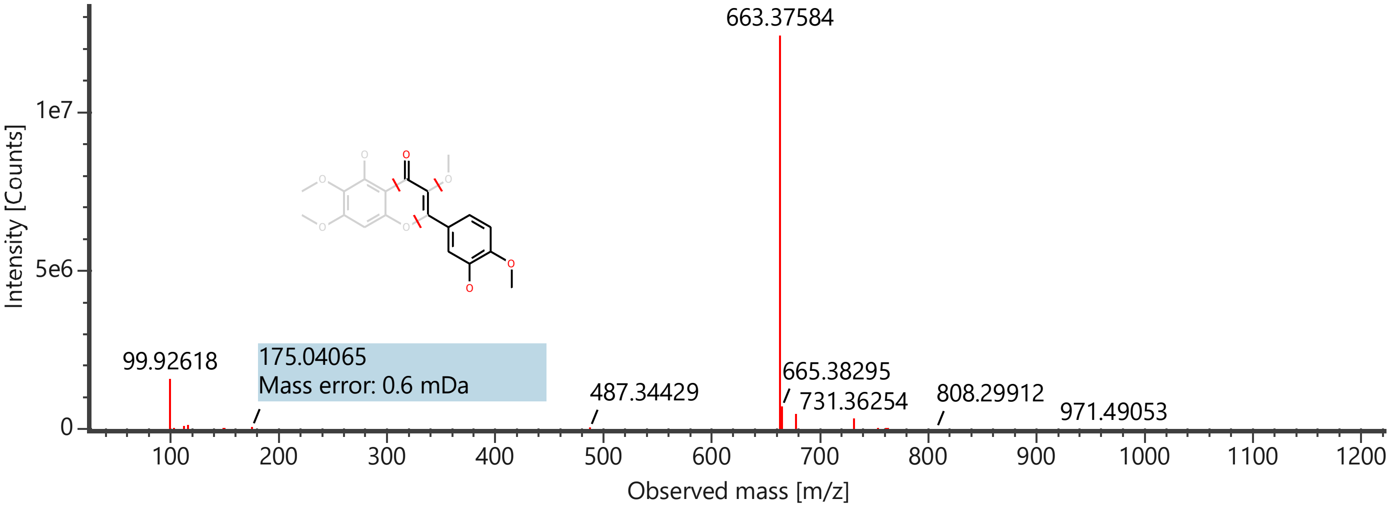

Component name: **1-[2-(3,4-Dihydroxyphenyl)-1-carboxy]ethoxycarbonyl-2-(3,4-dihydroxyphenyl)-7,8-dihydroxy-1,2-dihydronaphthalene-3-carboxylic acid**

Item name: Sep257-ve
Channel name: 1-[2-(3,4-Dihydroxyphenyl)-1-carboxy]ethoxycarbonyl-2-(3,4-dihydroxyphenyl)-7,8-dihydroxy-1,2-dihydronaphthalene-3-carboxylic acid [-H] : (52.5 PPM) 537.1024

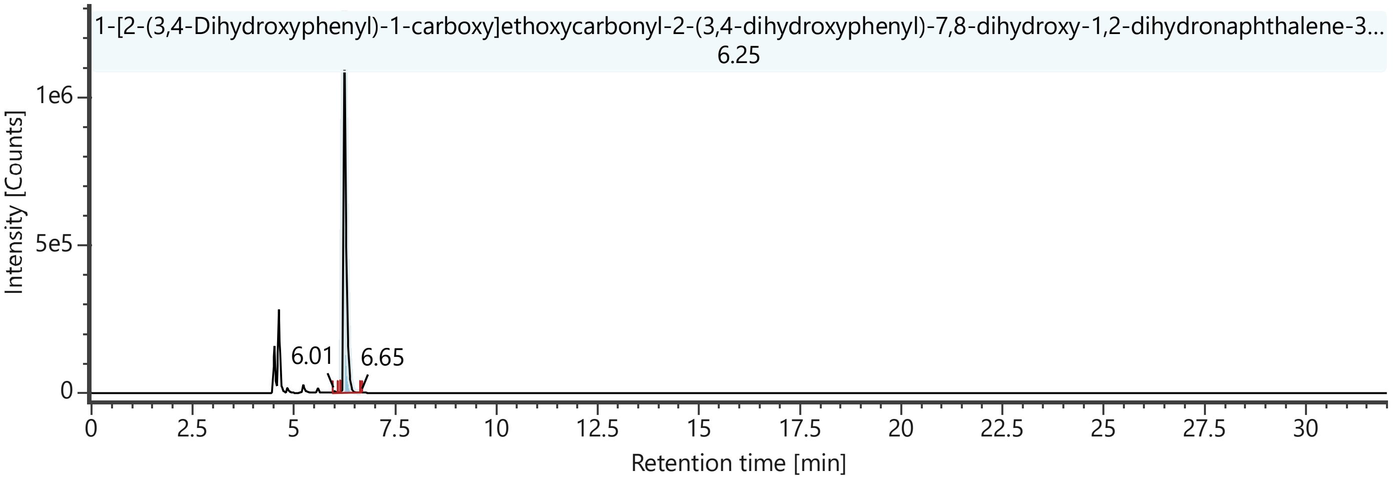

Item name: Sep257-ve
Item description: Mervat253
Channel name: Low energy : Time 6.2497 +/- 0.0222 minutes

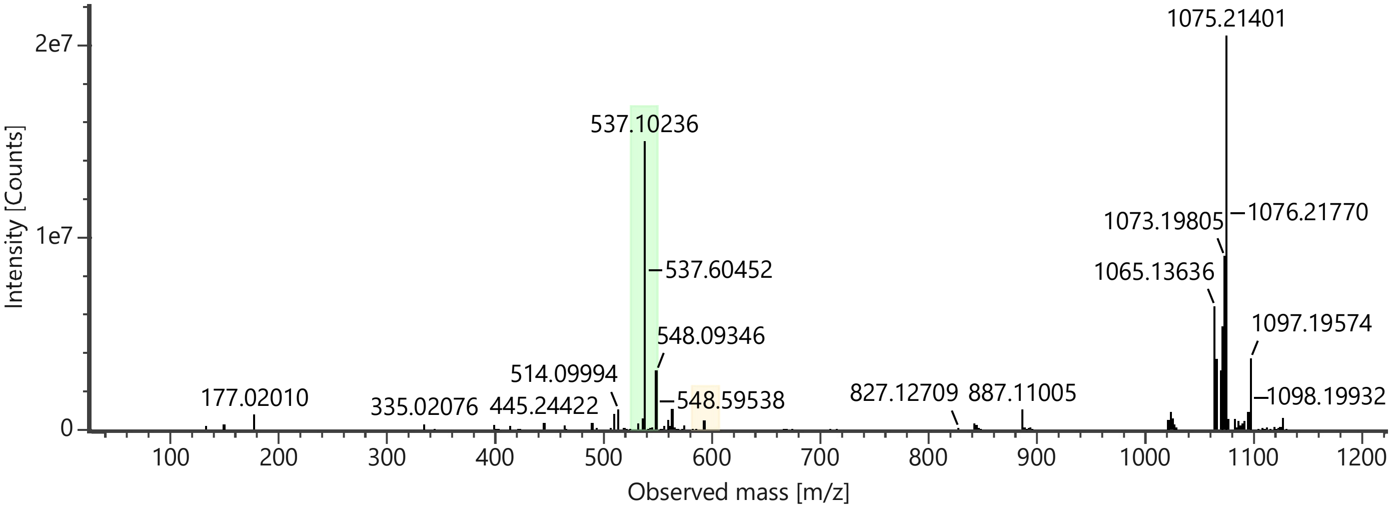

Created by: Ayad, Younan

Created on: Sep 18, 2025

Item name: Lamiaceae family -ve mode

Created time: 13:05:43 Egypt Standard Time

Item name: Sep257-ve
Item description: Mervat253

Channel name: High energy : Time 6.2497 +/- 0.0222 minutes

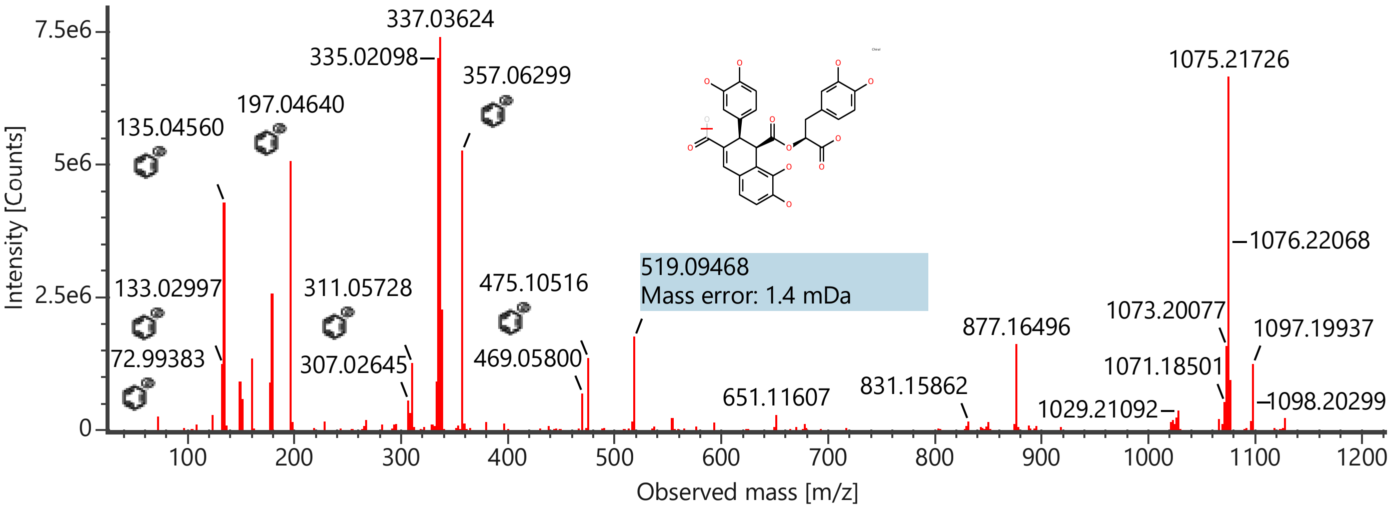

Component name: 6'-O-Caffeoylerigeroside

Item name: Sep257-ve
Channel name: 6'-O-Caffeoylerigeroside [-H] : (52.5 PPM) 435.0937

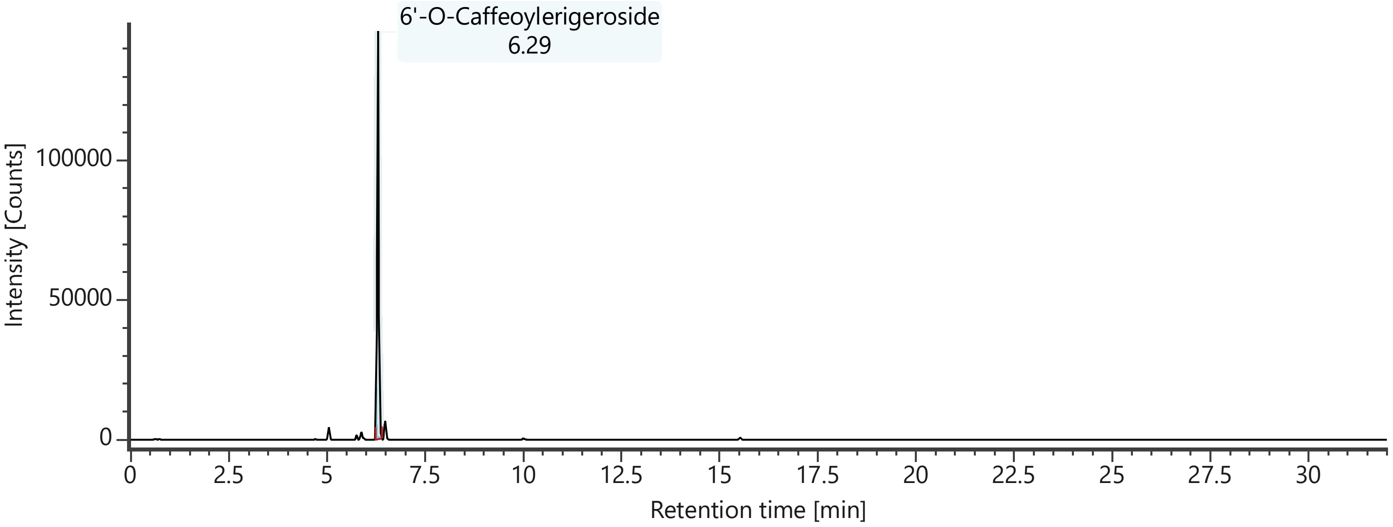

Item name: Sep257-ve
Item description: Mervat253
Channel name: Low energy : Time 6.2956 +/- 0.0222 minutes

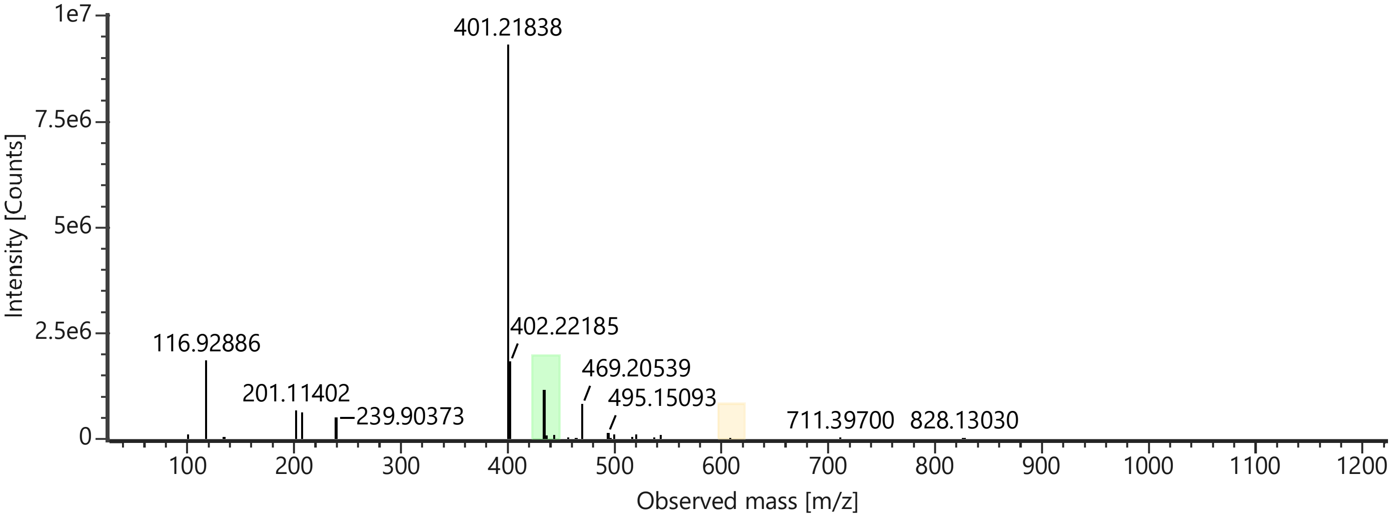

Created by: Ayad, Younan

Created on: Sep 18, 2025

Item name: Lamiaceae family -ve mode

Created time: 13:05:43 Egypt Standard Time

Item name: Sep257-ve
Item description: Mervat253

Channel name: High energy : Time 6.2956 +/- 0.0222 minutes

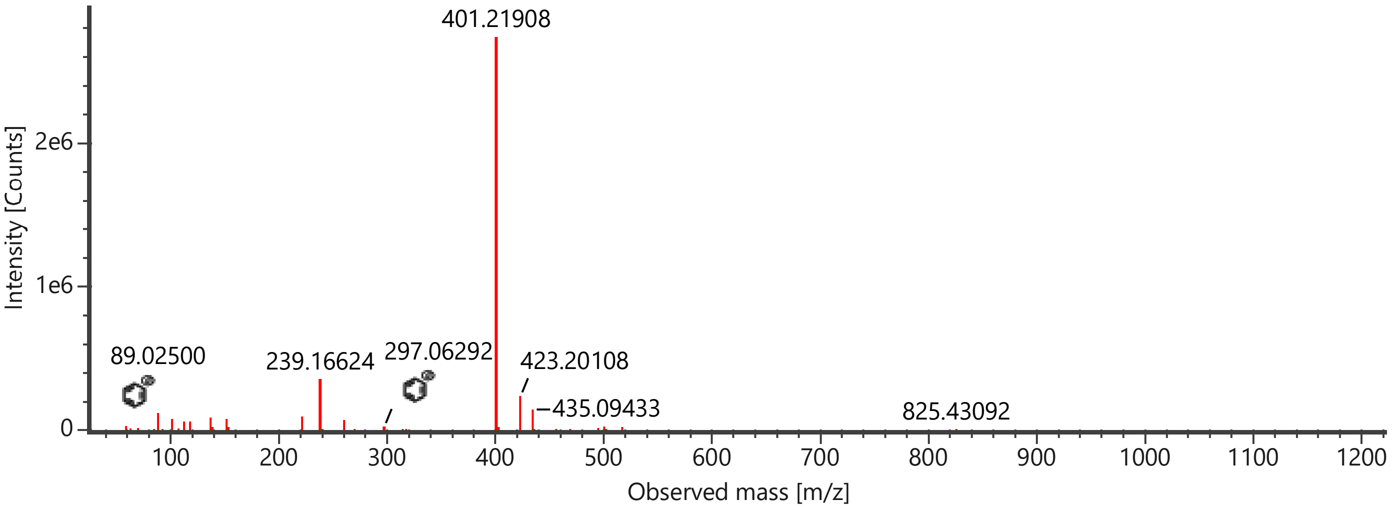

Component name: Luteolin

Item name: Sep257-ve
Channel name: Luteolin [-H] : (52.5 PPM) 285.0408

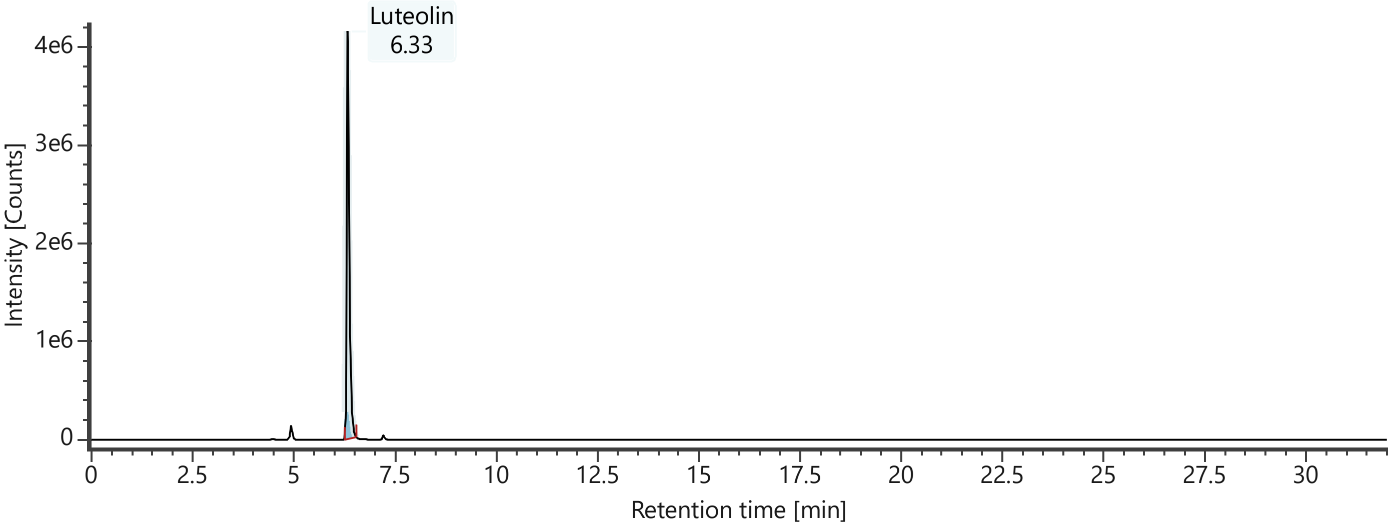

Item name: Sep257-ve
Item description: Mervat253
Channel name: Low energy : Time 6.3298 +/- 0.0222 minutes

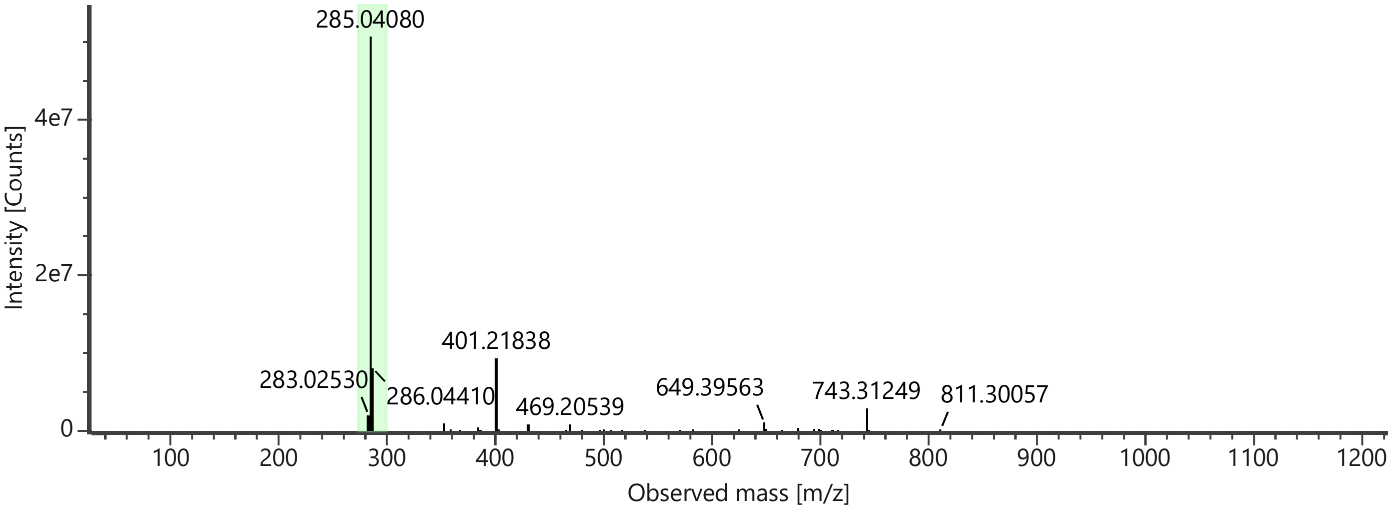

Item name: Sep257-ve
Item description: Mervat253

Channel name: High energy : Time 6.3298 +/- 0.0222 minutes

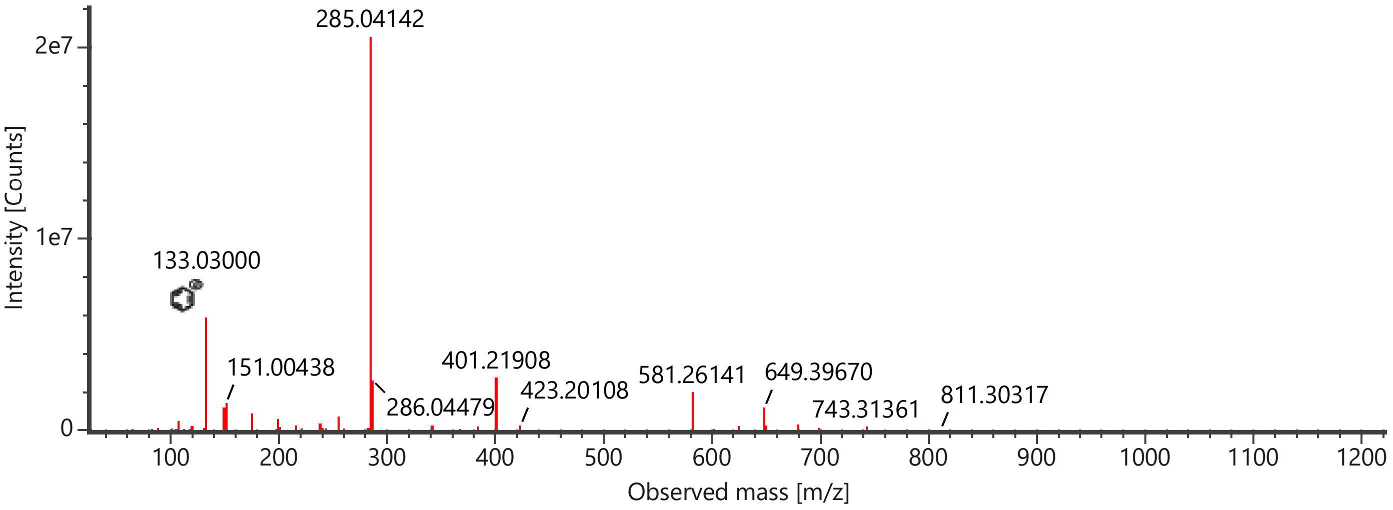

Component name: Quercetin 3'-methyl ether

Item name: Sep257-ve
Channel name: Quercetin 3'-methyl ether [-H] : (52.5 PPM) 315.0512

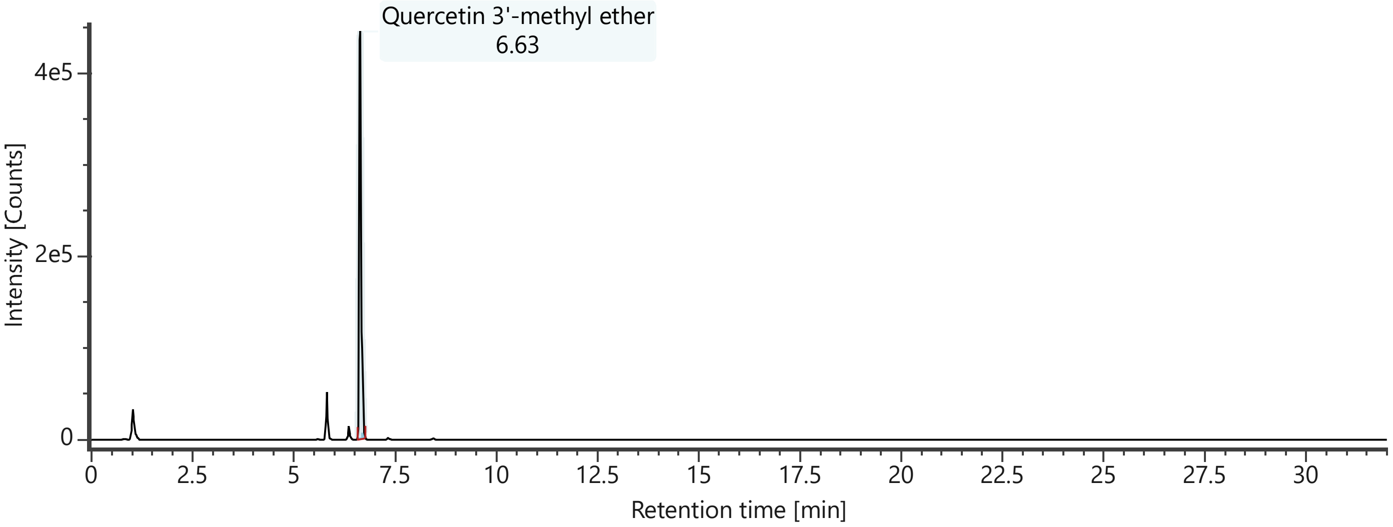

Item name: Sep257-ve
Item description: Mervat253
Channel name: Low energy : Time 6.6332 +/- 0.0222 minutes

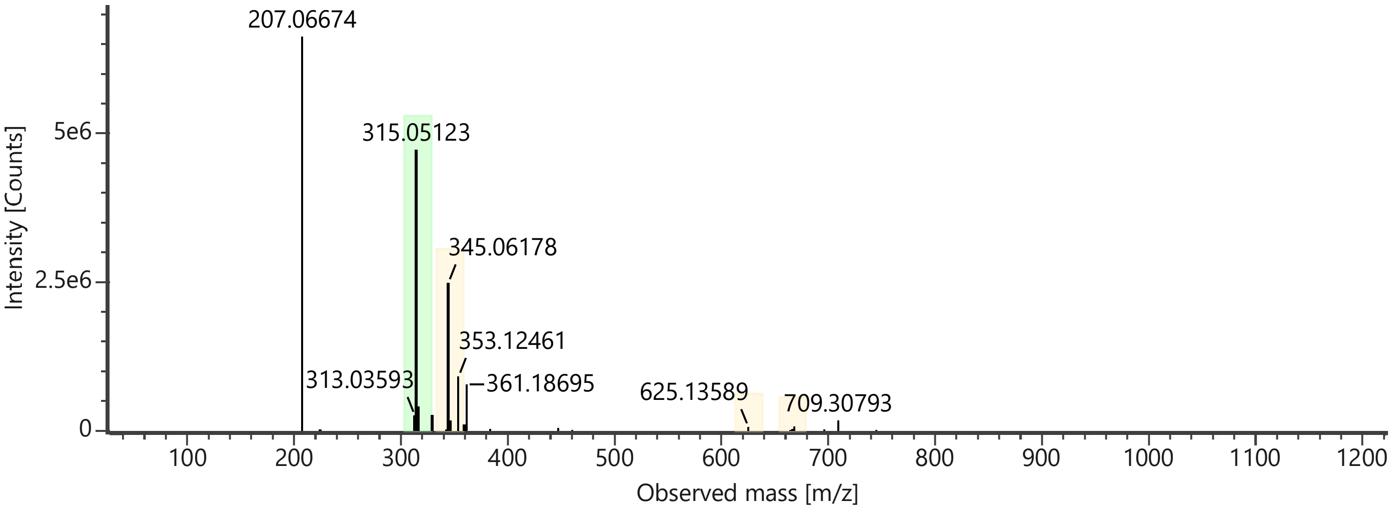

Item name: Sep257-ve
Item description: Mervat253

Channel name: High energy : Time 6.6332 +/- 0.0222 minutes

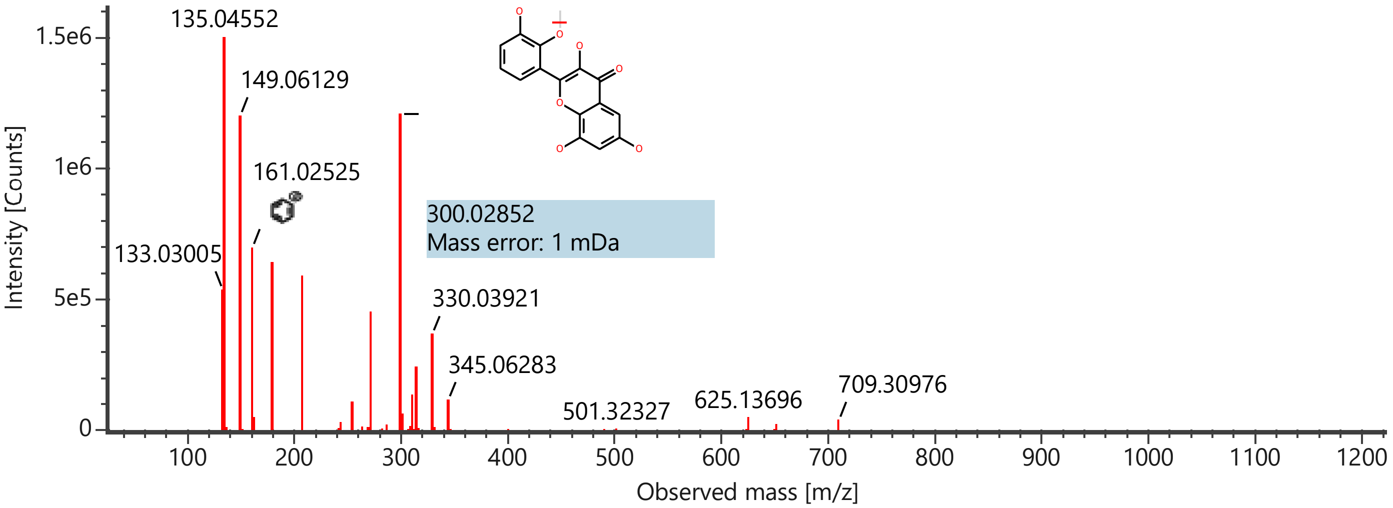

Component name: Quercetagetin 3,4'-Dimethyl Ether

Item name: Sep257-ve
Channel name: Quercetagetin 3,4'-Dimethyl Ether [-H] : (52.5 PPM) 345.0618

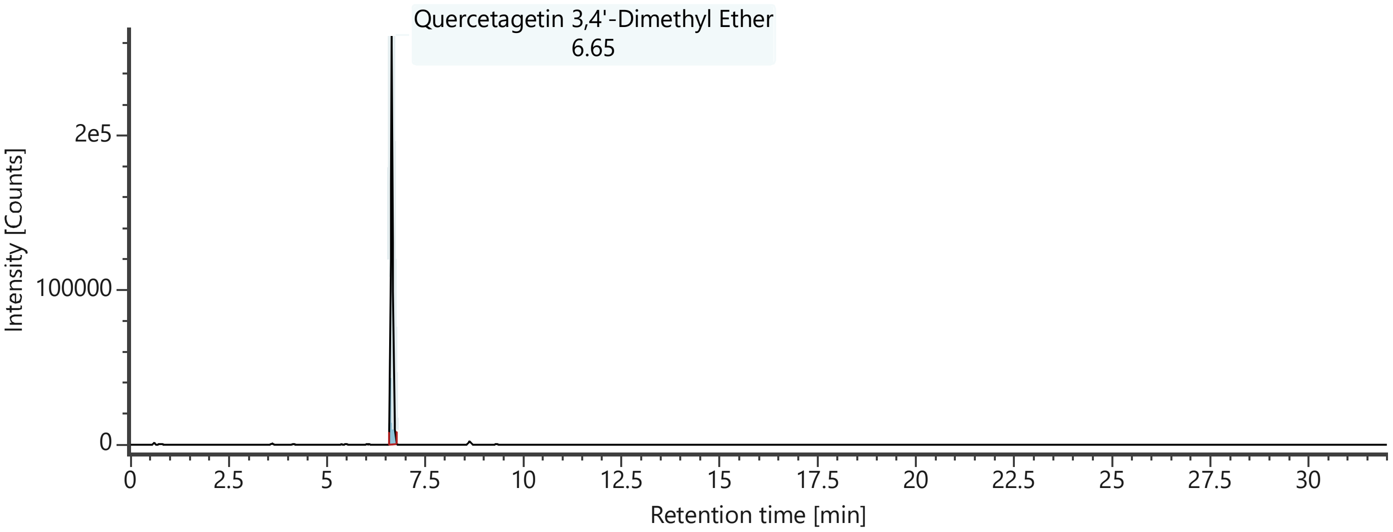

Item name: Sep257-ve
Item description: Mervat253
Channel name: Low energy : Time 6.6521 +/- 0.0222 minutes

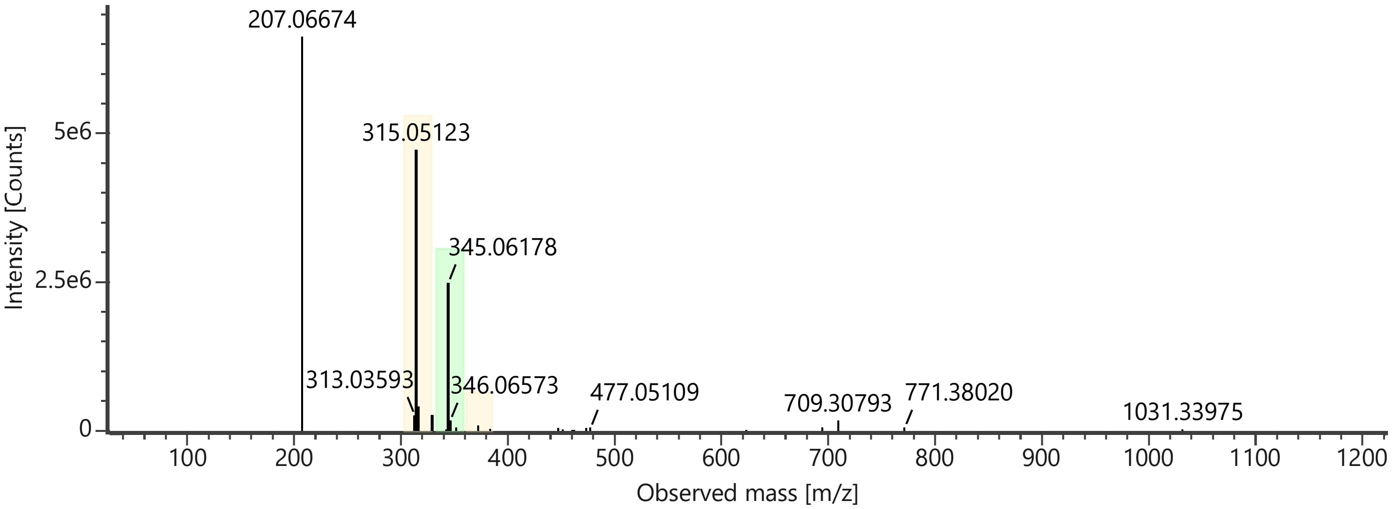

Item name: Sep257-ve
Item description: Mervat253

Channel name: High energy : Time 6.6521 +/- 0.0222 minutes

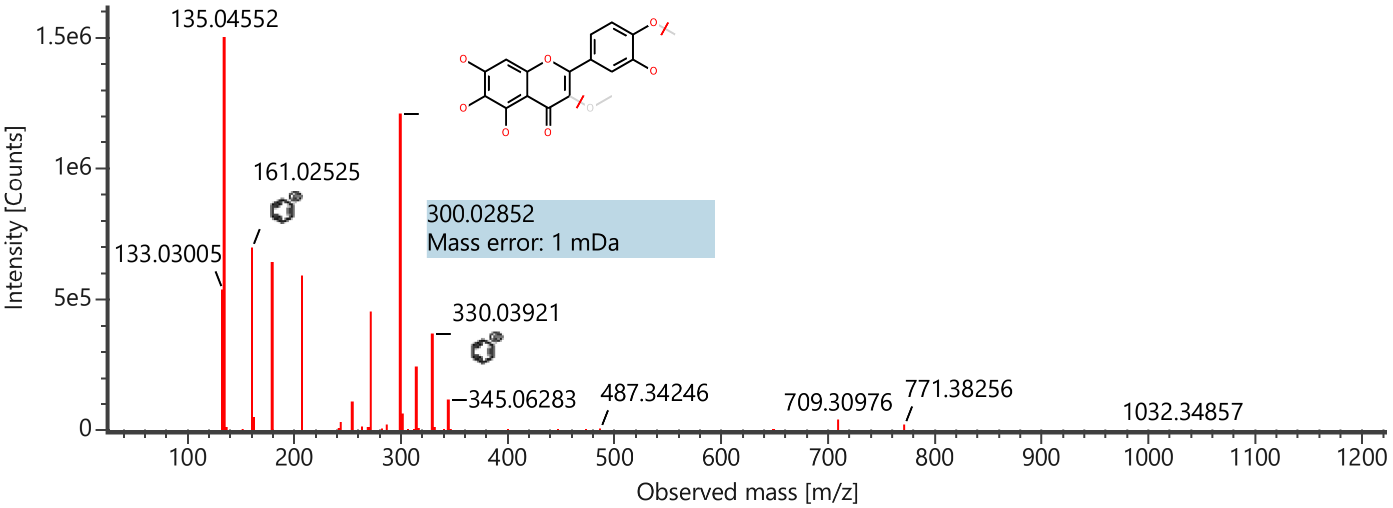

Component name: 3,5-Dihydroxy-4',7-dimethoxyflavone

Item name: Sep257-ve
Channel name: 3,5-Dihydroxy-4',7-dimethoxyflavone [-H] : (52.5 PPM) 313.0719

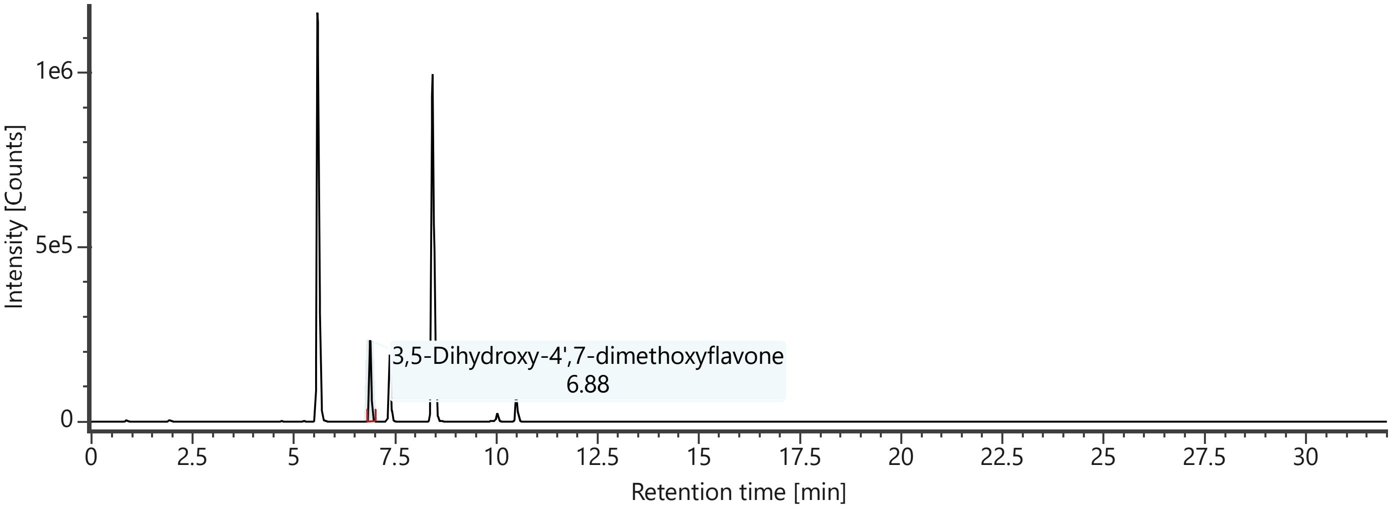

Item name: Sep257-ve
Item description: Mervat253
Channel name: Low energy : Time 6.8834 +/- 0.0222 minutes

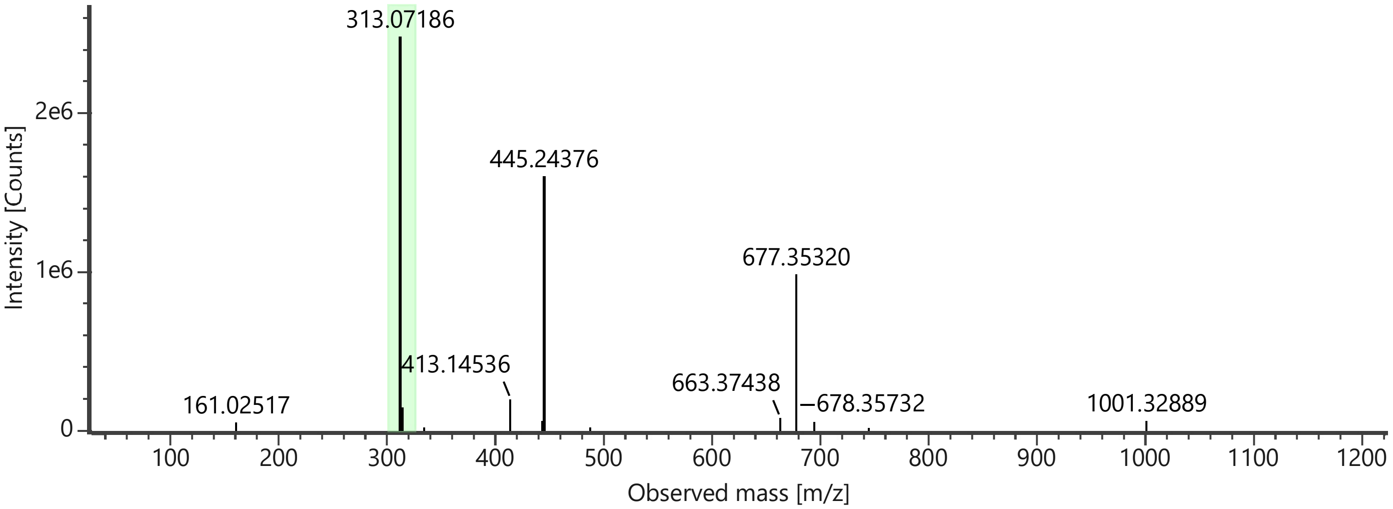

Item name: Sep257-ve
Item description: Mervat253

Channel name: High energy : Time 6.8834 +/- 0.0222 minutes

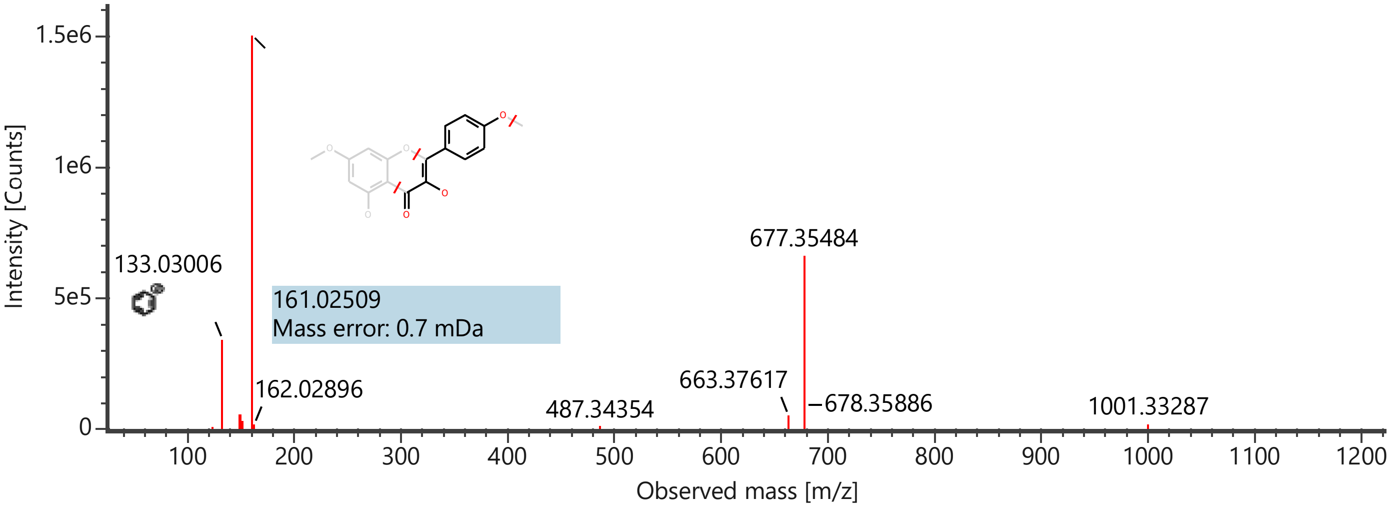

Component name: Apigenin

Item name: Sep257-ve
Channel name: Apigenin [-H] : (52.5 PPM) 269.0459

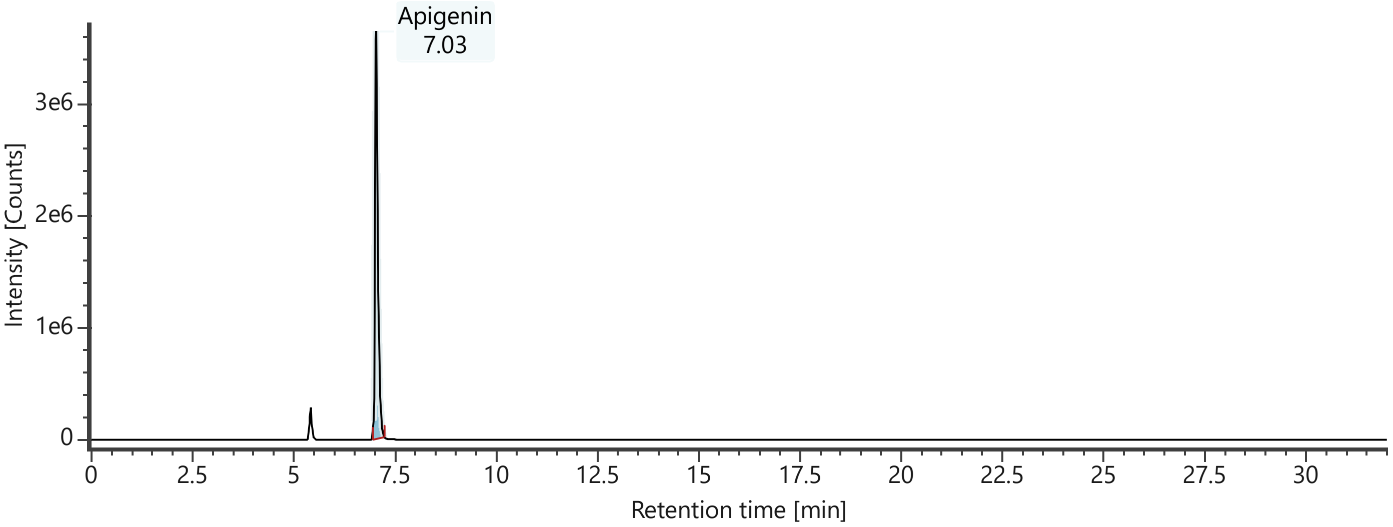

Item name: Sep257-ve
Item description: Mervat253
Channel name: Low energy : Time 7.0301 +/- 0.0222 minutes

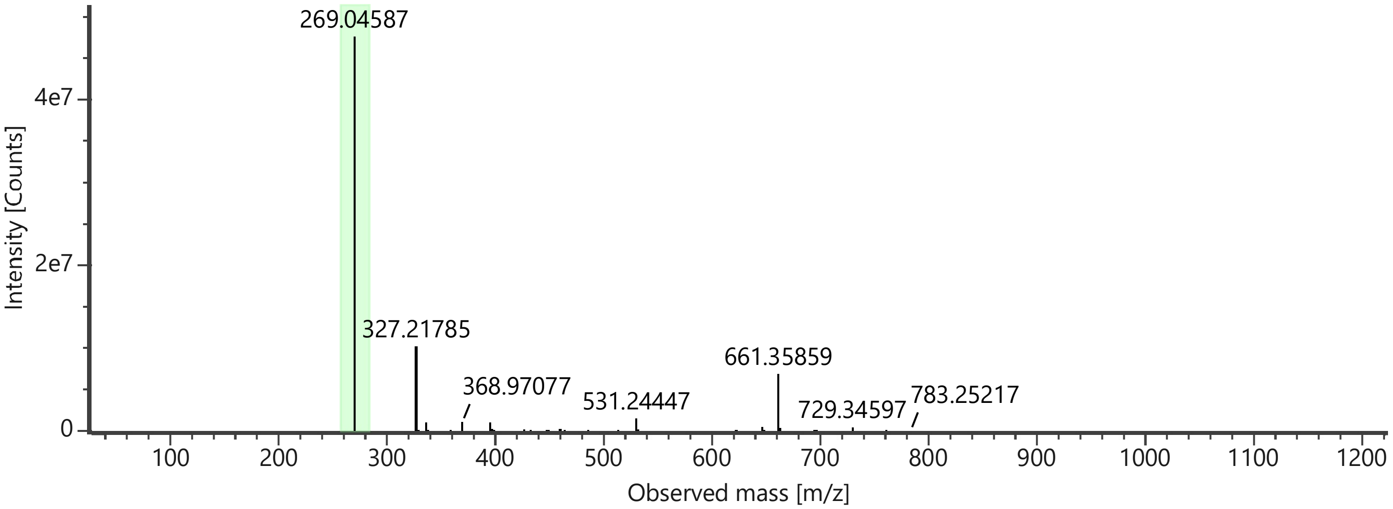

Item name: Sep257-ve
Item description: Mervat253

Channel name: High energy : Time 7.0301 +/- 0.0222 minutes

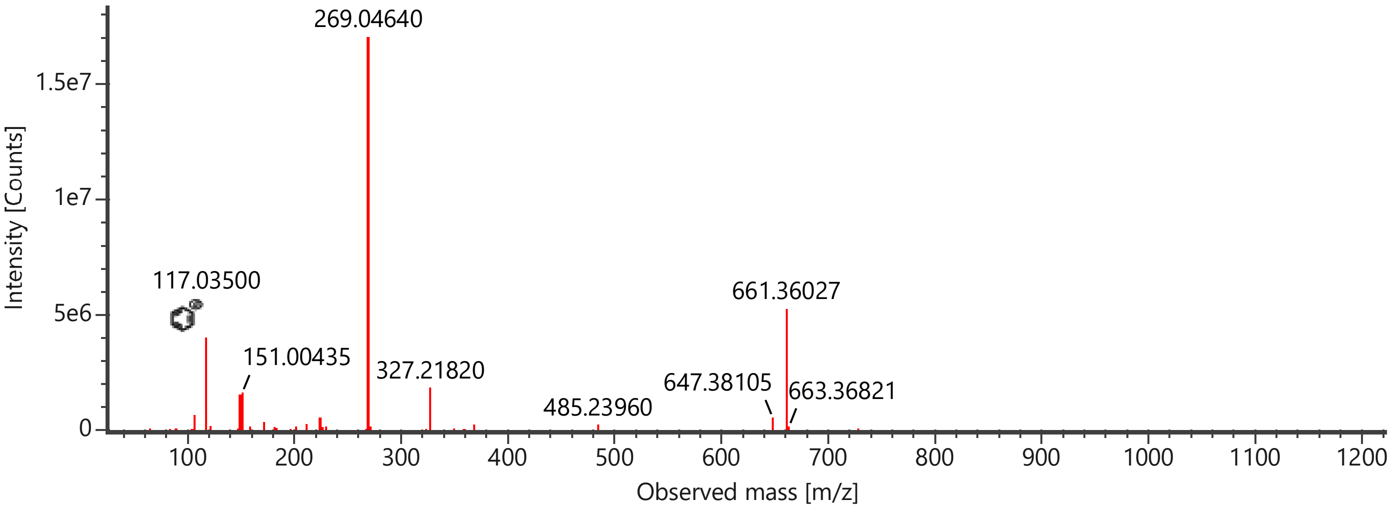

Component name: 3'-Hydroxygenkwanin

Item name: Sep257-ve
Channel name: 3'-Hydroxygenkwanin [-H] : (52.5 PPM) 299.0564

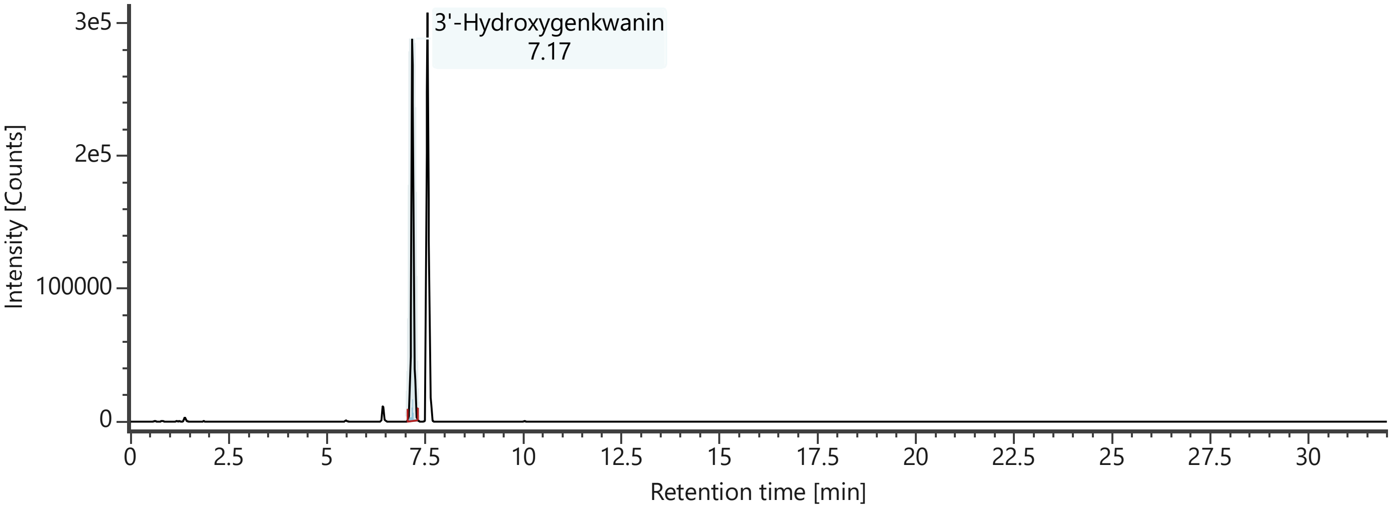

Item name: Sep257-ve
Item description: Mervat253
Channel name: Low energy : Time 7.1755 +/- 0.0222 minutes

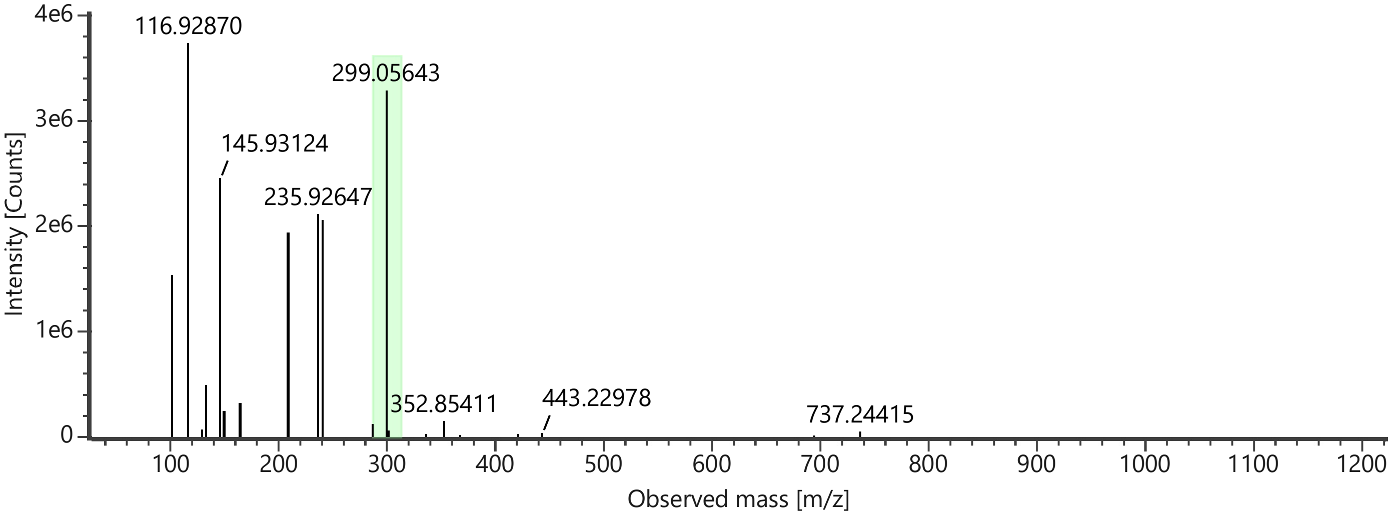

Item name: Sep257-ve
Item description: Mervat253

Channel name: High energy : Time 7.1755 +/- 0.0222 minutes

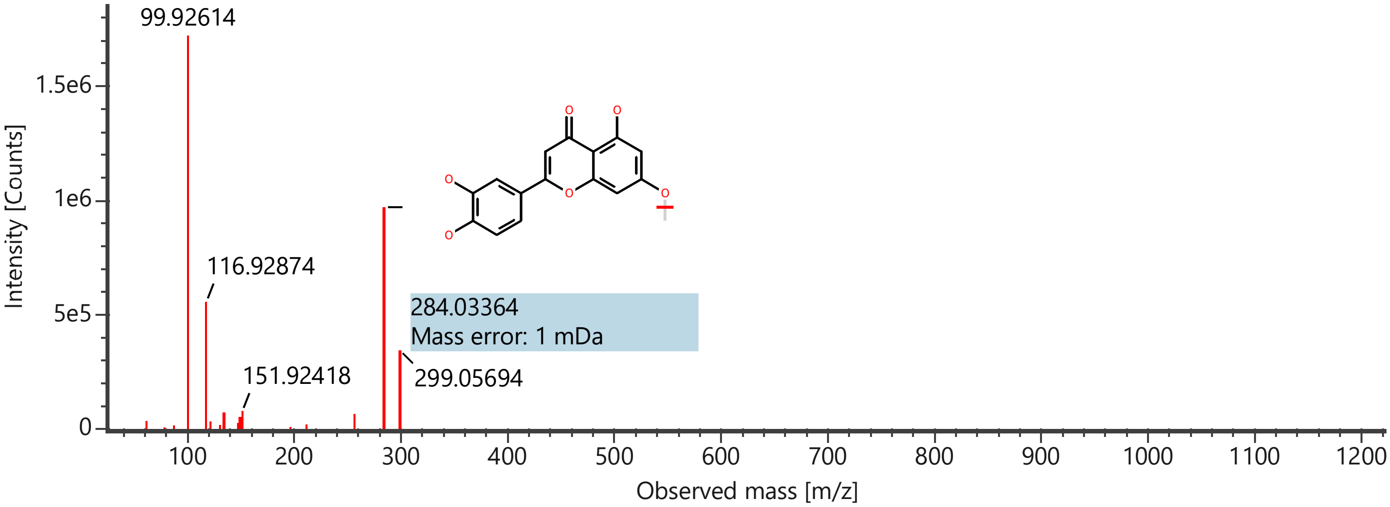

Component name: Ombuin

Item name: Sep257-ve
Channel name: Ombuin [-H] : (52.5 PPM) 329.0667

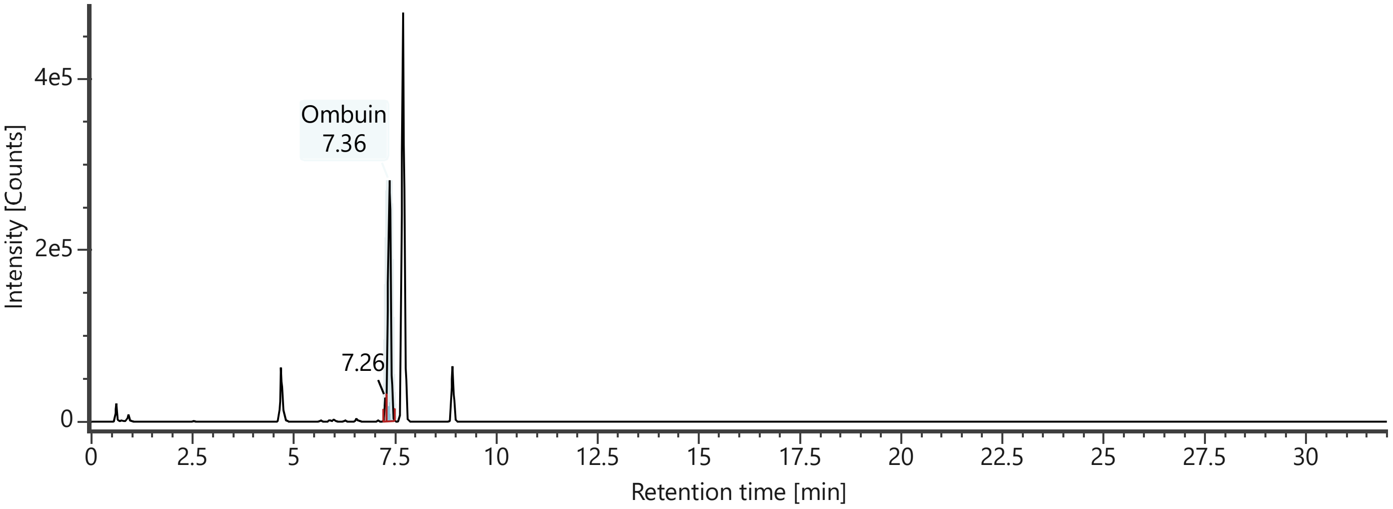

Item name: Sep257-ve
Item description: Mervat253
Channel name: Low energy : Time 7.3583 +/- 0.0222 minutes

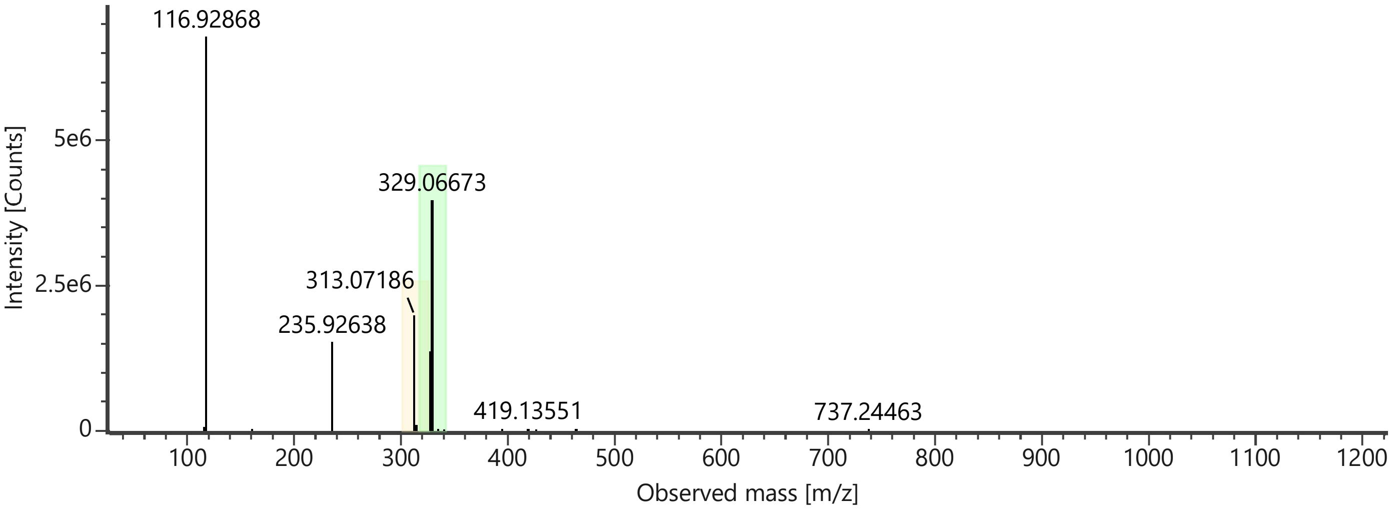

Item name: Sep257-ve
Item description: Mervat253

Channel name: High energy : Time 7.3583 +/- 0.0222 minutes

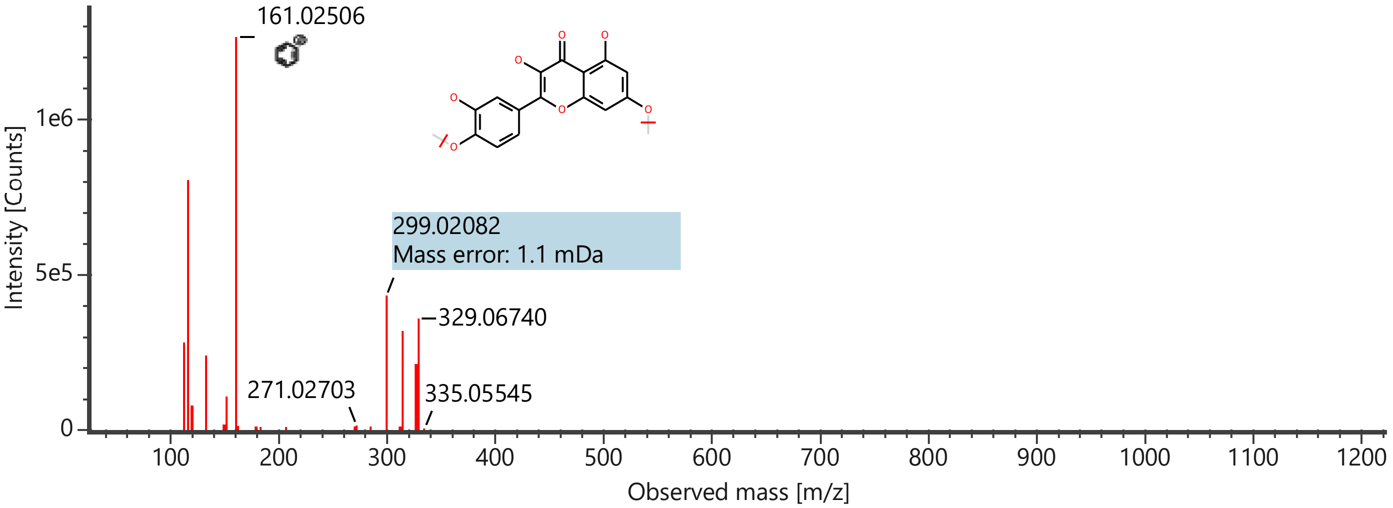

Component name: 3,5-Dihydroxy-4',7-dimethoxyflavone

Item name: Sep257-ve
Channel name: 3,5-Dihydroxy-4',7-dimethoxyflavone [-H] : (52.5 PPM) 313.0719

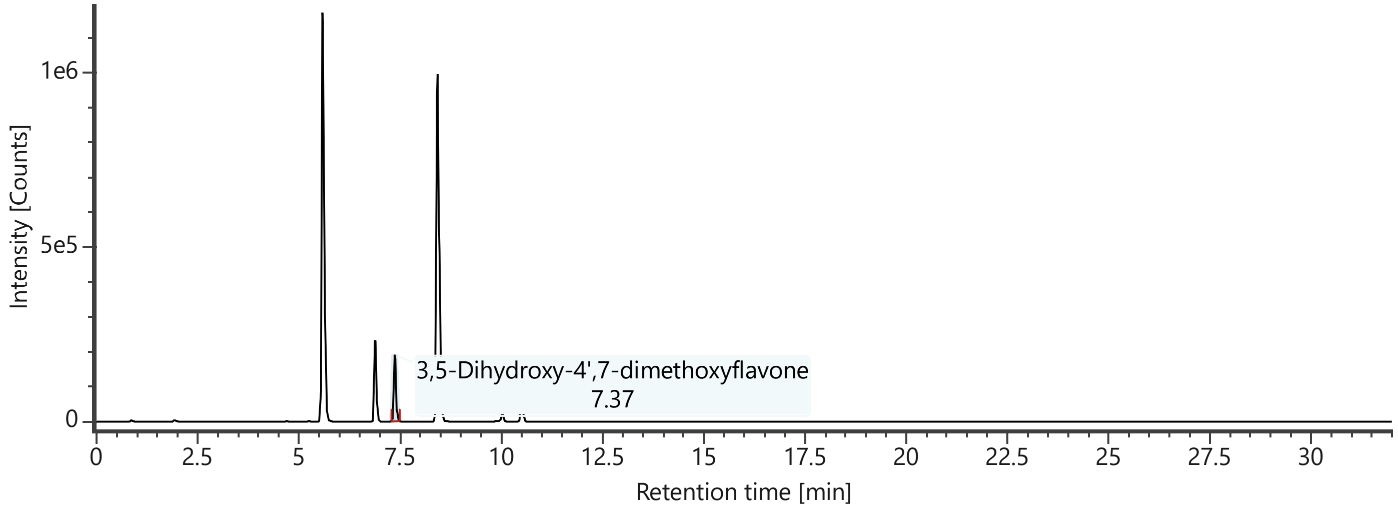

Item name: Sep257-ve
Item description: Mervat253
Channel name: Low energy : Time 7.3680 +/- 0.0222 minutes

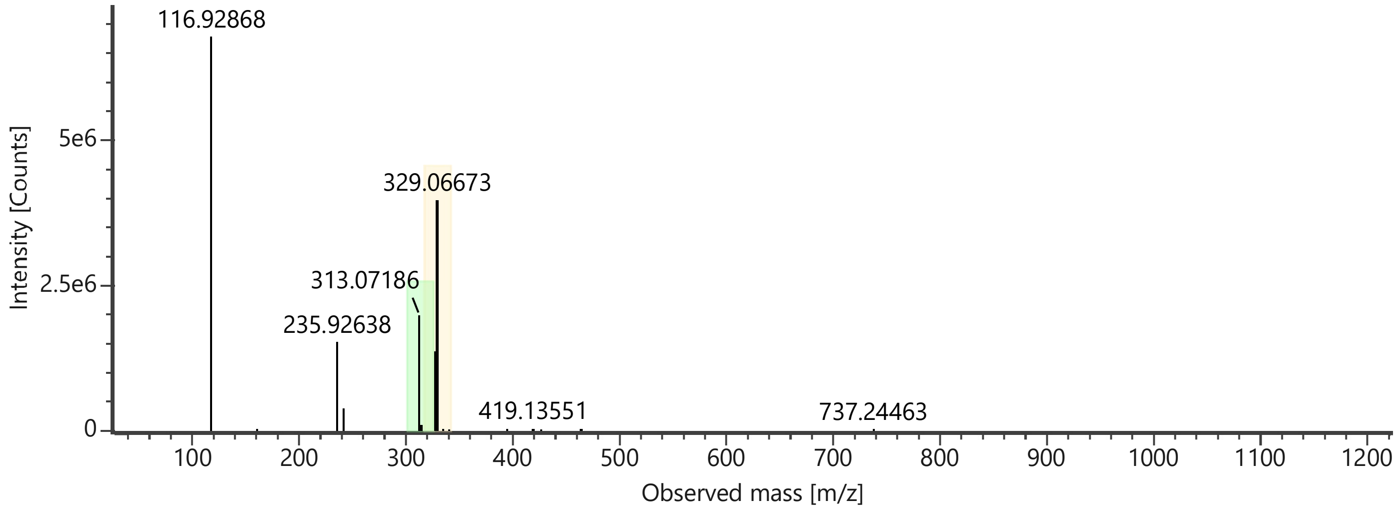

Item name: Lamiaceae family -ve mode

Created by: Ayad, Younan

Created on: Sep 18, 2025

Created time: 13:05:43 Egypt Standard Time

Item name: Sep257-ve
Item description: Mervat253

Channel name: High energy : Time 7.3680 +/- 0.0222 minutes

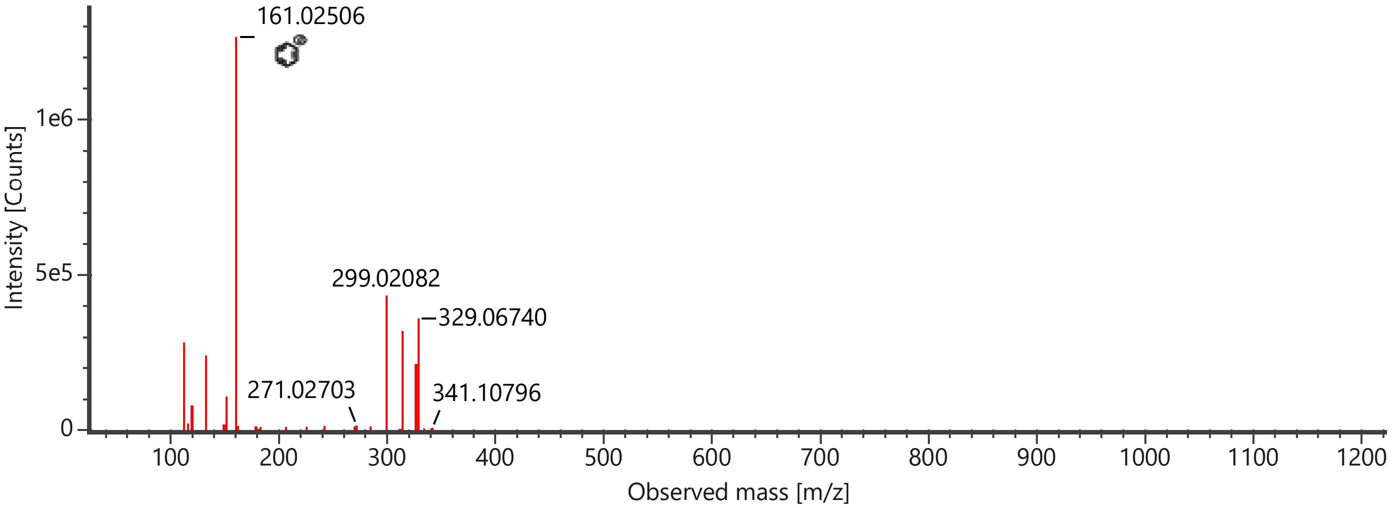

Component name: Kaempferide

Item name: Sep257-ve
Channel name: Kaempferide [-H] : (52.5 PPM) 299.0564

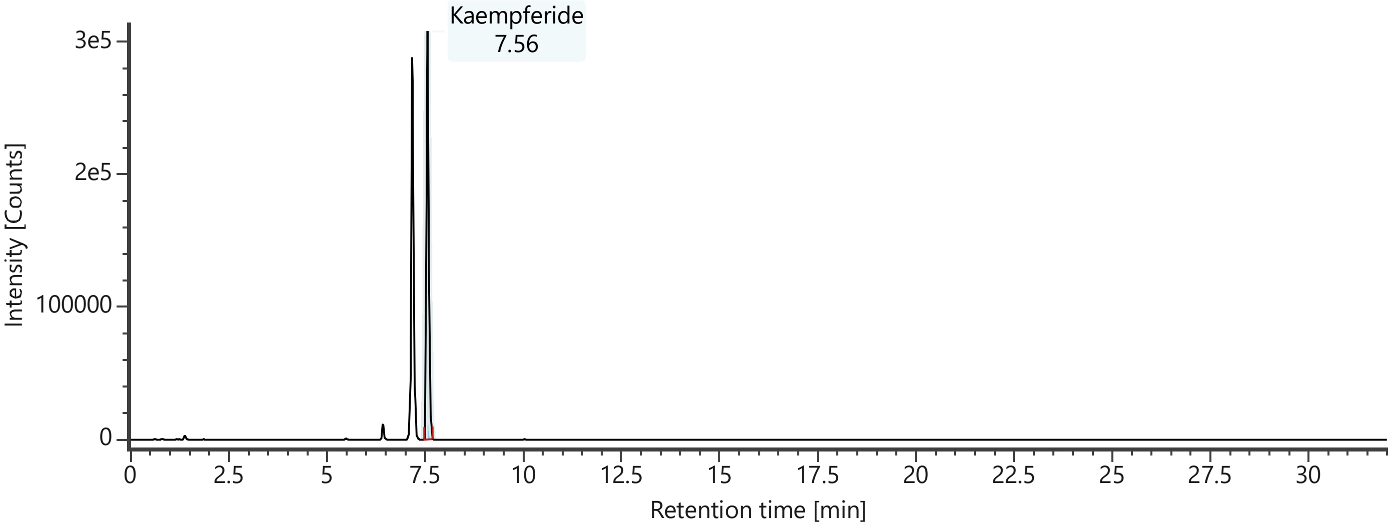

Item name: Sep257-ve
Item description: Mervat253
Channel name: Low energy : Time 7.5641 +/- 0.0222 minutes

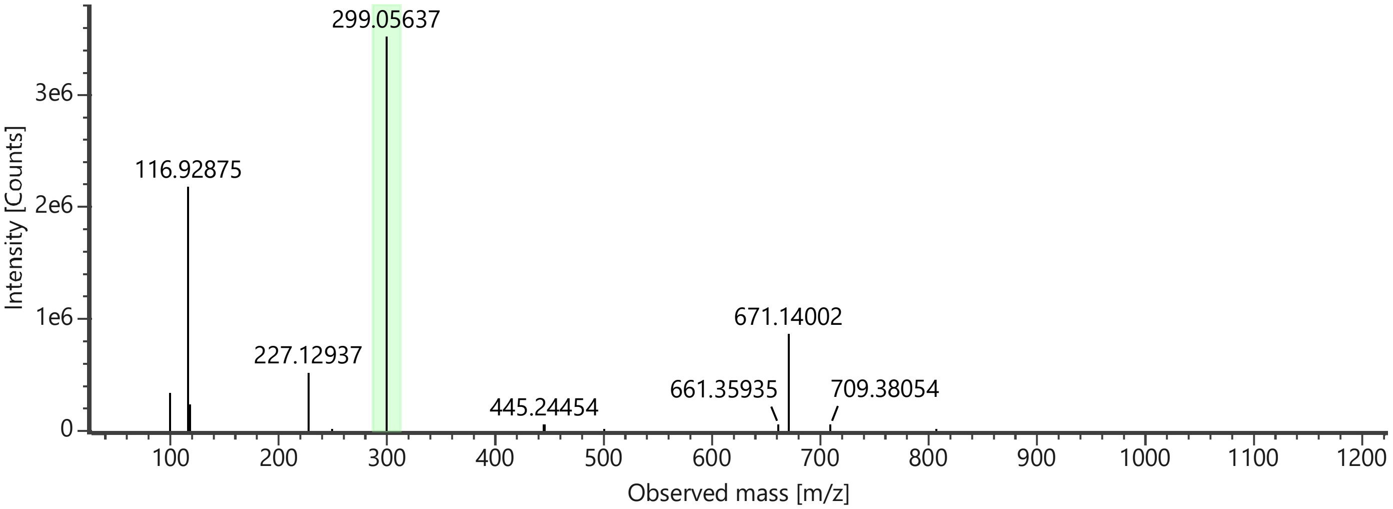

Item name: Sep257-ve
Item description: Mervat253

Channel name: High energy : Time 7.5641 +/- 0.0222 minutes

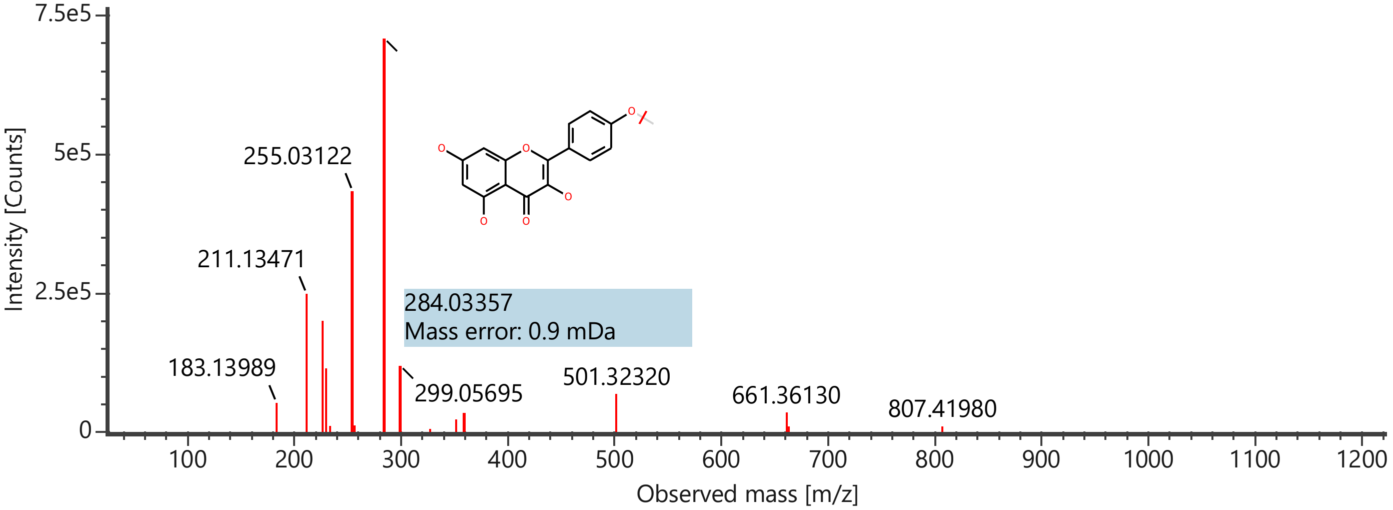

Component name: Quercetin 3,4'-dimethyl ether

Item name: Sep257-ve
Channel name: Quercetin 3,4'-dimethyl ether [-H] : (52.5 PPM) 329.0669

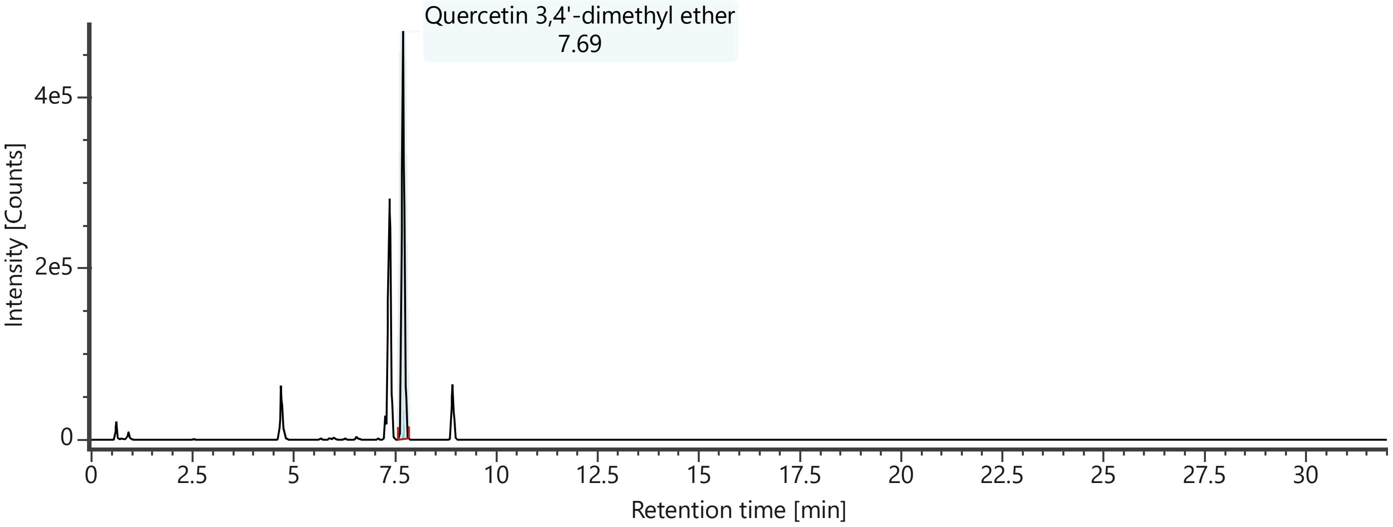

Item name: Sep257-ve
Item description: Mervat253
Channel name: Low energy : Time 7.6957 +/- 0.0222 minutes

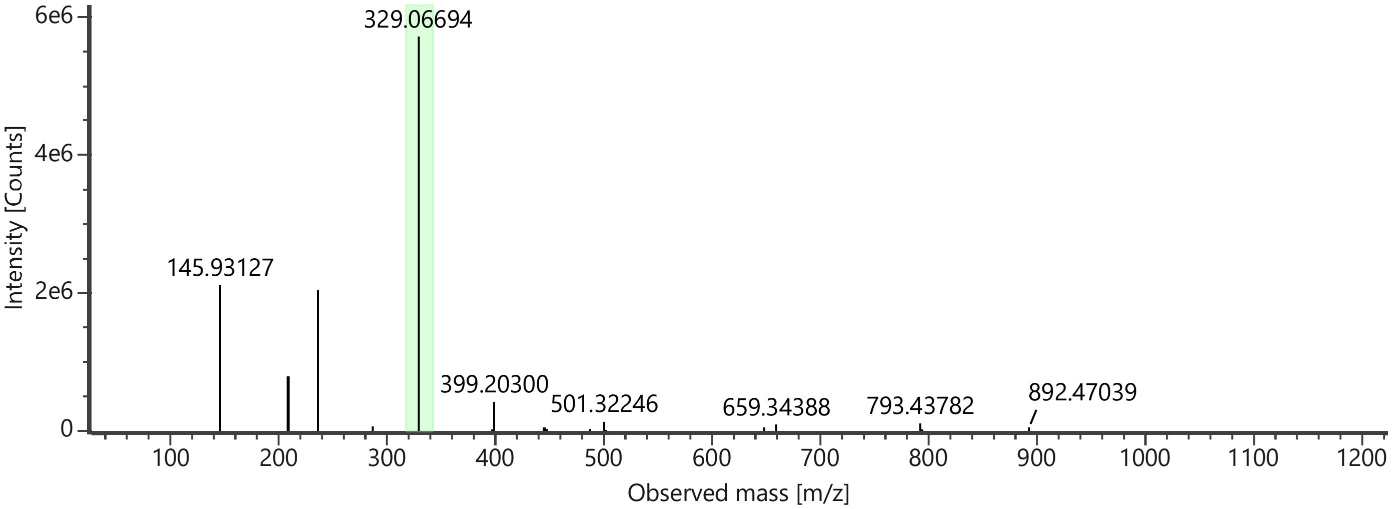

Item name: Sep257-ve
Item description: Mervat253

Channel name: High energy : Time 7.6957 +/- 0.0222 minutes

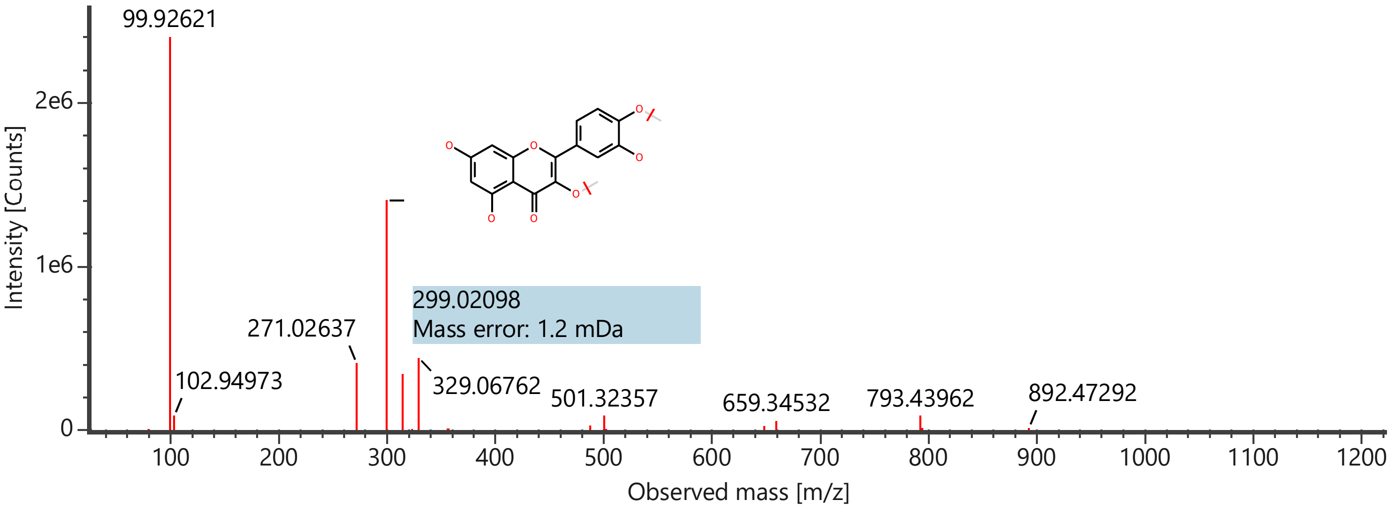

Component name: 3,5-Dihydroxy-4',7-dimethoxyflavone

Item name: Sep257-ve
Channel name: 3,5-Dihydroxy-4',7-dimethoxyflavone [-H] : (52.5 PPM) 313.0718

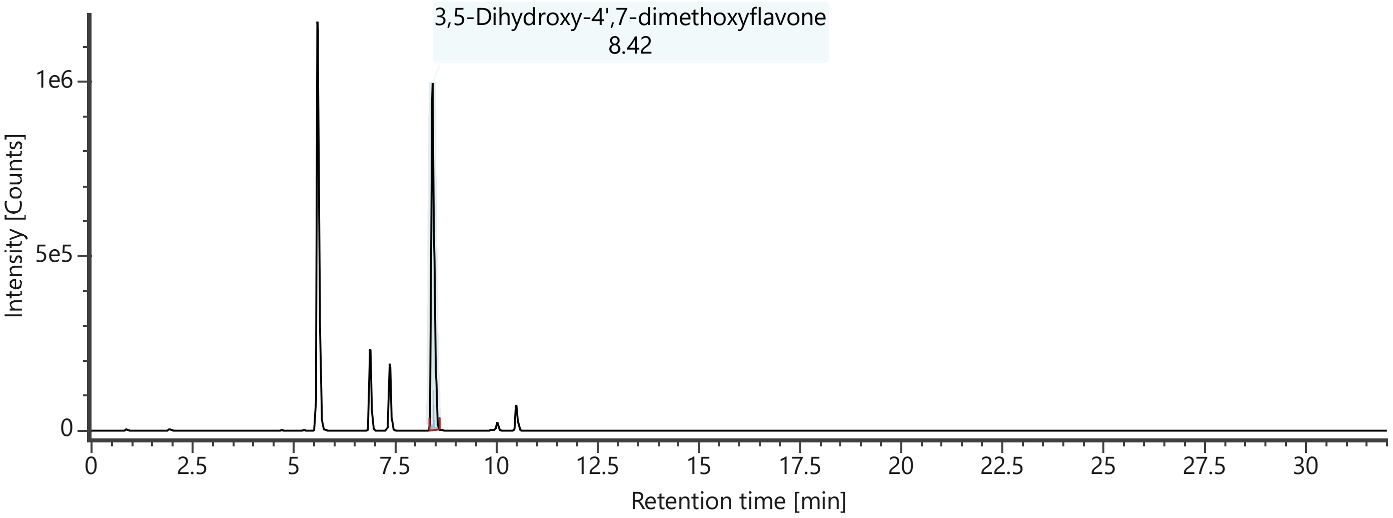

Item name: Sep257-ve
Item description: Mervat253
Channel name: Low energy : Time 8.4263 +/- 0.0222 minutes

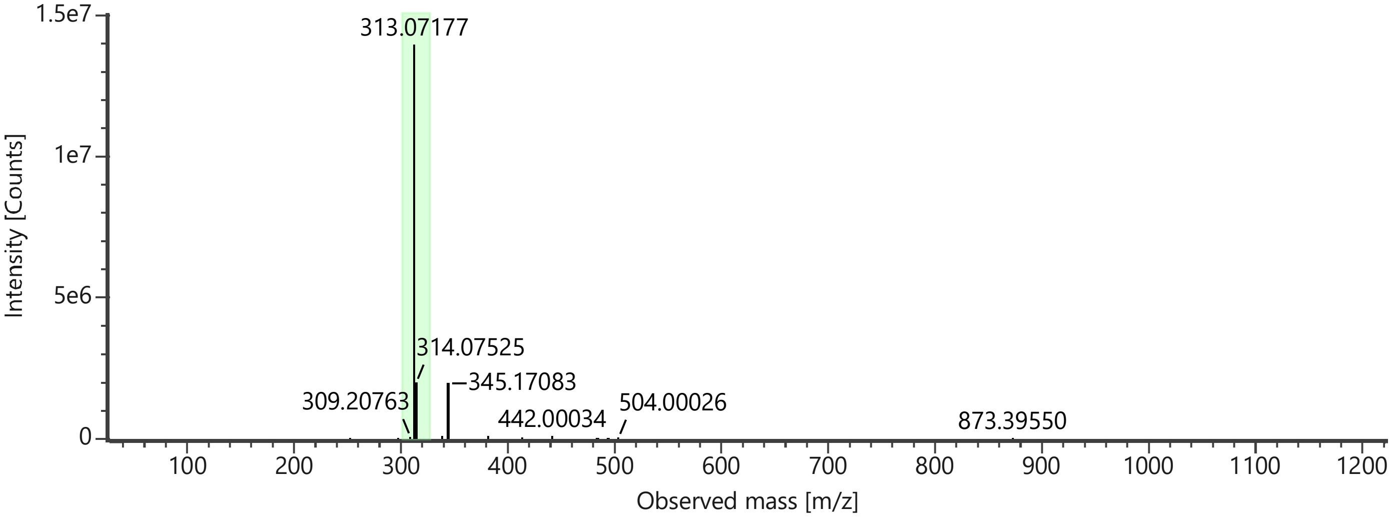

Item name: Sep257-ve
Item description: Mervat253

Channel name: High energy : Time 8.4263 +/- 0.0222 minutes

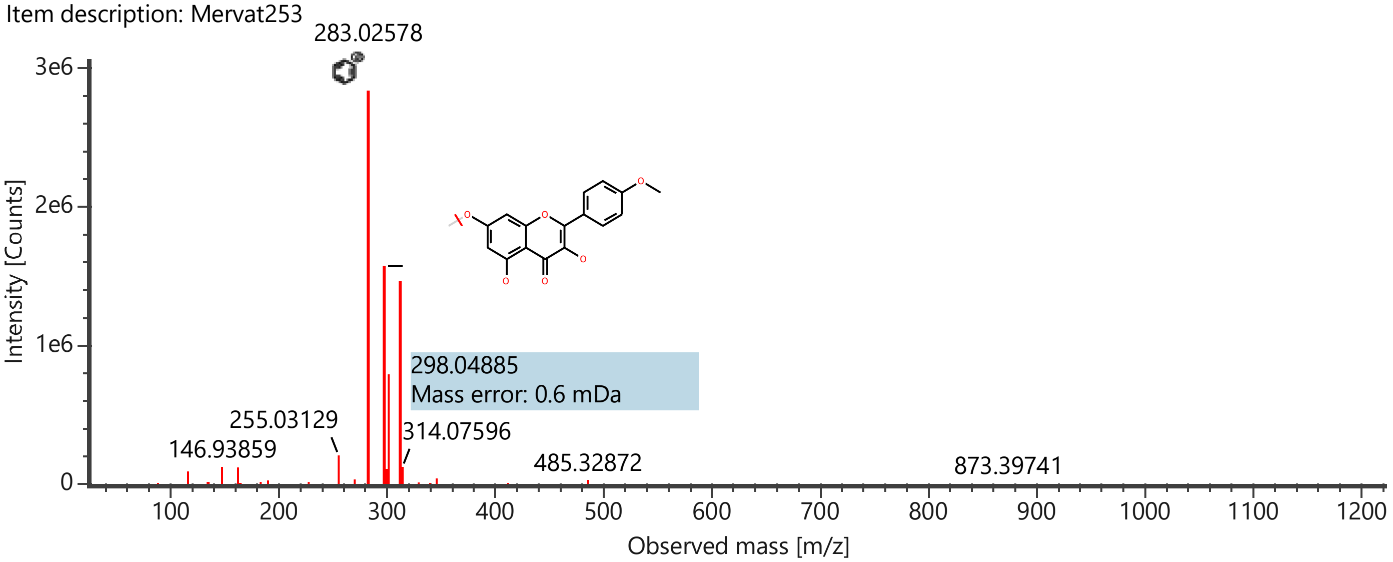

Component name: 3-Epioleanolic acid

Item name: Sep257-ve
Channel name: 3-Epioleanolic acid [-H] : (52.5 PPM) 455.3532

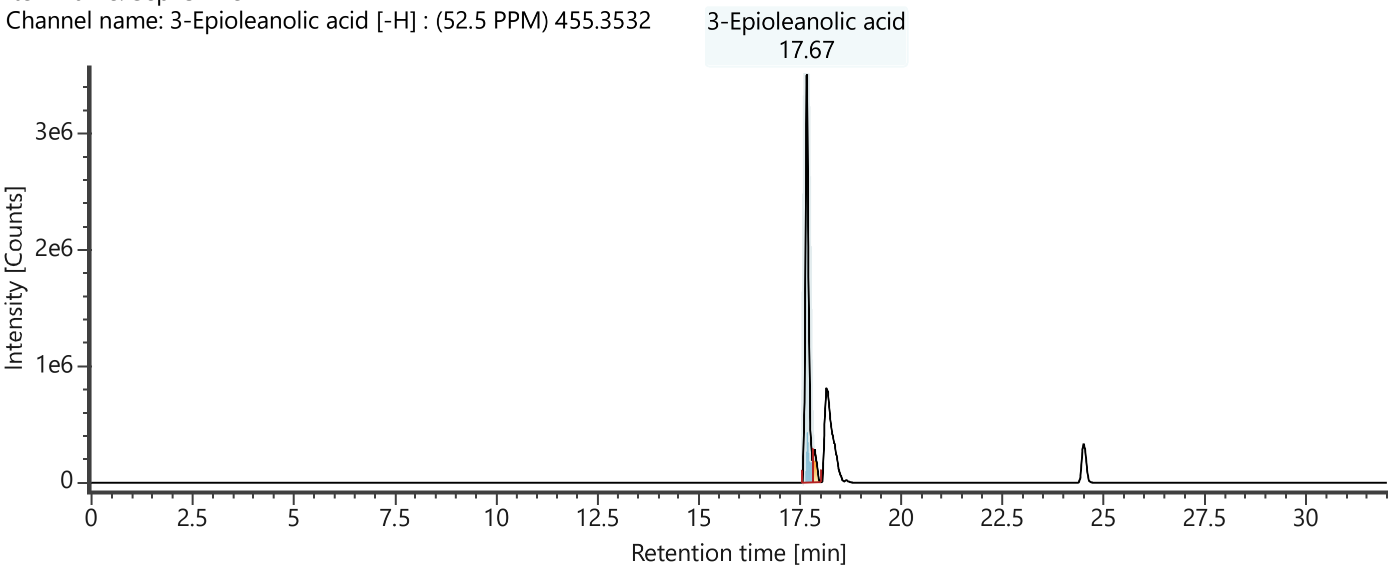

Item name: Sep257-ve
Item description: Mervat253
Channel name: Low energy : Time 17.6661 +/- 0.0222 minutes

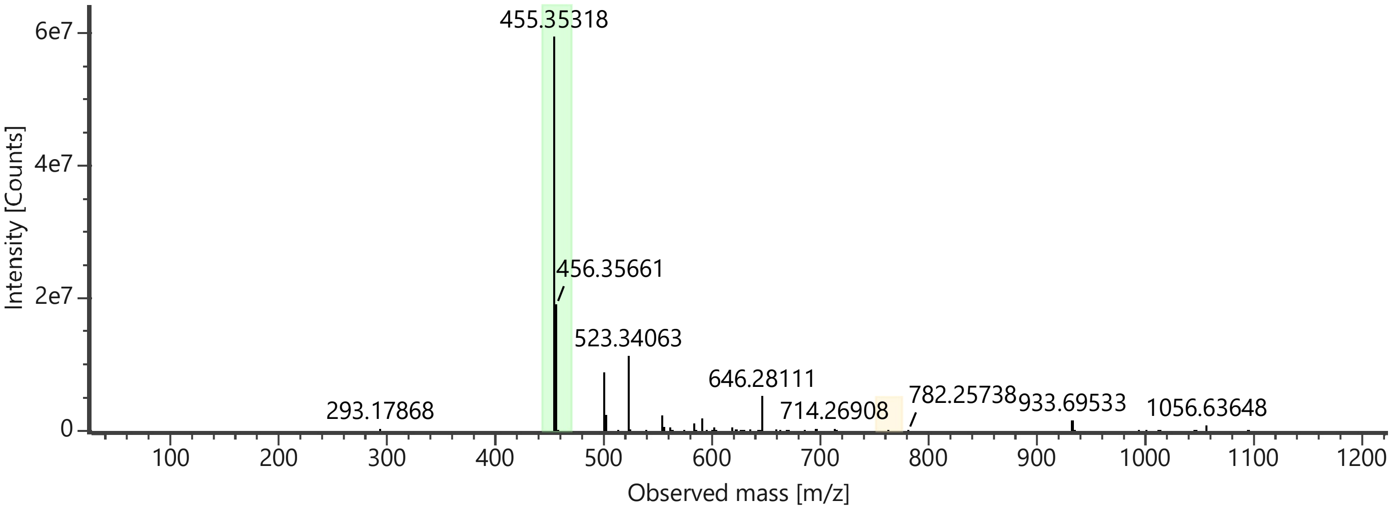

Item name: Sep257-ve
Item description: Mervat253

Channel name: High energy : Time 17.6661 +/- 0.0222 minutes

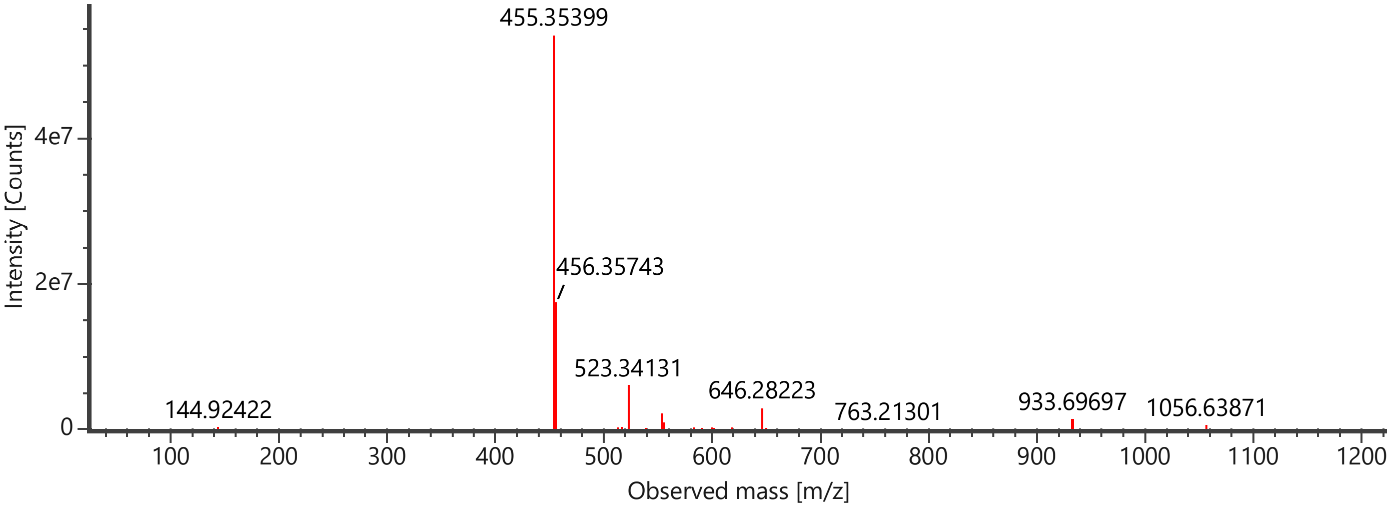

Component name: 3-Epioleanolic acid

Item name: Sep257-ve
Channel name: 3-Epioleanolic acid [-H] : (52.5 PPM) 455.3534

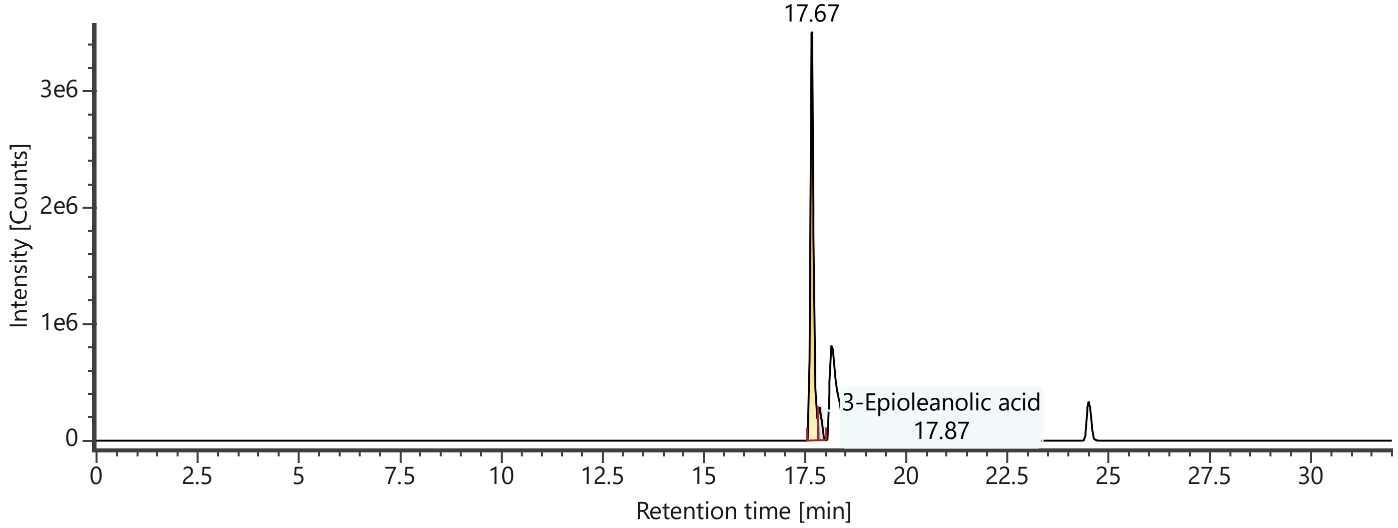

Item name: Sep257-ve
Item description: Mervat253
Channel name: Low energy : Time 17.8732 +/- 0.0222 minutes

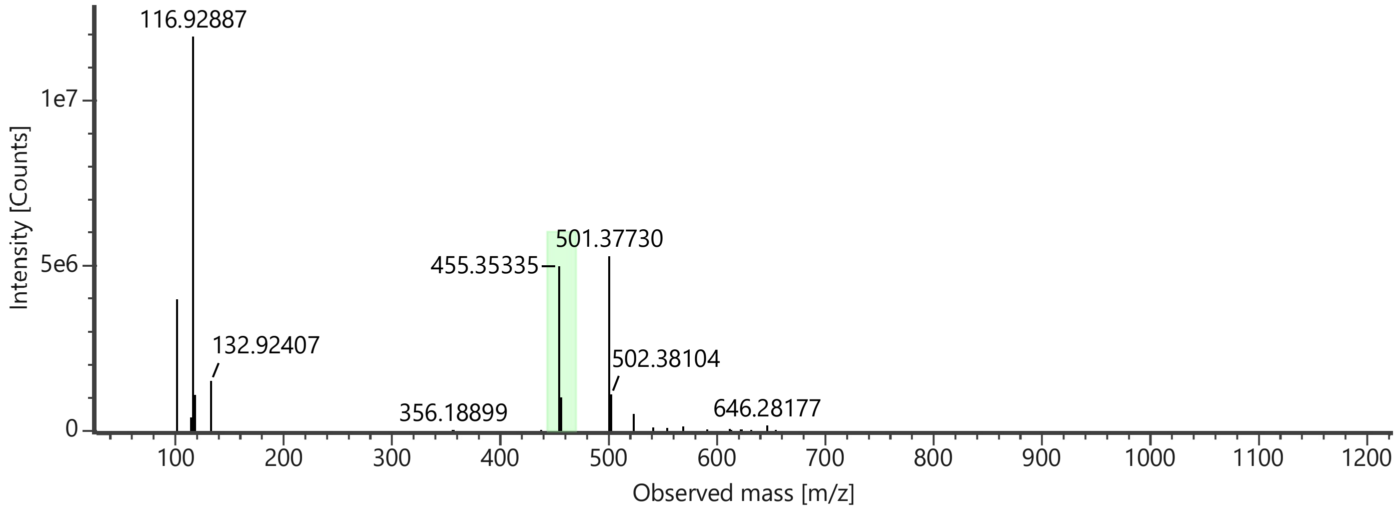

Created by: Ayad, Younan

Created on: Sep 18, 2025

Item name: Lamiaceae family -ve mode

Created time: 13:05:43 Egypt Standard Time

Item name: Sep257-ve
Item description: Mervat253

Channel name: High energy : Time 17.8732 +/- 0.0222 minutes

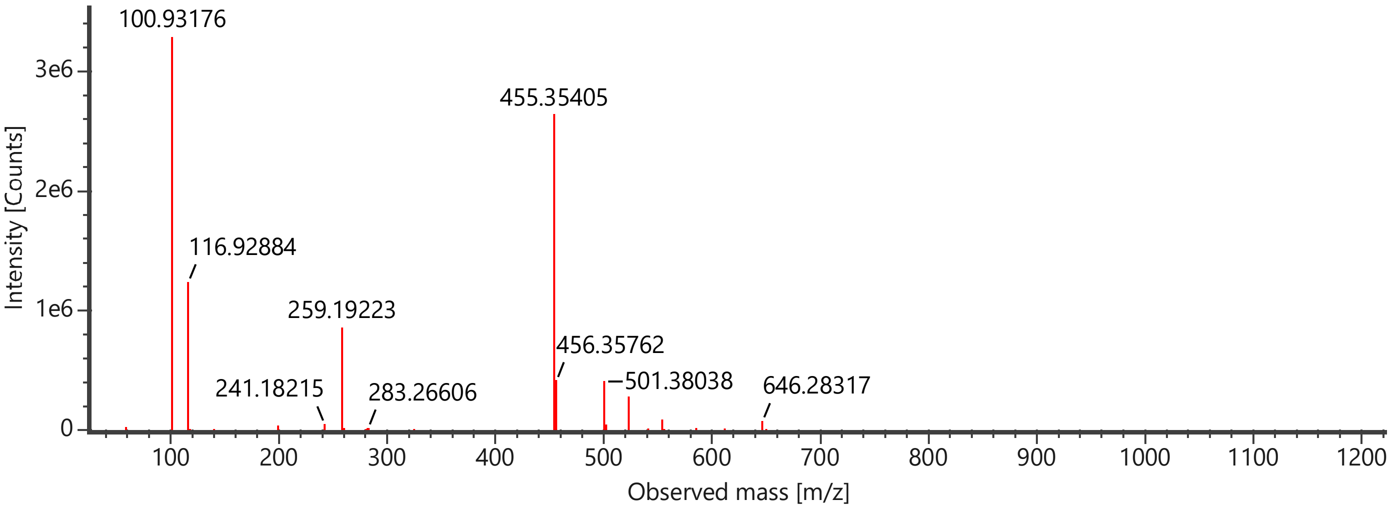

Component name: 3-Epioleanolic acid

Item name: Sep257-ve
Channel name: 3-Epioleanolic acid [-H] : (52.5 PPM) 455.3535

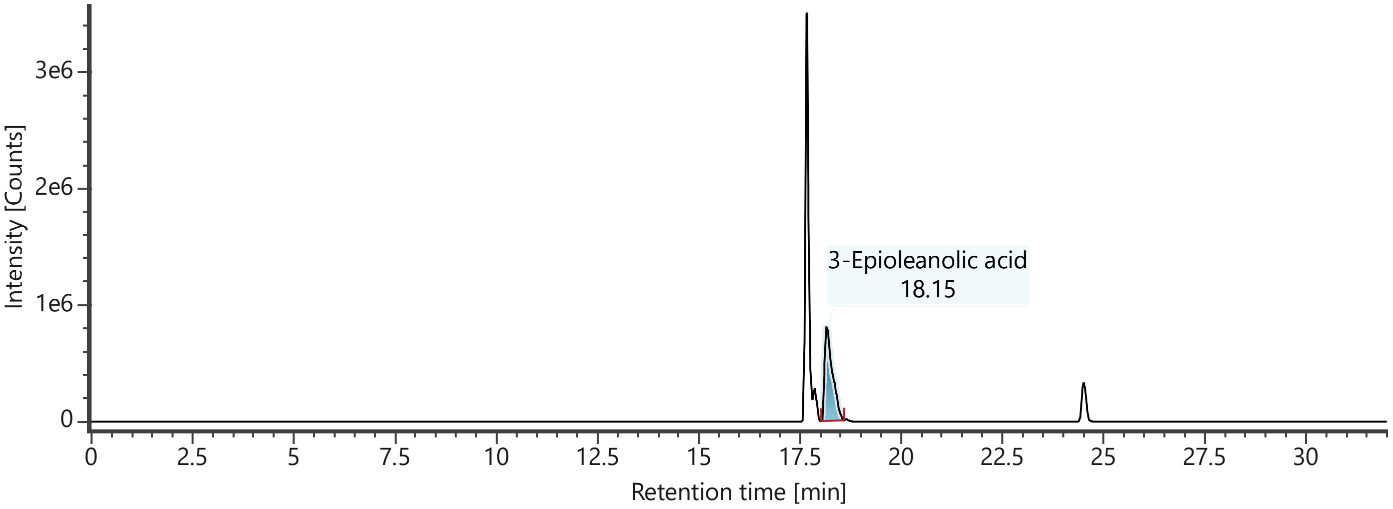

Item name: Sep257-ve
Item description: Mervat253

Channel name: Low energy : Time 18.1414 +/- 0.0222 minutes

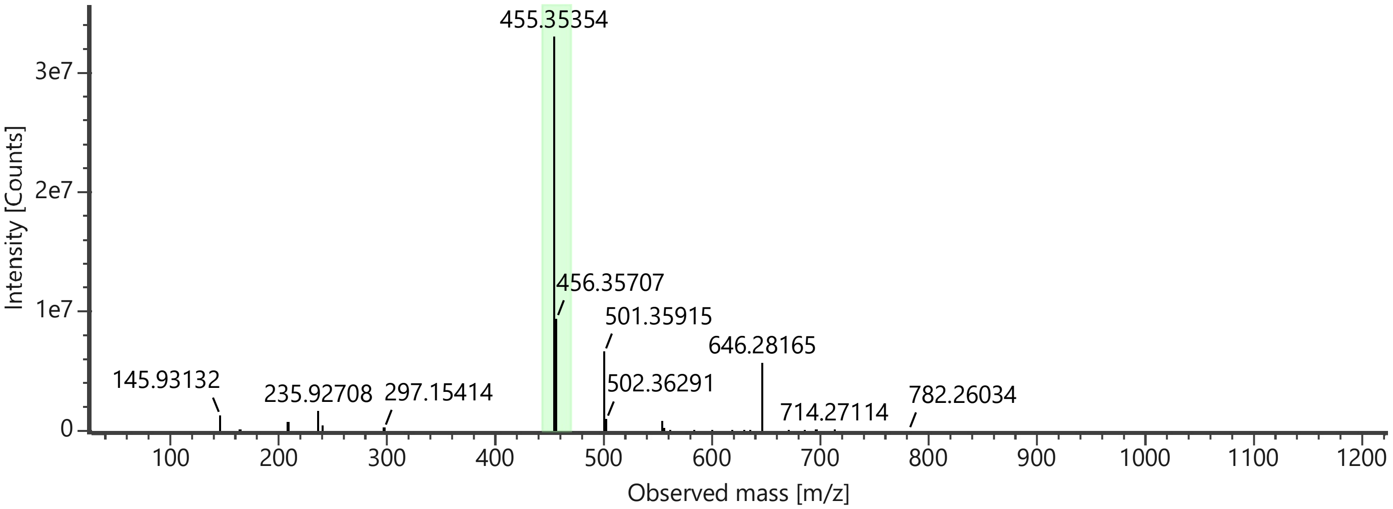

Item name: Sep257-ve
Item description: Mervat253

Channel name: High energy : Time 18.1414 +/- 0.0222 minutes

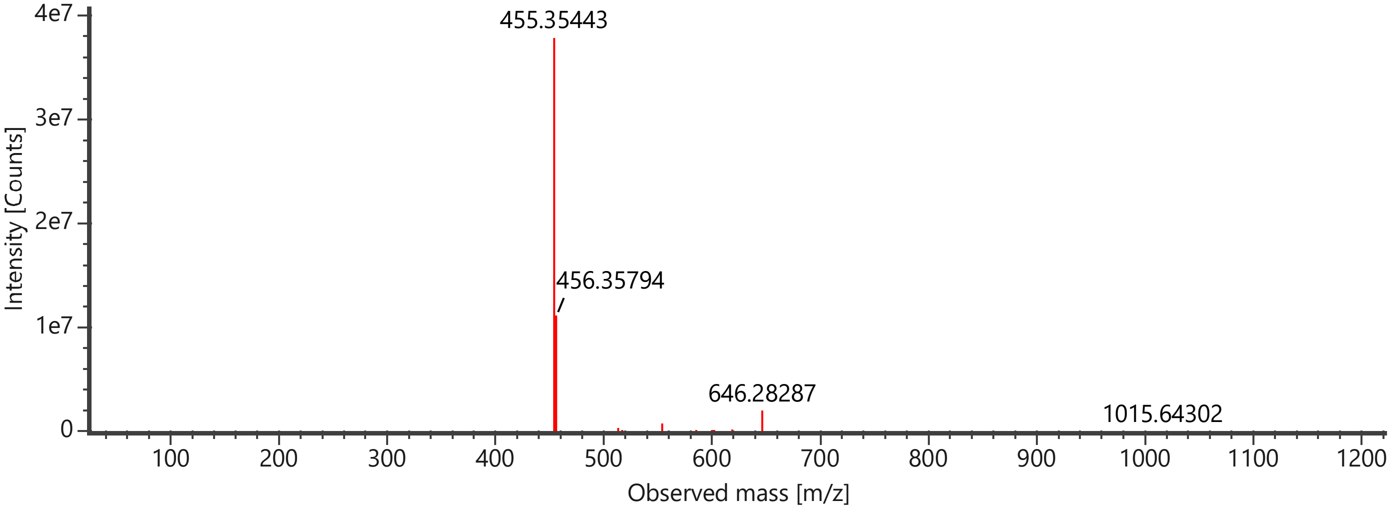

Component name: 3-Epioleanolic acid

Item name: Sep257-ve
Channel name: 3-Epioleanolic acid [-H] : (52.5 PPM) 455.3514

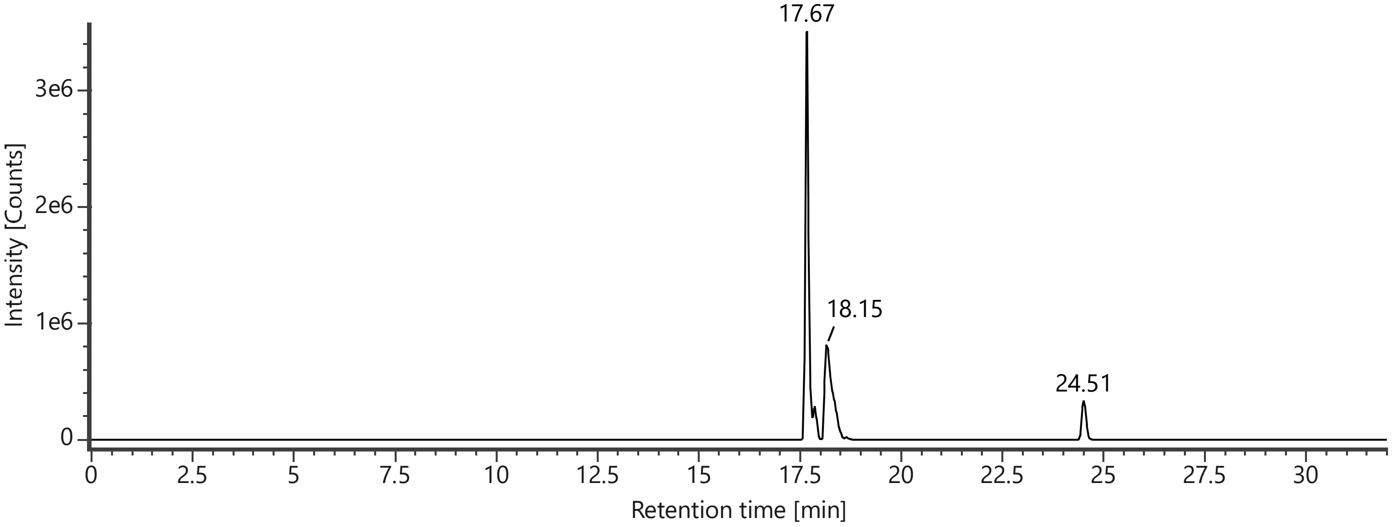

Item name: Sep257-ve
Item description: Mervat253
Channel name: Low energy : Time 24.5077 +/- 0.0222 minutes

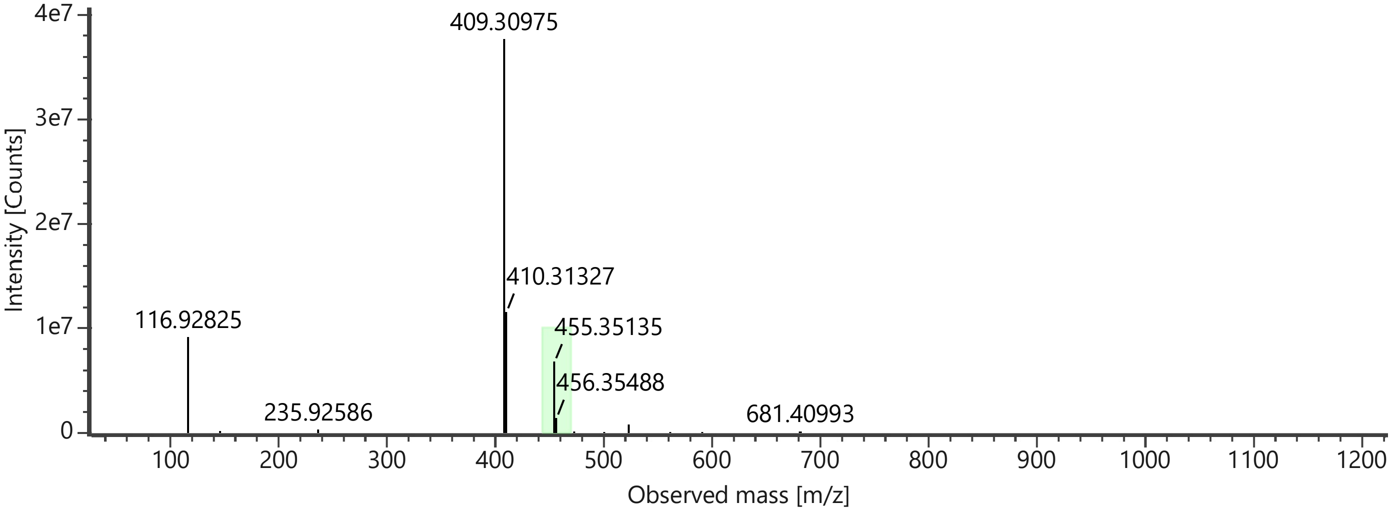

Item name: Sep257-ve
Item description: Mervat253

Channel name: High energy : Time 24.5077 +/- 0.0222 minutes

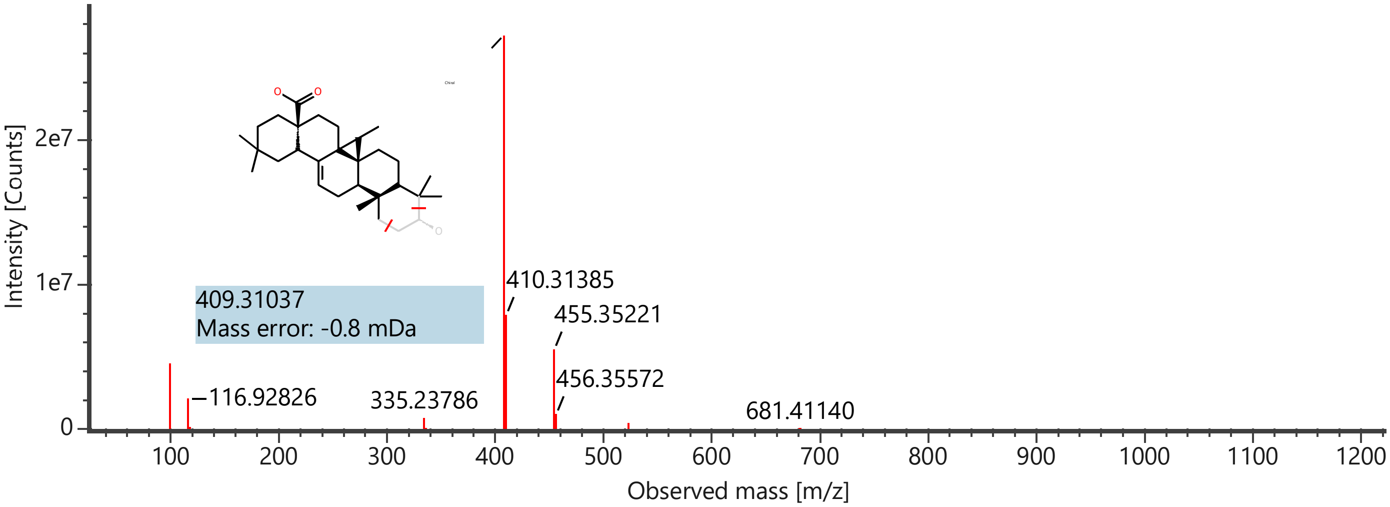

Acquisition was carried out on XEVO G3 QTof instrument , Waters Corporaton ,Milford,MA01757 U.S.A, Mass Spectrometer.

Library Name

Waters Traditional Medicine Library [Jun23]

Experimental Record : Sep257-ve

Quaternary Solvent Manager

Promoted Parameters

Gradient Table

General

Solvent Name A: Waters+0.1%FA

Solvent Name B: ACN+0.1%FA

Solvent Name C:

Solvent Name D:

Comment:

Low Pressure Limit: 0 psi

High Pressure Limit: 15000 psi

Gradient Table

Time (min)	Flow Rate (mL/min)	Composition A (%)	Composition B (%)	Composition C (%)	Composition D (%)	Curve
0.00	0.400	90.0	10.0	0.0	0.0	Initial
2.00	0.400	90.0	10.0	0.0	0.0	6
5.00	0.400	70.0	30.0	0.0	0.0	6
15.00	0.400	30.0	70.0	0.0	0.0	6
22.00	0.400	10.0	90.0	0.0	0.0	6
25.00	0.400	10.0	90.0	0.0	0.0	6
26.00	0.400	0.0	100.0	0.0	0.0	6
29.00	0.400	0.0	100.0	0.0	0.0	6
32.00	0.400	90.0	10.0	0.0	0.0	1

Data

System Pressure (psi) Channel Enable: Yes

Flow Rate (mL/min) Channel Enable: No

A Composition (%) Channel Enable: No

B Composition (%) Channel Enable: No

C Composition (%) Channel Enable: No

D Composition (%) Channel Enable: No

Primary Pressure (psi) Channel Enable: No

Accumulator Pressure (psi) Channel Enable: No

Degasser Pressure (psi) Channel Enable: No

Miscellaneous

Seal Wash Period: 5.00 min

Ramp rate accelerate to 2 mL/min in: 0.45 min

Calculated ramp rate: 4.444 mL/min2

Gradient start: At injection

Sample Manager FTN

General

Wash Solvent: Water

Wash Solvent Pre Inject: 0 s

Wash Solvent Post Inject: 6 s

Purge Solvent: Water

Load Ahead: Disabled

Loop Offline: Disabled

Column Temperature Enable: No

Sample Temperature Enable: No

Active Preheater: Disabled

Comment:

Data

Sample Temperature (°C) Channel Enabled: Yes

Column Temperature (°C) Channel Enabled: No

Ambient Temperature (°C) Channel Enabled: Yes

Sample Pressure (psi) Channel Enabled: No

Seal Force (%) Channel Enabled: No

Pre Heater Temperature (°C) Channel Enabled: No

Dilution

Dilution Enable: Disabled

Needle Placement (from bottom): Disabled

Dilution Dispense Purge Solvent: Disabled

Post Dilution Delay: Disabled

Events

Run Events: No

Advanced

Syringe Draw Rate: Automatic

Needle Placement (from bottom): Automatic

Pre Aspirate Air: Automatic
Post Aspirate Air: Automatic
Mix Stroke Cycles: Automatic
Mix Stroke Volume: Automatic

Xevo G3 QTof

Method

Polarity: Negative
Analyzer mode: Sensitivity

MS^E

Start time: 0.00 min
End time: 32.00 min
Low mass: 50 m/z
High mass: 1200 m/z
Scan time: 0.150 s
Low collision energy: 6 V
High collision energy ramp start: 15 V
High collision energy ramp end: 40 V
Intelligent Data Capture: On
Intelligent Data Capture threshold: Medium (10)

Source parameters

Source type: ESI
Source temperature: 120 °C
Desolvation temperature: 550 °C
Cone gas: 50 L/h
Desolvation gas: 1000 L/h
Capillary voltage: 1.00 kV
Sample cone voltage: 40 V

Lock correction

Mode: Automatic
Automatic sampling interval: Yes

Options

Acquisition check failure: Continue with lock correction
Automatic detector check: Off

Events

Time (min)	Event	Parameters
Initial	Flow state	LC, Sample

Method trigger

Trigger type: Network

Post Run Report

Quaternary Solvent Manager

Software version: 3.3.1
Firmware version: 1.72.415 (Aug 7 2018)
Checksum: 0x93efe762
Serial number: D23QSP719A
Minimum system pressure: 4834 psi
Maximum system pressure: 11860 psi
Mean system pressure: 8673 psi
Messages:

Sample Manager FTN

Software version: 3.3.1

Firmware Version: 1.71.395 (Feb 14 2018)

Checksum: 0x285858d7

Serial Number: L22FTP271G

Sample Syringe Size: 100 µL

Extended Loop Size: 0 µL

Needle Size: 15 µL

Column Type: ACQUITY UPLC® BEH C18 1.7µm

Column Serial Number: 02483432115760

Column Injections: 607

Min Sample Temperature: 22.3 °C

Max Sample Temperature: 22.6 °C

Mean Sample Temperature: 22.5 °C

Min Column Temperature: 22.9 °C

Max Column Temperature: 23.0 °C

Mean Column Temperature: 23.0 °C

Auto Defrost Enabled: False

Messages:

Xevo G3 QTof

Serial number: YGA0187

Instrument driver version: 1.2.0

Dynamic parameters

Scan time for function Reference: 0.050 s

Scan time for function MSe Low Collision: 0.150 s

Scan time for function MSe High Collision: 0.150 s

Calibration

Calibrated

Modes

ADC mode: Signum

Enhance mode: None

MSMS mode: MS

Analyser mode: Sensitivity

Polarity: Negative

Quad mode: 3940

Source mode: ESI

TOF mode: TOF

Quadrupole options: Automatic profile

ESI LockSpray

Capillary voltage: 1.00 kV

Reference capillary voltage: 3.00 kV

Sampling cone voltage: 40 V

Source temperature: 120 °C

Desolvation temperature: 550 °C

Cone gas flow rate: 50 L/h

Desolvation gas flow rate: 1000 L/h

Sample Fluidics

Reservoir: Wash

Flow path: Infusion

Infusion flow rate: 100.0 µl/min

Fill volume: 250 µL

Wash cycle: 2

Reference Fluidics

Reservoir: B

Flow path: Infusion

Infusion flow rate: 20.0 µl/min

Baffle position: Sample

Illumination: Off

StepWave

Source offset: 30 V

StepWave RF: 150 V

Ion guide RF: 350 V

Body gradient: 10.0 V

Head gradient: 10.0 V

Ion guide 2 offset: 0.3 V

Ion guide 1 offset: 3.0 V

Diff aperture 2: 0.0 V

Wave height: 0.5 V

Wave velocity: 150 m/s

Quadrupole

Low mass resolution: 4.7

High mass resolution: 15.0

Pre-filter: 2.0 V

Ion energy: 0.8 V

DRE

Collector: 60 V

Collector pulse: 10 V

Stopper: 10 V

Stopper pulse: 20 V

pDRE attenuate: Off

pDRE transmission: 1.0 %

Collision

Entrance: 2 V

Gradient: 1.0 V

Static offset: 120 V

Offset B: 0.0 V

Offset C: 0.5 V

Exit: 15 V

Cell RF: 150 V

Cell 2 RF gain: 10 V

Tof

Acceleration 1: 10 V

Acceleration 2: 120 V

Acceleration steering: -0.50 V

Aperture 2: 41 V

Transport 1: 40 V

Transport 2: 37 V

Steering: -0.50 V

Tube lens: 18 V

Entrance: 12 V

Pusher: 1900 V

Pusher offset: 0.00 V

Puller: 1400 V

Puller offset: 0.00 V

Flight tube: 9.00 kV

Reflectron: 1.602 kV

Reflectron grid: 1.7100 kV

ADC

Baseline threshold: -2364

Amplitude threshold: 11

Ion area threshold: 0

T0: -250 ns

Veff: 6340.577 V

Trigger threshold: 1.0

Centroid threshold: -1

Ion area offset: 0

Average single ion intensity: 30.6

Measured m/z: 554.2620

Measured charge: 1

ADC algorithm: ADC

Detector voltage: 2886 V

MS Profile

Quadrupole options: Automatic profile

Set mass: 785.80

Report Log

Template Report

Body Objects

Chapter: Analysis results

Untitled

Report Object [4]: Sample results

Object saved in the Report Template was modified

Group Report Object [5]: Untitled

Untitled

Group Report Object [5]: Untitled

Object saved in the Report Template was modified
