# Supplementary material for: Integrated metabolomic, nanoformulation, and network pharmacology approach reveals multifunctional bioactivities of an Ocimum sanctum nanoemulsion
Source: Front Bioeng Biotechnol. 2026 Mar 20;14:1731720. doi: 10.3389/fbioe.2026.1731720 (PMC13047145; doi:10.3389/fbioe.2026.1731720)

My GC-MS Report

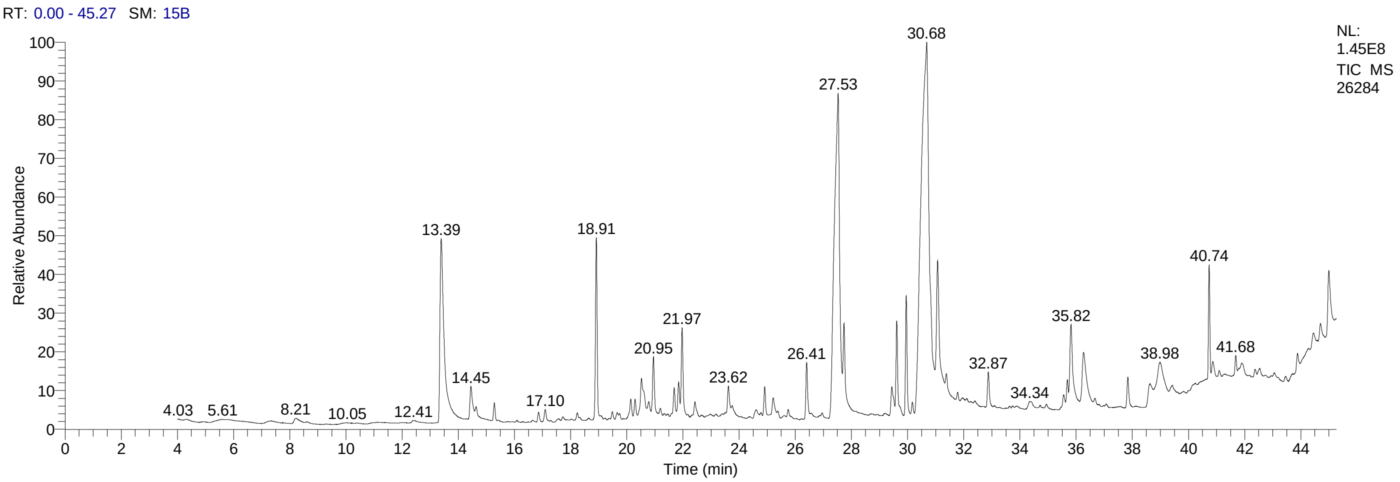

RT	Area %	Peak Area	Peak Height
13.39	6.97	368748573.28	58381409.08
14.45	1.06	56087652.35	11530514.53
15.29	0.50	26414816.98	7654893.05
17.10	0.37	19689478.58	4823086.50
18.92	5.00	264682331.80	73684731.85
20.14	0.56	29631469.59	7371576.46
20.29	0.47	24746688.45	7076091.75
20.52	1.38	72746476.07	12204172.14
20.95	1.61	85351777.29	22806580.07
21.69	0.69	36349764.16	11000570.62
21.84	0.80	42114626.34	12284210.44
21.97	2.44	128847190.66	36243532.45
22.43	0.47	24780779.67	5428609.31
23.62	0.94	49899793.51	11088517.30
24.91	0.81	43023445.58	12451716.43
25.21	0.58	30869380.39	6428469.55
26.40	1.61	84966028.92	23085340.31
27.53	19.33	1022172681.03	97147654.50
27.74	1.58	83633778.81	27292704.62
29.43	0.97	51090460.46	10614375.31
29.62	2.45	129329925.15	38477824.13
29.95	3.19	168469479.51	50428530.46
30.17	0.32	16873938.27	4929730.81
30.69	25.03	1323651720.12	90077438.36
30.84	0.23	12167356.05	5491342.68
31.07	3.80	200725382.26	42124117.53
31.38	0.36	18865535.98	5834383.41
32.87	1.06	56194423.45	14282197.55
35.55	0.32	16745908.70	4254620.61
35.69	0.46	24251605.03	8202813.24
35.81	2.42	128042165.75	25358501.60
36.26	1.98	104672178.36	15078913.52
37.84	0.90	47795839.50	12200557.30
38.61	0.69	36343178.64	5167699.36
38.97	1.01	53354808.67	6489497.99
40.74	2.71	143080495.48	53473206.81
40.87	0.65	34280460.50	6014783.19
41.68	0.44	23526622.90	7846414.02
43.87	0.51	27223686.75	6461753.94
44.45	0.52	27394607.09	4667247.92
44.70	0.70	36927106.11	6497287.37
44.99	2.13	112868055.91	22253076.81

My GC-MS Report

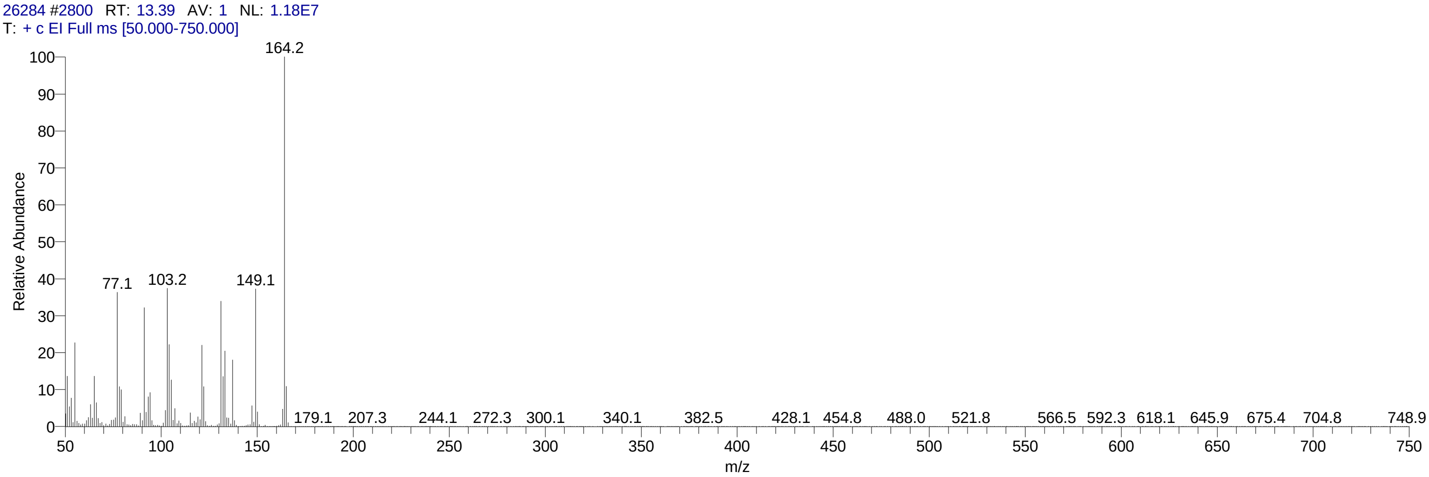

RT	Compound Name	Area %	MF	Molecular Formula	Molecular Weight	Cas #	Library
13.39	Eugenol	6.97	942	C10H12O2	164	97-53-0	replib
13.39	Eugenol	6.97	937	C10H12O2	164	97-53-0	replib
13.39	Phenol, 2-methoxy-3-(2-propenyl)-	6.97	936	C10H12O2	164	1941-12-4	mainlib
13.39	PHENOL, 2-METHOXY-3-(2-PROPENYL)-	6.97	936	C10H12O2	164	1941-12-4	WileyRegistry8e
13.39	PHENOL, 2-METHOXY-4-(2-PROPENYL)-	6.97	937	C10H12O2	164	97-53-0	WileyRegistry8e

Compound Structure | Hit Spectrum

Eugenol
Formula C10H12O2, MW 164, CAS# 97-53-0, Entry# 25332
Phenol, 2-methoxy-4-(2-propenyl)-

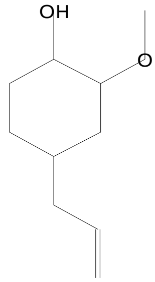

SI 939, RSI 942, replib, Entry# 25332, CAS# 97-53-0, Eugenol

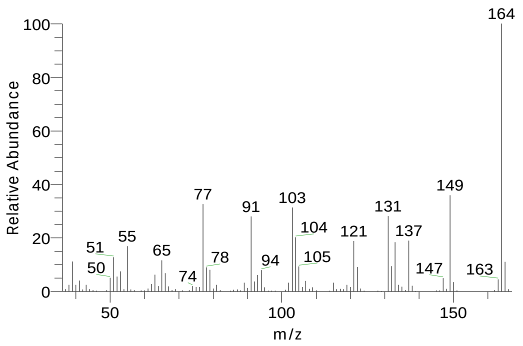

Eugenol
Formula C10H12O2, MW 164, CAS# 97-53-0, Entry# 25335
Phenol, 2-methoxy-4-(2-propenyl)-

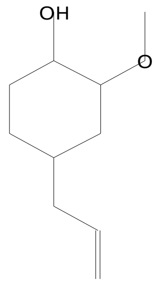

SI 936, RSI 937, replib, Entry# 25335, CAS# 97-53-0, Eugenol

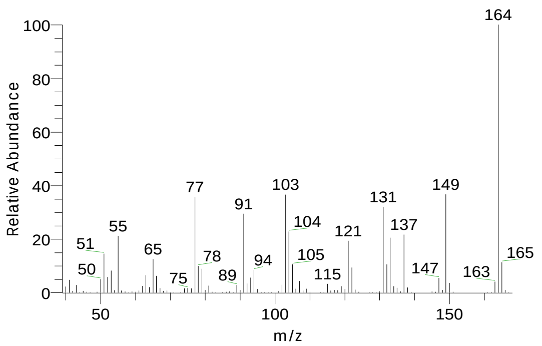

My GC-MS Report

Compound Structure	Hit Spectrum

Phenol, 2-methoxy-3-(2-propenyl)-
Formula C10H12O2, MW 164, CAS# 1941-12-4, Entry# 153401
3-Allyl-2-methoxyphenol #

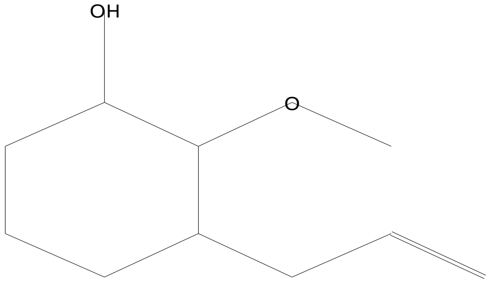

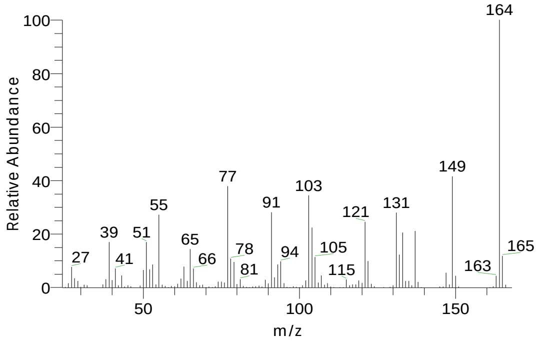

PHENOL, 2-METHOXY-3-(2-PROPENYL)-
Formula C10H12O2, MW 164, CAS# 1941-12-4, Entry# 390451
3-ALLYL-2-METHOXYPHENOL #

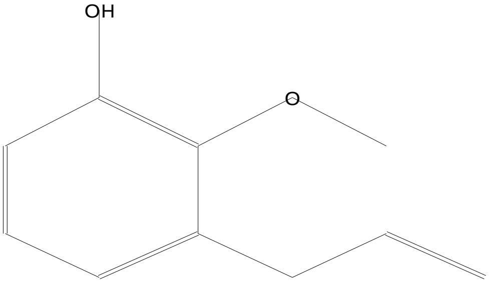

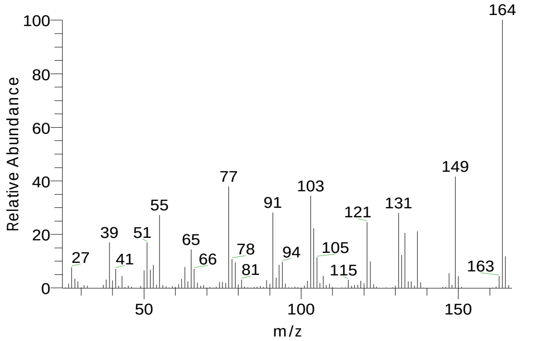

PHENOL, 2-METHOXY-4-(2-PROPENYL)-
Formula C10H12O2, MW 164, CAS# 97-53-0, Entry# 46893
4-ALLYL-2-METHOXY-PHENOL

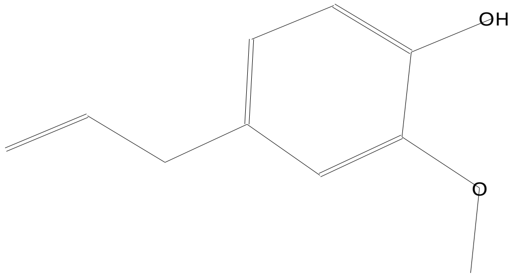

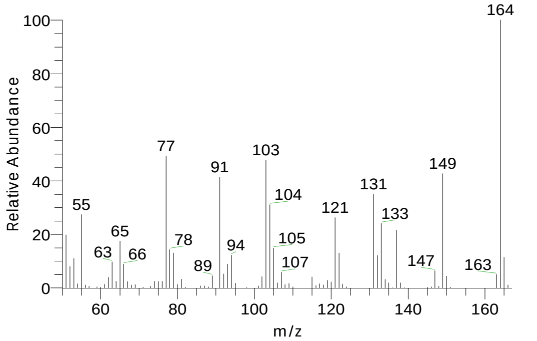

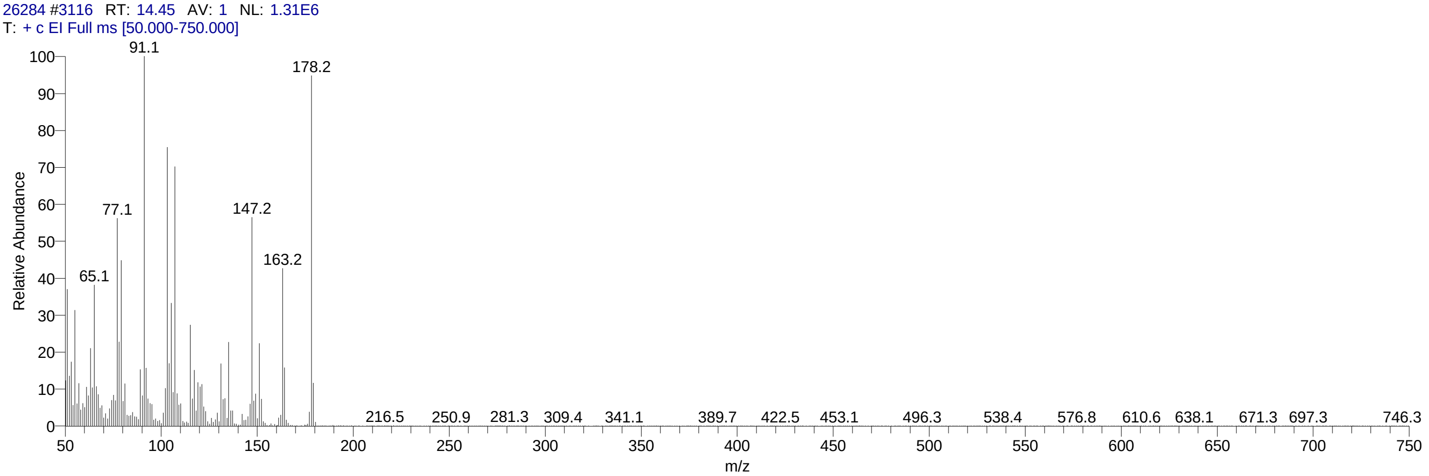

RT	Compound Name	Area %	MF	Molecular Formula	Molecular Weight	Cas #	Library
14.45	Methyleugenol	1.06	917	C11H14O2	178	93-15-2	replib
14.45	BENZENE, 1,2-DIMETHOXY-4-(2-PROPENYL)-	1.06	878	C11H14O2	178	93-15-2	WileyRegistry8e

My GC-MS Report

RT	Compound Name	Area %	MF	Molecular Formula	Molecular Weight	Cas #	Library
14.45	BENZENE, 1,2-DIMETHOXY-4-(2-PROPENYL)-	1.06	902	C11H14O2	178	93-15-2	WileyRegistry8e
14.45	Methyleugenol	1.06	871	C11H14O2	178	93-15-2	replib
14.45	BENZENE, 1,2-DIMETHOXY-4-(2-PROPENYL)-	1.06	877	C11H14O2	178	93-15-2	WileyRegistry8e

Compound Structure | Hit Spectrum

Methyleugenol
Formula C11H14O2, MW 178, CAS# 93-15-2, Entry# 26658
Benzene, 1,2-dimethoxy-4-(2-propenyl)-

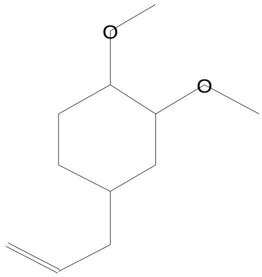

SI 868, RSI 917, replib, Entry# 26658, CAS# 93-15-2, Methyleugenol

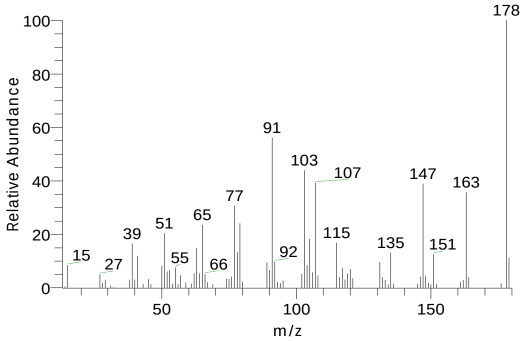

BENZENE, 1,2-DIMETHOXY-4-(2-PROPENYL)-
Formula C11H14O2, MW 178, CAS# 93-15-2, Entry# 60772
1, 2-DIMETHOXY-4-(2-PROPENYL)BENZENE

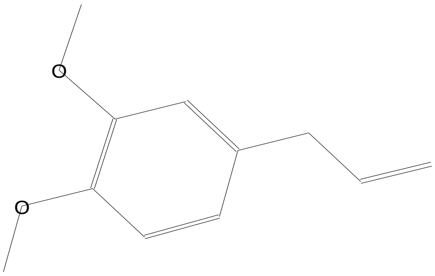

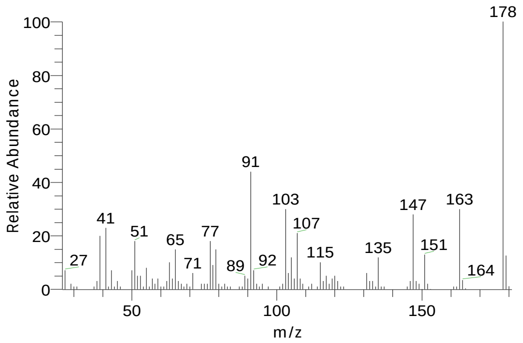

BENZENE, 1,2-DIMETHOXY-4-(2-PROPENYL)-
Formula C11H14O2, MW 178, CAS# 93-15-2, Entry# 60776
1, 2-DIMETHOXY-4-(2-PROPENYL)BENZENE

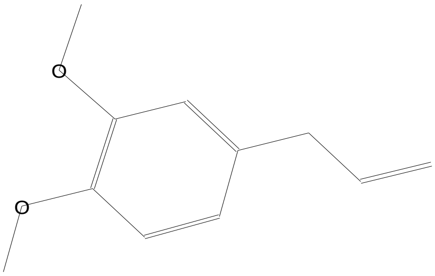

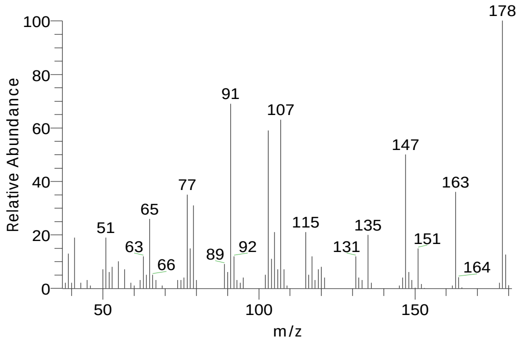

Methyleugenol
Formula C11H14O2, MW 178, CAS# 93-15-2, Entry# 26659
Benzene, 1,2-dimethoxy-4-(2-propenyl)-

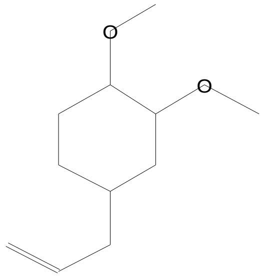

SI 853, RSI 871, replib, Entry# 26659, CAS# 93-15-2, Methyleugenol

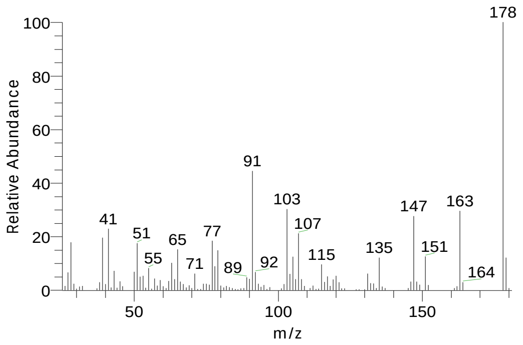

My GC-MS Report

Compound Structure | Hit Spectrum

BENZENE, 1,2-DIMETHOXY-4-(2-PROPENYL)-
Formula C11H14O2, MW 178, CAS# 93-15-2, Entry# 388723
1, 2-DIMETHOXY-4-(2-PROPENYL)BENZENE

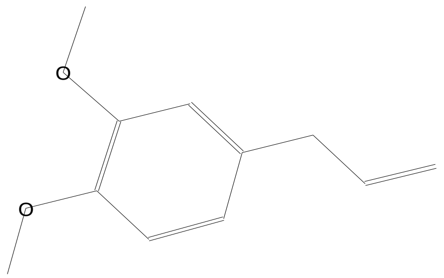

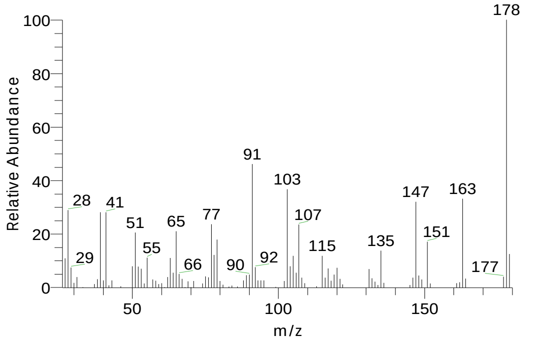

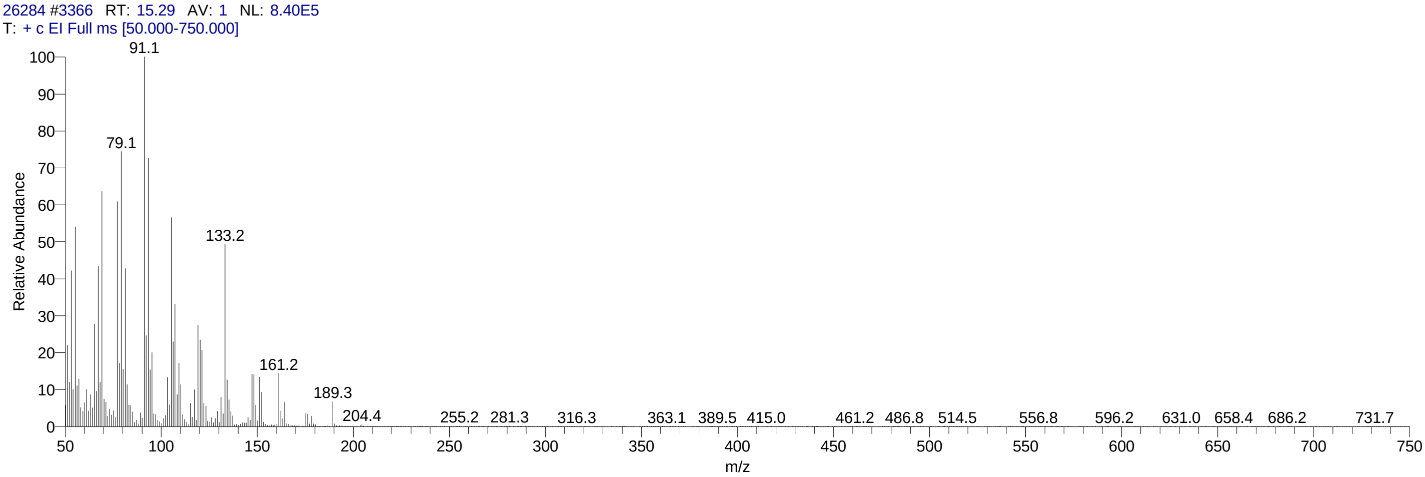

RT	Compound Name	Area %	MF	Molecular Formula	Molecular Weight	Cas #	Library
15.29	Caryophyllene	0.50	882	C15H24	204	87-44-5	replib
15.29	BICYCLO[7.2.0]UNDEC-4-ENE, 4,11,11-TRIMETHYL-8-METHYLENE-, [1R-(1R*,4E,9S*)]-	0.50	907	C15H24	204	87-44-5	WileyRegistry8e
15.29	BICYCLO[7.2.0]UNDEC-4-ENE, 4,11,11-TRIMETHYL-8-METHYLENE-, [1R-(1R*,4E,9S*)]-	0.50	931	C15H24	204	87-44-5	WileyRegistry8e
15.29	BICYCLO[7.2.0]UNDEC-4-ENE, 4,11,11-TRIMETHYL-8-METHYLENE-, [1R-(1R*,4E,9S*)]-	0.50	883	C15H24	204	87-44-5	WileyRegistry8e
15.29	BICYCLO[7.2.0]UNDEC-4-ENE, 4,11,11-TRIMETHYL-8-METHYLENE-, [1R-(1R*,4E,9S*)]-	0.50	890	C15H24	204	87-44-5	WileyRegistry8e

Compound Structure | Hit Spectrum

Caryophyllene
Formula C15H24, MW 204, CAS# 87-44-5, Entry# 1246
Bicyclo[7.2.0]undec-4-ene, 4,11,11-trimethyl-8-methylene-, [1R-(1R*,4E,9S*)]-

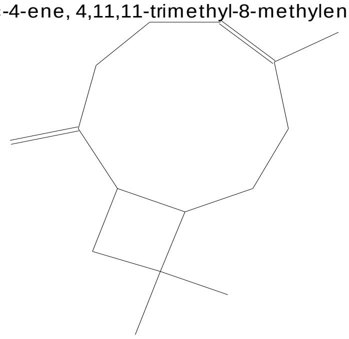

SI 848, RSI 882, replib, Entry# 1246, CAS# 87-44-5, Caryophyllene

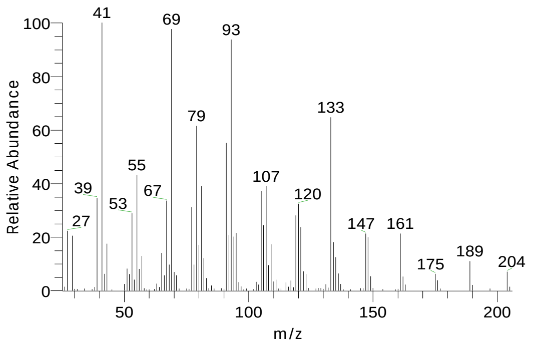

My GC-MS Report

Compound Structure | Hit Spectrum

BICYCLO[7.2.0]UNDEC-4-ENE, 4,11,11-TRIMETHYL-8-METHYLENE-, [1R-(1R*,4E,9S*)]-
Formula C15H24, MW 204, CAS# 87-44-5, Entry# 89170
2,6,10,10-TETRAMETHYLBICYCLO[7.2.0]UNDECA-1,6-DIENE #

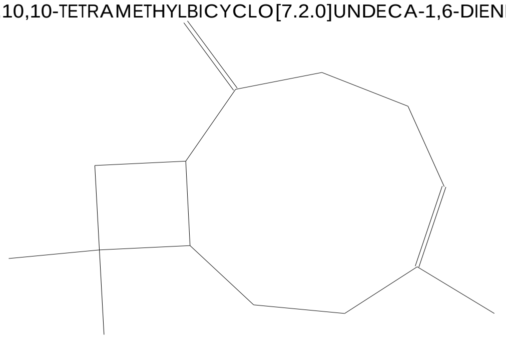

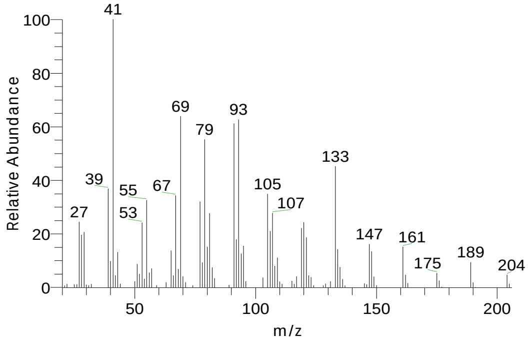

BICYCLO[7.2.0]UNDEC-4-ENE, 4,11,11-TRIMETHYL-8-METHYLENE-, [1R-(1R*,4E,9S*)]-
Formula C15H24, MW 204, CAS# 87-44-5, Entry# 89167
2,6,10,10-TETRAMETHYLBICYCLO[7.2.0]UNDECA-1,6-DIENE #

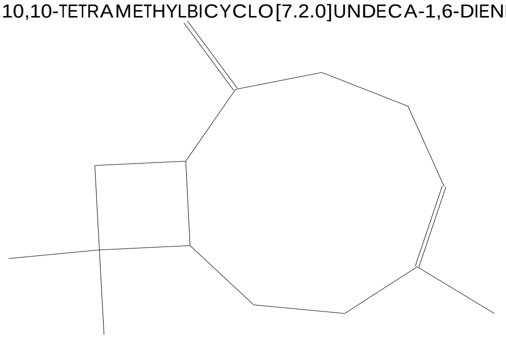

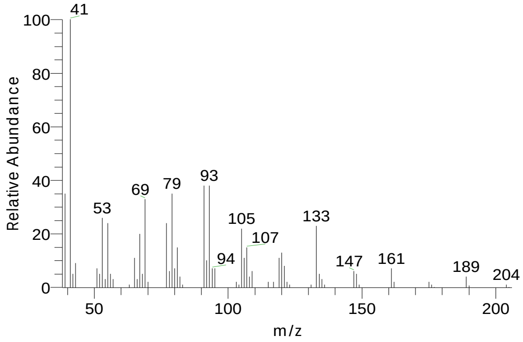

BICYCLO[7.2.0]UNDEC-4-ENE, 4,11,11-TRIMETHYL-8-METHYLENE-, [1R-(1R*,4E,9S*)]-
Formula C15H24, MW 204, CAS# 87-44-5, Entry# 89161
2,6,10,10-TETRAMETHYLBICYCLO[7.2.0]UNDECA-1,6-DIENE #

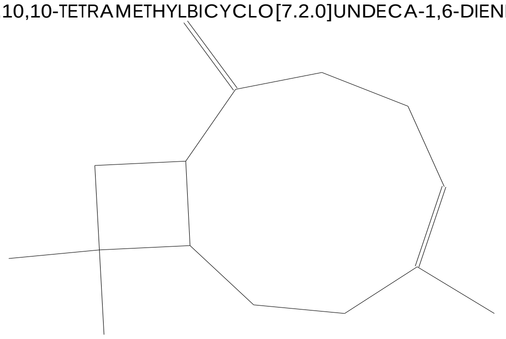

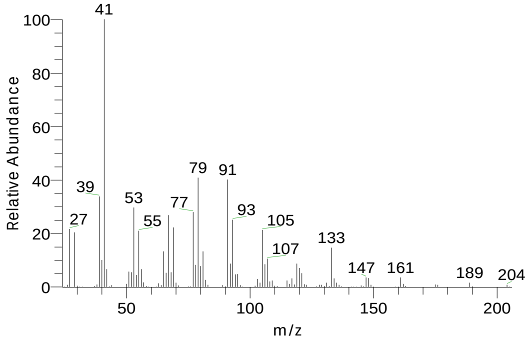

BICYCLO[7.2.0]UNDEC-4-ENE, 4,11,11-TRIMETHYL-8-METHYLENE-, [1R-(1R*,4E,9S*)]-
Formula C15H24, MW 204, CAS# 87-44-5, Entry# 89168
2,6,10,10-TETRAMETHYLBICYCLO[7.2.0]UNDECA-1,6-DIENE #

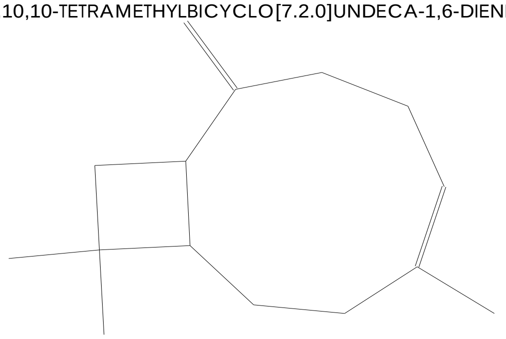

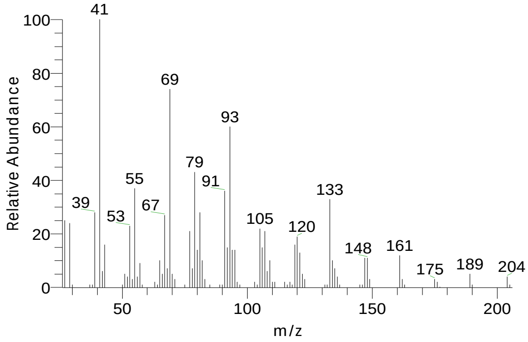

My GC-MS Report

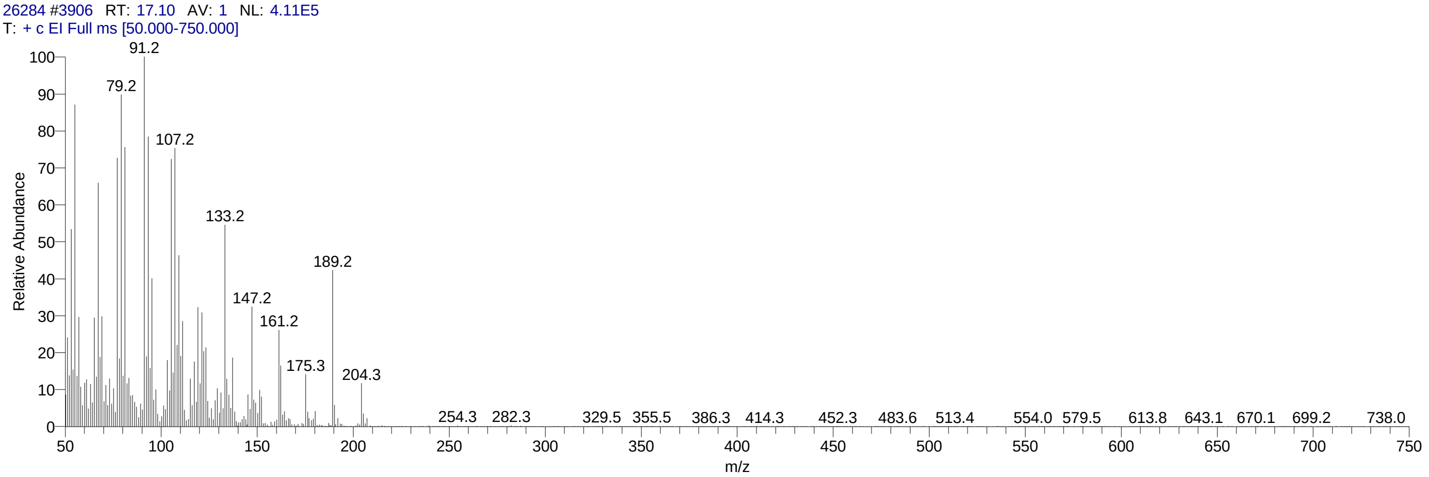

RT	Compound Name	Area %	MF	Molecular Formula	Molecular Weight	Cas #	Library
17.10	1,4-METHANOAZULENE, DECAHYDRO-4,8,8-TRIMETHYL-9-METHYLENE-, [1S-(1à,3Aá,4à,8Aá)]-	0.37	811	C15H24	204	475-20-7	WileyRegistry8e
17.10	Alloaromadendrene	0.37	900	C15H24	204	25246-27-9	replib
17.10	Naphthalene, 1,2,3,4,4a,5,6,8a-octahydro-4a,8-dimethyl-2-(1-methylethenyl)-, [2R-(2à,4aà,8aá)]-	0.37	869	C15H24	204	473-13-2	replib
17.10	à-Bulnesene	0.37	829	C15H24	204	NA	mainlib
17.10	Azulene, 1,2,3,3a,4,5,6,7-octahydro-1,4-dimethyl-7-(1-methylethenyl)-, [1R-(1à,3aá,4à,7á)]-	0.37	840	C15H24	204	22567-17-5	replib

Compound Structure	Hit Spectrum

1,4-METHANOAZULENE, DECAHYDRO-4,8,8-TRIMETHYL-9-METHYLENE-, [1S-(1à,3Aá,4à,8Aá)]-
Formula C15H24, MW 204, CAS# 475-20-7, Entry# 89232
(+)-LONGIFOLEN

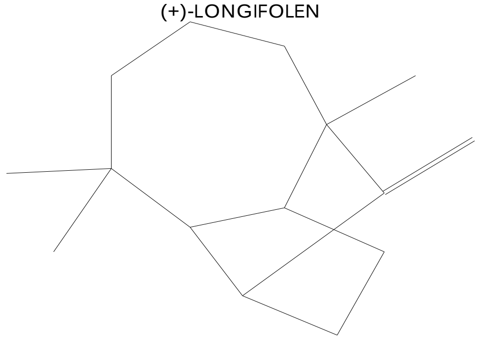

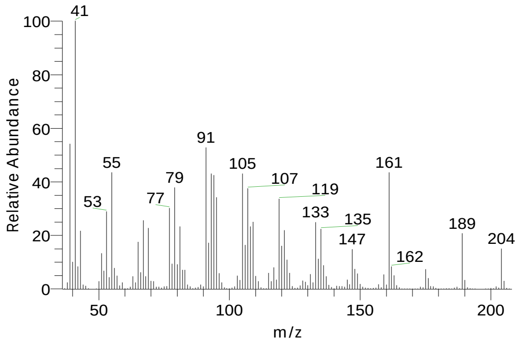

Alloaromadendrene
Formula C15H24, MW 204, CAS# 25246-27-9, Entry# 1320

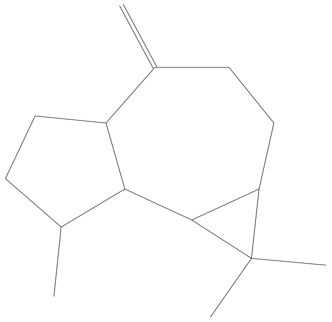

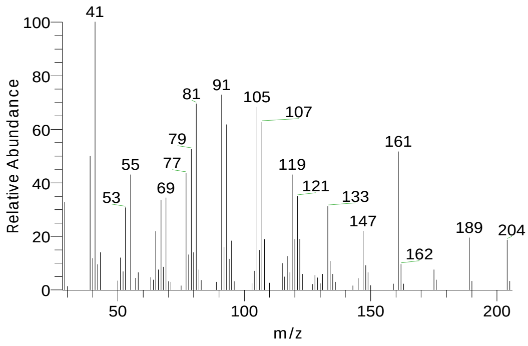

My GC-MS Report

Compound Structure | Hit Spectrum

Formula C15H24, MW 204, CAS# 473-13-2, Entry# 1295
à-Selinene

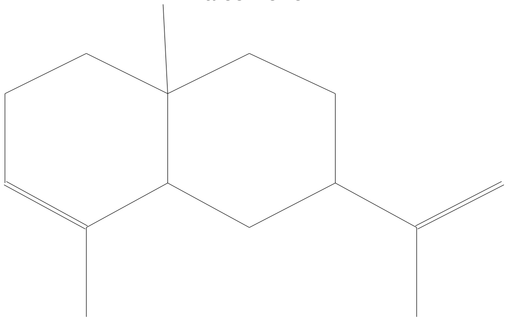

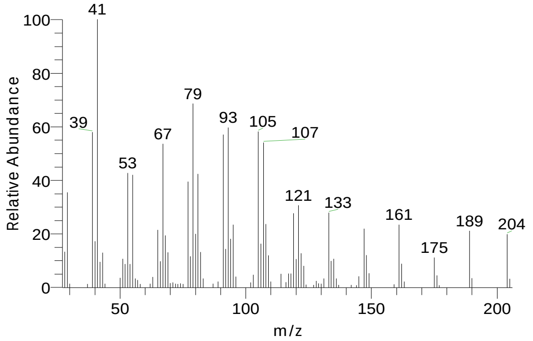

SI 792, RSI 829, mainlib, Entry# 83817, CAS# NA, à-Bulnesene

à-Bulnesene
Formula C15H24, MW 204, CAS# NA, Entry# 83817
$:28YHAJBLWYOIUHHM-UHFFFAOYSA-N

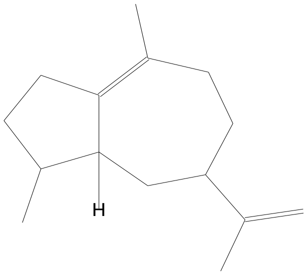

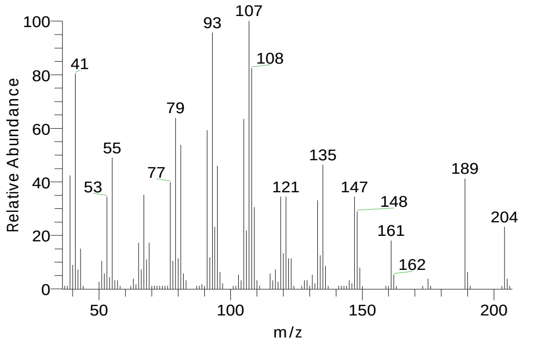

Azulene, 1,2,3,3a,4,5,6,7-octahydro-1,4-dimethyl-7-(1-methylethenyl)-, [1R-(1à,3aá,4à,7á)]-
Formula C15H24, MW 204, CAS# 22567-17-5, Entry# 1321
1á,4áH,10áH-Guaia-5,11-diene

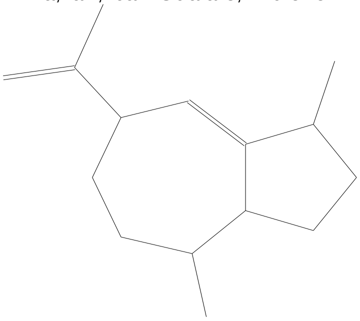

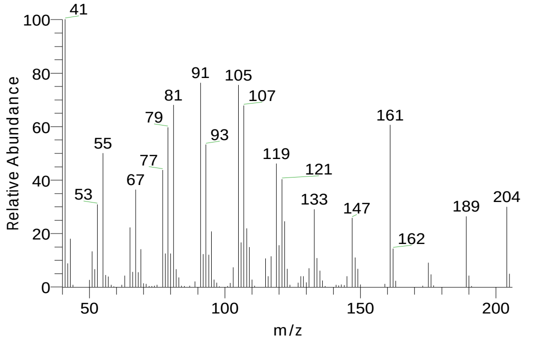

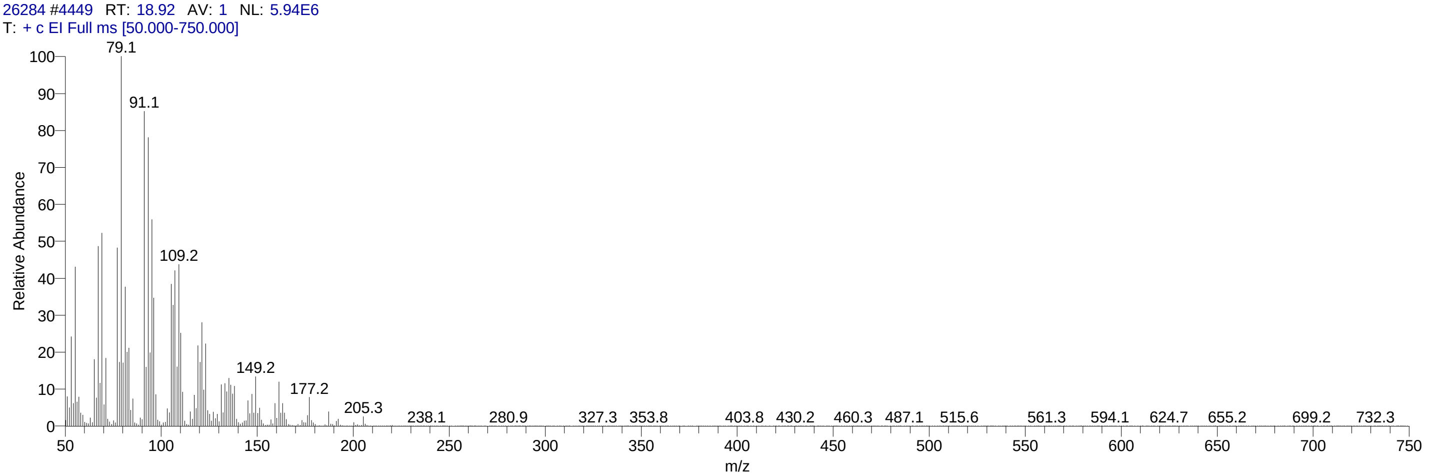

RT	Compound Name	Area %	MF	Molecular Formula	Molecular Weight	Cas #	Library
18.92	(-)-5-OXATRICYCLO[8.2.0.0(4,6)] DODECANE,,12-TRIMETHYL-9-M ETHYLENE-, [1R-(1R*,4R*,6R*,10S*)]-	5.00	953	C15H24O	220	1139-30-6	WileyRegistry8e

My GC-MS Report

RT	Compound Name	Area %	MF	Molecular Formula	Molecular Weight	Cas #	Library
18.92	Caryophyllene oxide	5.00	937	C15H24O	220	1139-30-6	mainlib
18.92	4,12,12-TRIMETHYL-9-METHYLENE-5-OXATRICYCLO[8.2.0.0~4,6~]DODECANE	5.00	937	C15H24O	220	NA	WileyRegistry8e
18.92	(-)-5-OXATRICYCLO[8.2.0.0(4,6)]DODECANE,,12-TRIMETHYL-9-METHYLENE-, [1R-(1R*,4R*,6R*,10S*)]-	5.00	940	C15H24O	220	1139-30-6	WileyRegistry8e
18.92	(-)-5-OXATRICYCLO[8.2.0.0(4,6)]DODECANE,,12-TRIMETHYL-9-METHYLENE-, [1R-(1R*,4R*,6R*,10S*)]-	5.00	940	C15H24O	220	1139-30-6	WileyRegistry8e

Compound Structure	Hit Spectrum

Formula C15H24O, MW 220, CAS# 1139-30-6, Entry# 107245
5-OXATRICYCLO[8.2.0.04,6]DODECANE, 4,12,12-TRIMETHYL-9-METHYLENE-, (1R,4R,6R,10S)-

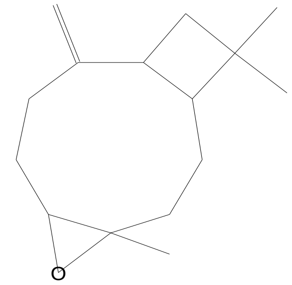

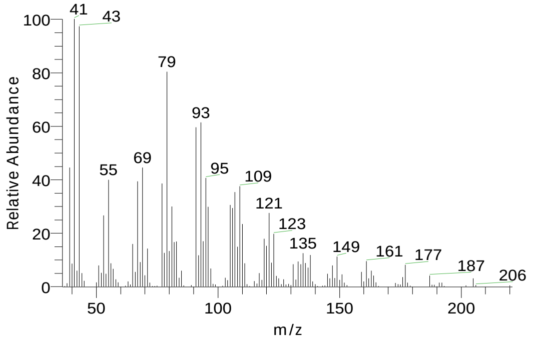

Caryophyllene oxide
Formula C15H24O, MW 220, CAS# 1139-30-6, Entry# 6247

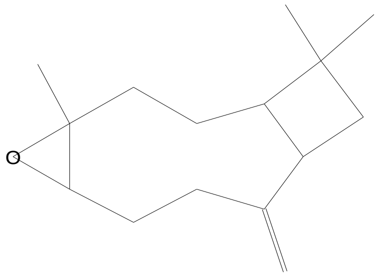

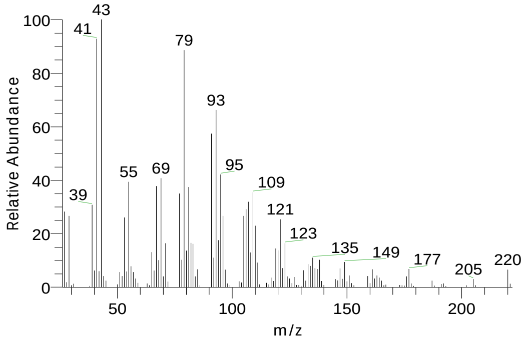

4,12,12-TRIMETHYL-9-METHYLENE-5-OXATRICYCLO[8.2.0.0~4,6~]DODECANE
Formula C15H24O, MW 220, CAS# NA, Entry# 388109
CARYOPHYLLENOXID

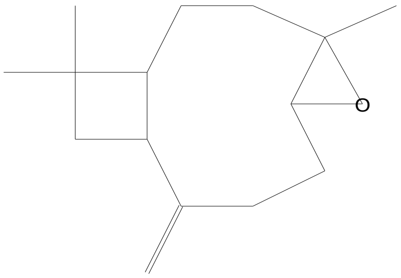

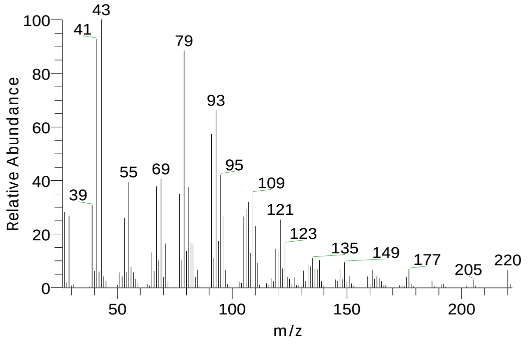

My GC-MS Report

Compound Structure | Hit Spectrum

Formula C15H24O, MW 220, CAS# 1139-30-6, Entry# 107244
5-OXATRICYCLO[8.2.0.04,6]DODECANE, 4,12,12-TRIMETHYL-9-METHYLENE-, (1R,4R,6R,10S)-

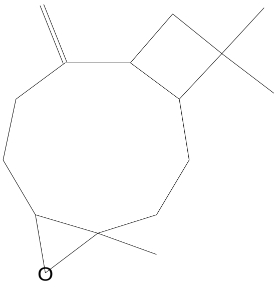

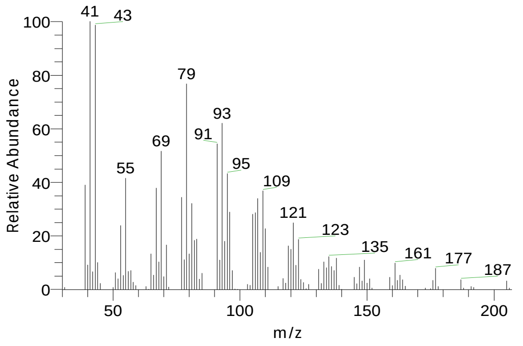

Formula C15H24O, MW 220, CAS# 1139-30-6, Entry# 107246
5-OXATRICYCLO[8.2.0.04,6]DODECANE, 4,12,12-TRIMETHYL-9-METHYLENE-, (1R,4R,6R,10S)-

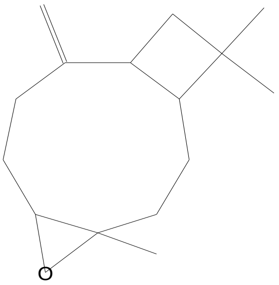

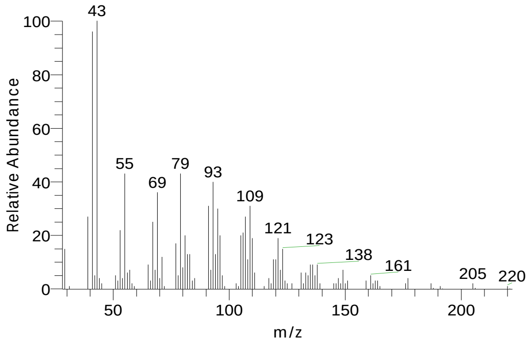

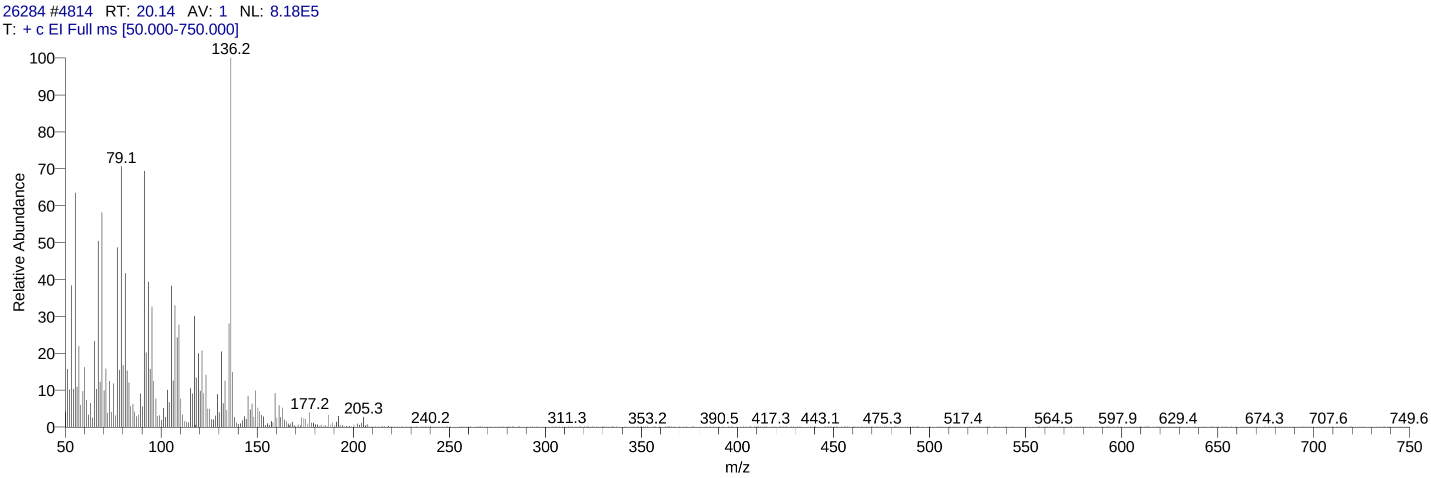

RT	Compound Name	Area %	MF	Molecular Formula	Molecular Weight	Cas #	Library
20.14	Caryophylla-4(12),8(13)-dien-5à-ol	0.56	888	C15H24O	220	19431-79-9	mainlib
20.14	11,11-Dimethyl-4,8-dimethylenebicyclo[7.2.0]undecan-3-ol	0.56	892	C15H24O	220	79580-01-1	mainlib
20.14	10,10-Dimethyl-2,6-dimethylenebicyclo[7.2.0]undecan-5á-ol	0.56	846	C15H24O	220	19431-80-2	mainlib
20.14	Tetracyclo[6.3.2.0(2,5).0(1,8)]tridecan-9-ol, 4,4-dimethyl-	0.56	828	C15H24O	220	NA	mainlib
20.14	TETRACYCLO[6.3.2.0E2,5.0E1,8]TRIDECAN-9-OL, 4,4-DIMETHYL-	0.56	828	C15H24O	220	NA	WileyRegistry8e

My GC-MS Report

Compound Structure | Hit Spectrum

Caryophylla-4(12),8(13)-dien-5à-ol
Formula C15H24O, MW 220, CAS# 19431-79-9, Entry# 122225
Bicyclo[7.2.0]undecan-5-ol, 10,10-dimethyl-2,6-bis(methylene)-, (1S,5R,9R)-

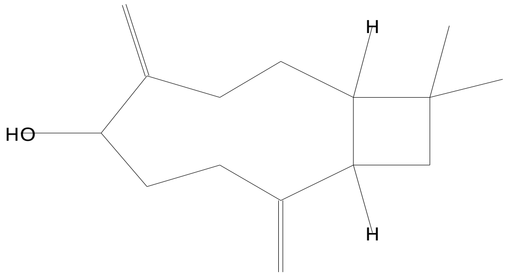

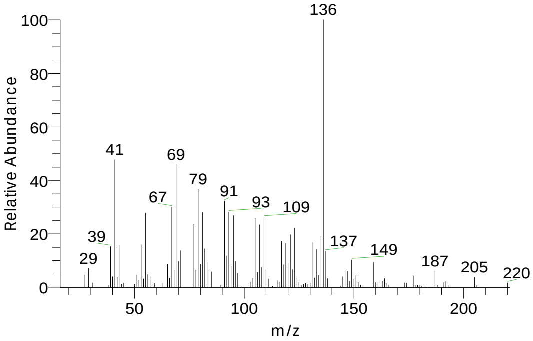

11,11-Dimethyl-4,8-dimethylenebicyclo[7.2.0]undecan-3-ol
Formula C15H24O, MW 220, CAS# 79580-01-1, Entry# 122353
Bicyclo[7.2.0]undecan-3-ol, 11,11-dimethyl-4,8-bis(methylene)-

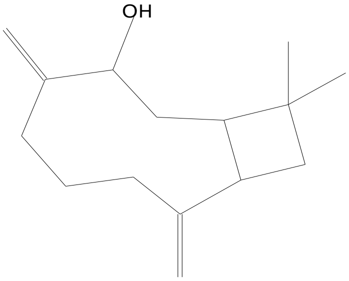

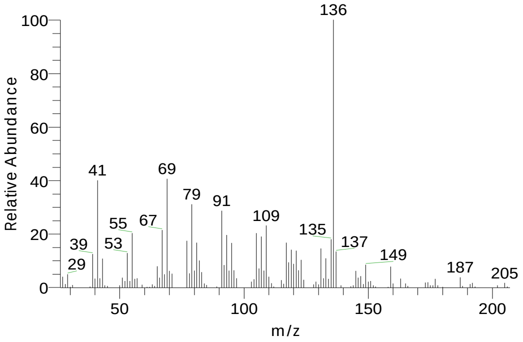

10,10-Dimethyl-2,6-dimethylenebicyclo[7.2.0]undecan-5á-ol
Formula C15H24O, MW 220, CAS# 19431-80-2, Entry# 122355
10,10-Dimethyl-2,6-dimethylenebicyclo[7.2.0]undecan-5-ol #

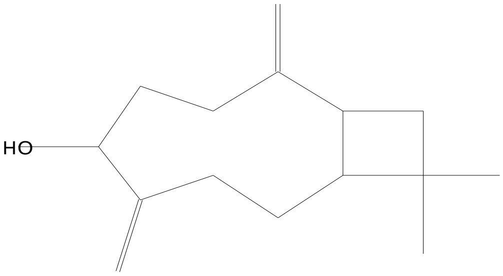

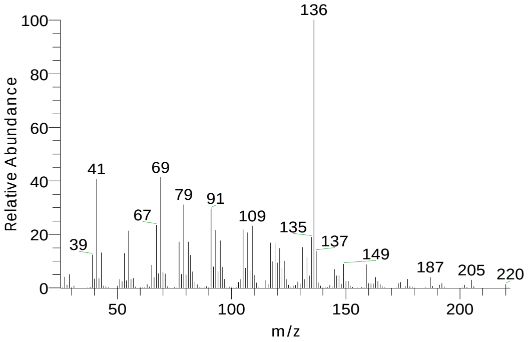

Tetracyclo[6.3.2.0(2,5).0(1,8)]tridecan-9-ol, 4,4-dimethyl-
Formula C15H24O, MW 220, CAS# NA, Entry# 122227
$:28DFBIFSIVSFAGEV-UHFFFAOYSA-N

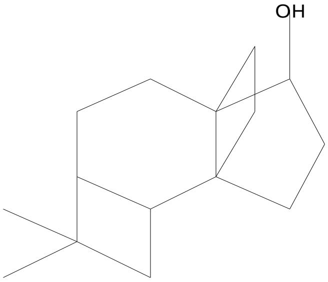

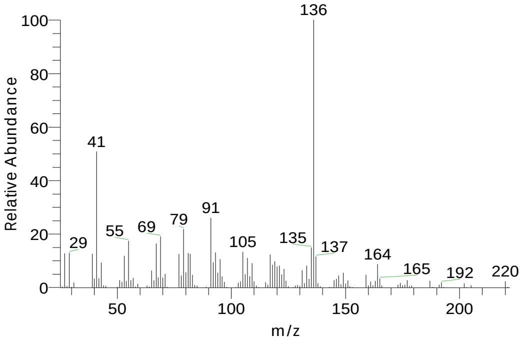

My GC-MS Report

Compound Structure

TETRACYCLO[6.3.2.0E2,5.0E1,8]TRIDECAN-9-OL, 4,4-DIMETHYL-
Formula C15H24O, MW 220, CAS# NA, Entry# 383512

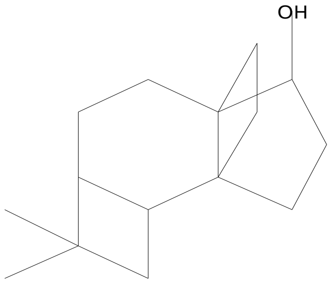

Hit Spectrum

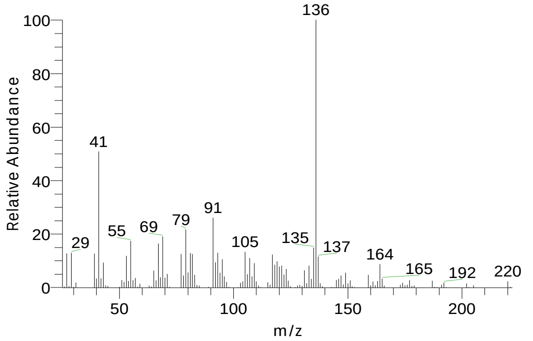

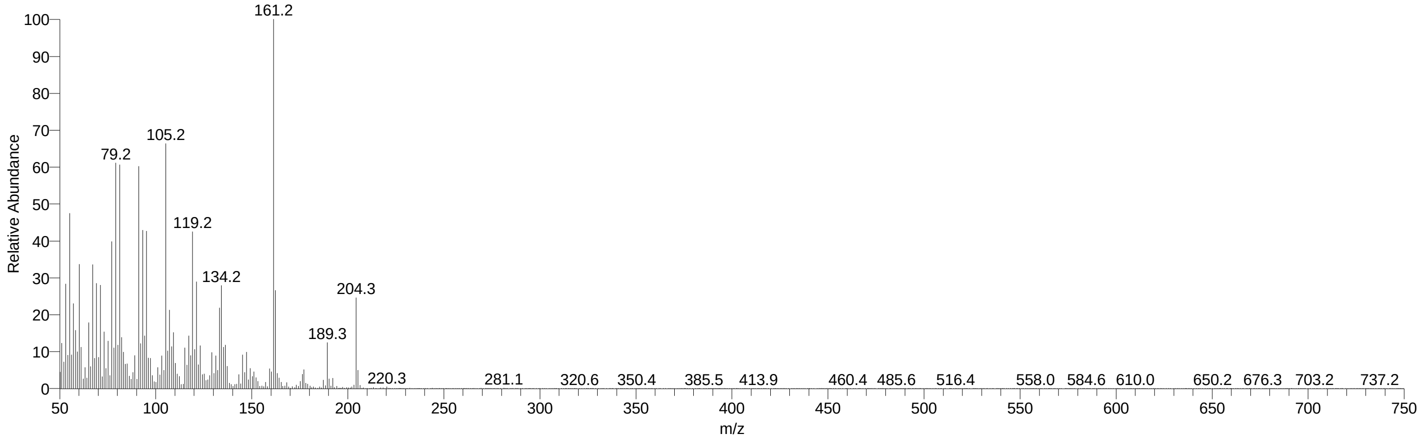

RT	Compound Name	Area %	MF	Molecular Formula	Molecular Weight	Cas #	Library
20.29	á-copaene	0.47	840	C15H24	204	NA	mainlib
20.29	(3R,3aR,3bR,4S,7R,7aR)-4-Isopropyl-3,7-dimethyloctahydro-1H-cyclopenta[1,3]cyclopropa[1,2]benzen-3-ol	0.47	824	C15H26O	222	38230-60-3	replib
20.29	.tau.-Cadinol	0.47	862	C15H26O	222	5937-11-1	mainlib
20.29	á-ylangene	0.47	822	C15H24	204	NA	mainlib
20.29	à-acorenol	0.47	820	C15H26O	222	NA	mainlib

Compound Structure

á-copaene
Formula C15H24, MW 204, CAS# NA, Entry# 150485
$:28UPVZPMJSRSWJHQ-UHFFFAOYSA-N

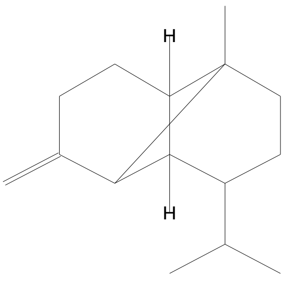

Hit Spectrum

SI 818, RSI 840, mainlib, Entry# 150485, CAS# NA, á-copaene

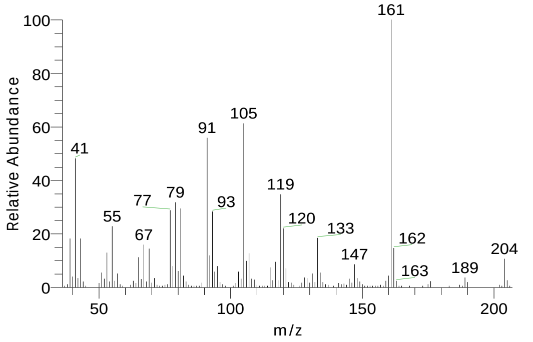

My GC-MS Report

Compound Structure | Hit Spectrum

Formula C15H26O, MW 222, CAS# 38230-60-3, Entry# 24882
Epicubebol

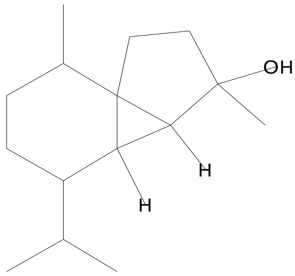

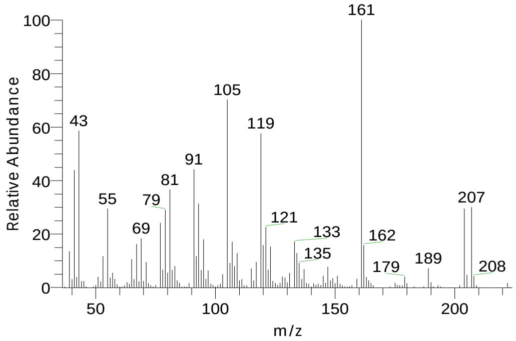

.tau.-Cadinol
Formula C15H26O, MW 222, CAS# 5937-11-1, Entry# 150240

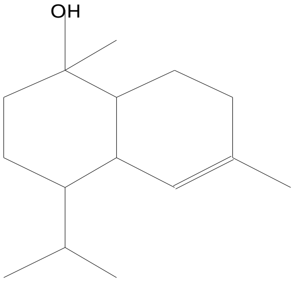

SI 803, RSI 862, mainlib, Entry# 150240, CAS# 5937-11-1, .tau.-Cadinol

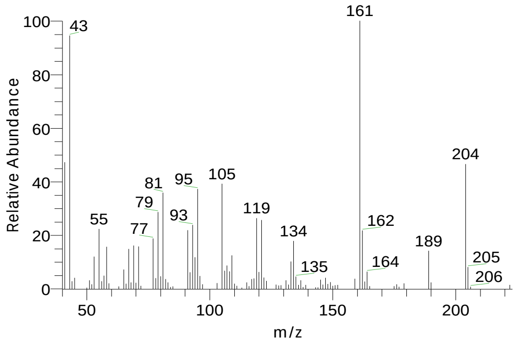

á-ylangene
Formula C15H24, MW 204, CAS# NA, Entry# 101899
$:28UPVZPMJSRSWJHQ-UHFFFAOYSA-N

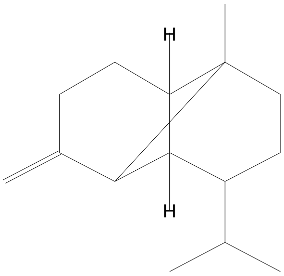

SI 799, RSI 822, mainlib, Entry# 101899, CAS# NA, á-ylangene

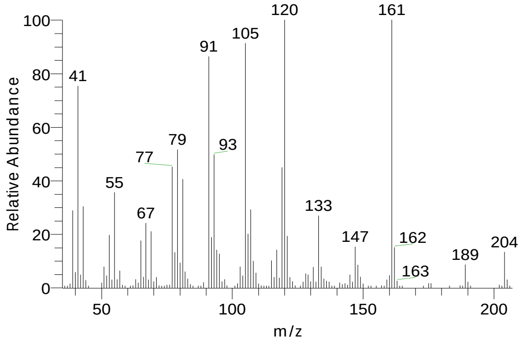

à-acorenol
Formula C15H26O, MW 222, CAS# NA, Entry# 99931
$:28XDVDHFJMCJWDPI-UHFFFAOYSA-N

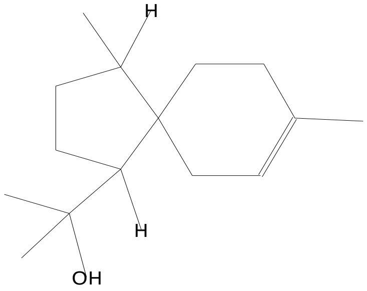

SI 799, RSI 820, mainlib, Entry# 99931, CAS# NA, à-acorenol

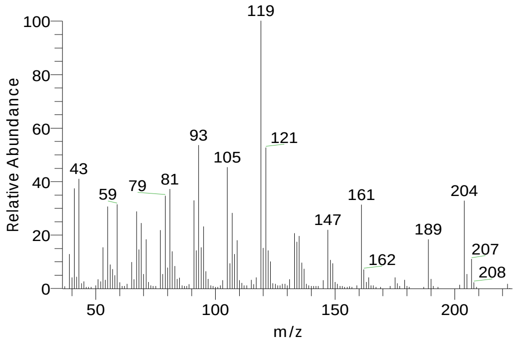

My GC-MS Report

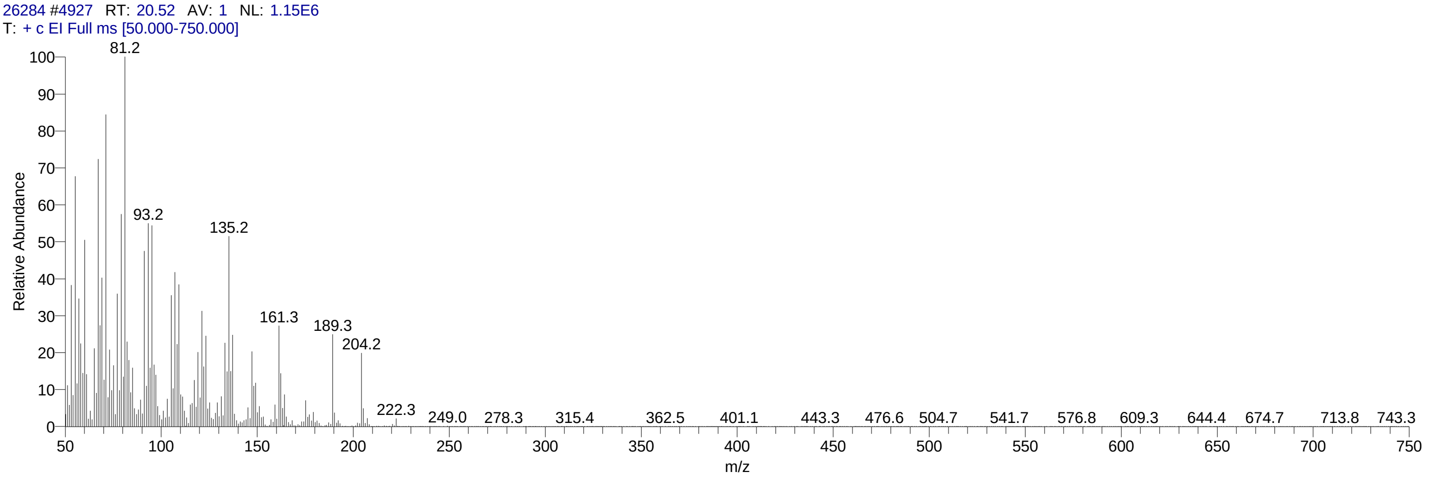

RT	Compound Name	Area %	MF	Molecular Formula	Molecular Weight	Cas #	Library
20.52	Neointermedeol	1.38	867	C15H26O	222	5945-72-2	mainlib
20.52	Epiglobulol	1.38	824	C15H26O	222	NA	mainlib
20.52	1,1,4,7-TETRAMETHYLDECAHYDRO-1H-CYCLOPROPA[E]AZULEN-4-OL #	1.38	824	C15H26O	222	552-02-3	WileyRegistry8e
20.52	1,1,4,7-TETRAMETHYLDECAHYDRO-1H-CYCLOPROPA[E]AZULEN-4-OL	1.38	824	C15H26O	222	NA	WileyRegistry8e
20.52	Globulol	1.38	845	C15H26O	222	51371-47-2	mainlib

Compound Structure	Hit Spectrum

Neointermedeol
Formula C15H26O, MW 222, CAS# 5945-72-2, Entry# 49873
(1S,4aR,7R,8aR)-1,4a-Dimethyl-7-(prop-1-en-2-yl)decahydronaphthalen-1-ol

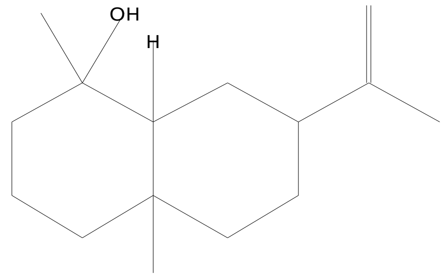

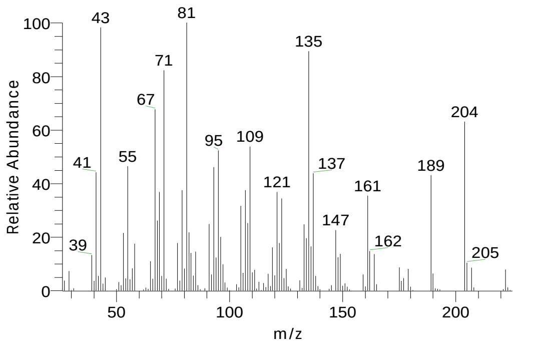

SI 814, RSI 824, mainlib, Entry# 6258, CAS# NA, Epiglobulol

Epiglobulol
Formula C15H26O, MW 222, CAS# NA, Entry# 6258
1,1,4,7-Tetramethyldecahydro-1H-cyclopropa[e]azulen-4-ol #

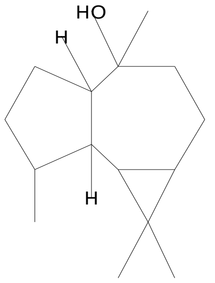

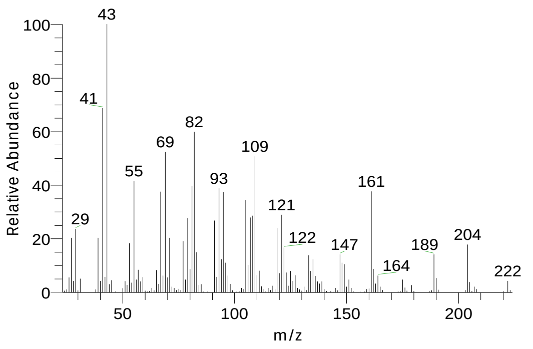

My GC-MS Report

Compound Structure	Hit Spectrum

1,1,4,7-TETRAMETHYLDECAHYDRO-1H-CYCLOPROPA[E]AZULEN-4-OL #
Formula C15H26O, MW 222, CAS# 552-02-3, Entry# 109924

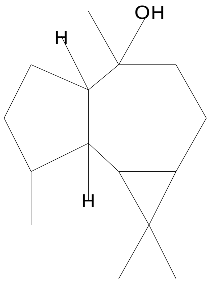

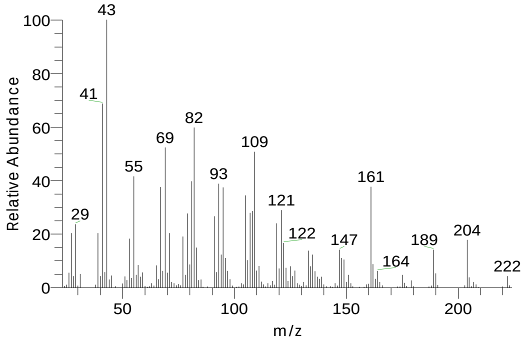

1,1,4,7-TETRAMETHYLDECAHYDRO-1H-CYCLOPROPA[E]AZULEN-4-OL
Formula C15H26O, MW 222, CAS# NA, Entry# 390795
EPIGLOBULOL

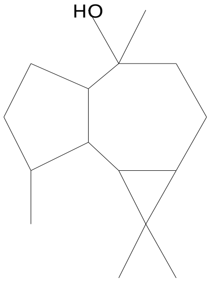

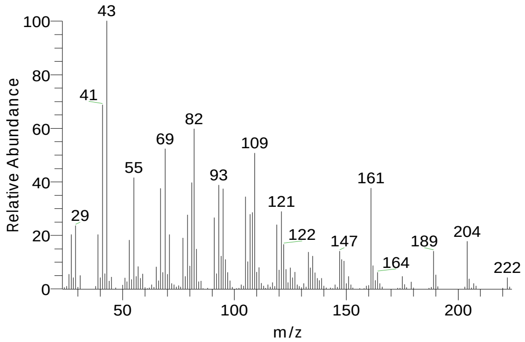

Globulol
Formula C15H26O, MW 222, CAS# 51371-47-2, Entry# 6178
1,1,4,7-Tetramethyldecahydro-1H-cyclopropa[e]azulen-4-ol, (1aà,4à,4aà,7à,7aá,7bà)-

SI 811, RSI 845, mainlib, Entry# 6178, CAS# 51371-47-2, Globulol

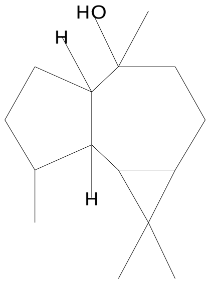

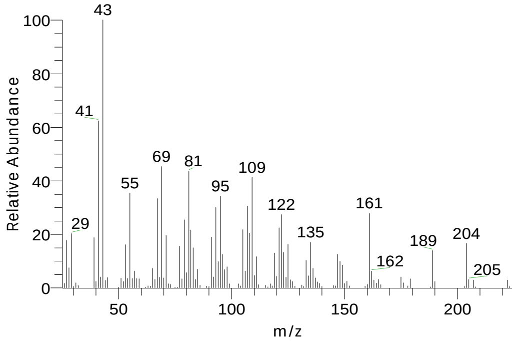

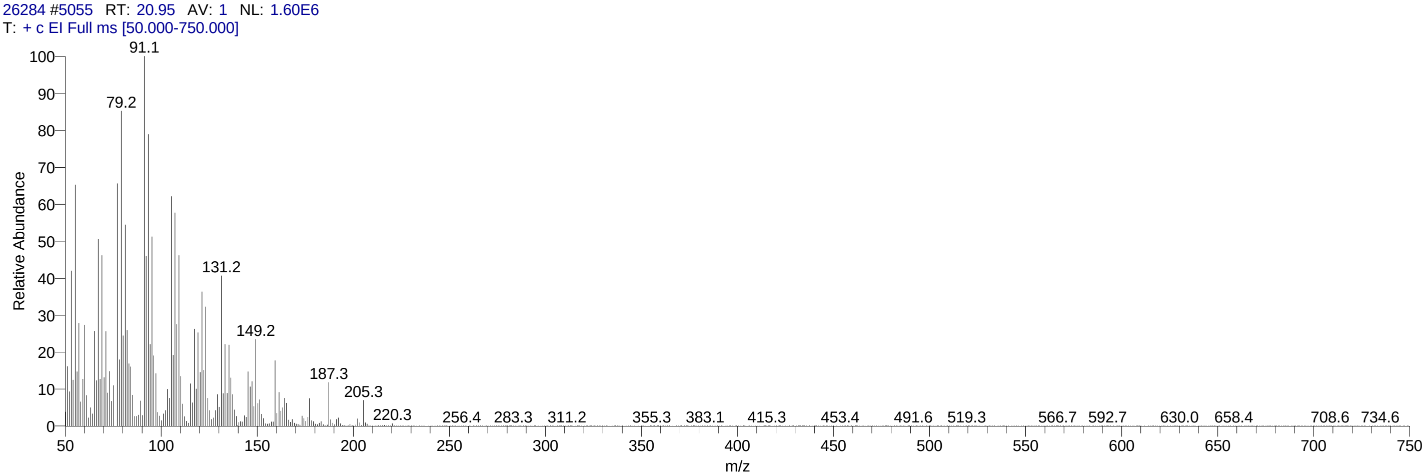

RT	Compound Name	Area %	MF	Molecular Formula	Molecular Weight	Cas #	Library
20.95	Caryophyllene oxide	1.61	884	C15H24O	220	1139-30-6	replib

My GC-MS Report

RT	Compound Name	Area %	MF	Molecular Formula	Molecular Weight	Cas #	Library
20.95	(-)-5-OXATRICYCLO[8.2.0.0(4,6)] DODECANE,,12-TRIMETHYL-9-METHYLENE-, [1R-(1R*,4R*,6R*,10S*)]-	1.61	850	C15H24O	220	1139-30-6	WileyRegistry8e
20.95	trans-Z-à-Bisabolene epoxide	1.61	862	C15H24O	220	NA	mainlib
20.95	Caryophylla-4(12),8(13)-dien-5à-ol	1.61	863	C15H24O	220	19431-79-9	mainlib
20.95	cis-Z-à-Bisabolene epoxide	1.61	815	C15H24O	220	NA	mainlib

Compound Structure | Hit Spectrum

Caryophyllene oxide
Formula C15H24O, MW 220, CAS# 1139-30-6, Entry# 915

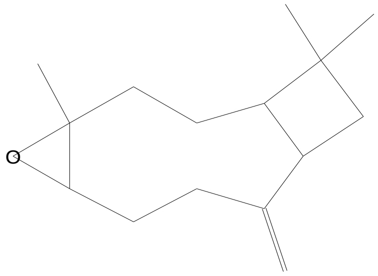

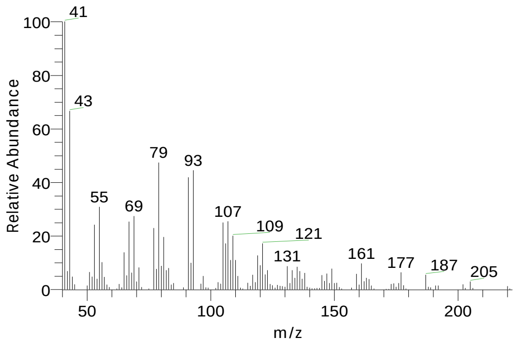

Formula C15H24O, MW 220, CAS# 1139-30-6, Entry# 107245
5-OXATRICYCLO[8.2.0.04,6]DODECANE, 4,12,12-TRIMETHYL-9-METHYLENE-, (1R,4R,6R,10S)-

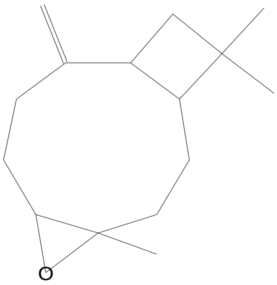

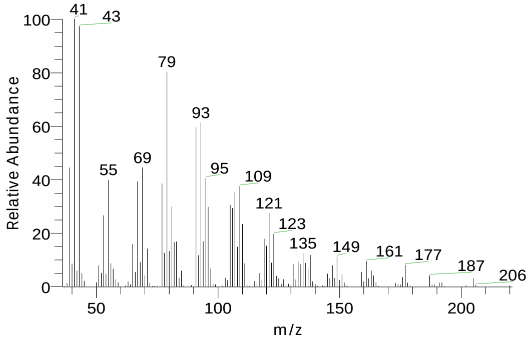

trans-Z-à-Bisabolene epoxide
Formula C15H24O, MW 220, CAS# NA, Entry# 5901
4-[(1Z)-1,5-Dimethyl-1,4-hexadienyl]-1-methyl-7-oxabicyclo[4.1.0]heptane #

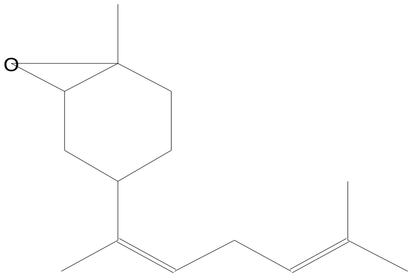

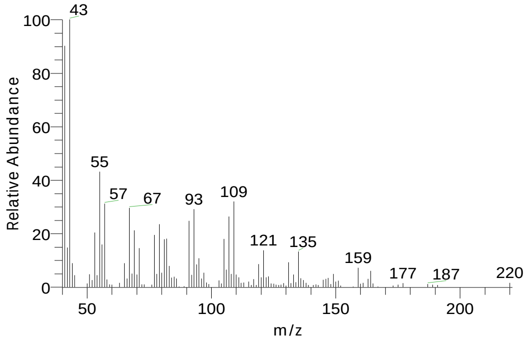

Caryophylla-4(12),8(13)-dien-5à-ol
Formula C15H24O, MW 220, CAS# 19431-79-9, Entry# 122225
Bicyclo[7.2.0]undecan-5-ol, 10,10-dimethyl-2,6-bis(methylene)-, (1S,5R,9R)-

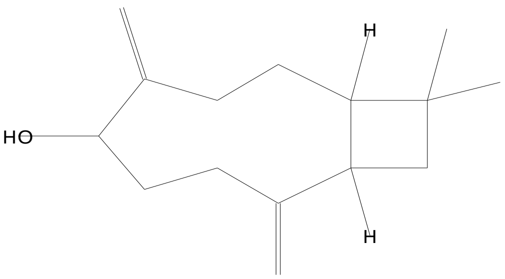

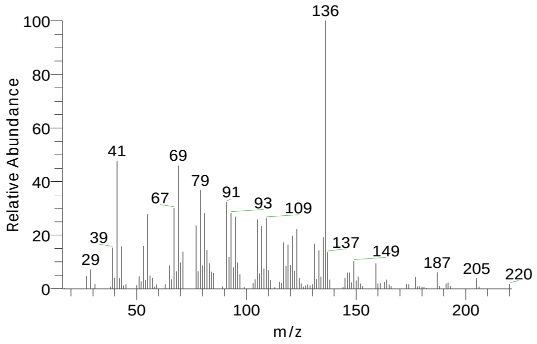

My GC-MS Report

Compound Structure | Hit Spectrum

cis-Z-à-Bisabolene epoxide
Formula C15H24O, MW 220, CAS# NA, Entry# 6348
4-[(1Z)-1,5-Dimethyl-1,4-hexadienyl]-1-methyl-7-oxabicyclo[4.1.0]heptane #

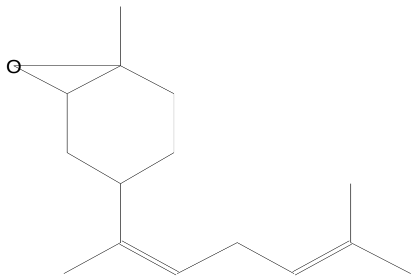

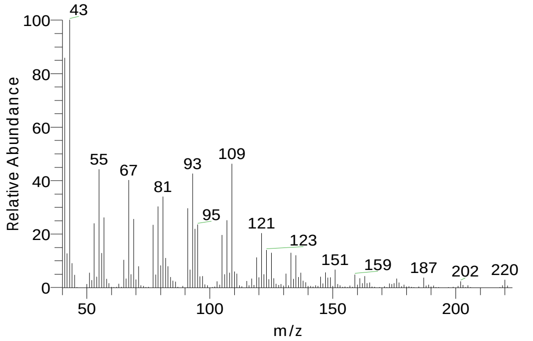

26284 #5275 RT: 21.69 AV: 1 NL: 8.63E5
T: + c EI Full ms [50.000-750.000]

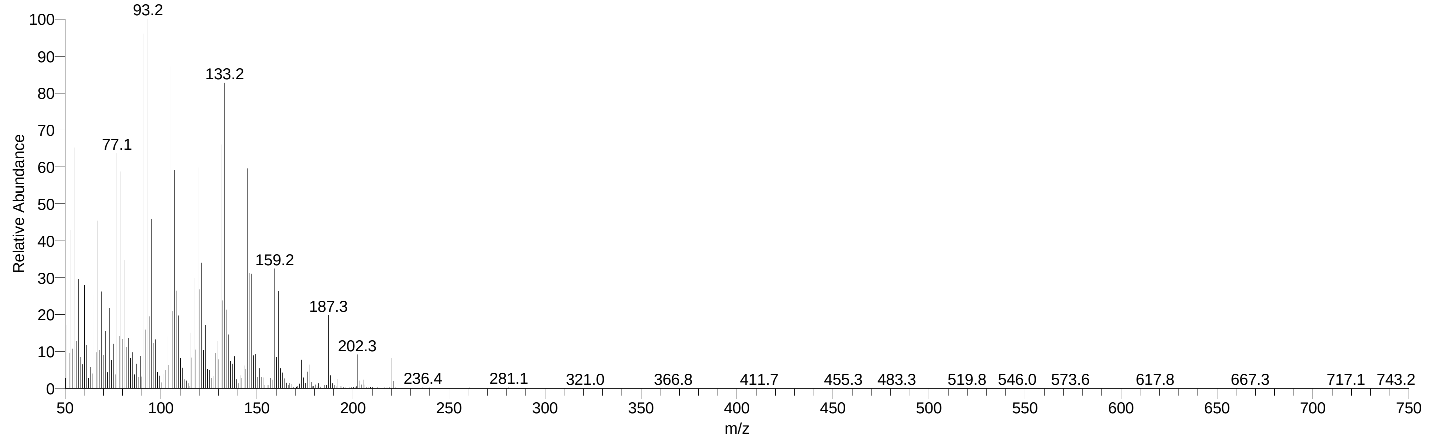

RT	Compound Name	Area %	MF	Molecular Formula	Molecular Weight	Cas #	Library
21.69	Ledene oxide-(II)	0.69	823	C15H24O	220	NA	mainlib
21.69	1,4A,7,7-TETRAMETHYLDECAHYDROCYCLOPROPA[7,8]AZULENO[3A,4-B]OXIRENE	0.69	823	C15H24O	220	NA	WileyRegistry8e
21.69	Cedren-13-ol, 8-	0.69	829	C15H24O	220	18319-35-2	mainlib
21.69	2-((2R,4aR,8aS)-4a-Methyl-8-methylenedecahydronaphthalen-2-yl)prop-2-en-1-ol	0.69	807	C15H24O	220	515-20-8	mainlib
21.69	6-ISOPROPENYL-4,8A-DIMETHYL-1,2,3,5,6,7,8,8A-OCTAHYDRO-2-NAPHTHALENOL	0.69	799	C15H24O	220	NA	WileyRegistry8e

Compound Structure | Hit Spectrum

Ledene oxide-(II)
Formula C15H24O, MW 220, CAS# NA, Entry# 5698
1,4a,7,7-Tetramethyldecahydrocyclopropa[7,8]azuleno[3a,4-b]oxirene #

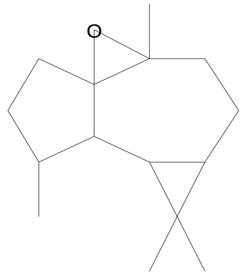

SI 819, RSI 823, mainlib, Entry# 5698, CAS# NA, Ledene oxide-(II)

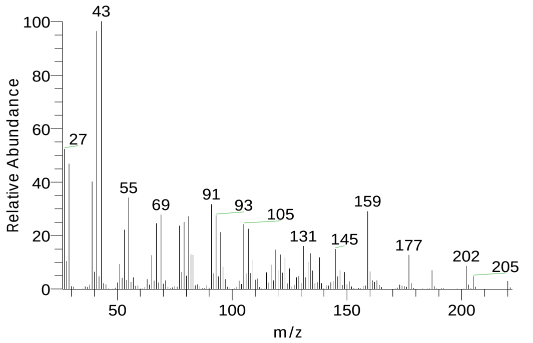

My GC-MS Report

Compound Structure | Hit Spectrum

1,4A,7,7-TETRAMETHYLDECAHYDROCYCLOPROPA[7,8]AZULENO[3A,4-B]OXIRENE
Formula C15H24O, MW 220, CAS# NA, Entry# 381993
LEDENOXID-(II)

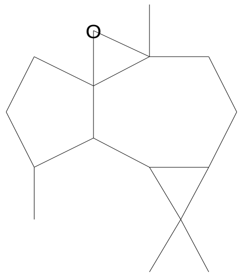

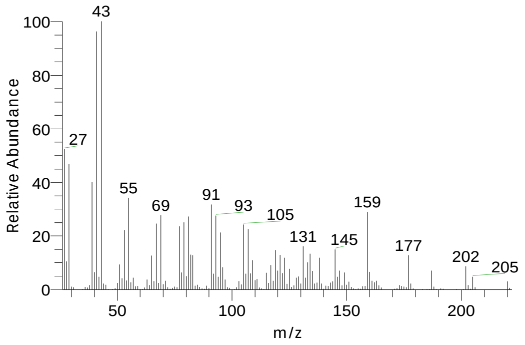

Cedren-13-ol, 8-
Formula C15H24O, MW 220, CAS# 18319-35-2, Entry# 98773
Cedr-8-en-13-ol

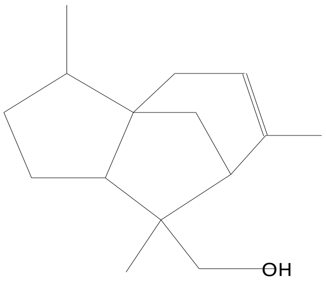

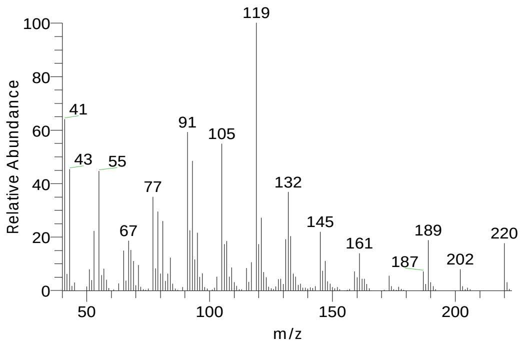

2-((2R,4aR,8aS)-4a-Methyl-8-methylenedecahydronaphthalen-2-yl)prop-2-en-1-ol
Formula C15H24O, MW 220, CAS# 515-20-8, Entry# 80237
2-Naphthaleneethanol, decahydro-4a-methyl-á,8-bis(methylene)-, (2R,4aR,8aS)-

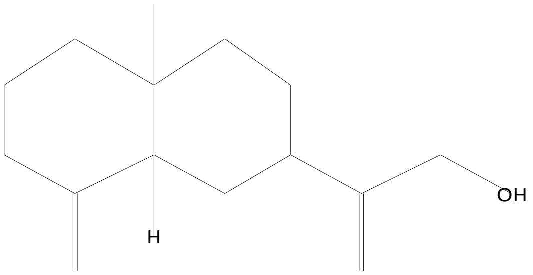

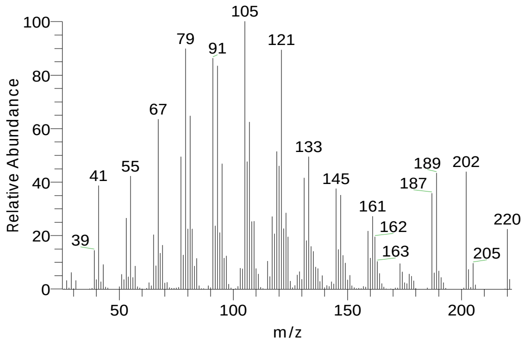

6-ISOPROPENYL-4,8A-DIMETHYL-1,2,3,5,6,7,8,8A-OCTAHYDRO-2-NAPHTHALENOL
Formula C15H24O, MW 220, CAS# NA, Entry# 372611
6-ISOPROPENYL-4,8A-DIMETHYL-1,2,3,5,6,7,8,8A-OCTAHYDRO-NAPHTHALEN-2-OL

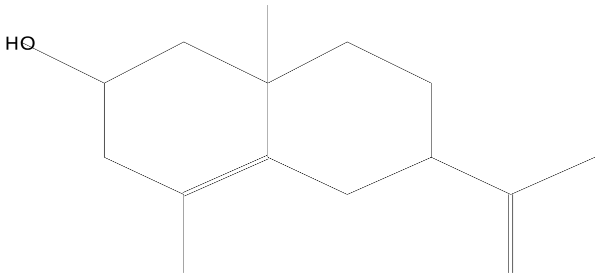

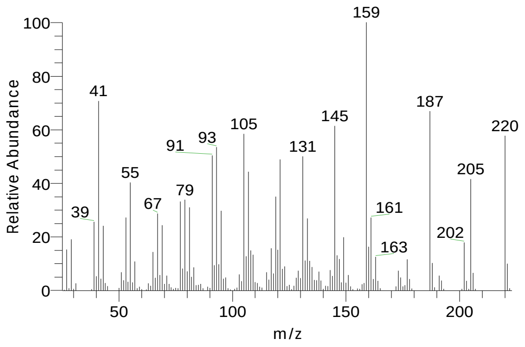

My GC-MS Report

26284 #5322 RT: 21.84 AV: 1 NL: 9.10E5
T: + c EI Full ms [50.000-750.000]

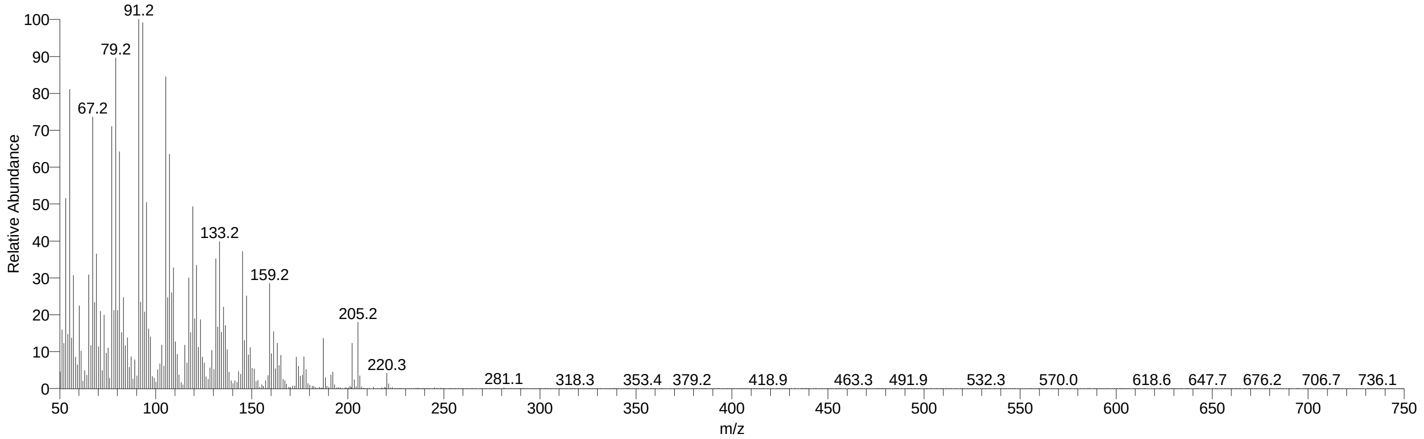

RT	Compound Name	Area %	MF	Molecular Formula	Molecular Weight	Cas #	Library
21.84	2-((2R,4aR,8aS)-4a-Methyl-8-methylenedecahydronaphthalen-2-yl)prop-2-en-1-ol	0.80	811	C15H24O	220	515-20-8	mainlib
21.84	3-METHYL-5-(2,6,6-TRIMETHYL-1-CYCLOHEXEN-1-YL)-1-PENTYN-3-OL	0.80	817	C15H24O	220	NA	WileyRegistry8e
21.84	(-)-Spathulenol	0.80	802	C15H24O	220	77171-55-2	mainlib
21.84	(-)-SPATHULENOL	0.80	801	C15H24O	220	77171-55-2	WileyRegistry8e
21.84	1H-Cycloprop[e]azulen-7-ol, decahydro-1,1,7-trimethyl-4-methylene-, [1ar-(1aà,4aà,7á,7aá,7bà)]-	0.80	800	C15H24O	220	6750-60-3	replib

Compound Structure | Hit Spectrum

2-((2R,4aR,8aS)-4a-Methyl-8-methylenedecahydronaphthalen-2-yl)prop-2-en-1-ol
Formula C15H24O, MW 220, CAS# 515-20-8, Entry# 80237
2-Naphthaleneethanol, decahydro-4a-methyl-á,8-bis(methylene)-, (2R,4aR,8aS)-

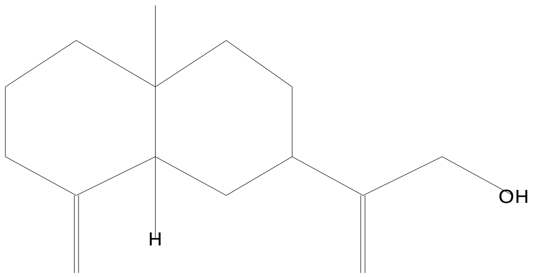

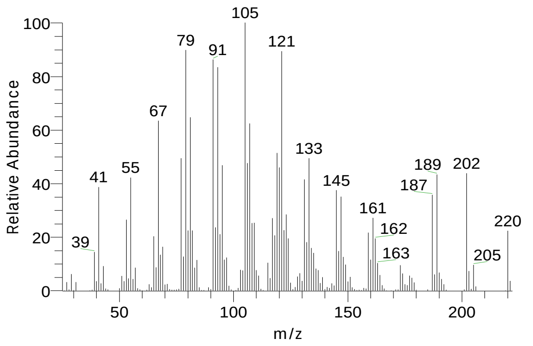

3-METHYL-5-(2,6,6-TRIMETHYL-1-CYCLOHEXEN-1-YL)-1-PENTYN-3-OL
Formula C15H24O, MW 220, CAS# NA, Entry# 322092
PENT-1-YN-3-OL, 3-METHYL-5-(2,6,6-TRIMETHYL-1-CYCLOHEXENYL)-

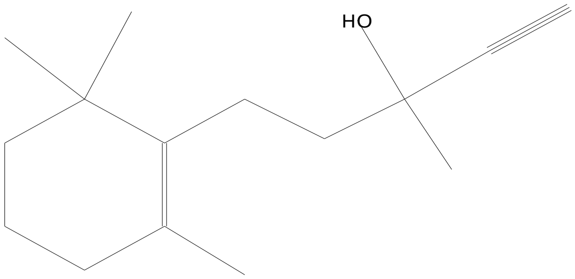

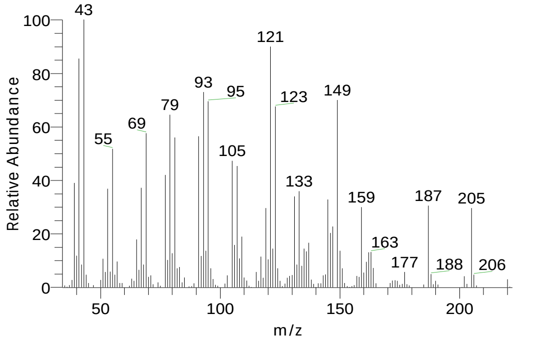

My GC-MS Report

Compound Structure	Hit Spectrum

SI 796, RSI 802, mainlib, Entry# 6311, CAS# 77171-55-2, (-)-Spathulenol

(-)-Spathulenol
Formula C15H24O, MW 220, CAS# 77171-55-2, Entry# 6311
(1aS,4aS,7R,7aS,7bS)-1,1,7-Trimethyl-4-methylenedecahydro-1H-cyclopropa[e]azulen-7-ol

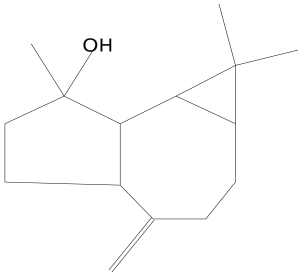

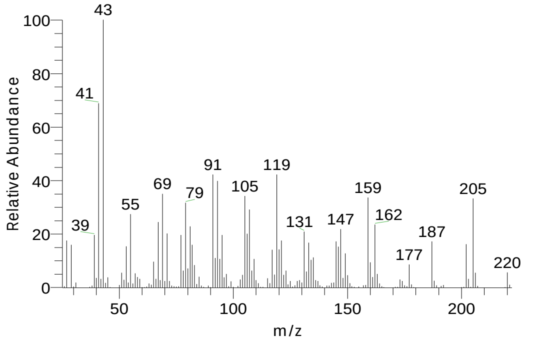

(-)-SPATHULENOL
Formula C15H24O, MW 220, CAS# 77171-55-2, Entry# 107259
1,1,7-TRIMETHYL-4-METHYLENEDECAHYDRO-1H-CYCLOPROPA[E]AZULEN-7-OL #

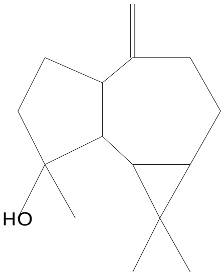

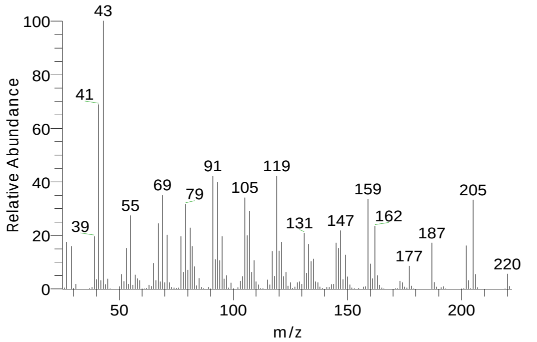

Formula C15H24O, MW 220, CAS# 6750-60-3, Entry# 18515
(1aR,4aR,7S,7aR,7bR)-1,1,7-Trimethyl-4-methylenedecahydro-1H-cyclopropa[e]azulen-7-ol

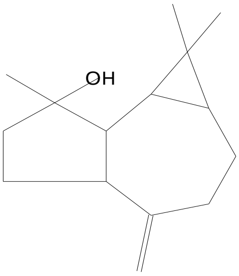

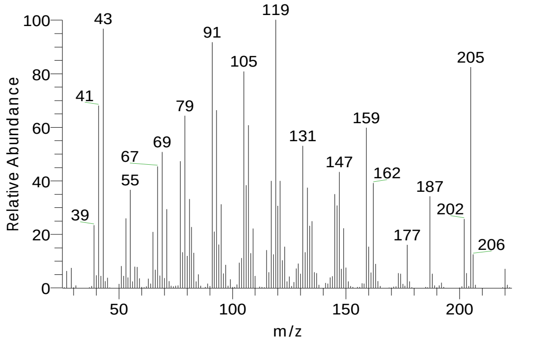

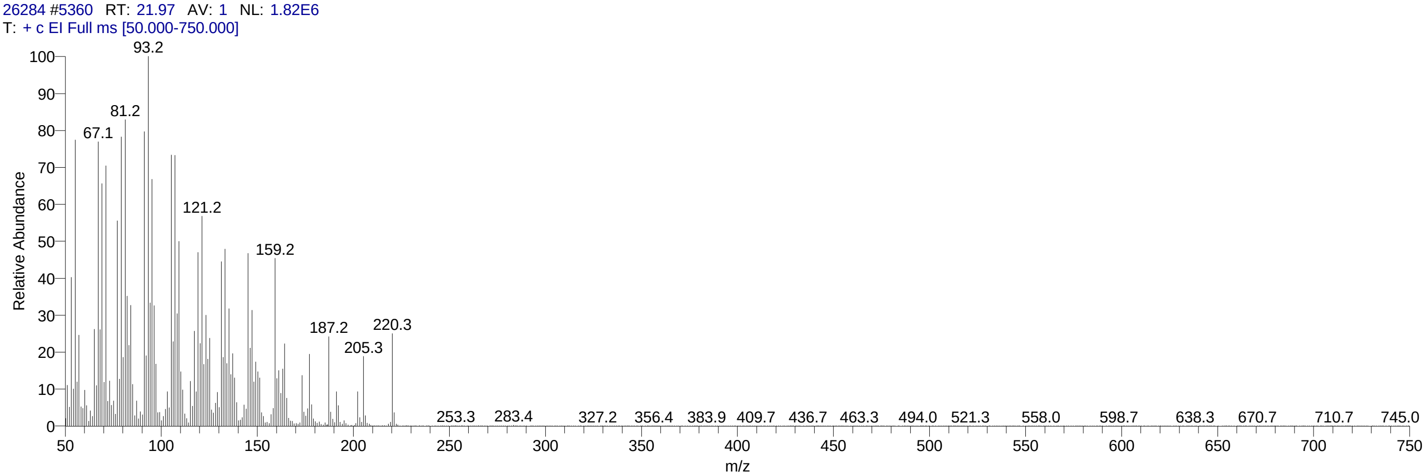

RT	Compound Name	Area %	MF	Molecular Formula	Molecular Weight	Cas #	Library
21.97	Longipinocarveol, trans-	2.44	868	C15H24O	220	NA	mainlib
21.97	2,2,6-TRIMETHYL-10-METHYLENETRICYCLO[5.3.1.0~1,6~]UNDECAN-9-OL	2.44	868	C15H24O	220	NA	WileyRegistry8e
21.97	Aromadendrene oxide-(2)	2.44	874	C15H24O	220	NA	mainlib

My GC-MS Report

RT	Compound Name	Area %	MF	Molecular Formula	Molecular Weight	Cas #	Library
21.97	AROMADENDRENOXID-(2)	2.44	874	C15H24O	220	NA	WileyRegistry8e
21.97	ALLOAROMADENDRENOXID-(1)	2.44	871	C15H24O	220	85710-39-0	WileyRegistry8e

Compound Structure | Hit Spectrum

Longipinocarveol, trans-
Formula C15H24O, MW 220, CAS# NA, Entry# 2806
$:28QZIBYWPRLPFUFP-UHFFFAOYSA-N

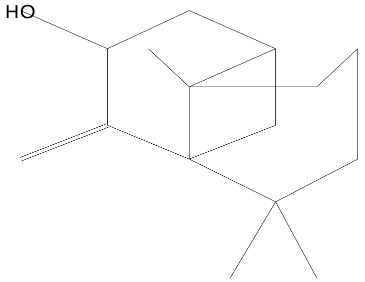

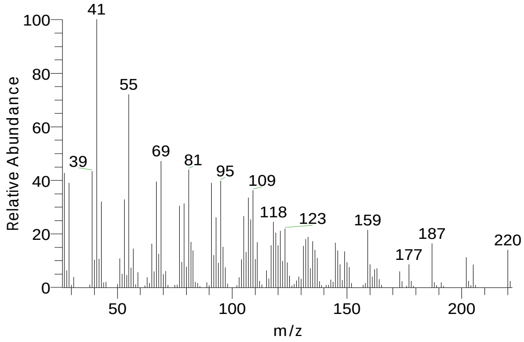

2,2,6-TRIMETHYL-10-METHYLENETRICYCLO[5.3.1.0~1,6~]UNDECAN-9-OL
Formula C15H24O, MW 220, CAS# NA, Entry# 381995
LONGIPINOCARVEOL, TRANS-

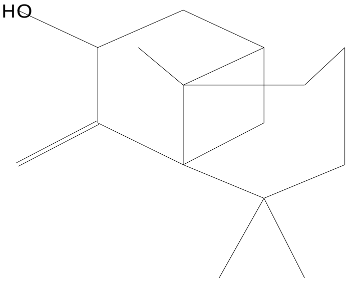

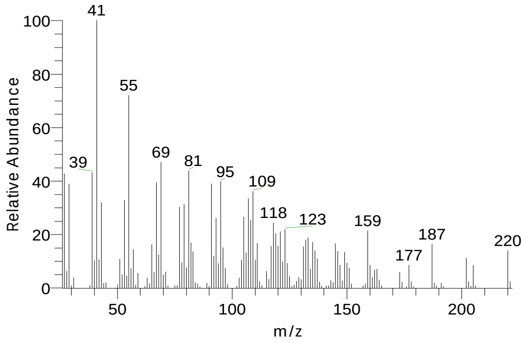

Aromadendrene oxide-(2)
Formula C15H24O, MW 220, CAS# NA, Entry# 2891
$:28XPGWKKLDFXNBPJ-UHFFFAOYSA-N

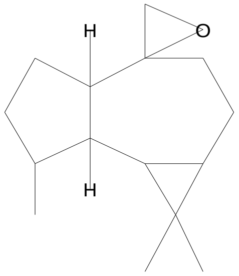

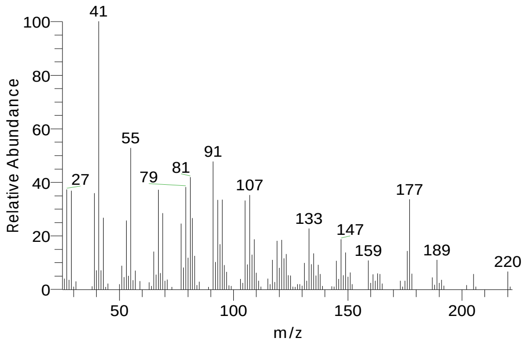

AROMADENDRENOXID-(2)
Formula C15H24O, MW 220, CAS# NA, Entry# 387063

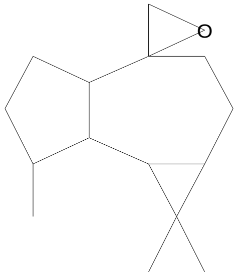

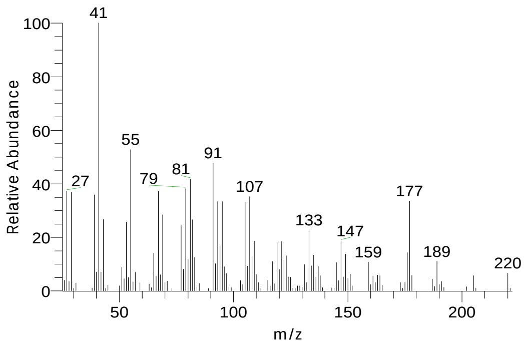

My GC-MS Report

Compound Structure | Hit Spectrum

ALLOAROMADENDRENOXID-(1)
Formula $C_{15}H_{24}O$, MW 220, CAS# 85710-39-0, Entry# 107243
AROMADENDRENEPOXIDE-(I)

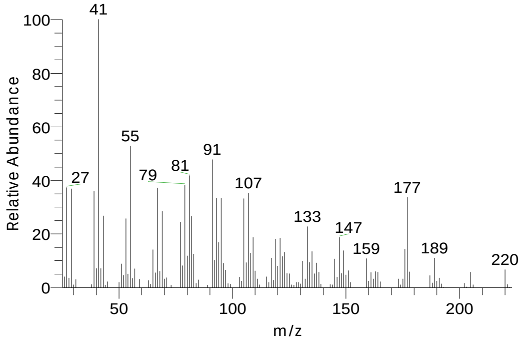

26284 #5495 RT: 22.43 AV: 1 NL: 4.77E5
T: + c EI Full ms [50.000-750.000]

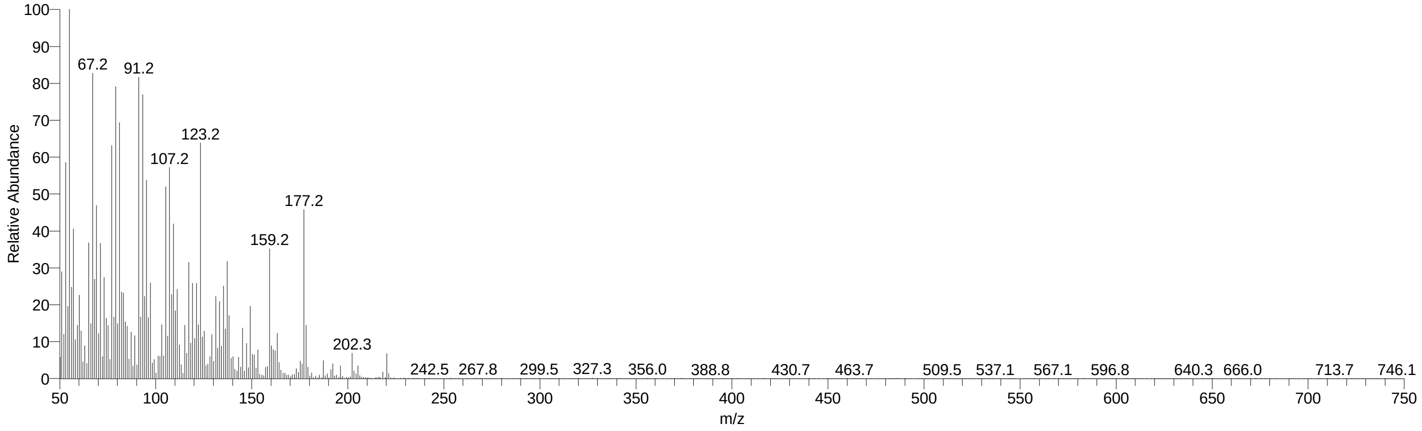

RT	Compound Name	Area %	MF	Molecular Formula	Molecular Weight	Cas #	Library
22.43	Ledene oxide-(II)	0.47	819	C15H24O	220	NA	mainlib
22.43	1,4A,7,7-TETRAMETHYLDECAHYDROCYCLOPROPA[7,8]AZULENO[3A,4-B]OXIRENE	0.47	819	C15H24O	220	NA	WileyRegistry8e
22.43	Aromadendrene oxide-(2)	0.47	852	C15H24O	220	NA	mainlib
22.43	AROMADENDRENOXID-(2)	0.47	852	C15H24O	220	NA	WileyRegistry8e
22.43	ALLOAROMADENDRENOXID-(1)	0.47	850	C15H24O	220	85710-39-0	WileyRegistry8e

Compound Structure | Hit Spectrum

Ledene oxide-(II)
Formula $C_{15}H_{24}O$, MW 220, CAS# NA, Entry# 5698
1,4a,7,7-Tetramethyldecahydrocyclopropa[7,8]azuleno[3a,4-b]oxirene #

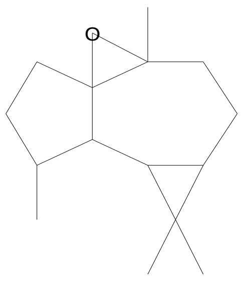

SI 811, RSI 819, mainlib, Entry# 5698, CAS# NA, Ledene oxide-(II)

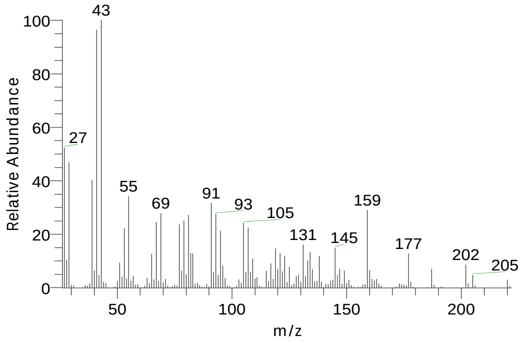

My GC-MS Report

Compound Structure | Hit Spectrum

1,4A,7,7-TETRAMETHYLDECAHYDROCYCLOPROPA[7,8]AZULENO[3A,4-B]OXIRENE
Formula C15H24O, MW 220, CAS# NA, Entry# 381993
LEDENOXID-(II)

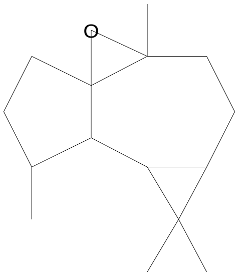

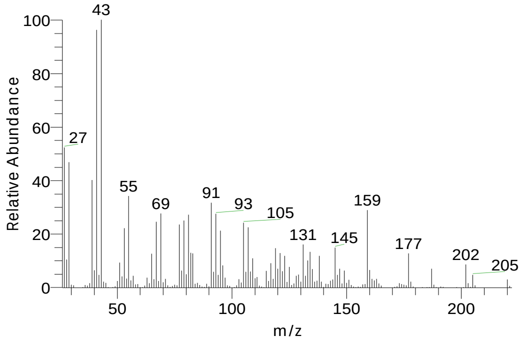

Aromadendrene oxide-(2)
Formula C15H24O, MW 220, CAS# NA, Entry# 2891
$:28XPGWKKLDFXNBPJ-UHFFFAOYSA-N

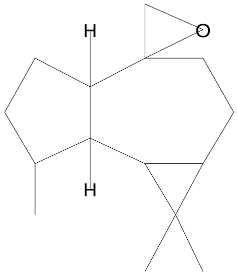

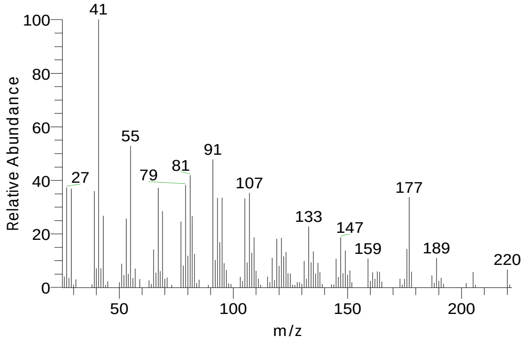

AROMADENDRENOXID-(2)
Formula C15H24O, MW 220, CAS# NA, Entry# 387063

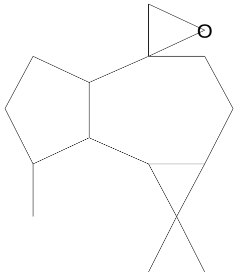

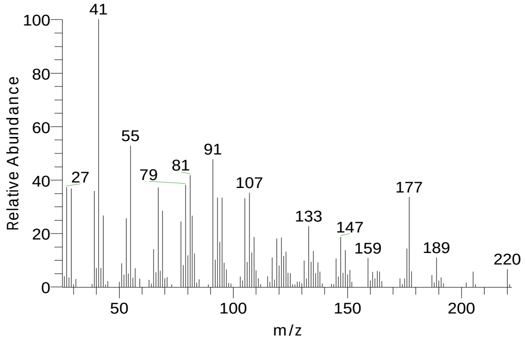

ALLOAROMADENDRENOXID-(1)
Formula C15H24O, MW 220, CAS# 85710-39-0, Entry# 107243
AROMADENDRENEPOXIDE-(I)

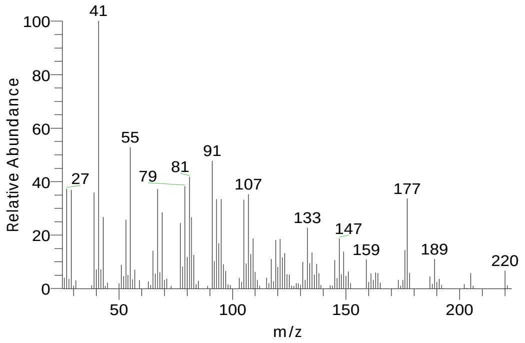

My GC-MS Report

26284 #5851 RT: 23.62 AV: 1 NL: 9.86E5
T: + c EI Full ms [50.000-750.000]

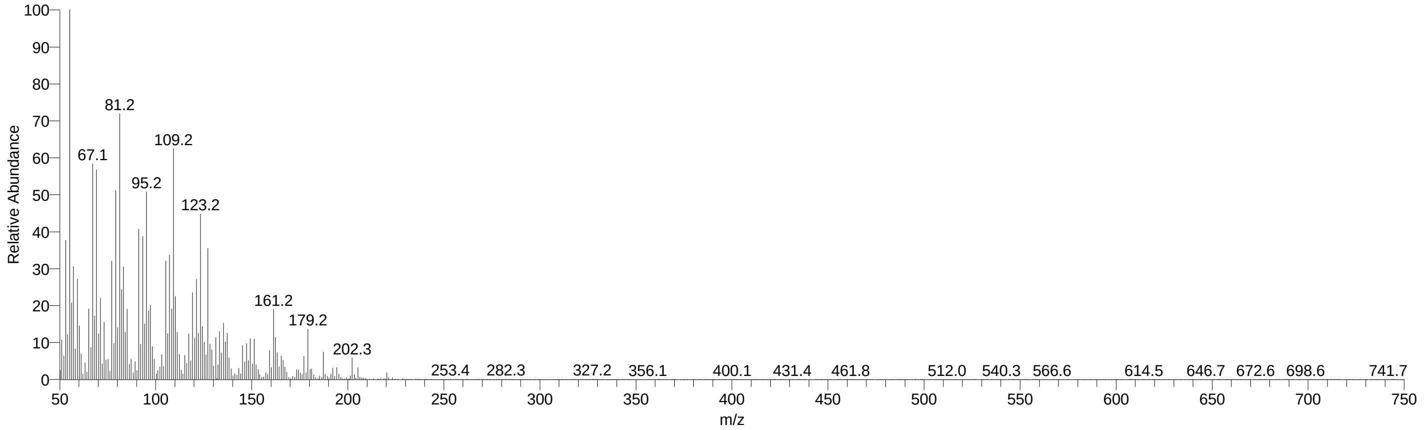

RT	Compound Name	Area %	MF	Molecular Formula	Molecular Weight	Cas #	Library
23.62	Cholestan-3-ol, 2-methylene-, (3á,5à)-	0.94	828	C28H48O	400	22599-96-8	mainlib
23.62	CHOLESTAN-3-OL, 2-METHYLENE-, (3á,5à)-	0.94	827	C28H48O	400	22599-96-8	WileyRegistry8e
23.62	cis-Z-à-Bisabolene epoxide	0.94	815	C15H24O	220	NA	mainlib
23.62	5,8,11,14-Eicosatetraenoic acid, methyl ester, (all-Z)-	0.94	792	C21H34O2	318	2566-89-4	replib
23.62	CYCLOOCTENONE, DIMER	0.94	786	C16H24O2	248	61219-51-0	WileyRegistry8e

Compound Structure | Hit Spectrum

Cholestan-3-ol, 2-methylene-, (3á,5à)-
Formula C28H48O, MW 400, CAS# 22599-96-8, Entry# 35411
5à-Cholestan-3á-ol, 2-methylene-

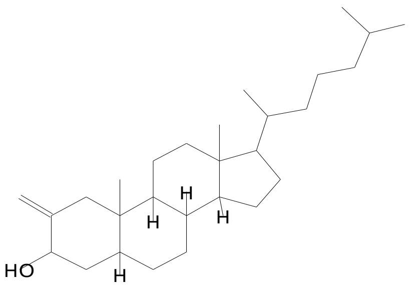

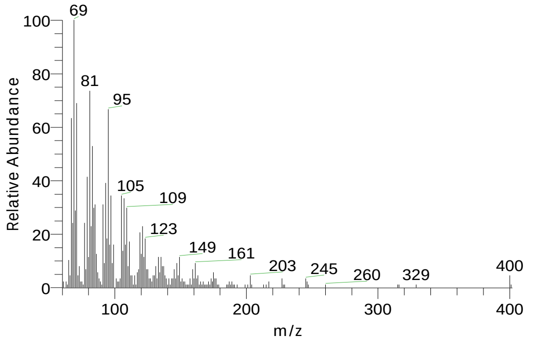

CHOLESTAN-3-OL, 2-METHYLENE-, (3á,5à)-
Formula C28H48O, MW 400, CAS# 22599-96-8, Entry# 256365
2-METHYLENECHOLESTAN-3-OL #

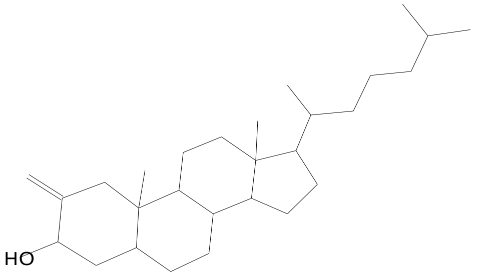

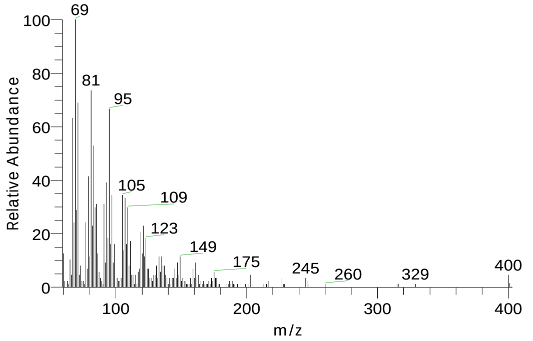

My GC-MS Report

Compound Structure | Hit Spectrum

cis-Z-à-Bisabolene epoxide
Formula C15H24O, MW 220, CAS# NA, Entry# 6348
4-[(1Z)-1,5-Dimethyl-1,4-hexadienyl]-1-methyl-7-oxabicyclo[4.1.0]heptane #

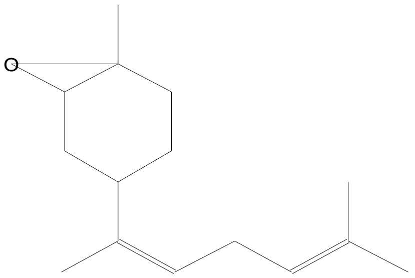

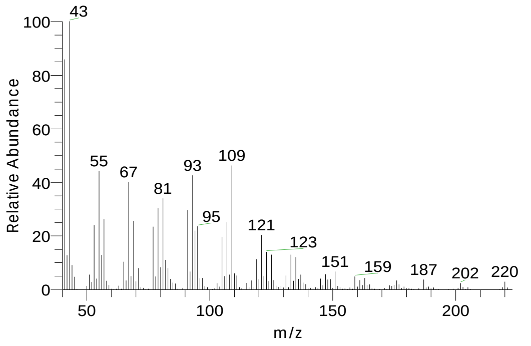

5,8,11,14-Eicosatetraenoic acid, methyl ester, (all-Z)-
Formula C21H34O2, MW 318, CAS# 2566-89-4, Entry# 916
Arachidonic acid methyl ester

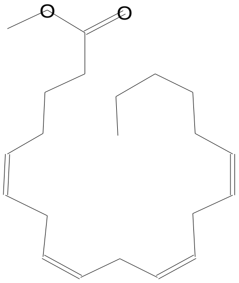

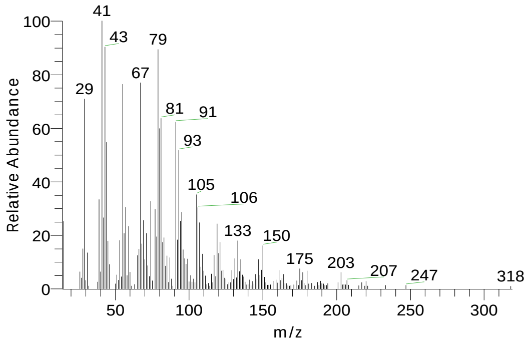

CYCLOOCTENONE, DIMER
Formula C16H24O2, MW 248, CAS# 61219-51-0, Entry# 138182
TRICYCLO[8.6.0.0E2,9]HEXADECA-8,16,KOPF,SCHWANZ-DION, CIS-2,9-TRANSOID-9,10-CIS-

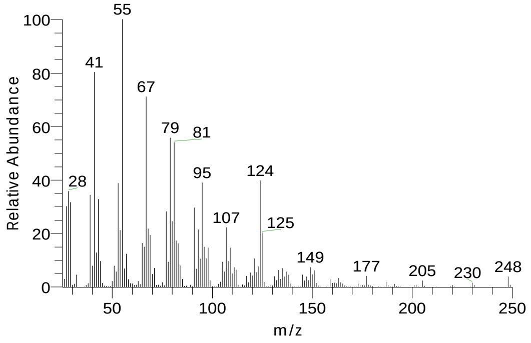

26284 #6236 RT: 24.91 AV: 1 NL: 1.22E6
T: + c EI Full ms [50.000-750.000]

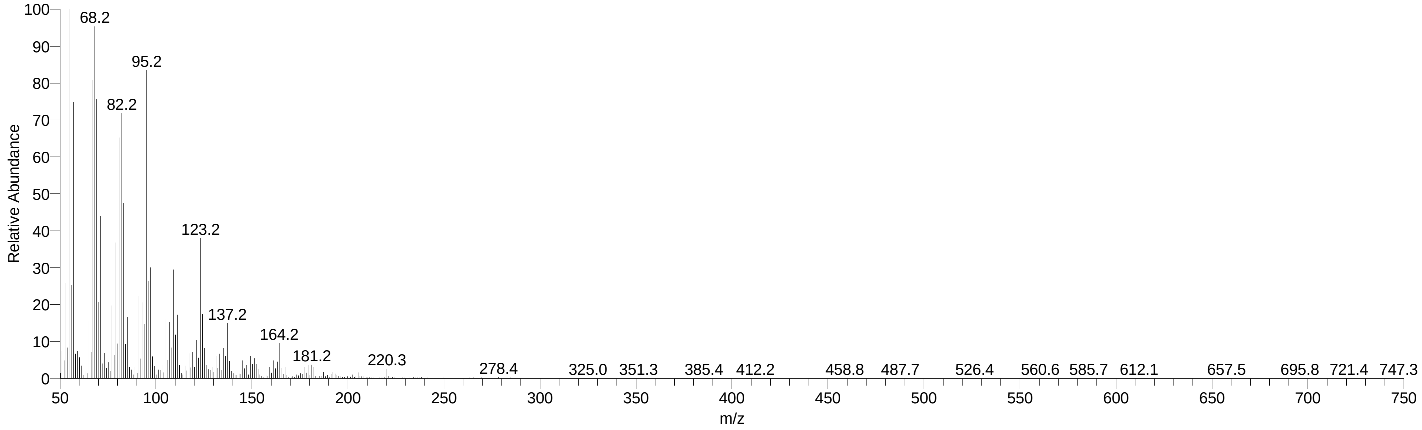

RT	Compound Name	Area %	MF	Molecular Formula	Molecular Weight	Cas #	Library
24.91	2-[12-(2-OXIRANYL)DODECYL] OXIRANE	0.81	807	C16H30O2	254	NA	WileyRegistry8e
24.91	1,2-15,16-Diepoxyhexadecane	0.81	807	C16H30O2	254	NA	mainlib
24.91	17-Octadecynoic acid	0.81	792	C18H32O2	280	34450-18-5	mainlib

My GC-MS Report

RT	Compound Name	Area %	MF	Molecular Formula	Molecular Weight	Cas #	Library
24.91	9,12-Octadecadienoic acid (Z,Z)-	0.81	788	C18H32O2	280	60-33-3	replib
24.91	3,7,11,15-Tetramethyl-2-hexadecen-1-ol	0.81	904	C20H40O	296	102608-53-7	mainlib

Compound Structure | Hit Spectrum

2-[12-(2-OXIRANYL)DODECYL]OXIRANE
Formula C16H30O2, MW 254, CAS# NA, Entry# 369137

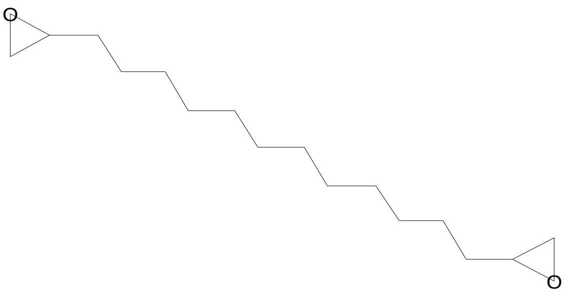

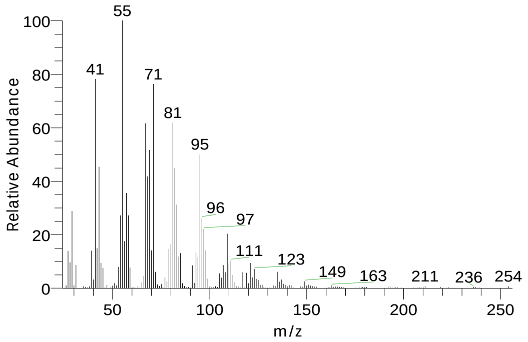

1,2-15,16-Diepoxyhexadecane
Formula C16H30O2, MW 254, CAS# NA, Entry# 19336
2-[12-(2-Oxiranyl)dodecyl]oxirane #

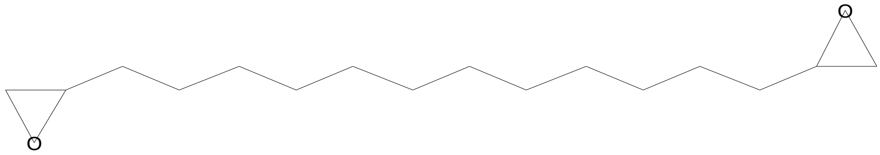

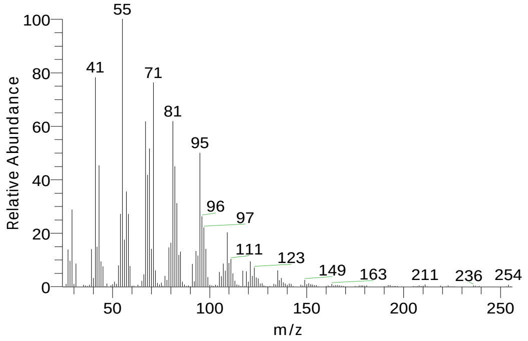

17-Octadecynoic acid
Formula C18H32O2, MW 280, CAS# 34450-18-5, Entry# 20510
$:28DZIILFGADWDKMF-UHFFFAOYSA-N

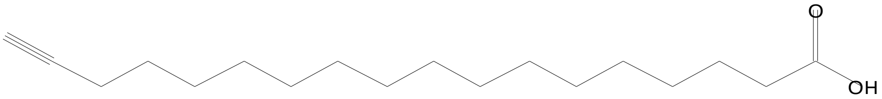

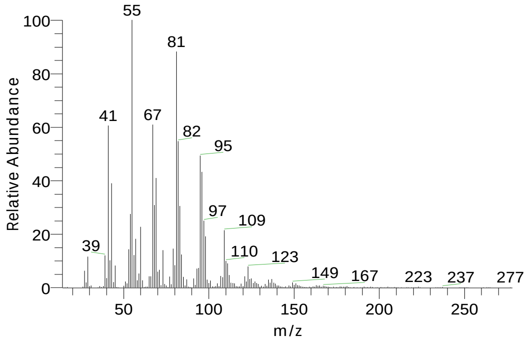

9,12-Octadecadienoic acid (Z,Z)-
Formula C18H32O2, MW 280, CAS# 60-33-3, Entry# 8112
cis-9,cis-12-Octadecadienoic acid

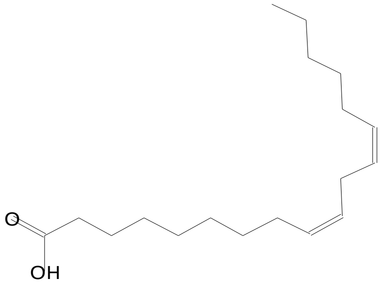

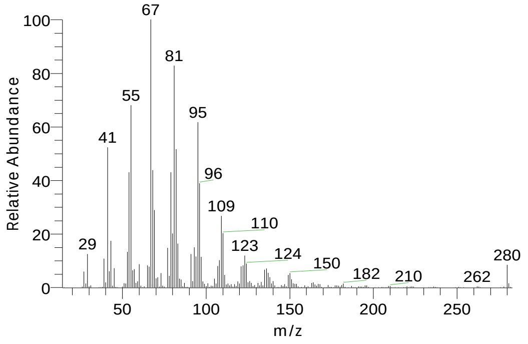

My GC-MS Report

Compound Structure | Hit Spectrum

3,7,11,15-Tetramethyl-2-hexadecen-1-ol
Formula C20H40O, MW 296, CAS# 102608-53-7, Entry# 50359
2-Hexadecen-1-ol, 3,7,11,15-tetramethyl

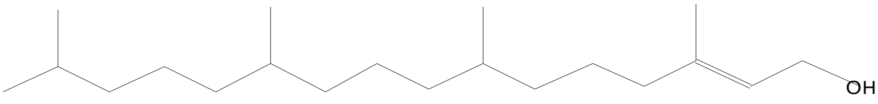

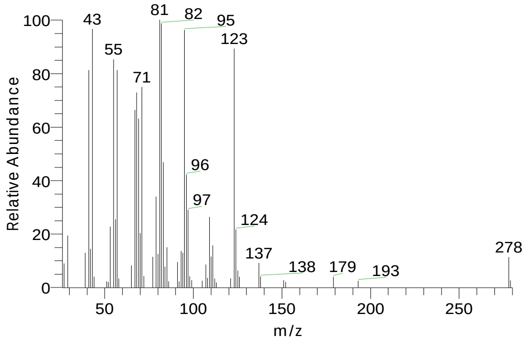

26284 #6325 RT: 25.21 AV: 1 NL: 5.46E5
T: + c EI Full ms [50.000-750.000]

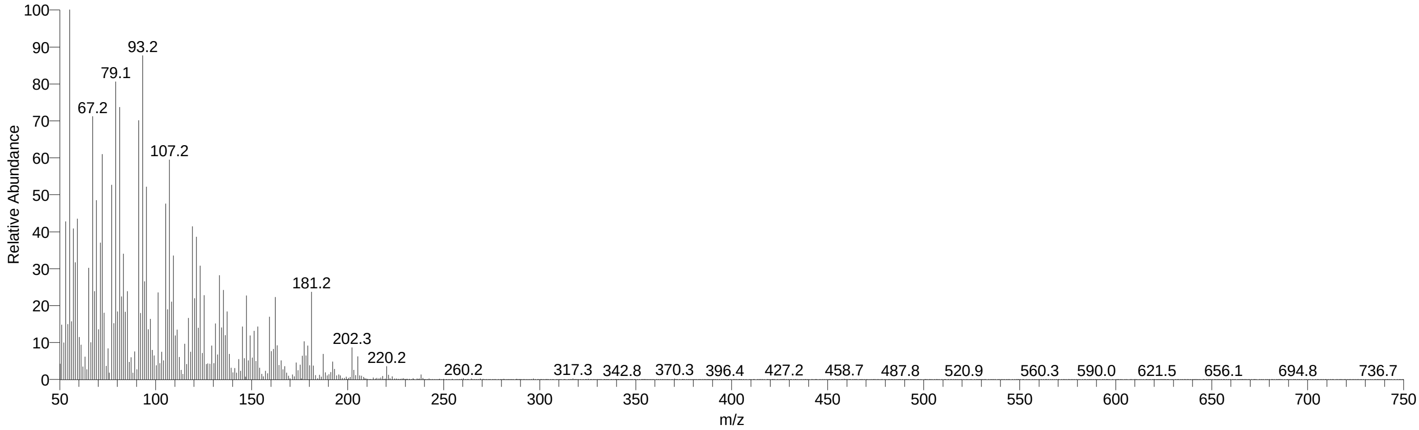

RT	Compound Name	Area %	MF	Molecular Formula	Molecular Weight	Cas #	Library
25.21	Isoaromadendrene epoxide	0.58	876	C15H24O	220	NA	mainlib
25.21	1,3B,6,6-TETRAMETHYLDECAHYDRO-1H-CYCLOPROPA[7,8]AZULENO[4,5-B]OXIRENE	0.58	876	C15H24O	220	NA	WileyRegistry8e
25.21	Aromadendrene oxide-(2)	0.58	846	C15H24O	220	NA	mainlib
25.21	AROMADENDRENOXID-(2)	0.58	846	C15H24O	220	NA	WileyRegistry8e
25.21	ALLOAROMADENDRENOXID-(1)	0.58	841	C15H24O	220	85710-39-0	WileyRegistry8e

Compound Structure | Hit Spectrum

Isoaromadendrene epoxide
Formula C15H24O, MW 220, CAS# NA, Entry# 2371
1,3b,6,6-Tetramethyldecahydro-1H-cyclopropa[7,8]azuleno[4,5-b]oxirene #

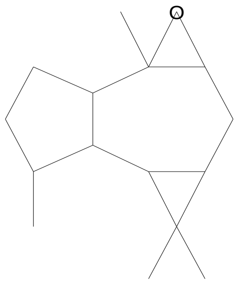

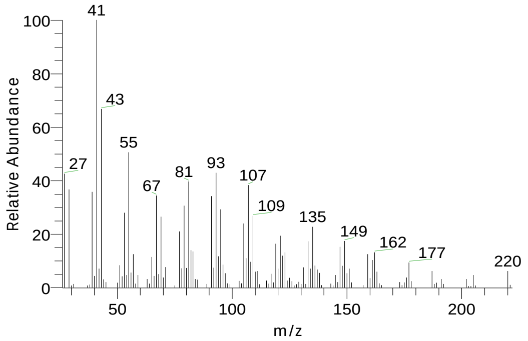

My GC-MS Report

Compound Structure | Hit Spectrum

1,3B,6,6-TETRAMETHYLDECAHYDRO-1H-CYCLOPROPA[7,8]AZULENO[4,5-B]OXIRENE
Formula C15H24O, MW 220, CAS# NA, Entry# 381994
ISOAROMADENDRENEPOXID

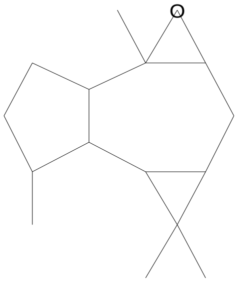

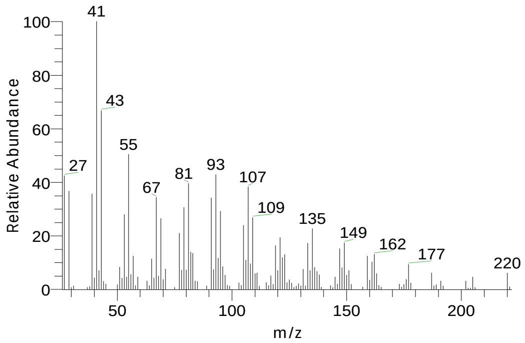

Aromadendrene oxide-(2)
Formula C15H24O, MW 220, CAS# NA, Entry# 2891
$:28XPGWKKLDFXNBPJ-UHFFFAOYSA-N

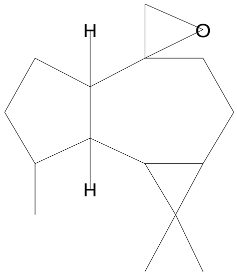

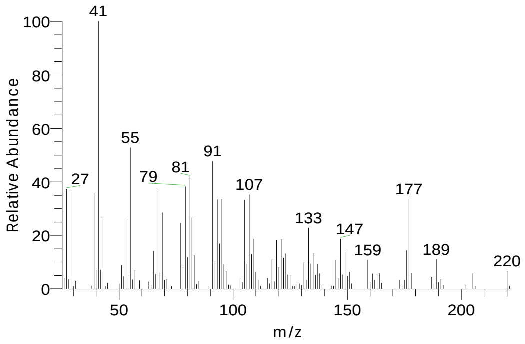

AROMADENDRENOXID-(2)
Formula C15H24O, MW 220, CAS# NA, Entry# 387063

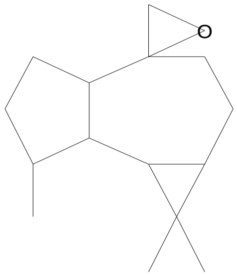

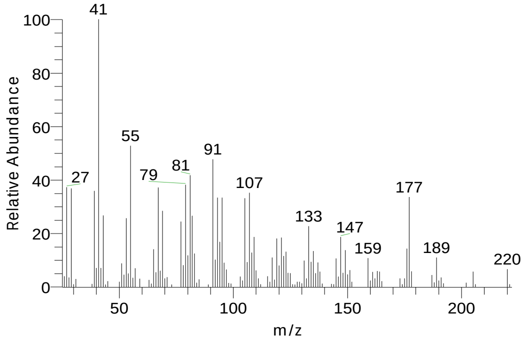

ALLOAROMADENDRENOXID-(1)
Formula C15H24O, MW 220, CAS# 85710-39-0, Entry# 107243
AROMADENDRENEPOXIDE-(I)

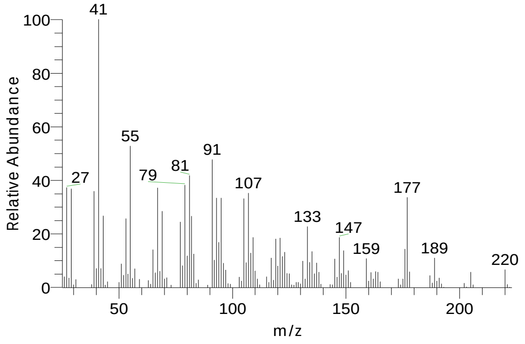

My GC-MS Report

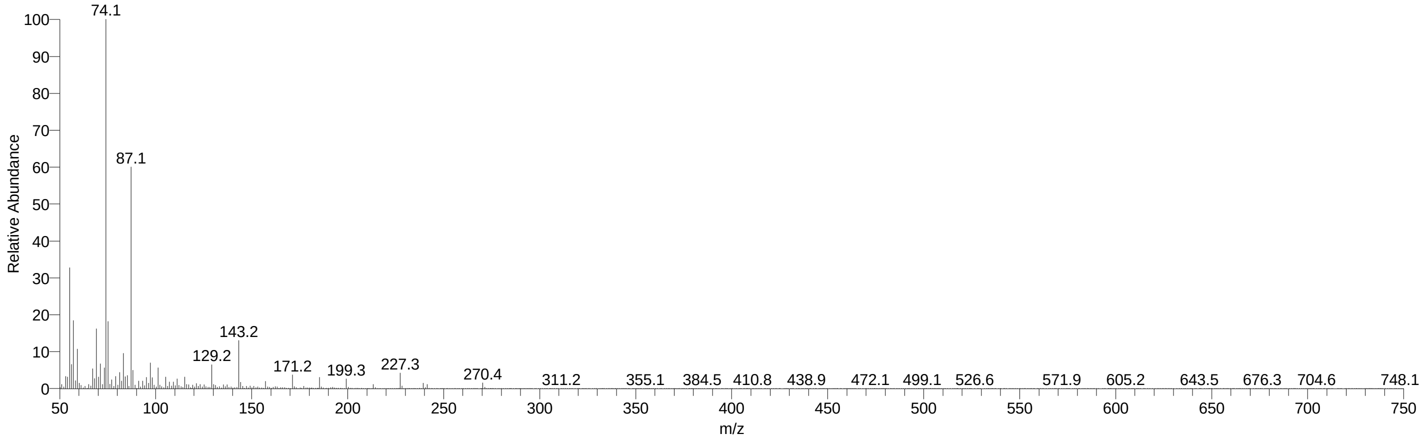

RT	Compound Name	Area %	MF	Molecular Formula	Molecular Weight	Cas #	Library
26.40	HEXADECANOIC ACID, METHYL ESTER	1.61	860	C17H34O2	270	112-39-0	WileyRegistry8e
26.40	HEXADECANOIC ACID, METHYL ESTER	1.61	905	C17H34O2	270	112-39-0	WileyRegistry8e
26.40	HEXADECANOIC ACID, METHYL ESTER	1.61	871	C17H34O2	270	112-39-0	WileyRegistry8e
26.40	Hexadecanoic acid, methyl ester	1.61	903	C17H34O2	270	112-39-0	replib
26.40	HEXADECANOIC ACID, METHYL ESTER	1.61	882	C17H34O2	270	112-39-0	WileyRegistry8e

Compound Structure | Hit Spectrum

HEXADECANOIC ACID, METHYL ESTER
Formula C17H34O2, MW 270, CAS# 112-39-0, Entry# 161288
METHYL HEXADECANOATE

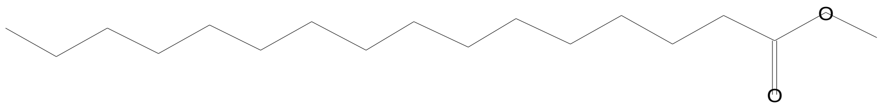

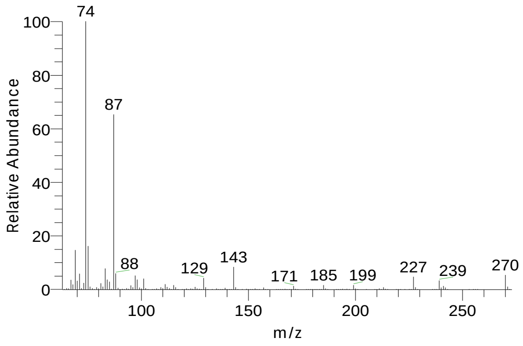

HEXADECANOIC ACID, METHYL ESTER
Formula C17H34O2, MW 270, CAS# 112-39-0, Entry# 161275
METHYL HEXADECANOATE

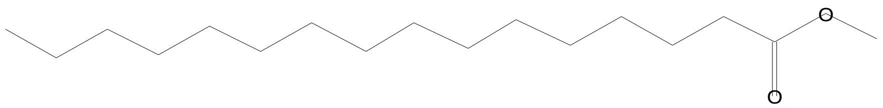

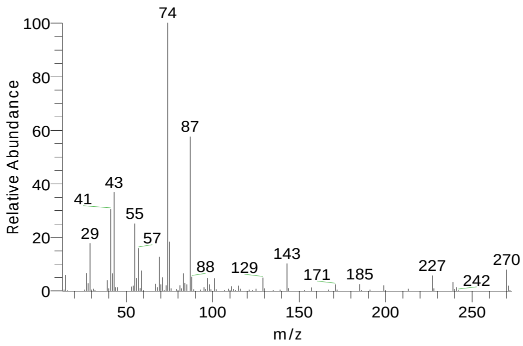

My GC-MS Report

Compound Structure | Hit Spectrum

HEXADECANOIC ACID, METHYL ESTER
Formula C17H34O2, MW 270, CAS# 112-39-0, Entry# 161274
METHYL HEXADECANOATE

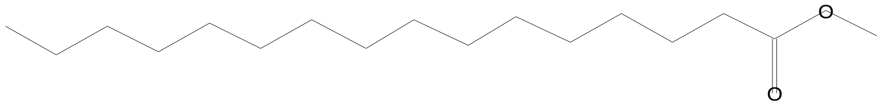

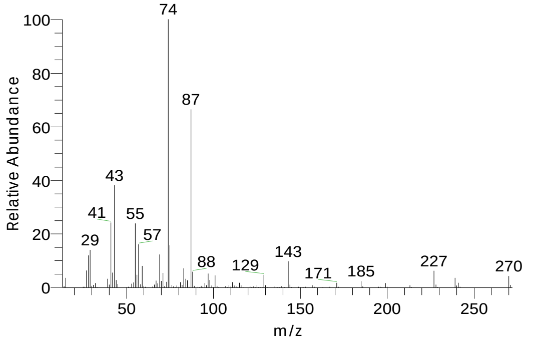

Hexadecanoic acid, methyl ester
Formula C17H34O2, MW 270, CAS# 112-39-0, Entry# 10412
Palmitic acid, methyl ester

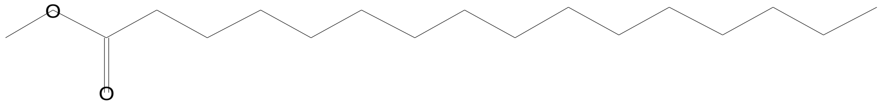

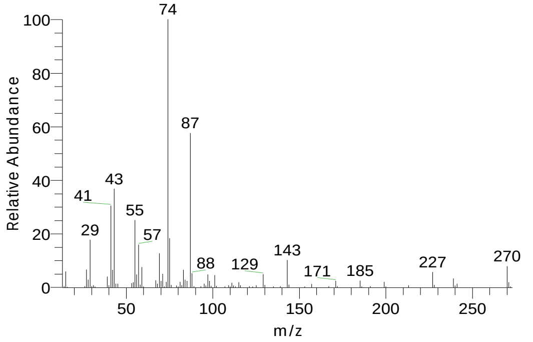

HEXADECANOIC ACID, METHYL ESTER
Formula C17H34O2, MW 270, CAS# 112-39-0, Entry# 161277
METHYL HEXADECANOATE

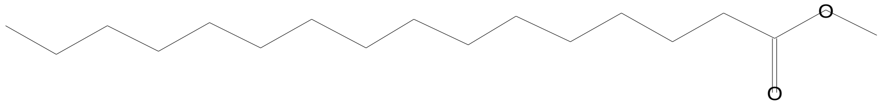

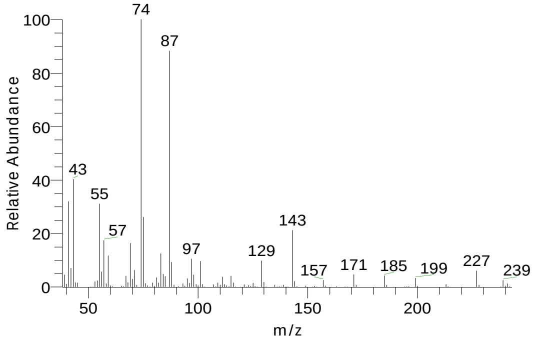

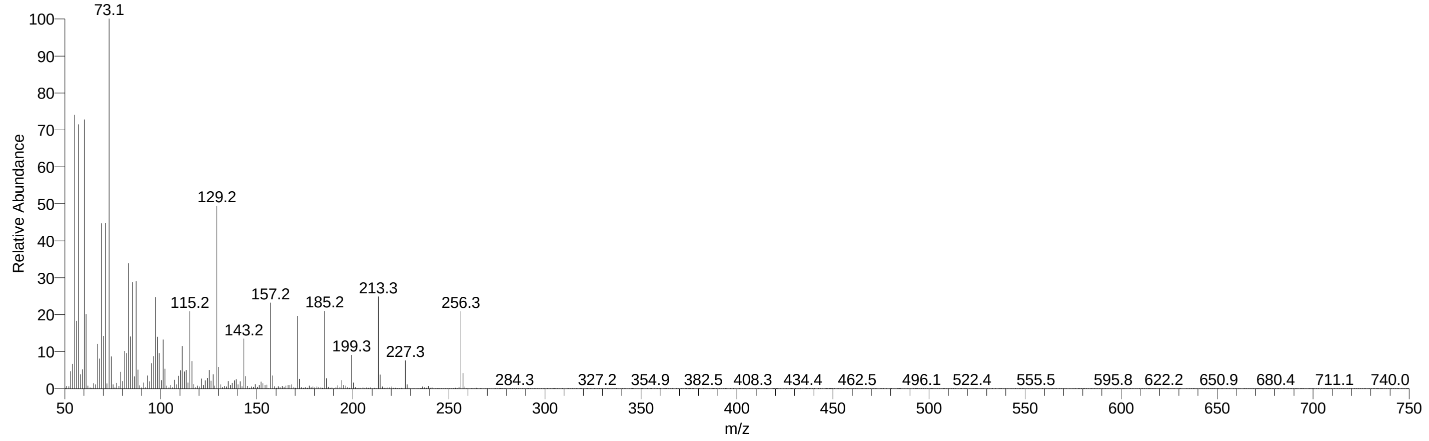

RT	Compound Name	Area %	MF	Molecular Formula	Molecular Weight	Cas #	Library
27.53	n-Hexadecanoic acid	19.33	925	C16H32O2	256	57-10-3	replib
27.53	n-Hexadecanoic acid	19.33	921	C16H32O2	256	57-10-3	replib
27.53	HEXADECANOIC ACID	19.33	911	C16H32O2	256	57-10-3	WileyRegistry8e

My GC-MS Report

RT	Compound Name	Area %	MF	Molecular Formula	Molecular Weight	Cas #	Library
27.53	HEXADECANOIC ACID	19.33	911	C16H32O2	256	57-10-3	WileyRegistry8e
27.53	n-Hexadecanoic acid	19.33	875	C16H32O2	256	57-10-3	replib

Compound Structure | Hit Spectrum

n-Hexadecanoic acid
Formula C16H32O2, MW 256, CAS# 57-10-3, Entry# 7566
Hexadecanoic acid

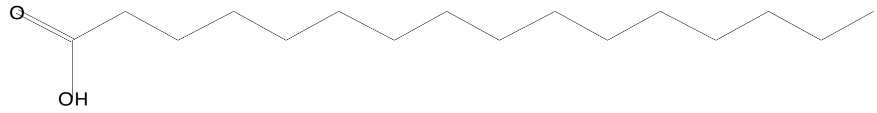

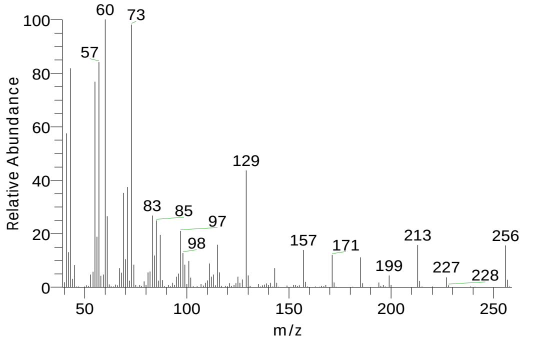

n-Hexadecanoic acid
Formula C16H32O2, MW 256, CAS# 57-10-3, Entry# 9622
Hexadecanoic acid

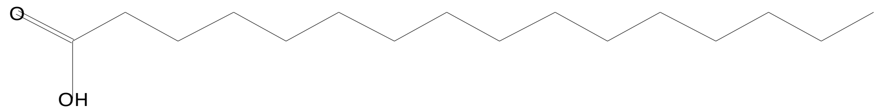

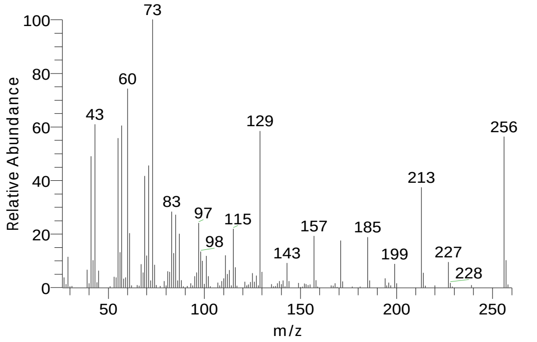

HEXADECANOIC ACID
Formula C16H32O2, MW 256, CAS# 57-10-3, Entry# 146744
HEXADECANOATE

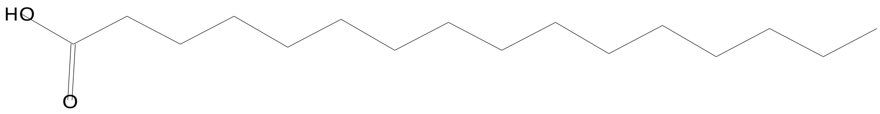

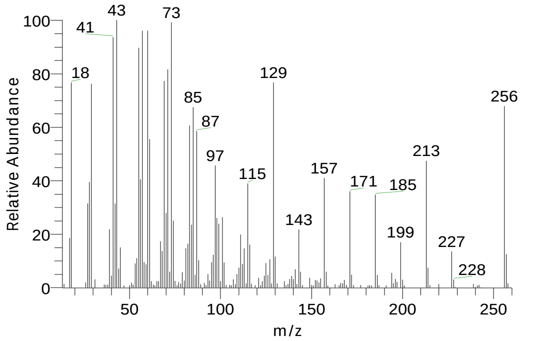

HEXADECANOIC ACID
Formula C16H32O2, MW 256, CAS# 57-10-3, Entry# 397116
HEXADECANOATE

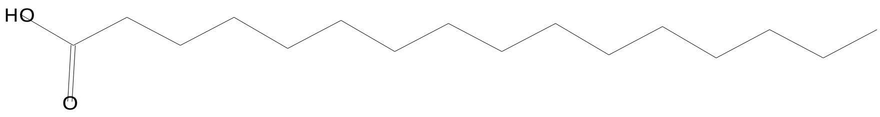

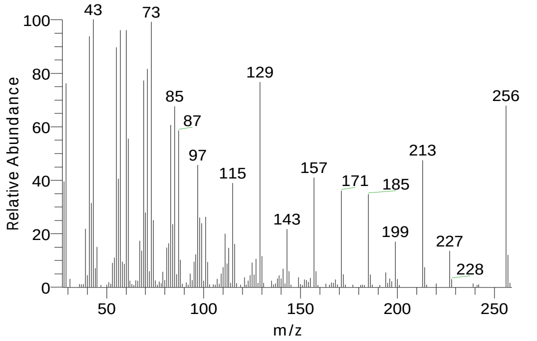

My GC-MS Report

Compound Structure

n-Hexadecanoic acid
Formula C16H32O2, MW 256, CAS# 57-10-3, Entry# 2779
Hexadecanoic acid

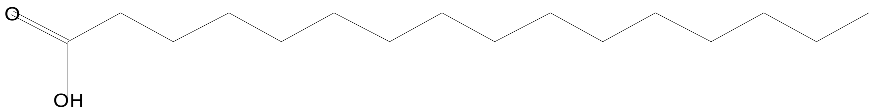

Hit Spectrum

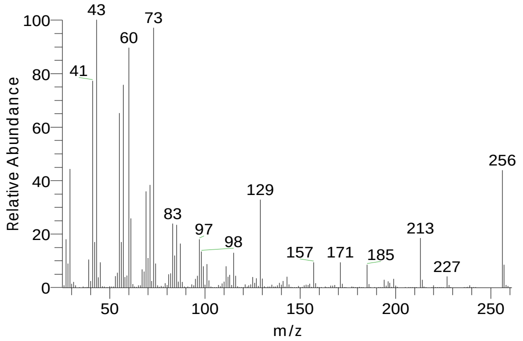

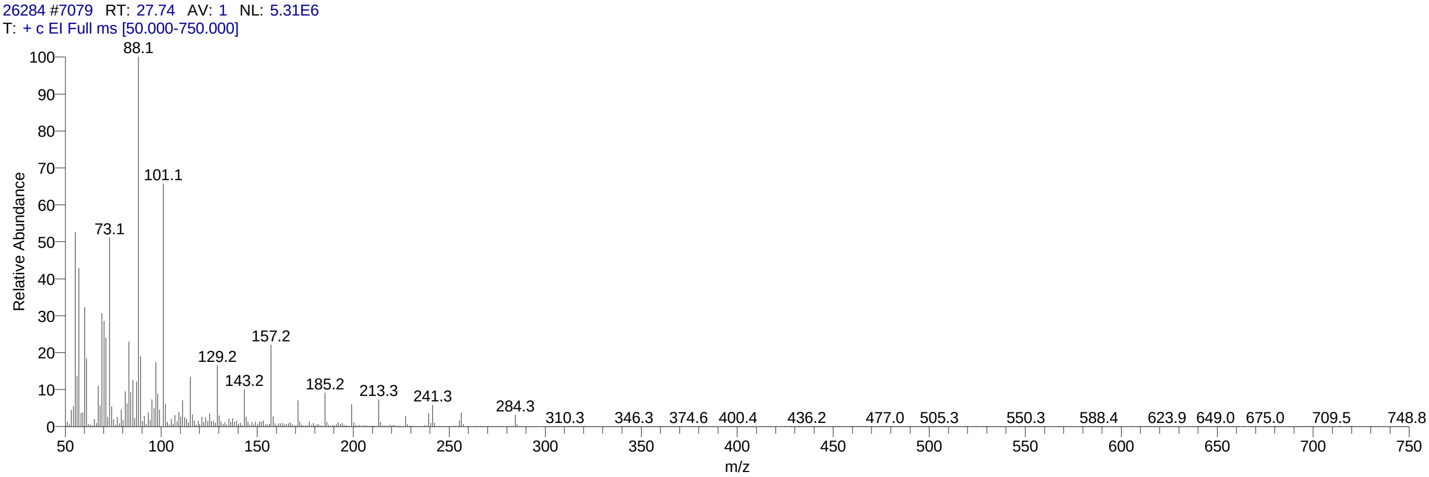

RT	Compound Name	Area %	MF	Molecular Formula	Molecular Weight	Cas #	Library
27.74	Hexadecanoic acid, ethyl ester	1.58	842	C18H36O2	284	628-97-7	mainlib
27.74	HEXADECANOIC ACID, ETHYL ESTER	1.58	858	C18H36O2	284	628-97-7	WileyRegistry8e
27.74	Hexadecanoic acid, ethyl ester	1.58	820	C18H36O2	284	628-97-7	replib
27.74	HEXADECANOIC ACID, ETHYL ESTER	1.58	821	C18H36O2	284	628-97-7	WileyRegistry8e
27.74	ETHYL PENTADECANOATE	1.58	860	C17H34O2	270	41114-00-5	WileyRegistry8e

Compound Structure

Hexadecanoic acid, ethyl ester
Formula C18H36O2, MW 284, CAS# 628-97-7, Entry# 57965
Palmitic acid, ethyl ester

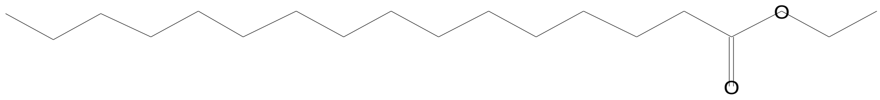

Hit Spectrum

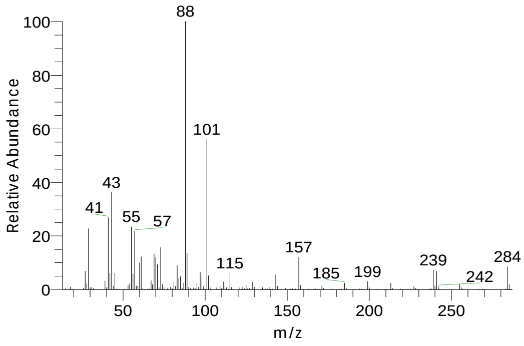

My GC-MS Report

Compound Structure | Hit Spectrum

HEXADECANOIC ACID, ETHYL ESTER
Formula C18H36O2, MW 284, CAS# 628-97-7, Entry# 174949
ETHYL HEXADECANOATE

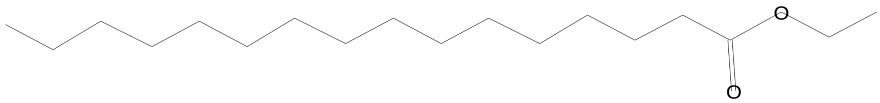

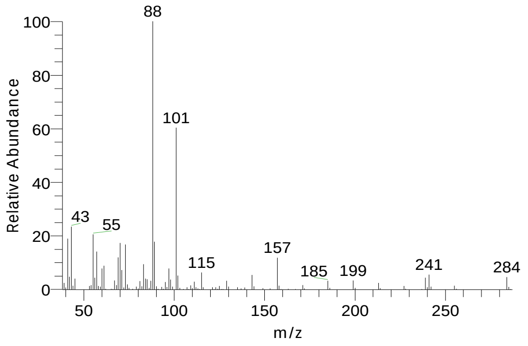

Hexadecanoic acid, ethyl ester
Formula C18H36O2, MW 284, CAS# 628-97-7, Entry# 12849
Palmitic acid, ethyl ester

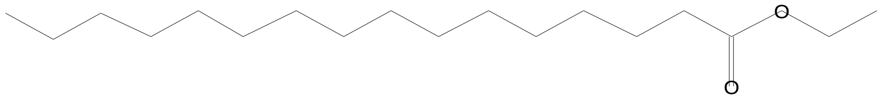

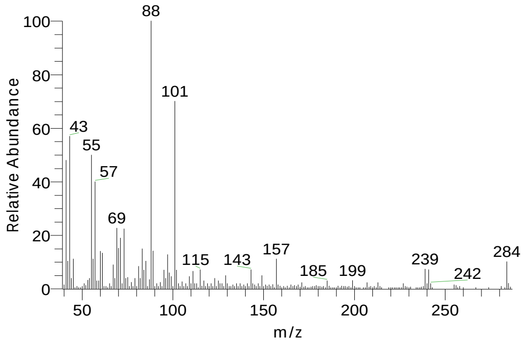

HEXADECANOIC ACID, ETHYL ESTER
Formula C18H36O2, MW 284, CAS# 628-97-7, Entry# 174956
ETHYL HEXADECANOATE

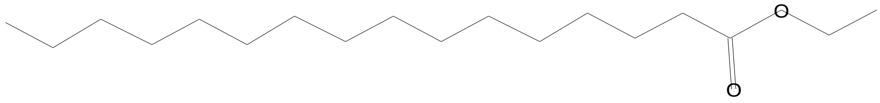

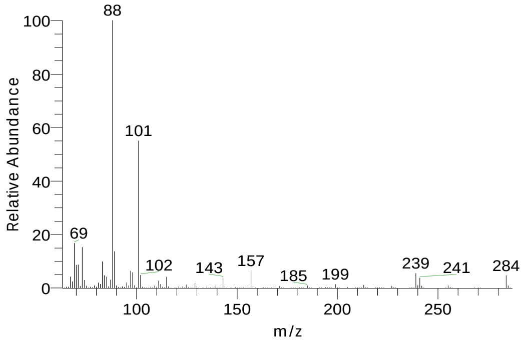

ETHYL PENTADECANOATE
Formula C17H34O2, MW 270, CAS# 41114-00-5, Entry# 161308
EINECS 255-223-8

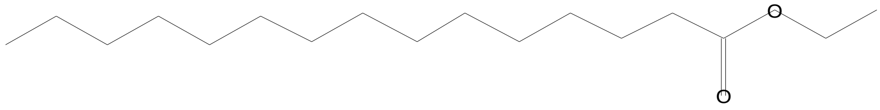

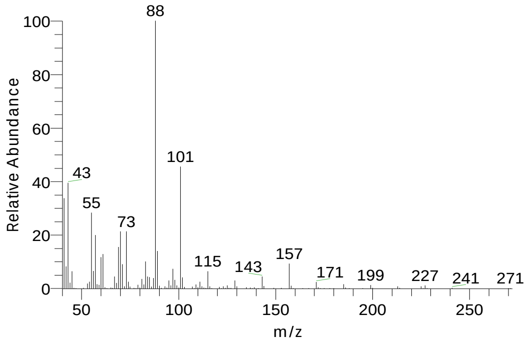

My GC-MS Report

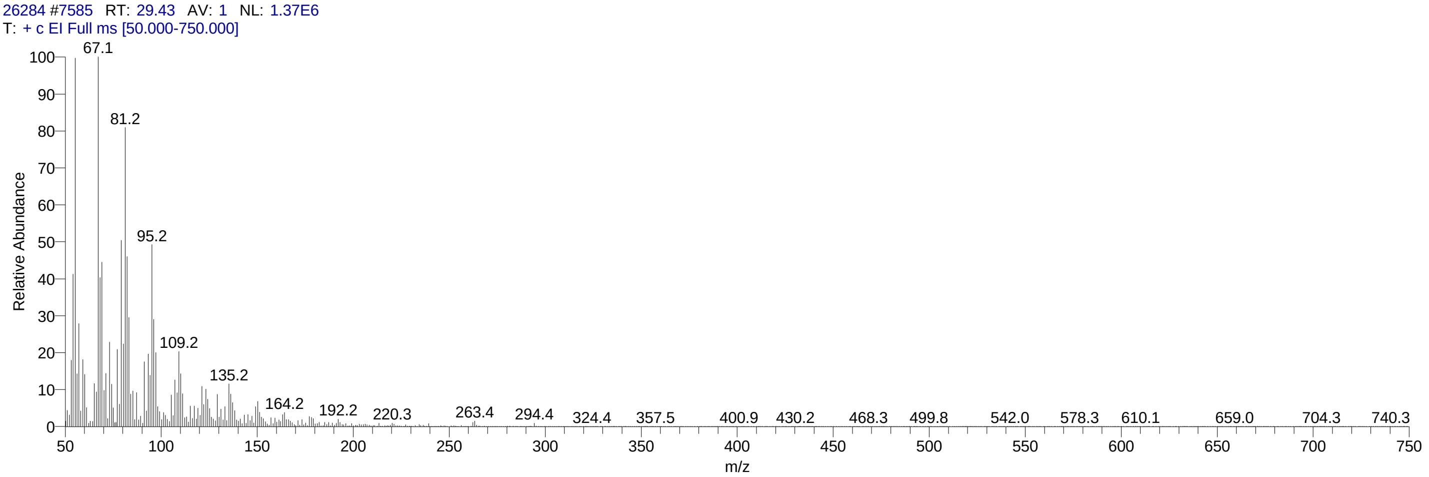

RT	Compound Name	Area %	MF	Molecular Formula	Molecular Weight	Cas #	Library
29.43	ETHYL (9Z,12Z)-9,12-OCTADECADIENOATE #	0.97	883	C20H36O2	308	544-35-4	WileyRegistry8e
29.43	Cyclopropaneoctanoic acid, 2-[[2-[(2-ethylcyclopropyl)methyl]cyclopropyl]methyl]-, methyl ester	0.97	862	C22H38O2	334	10152-71-3	mainlib
29.43	9,12-OCTADECADIENOIC ACID, METHYL ESTER, (E,E)-	0.97	844	C19H34O2	294	2566-97-4	WileyRegistry8e
29.43	CYCLOPROPANEOCTANOIC ACID, 2-[[2-[(2-ETHYLCYCLOPROPYL)METHYL]CYCLOPROPYL]METHYL]-, METHYL ESTER	0.97	861	C22H38O2	334	10152-71-3	WileyRegistry8e
29.43	9,12-OCTADECADIENOIC ACID (Z,Z)-, METHYL ESTER	0.97	849	C19H34O2	294	112-63-0	WileyRegistry8e

Compound Structure | Hit Spectrum

ETHYL (9Z,12Z)-9,12-OCTADECADIENOATE #
Formula C20H36O2, MW 308, CAS# 544-35-4, Entry# 196852
9,12-OCTADECADIENOIC ACID (9Z,12Z)-, ETHYL ESTER

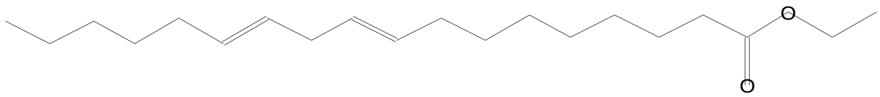

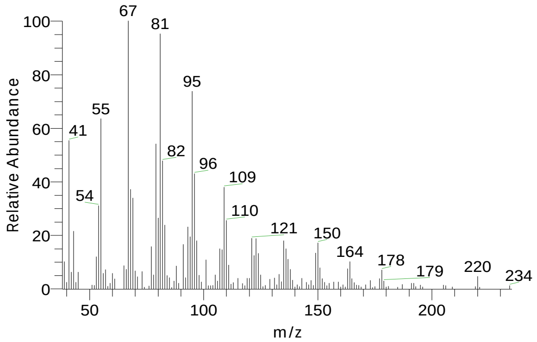

Formula C22H38O2, MW 334, CAS# 10152-71-3, Entry# 2765
Methyl 8-[2-((2-[(2-ethylcyclopropyl)methyl]cyclopropyl)methyl)cyclopropyl]octanoate #

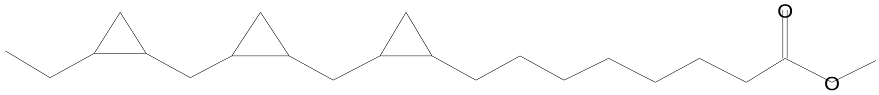

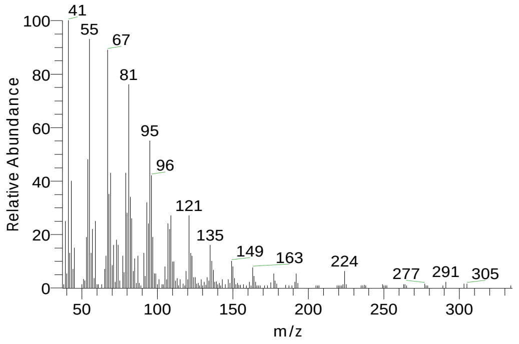

My GC-MS Report

Compound Structure | Hit Spectrum

9,12-OCTADECADIENOIC ACID, METHYL ESTER, (E,E)-
Formula C19H34O2, MW 294, CAS# 2566-97-4, Entry# 184250
METHYL (9E,12E)-9,12-OCTADECADIENOATE #

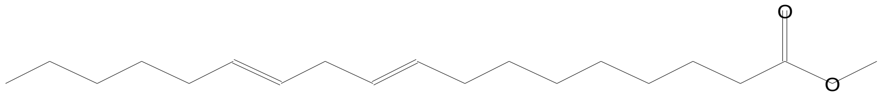

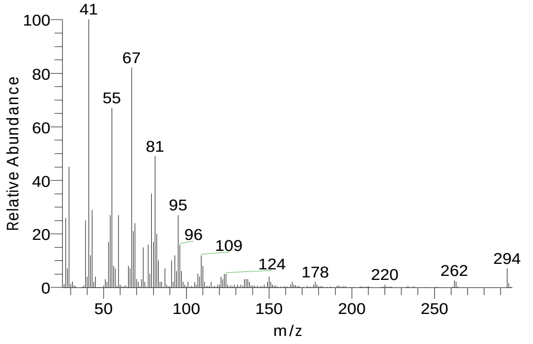

Formula C22H38O2, MW 334, CAS# 10152-71-3, Entry# 217974

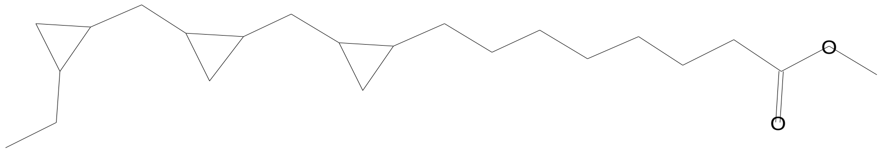

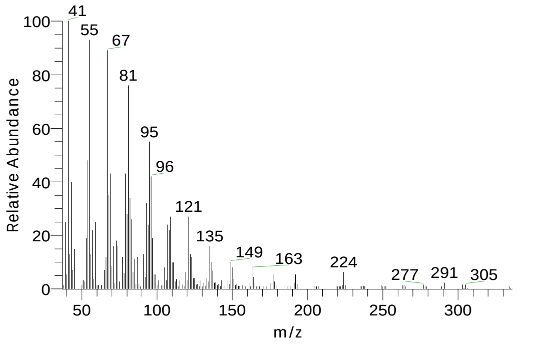

9,12-OCTADECADIENOIC ACID (Z,Z)-, METHYL ESTER
Formula C19H34O2, MW 294, CAS# 112-63-0, Entry# 184282
METHYL (9Z,12Z)-9,12-OCTADECADIENOATE #

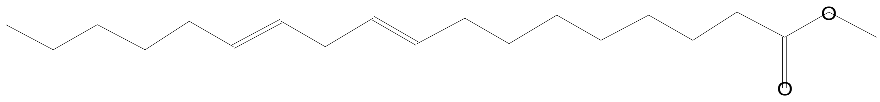

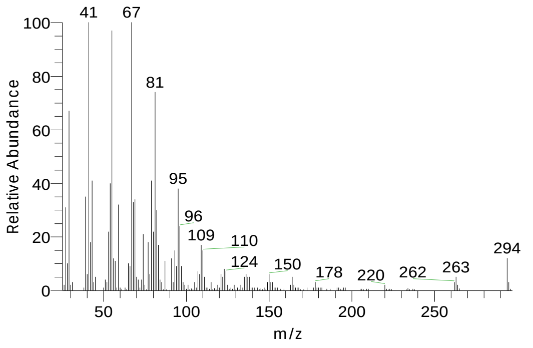

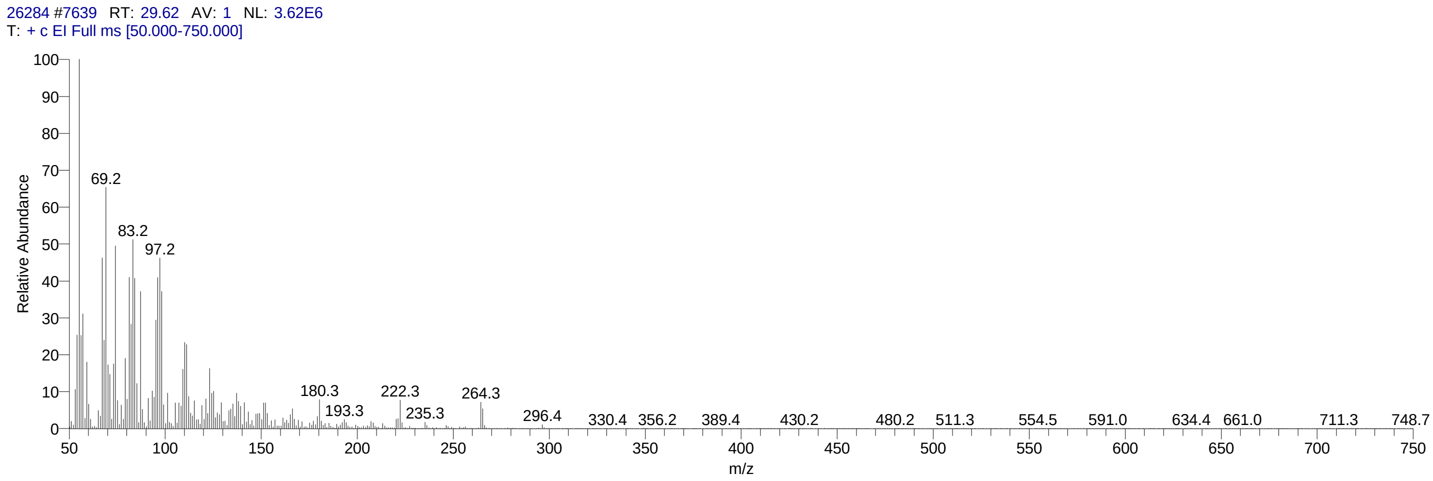

RT	Compound Name	Area %	MF	Molecular Formula	Molecular Weight	Cas #	Library
29.62	9-OCTADECENOIC ACID (Z)-, METHYL ESTER	2.45	935	C19H36O2	296	112-62-9	WileyRegistry8e
29.62	9-Octadecenoic acid (Z)-, methyl ester	2.45	933	C19H36O2	296	112-62-9	replib

My GC-MS Report

RT	Compound Name	Area %	MF	Molecular Formula	Molecular Weight	Cas #	Library
29.62	trans-13-Octadecenoic acid, methyl ester	2.45	897	C19H36O2	296	NA	mainlib
29.62	11-Octadecenoic acid, methyl ester	2.45	892	C19H36O2	296	52380-33-3	replib
29.62	9-Octadecenoic acid, methyl ester, (E)-	2.45	891	C19H36O2	296	1937-62-8	replib

Compound Structure | Hit Spectrum

9-OCTADECENOIC ACID (Z)-, METHYL ESTER
Formula C19H36O2, MW 296, CAS# 112-62-9, Entry# 186152
9-OCTADECENOIC ACID, METHYL ESTER

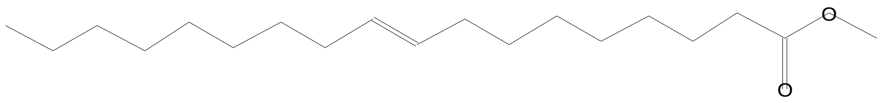

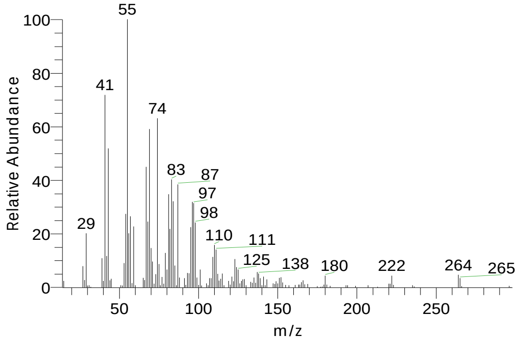

9-Octadecenoic acid (Z)-, methyl ester
Formula C19H36O2, MW 296, CAS# 112-62-9, Entry# 4747
Oleic acid, methyl ester

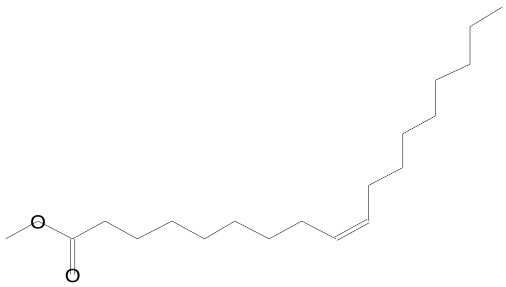

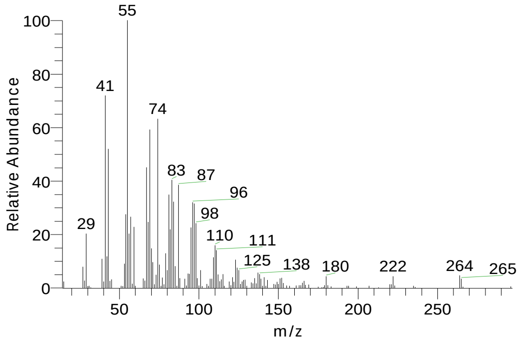

trans-13-Octadecenoic acid, methyl ester
Formula C19H36O2, MW 296, CAS# NA, Entry# 20067
$:28OPLQDSJPOHPOSZ-VOTSOKGWSA-N

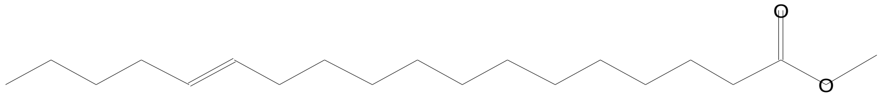

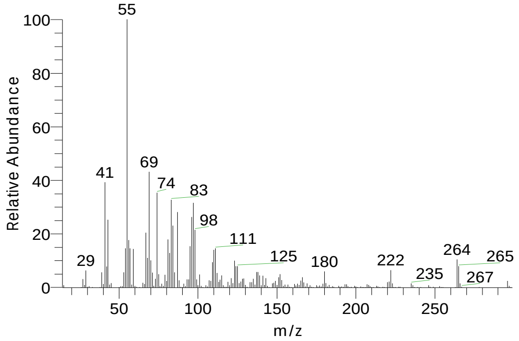

11-Octadecenoic acid, methyl ester
Formula C19H36O2, MW 296, CAS# 52380-33-3, Entry# 5005
Methyl 11-octadecenoate

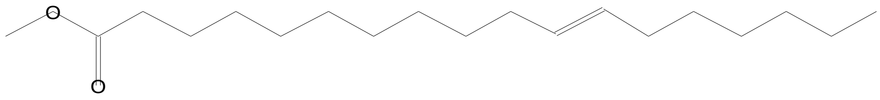

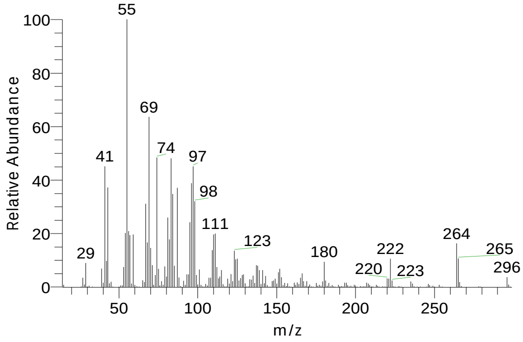

My GC-MS Report

Compound Structure | Hit Spectrum

9-Octadecenoic acid, methyl ester, (E)-
Formula C19H36O2, MW 296, CAS# 1937-62-8, Entry# 4987
Elaidic acid, methyl ester

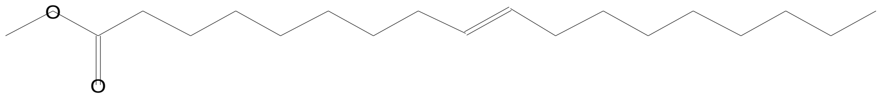

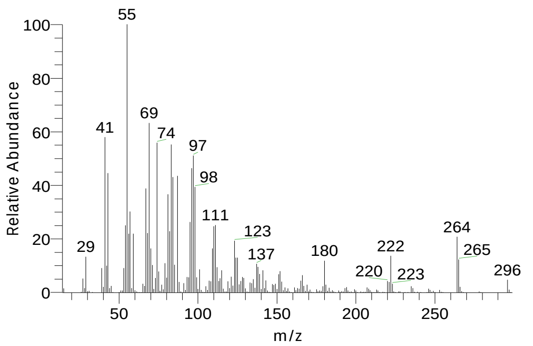

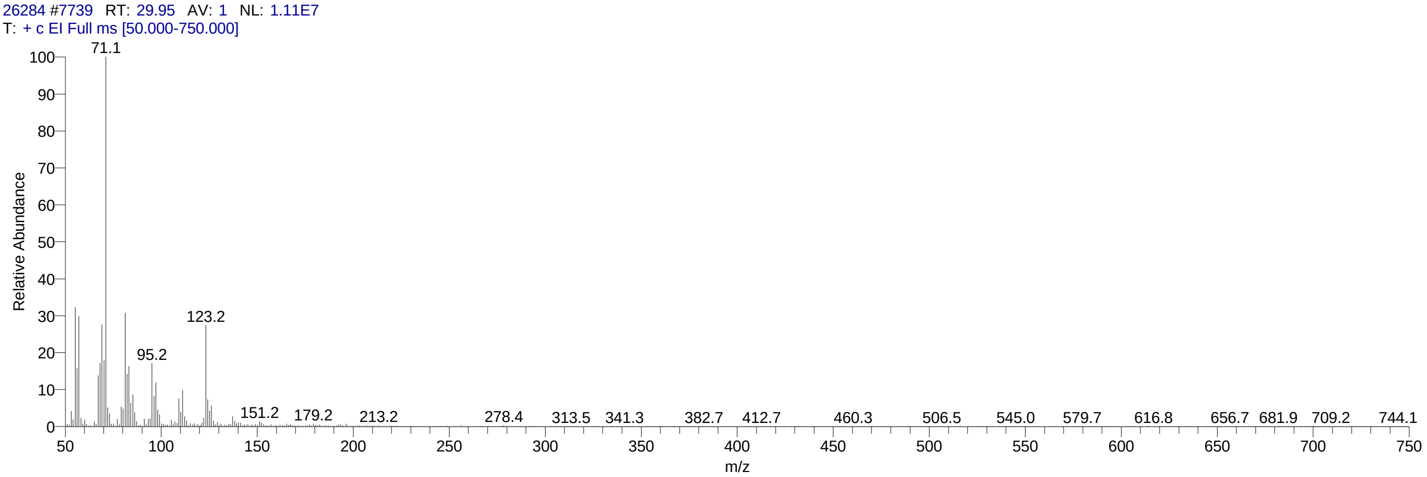

RT	Compound Name	Area %	MF	Molecular Formula	Molecular Weight	Cas #	Library
29.95	2-HEXADECEN-1-OL, 3,7,11,15-TETRAMETHYL-, [R-[R*,R*-(E)]]-	3.19	950	C20H40O	296	150-86-7	WileyRegistry8e
29.95	Phytol	3.19	906	C20H40O	296	150-86-7	mainlib
29.95	Phytol	3.19	898	C20H40O	296	150-86-7	replib
29.95	2-HEXADECEN-1-OL, 3,7,11,15-TETRAMETHYL-, [R-[R*,R*-(E)]]- (T-PHYTOL)	3.19	898	C20H40O	296	NA	WileyRegistry8e
29.95	2-HEXADECEN-1-OL, 3,7,11,15-TETRAMETHYL-, [R-[R*,R*-(E)]]-	3.19	925	C20H40O	296	150-86-7	WileyRegistry8e

Compound Structure | Hit Spectrum

2-HEXADECEN-1-OL, 3,7,11,15-TETRAMETHYL-, [R-[R*,R*-(E)]]-
Formula C20H40O, MW 296, CAS# 150-86-7, Entry# 186295
3,7,11,15-TETRAMETHYLHEXADEC-2-EN-1-OL

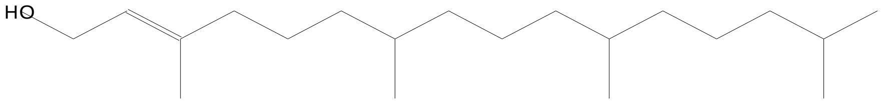

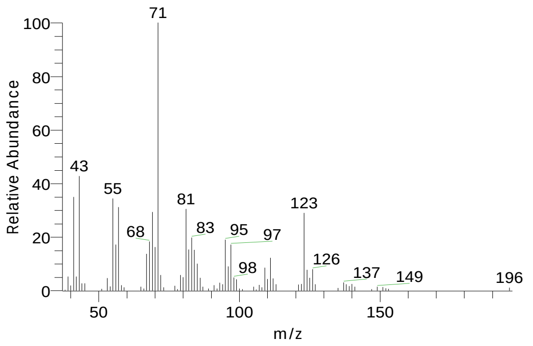

My GC-MS Report

Compound Structure | Hit Spectrum

Phytol
Formula C20H40O, MW 296, CAS# 150-86-7, Entry# 38432
2-Hexadecen-1-ol, 3,7,11,15-tetramethyl-, [R-[R*,R*-(E)]]-

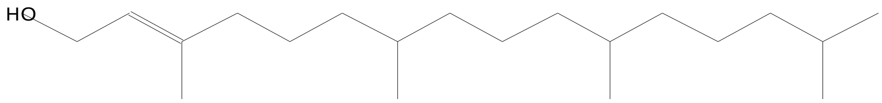

SI 886, RSI 906, mainlib, Entry# 38432, CAS# 150-86-7, Phytol

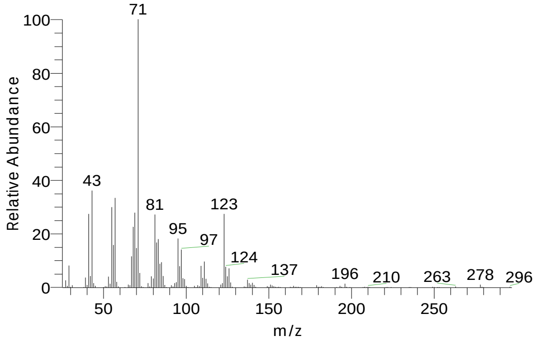

Phytol
Formula C20H40O, MW 296, CAS# 150-86-7, Entry# 9128
2-Hexadecen-1-ol, 3,7,11,15-tetramethyl-, [R-[R*,R*-(E)]]-

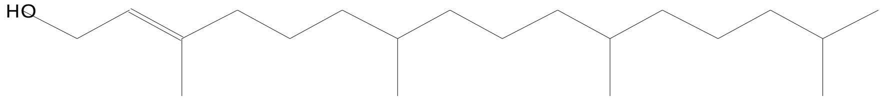

SI 877, RSI 898, replib, Entry# 9128, CAS# 150-86-7, Phytol

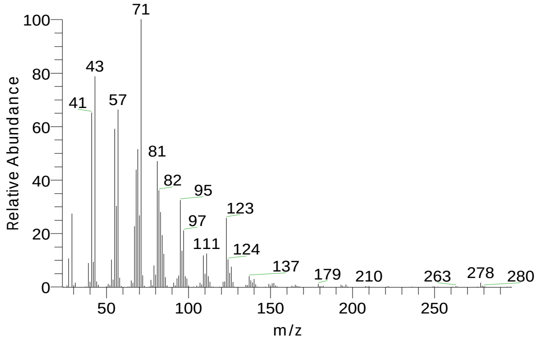

2-HEXADECEN-1-OL, 3,7,11,15-TETRAMETHYL-, [R-[R*,R*-(E)]]- (T-PHYTOL)
Formula C20H40O, MW 296, CAS# NA, Entry# 383453
3,7,11,15-TETRAMETHYL-2-HEXADECEN-1-OL

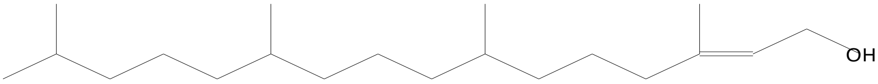

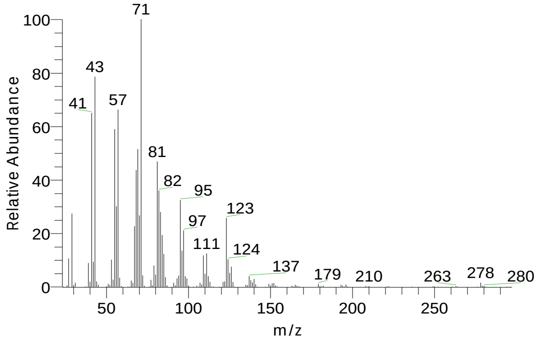

2-HEXADECEN-1-OL, 3,7,11,15-TETRAMETHYL-, [R-[R*,R*-(E)]]-
Formula C20H40O, MW 296, CAS# 150-86-7, Entry# 186297
3,7,11,15-TETRAMETHYLHEXADEC-2-EN-1-OL

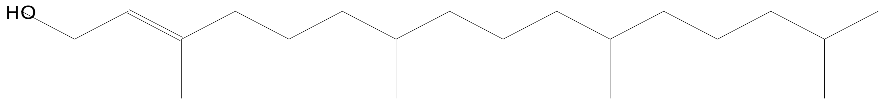

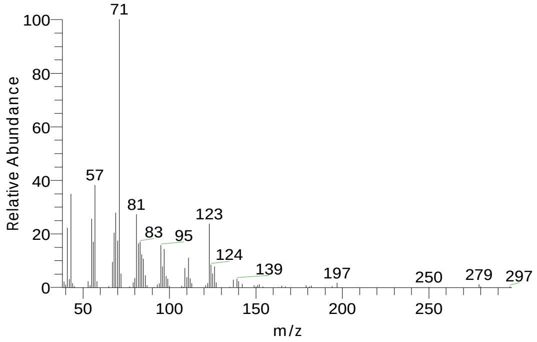

My GC-MS Report

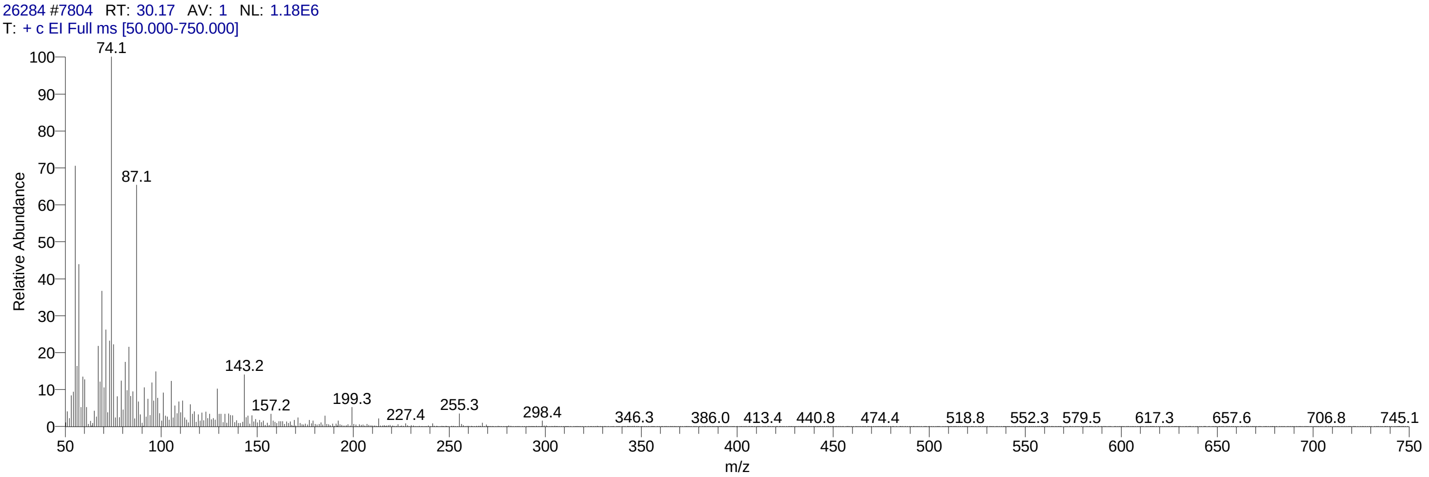

RT	Compound Name	Area %	MF	Molecular Formula	Molecular Weight	Cas #	Library
30.17	14-PENTADECYNOIC ACID, METHYL ESTER	0.32	822	C16H28O2	252	56909-04-7	WileyRegistry8e
30.17	CYCLOPENTANETRIDECANOIC ACID, METHYL ESTER	0.32	760	C19H36O2	296	24828-61-3	WileyRegistry8e
30.17	METHYL-9,9,10,10-D4-OCTADECANOATE	0.32	745	C19H34D4O2	302	56554-85-9	WileyRegistry8e
30.17	Cyclopropanepentanoic acid, 2-undecyl-, methyl ester, trans-	0.32	749	C20H38O2	310	42199-20-2	mainlib
30.17	CYCLOPROPANEPENTANOIC ACID, 2-UNDECYL-, METHYL ESTER, TRANS-	0.32	748	C20H38O2	310	42199-20-2	WileyRegistry8e

Compound Structure | Hit Spectrum

14-PENTADECYNOIC ACID, METHYL ESTER
Formula C16H28O2, MW 252, CAS# 56909-04-7, Entry# 142682
METHYL-PENTADEC-14-YNOATE

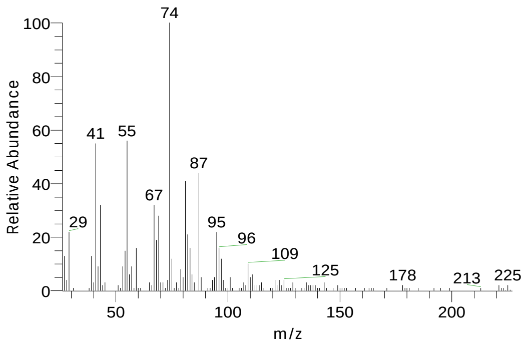

CYCLOPENTANETRIDECANOIC ACID, METHYL ESTER
Formula C19H36O2, MW 296, CAS# 24828-61-3, Entry# 186195
METHYL 13-CYCLOPENTYLTRIDECANOATE

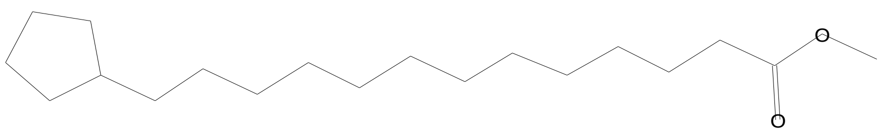

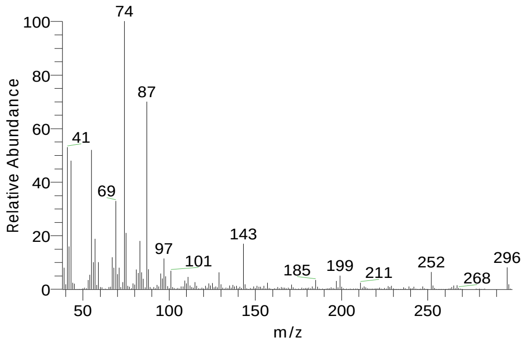

My GC-MS Report

Compound Structure	Hit Spectrum

METHYL-9,9,10,10-D4-OCTADECANOATE
Formula C19H34D4O2, MW 302, CAS# 56554-85-9, Entry# 187995

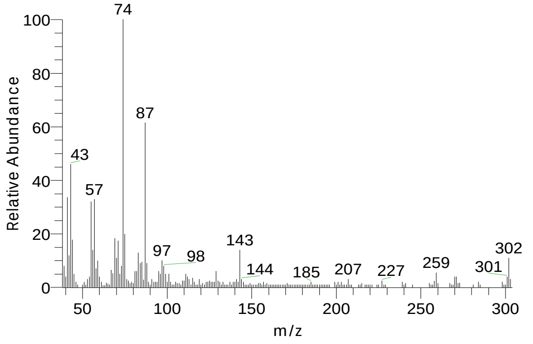

Cyclopropanepentanoic acid, 2-undecyl-, methyl ester, trans-
Formula C20H38O2, MW 310, CAS# 42199-20-2, Entry# 2425
Methyl 5-(2-undecylcyclopropyl)pentanoate, trans-

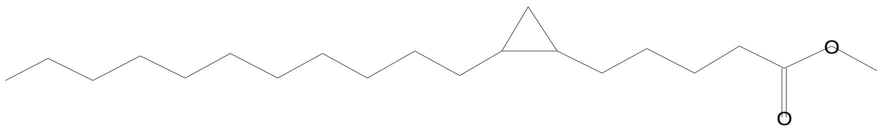

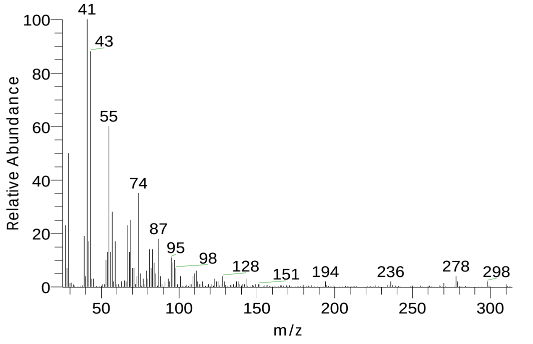

CYCLOPROPANEPENTANOIC ACID, 2-UNDECYL-, METHYL ESTER, TRANS-
Formula C20H38O2, MW 310, CAS# 42199-20-2, Entry# 198559
METHYL 5-(2-UNDECYLCYCLOPROPYL)PENTANOATE

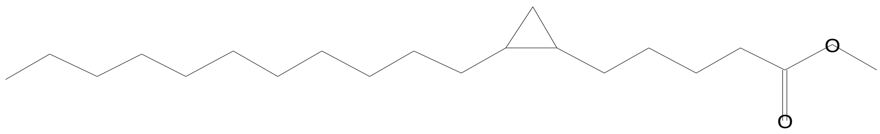

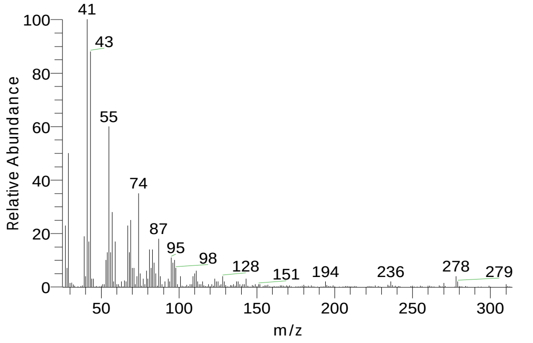

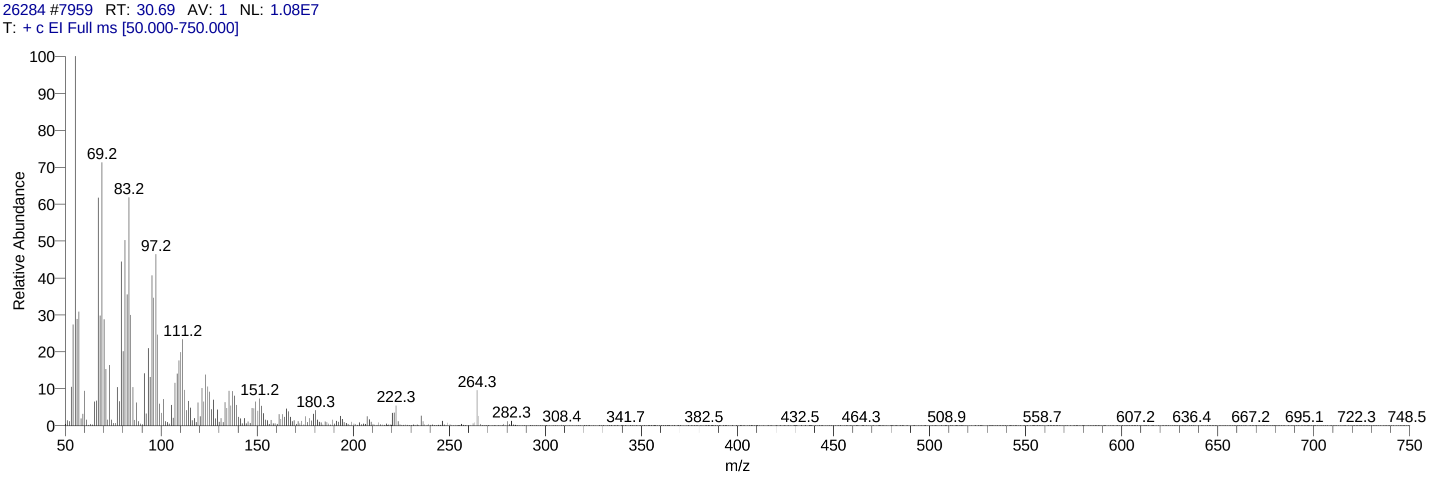

RT	Compound Name	Area %	MF	Molecular Formula	Molecular Weight	Cas #	Library
30.69	cis-Vaccenic acid	25.03	897	C18H34O2	282	506-17-2	mainlib
30.69	Oleic Acid	25.03	896	C18H34O2	282	112-80-1	replib

My GC-MS Report

RT	Compound Name	Area %	MF	Molecular Formula	Molecular Weight	Cas #	Library
30.69	9-OCTADECENOIC ACID (Z)-	25.03	869	C18H34O2	282	112-80-1	WileyRegistry8e
30.69	Oleic Acid	25.03	868	C18H34O2	282	112-80-1	replib
30.69	6-Octadecenoic acid	25.03	868	C18H34O2	282	NA	mainlib

Compound Structure

Hit Spectrum

cis-Vaccenic acid
Formula C18H34O2, MW 282, CAS# 506-17-2, Entry# 20090
11-Octadecenoic acid, (Z)-

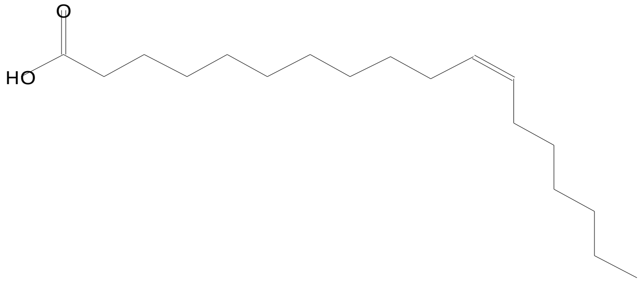

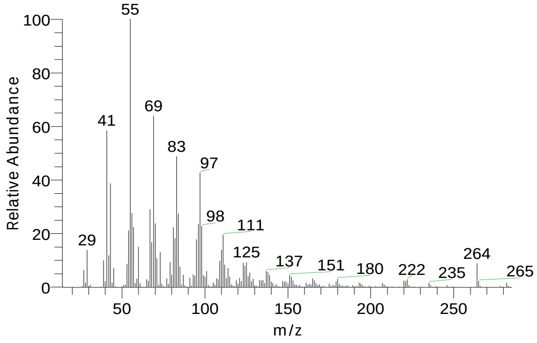

SI 885, RSI 896, replib, Entry# 5017, CAS# 112-80-1, Oleic Acid

Oleic Acid
Formula C18H34O2, MW 282, CAS# 112-80-1, Entry# 5017
9-Octadecenoic acid (Z)-

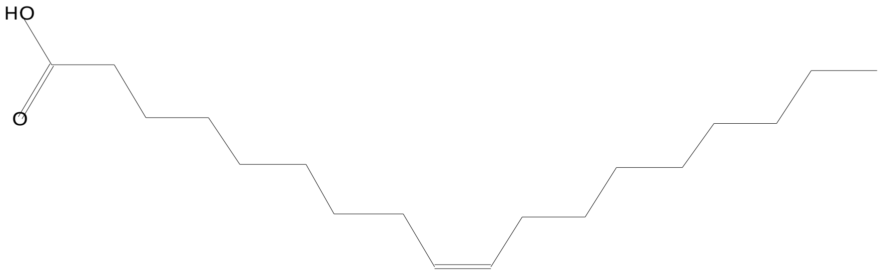

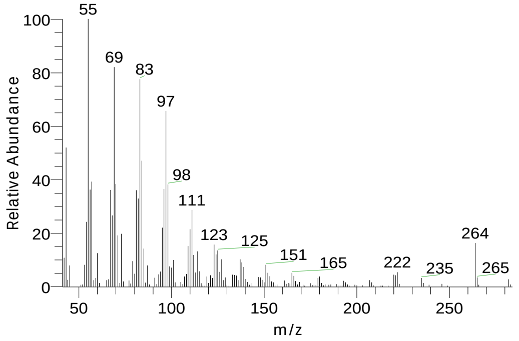

9-OCTADECENOIC ACID (Z)-
Formula C18H34O2, MW 282, CAS# 112-80-1, Entry# 172910
OCTADEC-9-ENOIC ACID

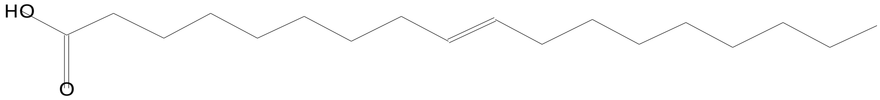

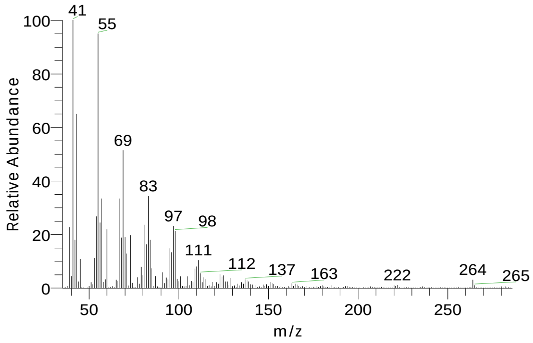

SI 868, RSI 868, replib, Entry# 4977, CAS# 112-80-1, Oleic Acid

Oleic Acid
Formula C18H34O2, MW 282, CAS# 112-80-1, Entry# 4977
9-Octadecenoic acid (Z)-

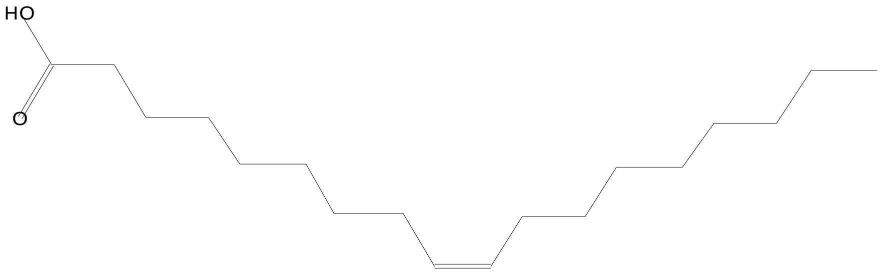

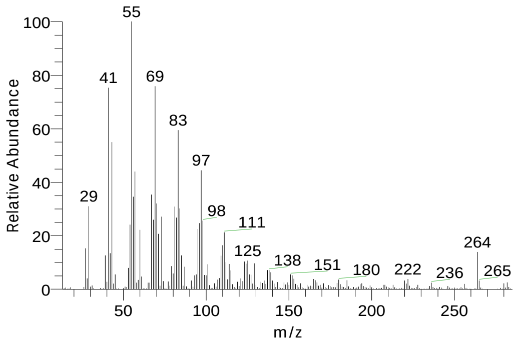

My GC-MS Report

Compound Structure

Hit Spectrum

6-Octadecenoic acid
Formula C18H34O2, MW 282, CAS# NA, Entry# 21117
$:28CNVZJPUDSLNTQU-SEYXRHQNSA-N

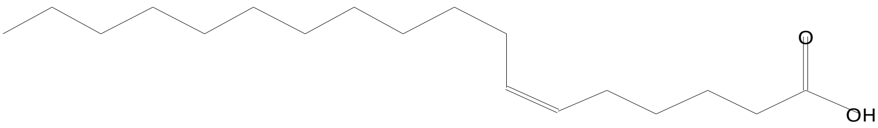

SI 859, RSI 868, mainlib, Entry# 21117, CAS# NA, 6-Octadecenoic acid

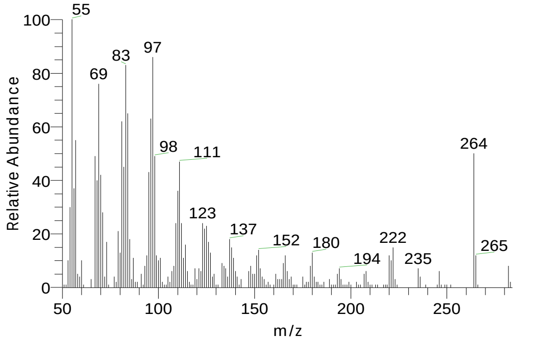

26284 #8003 RT: 30.84 AV: 1 NL: 3.46E6
T: + c EI Full ms [50.000-750.000]

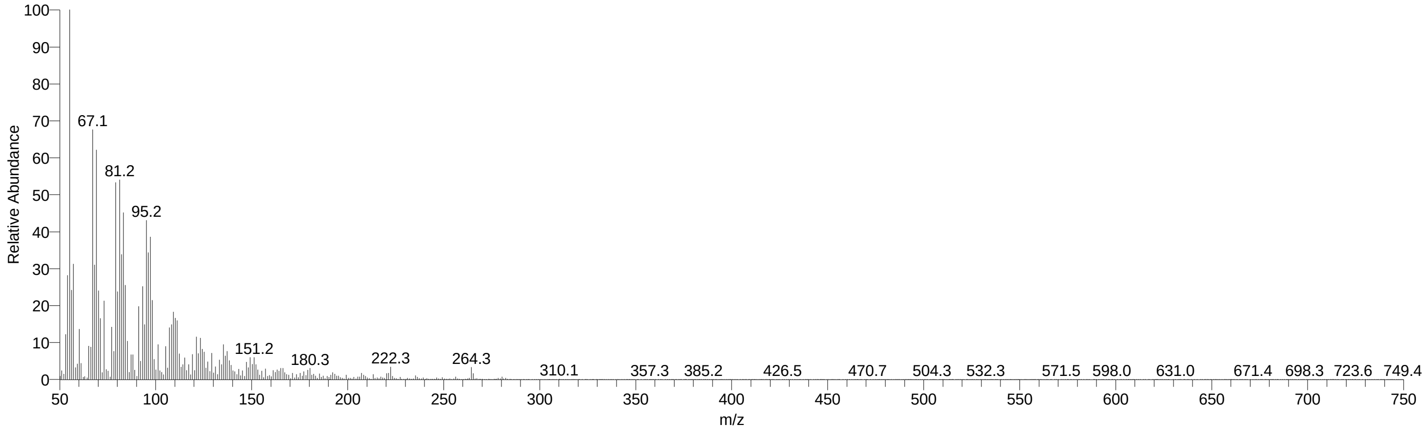

RT	Compound Name	Area %	MF	Molecular Formula	Molecular Weight	Cas #	Library
30.84	9-OCTADECENOIC ACID (Z)-	0.23	856	C18H34O2	282	112-80-1	WileyRegistry8e
30.84	9,12-Octadecadienoyl chloride, (Z,Z)-	0.23	861	C18H31ClO	298	7459-33-8	replib
30.84	(9E,12E)-9,12-OCTADECADIENOYL CHLORIDE #	0.23	861	C18H31ClO	298	7459-33-8	WileyRegistry8e
30.84	Z-(13,14-Epoxy)tetradec-11-en-1-ol acetate	0.23	831	C16H28O3	268	NA	mainlib
30.84	9,12-Octadecadienoic acid (Z,Z)-	0.23	834	C18H32O2	280	60-33-3	replib

Compound Structure

Hit Spectrum

9-OCTADECENOIC ACID (Z)-
Formula C18H34O2, MW 282, CAS# 112-80-1, Entry# 172910
OCTADEC-9-ENOIC ACID

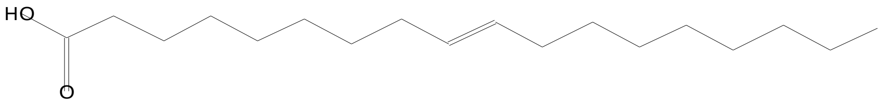

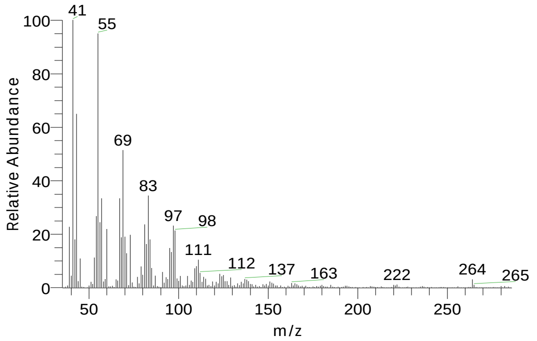

My GC-MS Report

Compound Structure | Hit Spectrum

9,12-Octadecadienoyl chloride, (Z,Z)-
Formula C18H31ClO, MW 298, CAS# 7459-33-8, Entry# 4940
Linoleoyl chloride

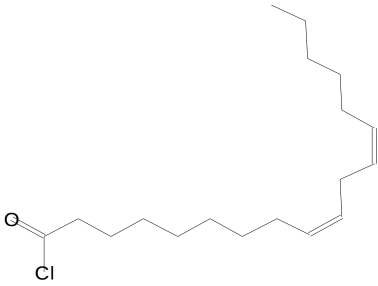

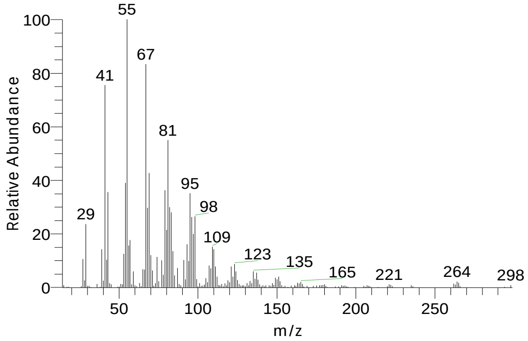

(9E,12E)-9,12-OCTADECADIENOYL CHLORIDE #
Formula C18H31ClO, MW 298, CAS# 7459-33-8, Entry# 187801
(9E,12E)-9,12-OCTADECADIENOYL CHLORIDE

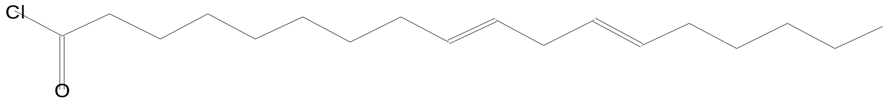

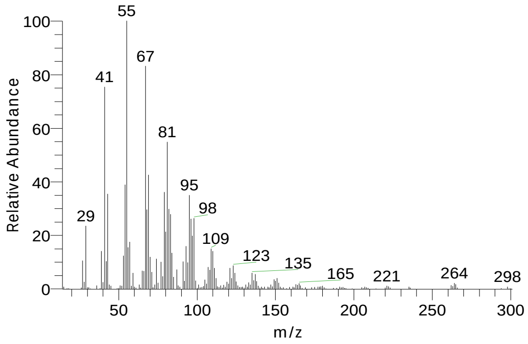

Z-(13,14-Epoxy)tetradec-11-en-1-ol acetate
Formula C16H28O3, MW 268, CAS# NA, Entry# 10459
(11Z)-12-(2-Oxiranyl)-11-dodecenyl acetate #

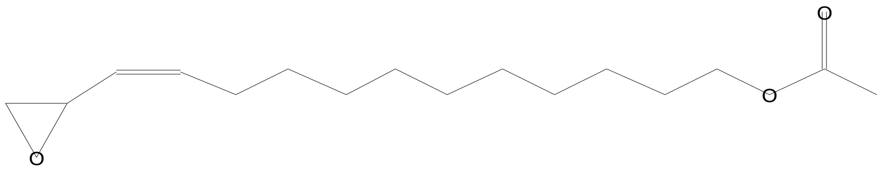

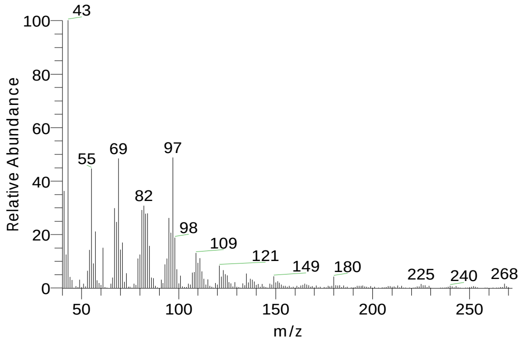

9,12-Octadecadienoic acid (Z,Z)-
Formula C18H32O2, MW 280, CAS# 60-33-3, Entry# 8057
cis-9,cis-12-Octadecadienoic acid

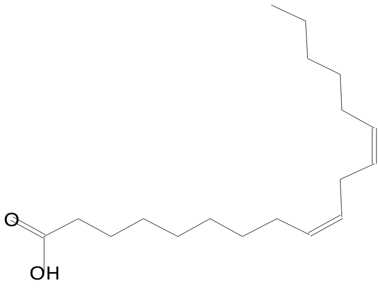

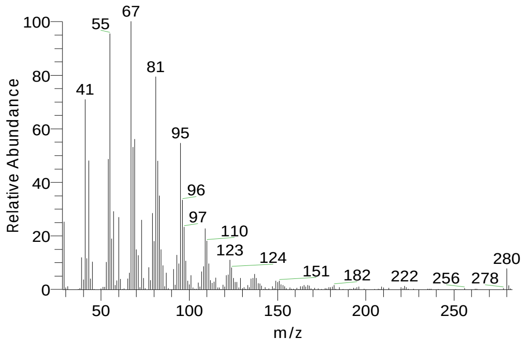

My GC-MS Report

26284 #8072 RT: 31.07 AV: 1 NL: 4.61E6
T: + c EI Full ms [50.000-750.000]

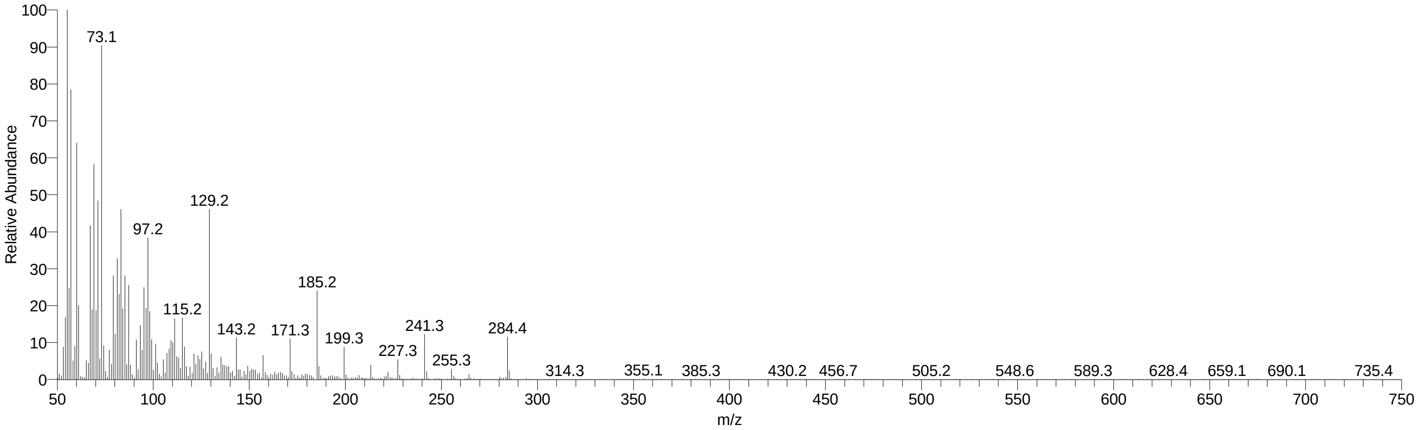

RT	Compound Name	Area %	MF	Molecular Formula	Molecular Weight	Cas #	Library
31.07	OCTADECANOIC ACID	3.80	845	C18H36O2	284	57-11-4	WileyRegistry8e
31.07	Octadecanoic acid	3.80	831	C18H36O2	284	57-11-4	mainlib
31.07	Octadecanoic acid	3.80	837	C18H36O2	284	57-11-4	replib
31.07	Octadecanoic acid	3.80	897	C18H36O2	284	57-11-4	replib
31.07	OCTADECANOIC ACID	3.80	831	C18H36O2	284	57-11-4	WileyRegistry8e

Compound Structure	Hit Spectrum

OCTADECANOIC ACID
Formula C18H36O2, MW 284, CAS# 57-11-4, Entry# 174897
STEARATE

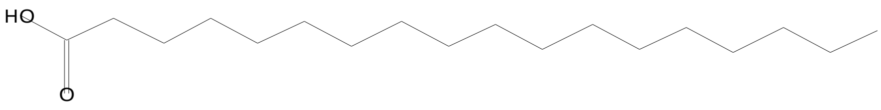

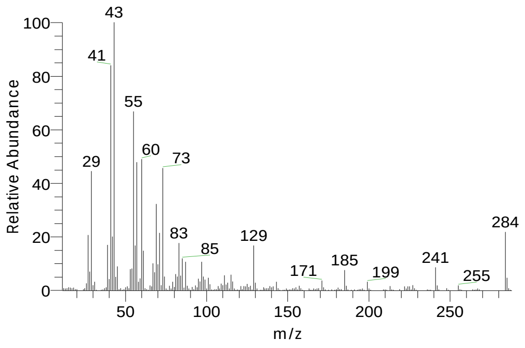

Octadecanoic acid
Formula C18H36O2, MW 284, CAS# 57-11-4, Entry# 9210
Stearic acid

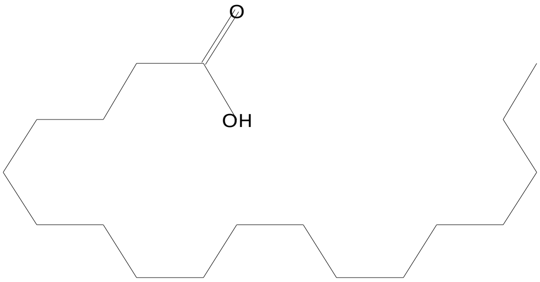

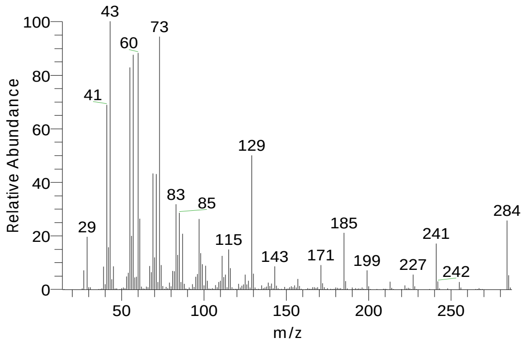

My GC-MS Report

Compound Structure | Hit Spectrum

Octadecanoic acid
Formula C18H36O2, MW 284, CAS# 57-11-4, Entry# 1866
Stearic acid

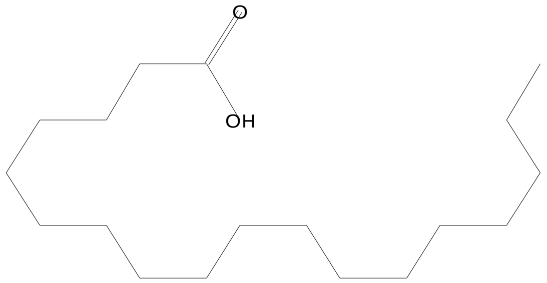

SI 806, RSI 837, replib, Entry# 1866, CAS# 57-11-4, Octadecanoic acid

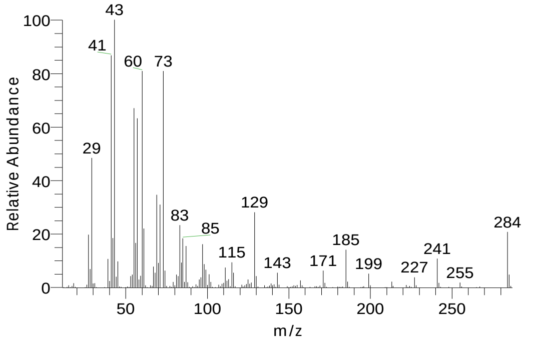

Octadecanoic acid
Formula C18H36O2, MW 284, CAS# 57-11-4, Entry# 2781
Stearic acid

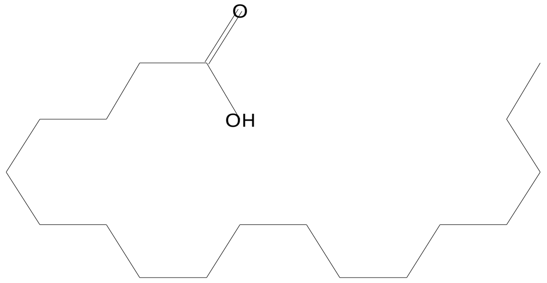

SI 801, RSI 897, replib, Entry# 2781, CAS# 57-11-4, Octadecanoic acid

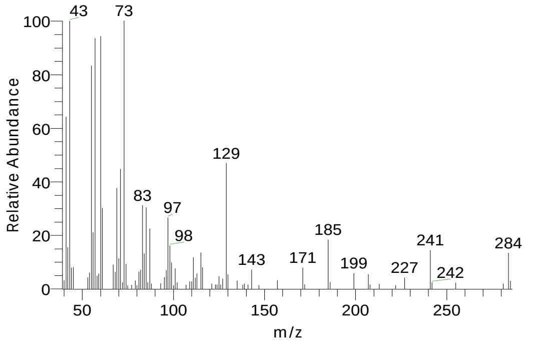

OCTADECANOIC ACID
Formula C18H36O2, MW 284, CAS# 57-11-4, Entry# 174902
STEARATE

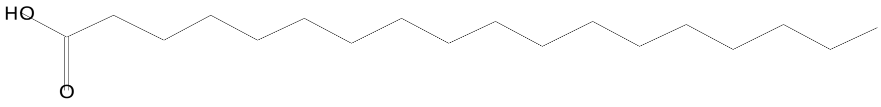

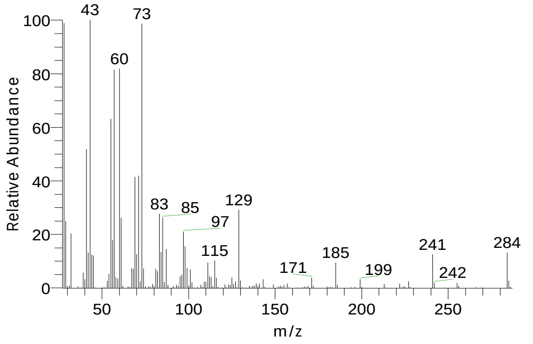

26284 #8165 RT: 31.38 AV: 1 NL: 1.77E6
T: + c EI Full ms [50.000-750.000]

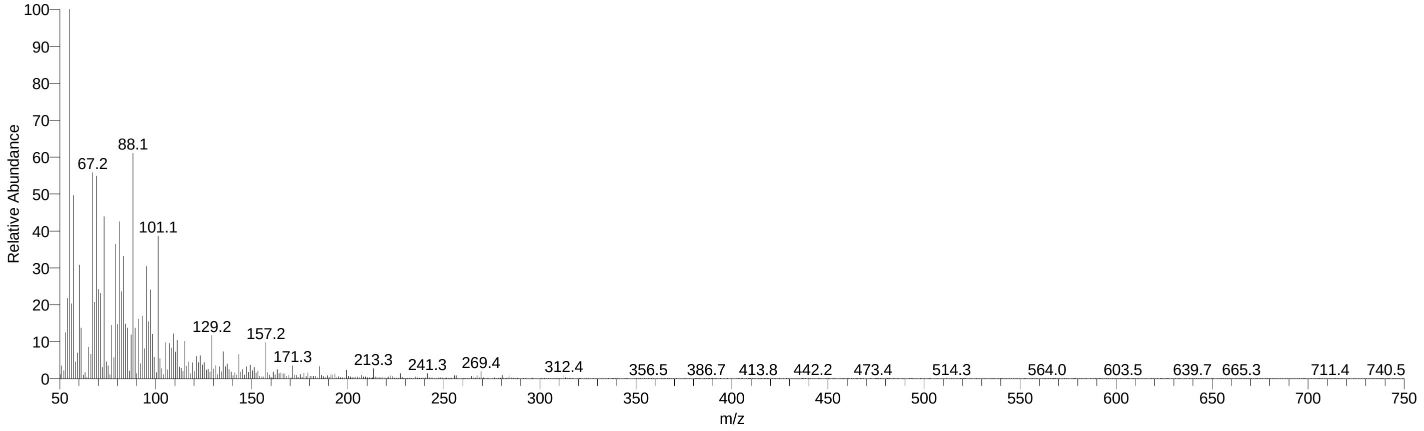

RT	Compound Name	Area %	MF	Molecular Formula	Molecular Weight	Cas #	Library
31.38	9-OCTADECENOIC ACID (Z)-	0.36	797	C18H34O2	282	112-80-1	WileyRegistry8e
31.38	OCTADECANOIC ACID, ETHYL ESTER	0.36	771	C20H40O2	312	111-61-5	WileyRegistry8e

My GC-MS Report

RT	Compound Name	Area %	MF	Molecular Formula	Molecular Weight	Cas #	Library
31.38	Hexadecanoic acid, ethyl ester	0.36	771	C18H36O2	284	628-97-7	replib
31.38	Oleic Acid	0.36	804	C18H34O2	282	112-80-1	replib
31.38	9-OCTADECENOIC ACID	0.36	804	C18H34O2	282	NA	WileyRegistry8e

Compound Structure | Hit Spectrum

9-OCTADECENOIC ACID (Z)-
Formula C18H34O2, MW 282, CAS# 112-80-1, Entry# 172910
OCTADEC-9-ENOIC ACID

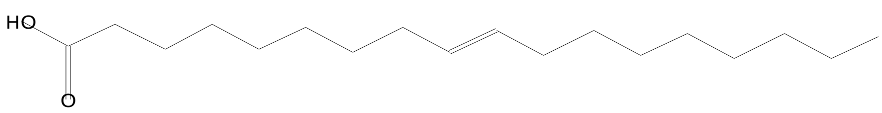

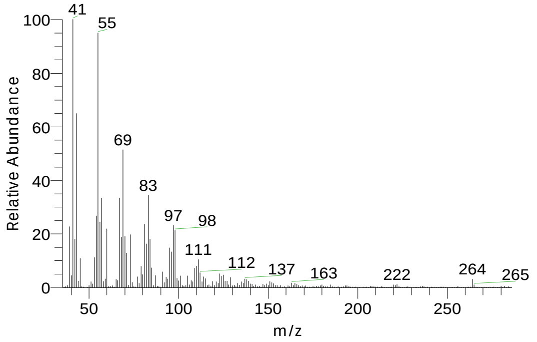

OCTADECANOIC ACID, ETHYL ESTER
Formula C20H40O2, MW 312, CAS# 111-61-5, Entry# 200367
ETHYL OCTADECANOATE

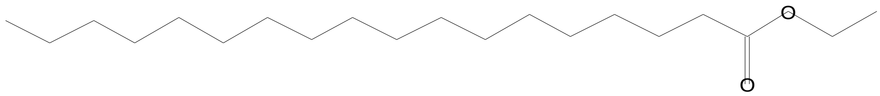

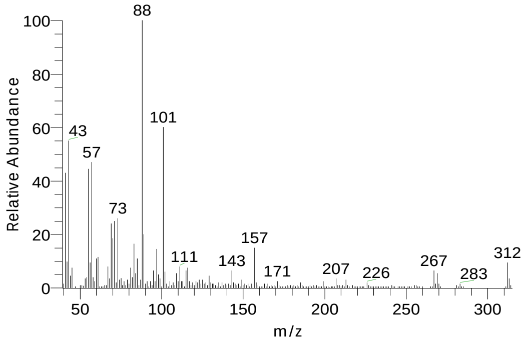

Hexadecanoic acid, ethyl ester
Formula C18H36O2, MW 284, CAS# 628-97-7, Entry# 12849
Palmitic acid, ethyl ester

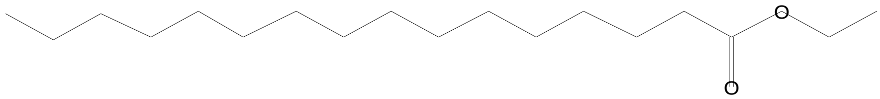

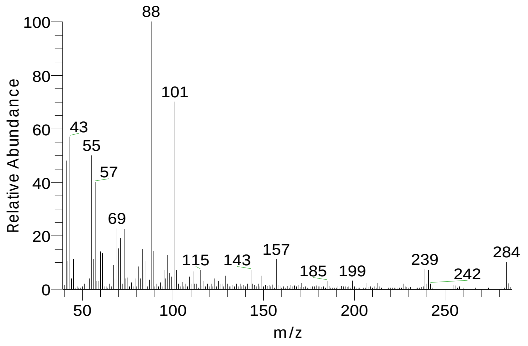

Oleic Acid
Formula C18H34O2, MW 282, CAS# 112-80-1, Entry# 4727
9-Octadecenoic acid (Z)-

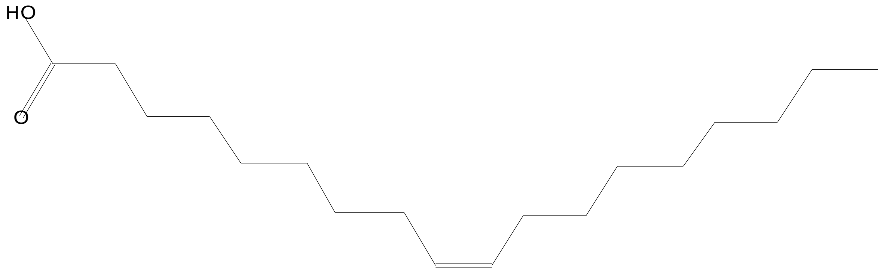

SI 761, RSI 804, replib, Entry# 4727, CAS# 112-80-1, Oleic Acid

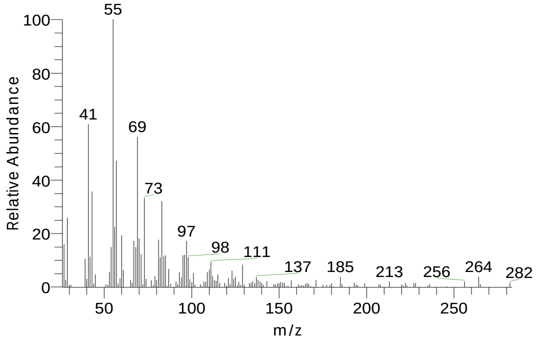

My GC-MS Report

Compound Structure

Hit Spectrum

9-OCTADECENOIC ACID
Formula C18H34O2, MW 282, CAS# NA, Entry# 384641
9-OCTADECENSAEURE, (Z)- (OELSAEURE)

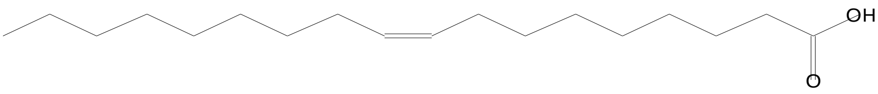

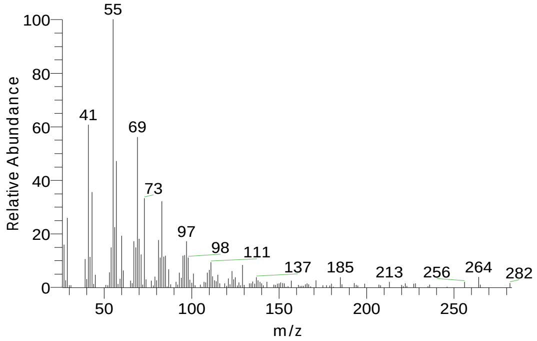

26284 #8610 RT: 32.87 AV: 1 NL: 1.81E6
T: + c EI Full ms [50.000-750.000]

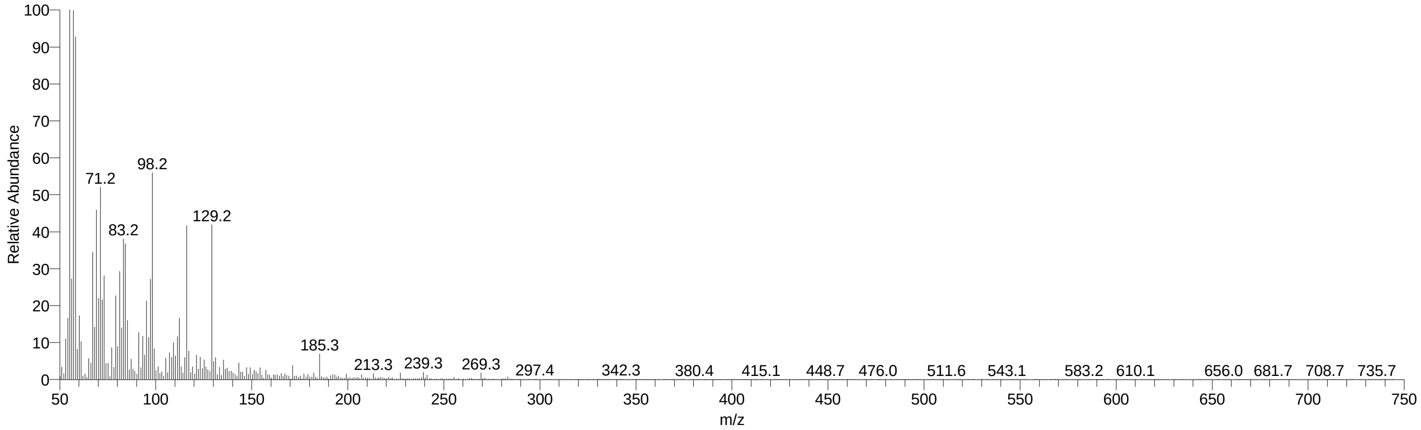

RT	Compound Name	Area %	MF	Molecular Formula	Molecular Weight	Cas #	Library
32.87	HEXADECANOIC ACID, 2,3-DIHYDROXYPROPYL ESTER	1.06	762	C19H38O4	330	542-44-9	WileyRegistry8e
32.87	2,2,3,3,4,4 HEXADEUTERO OCTADECANAL	1.06	733	C18H30D6O	274	56554-51-9	WileyRegistry8e
32.87	Oleic Acid	1.06	770	C18H34O2	282	112-80-1	replib
32.87	9-OCTADECENOIC ACID	1.06	770	C18H34O2	282	NA	WileyRegistry8e
32.87	2-Hexadecanol	1.06	738	C16H34O	242	14852-31-4	replib

Compound Structure

Hit Spectrum

HEXADECANOIC ACID, 2,3-DIHYDROXYPROPYL ESTER
Formula C19H38O4, MW 330, CAS# 542-44-9, Entry# 214589
2,3-DIHYDROXYPROPYL PALMITATE #

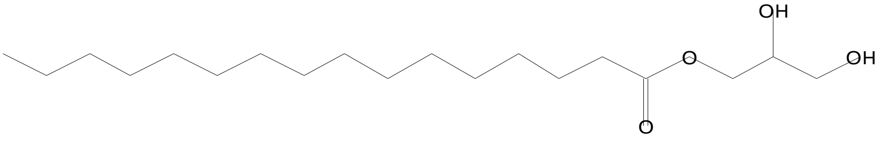

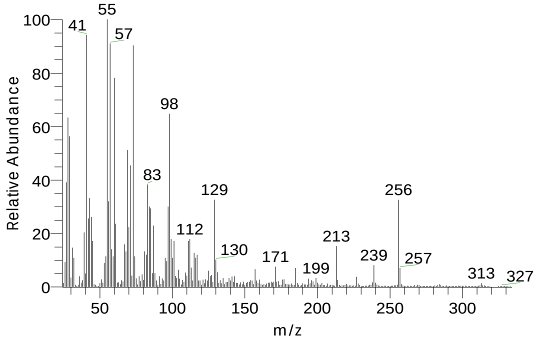

My GC-MS Report

Compound Structure | Hit Spectrum

2,2,3,3,4,4 HEXADEUTERO OCTADECANAL
Formula C18H30D6O, MW 274, CAS# 56554-51-9, Entry# 159360

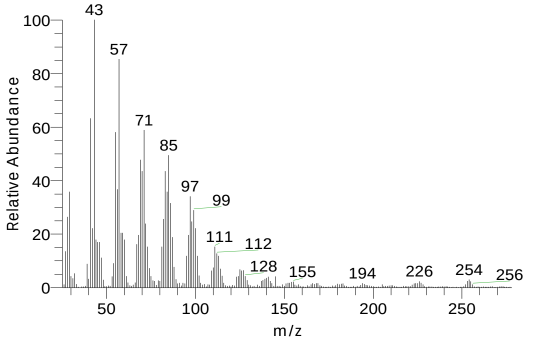

Oleic Acid
Formula C18H34O2, MW 282, CAS# 112-80-1, Entry# 4727
9-Octadecenoic acid (Z)-

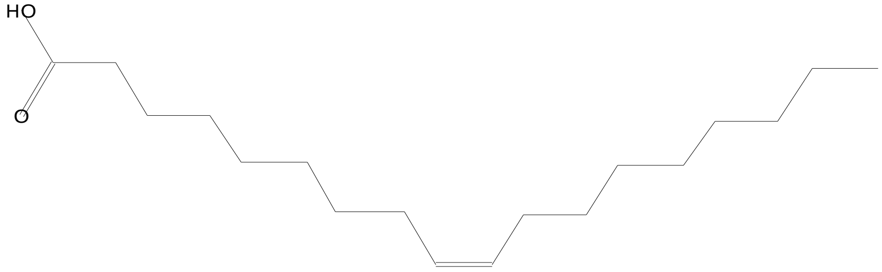

SI 730, RSI 770, replib, Entry# 4727, CAS# 112-80-1, Oleic Acid

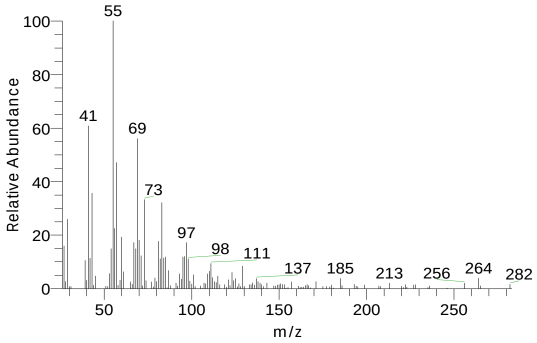

9-OCTADECENOIC ACID
Formula C18H34O2, MW 282, CAS# NA, Entry# 384641
9-OCTADECENSAEURE, (Z)- (OELSAEURE)

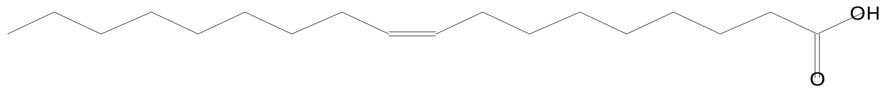

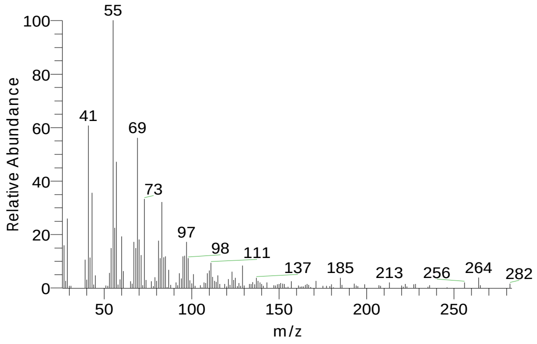

2-Hexadecanol
Formula C16H34O, MW 242, CAS# 14852-31-4, Entry# 4015
Hexadecanol-2

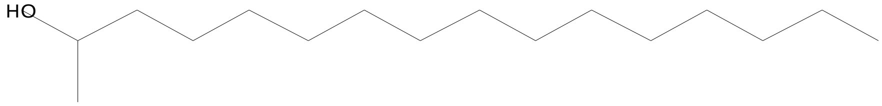

SI 730, RSI 738, replib, Entry# 4015, CAS# 14852-31-4, 2-Hexadecanol

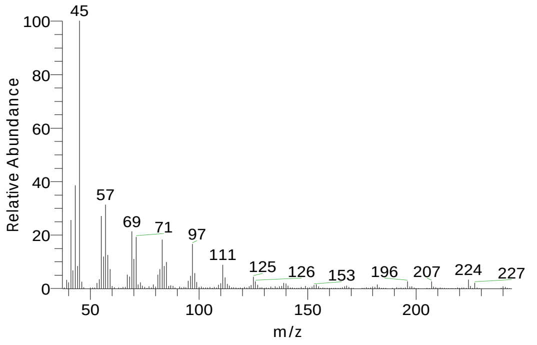

My GC-MS Report

26284 #9409 RT: 35.55 AV: 1 NL: 1.41E6
T: + c EI Full ms [50.000-750.000]

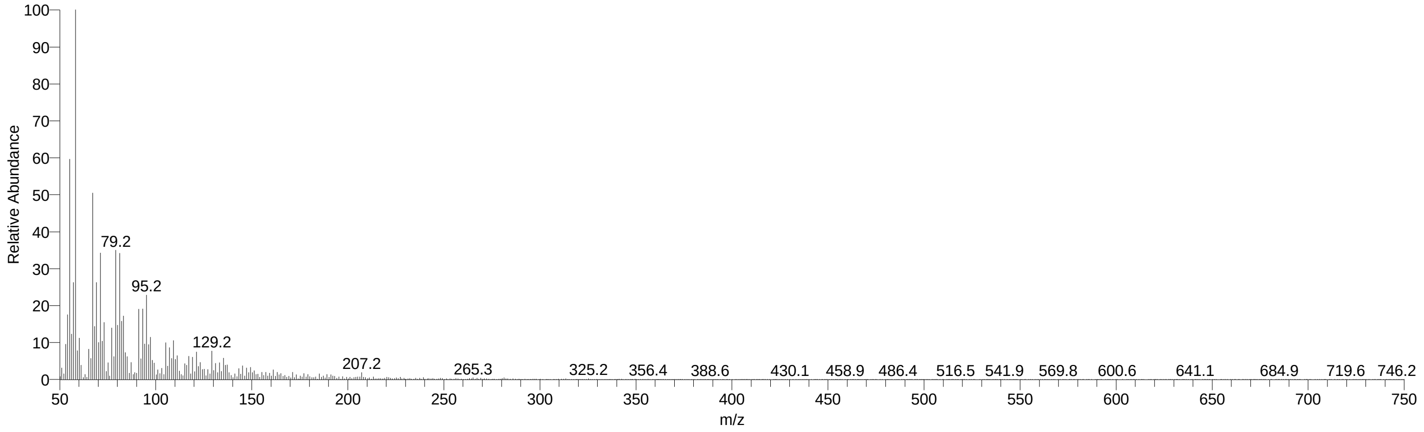

RT	Compound Name	Area %	MF	Molecular Formula	Molecular Weight	Cas #	Library
35.55	Cholestan-3-ol, 2-methylene-, (3á,5à)-	0.32	850	$C_{28}H_{48}O$	400	22599-96-8	mainlib
35.55	CHOLESTAN-3-OL, 2-METHYLENE-, (3á,5à)-	0.32	849	$C_{28}H_{48}O$	400	22599-96-8	WileyRegistry8e
35.55	8,11,14-Eicosatrienoic acid, (Z,Z,Z)-	0.32	827	$C_{20}H_{34}O_2$	306	1783-84-2	replib
35.55	8,11,14-EICOSATRIENOIC ACID, (Z,Z,Z)-	0.32	827	$C_{20}H_{34}O_2$	306	1783-84-2	WileyRegistry8e
35.55	5,8,11,14-Eicosatetraenoic acid, methyl ester, (all-Z)-	0.32	819	$C_{21}H_{34}O_2$	318	2566-89-4	replib

Compound Structure	Hit Spectrum

Cholestan-3-ol, 2-methylene-, (3á,5à)-
Formula C28H48O, MW 400, CAS# 22599-96-8, Entry# 35411
5à-Cholestan-3á-ol, 2-methylene-

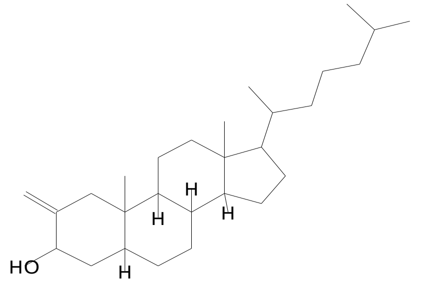

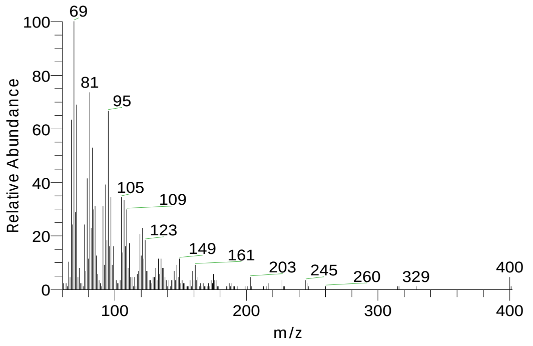

CHOLESTAN-3-OL, 2-METHYLENE-, (3á,5à)-
Formula C28H48O, MW 400, CAS# 22599-96-8, Entry# 256365
2-METHYLENECHOLESTAN-3-OL #

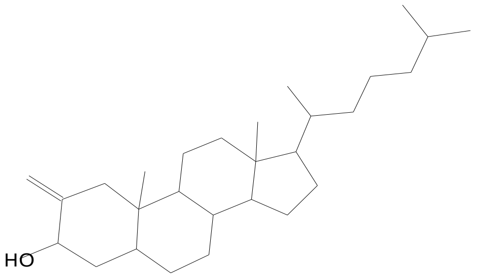

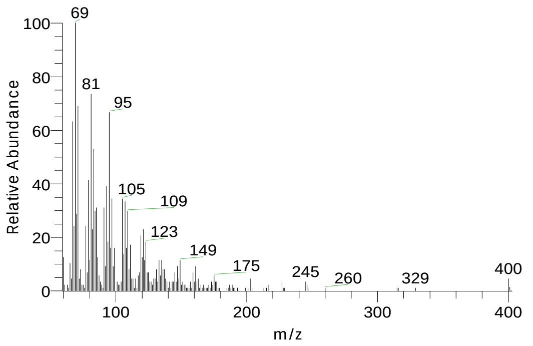

My GC-MS Report

Compound Structure | Hit Spectrum

8,11,14-Eicosatrienoic acid, (Z,Z,Z)-
Formula C20H34O2, MW 306, CAS# 1783-84-2, Entry# 1122
cis-8,11,14-Eicosatrienoic Acid

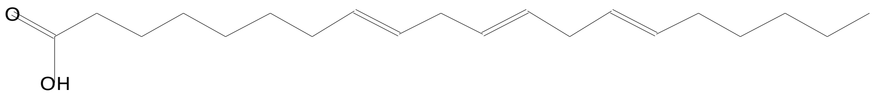

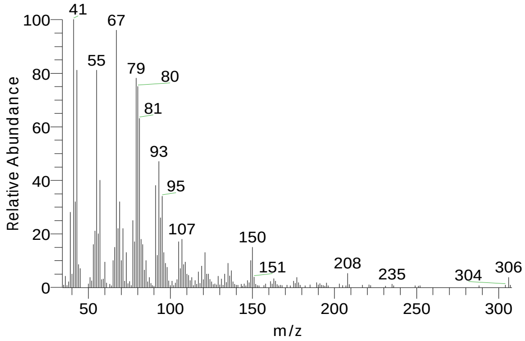

8,11,14-EICOSATRIENOIC ACID, (Z,Z,Z)-
Formula C20H34O2, MW 306, CAS# 1783-84-2, Entry# 195094
(8E,11E,14E)-8,11,14-ICOSATRIENOIC ACID #

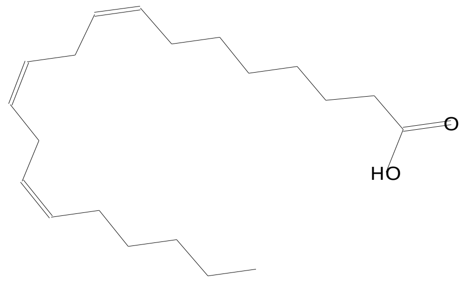

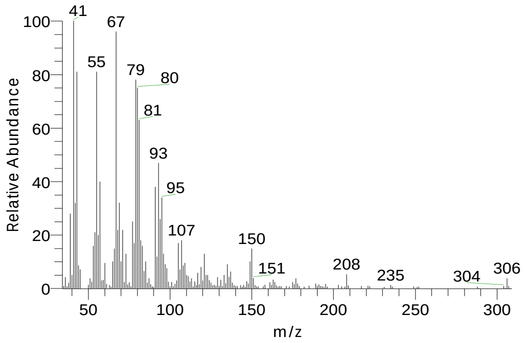

5,8,11,14-Eicosatetraenoic acid, methyl ester, (all-Z)-
Formula C21H34O2, MW 318, CAS# 2566-89-4, Entry# 916
Arachidonic acid methyl ester

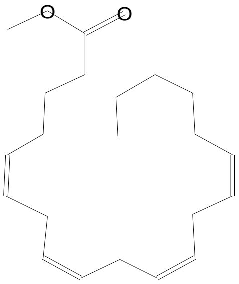

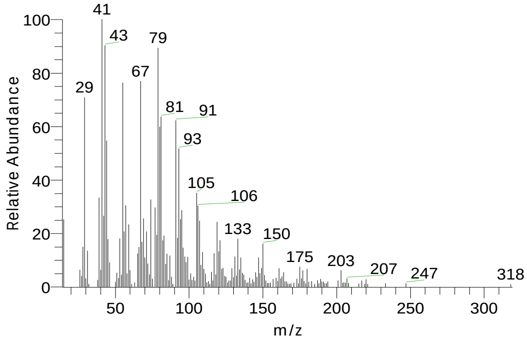

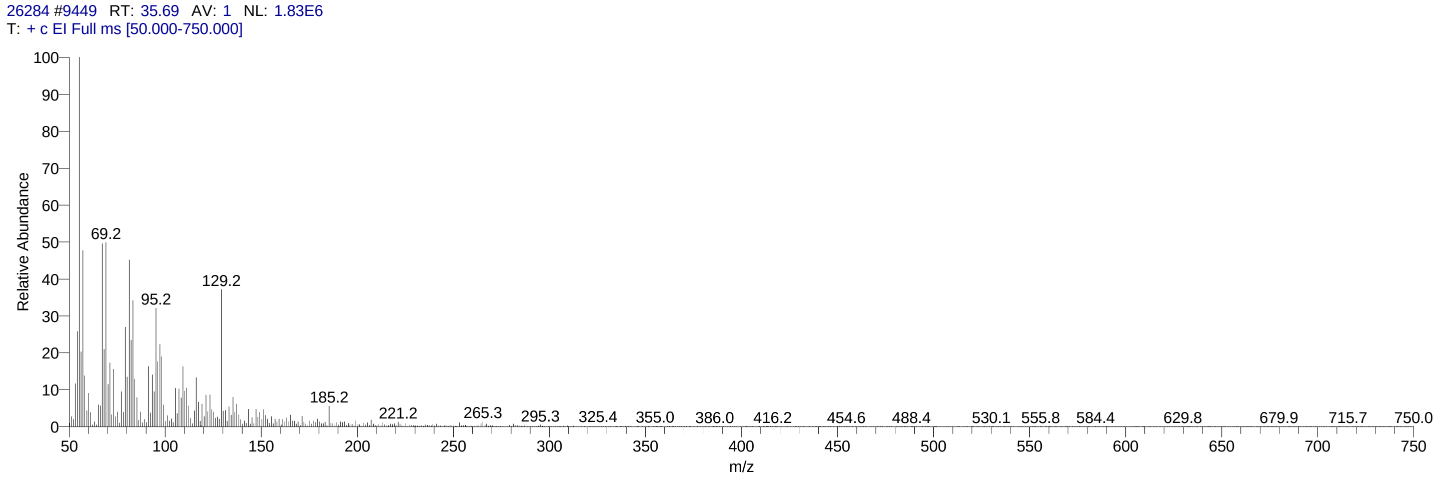

RT	Compound Name	Area %	MF	Molecular Formula	Molecular Weight	Cas #	Library
35.69	9-OCTADECENOIC ACID (Z)-	0.46	821	C18H34O2	282	112-80-1	WileyRegistry8e
35.69	Glycidyl oleate	0.46	822	C21H38O3	338	NA	mainlib
35.69	9,12-Octadecadienoyl chloride, (Z,Z)-	0.46	837	C18H31ClO	298	7459-33-8	replib

My GC-MS Report

RT	Compound Name	Area %	MF	Molecular Formula	Molecular Weight	Cas #	Library
35.69	(9E,12E)-9,12-OCTADECADIENOYL CHLORIDE #	0.46	837	C18H31ClO	298	7459-33-8	WileyRegistry8e
35.69	1-Heptatriacotanol	0.46	792	C37H76O	536	105794-58-9	mainlib

Compound Structure	Hit Spectrum

9-OCTADECENOIC ACID (Z)-
Formula C18H34O2, MW 282, CAS# 112-80-1, Entry# 172910
OCTADEC-9-ENOIC ACID

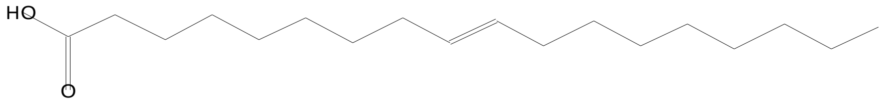

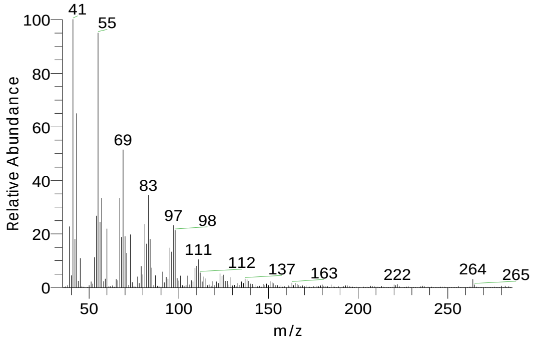

Glycidyl oleate
Formula C21H38O3, MW 338, CAS# NA, Entry# 112970
$:28VWYIWOYBERNXLX-KTKRTIGZSA-N

SI 812, RSI 822, mainlib, Entry# 112970, CAS# NA, Glycidyl oleate

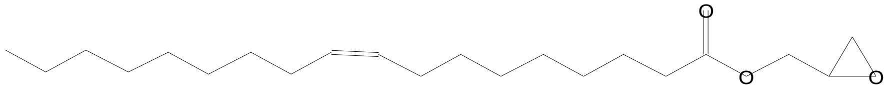

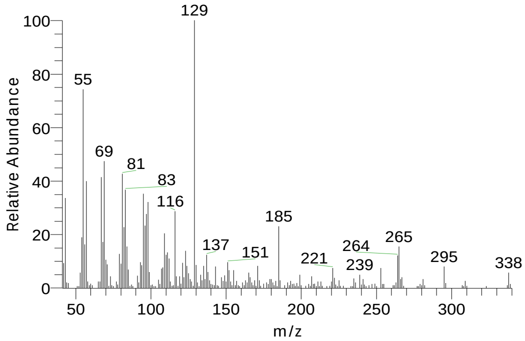

9,12-Octadecadienoyl chloride, (Z,Z)-
Formula C18H31ClO, MW 298, CAS# 7459-33-8, Entry# 4940
Linoleoyl chloride

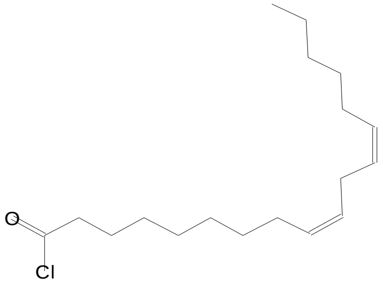

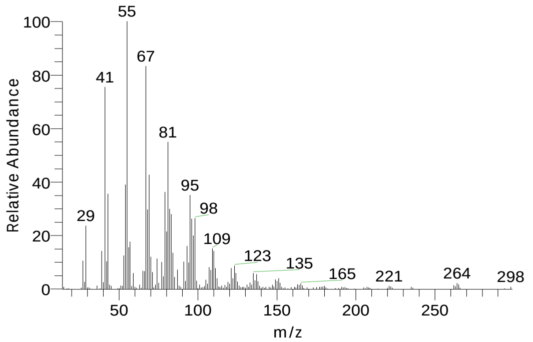

(9E,12E)-9,12-OCTADECADIENOYL CHLORIDE #
Formula C18H31ClO, MW 298, CAS# 7459-33-8, Entry# 187801
(9E,12E)-9,12-OCTADECADIENOYL CHLORIDE

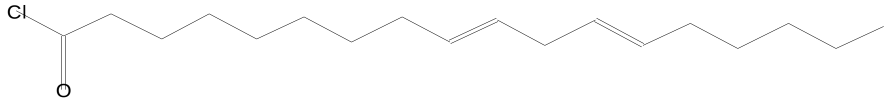

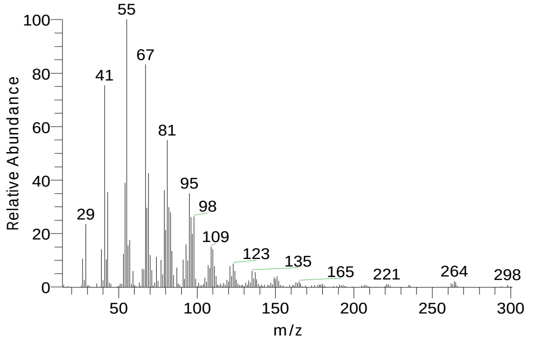

My GC-MS Report

Compound Structure | Hit Spectrum

1-Heptatriacotanol
Formula C37H76O, MW 536, CAS# 105794-58-9, Entry# 7279
1-Heptatriacontanol #

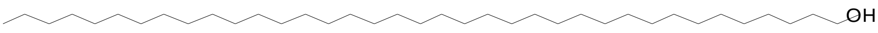

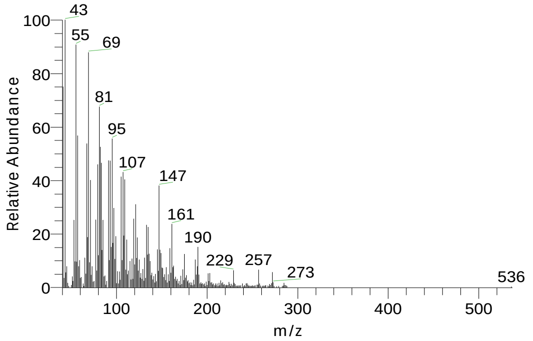

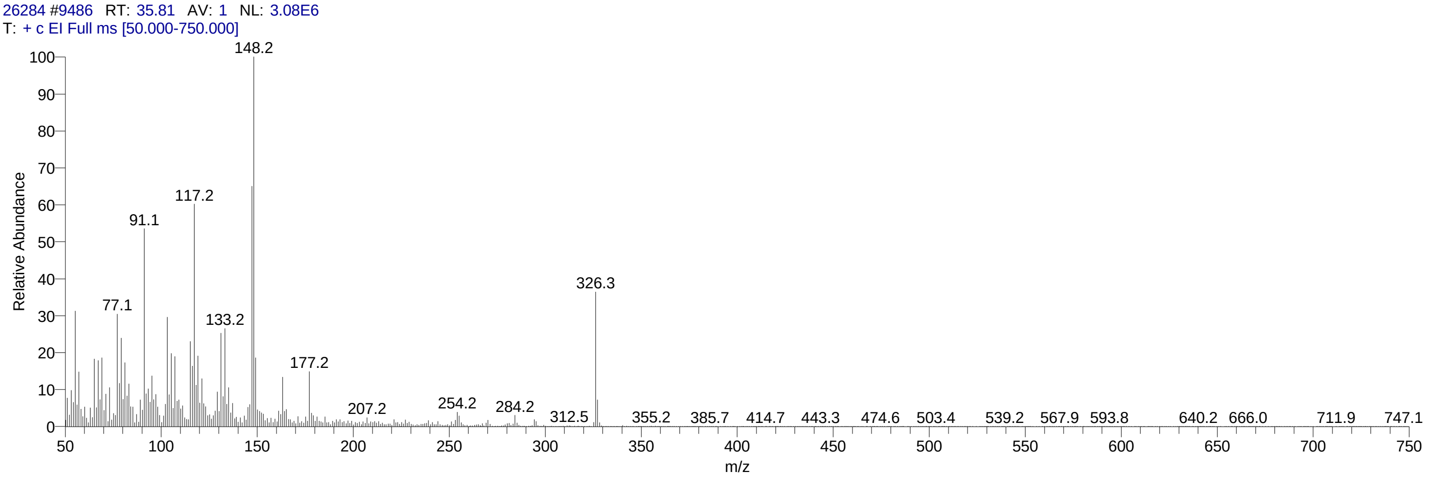

RT	Compound Name	Area %	MF	Molecular Formula	Molecular Weight	Cas #	Library
35.81	PREGNA-1,4-DIENE-3,11,20-TRIONE, 17,21-DIHYDROXY-	2.42	698	C21H26O5	358	53-03-2	WileyRegistry8e
35.81	Prednisone	2.42	697	C21H26O5	358	53-03-2	replib
35.81	4,7-Octadecadiynoic acid, methyl ester	2.42	755	C19H30O2	290	18202-20-5	mainlib
35.81	Methyl octadec-6,9-dien-12-ynoate	2.42	763	C19H30O2	290	NA	mainlib
35.81	4,7-OCTADECADIYNOIC ACID, METHYL ESTER	2.42	748	C19H30O2	290	18202-20-5	WileyRegistry8e

Compound Structure | Hit Spectrum

PREGNA-1,4-DIENE-3,11,20-TRIONE, 17,21-DIHYDROXY-
Formula C21H26O5, MW 358, CAS# 53-03-2, Entry# 395922
.DELTA. E

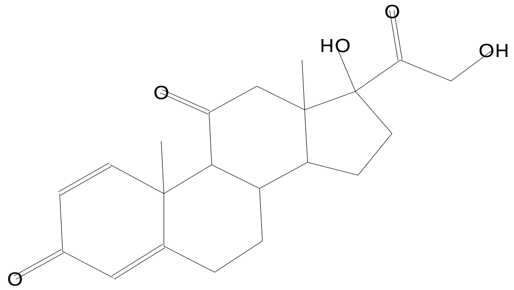

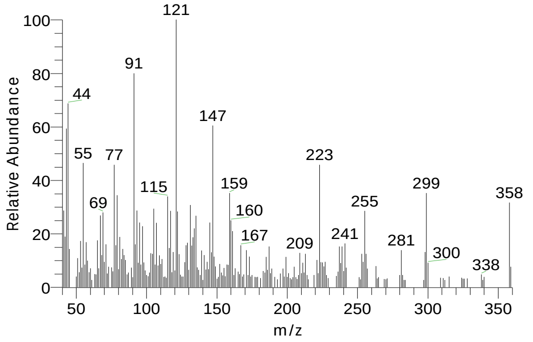

My GC-MS Report

Compound Structure | Hit Spectrum

Prednisone
Formula C21H26O5, MW 358, CAS# 53-03-2, Entry# 19140
Pregna-1,4-diene-3,11,20-trione, 17,21-dihydroxy-

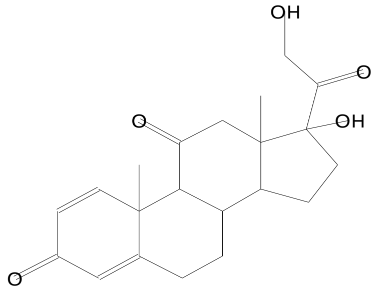

SI 679, RSI 697, replib, Entry# 19140, CAS# 53-03-2, Prednisone

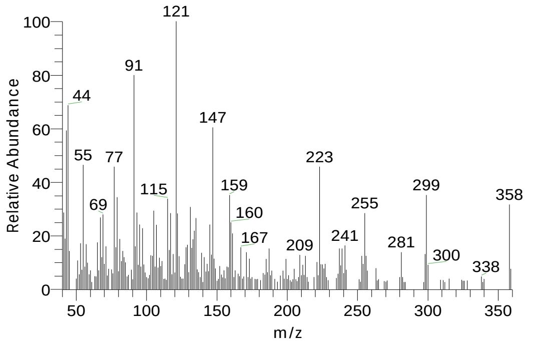

4,7-Octadecadiynoic acid, methyl ester
Formula C19H30O2, MW 290, CAS# 18202-20-5, Entry# 77955
Methyl 4,7-octadecadiynoate #

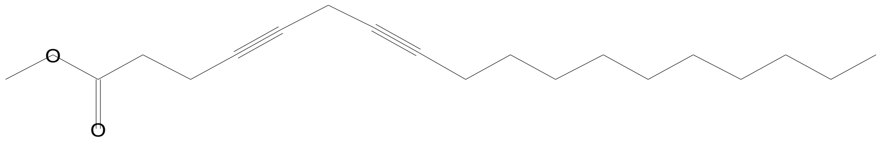

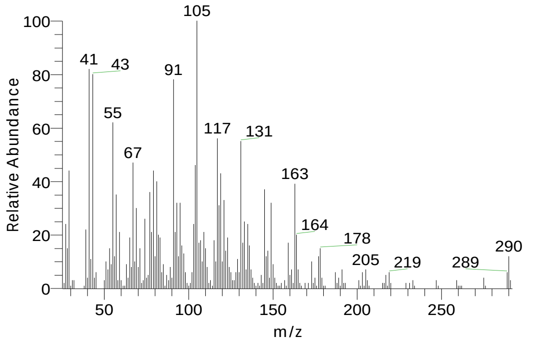

Methyl octadec-6,9-dien-12-ynoate
Formula C19H30O2, MW 290, CAS# NA, Entry# 60972
$:28ZZCKBSQXBOGYRI-XVTLYKPTSA-N

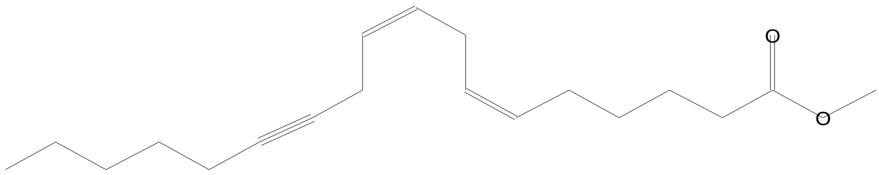

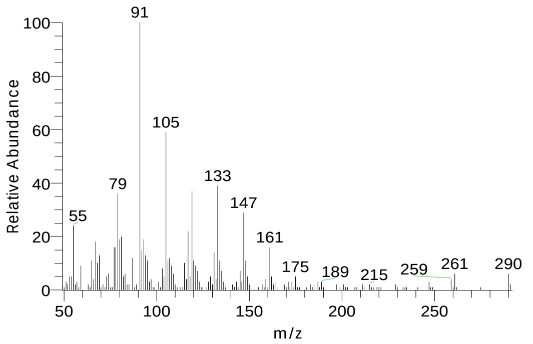

4,7-OCTADECADIYNOIC ACID, METHYL ESTER
Formula C19H30O2, MW 290, CAS# 18202-20-5, Entry# 180552
METHYL 4,7-OCTADECADIYNOATE #

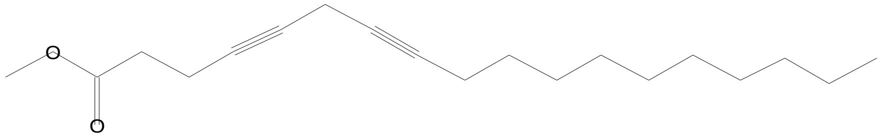

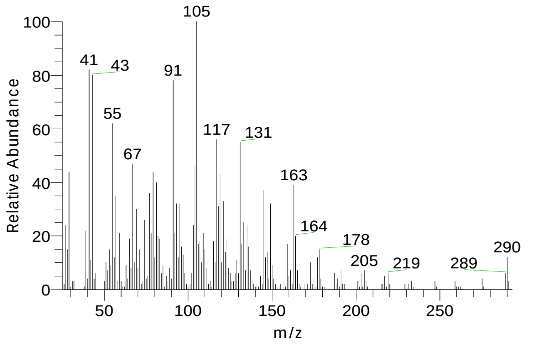

My GC-MS Report

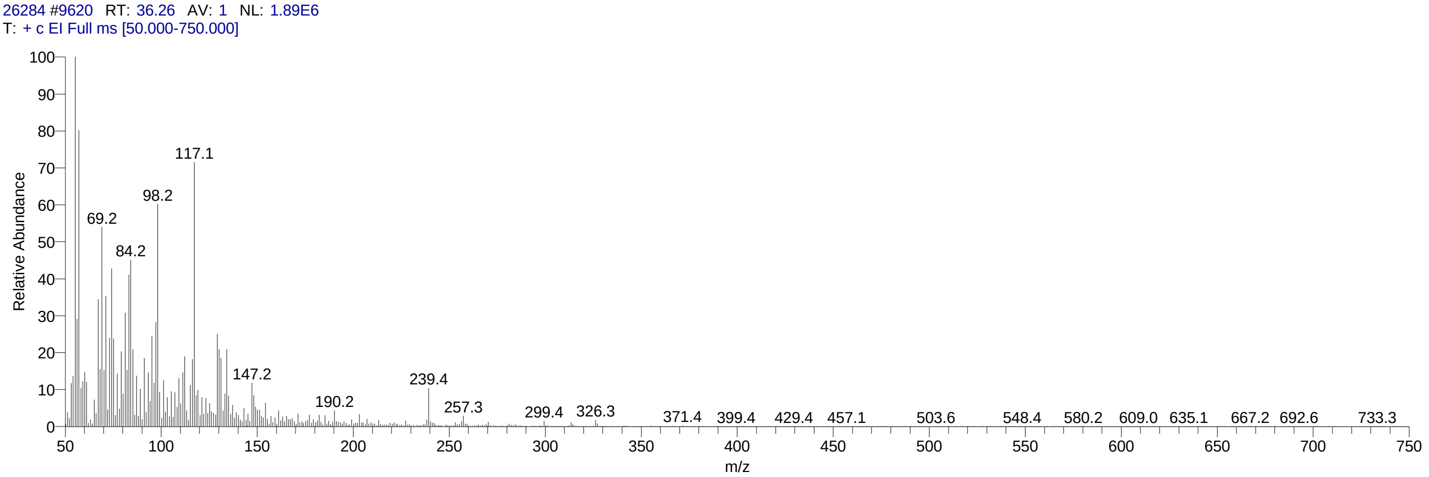

RT	Compound Name	Area %	MF	Molecular Formula	Molecular Weight	Cas #	Library
36.26	HEXADECANOIC ACID, 2,3-DIHYDROXYPROPYL ESTER	1.98	783	C19H38O4	330	542-44-9	WileyRegistry8e
36.26	HEXADECANOIC ACID, 1-(HYDROXYMETHYL)-1,2-ETHANEDIYL ESTER	1.98	771	C35H68O5	568	761-35-3	WileyRegistry8e
36.26	Hexadecanoic acid, 2-hydroxy-1-(hydroxymethyl)ethyl ester	1.98	813	C19H38O4	330	23470-00-0	mainlib
36.26	HEXADECANOIC ACID, 2-HYDROXY-1-(HYDROXYMETHYL)ETHYL ESTER	1.98	806	C19H38O4	330	23470-00-0	WileyRegistry8e
36.26	Glycerol 1-palmitate	1.98	803	C19H38O4	330	542-44-9	mainlib

Compound Structure	Hit Spectrum

HEXADECANOIC ACID, 2,3-DIHYDROXYPROPYL ESTER
Formula C19H38O4, MW 330, CAS# 542-44-9, Entry# 214589
2,3-DIHYDROXYPROPYL PALMITATE #

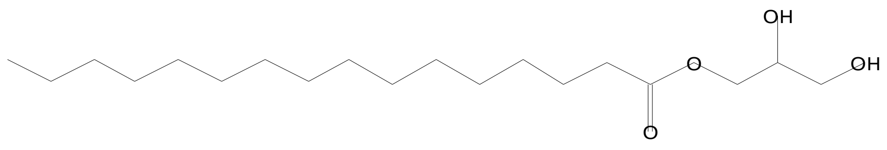

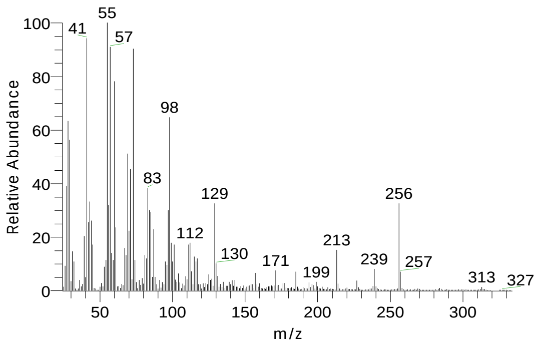

HEXADECANOIC ACID, 1-(HYDROXYMETHYL)-1,2-ETHANEDIYL ESTER
Formula C35H68O5, MW 568, CAS# 761-35-3, Entry# 294146
2-HYDROXY-1-[(PALMITOYLOXY)METHYL]ETHYL PALMITATE #

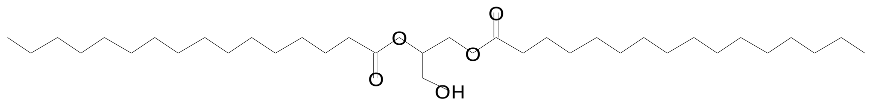

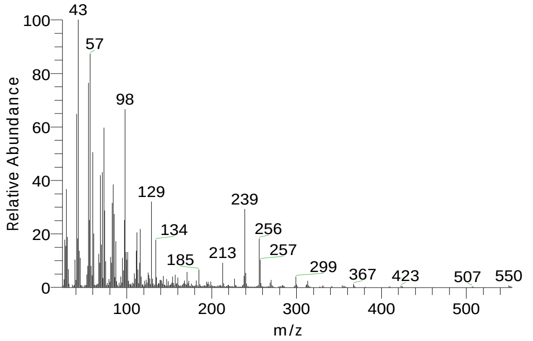

My GC-MS Report

Compound Structure | Hit Spectrum

Hexadecanoic acid, 2-hydroxy-1-(hydroxymethyl)ethyl ester
Formula C19H38O4, MW 330, CAS# 23470-00-0, Entry# 7660
Palmitin, 2-mono-

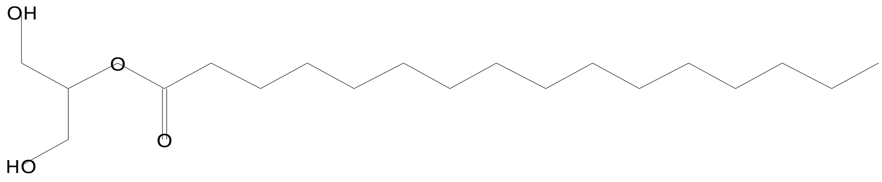

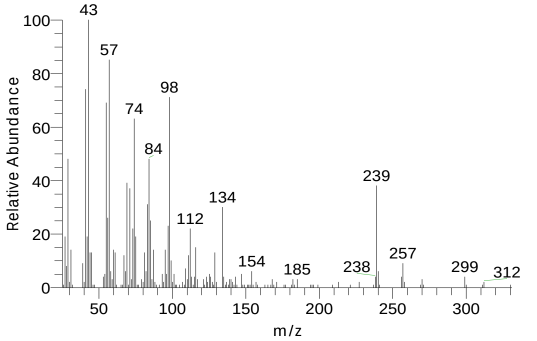

HEXADECANOIC ACID, 2-HYDROXY-1-(HYDROXYMETHYL)ETHYL ESTER
Formula C19H38O4, MW 330, CAS# 23470-00-0, Entry# 214591
2-HYDROXY-1-(HYDROXYMETHYL)ETHYL PALMITATE #

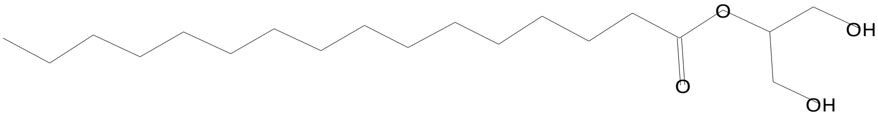

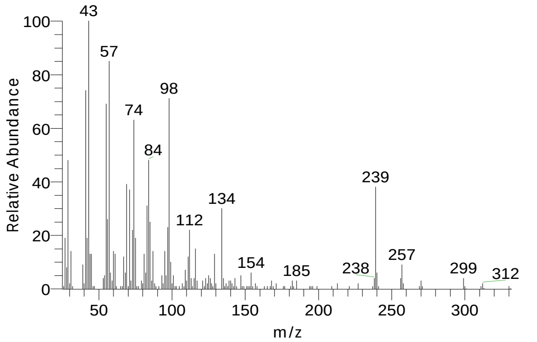

Glycerol 1-palmitate
Formula C19H38O4, MW 330, CAS# 542-44-9, Entry# 7679
Hexadecanoic acid, 2,3-dihydroxypropyl ester

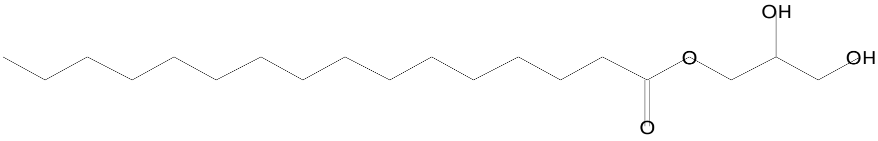

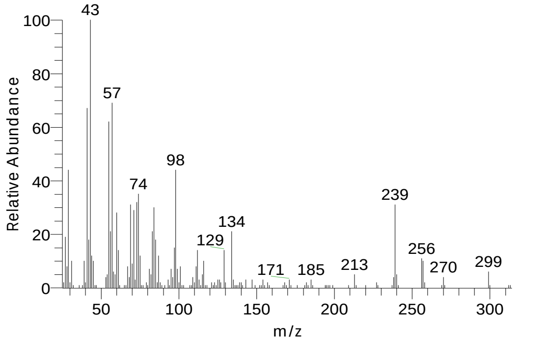

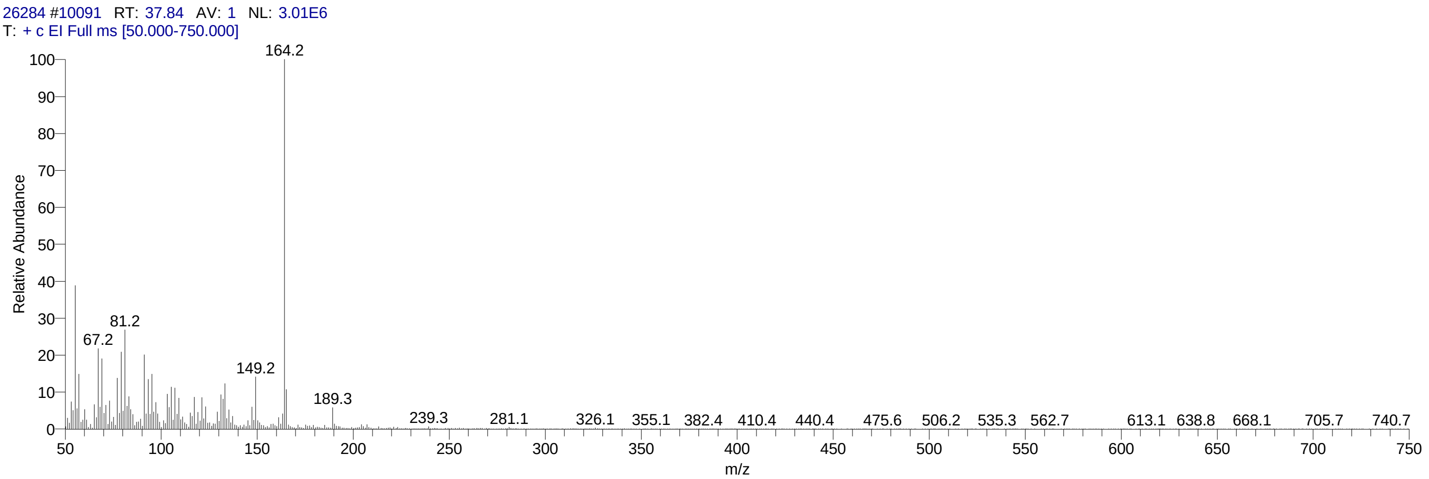

RT	Compound Name	Area %	MF	Molecular Formula	Molecular Weight	Cas #	Library
37.84	6,9,12,15-Docosatetraenoic acid, methyl ester	0.90	723	C23H38O2	346	17364-34-0	mainlib
37.84	6,9,12,15-DOCOSATETRAENOIC ACID, METHYL ESTER	0.90	723	C23H38O2	346	17364-34-0	WileyRegistry8e

My GC-MS Report

RT	Compound Name	Area %	MF	Molecular Formula	Molecular Weight	Cas #	Library
37.84	PHENOL, 2-METHOXY-4-(1-PROPENYL)-	0.90	800	C10H12O2	164	97-54-1	WileyRegistry8e
37.84	5,8,11,14-Eicosatetraenoic acid, methyl ester, (all-Z)-	0.90	723	C21H34O2	318	2566-89-4	replib
37.84	3-METHYL-2-PENT-2-ENYL-CYCLOPENT-2-ENONE	0.90	740	C11H16O	164	NA	WileyRegistry8e

Compound Structure | Hit Spectrum

6,9,12,15-Docosatetraenoic acid, methyl ester
Formula C23H38O2, MW 346, CAS# 17364-34-0, Entry# 2481
Methyl (6E,9E,12E,15E)-6,9,12,15-docosatetraenoate #

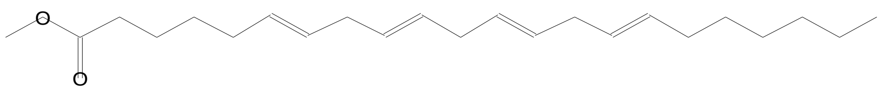

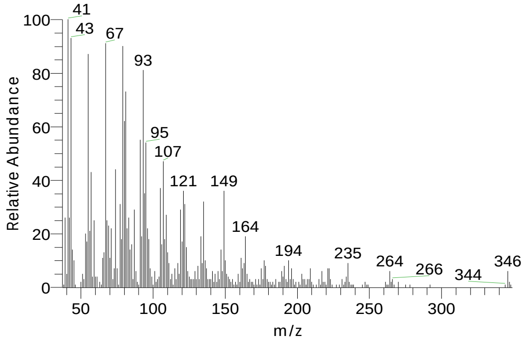

6,9,12,15-DOCOSATETRAENOIC ACID, METHYL ESTER
Formula C23H38O2, MW 346, CAS# 17364-34-0, Entry# 226311
METHYL (6E,9E,12E,15E)-6,9,12,15-DOCOSATETRAENOATE #

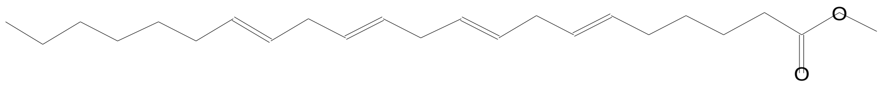

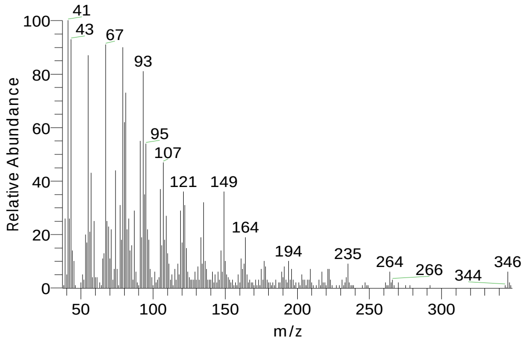

PHENOL, 2-METHOXY-4-(1-PROPENYL)-
Formula C10H12O2, MW 164, CAS# 97-54-1, Entry# 46882
2-METHOXY-4-[(1E)-1-PROPENYL]PHENOL #

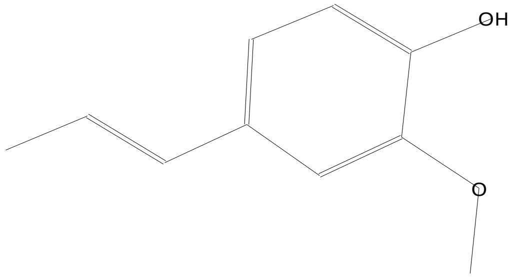

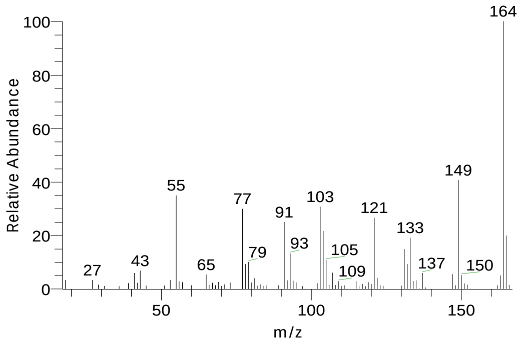

5,8,11,14-Eicosatetraenoic acid, methyl ester, (all-Z)-
Formula C21H34O2, MW 318, CAS# 2566-89-4, Entry# 916
Arachidonic acid methyl ester

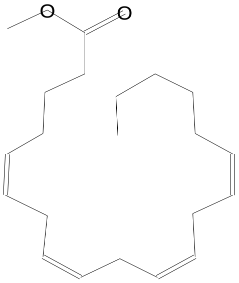

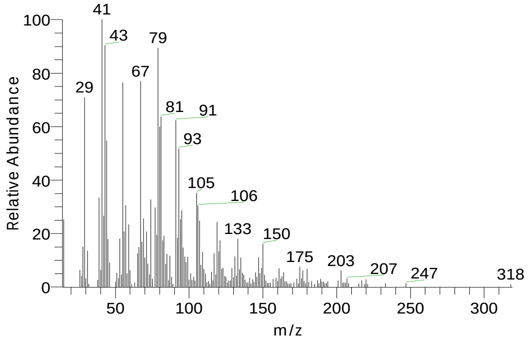

My GC-MS Report

Compound Structure | Hit Spectrum

3-METHYL-2-PENT-2-ENYL-CYCLOPENT-2-ENONE
Formula C11H16O, MW 164, CAS# NA, Entry# 361185
3-METHYL-2-[2-PENTENYL]-2-CYCLOPENTEN-1-ONE

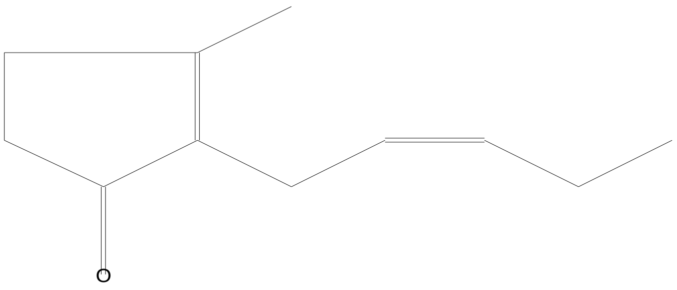

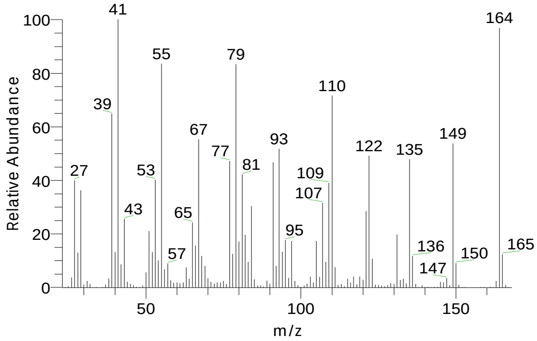

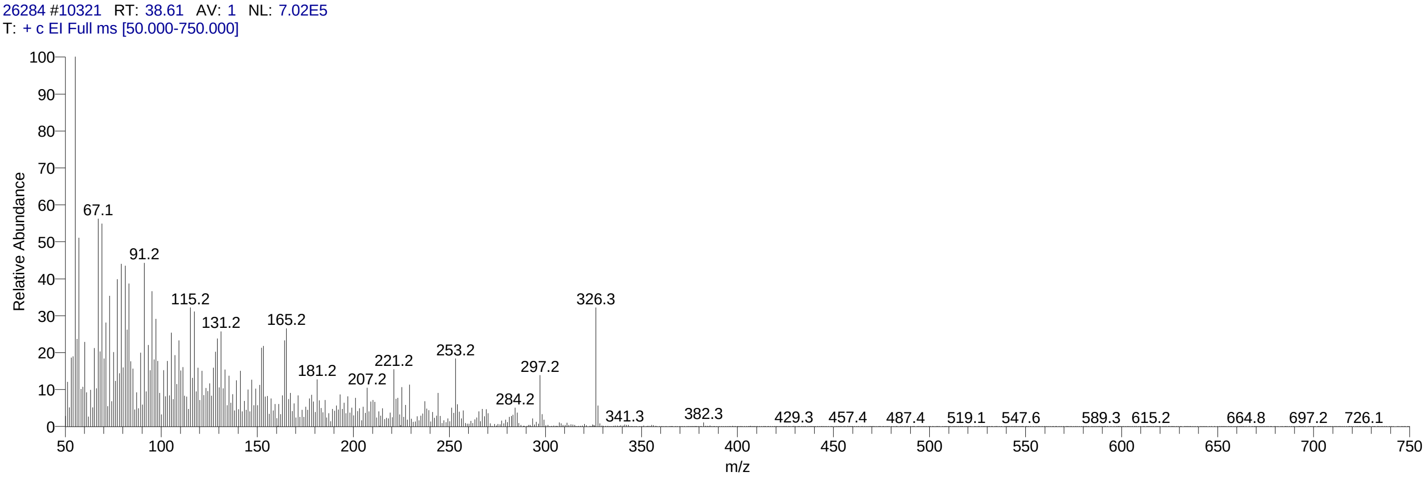

RT	Compound Name	Area %	MF	Molecular Formula	Molecular Weight	Cas #	Library
38.61	5-(3,4-Dimethoxyphenyl)-3-(2-hydroxyphenyl)-4,5-dihydropyrazole-1-carbaldehyde	0.69	689	C18H18N2O4	326	NA	mainlib
38.61	[1,1'-Biphenyl]-2,2'-diol, 3,3'-dimethoxy-5,5'-di-2-propenyl-	0.69	742	C20H22O4	326	4433-08-3	replib
38.61	6-Amino-5-cyano-2-propyl-4-p-tolyl-4H-pyran-3-carboxylic acid ethyl ester	0.69	713	C19H22N2O3	326	NA	mainlib
38.61	ETHYL 6-AMINO-5-CYANO-4-(4-METHYLPHENYL)-2-PROPYL-4H-PYRAN-3-CARBOXYLATE	0.69	713	C19H22N2O3	326	NA	WileyRegistry8e
38.61	3'-Benzyloxy-5,6,7,4'-tetramethoxyflavone	0.69	680	C26H24O7	448	21764-08-9	nist_msms

Compound Structure | Hit Spectrum

5-(3,4-Dimethoxyphenyl)-3-(2-hydroxyphenyl)-4,5-dihydropyrazole-1-carbaldehyde
Formula C18H18N2O4, MW 326, CAS# NA, Entry# 46865
$:28XDCJABUHKANNPE-UHFFFAOYSA-N

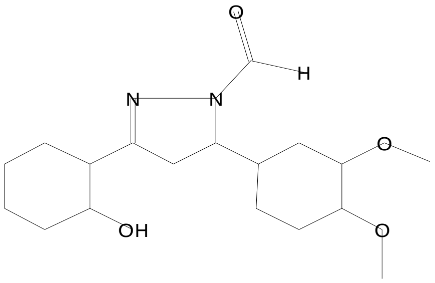

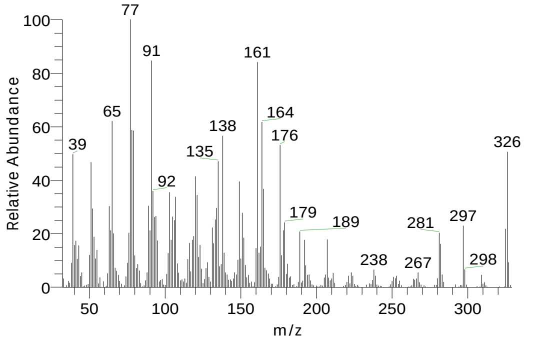

My GC-MS Report

Compound Structure | Hit Spectrum

[1,1'-Biphenyl]-2,2'-diol, 3,3'-dimethoxy-5,5'-di-2-propenyl-
Formula C20H22O4, MW 326, CAS# 4433-08-3, Entry# 32971
2,2'-Biphenyldiol, 5,5'-diallyl-3,3'-dimethoxy-

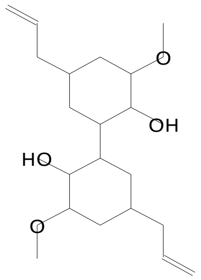

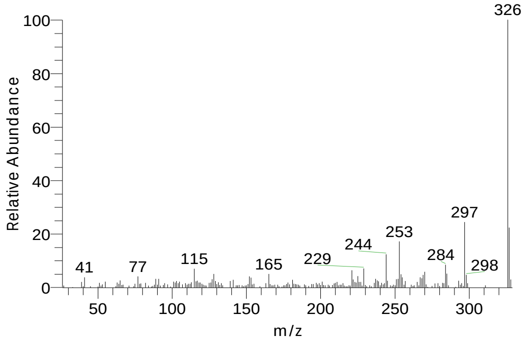

6-Amino-5-cyano-2-propyl-4-p-tolyl-4H-pyran-3-carboxylic acid ethyl ester
Formula C19H22N2O3, MW 326, CAS# NA, Entry# 13948
Ethyl 6-amino-5-cyano-4-(4-methylphenyl)-2-propyl-4H-pyran-3-carboxylate #

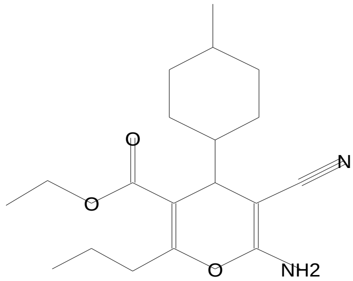

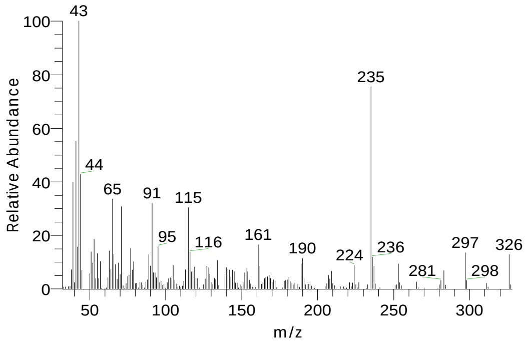

ETHYL 6-AMINO-5-CYANO-4-(4-METHYLPHENYL)-2-PROPYL-4H-PYRAN-3-CARBOXYLATE
Formula C19H22N2O3, MW 326, CAS# NA, Entry# 328887
6-AMINO-5-CYANO-2-PROPYL-4-P-TOLYL-4H-PYRAN-3-CARBOXYLIC ACID ETHYL ESTER

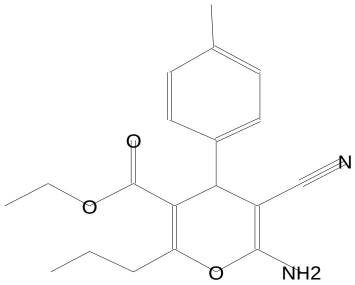

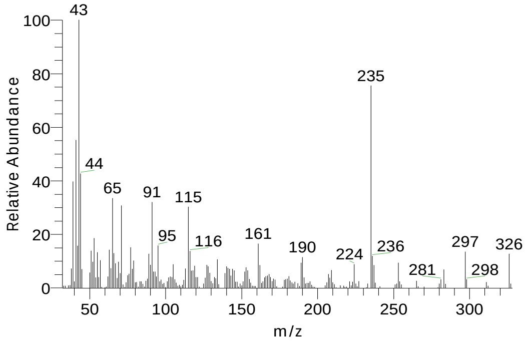

3'-Benzyloxy-5,6,7,4'-tetramethoxyflavone
Formula C26H24O7, MW 448, CAS# 21764-08-9, Entry# 87100
$:03[M+H]+

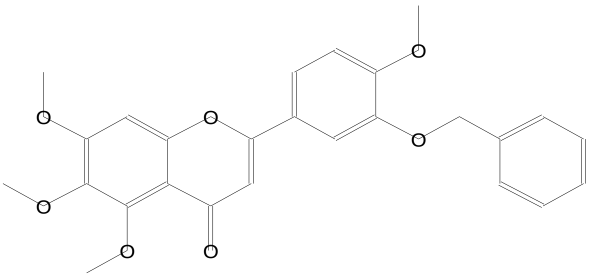

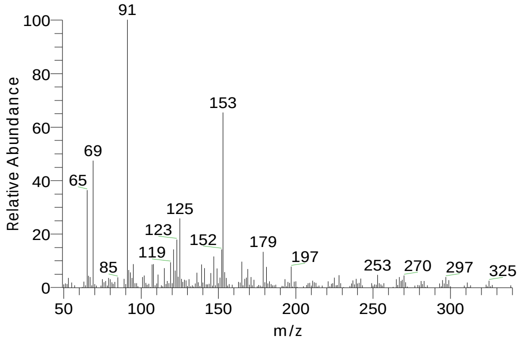

My GC-MS Report

26284 #10429 RT: 38.97 AV: 1 NL: 1.66E6
T: + c EI Full ms [50.000-750.000]

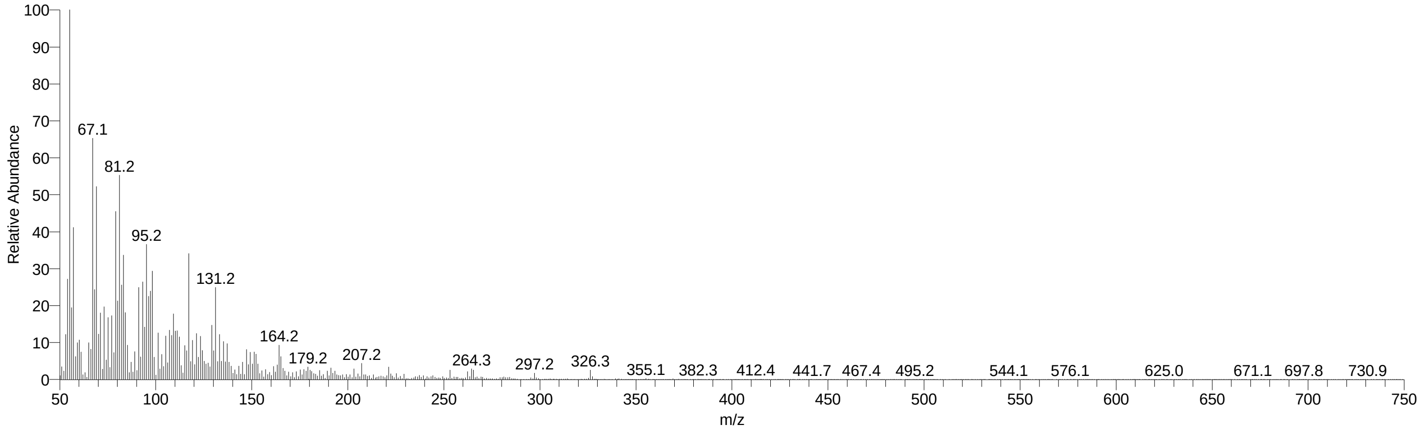

RT	Compound Name	Area %	MF	Molecular Formula	Molecular Weight	Cas #	Library
38.97	HI-OLEIC SAFFLOWER OIL	1.01	822	C21H22O11	450	8001-23-8	WileyRegistry8e
38.97	9,12-Octadecadienoyl chloride, (Z,Z)-	1.01	839	C18H31ClO	298	7459-33-8	replib
38.97	(9E,12E)-9,12-OCTADECADIENOYL CHLORIDE #	1.01	837	C18H31ClO	298	7459-33-8	WileyRegistry8e
38.97	9,12-Octadecadienoic acid (Z,Z)-, 2-hydroxy-1-(hydroxymethyl)ethyl ester	1.01	805	C21H38O4	354	3443-82-1	replib
38.97	Glycidyl oleate	1.01	777	C21H38O3	338	NA	mainlib

Compound Structure | Hit Spectrum

HI-OLEIC SAFFLOWER OIL
Formula C21H22O11, MW 450, CAS# 8001-23-8, Entry# 274191
SAFFLOWER OIL

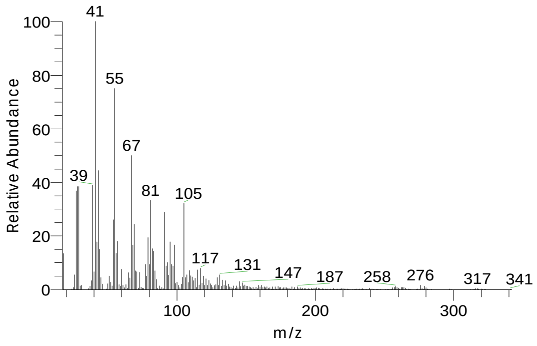

9,12-Octadecadienoyl chloride, (Z,Z)-
Formula C18H31ClO, MW 298, CAS# 7459-33-8, Entry# 4940
Linoleoyl chloride

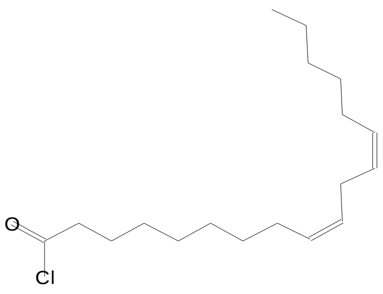

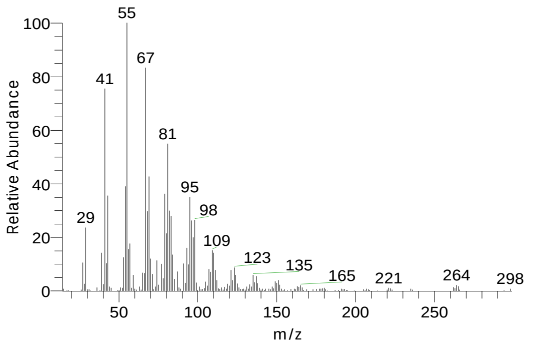

My GC-MS Report

Compound Structure | Hit Spectrum

(9E,12E)-9,12-OCTADECADIENOYL CHLORIDE #
Formula C18H31ClO, MW 298, CAS# 7459-33-8, Entry# 187801
(9E,12E)-9,12-OCTADECADIENOYL CHLORIDE

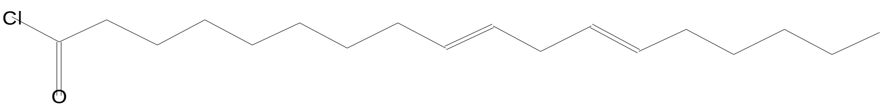

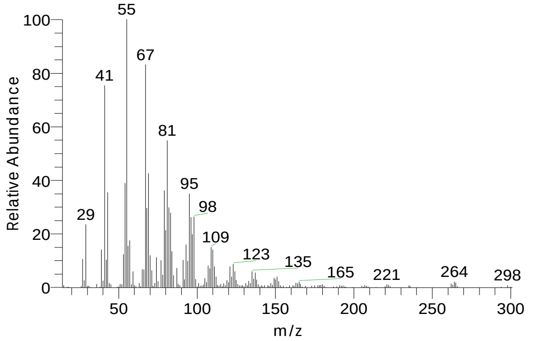

9,12-Octadecadienoic acid (Z,Z)-, 2-hydroxy-1-(hydroxymethyl)ethyl ester
Formula C21H38O4, MW 354, CAS# 3443-82-1, Entry# 8055
Linolein, 2-mono-

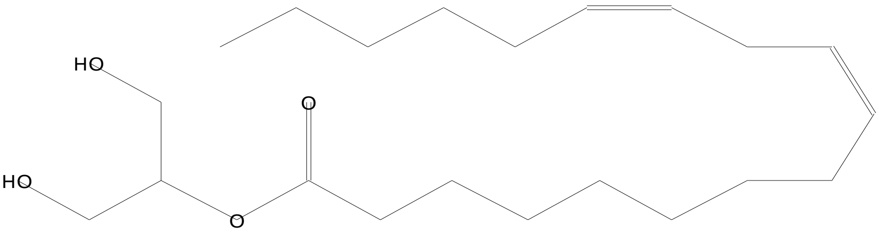

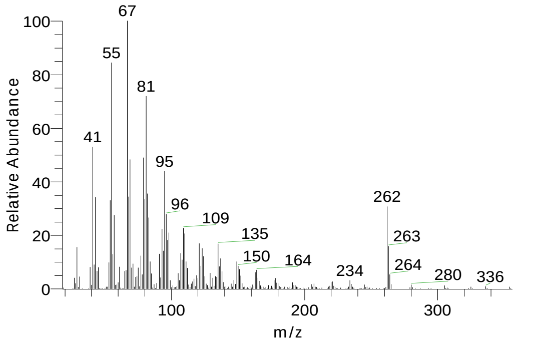

Glycidyl oleate
Formula C21H38O3, MW 338, CAS# NA, Entry# 112970
$:28VWYIWOYBERNXLX-KTKRTIGZSA-N

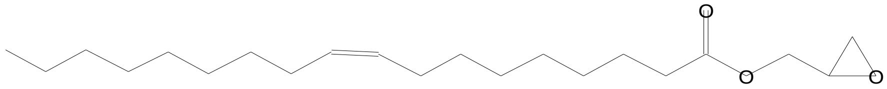

SI 752, RSI 777, mainlib, Entry# 112970, CAS# NA, Glycidyl oleate

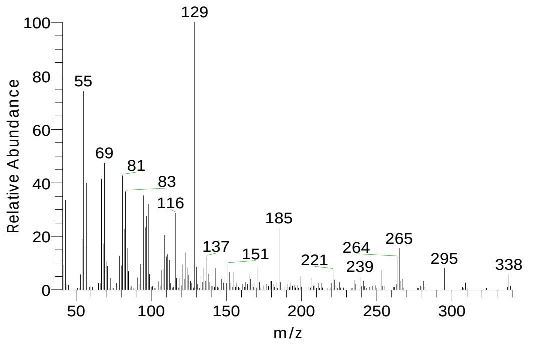

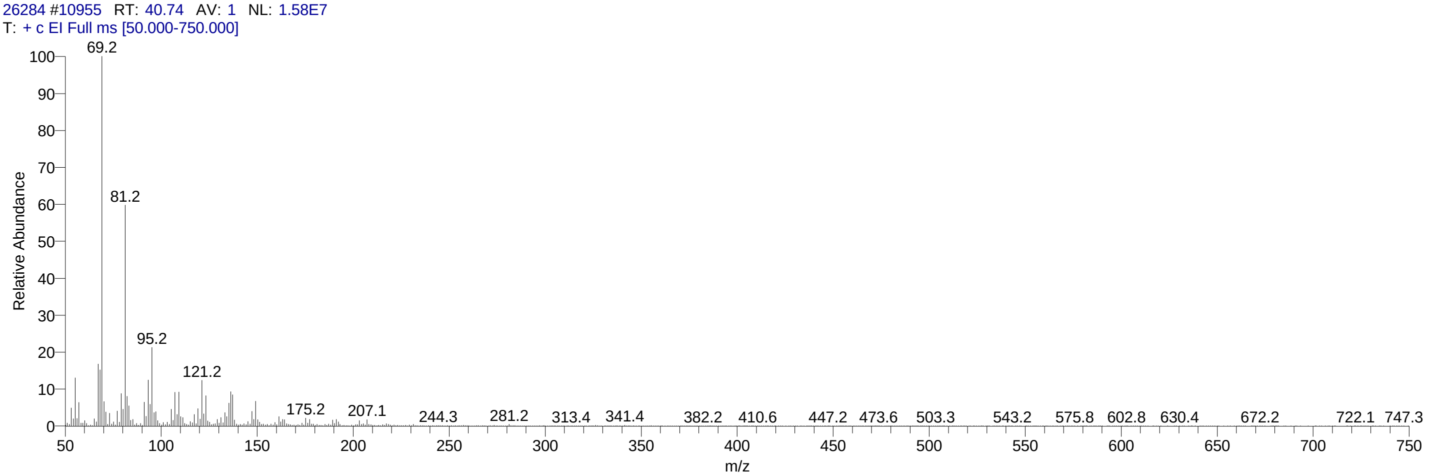

RT	Compound Name	Area %	MF	Molecular Formula	Molecular Weight	Cas #	Library
40.74	Squalene	2.71	910	C30H50	410	111-02-4	replib
40.74	trans-Geranylgeraniol	2.71	862	C20H34O	290	24034-73-9	mainlib

My GC-MS Report

RT	Compound Name	Area %	MF	Molecular Formula	Molecular Weight	Cas #	Library
40.74	1,6,10,14,18,22-Tetracosahexaen-3-ol, 2,6,10,15,19,23-hexamethyl-, (all-E)-(ñ)-	2.71	880	C30H50O	426	97232-74-1	mainlib
40.74	Squalene	2.71	892	C30H50	410	111-02-4	replib
40.74	Squalene	2.71	872	C30H50	410	111-02-4	mainlib

Compound Structure | Hit Spectrum

Squalene
Formula C30H50, MW 410, CAS# 111-02-4, Entry# 8707
2,6,10,14,18,22-Tetracosahexaene, 2,6,10,15,19,23-hexamethyl-, (all-E)-

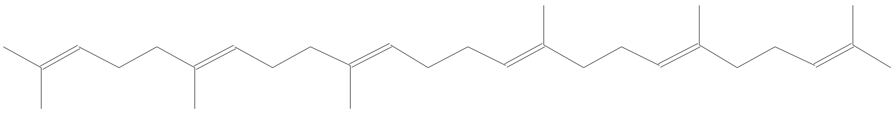

SI 853, RSI 910, replib, Entry# 8707, CAS# 111-02-4, Squalene

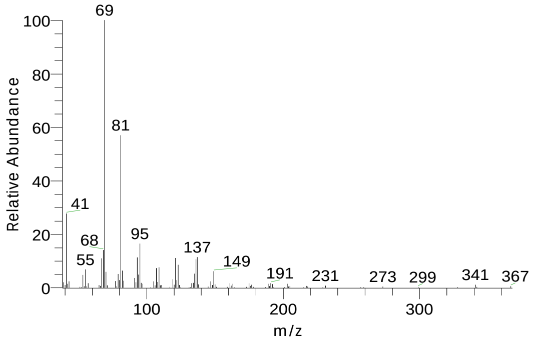

trans-Geranylgeraniol
Formula C20H34O, MW 290, CAS# 24034-73-9, Entry# 35392
2,6,10,14-Hexadecatetraen-1-ol, 3,7,11,15-tetramethyl-, (E,E,E)-

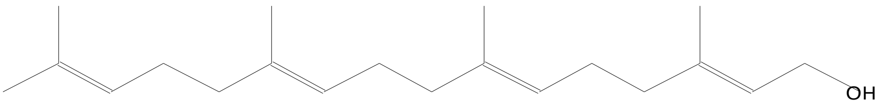

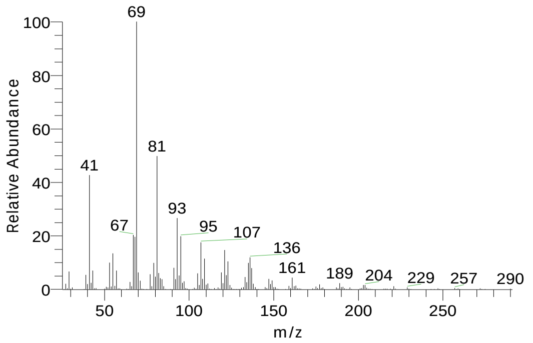

1,6,10,14,18,22-Tetracosahexaen-3-ol, 2,6,10,15,19,23-hexamethyl-, (all-E)-(ñ)-
Formula C30H50O, MW 426, CAS# 97232-74-1, Entry# 35419
$:28JLUBMMAQMKVTGL-RLROCYJYSA-N

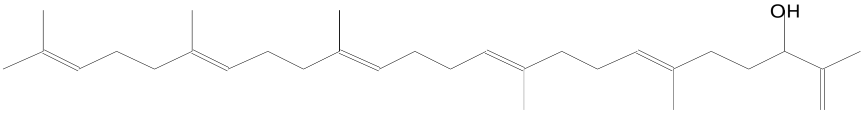

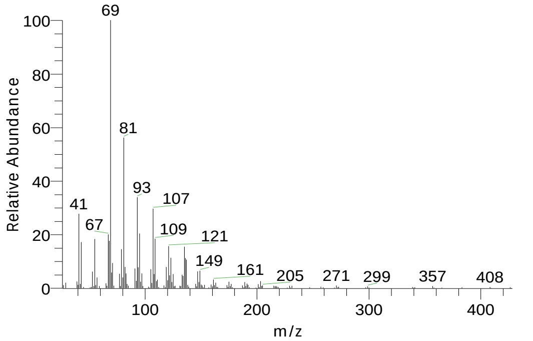

Squalene
Formula C30H50, MW 410, CAS# 111-02-4, Entry# 8705
2,6,10,14,18,22-Tetracosahexaene, 2,6,10,15,19,23-hexamethyl-, (all-E)-

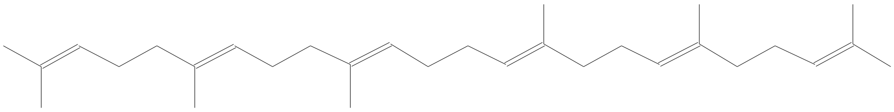

SI 842, RSI 892, replib, Entry# 8705, CAS# 111-02-4, Squalene

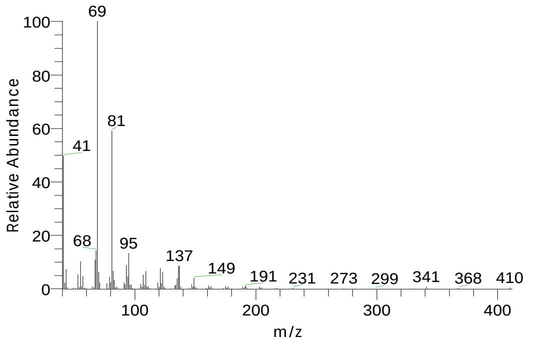

My GC-MS Report

Compound Structure | Hit Spectrum

Squalene
Formula C30H50, MW 410, CAS# 111-02-4, Entry# 35390
2,6,10,14,18,22-Tetracosahexaene, 2,6,10,15,19,23-hexamethyl-, (all-E)-

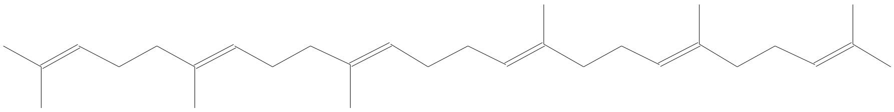

SI 839, RSI 872, mainlib, Entry# 35390, CAS# 111-02-4, Squalene

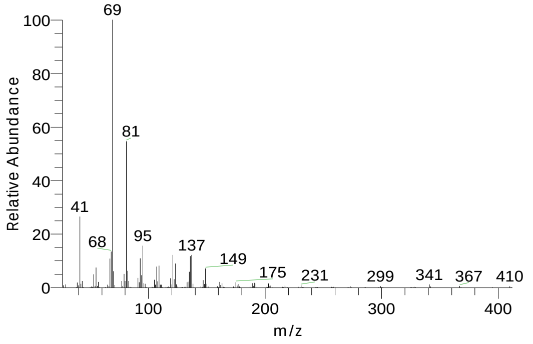

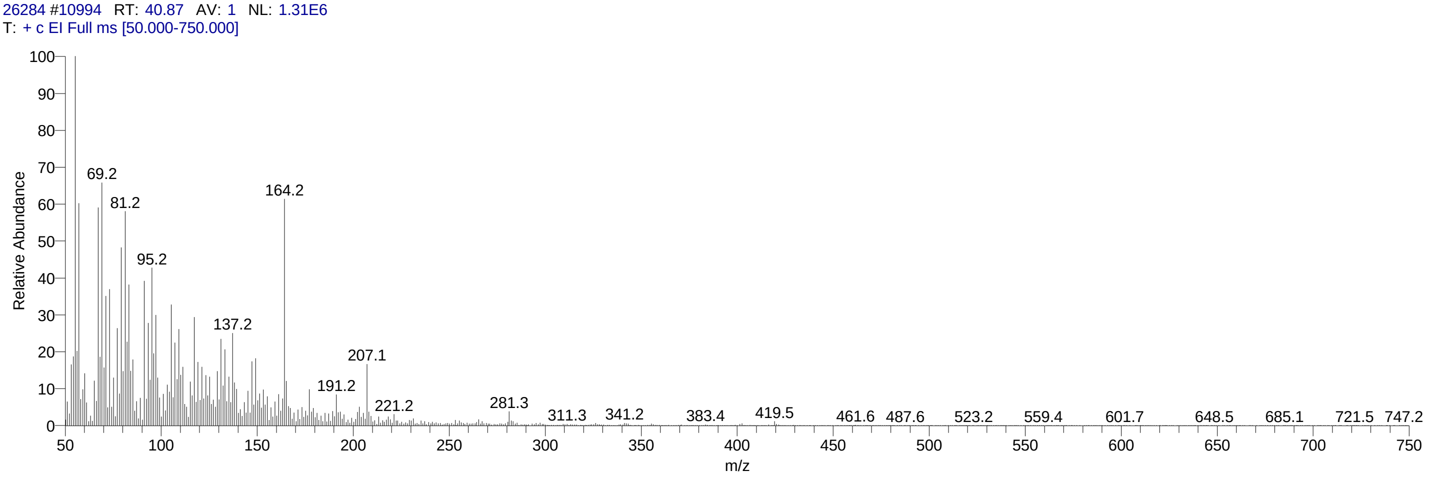

RT	Compound Name	Area %	MF	Molecular Formula	Molecular Weight	Cas #	Library
40.87	1-Heptatriacotanol	0.65	822	C37H76O	536	105794-58-9	mainlib
40.87	Ethyl iso-allocholate	0.65	781	C26H44O5	436	NA	mainlib
40.87	ETHYL ISO-ALLOCHOLATE	0.65	781	C26H44O5	436	NA	WileyRegistry8e
40.87	01297107001 TETRANEURIN - A - DIOL	0.65	788	C15H20O5	280	NA	WileyRegistry8e
40.87	HEXADECADIENOIC ACID, METHYL ESTER	0.65	777	C17H30O2	266	29961-54-4	WileyRegistry8e

Compound Structure | Hit Spectrum

1-Heptatriacotanol
Formula C37H76O, MW 536, CAS# 105794-58-9, Entry# 7279
1-Heptatriacontanol #

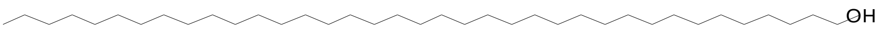

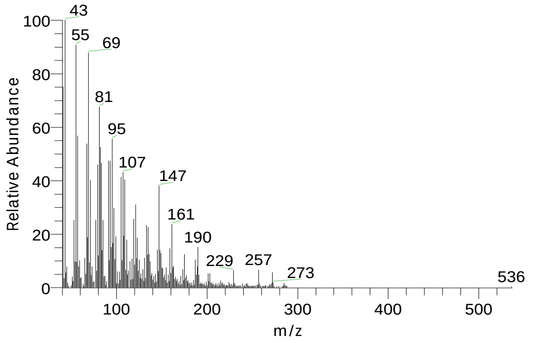

My GC-MS Report

Compound Structure | Hit Spectrum

Ethyl iso-allocholate
Formula C26H44O5, MW 436, CAS# NA, Entry# 7020
Ethyl 3,7,12-trihydroxycholan-24-oate #

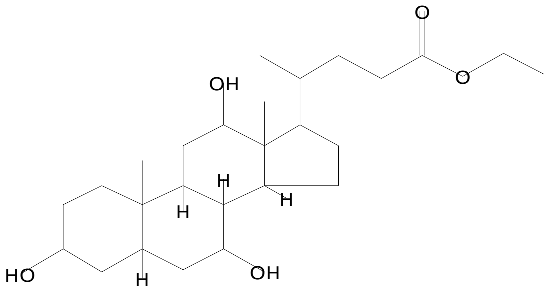

SI 780, RSI 781, mainlib, Entry# 7020, CAS# NA, Ethyl iso-allocholate

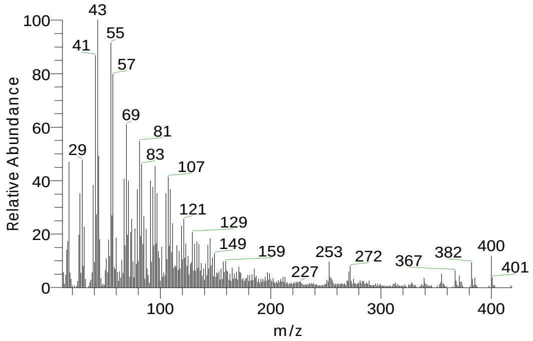

ETHYL ISO-ALLOCHOLATE
Formula C26H44O5, MW 436, CAS# NA, Entry# 270212

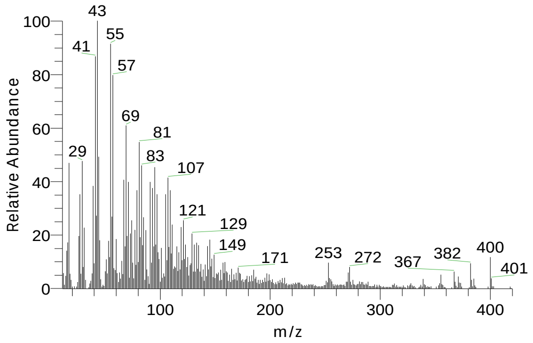

01297107001 TETRANEURIN - A - DIOL
Formula C15H20O5, MW 280, CAS# NA, Entry# 170378

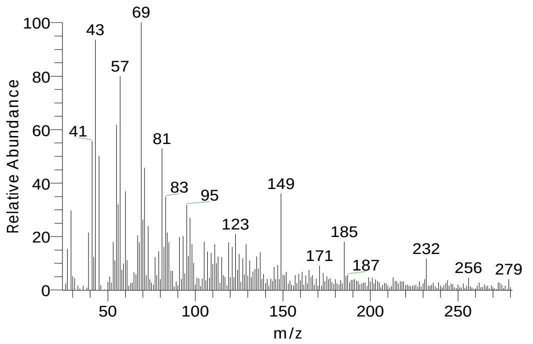

HEXADECADIENOIC ACID, METHYL ESTER
Formula C17H30O2, MW 266, CAS# 29961-54-4, Entry# 157129
METHYL HEXADECADIENOATE

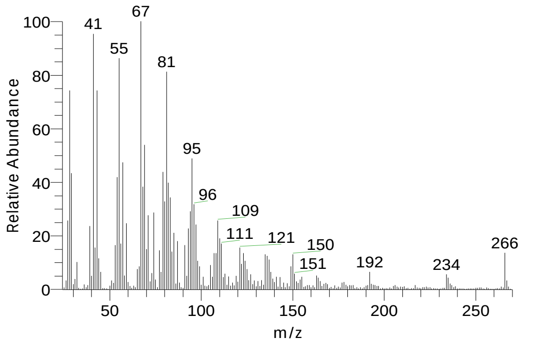

My GC-MS Report

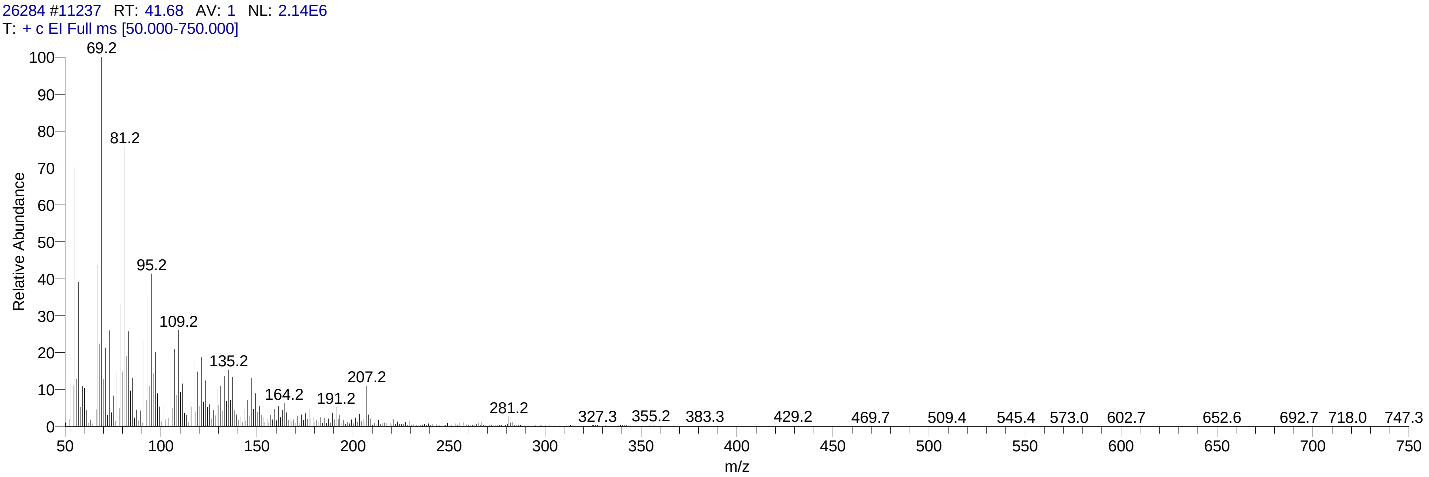

RT	Compound Name	Area %	MF	Molecular Formula	Molecular Weight	Cas #	Library
41.68	1-Heptatriacotanol	0.44	855	C37H76O	536	105794-58-9	mainlib
41.68	Tricyclo[20.8.0.0(7,16)]triacontane, 1(22),7(16)-diepoxy-	0.44	812	C30H52O2	444	NA	mainlib
41.68	TRICYCLO[20.8.0.0E7,16]TRIACONTAN, 1(22),7(16)-DIEPOXY-	0.44	812	C30H52O2	444	NA	WileyRegistry8e
41.68	Thunbergol	0.44	837	C20H34O	290	25269-17-4	mainlib
41.68	2,2,4-Trimethyl-3-(3,8,12,16-tetramethyl-heptadeca-3,7,11,15-tetraenyl)-cyclohexanol	0.44	766	C30H52O	428	NA	mainlib

Compound Structure	Hit Spectrum

1-Heptatriacotanol
Formula C37H76O, MW 536, CAS# 105794-58-9, Entry# 7279
1-Heptatriacontanol #

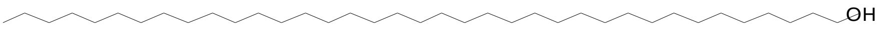

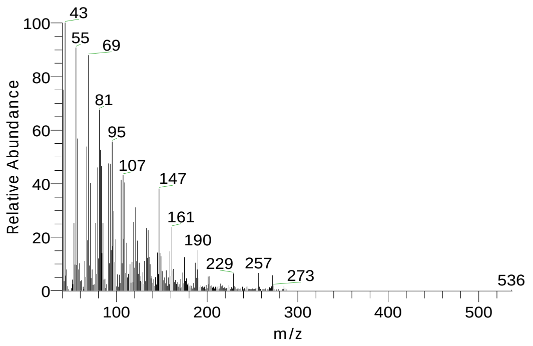

Tricyclo[20.8.0.0(7,16)]triacontane, 1(22),7(16)-diepoxy-
Formula C30H52O2, MW 444, CAS# NA, Entry# 20028
$:28XVGPDAFFXRGERF-UHFFFAOYSA-N

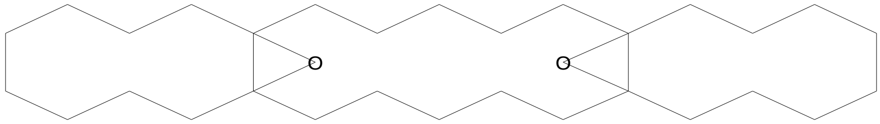

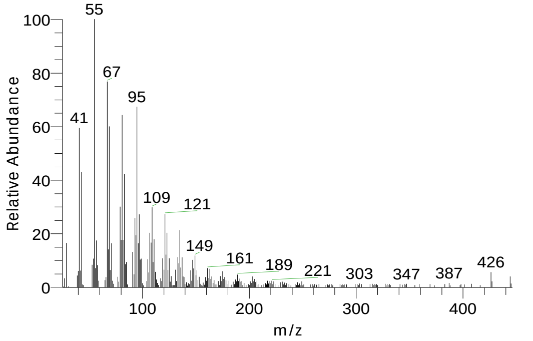

My GC-MS Report

Compound Structure | Hit Spectrum

TRICYCLO[20.8.0.0E7,16]TRIACONTAN, 1(22),7(16)-DIEPOXY-
Formula C30H52O2, MW 444, CAS# NA, Entry# 388503

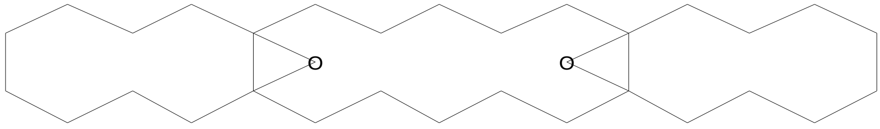

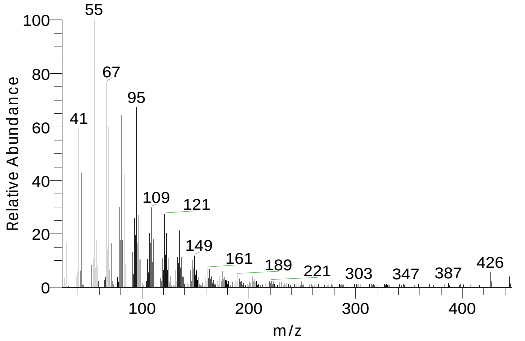

Thunbergol
Formula C20H34O, MW 290, CAS# 25269-17-4, Entry# 9470
(1R,2E,4S,7E,11E)-4-Isopropyl-1,7,11-trimethylcyclotetradeca-2,7,11-trienol

SI 759, RSI 837, mainlib, Entry# 9470, CAS# 25269-17-4, Thunbergol

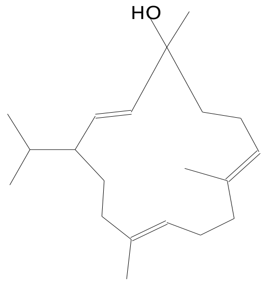

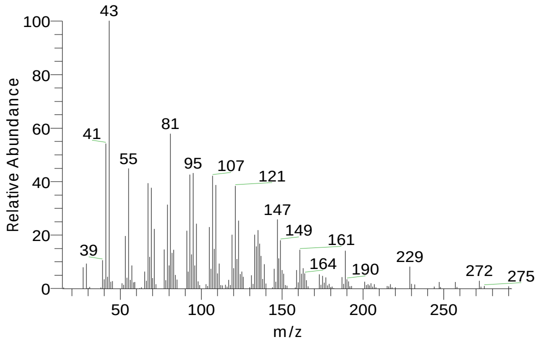

2,2,4-Trimethyl-3-(3,8,12,16-tetramethyl-heptadeca-3,7,11,15-tetraenyl)-cyclohexanol
Formula C30H52O, MW 428, CAS# NA, Entry# 35374

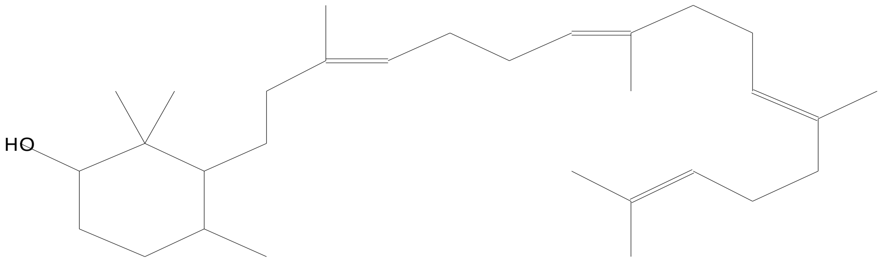

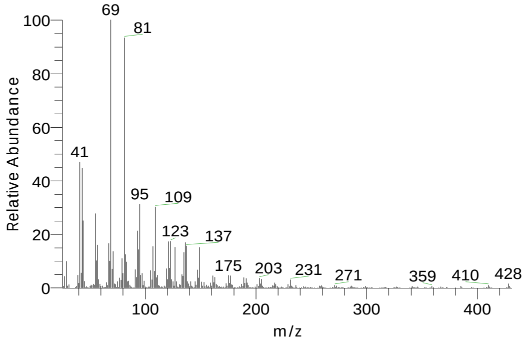

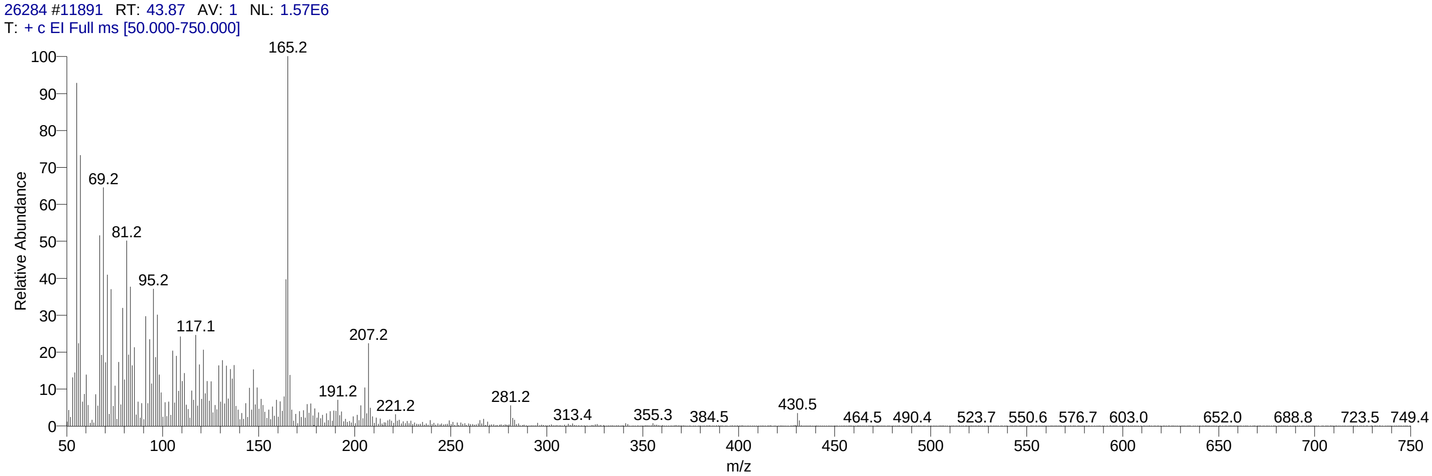

RT	Compound Name	Area %	MF	Molecular Formula	Molecular Weight	Cas #	Library
43.87	1-Heptatriacotanol	0.51	803	C37H76O	536	105794-58-9	mainlib
43.87	Ethyl iso-allocholate	0.51	759	C26H44O5	436	NA	mainlib
43.87	ETHYL ISO-ALLOCHOLATE	0.51	758	C26H44O5	436	NA	WileyRegistry8e

My GC-MS Report

RT	Compound Name	Area %	MF	Molecular Formula	Molecular Weight	Cas #	Library
43.87	01297107001 TETRANEURIN - A - DIOL	0.51	765	C15H20O5	280	NA	WileyRegistry8e
43.87	HEXADECADIENOIC ACID, METHYL ESTER	0.51	749	C17H30O2	266	29961-54-4	WileyRegistry8e

Compound Structure | Hit Spectrum

1-Heptatriacotanol
Formula C37H76O, MW 536, CAS# 105794-58-9, Entry# 7279
1-Heptatriacontanol #

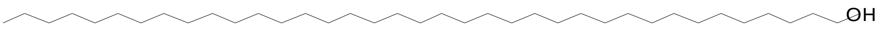

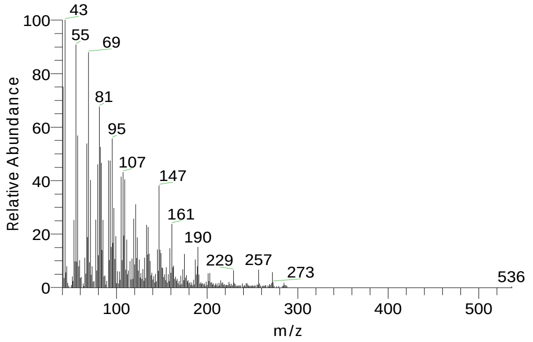

Ethyl iso-allocholate
Formula C26H44O5, MW 436, CAS# NA, Entry# 7020
Ethyl 3,7,12-trihydroxycholan-24-oate #

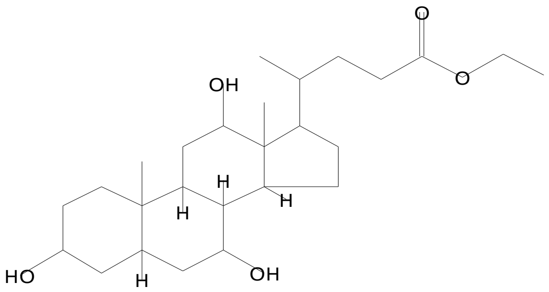

SI 754, RSI 759, mainlib, Entry# 7020, CAS# NA, Ethyl iso-allocholate

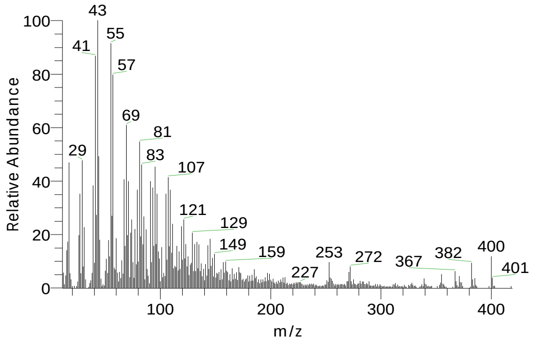

ETHYL ISO-ALLOCHOLATE
Formula C26H44O5, MW 436, CAS# NA, Entry# 270212

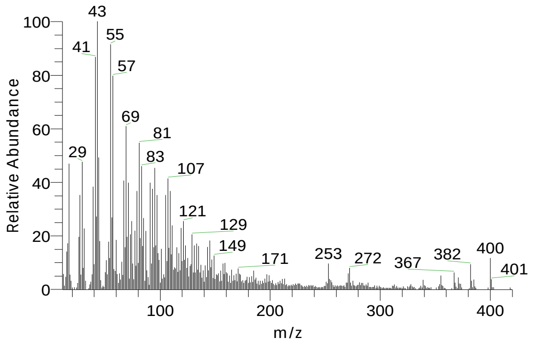

01297107001 TETRANEURIN - A - DIOL
Formula C15H20O5, MW 280, CAS# NA, Entry# 170378

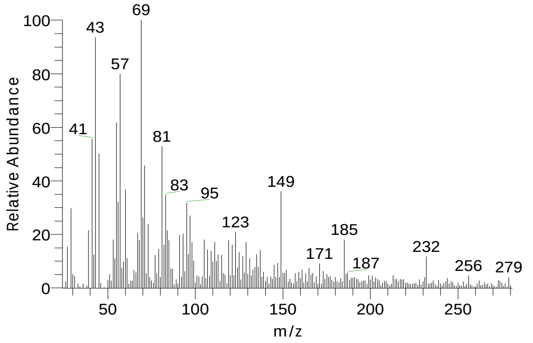

My GC-MS Report

Compound Structure	Hit Spectrum

HEXADECADIENOIC ACID, METHYL ESTER
Formula C17H30O2, MW 266, CAS# 29961-54-4, Entry# 157129
METHYL HEXADECADIENOATE

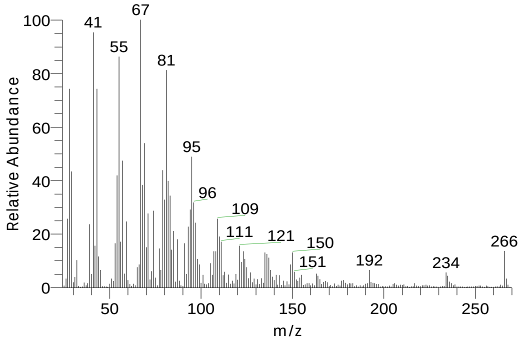

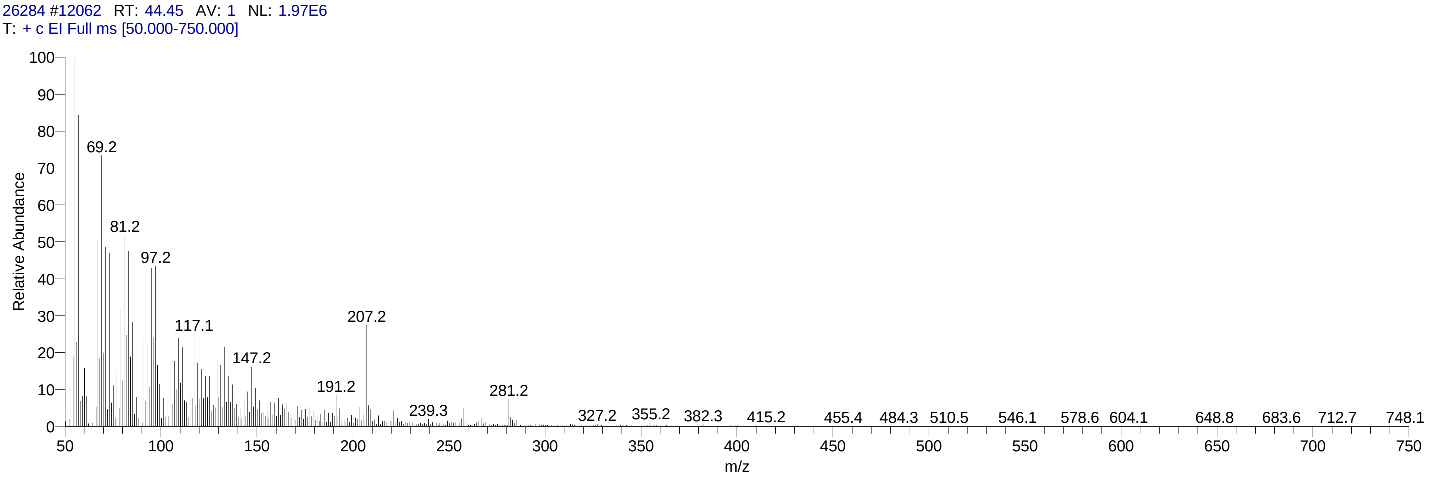

RT	Compound Name	Area %	MF	Molecular Formula	Molecular Weight	Cas #	Library
44.45	Z-(13,14-Epoxy)tetradec-11-en-1-ol acetate	0.52	772	C16H28O3	268	NA	mainlib
44.45	2-HYDROXY-3-[(9E)-9-OCTADECENOYLOXY]PROPYL (9E)-9-OCTADECENOATE #	0.52	744	C39H72O5	620	2465-32-9	WileyRegistry8e
44.45	11-OCTADECENAL (SPECTRUM DISAGREES)	0.52	746	C18H34O	266	56554-95-1	WileyRegistry8e
44.45	12-Methyl-E,E-2,13-octadecadien-1-ol	0.52	795	C19H36O	280	NA	mainlib
44.45	Glycidyl oleate	0.52	757	C21H38O3	338	NA	mainlib

Compound Structure	Hit Spectrum

Z-(13,14-Epoxy)tetradec-11-en-1-ol acetate
Formula C16H28O3, MW 268, CAS# NA, Entry# 10459
(11Z)-12-(2-Oxiranyl)-11-dodecenyl acetate #

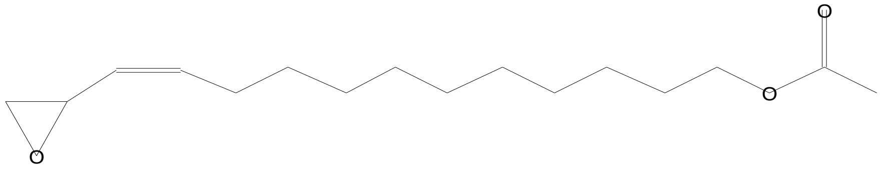

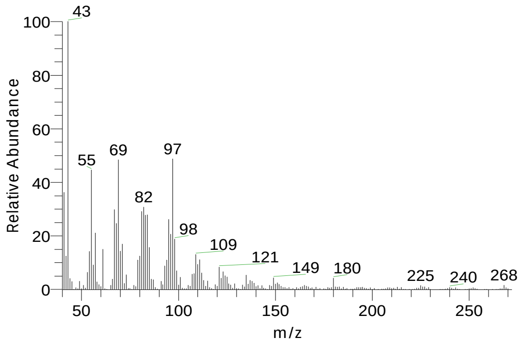

My GC-MS Report

Compound Structure | Hit Spectrum

2-HYDROXY-3-[(9E)-9-OCTADECENOYLOXY]PROPYL (9E)-9-OCTADECENOATE #
Formula C39H72O5, MW 620, CAS# 2465-32-9, Entry# 298152
(Z,Z)-1,3-DIOCTADECENOYL GLYCEROL

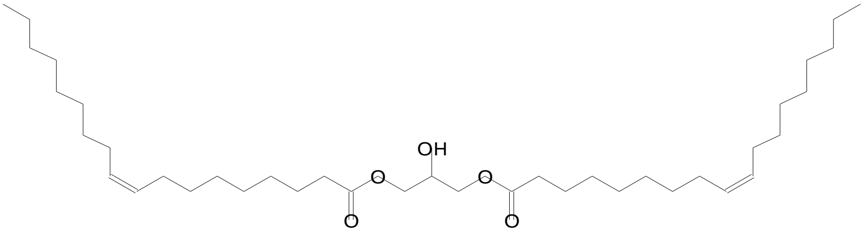

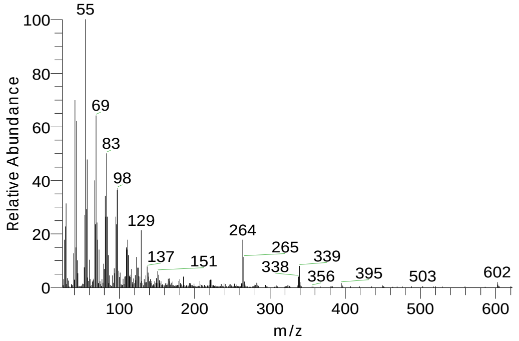

11-OCTADECENAL (SPECTRUM DISAGREES)
Formula C18H34O, MW 266, CAS# 56554-95-1, Entry# 157333
11-OCTADECENAL

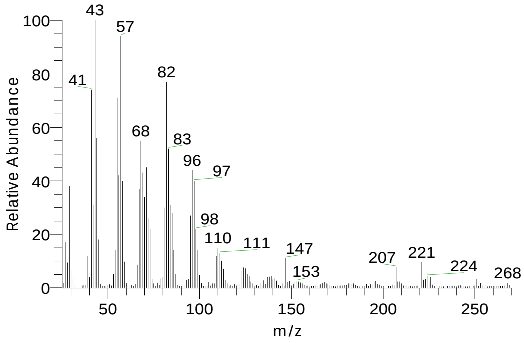

12-Methyl-E,E-2,13-octadecadien-1-ol
Formula C19H36O, MW 280, CAS# NA, Entry# 19016
(2E,15Z)-14-Methyl-2,15-octadecadien-1-ol #

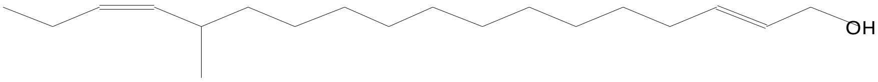

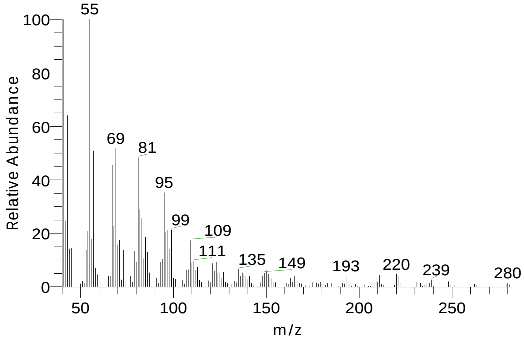

Glycidyl oleate
Formula C21H38O3, MW 338, CAS# NA, Entry# 112970
$:28VWYIWOYBERNXLX-KTKRTIGZSA-N

SI 722, RSI 757, mainlib, Entry# 112970, CAS# NA, Glycidyl oleate

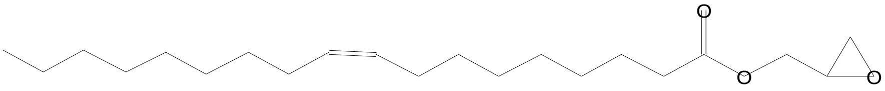

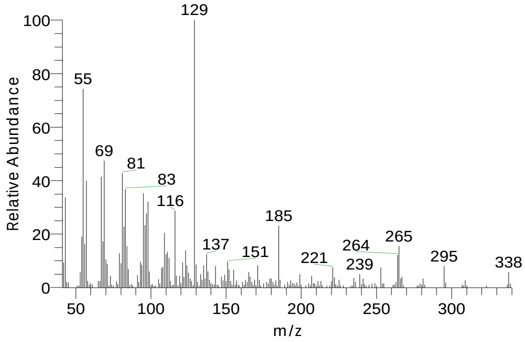

My GC-MS Report

26284 #12136 RT: 44.70 AV: 1 NL: 1.94E6
T: + c EI Full ms [50.000-750.000]

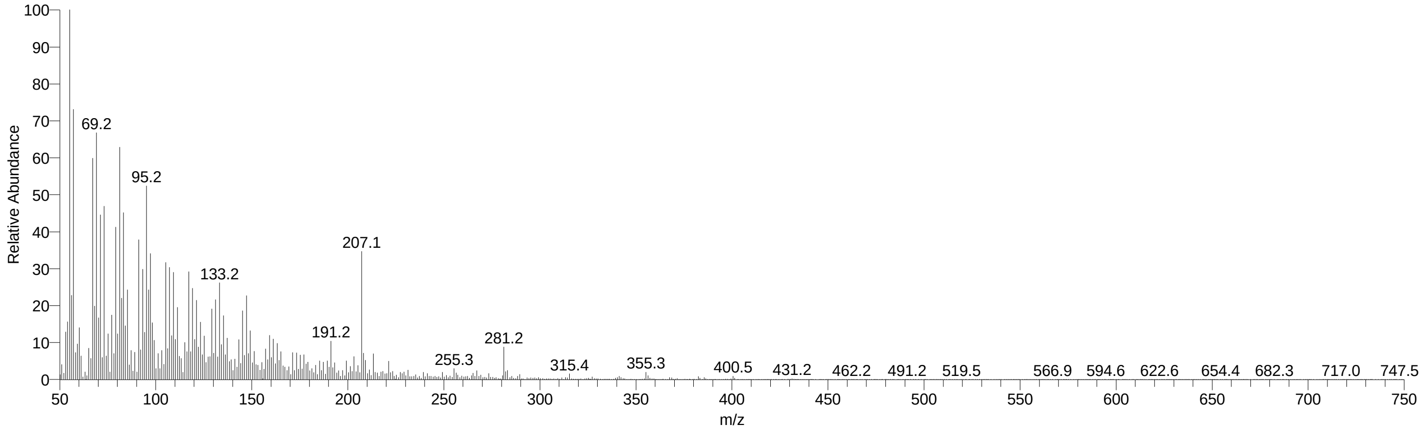

RT	Compound Name	Area %	MF	Molecular Formula	Molecular Weight	Cas #	Library
44.70	1-Heptatriacotanol	0.70	845	C37H76O	536	105794-58-9	mainlib
44.70	Tricyclo[20.8.0.0(7,16)]triacontane, 1(22),7(16)-diepoxy-	0.70	808	C30H52O2	444	NA	mainlib
44.70	TRICYCLO[20.8.0.0E7,16]TRIACONTAN, 1(22),7(16)-DIEPOXY-	0.70	808	C30H52O2	444	NA	WileyRegistry8e
44.70	TRIDEUTERIOMETHYL 10-EPOXY-7-ETHYL-3,11-DIMETHYLTRIDECA-2,6-DIENOATE	0.70	765	C18H27D3O3	297	56805-11-9	WileyRegistry8e
44.70	Linoleic acid ethyl ester	0.70	732	C20H36O2	308	544-35-4	replib

Compound Structure	Hit Spectrum

1-Heptatriacotanol
Formula C37H76O, MW 536, CAS# 105794-58-9, Entry# 7279
1-Heptatriacontanol #

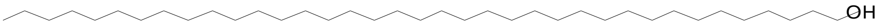

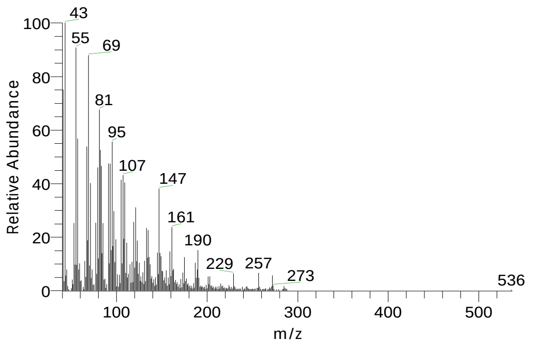

Tricyclo[20.8.0.0(7,16)]triacontane, 1(22),7(16)-diepoxy-
Formula C30H52O2, MW 444, CAS# NA, Entry# 20028
$:28XVGPDAFFXRGERF-UHFFFAOYSA-N

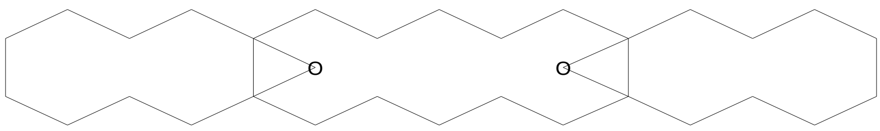

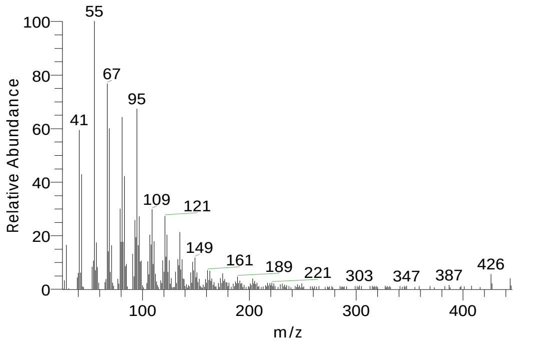

My GC-MS Report

Compound Structure | Hit Spectrum

TRICYCLO[20.8.0.0E7,16]TRIACONTAN, 1(22),7(16)-DIEPOXY-
Formula C30H52O2, MW 444, CAS# NA, Entry# 388503

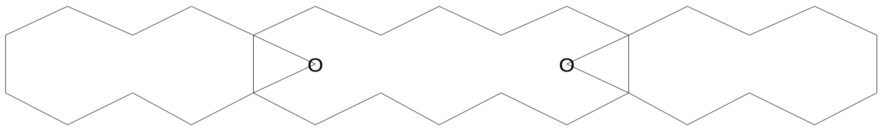

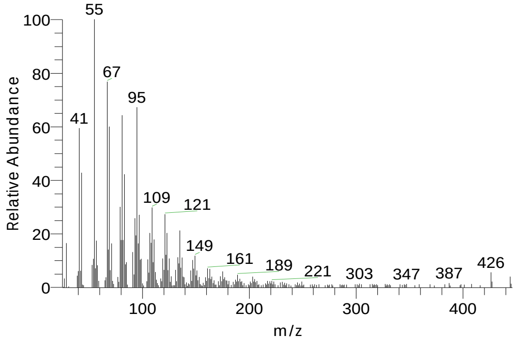

TRIDEUTERIOMETHYL 10-EPOXY-7-ETHYL-3,11-DIMETHYLTRIDECA-2,6-DIENOATE
Formula C18H27D3O3, MW 297, CAS# 56805-11-9, Entry# 184042

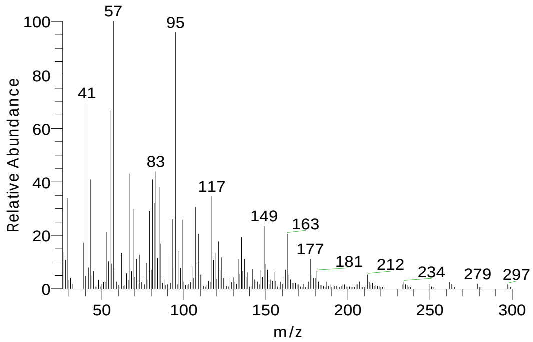

Linoleic acid ethyl ester
Formula C20H36O2, MW 308, CAS# 544-35-4, Entry# 8097
Ethyl linoleate

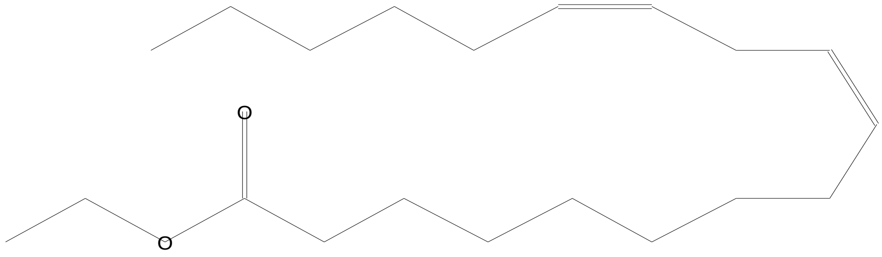

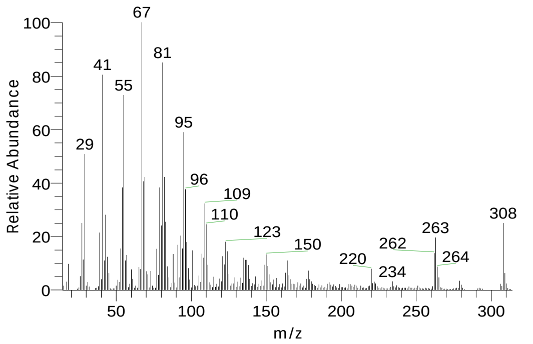

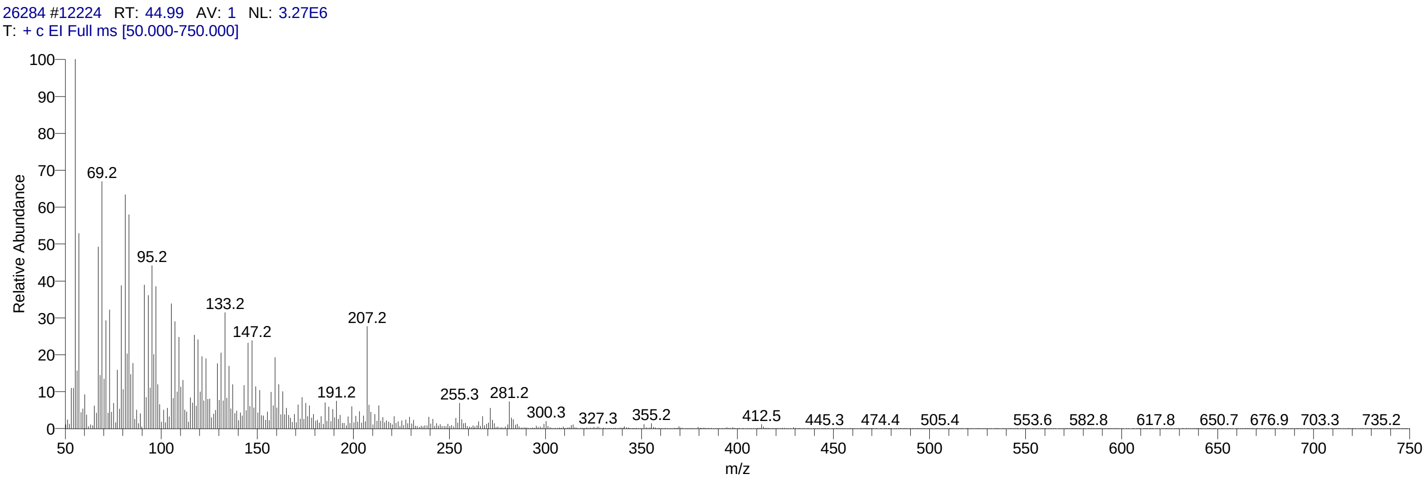

RT	Compound Name	Area %	MF	Molecular Formula	Molecular Weight	Cas #	Library
44.99	TRIDEUTERIOMETHYL 10-EPOXY-7-ETHYL-3,11-DIMETHYLTRIDECA-2,6-DIENOATE	2.13	772	C18H27D3O3	297	56805-11-9	WileyRegistry8e

My GC-MS Report

RT	Compound Name	Area %	MF	Molecular Formula	Molecular Weight	Cas #	Library
44.99	2-HYDROXY-3-[(9E)-9-OCTADECENOYLOXY]PROPYL (9E)-9-OCTADECENOATE #	2.13	722	C39H72O5	620	2465-32-9	WileyRegistry8e
44.99	9-OCTADECENOIC ACID (Z)-	2.13	722	C18H34O2	282	112-80-1	WileyRegistry8e
44.99	Gorgost-5-en-3-ol, (3á)-	2.13	765	C30H50O	426	29782-65-8	mainlib
44.99	GORGOST-5-EN-3-OL, (3á)-	2.13	760	C30H50O	426	29782-65-8	WileyRegistry8e

Compound Structure | Hit Spectrum

TRIDEUTERIOMETHYL 10-EPOXY-7-ETHYL-3,11-DIMETHYLTRIDECA-2,6-DIENOATE
Formula C18H27D3O3, MW 297, CAS# 56805-11-9, Entry# 184042

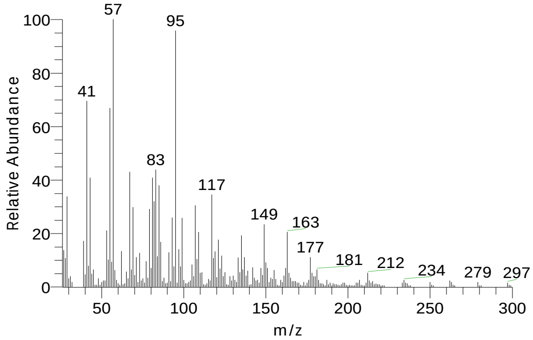

2-HYDROXY-3-[(9E)-9-OCTADECENOYLOXY]PROPYL (9E)-9-OCTADECENOATE #
Formula C39H72O5, MW 620, CAS# 2465-32-9, Entry# 298152
(Z,Z)-1,3-DIOCTADECENOYL GLYCEROL

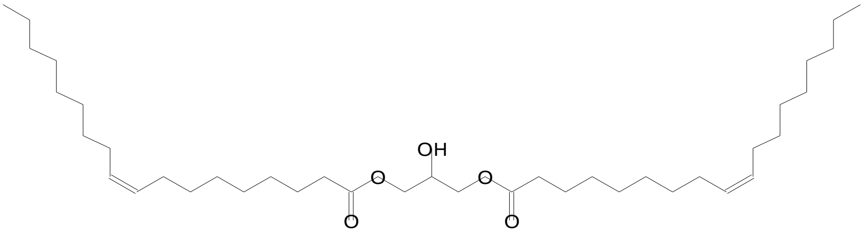

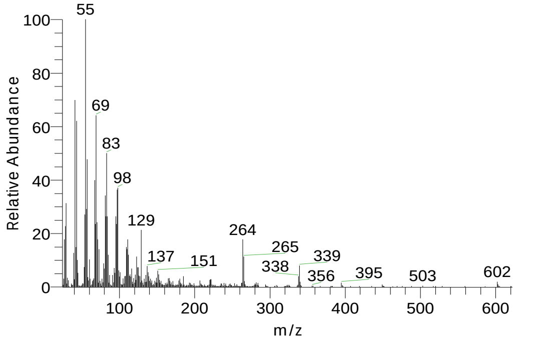

9-OCTADECENOIC ACID (Z)-
Formula C18H34O2, MW 282, CAS# 112-80-1, Entry# 172910
OCTADEC-9-ENOIC ACID

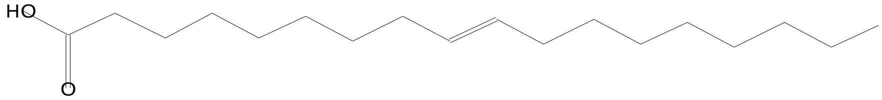

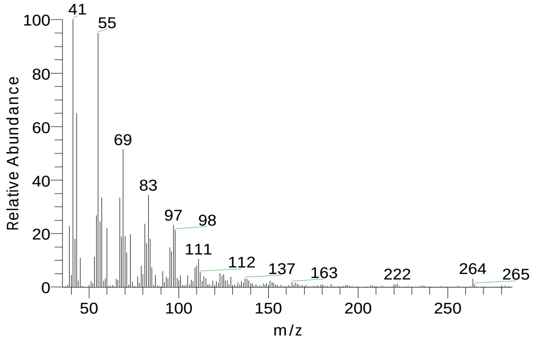

Gorgost-5-en-3-ol, (3á)-
Formula C30H50O, MW 426, CAS# 29782-65-8, Entry# 19693
Pregn-5-en-3á-ol, 20à-[(1R,2R)-2-(1R)-1,2-dimethylpropyl-2-methylcyclopropyl]-

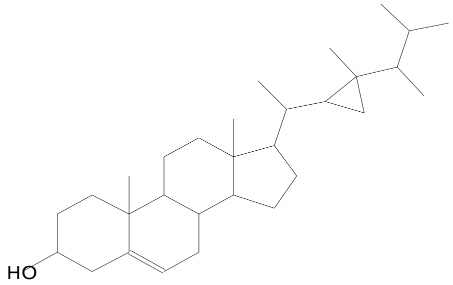

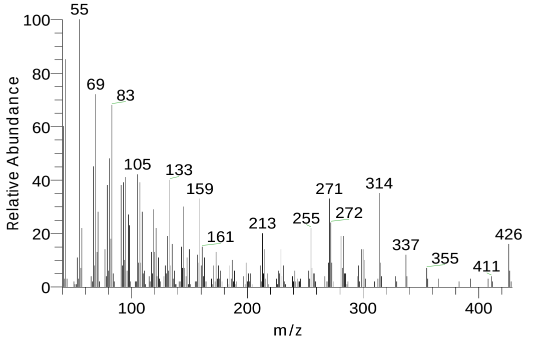

My GC-MS Report

Compound Structure

GORGOST-5-EN-3-OL, (3á)-
Formula C30H50O, MW 426, CAS# 29782-65-8, Entry# 266749
GORGOST-5-EN-3-OL #

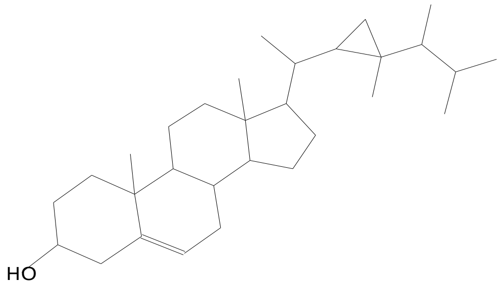

Hit Spectrum

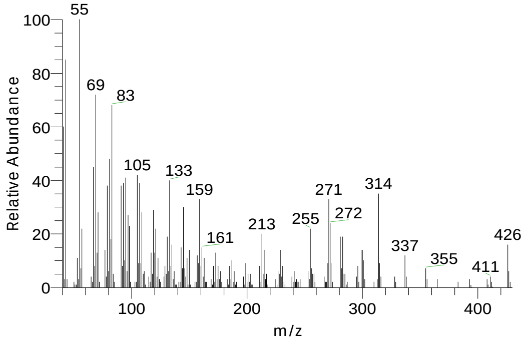

Supplement: Supplementary file 3 [file DataSheet1.pdf]
